# Origin and Evolution of Rickettsial Plasmids

**DOI:** 10.1371/journal.pone.0147492

**Published:** 2016-02-11

**Authors:** Khalid El Karkouri, Pierre Pontarotti, Didier Raoult, Pierre-Edouard Fournier

**Affiliations:** 1 Unité de Recherche en Maladies Infectieuses et Tropicales Emergentes (URMITE), UM63, CNRS7278, IRD198, INSERMU1095, Institut Hospitalo-Universitaire Méditerranée-Infection, Aix-Marseille Université, Faculté de Médecine, 27 boulevard Jean Moulin, 13385 Marseille cedex 5, France; 2 Aix Marseille Université, CNRS, Centrale Marseille, I2M UMR 7373, Equipe Evolution Biologique et Modélisation, Marseille, France; Academia Sinica, TAIWAN

## Abstract

**Background:**

*Rickettsia* species are strictly intracellular bacteria that have undergone a reductive genomic evolution. Despite their allopatric lifestyle, almost half of the 26 currently validated *Rickettsia* species have plasmids. In order to study the origin, evolutionary history and putative roles of rickettsial plasmids, we investigated the evolutionary processes that have shaped 20 plasmids belonging to 11 species, using comparative genomics and phylogenetic analysis between rickettsial, microbial and non-microbial genomes.

**Results:**

Plasmids were differentially present among *Rickettsia* species. The 11 species had 1 to 4 plasmid (s) with a size ranging from 12 kb to 83 kb. We reconstructed pRICO, the last common ancestor of the current rickettsial plasmids. pRICO was vertically inherited mainly from *Rickettsia/Orientia* chromosomes and diverged vertically into a single or multiple plasmid(s) in each species. These plasmids also underwent a reductive evolution by progressive gene loss, similar to that observed in rickettsial chromosomes, possibly leading to cryptic plasmids or complete plasmid loss. Moreover, rickettsial plasmids exhibited ORFans, recent gene duplications and evidence of horizontal gene transfer events with rickettsial and non-rickettsial genomes mainly from the α/γ-proteobacteria lineages. Genes related to maintenance and plasticity of plasmids, and to adaptation and resistance to stress mostly evolved under vertical and/or horizontal processes. Those involved in nucleotide/carbohydrate transport and metabolism were under the influence of vertical evolution only, whereas genes involved in cell wall/membrane/envelope biogenesis, cycle control, amino acid/lipid/coenzyme and secondary metabolites biosynthesis, transport and metabolism underwent mainly horizontal transfer events.

**Conclusion:**

Rickettsial plasmids had a complex evolution, starting with a vertical inheritance followed by a reductive evolution associated with increased complexity via horizontal gene transfer as well as gene duplication and genesis. The plasmids are plastic and mosaic structures that may play biological roles similar to or distinct from their co-residing chromosomes in an obligate intracellular lifestyle.

## Introduction

*Rickettsia* species (Order *Rickettsiales*, Family *Rickettsiaceae*) are obligate intracellular α-proteobacteria associated with diverse eukaryotic hosts. The genus *Rickettsia* emerged approximately 150 million years ago after several transitions from a presumably free-living ancestor of *Rickettsiales* to an intracellular life and then to primarily infecting arthropod lineages approx. 525–425 million years ago [1, 2]. Rickettsiae can infect humans or animals, mostly through arthropod bites, and can cause a range of mild to fatal diseases such as epidemic typhus and Rocky Mountain spotted fever. Twenty-six species currently have standing in the nomenclature, but many isolates await taxonomic classification [3]. *Rickettsia* species diverged into three phylogenetic groups: the spotted fever group (SFG) associated with ticks, fleas and mites and causing spotted fevers, the typhus group (TG) including *R*. *prowazekii* and *R*. *typhii* associated with body lice and rat fleas, respectively, and causing typhus, and the so-called ancestral group (AG) containing *R*. *bellii* and *R*. *canadensis*, associated with ticks [4,5]. However, recent studies have showed that other *Rickettsia* lineages exist, notably associated with protists, leeches or arthropods [1, 2].

Deciphering rickettsial genomes revealed unexpected genetic and evolutionary features. These bacteria have small genomes (1.1–1.8 Mbp) that have undergone a reductive evolution, possibly in relation to their strict intracellular lifestyle in which some metabolic pathways are progressively lost as host cells provide the missing metabolites. Consequences of this progressive genomic reduction included: i) an enrichment in A+T content, polyA/T homopolymers, conjugative clusters and mechanisms of adhesion to host and motility; ii) a high degree of inter-species genomic synteny; iii) an increased virulence; and iv) a variable distribution of plasmids [2, 6–11]. Although initially thought to be devoid of plasmids, such mobile genetic elements (MGEs) have now been identified in at least 11 *Rickettsia* species. In addition, these plasmids may be diversely distributed among strains of a species, some strains having no, one or several plasmids [12–22]. Finally, the presence of plasmids may also vary according to the passage history or intra-specific variability, as was observed in *R*. *akarii* and *R*. *bellii* [21, 22]. However, the mechanisms underlying the observed differences in plasmid content among species and strains remain unknown.

Rickettsial plasmids (RPs) were first detected in *R*. *felis* in which two isoforms of the putative conjugative pRf plasmid were identified [14]. Based on phylogenomic analysis, the genes in the *R*. *felis* pRf plasmid were suggested to have been acquired through a single horizontal gene transfer event in *R*. *felis* or in an ancestor before the divergence of the *R*. *felis/ R*. *akari* clade [14, 17]. Furthermore, on the basis of the presence of the pRf plasmid and one unpublished sequence of a putative plasmid in *R*. *bellii* OSU85-389, it was hypothesized that the primitive rickettsial ancestor harbored a plasmid system that was lost in certain lineages [23]. Moreover, Baldridge et al. [21, 22] suggested multiple possible origins of RPs as well as likely horizontal transfers from plasmids to chromosomes and *vice versa* using phylogenetic analyses of the *par*A, *hsp*1 and *hsp*2 genes.

However, to date, most of the studies on RPs have been based on a limited number of chromosomic and/or plasmidic sequences. Thus, the putative origin(s) and evolutionary processes of RPs remain mostly unknown. As recent studies have demonstrated that plasmids, not virus, are key vectors of genetic exchanges between bacterial chromosomes, even between distant phylogenetic groups [24, 25], we accepted the opportunity provided by the expanded number of publicly available genomes from arthropod-associated *Rickettsia* species to study the RPs by comparative genomic analysis and to reconstruct the evolutionary scenario of forces that shaped their structures and predicted functions. We inferred the origin, evolution and putative roles of RPs.

## Material and Methods

### Genomic sequences and annotation

Genomic sequences of plasmids and chromosomes (100% and 94% of which are both complete and circular, respectively) belonging to 26 species and 35 strains of *Rickettsia* and *Orientia* genera were downloaded from the NCBI ftp server (ftp://ftp.ncbi.nih.gov/Genome/). These species were collected and identified in diverse arthropod hosts (ticks, insects and fleas) or clinical patients (humans) from Africa, Europe, America, Azia and Australia from 1941 to 2002, and they are members of either spotted fever group (SFG), typhus group (TG) and ancestral groups (AG) rickettsiaes. To avoid potential biases across the originally published and unpublished data, that were generated by different gene identification and annotations, all genomes were subjected to CDSs (CoDing Sequences) predictions with the same AMIgene software [26] and automatic functional re-annotation against the RickBase [17] and non-redundant NR databases using pipRick (an in-house annotation pipeline written in Perl language) including the BLASTp algorithm [27]. The re-annotated plasmids were then manually curated and either complete (coverage > = 80% of the longest homolog), split (gene with at least two CDSs), fragment (coverage <80% of the longest homolog) or chimeric genes were distinguished [14, 17]. Functional classification of gene families (COG ID and Letters) was searched using COGnitor and COGsoft against COG database [28, 29]. Thus, a standardized database named *Rickettsia*DB was constructed for further investigations.

### Comparative genomics and phylogenetics

In order to examine evolutionary relationships between *Rickettsia* plasmids, proteins were subjected to a reciprocical best BLAST hit (BBH) algorithm with all-against-all search (coverage of the query length > = 60% and *E-value* < 10^-5^) using COGsoft software [28, 29]. Each putative orthologous groups of plasmidic rickettsial genes was named pRIGs, and then a manual curation was performed to detect false negatives. A gene content microarray data matrix (pRIGs x plasmids) based on the presence and absence of genes including the degradation levels (complete: red, split: yellow, fragment: blue, chimeric: green, and absent or remnant: violet) was then constructed using TMeV software (http://sigenae.org/index.php?id=88). Sequence datasets of each pRIG and COGID were extracted and packaged into single fasta files for further analysis.

Evolutionary relationships between rickettsial plasmids, *Rickettsia* and *Orientia* chromosomes as well as microbial and non-microbial genomes were, first, examined using BLASTp and tBLASTn algorithms (Cutoffs: aa sequence identity > = 25%, coverage query> = 60%, *E-value*<10^-5^), search against *Rickettsia*DB (excluding plasmid sequences), non-redundant NR and RefSeq_genomic databases to identify their corresponding best homologs. The genes which did not exhibit, any significant hit neither with rickettsial chromosomes nor with other genomes from NCBI databases and having an *E-value*>0.01, were considered as ORFans. New visually inspected datasets that gathered sequences of rickettsial plasmids (each pRIG and COGID) and their corresponding homologs were downloaded and packaged into single FASTA files to perform phylogenetic analysis. Split genes were concatenated while highly degraded sequences (either very short fragments or remnants) were removed. Moreover, redundant sequences of the same gene from different BLAST analysis were removed to retain some representatives. Multiple sequence alignments were carried out using CLUSTALX and/or MUSCLE applications [30, 31]. Phylogenetic trees were computed with MEGA version 6 [32]. Both Neighbor-Joining (NJ) and Maximum Likelihood (ML) methods, respectively, under the JTT amino acid substitution matrix and the WAG model plus the Nearest-Neighbor-Interchange (NNI) were examined. Each tree was constructed by examining the following parameters: uniform sites, gamma (Γ) distribution of parameter α to account for substitution rate heterogeneity among sites and deletion either complete or partial (> = 90%). The robustness of the tree nodes was estimated by Bootstrap Percentage (BP) using 1000 and 100 replicates for NJ and ML, respectively. For each pRIG, either single or multiple phylogeny (ies) were examined to retain a consistent dataset for both evolutionary methods.

The evolutionary events including vertical and horizontal gene transfers (VGT and HGT) were identified using distinct cutoffs from best BLAST hits and/or their BP confidence in each phylogenetic trees as follows: VGT (proteins with 28> = aa identity < = 100%), evident HGT (aa identity > = 50% and BP> = 60) and probable HGT (25> = aa identity < = 80%). The recent duplications (*i*.*e*., inparalogs after the divergence of each species) were identified in phylogenetic trees or in case of ORFans by BLASTp with an identity cutoff > = 80%. To perform plasmid grouping and their putative evolutionary origins, the supertree algorithm based on the subtree prune-and-regraft distance [33] was also used from NJ trees of 10 selected genes present in most plasmids. The phylogenetic results were summarized into evolutionary networks between the rickettsial plasmids and microbial as well as non-microbial genomes using CYTOSCAPE software [34]. In the networks, the nodes represent the genes while the edges represent the links connecting them. Last, complete sequences of all plasmids were started from the *dna*A-like gene and ended with the *par*A and some unknown genes, and then multiple pairwise alignments (cutoff: *Evalue*<10^-5^) were performed according to plasmid grouping using BLASTn and viewed under genoPlotR tool [35].

### Statistical analysis

The correlation between *Rickettsia* plasmids and chromosomes sizes was performed using the correlation coefficient R of Pearson and the coefficient of determination R². Statistical analyses were performed using the R Commander software package (http://r-forge.r-project.org).

## Results/Discussion

### RPs are in a process of reductive evolution

**Fig 1:**
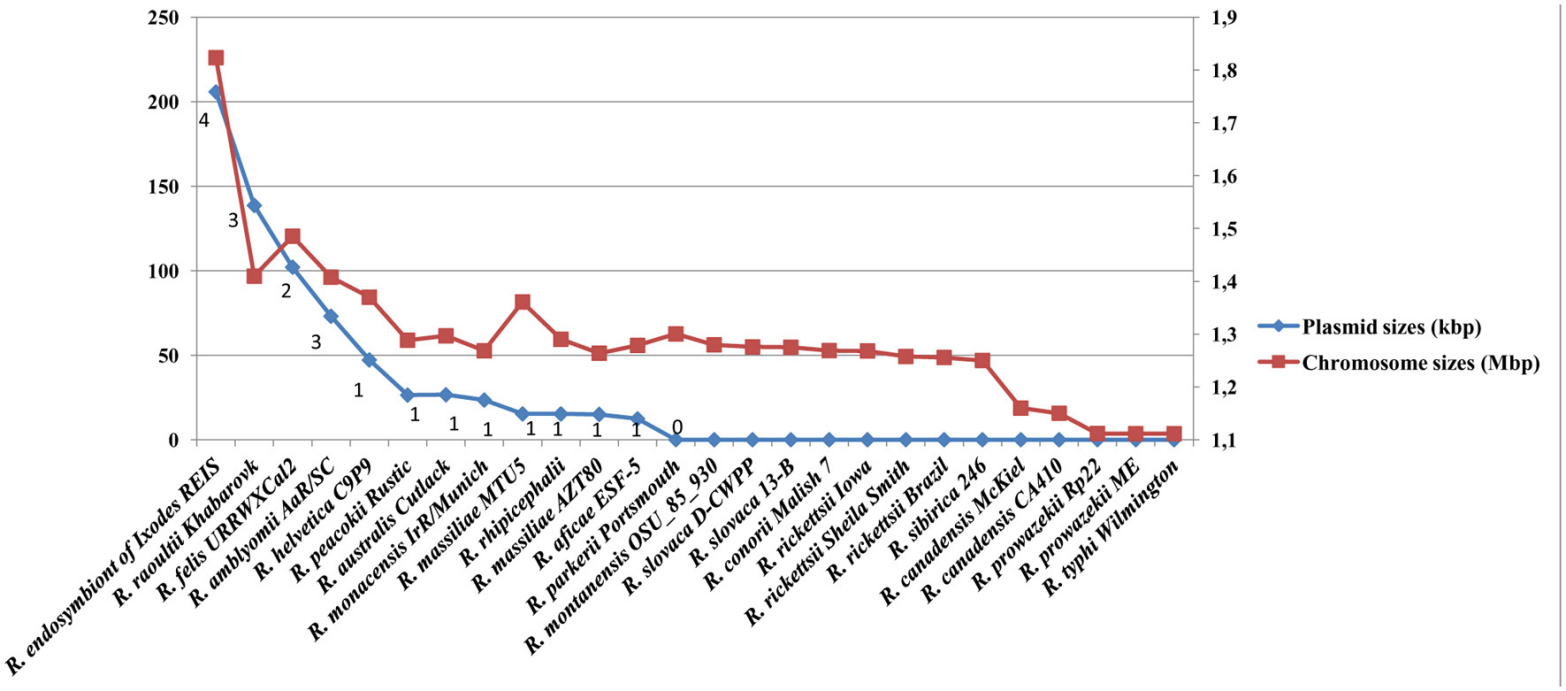
Rickettsial plasmid sizes (pooled) and numbers plotted with their chromosome sizes.

A total of 20 plasmids occurred in 11 SFG *Rickettsia* species, represented by 13 strains collected from various geographical locations worldwide (Table 1). In contrast, 9 SFG and TG species, represented by 15 strains, had no plasmids (Table 1). These included *R*. *conorii*, *R*. *montanensis*, *R*. *parkeri*, *R*. *rickettsii*, *R sibirica*, *R*. *slovaca*, *R*. *prowazekii*, *R*. *typhi*, and *R*. *canadensis* in which the absence of plasmid was confirmed in at least two strains by whole-genome sequencing (sequences available in GenBank) and/or pulsed-field gel electrophoresis (PFGE) [21,22]. In another three species (*R*. *japonica*, *R*. *philipii* and *R*. *heilongjiangensis*) the absence of plasmid needs to be confirmed in more than one strain. In *R*. *akari* and *R*. *bellii* (two SFG and AG species, respectively), Baldridge et al. [21, 22] detected plasmids in some strains by PFGE, but the currently available genomes (Rak strain Hartford and Rbe strains RML369-C and OSU-89-389) had none. This may be explained by strain differences (*i*. *e*., plasmid loss in nature), as observed in *R*. *africae* [9] or their passage history in cell culture, as observed in *R*. *felis* [36] or other bacteria such as *Borrelia burgdorferi* [37]. Among the other 5 genera within the order *Rickettsiales* (*Anaplasma*, *Ehrlichia*, *Neorickettsia*, *Orientia*, *Wolbachia*), all available genome sequences were devoid of plasmids [23]. RPs ranged in number and size per species from 1 to 4 plasmids and from 12 kb to 83 kb, respectively, and contained 15 to 85 genes (Table A in S1 File). A decrease in number and pooled plasmid size correlated with the decrease in chromosome size in rickettsiae (Fig 1, Figure A in S2 File). Such results had previously been observed in prokaryotes [38]. We did not find any evident correlation between presence, number and/or size of plasmids, and rickettsial host range (Table 1).

**Table 1:** *Rickettsia* and *Orientia* genomes extracted from the National Center for Biotechnology Information (NCBI) database.

Species	Strain	Plasmid (s)	Host	Origin	Year	Rickettsiosis	Accession. No.
*R*. *africae*	ESF-5	pRaf	Tick: *Amblyomma variegatum*	Ethiopia	1966	African tick bite fever	NC_012633–34
*R*. *massiliae*	MTU5	pRma	Tick: *Rhipicephalus turanicus*	France	1990	Unnamed rickettsiosis	NC_009897–900
	AZT80	pRmaB	Tick: *Rhipicephalus sanguineus*	USA	2004		CP003319-20
*R*. *peacokii*	Rustic	pRpe	Tick: *Dermacentor andersoni*	USA	Unknown	Not pathogenic	NC_012730–32
*R*. *monacensis*	IrR/Munich	pRmo	Tick: *Ixodes ricinus*	Germany	1998	Unknown pathogenesis	NC_010927
*R*. *Helvetica*	C9P9	pRhe	Tick: *Ixodes ricinus*	Switzeland	1979	Unnamed rickettsiosis	CM001467-68
*R*. *felis*	URRWXCal2	pRfe/pdRfe	Flea: *Ctenocephalides felis*	USA	1990	Flea spotted fever	NC_007109–11
	RfeI1	pRfeI1	Insect: *Liposcelis botrychophila*	Australia	1997		GQ329881
*C*. *R*. *amblyomii*	AaR/SC	pRam18/23/32	Tick: *Amblyomma americanus*	South Carolina	1999	Unknown pathogenesis	NC_013937–38
*R*. *endosy*. of *Ixodes*	REIS	pReis1/2/3/4	Tick: *Ixodes scapularis*	USA	2008	Unknown pathogenesis	CM000770-73 GG688301-16
*R*. *raoultii*	Khabarovsk	pRra1/2/3	Tick: *Dermacentor silvarum*	Russia	2005	SENLAT	CP010969-72
*R*. *australis*	Cutlack	pRau	Unknown	Unknown	Unknown	Queensland tick typhus	CP003338-39
*R*. *rhipicephalii*	3-7-female6-CWPP	pRrh	Unknown	Unknown	Unknown	Unknown pathogenesis	CP003342-43
*R*. *conorii*	Malish 7	-	Tick: *Rhipicephalus* sp.	South Africa	1946	Mediterranean spotted fever	NC_003103
*R*. *slovaca*	13-B	-	Tick: *Dermacentor* sp.	Slovakia	1968	Tick-borne lymphadenitis	CP002428
	D-CWPP	-	Unknown	Unknown	Unknown		CP003375
*R*. *sibirica*	246	-	Unknown	Russia	Unknown	Siberian tick typhus	NZ_AABW00000000
*R*. *philipii*	364D	-	Unknown	Unknown	Unknown	Unknown pathogenesis	CP003308
*R*. *japonica*	YH	-	Human: *Homo sapiens sapiens*	Japan	1985	Japanese spotted fever	AP011533
*R*. *parkerii*	Portsmouth	-	Human: *Homo sapiens sapiens*	USA	2002	Unnamed rickettsiosis	CP003341
*R*. *montanensis*	OSU_85_930	-	Unknown	Unknown	Unknown	Unknown pathogenesis	CP003340
*R*. *heilongjiangensis*	054	-	Unknown	Unknown	Unknown	Far-Eastern tick-borne rickett.	CP002912
*R*. *rickettsia*	Sheila Smith	-	Human: *Homo sapiens sapiens*	Unknown	Unknown	Rocky mountain spotted fever	NC_009882
	Brazil	-	Unknown	Unknown	Unknown		NC_010263
	Iowa	-	Unknown	Unknown	Unknown	Not pathogenic mutant	CP003305
*R*. *akari*	Hartford	-	Mite:?	USA	Unknown	Rickettsial pox	NC_009881
*R*. *prowazekii*	Rp22	-	Human: *Homo sapiens sapiens*	Algeria	Unknown	Epidemic typhus	CP001584
	Madrid E	-	Human: *Homo sapiens sapiens*	Spain	1941	Not pathogenic mutant	NC_000963
*R*. *typhi*	Wilmington	-	Unknown	Unknown	Unknown	Murine typhus	NC_006142
*R*. *canadensis*	McKiel	-	Tick: *Haemaph*. *leporispalustris*	Canada	Unknown	Unknown pathogenesis	NC_009879
	CA410	-	Unknown	Unknown	Unknown		CP003304
*R*. *bellii*	RML369-C	-	Tick: *Dermacentor varabilis*	USA	1966	Unknown pathogenesis	NC_007940
	OSU-89-389	u. s.	Unknown	Unknown	Unknown		NC_009883
*O*. *tsutsugamushi*	Boryong	-	Human: *Homo sapiens sapiens*	Japan	1995	Scrub typhus	NC_009488.1
	Ikeda	-	Human: *Homo sapiens sapiens*	Japan	1979		NC_010793.1

Data were reported from [2, 3] as well as PATRIC (http://patricbrc.org) and GENBANK (http://www.ncbi.nih.gov) databases. -, species without any plasmid. u.s., unavailabale sequences.

**Fig 2:**
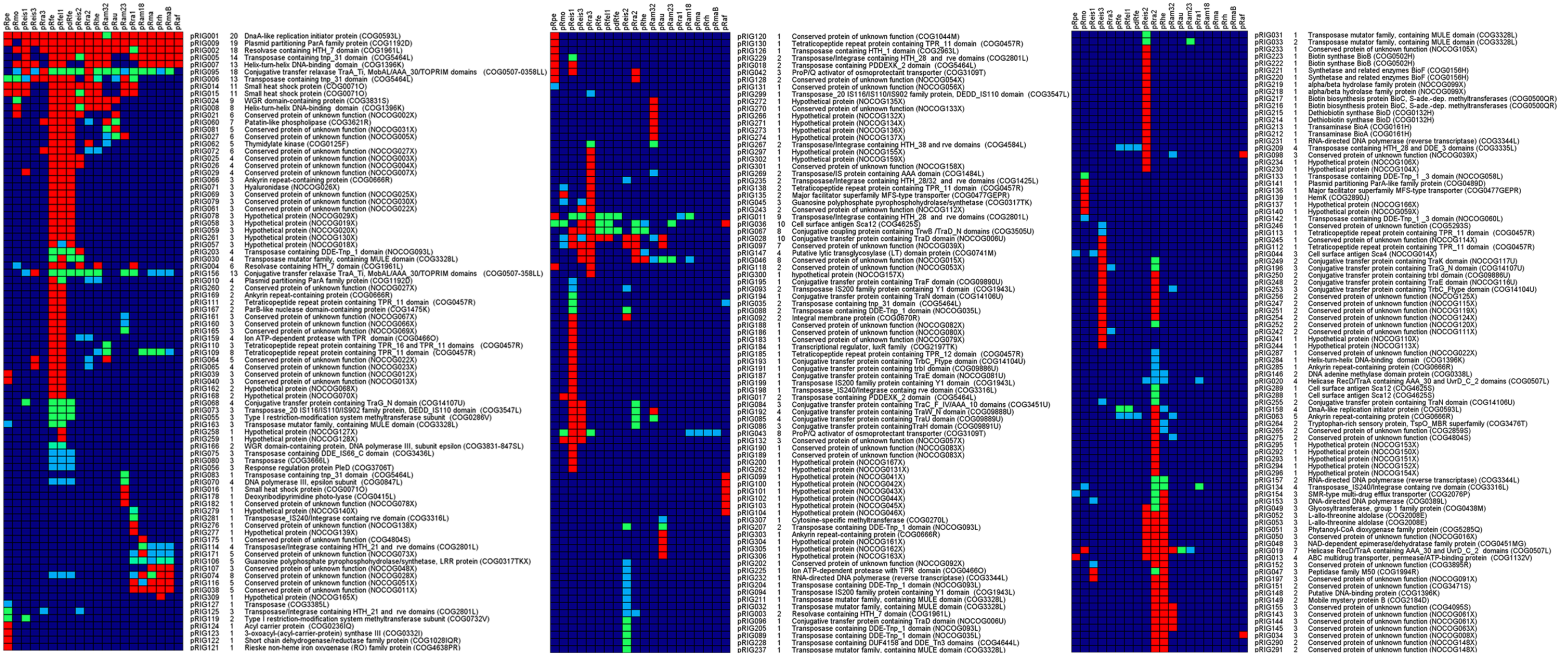
Gene content array of *Rickettsia* plasmidome obtained from BBH analysis. The right three columns showed the pRIG references of putative ortholog gene groups, the total number of genes for each pRIG and its corresponding annotation including its COGID form COG database. Each member of the pRIG was annotated as either complete (red), split (yellow), fragment (blue), chimeric (green) and absent (or remnant, violet).

**Fig 3:**
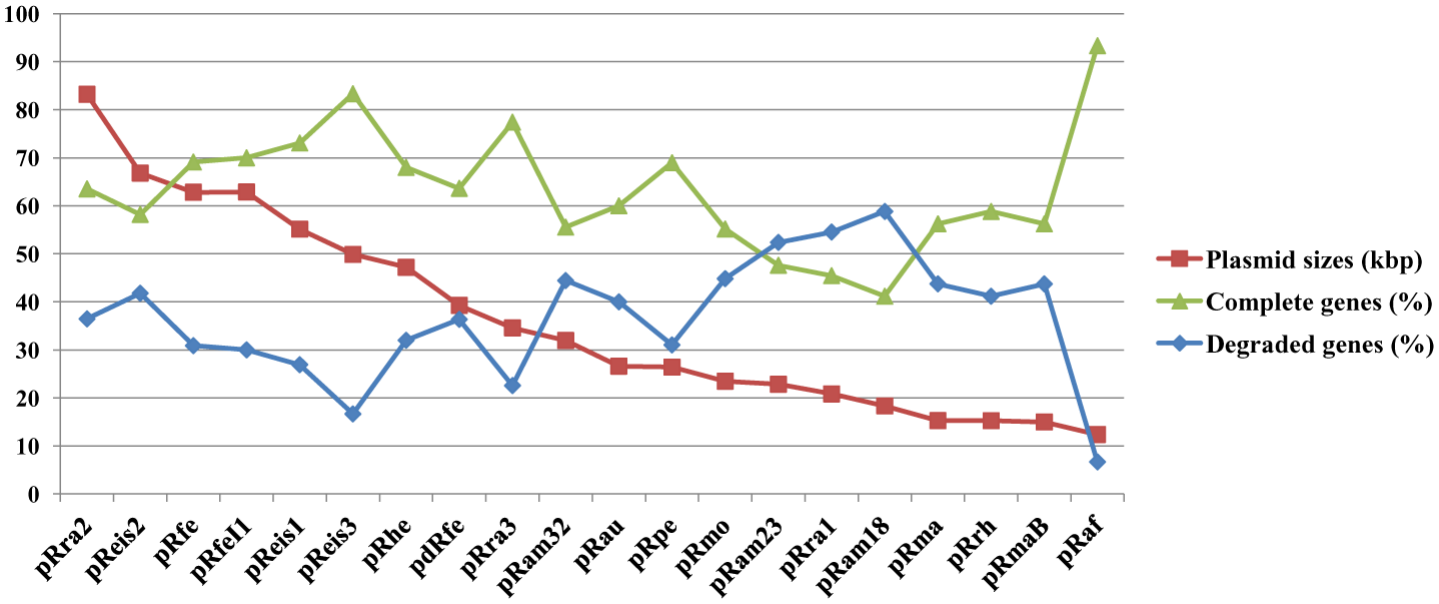
Frequencies of complete and degraded (split, fragment or chimeric) genes identified in the plasmidome of *Rickettsia*.

The plasmidic gene content (plasmidome) counted 260 pRIGs (*i*.*e*., putative orthologous groups of plasmidic rickettsial genes) constructed from 747 protein-coding genes (Fig 2). Of these genes, 65% were full-length genes, whereas 35% were partially degraded (*i*.*e*., either split, fragment or chimerical). These genes can also be either highly degraded remnants, absent or lost in several plasmids. Overall, the degradation levels varied among plasmids, ranging from 16 to 40% in larger plasmids (size >47 kbp) to 44 to 59% in smaller plasmids (<40 kbp), suggesting that RPs are in a progressive degradation process and size reduction (Fig 3). This may also contribute to variations in the coding capacity of the plasmids which ranged from 75 to 92% (Table A in S1 File).

Overall, these data suggest that modern RPs are in a process of reductive evolution similar to that observed in rickettsial chromosomes [17]. Such a phenomenon is typical of symbiotic/parasitic lifestyles [7, 12, 13, 17, 39]. However, this finding is in contrast with the megaplasmids of the free-living *Bacillus cereus* group which are a fusion of smaller plasmids [40]. RPs also exhibited G+C contents ranging from 32 to 35% (Table A in S1 File). Such values are similar to those of the rickettsial chromosomes (Table A in S1 File), suggesting that similar evolutionary forces such as vertical and horizontal gene transfers (VGT and HGT), duplications and selection pressure, impacted both genomic components.

### Origin and divergence of RPs

We performed phylogenetic reconstructions of all 260 pRIGs to quantify the evolutionary processes that may have shaped the 20 current RPs, and to reconstruct the gene content of their last common ancestor and their evolutionary scenarios. The phylogenetic analysis revealed that some pRIGs contained sub-clusters of orthologous genes from multiple origins (see below *e*.*g*., *par*A gene); These sub-clusters were subsequently considered as distinct pRIGs, thus increasing the plasmidome pRIG content from 260 to 275. A total of 136 phylogenetic trees of 184 (67%) pRIGs were retained, for which both neighbor-joining (NJ) and maximum likelihood (ML) methods displayed similar topologies. For the remaining 91 (33%) pRIGs no phylogeny could be inferred.

#### Vertical evolution

**Fig 4:**
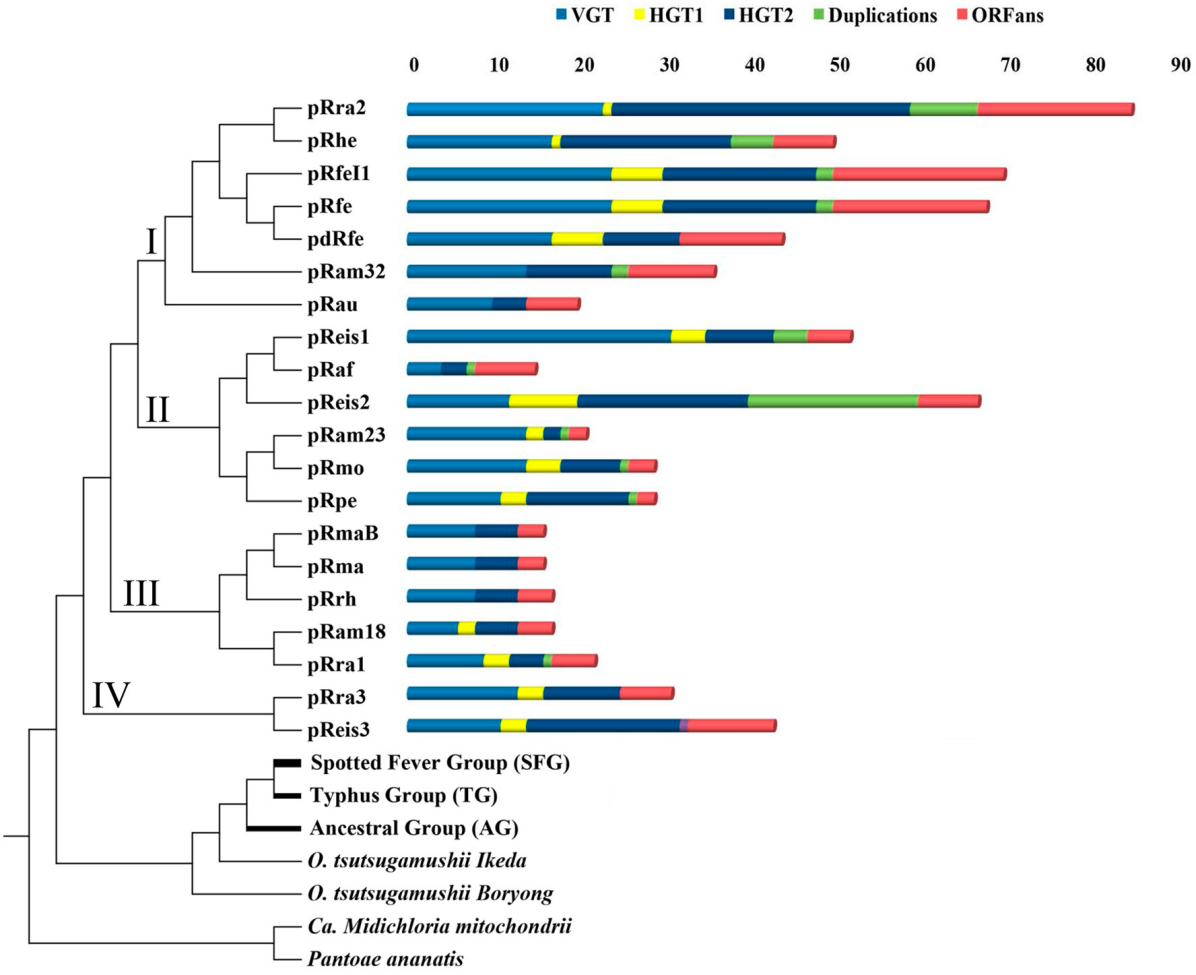
Supertree obtained from 10 genes of *Rickettsia* plasmids as well as *Rickettsia* and *Orientia* chromosomes using subtree prune-and-regraft distance (D = 64). *Candidatus Midichloria mitochondrii* (YP_004679220.1) and *Pantoea ananatis* (YP_005193440.1) were used as outgroups. The list of genes used are two transposases, patatin-like phospholipase, thymidylate kinase, heat shock protein, *dna*A-like replication initiator proteins, helix-turn-helix DNA-binding domain, cell surface antigen Sca12, conjugative transfer protein containing *tra*D domain, and leucine rich-repeat containing protein. In the right, summary of evolutionary events that shaped rickettsial plasmids.

Phylogenetic trees of 12 pRIGs (4.4%), conserved in most plasmids, exhibited two phylogenetically-related clades, one including 5 to 20 RPs and the other made of 1 to 26 chromosomes from *Rickettsia* and/or *Orientia* (Figures A1 to A9 in S3 File). Moreover, the supertree constructed from 10 of the 12 pRIGs confirmed these two phylogenetically-related clades, one containing all the 20 RPs and the other all *Rickettsia*/*Orientia* chromosomes (Fig 4).

This supertree was used as a reference for all following analyses. Phylogenetic analysis of a second set of 41 (15%) pRIGs showed the same two related clusters in each tree, one containing 1 to 11 RPs and the other 1 to 30 *Rickettsia*/*Orientia* chromosomes (Figures A10 to A49 in S3 File). Moreover, the analysis of another four (1.4%) pRIGs (mostly present as fragments), did not result in any reliable phylogeny, but they best matched with homologs in rickettsial chromosomes (data not shown). In all, our results suggest that the current RPs have a vertical origin (VGT) from a last common plasmidic ancestor that we named “pRICO” (Fig 5).

**Fig 5:**
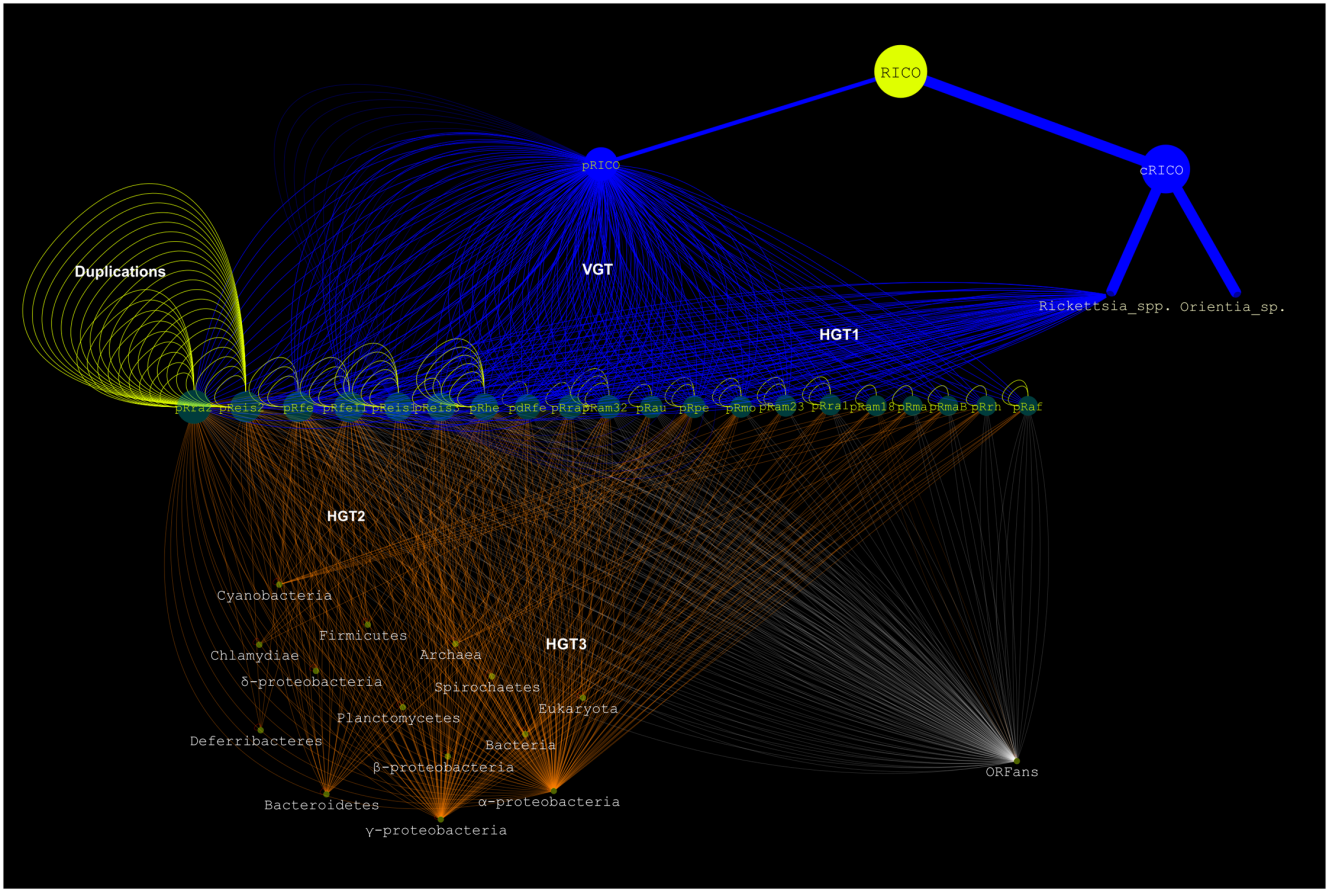
Evolutionary scenario of rickettsial plasmids.

Both “pRICO” and “cRICO” (i.e., the last common ancestor of the current *Rickettsia/Orientia* chromosomes) may have been vertically inherited from a last common chromosomal ancestor, “RICO” (Figs 4 and 5). However, we observed that the internal phylogenetic clades inferred from plasmids and chromosomes were not often identical. This may be due to the facts that i) each species can harbor 1 to 4 plasmid(s) with similar or distinct evolutionary history, ii) both plasmids and chromosomes are in a process of gene degradation and loss, and iii) the selection pressure shaping plasmids and chromosomes may be similar or different in each species and/or between species. Moreover, 8 (3%) pRIGs were also vertically transmitted to RPs but matched non-*Rickettsia/Orientia* species, suggesting that they might have been horizontally acquired by pRICO from unknown species (Figures A50 to A53 in S3 File). Thus, our data strongly support that pRICO harbored around 65 (23%) genes, suggesting that it might have had an estimated size around 60 to 70 kbp (Fig 5). The vertical inheritance is also corroborated by the presence of plasmids in two distinct rickettsial groups (SFG and AG). In *Salmonella enterica* serovars, virulence plasmids were also described to be vertically transmitted [41], although no gene phylogeny and evolutionary relationships were carried out between *Salmonella* plasmids and chromosomes as well as microbial and non-microbial sequenced genomes to infer their origin and evolution.

In the supertree, the pRICO ancestor diverged into a single or multiple plasmid(s) in rickettsial species, and that these plasmids clustered into four putative groups (I to IV) (Fig 4): group I included 4 large and 3 small plasmids of 5 species (pRra2/pRhe/pRfe/pRfeI1/pdRfe/pRam32/pRau); group II clustered 2 large and 4 small plasmids belonging to 5 species (pReis1/pRaf/pReis2/pRam23/pRmo/pRpe); group III contained 5 small plasmids of 4 species (pRam18/pRrh/pRra1/pRma/pRmaB); and group IV gathered one large and one small plasmids from two species (pReis3/pRra3). At the inter-species level, plasmids of the same group showed variable sequence conservations (Fig 4 and Figures BA to BD in S2 File). At the intra-species level, plasmids either belonged to the same group with high sequence conservation, as was the case for *R*. *felis* (pRfe, pdRfe and pRfeI1) and *R*. *massiliae* (pRma and pRmaB) strains (Fig 4 and e.g., Figure BA in S2 File). In contrast, the plasmids from either *R*. *raoultii* (pRra1/2/3), *Rickettsia* endosymbiont of *I*. *scapularis* (pReis1/3, except pReis1/2) or *R*. *amblyomii* (pRam18/23/32) strains were resolved into distinct phylogenetic groups with variable sequence conservation, although they lived in the same bacterial strain as well as the same host-species (Fig 4 and Figures BE to BG in S2 File).

The current RPs conserved highly variable numbers of genes acquired by VGT across the branches of the tree, ranging from 4 genes (6% out of the 65 pRICO genes) in the small pRaf plasmid to 31 (47% out of 65) in the large pReis1 plasmid (Fig 4). Therefore, the loss of ancestral genes in RPs ranged from 53% in pReis1 to 94% in pRaf. The latter was the compacted RP in which reduction was the greatest driving force.

#### Horizontal evolution

The plasmidome exhibited 23 (8%) pRIGs resulting from evident horizontal gene exchanges (HGT1, plasmid-chromosome and/or plasmid-plasmid) within a given *Rickettsia* species, and/or between distinct *Rickettsia* species (Fig 5, Figures A37 and A54 to A74 in S3 File). RPs contained variable numbers of pRIGs resulting from HGT1 across the tree ranging from 0 in the small pRaf plasmid to 7 in the large pReis2 plasmid (Fig 4). Unexpectedly, 4 pRIGs (1.4%, originally present in pRICO) of pRfe, pdRfe, pRfeI1, pRam32 and pRau exhibited close phylogenetic relationships with a gene cluster in *R*. *prowazekii* chromosomes (Rpr22_687 to Rpr22_698 and RprME_862 to RprME_879, Figures A1, A5, A57 and A100 in S3 File). Similarly, 6 pRIGs (out of the 34 pRIGs acquired by HGT2) in pRpe showed close phylogenetic relationships with a gene cluster in the *R*. *raoultii* chromosome (Rra_909 to Rra_916, Figure C in S2 File, Figures A87 to A92 in S3 File). These data suggest that both *Rickettsia* species may have had one (for *R*. *prowazekii*) or an additional (for *R*. *raoultii*) plasmid that was disrupted in the course of the reductive genomic evolution in the genus *Rickettsia*, with subsequent HGT1 integration of plasmidic genes in their chromosomes. Alternatively, HGT1 events may have occurred between the pRfe, pRam or pRau plasmids and the *R*. *prowazekii* chromosome, and between pRpe and the *R*. *raoultii* chromosome, respectively.

Thirty-four (12%) pRIGs exhibited evident horizontal gene transfers (HGT2, plasmid-genome) with known non-*Rickettsia/Orientia* species, mostly belonging to α/γ-proteobacteria lineages (Fig 5, Figure D in S2 File, Figures A75 to A111 in S3 File). Moreover, 55 (20%) pRIGs displayed putative horizontal gene exchanges (HGT2) of unknown origin, but matching homologs of non-*Rickettsia/Orientia* species, also mostly belonging to α/γ-proteobacteria lineages (Fig 5, Figure E in S2 File, Figures A39 to A44, A46, A47, A100 and A112 to A136 in S3 File). RPs harbored highly variable numbers of genes exchanged by HGT2 events ranging from 0 in the small pRam23 plasmid to 24 in the large pRra2 plasmid (Fig 4).

Finally, five pRIGs (1.8%, originally present in 65 pRIGs inherited by VGT) and one pRIG (0,4%, originally present in the 34 pRIGs exchanged by HGT2) displayed evidence of additional gene transfers (HGT3, plasmid-genome) between *Rickettsia* and distant lineages including *Cardinium* endosymbiont of *Encarsia pergandiella*, *Candidatus* Amoebophilus asiaticus (Bacteroidetes) as well as bacterial endosymbionts of either *Trichoplax adhaerens* or *Hydra magnipapillata* (Bacteria) (Fig 5, Figure D in S2 File, Figures A10, A11, A55, A57, A58 and A101 in S3 File) [42–44].

#### ORFans

The plasmidome contained a set of 64 pRIGs (23%, one pRIG in 1 to 8 plasmid(s)) classified as ORFans, including 40 that are specific for one given plasmid (Fig 5). RPs harbored highly variable numbers of ORFans ranging from 2 in the small pRaf plasmid to 18 in the large pRra2 plasmid (Fig 4). These ORFans may have been horizontally acquired from microbes or eukaryotes not yet identified or that have disappeared, or have the *de novo* origin (gene genesis) either from non-coding regions, through an overprinting phenomenon of genes and/or underwent gene degradation processes [45–47].

#### Duplications

Recent duplication events drove 34 pRIGs (12%, one inparalog can be present 1 to 5 times in 1 to 4 plasmid(s)) after the divergence of pRICO (Fig 5). Of these, 21, 9 and 4 pRIGs were duplicated from pRIGs acquired by VGT and HGT2 (Figures A1, A4, A6 to A8, A10, A38, A50, A55 to A58, A60, A61, A81 to A86, A97, A100 and A129 in S3 File) as well as from ORFan pRIGs, respectively. The plasmids contained highly variable numbers of inparalogs across the tree ranging from 0 in the small plasmid pRaf to 20 in the large plasmid pReis2 (Fig 4). Overall, the gene proliferation by recent duplications in plasmids was found to be similar to duplication phenomenon in the corresponding chromosomes (see phylogenetic trees).

In summary, the current RPs were vertically transmitted from their last common ancestor pRICO. They evolved dynamically under an obligate intracellular lifestyle in two main phases: i) reductive evolution including pseudogenisation as well as gene degradation and loss, and ii) gene gain and innovation via HGT and proliferation-duplication as well as gene genesis. This mode of evolution is described in parasitic and symbiotic organisms as a recurrent biphasic model dominated by longer phases of genome reduction and simplification, punctuated by shorter phases of episodic complexification [39]. Although the expansion via HGT and gene duplications occurred in RPs, the contraction via gene degradation and loss severely contributed to the reduction in gene content and plasmid size, and thus probably led to plasmid degradation and/or loss in several rickettsial species. This evolution of RPs seems to be in accordance with a recent study which revealed that the prevailing mode of evolution in bacteria is genome reduction, which is partially compensated by the gain of new gene families via HGT [48]. The HGT process conferred on RPs a potential to exchange and disseminate their gene pools with their co-residing chromosomes and genomes of closely-related and distant phylogenetic lineages, while duplication events enabled an internal genetic amplification. This indicates that RPs were able to overcome the genetic barriers to HGT between distant phylogenetic lineages such as α-, γ-proteobacteria and/or Bacteroidetes. Similar horizontal transfer events between phylogenetically-distant clades were previously described (*e*.*g*., between plasmids from α-proteobacteria and Deinococci), underlying the key role of plasmids in genetic exchange throughout the microbial world [25]. Furthermore, all the identified evolutionary processes variably occurred across RPs suggesting that rickettsiaes may have been adapted to distinct biotic and/or abiotic factors for the invasion, survival and/or defense in eukaryotic host cells. Snel et al. [49] suggested that gene loss is under negative selection, while the process that adds genes is under positive selection.

### Impact of evolution on functional diversity of RPs

**Fig 6:**
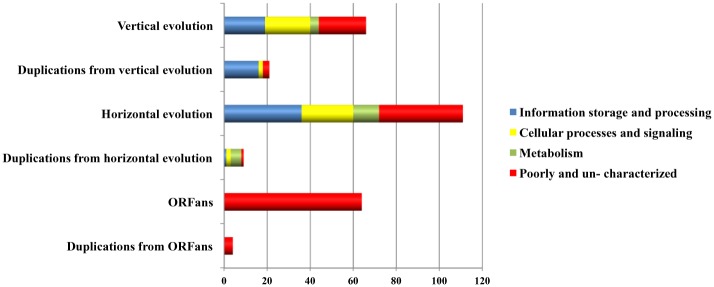
COG functional categories of *Rickettsia* plasmidome originated from vertical and horizontal evolutions as well as duplications.

We examined the impact of the identified evolutionary forces (*i*. *e*., VGT, HGT, gene genesis, duplications and genome reduction) on the functional diversity of the RPs by a comparative analysis of the COG functional categories of the plasmidome (Fig 6). Overall, VGT had a weaker influence than HGT on pRIGs involved in information storage and processing (6% vs 14%, resp.), metabolism (1.4% vs 4%, resp.) and poorly- or un-characterized genes (8% vs 14%, resp.). In contrast, both forces had a similar impact on pRIGs related to cellular processes and signaling (7.6% vs 8.7%, resp.).

### Information storage and processing categories

In information storage and processing categories, both VGT and HGT forces drove high and distinct proportions of pRIGs associated with replication, recombination and repair (5 vs 13%, resp.), but less and similar proportions on those involved in transcription (0.7 vs 0.3%, resp.) and translation (0 vs 0.3%, resp.) (see details in Table B in S1 File). Moreover, duplication forces acted on pRIGs resulting more from VGT than HGT events, and involved in replication, recombination and repair (6 vs 0.3%, resp.) (see details in Table B in S1 File). The abundance of HGT pRIGs from rickettsial and non-rickettsial chromosomes and duplications from VGT pRIGs in the replication, recombination and repair category were mainly due to mobile transposable elements (*i*.*e*., transposases and integrases, 9 out of 13% and 5.1 out of 6%, resp.). These data from rickettsial plasmids corroborate a recent large-scale evolutionary study that showed that microbial plasmids share mainly genes encoding proteins involved in DNA transposition and recombination, some functions strongly linked to HGT process [25]. The single pRIG associated to translation was also exchanged with rickettsial chromosomes. Thus, VGT, HGT and duplication forces have driven pRIGs (*e*.*g*., *dna*A-like, transposases, integrases, resolvases and relaxases) that may contribute to maintenance, rearrangements and plasticity of plasmids, making them self-replicative and simultaneously dynamic to gene exchange with various evolutionary lineages and consequent diversification of rickettsial biological activities and phenotypic traits.

### Cellular processes and signaling categories

In cellular processes and signaling categories, both VGT and HGT forces acted in nearly equal proportions on pRIGs related to intracellular trafficking and secretion (*tra* operons) (4.3 vs 4%, resp.) (see details in Table B in S1 File). Based on the classification of plasmids by their mobility [38], our data predict that three (15%) large plasmids (pRra2/pReis1/pReis3) are self-transmissible or conjugative (*i*.*e*., having MOB or relaxases and TRA modules), whereas 8 (40%) small or large plasmids (pRam32/pRra3/pReis2/pRau/pdRfe/pRfe/pRfeI1/pRhe) are mobilisable (i.e., having MOB and/or degraded or no TRA modules). Some of these mobilisable replicons (e.g., pRra3) could be transmissible if using conjugative machineries of co-residing self-transmissible replicons (e.g., pRra2) [38, 50, 51]. The 9 (45%) remaining small plasmids (e.g., pRaf/pRam12/pRam23/pRrh/pRpe/pRmo/pRra1/pRma/pRmaB), lacked MOB and TRA modules and may not be transmissible. The three mobility types in RPs are common in various bacterial phyla including α-proteobacteria [38]. Overall, the presence of conjugative system in RPs may have strong implications in their evolutionary dynamics and history as well as in the acquisition and spread of ecologically important traits (novel and/or ancestral among various bacteria) that may be associated to rearrangement, adaptation and/or pathogenesis of rickettsiae. This is consistent with the analysis of gene sharing networks among 329 proteobacteria that suggested that gene transfer in this phylum is frequently mediated by conjugation [52].

Moreover, both VGT and HGT forces drove in a similar way pRIGs related to posttranslational modification and chaperones (0.7 vs 0.3%, resp.) and defense mechanisms (0.3 vs 0.7%, resp.), but not pRIGs associated to transduction mechanisms (e.g., 2 vs 0.3%, resp.) (see details in Table B in S1 File). Among these processes we can cite pRIGs associated to adaptation (e.g., *hsp*), defense (type I restriction-modification system methyltransferase subunit) and stress (e.g., *spo*T) that may help the survival of host bacteria in an obligate intracellular life style. However, HGT, but not VGT forces, drove 3.3% of pRIGs related to cell wall/membrane/envelope biogenesis and cycle control, including genes associated to plasmid segregation (*e*.*g*., *par*A/B) and regulator of cell division and wall (*e*.*g*., mobile mystery protein B, putative lytic transglycosylase, glycosyltransferase, group 1 family protein). In addition, duplication forces drove equal proportions of pRIGs resulting from both VGT and HGT events, and associated with intracellular trafficking and secretion, posttranslational modification and chaperones and cycle control (0.3 vs 0.3%, resp.).

### Metabolism categories

In metabolism categories, both VGT and HGT forces drove equal proportions of pRIGs involved in inorganic ion transport and metabolism (0.3 vs 0.3%, resp.) (see details in Table B in S1 File). However, only VGT forces acted on 1.1% of pRIGs related to nucleotide/carbohydrate transport and metabolism (thymidylate kinase, major facilitator superfamily MFS-type transporter). In contrast, only HGT forces drove 4.4% of pRIGs associated with amino acid/lipid/coenzyme and secondary metabolites biosynthesis, transport and metabolism (*e*.*g*., L-allo-threonine aldolases, 3-oxoacyl-(acyl-carrier-protein) synthase III, *bio*A, B, C, D and F). Furthermore, duplication forces acted on 1.8% of pRIGs including *bio*A, B, C, D and F. Overall, VGT and duplication forces drove pRIGs common to RPs and *Rickettsia/Orientia* chromosomes which may increase the biological activities of some rickettsiae, whereas HGT forces enabled some RPs to acquire novel metabolic functions absent in *Rickettsia/Orientia* chromosomes that may complete host-metabolic gaps and confer host-adaptive phenotypes to obligate intracellular lifestyle.

### Poorly and uncharacterized categories

In poorly and uncharacterized categories, VGT, HGT and *de novo* gene genesis drove distinct proportions of pRIGs (7.6, 13.8 and 23.3%, resp.) which have known or unknown functions and/or poorly or uncharacterized COG assignments and that remain to be elucidated for the biology of *Rickettsia* species (see details in Table B in S1 File). Moreover, duplication forces acted on similar proportions of pRIGs resulting from VGT, HGT and *de novo* genes genesis (1.1, 0.3 and 1.4%, resp.). Among the poorly and uncharacterized categories, VGT forces drove some pRIGs (2.5%) related to adhesion and/or infection such as those encoding the cell surface antigen proteins Sca and patatin-like phospholipase Pat [10, 53], whereas HGT forces acted on some pRIGs (5.8%) coding ankyrin repeat-containing proteins Ank, tetratricopeptide repeat-containing proteins Tpr known to play a role in protein-protein interactions in eukaryotes, and suspected to be associated to adaptation to hosts and/or to virulence [14].

In several human bacterial pathogens, such as *Salmonella* spp., *Escherichia coli* and *Borrelia* spp., virulence plasmids have been identified [37, 41, 54]. However, among the most pathogenic rickettsiae, *R*. *prowazekii* and *R*. *conorii* do not harbor any plasmid whereas *R*. *australis* contains one plasmid. These data suggest that the role of RPs in rickettsial virulence, remains uncertain and requires further investigations. In addition, the functional diversity of RPs observed in the present study suggests that some of them may be used as transformation vectors for rickettsiae, as was recently described using a shuttle vector system derived from *R*. *amblyomii* plasmids that enabled stable transformations of diverse rickettsiae [55, 56]. Thus, the genetic manipulation of rickettsiae using RPs may provide new insights in the pathogenesis of these obligate intracellular bacteria as well as the dissemination of their genotypic and phenotypic traits.

## Conclusions

This study offers an overview of the evolution and functional diversity of rickettsial plasmids. The current RPs have been shaped by multiple evolutionary forces in a bottleneck ecological niche. Initially, they were vertically inherited and then may have evolved under a biphasic model including a strong reductive evolution as well as innovations by horizontal gene transfer and gene duplication and genesis. This study demonstrates for the first time that plasmids of obligate intracellular bacteria can evolve under both vertical and reductive forces similar to those shaping their co-residing chromosomes. The evolutionary processes that have shaped RPs suggest that they might be influenced by molecular interactions between rickettsiae and eukaroytic host cells including biological needs for adaptation and survival, as suggested for *Buchnera aphidicola* in which metabolic requirements could determine when genome reduction occurs [39]. RPs may play diverse functional roles that are either novel or similar to those of rickettsial chromosomes via various evolutionary mechanisms. Overall, this study provides insights into rickettsial plasmids and their biology and evolution.

## Supporting Information

S1 File (PDF)

S2 File (PDF)

S3 File (PDF)
