# Supplementary material for: Origin and Evolution of Rickettsial Plasmids
Source: PLoS One. 2016 Feb 11;11(2):e0147492. doi: 10.1371/journal.pone.0147492 (PMC4750851; doi:10.1371/journal.pone.0147492)
Supplement: S1 File — (PDF) [file pone.0147492.s001.pdf]

Table A. General features of circular sequenced plasmids of the genus *Rickettsia*.

Species & strain	Name	Size	Genes	Coding (%)	GC (%)
R. africae ESF-5	pRaf	12,377	15	88	34
R. massiliae MTU5	pRma	15,286	16	78	32
R. massiliae AZT80	pRmaB	15,000	16	81	32
R. peacokii Rustic	pRpe	26,406	29	87	35
R. monacensis IrR	pRmo	23,486	29	82	32
R. helvetica C9P9	pRhe	47,188	50	90	33
R. felis URRWXCal2	pRfe	62,829	68	86	34
	pdRfe	39,263	44	86	33
R. felis I1	pRfeI1	62,882	70	86	34
C. R. amblyomii AaR/SC	pRam18	18,344	17	75	32
	pRam23	22,852	21	75	33
	pRam32	31,972	36	84	34
R. endosymbiont REIS	pReis1	55,147	52	87	34
	pReis2	66,811	67	83	32
	pReis3	49,883	42	92	35
R. raoultii DNS14	pRra1	20,840	22	81	32
	pRra2	83,219	85	92	34
	pRra3	34,583	31	90	34
R. australis	pRau	26,610	20	82	34
R. rhipicephalii	pRrh	15,099	17	80	32

***** pReis4 plasmid has a size of 33,951 bp as an incomplete linear contig available in GenBank database.

Table B. Comparative COG functional categories of vertical and horizontal gene transfers (VGT and HGT, resp.) as well as duplicated genes between rickettsial plasmids and *Rickettsia/Orientia* as well as non-*Rickettsia/Orientia* genomes.

**Genes from VGT* and duplicated VGT****	**Genes from HGT1†, HGT2* and duplicated HGT2****
**Information storage and processing [L, K and J]**	
**Replication, recombination and repair**	**Replication, recombination and repair**
2 *dna*A-like replication initiator protein*	15 transposases and/or integrases†
6 transposases and/or integrases*	1 RNA-directed DNA polymerase†
2 helicases *rec*D/*tra*A*	1 Deoxyribodipyrimidine photo-lyase†
2 conjugative transfer relaxase *tra*A_Ti chimeric or not with topoisomease*	10 transposases and/or integrases*
1 DNA polymerase III, epsilon subunit *	5 resolvases*
1 DNA adenine methylase domain protein*	2 RNA-directed DNA polymerase *
14 transposases and/or integrases**	1 DNA-directed DNA polymerase*
1 helicases *rec*D/*tra*A**	1 Cytosine-specific methyltransferase*
	1 resolvase**
**Transcription**	**Transcription**
2 Helix-turn-helix DNA-binding domain*	1 Putative DNA-binding protein*
1 Helix-turn-helix DNA-binding domain**	1 ParB-like nuclease domain-containing protein*
**Translation**	**Translation**
	1 HemK†
**Cellular processes and signaling [O, U, M, D, V and T]**	
**Intracellular trafficking and secretion**	**Intracellular trafficking and secretion**
12 conjugative transfer/coupling proteins Tra, Trb and Trw*	11 conjugative transfer/coupling proteins Tra, Trb and Trw*
1 conjugative transfer protein containing TraD domain**	
**Postranslational modification, protein turnover, chaperones**	**Postranslational modification, protein turnover, chaperones**
2 small heat shock proteins Hsps*	1 lon ATP-dependent protease with TPR domain*
1 small heat shock proteins Hsps**	1 lon ATP-dependent protease with TPR domain**
**Cell wall/membrane and biosynthesis**	**Cell wall/membrane and biosynthesis**
	1 Putative lytic transglycosylase (LT) domain protein*

	1 Conserved protein of unknown function*<br>1 NAD-dependent epimerase/dehydratase family protein*<br>1 Glycosyltransferase, group 1 family protein*
**Cell cycle control**	**Cell cycle control**<br>3 Plasmid partitioning ParA family protein*<br>1 Plasmid partitioning ParA-like family protein*<br>1 Mobile mystery protein B*<br>1 Plasmid partitioning ParA-like family protein**
**Defense mechanisms**<br>1 Type I restriction-modification system methyltransferase subunit*	**Defense mechanisms**<br>1 Type I restriction-modification system methyltransferase subunit*<br>1 ABC multidrug transporter, permease/ATP-binding protein*
**Transductions mechanisms**<br>1 Guanosine polyphosphate pyrophosphohydrolase/synthetase SpoT22*<br>2 ProP/Q activator of osmoprotectant transporter*<br>1 Tryptophan-rich sensory protein, TspO_MBR superfamily*<br>1 Transcriptional regulator, luxR family*<br>1 Response regulation protein PleD*	**Transductions mechanisms**<br>1 Guanosine polyphosphate pyrophosphohydrolase/ synthetase SpoT23†
**Metabolism [E, F, G, I, P, H and Q]**	
**Nucleotide transport and metabolism**<br>1 Thymidylate kinase*	**Nucleotide transport and metabolism**
**Carbohydrate transport and metabolism**<br>2 Major facilitator superfamily MFS-type transporter*	**Carbohydrate transport and metabolism**
**Amino acid transport and metabolism**	**Amino acid transport and metabolism**<br>2 L-allo-threonine aldolase*
**Lipid transport and metabolism**	**Lipid transport and metabolism**<br>1 Short chain dehydrogenase/reductase family protein*<br>1 3-oxoacyl-(acyl-carrier-protein) synthase III*<br>1 Acyl carrier protein*

**Coenzyme, transport and metabolism**	**Coenzyme, transport and metabolism**<br>1 Transaminase BioA*<br>1 Dethiobiotin synthase BioD*<br>1 Synthetase and related enzymes BioF*<br>1 Biotin synthase BioB*<br>1 Transaminase BioA**<br>1 Dethiobiotin synthase BioD**<br>1 Synthetase and related enzymes BioF**<br>1 Biotin synthase BioB**
**Secondary metabolites biosynthesis, transport and catabolism**	**Secondary metabolites biosynthesis, transport and catabolism**<br>1 Biotin biosynthesis protein BioC, S-adenosylmethionine-dependent methyltransferases family protein*<br>1 Phytanoyl-CoA dioxygenase family protein*<br>1 Biotin biosynthesis protein BioC, S-adenosylmethionine-dependent methyltransferases family protein**
**Inorganic ion transport and metabolism**<br>1 SMR-type multi-drug efflux transporter*	**Inorganic ion transport and metabolism**<br>1 Rieske non-heme iron oxygenase (RO) family protein*
**Poorly- and uncharacterized**	
1 Cell surface antigen Sca12*<br>1 Cell surface antigen Sca4*<br>1 Patatin-like phospholipase*<br>2 WGR domain-containing protein*<br>1 Peptidase family M50*<br>1 Integral membrane protein*<br>15 Conserved protein of unknown function*	6 Conserved protein of unknown function†<br><br>8 Tetraticopeptide repeat-containing protein*<br>5 Ankyrin repeat-containing protein*<br>1 alpha/beta hydrolase family protein*<br>1 Hyaluronidase*<br>17 Conserved protein of unknown function*
2 Cell surface antigen Sca12**<br>1 Conserved protein of unknown function**	1 alpha/beta hydrolase family protein**
