# Supplementary material for: Origin and Evolution of Rickettsial Plasmids
Source: PLoS One. 2016 Feb 11;11(2):e0147492. doi: 10.1371/journal.pone.0147492 (PMC4750851; doi:10.1371/journal.pone.0147492)

Figure A. Correlation between pooled plasmid sizes and chromosome sizes of *Rickettsia* species. The coefficient of determination is R^2 =0.76 and the coefficient of correlation of Pearson is R=0.91, *P-value* <0.05.

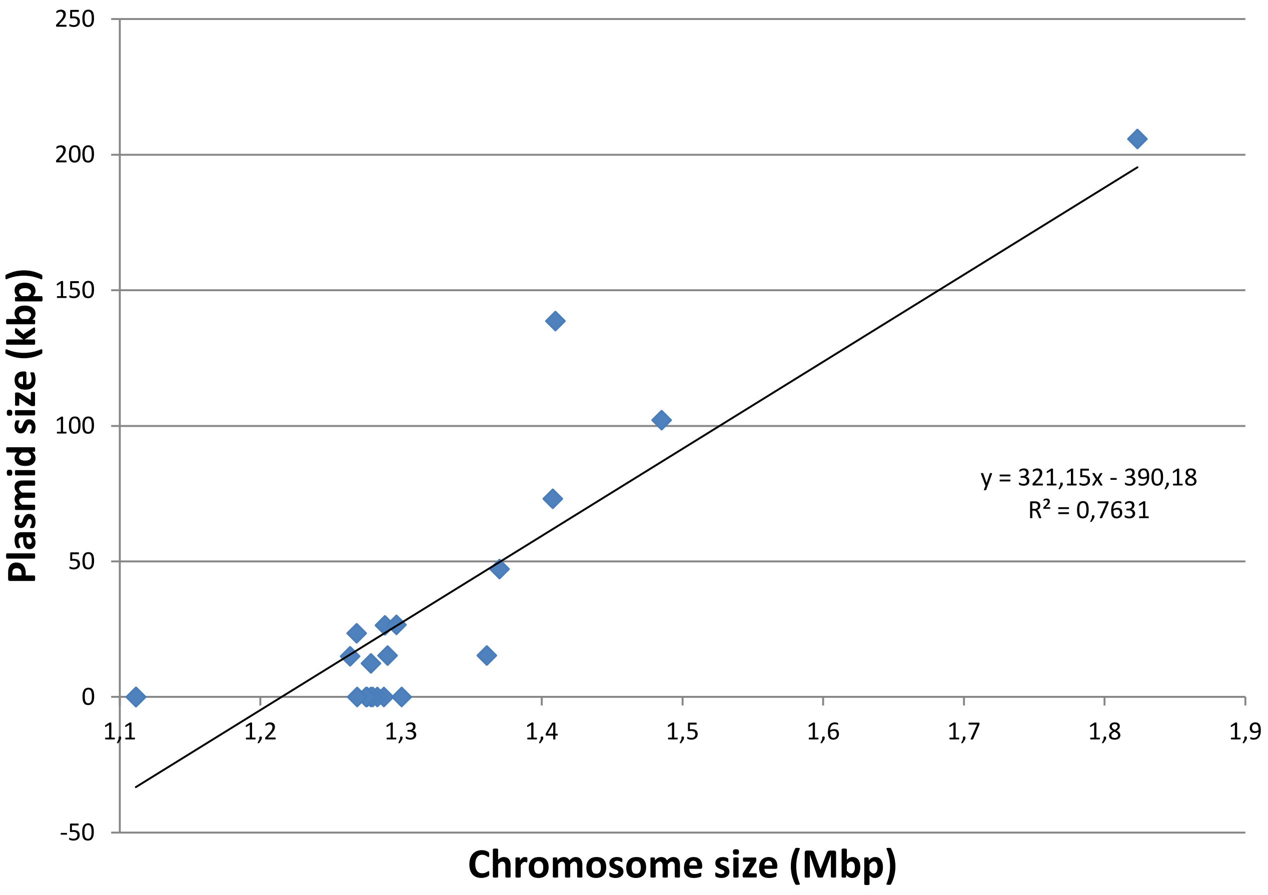

Figure B. Pairwise alignments of complete sequences of the four major groups I (A), II (B), III (C) and IV (D) of *Rickettsia* plasmids as well as of intra-cellular plasmid of *R. raoultii* (E), *R. endosymbionte of I. scapularis* (F) and *R. amblyomii* (G). The plasmid pRra2 was used as reference for all alignments. All sequences started with *dna*A-like gene and ended with *par*A and some unknown genes.

A

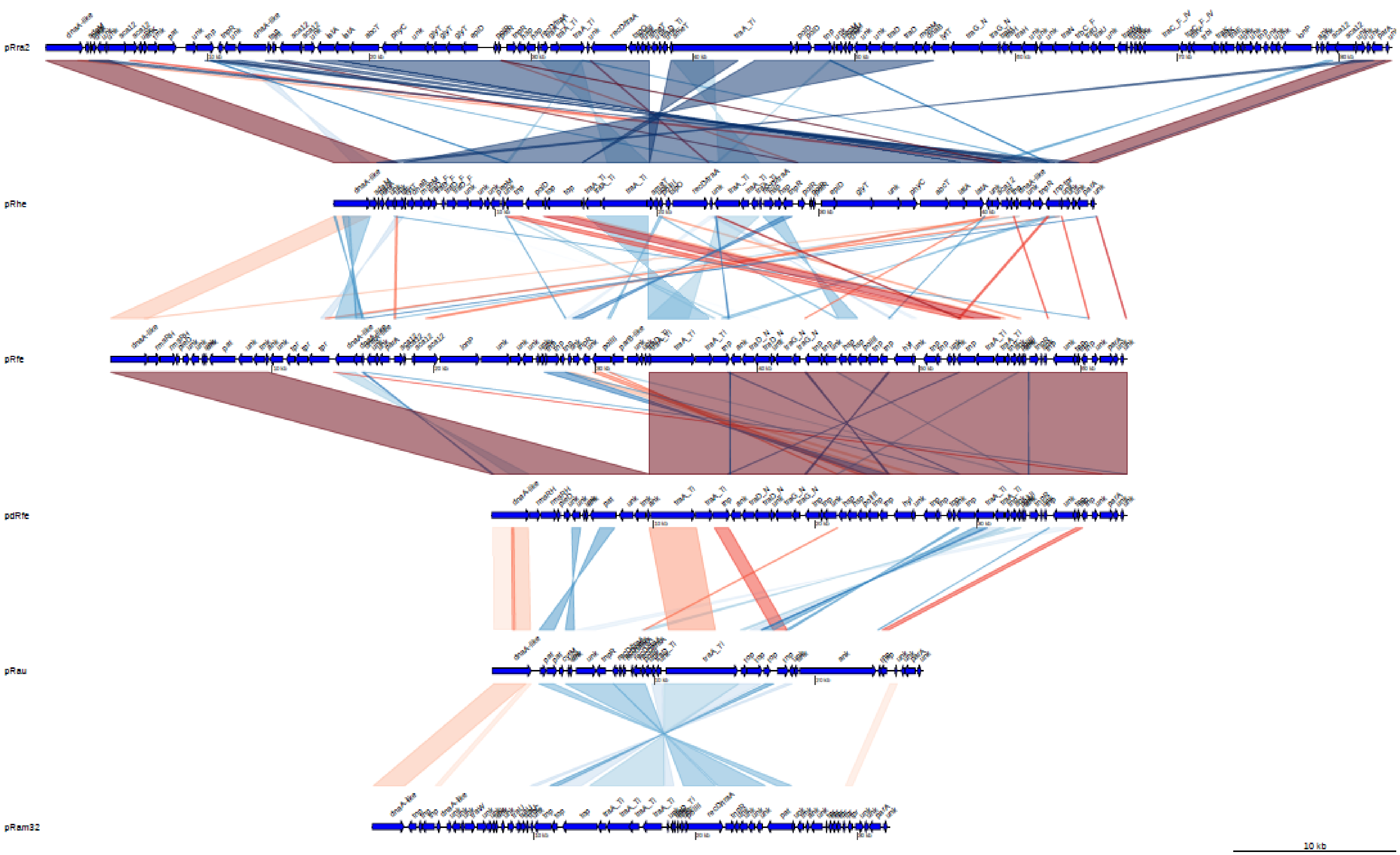

B

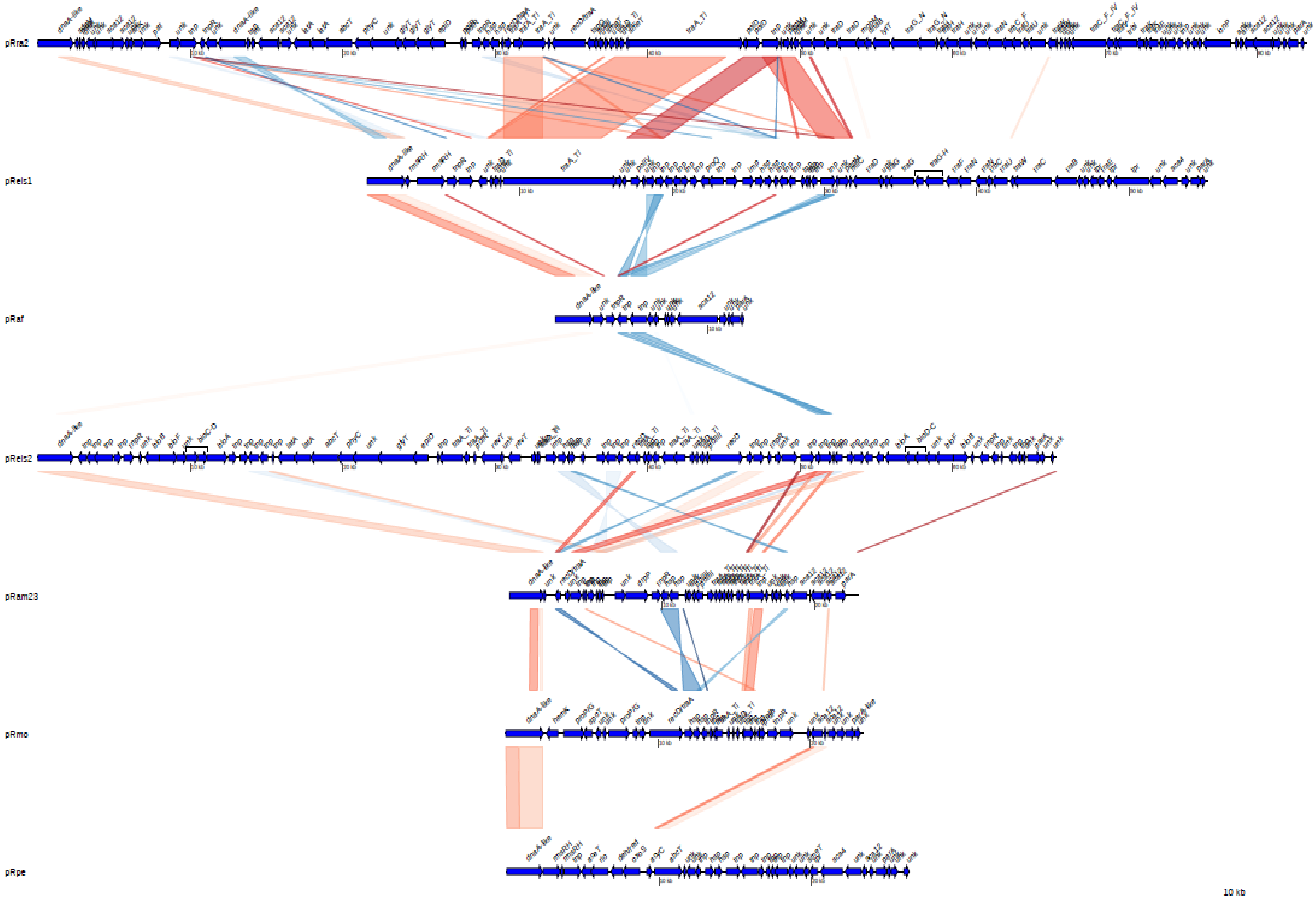

C

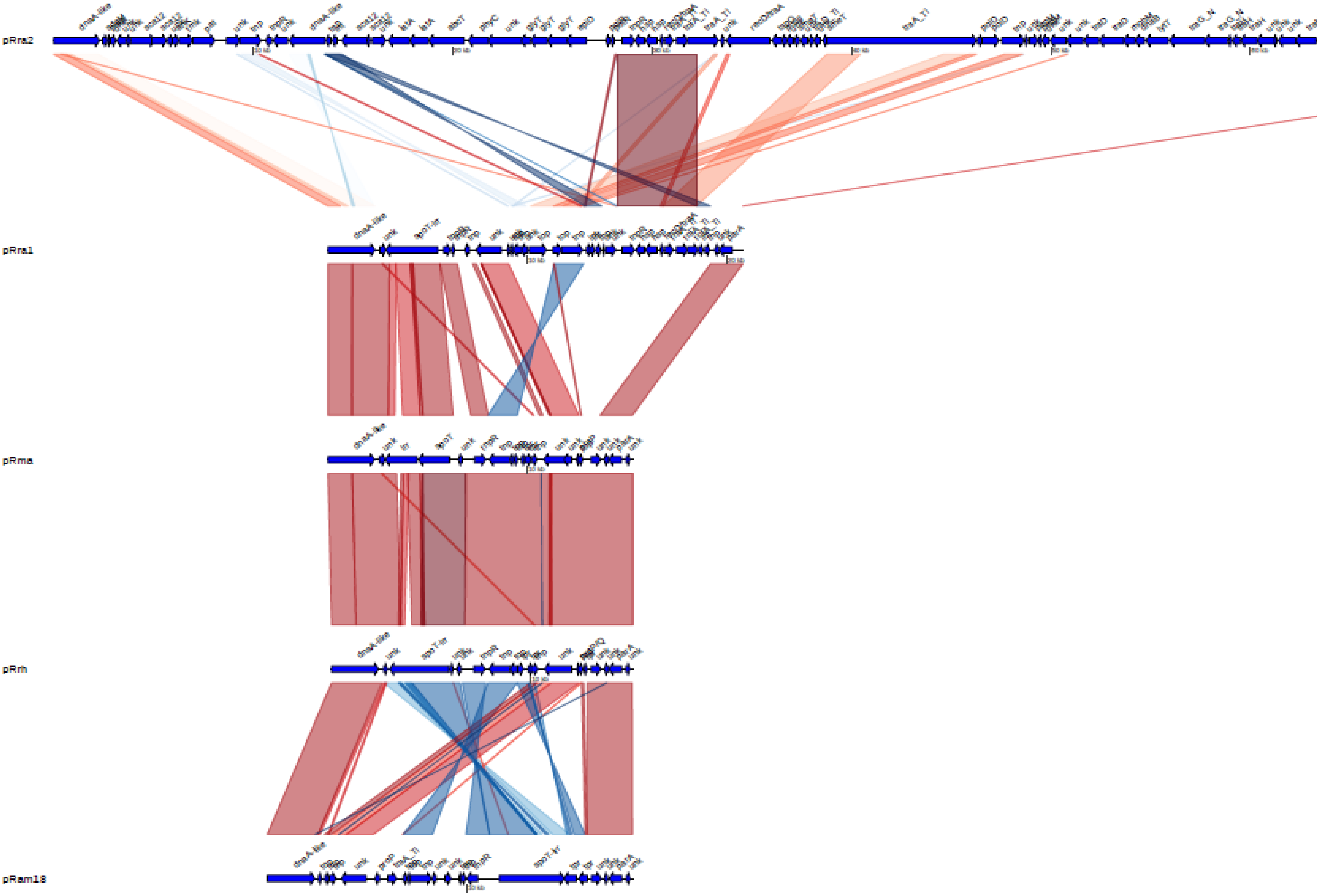

D

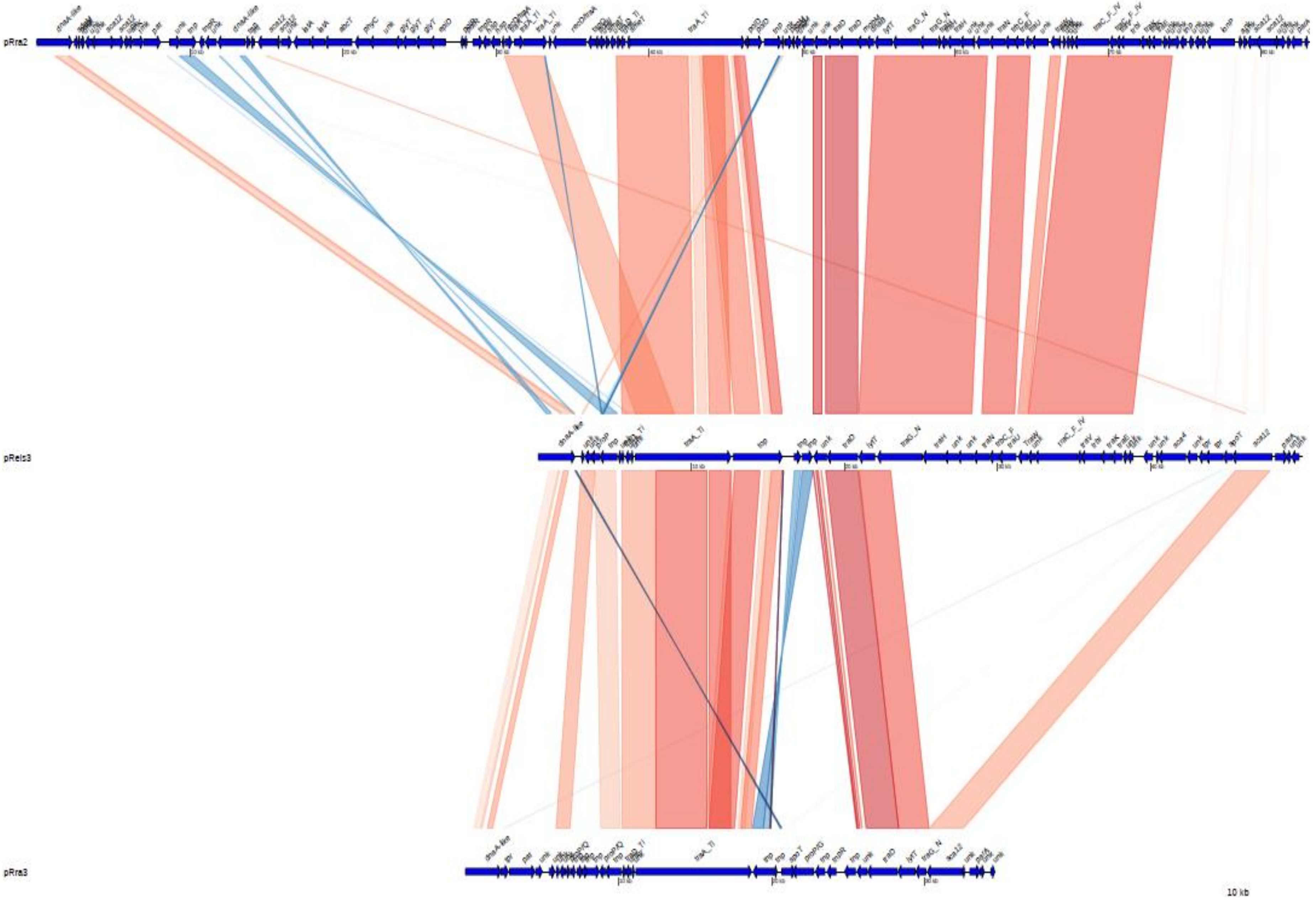

E

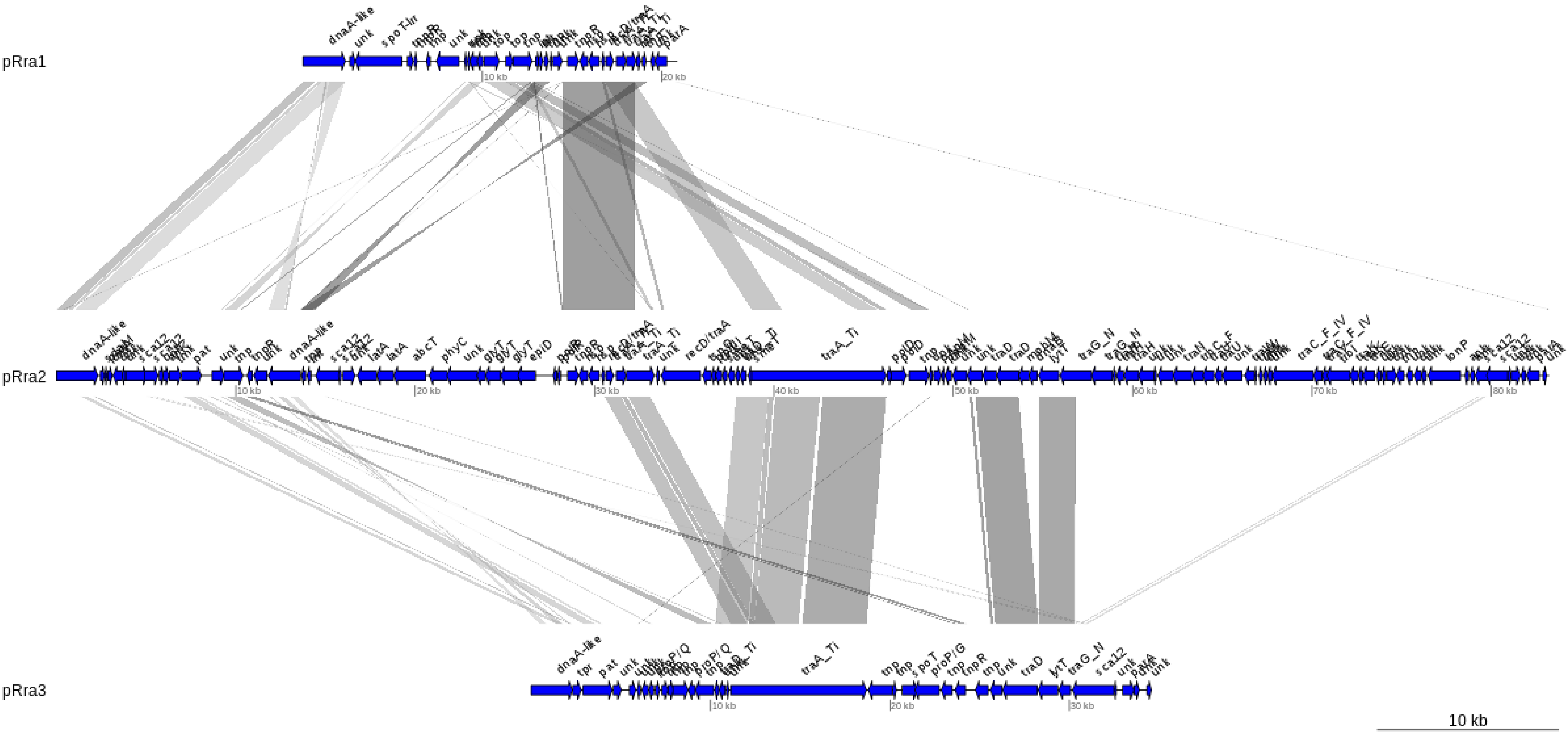

F

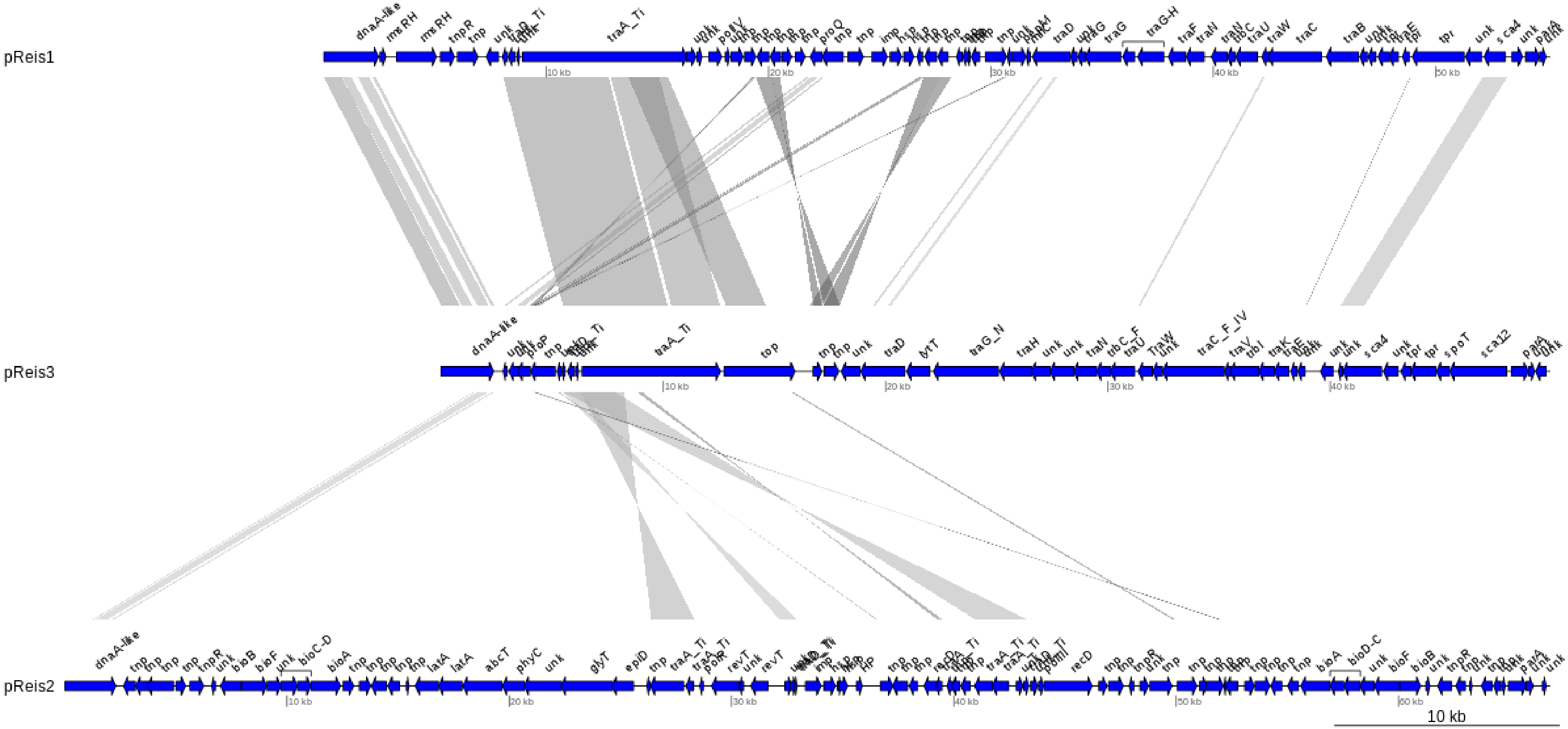

pReis1
pReis3
pReis2
dnaA-like
traA_Ti
traD
traC
traB
top
traG_N
bioA
bioF
bioB
latA
abcT
phyC
glyT
recD
parA
10 kb
20 kb
30 kb
40 kb
50 kb
60 kb

G

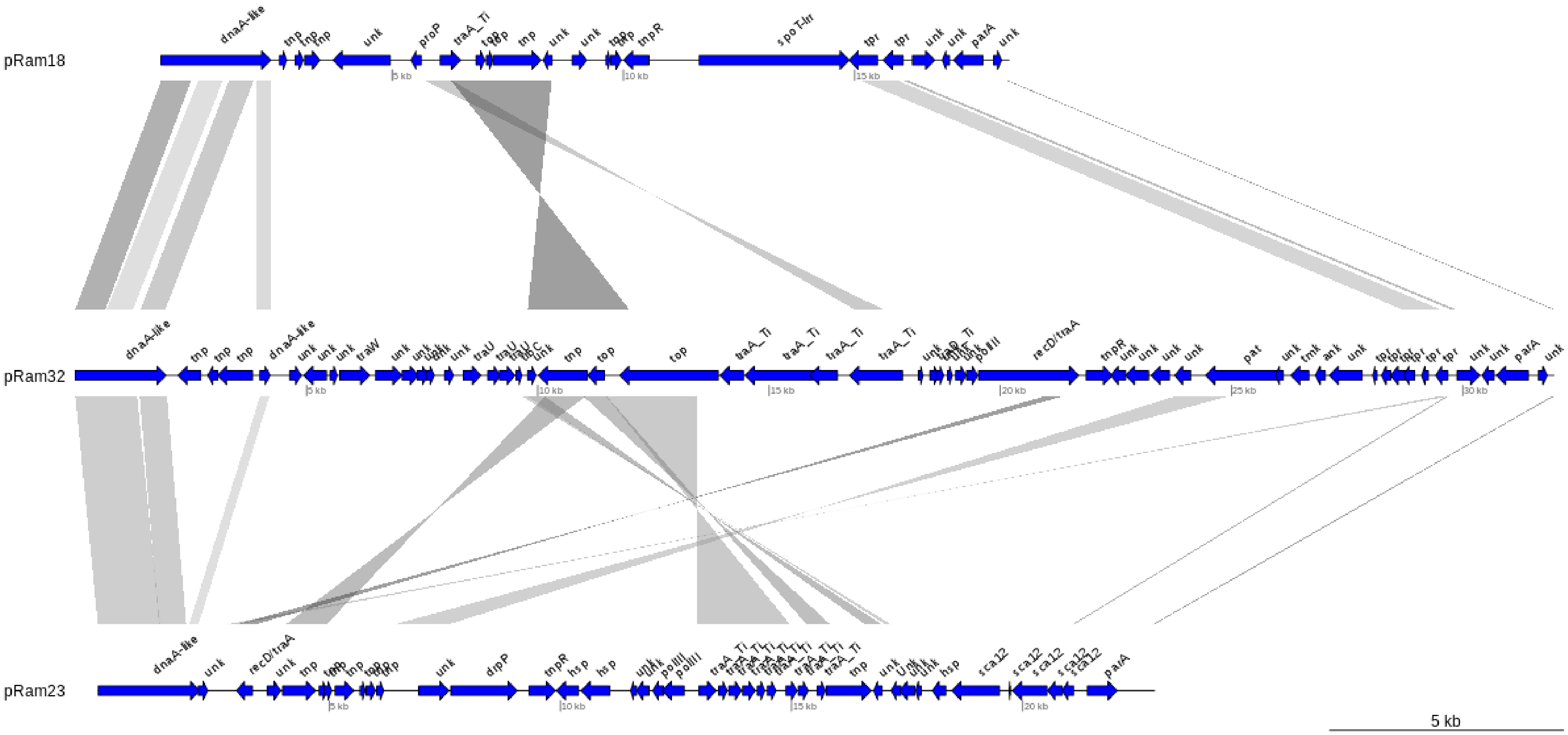

Figure C. Horizontal gene transfers occurred between *R. raoultii* chromosome, *R. peacokii* plasmid and *Pseudomonas aeruginosa* chromosome.

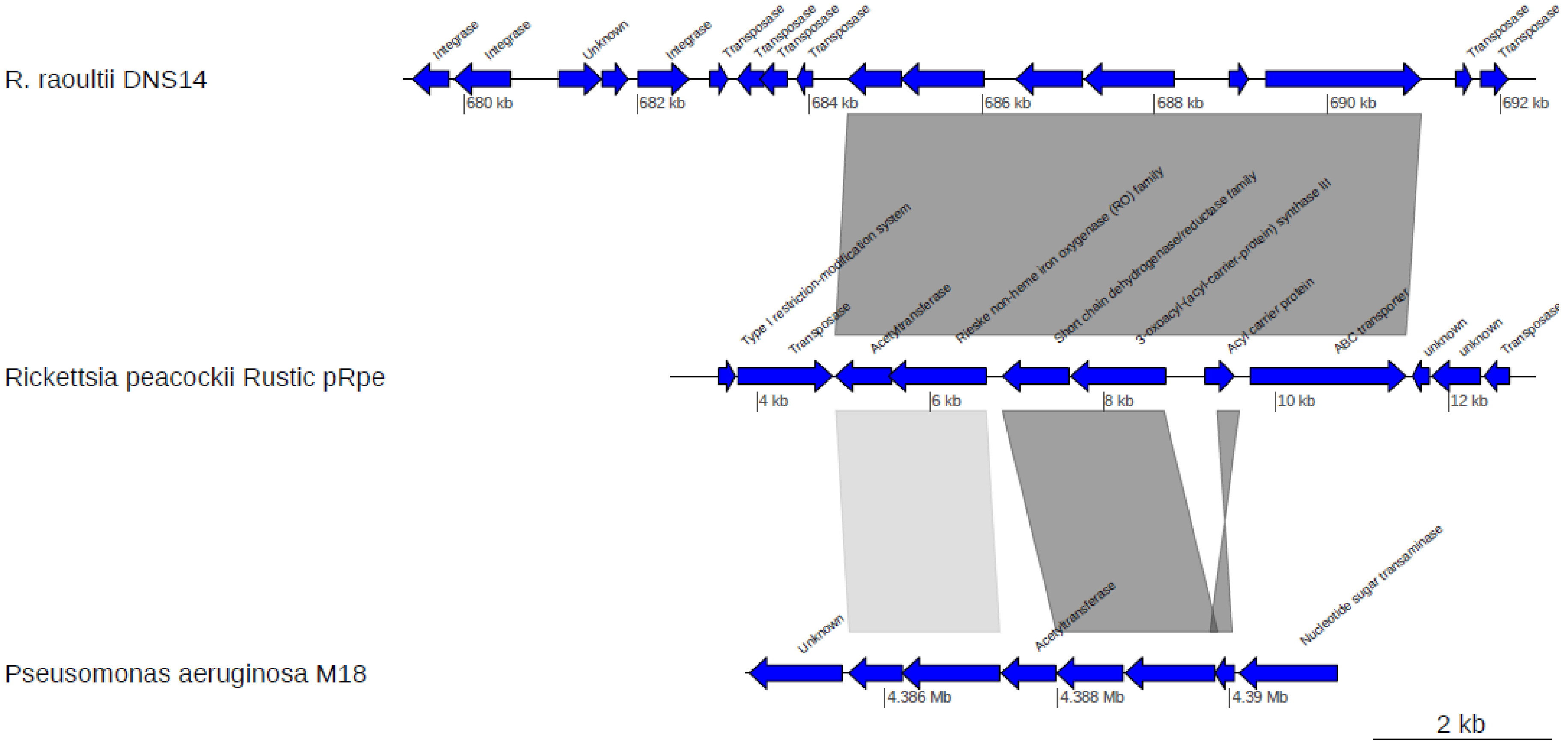

Figure D. Species details of evident HGT2 and 3 events occurred between rickettsial plasmids and non-*Rickettsia/Orientia* genomes, as inferred from best BLAST homology and phylogenetic relationships (50>=aa identity<=100 and BP>=60). Colors of species correspond to α-proteobacteria (blue), γ-proteobacteria (yellow), β-proteobacteria (cyan), δ-proteobacteria (beige), Bacteoidetes (red), Planctomycetes (green) and Other bacteria (white).

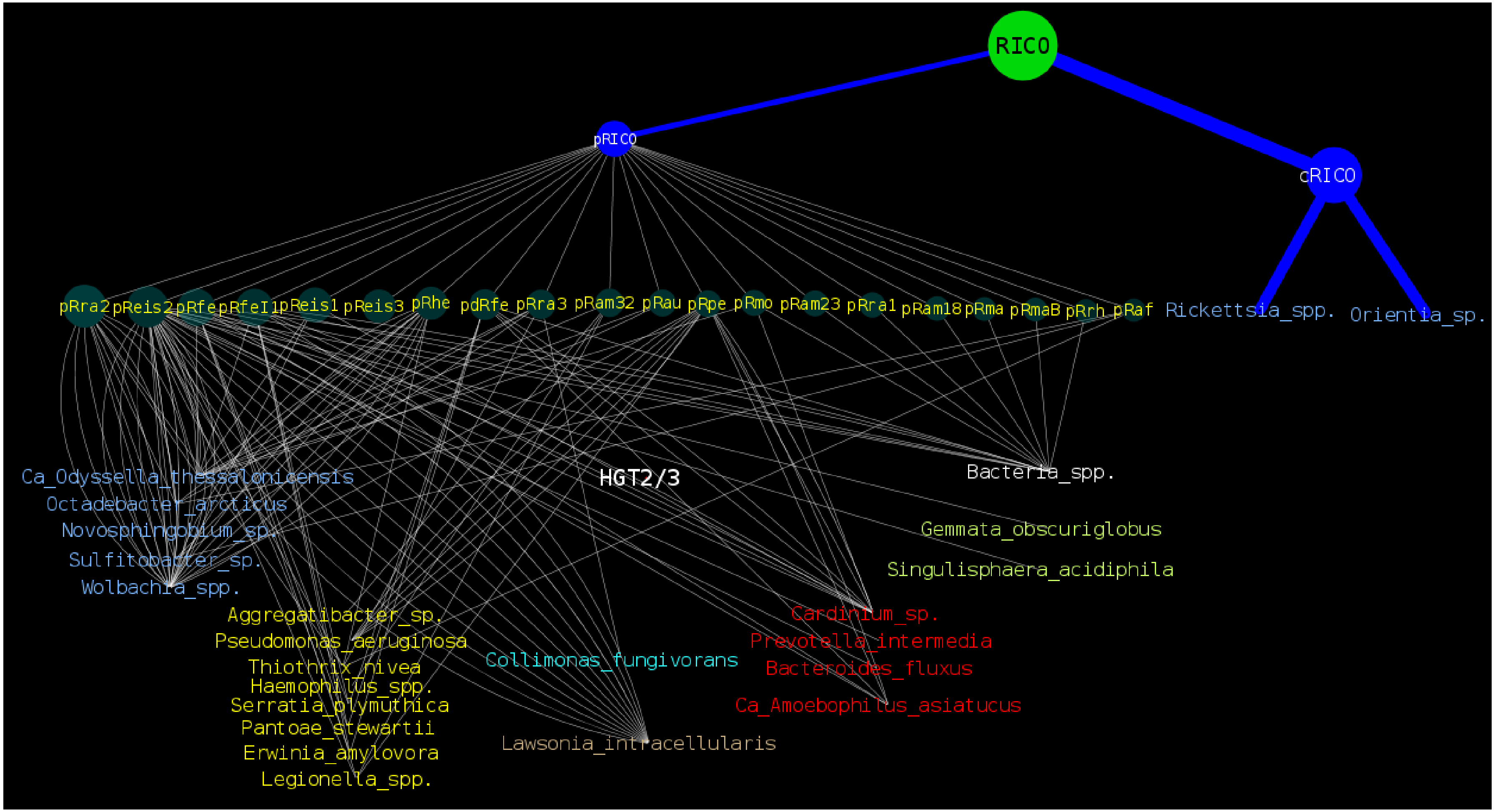

Figure E. Genus detail of putative HGT2 events occurred between rickettsial plasmids and unknown non-*Rickettsia/Orientia* genomes, as inferred from best BLAST homology (25>=aa identity<80). Colors of species correspond to α-proteobacteria (blue), γ-proteobacteria (yellow), β-proteobacteria (cyan), Bacteoidetes (red), Deferribacteres (yellow), Cyanobacteria (silver), Chlamydiae (white), Firmicutes (grey), Spirochaetes (white), Archaea (brown) and Eukaryota (turqoise).

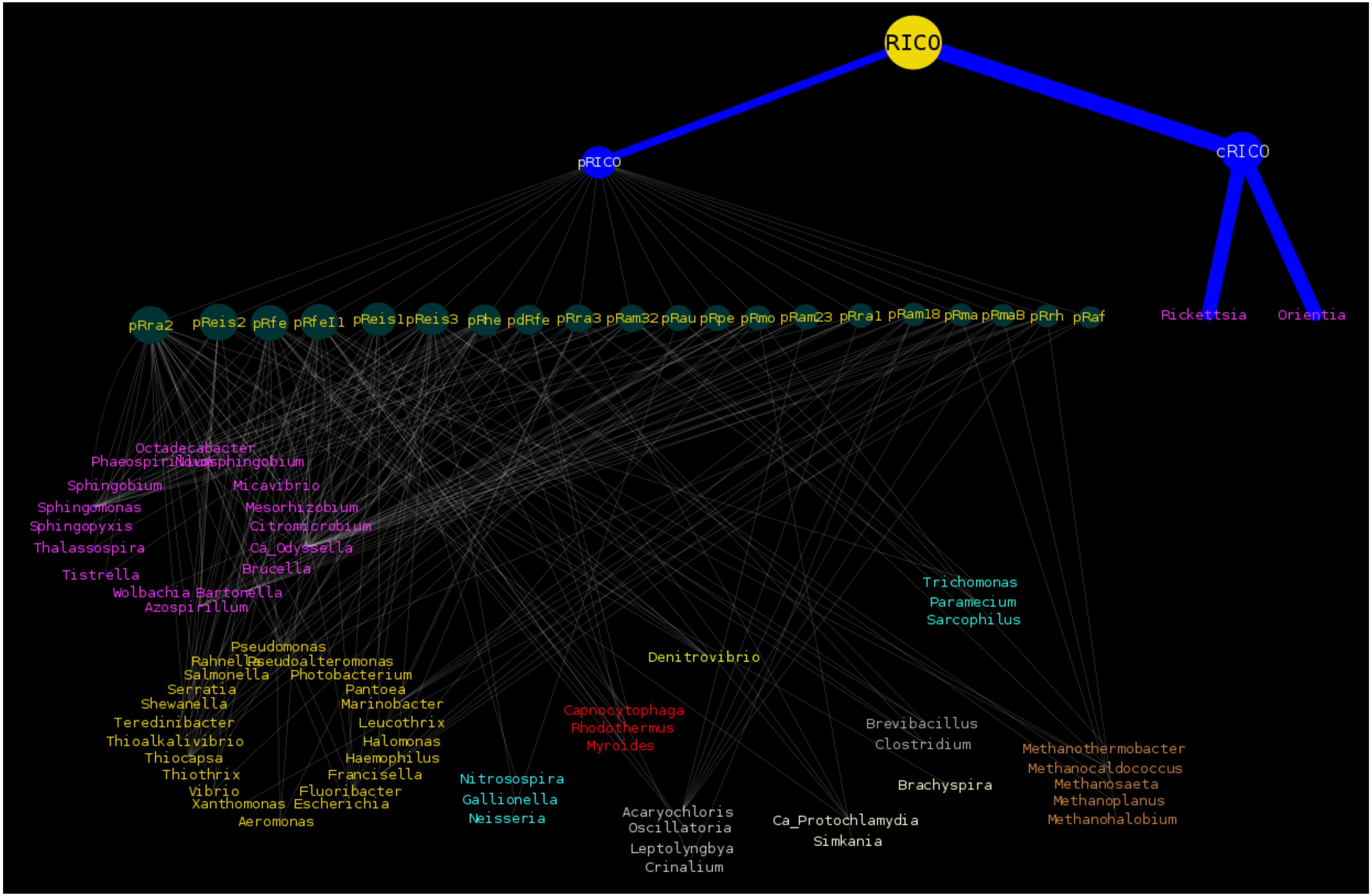

Supplement: S2 File — (PDF) [file pone.0147492.s002.pdf]
