# Supplementary material for: Origin and Evolution of Rickettsial Plasmids
Source: PLoS One. 2016 Feb 11;11(2):e0147492. doi: 10.1371/journal.pone.0147492 (PMC4750851; doi:10.1371/journal.pone.0147492)

Figure A1. Neighbor-joining (NJ) and maximum likelihood (ML) trees of transposases containing tnp_31 domain Tnp. Bootstrap supports higher than or equal to 60% are shown on the branches.

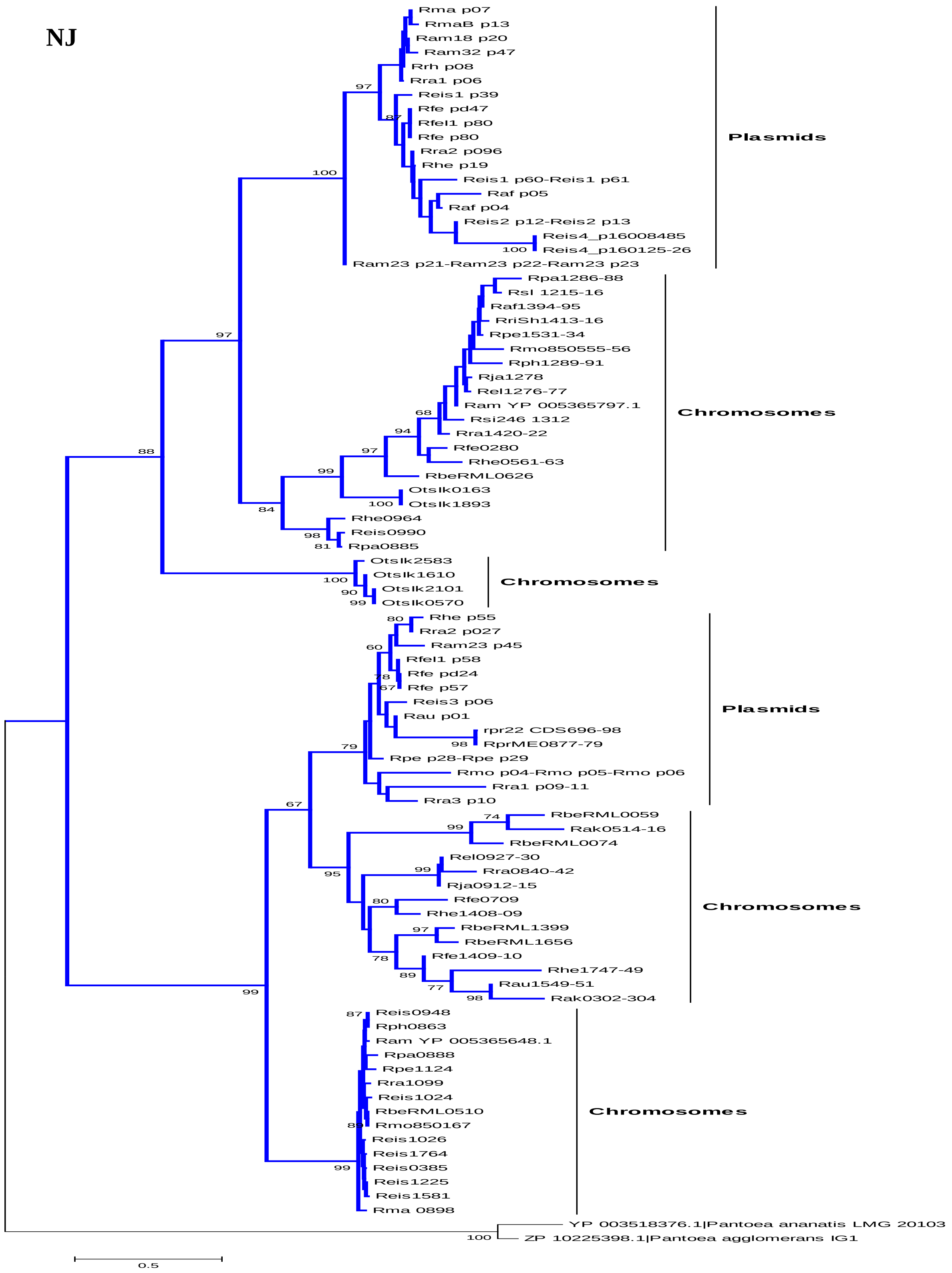

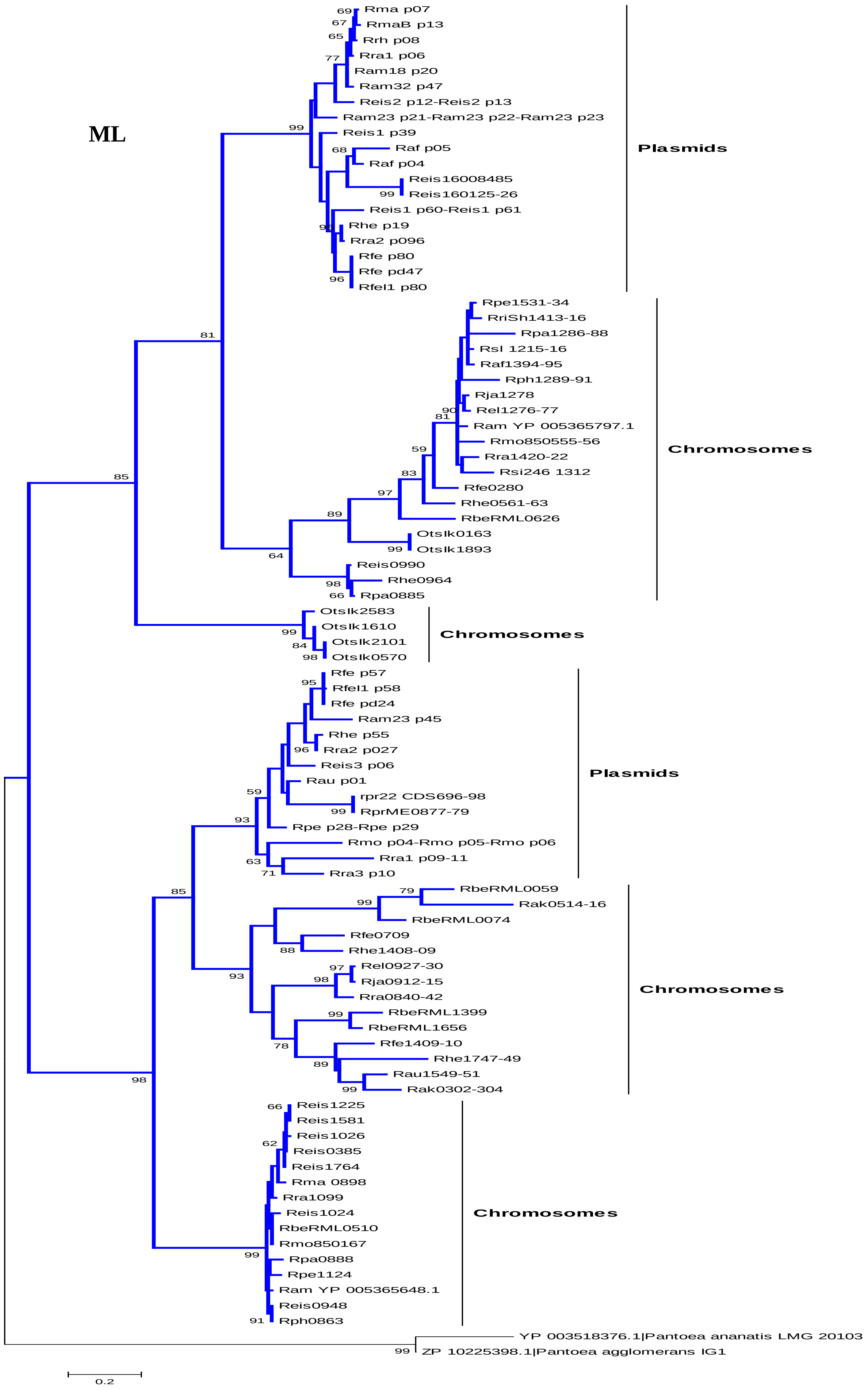
ML
Rma p07
RmaB p13
Rrh p08
Rra1 p06
Ram18 p20
Ram32 p47
Reis2 p12-Reis2 p13
Ram23 p21-Ram23 p22-Ram23 p23
Reis1 p39
Raf p05
Raf p04
Reis16008485
Reis160125-26
Reis1 p60-Reis1 p61
Rhe p19
Rra2 p096
Rfe p80
Rfe pd47
Rfel1 p80
Plasmids
Rpe1531-34
RriSh1413-16
Rpa1286-88
Rsl 1215-16
Raf1394-95
Rph1289-91
Rja1278
Rel1276-77
Ram YP 005365797.1
Rmo850555-56
Rra1420-22
Rsi246 1312
Rfe0280
Rhe0561-63
RbeRML0626
OtsIk0163
OtsIk1893
Reis0990
Rhe0964
Rpa0885
Chromosomes
OtsIk2583
OtsIk1610
OtsIk2101
OtsIk0570
Chromosomes
Rfe p57
Rfel1 p58
Rfe pd24
Ram23 p45
Rhe p55
Rra2 p027
Reis3 p06
Rau p01
rpr22 CDS696-98
RprME0877-79
Rpe p28-Rpe p29
Rmo p04-Rmo p05-Rmo p06
Rra1 p09-11
Rra3 p10
Plasmids
RbeRML0059
Rak0514-16
RbeRML0074
Rfe0709
Rhe1408-09
Rel0927-30
Rja0912-15
Rra0840-42
RbeRML1399
RbeRML1656
Rfe1409-10
Rhe1747-49
Rau1549-51
Rak0302-304
Chromosomes
Reis1225
Reis1581
Reis1026
Reis0385
Reis1764
Rma 0898
Rra1099
Reis1024
RbeRML0510
Rmo850167
Rpa0888
Rpe1124
Ram YP 005365648.1
Reis0948
Rph0863
Chromosomes
YP 003518376.1|Pantoea ananatis LMG 20103
ZP 10225398.1|Pantoea agglomerans IG1
0.2

Figure A2. Neighbor-joining (NJ) and maximum likelihood (ML) trees of patatin-like phospholipases Pat. Bootstrap supports higher than or equal to 60% are shown on the branches.

NJ

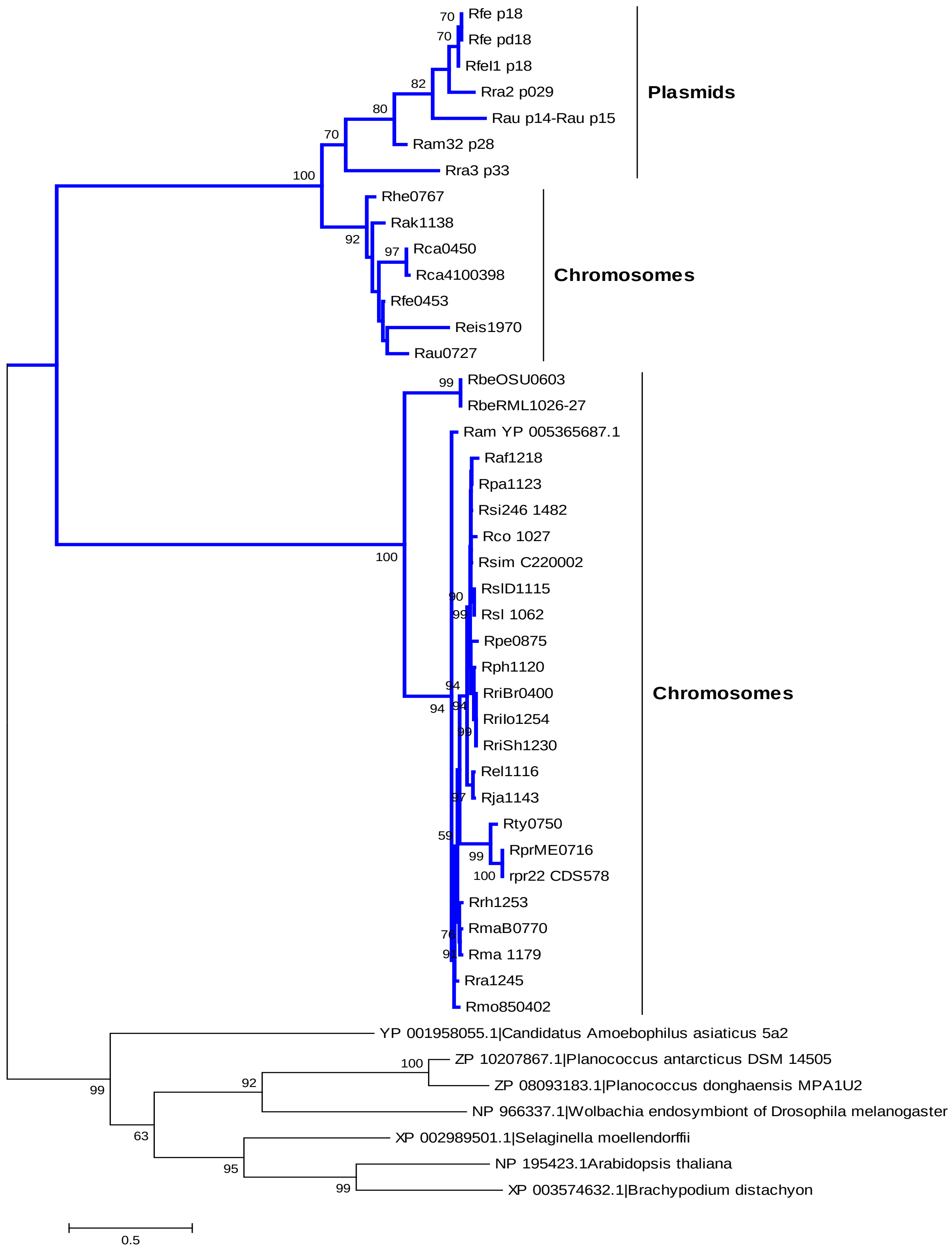

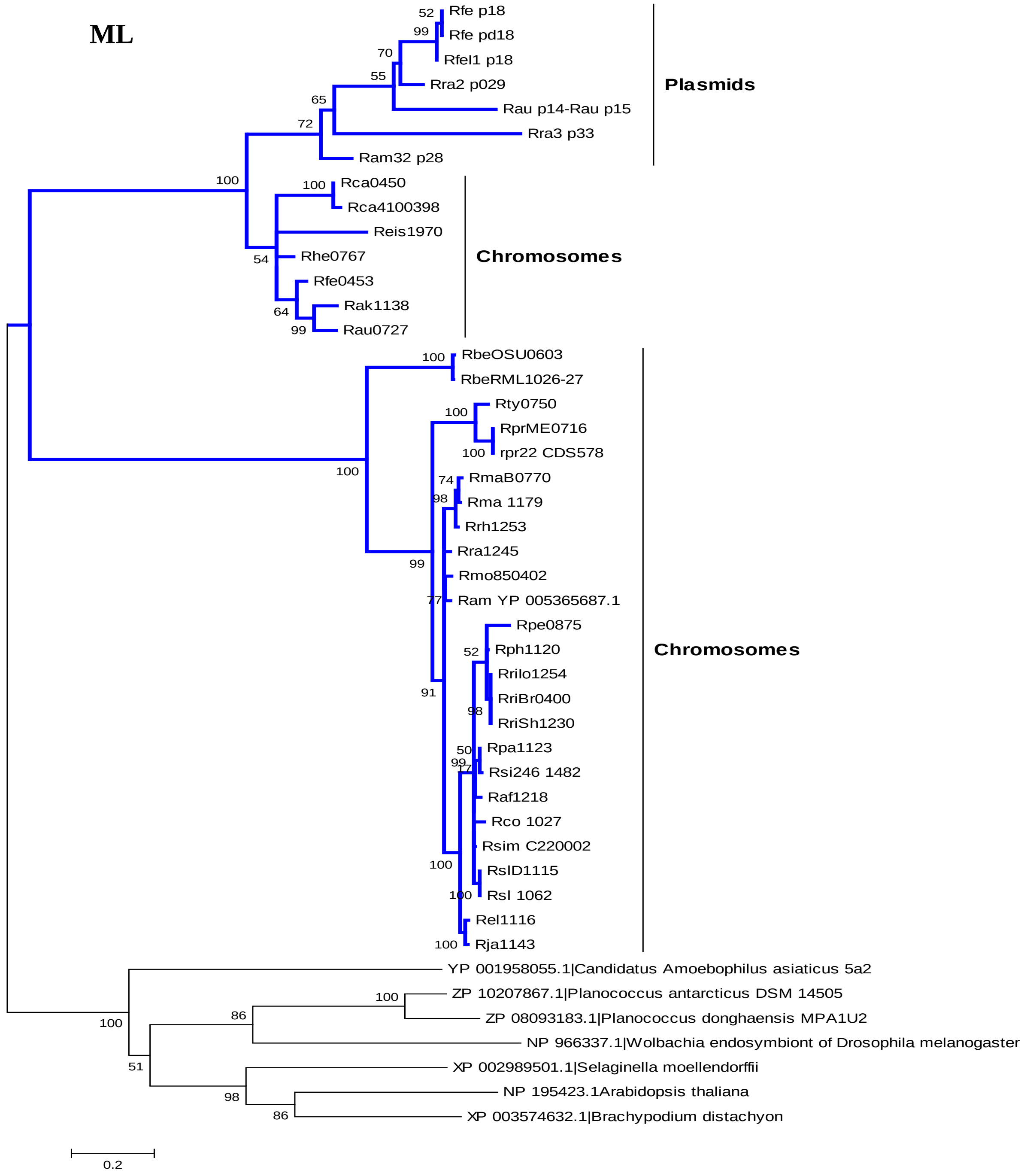
ML
Rfe p18
Rfe pd18
Rfel1 p18
Rra2 p029
Rau p14-Rau p15
Rra3 p33
Ram32 p28
Plasmids
Rca0450
Rca4100398
Reis1970
Rhe0767
Rfe0453
Rak1138
Rau0727
Chromosomes
RbeOSU0603
RbeRML1026-27
Rty0750
RprME0716
rpr22 CDS578
RmaB0770
Rma 1179
Rrh1253
Rra1245
Rmo850402
Ram YP 005365687.1
Rpe0875
Rph1120
Rrilo1254
RriBr0400
RriSh1230
Rpa1123
Rsi246 1482
Raf1218
Rco 1027
Rsim C220002
RslD1115
Rsl 1062
Rel1116
Rja1143
Chromosomes
YP 001958055.1|Candidatus Amoebophilus asiaticus 5a2
ZP 10207867.1|Planococcus antarcticus DSM 14505
ZP 08093183.1|Planococcus donghaensis MPA1U2
NP 966337.1|Wolbachia endosymbiont of Drosophila melanogaster
XP 002989501.1|Selaginella moellendorffii
NP 195423.1Arabidopsis thaliana
XP 003574632.1|Brachypodium distachyon
0.2

Figure A3. Neighbor-joining (NJ) and maximum likelihood (ML) trees of thymidylate kinase. Bootstrap supports higher than or equal to 60% are shown on the branches.

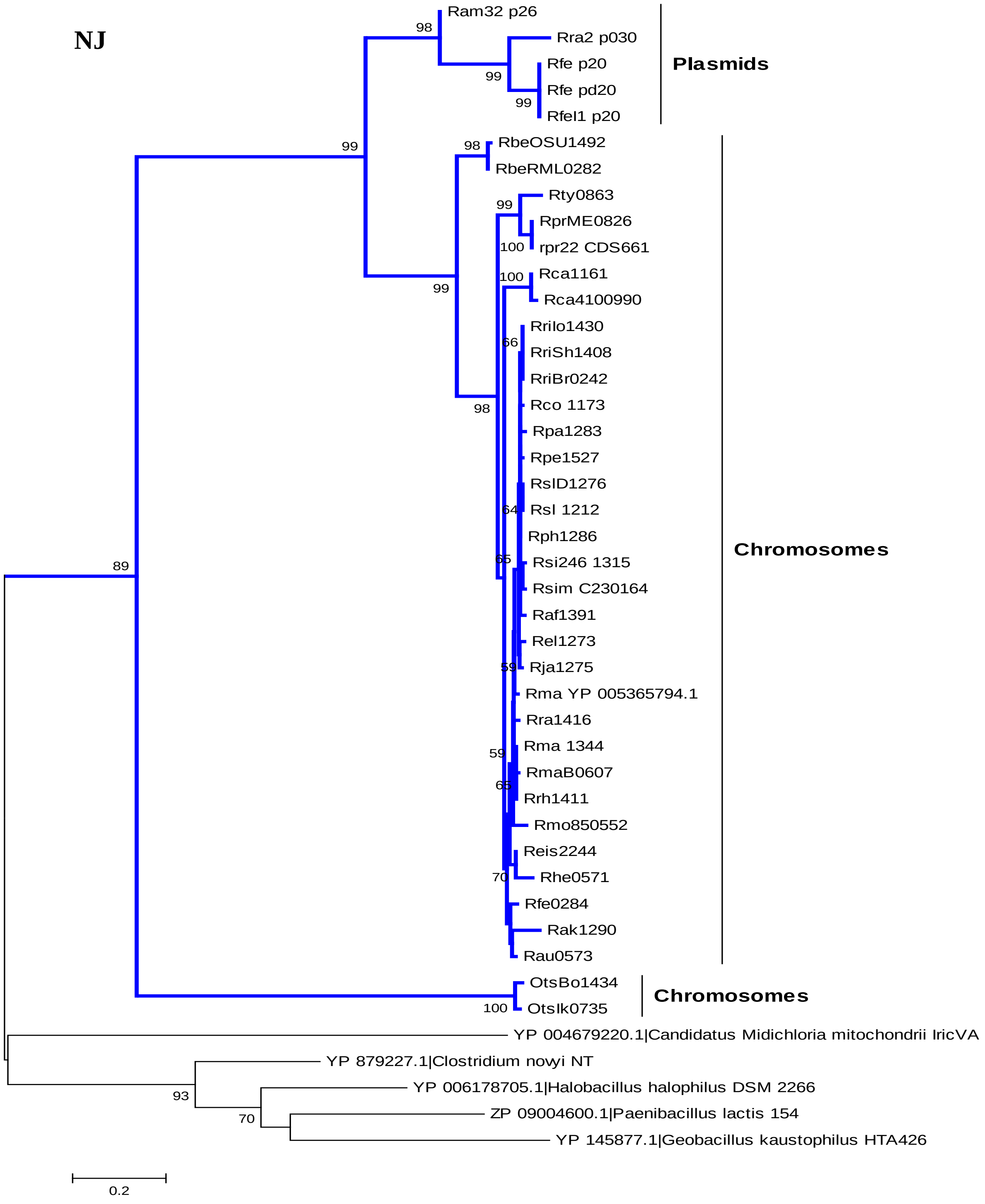

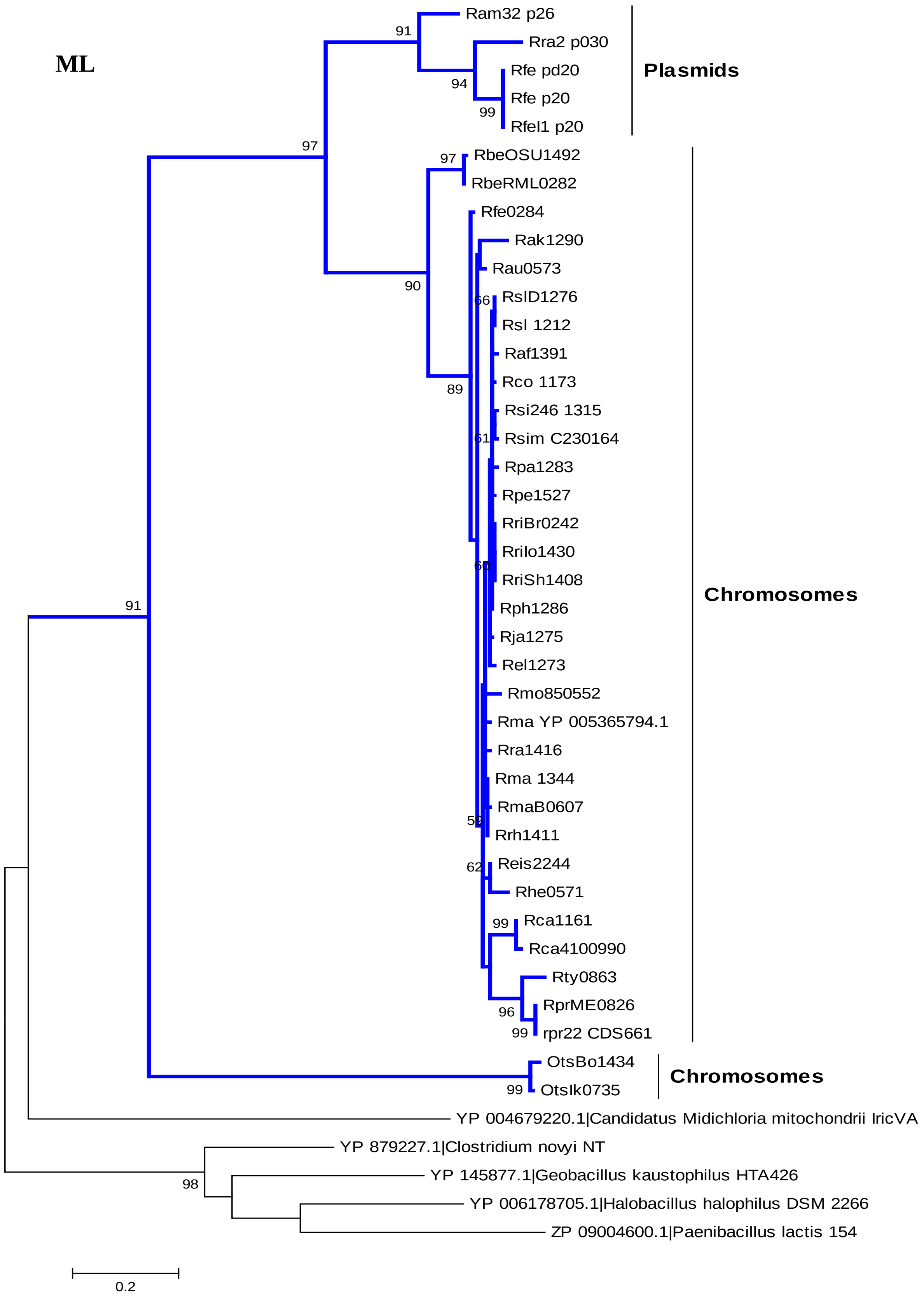

ML
Ram32 p26
Rra2 p030
Rfe pd20
Rfe p20
Rfel1 p20
Plasmids
RbeOSU1492
RbeRML0282
Rfe0284
Rak1290
Rau0573
RsID1276
Rsl 1212
Raf1391
Rco 1173
Rsi246 1315
Rsim C230164
Rpa1283
Rpe1527
RriBr0242
RriIo1430
RriSh1408
Rph1286
Rja1275
Rel1273
Rmo850552
Rma YP 005365794.1
Rra1416
Rma 1344
RmaB0607
Rrh1411
Reis2244
Rhe0571
Rca1161
Rca4100990
Rty0863
RprME0826
rpr22 CDS661
Chromosomes
OtsBo1434
OtsIk0735
Chromosomes
YP 004679220.1|Candidatus Midichloria mitochondrii IricVA
YP 879227.1|Clostridium novyi NT
YP 145877.1|Geobacillus kaustophilus HTA426
YP 006178705.1|Halobacillus halophilus DSM 2266
ZP 09004600.1|Paenibacillus lactis 154
0.2

Figure A4. Neighbor-joining (NJ) and maximum likelihood (ML) trees of heat shock protein Hsps. Bootstrap supports higher than or equal to 60% are shown on the branches.

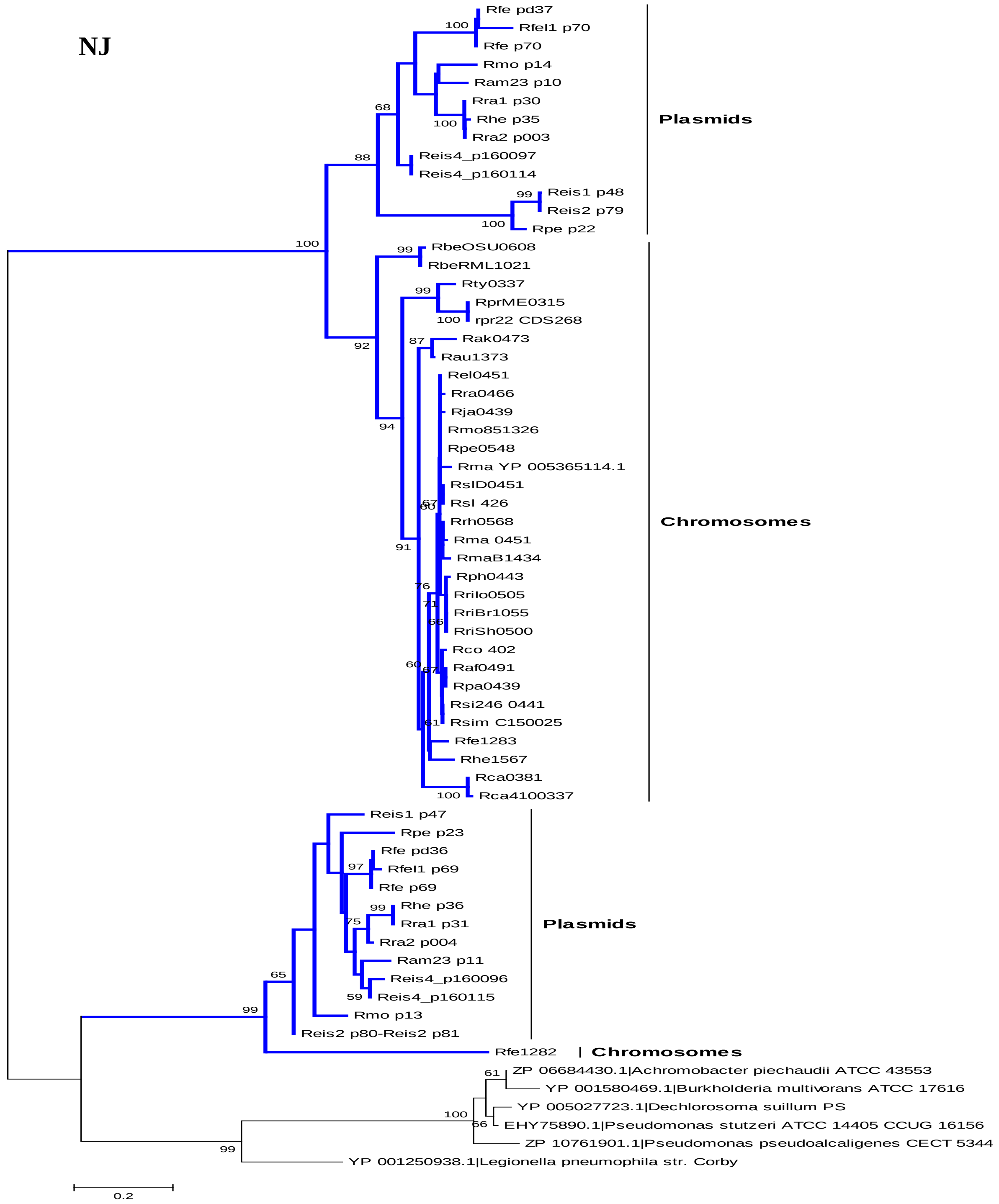

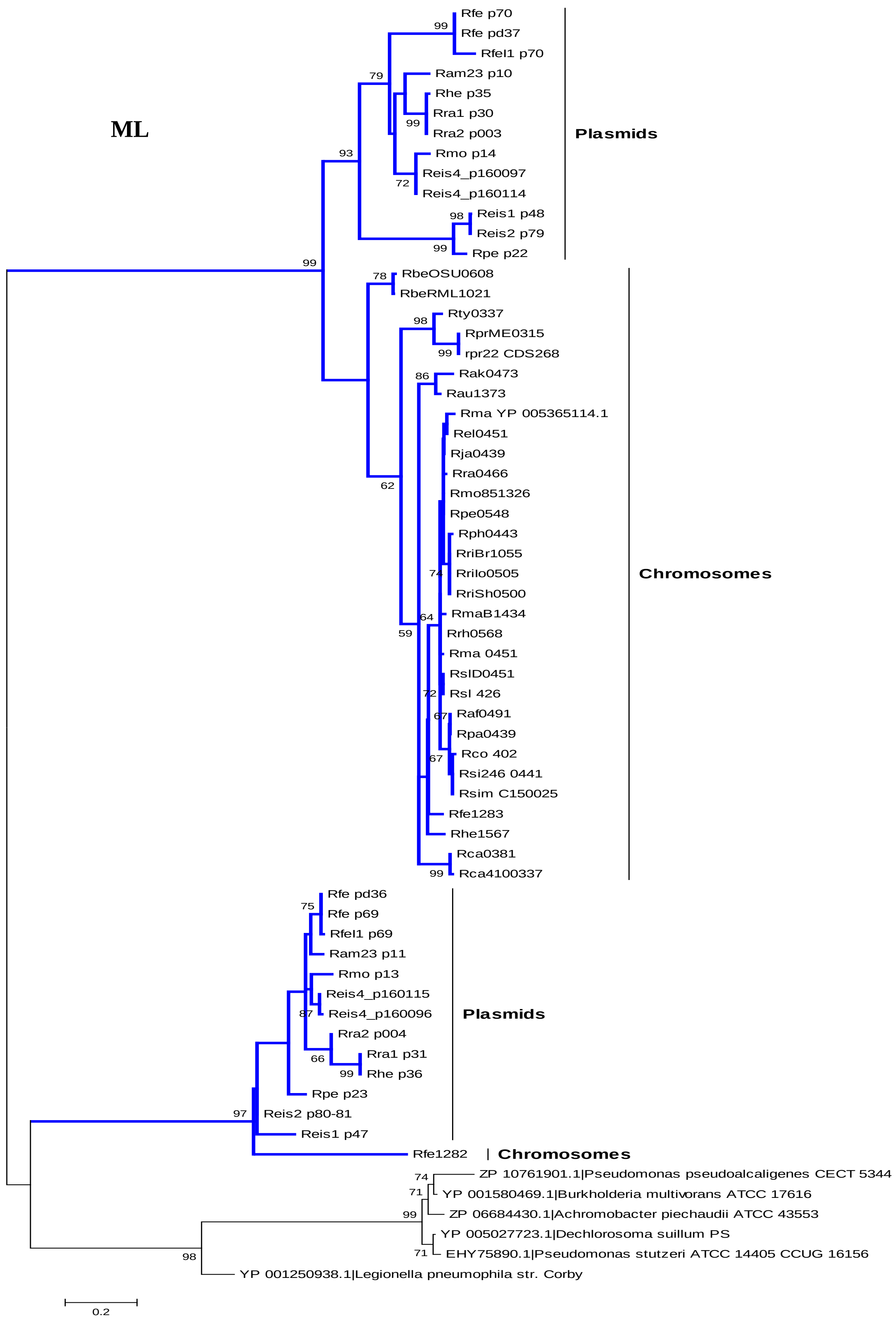
ML
Rfe p70
Rfe pd37
Rfel1 p70
Ram23 p10
Rhe p35
Rra1 p30
Rra2 p003
Rmo p14
Reis4_p160097
Reis4_p160114
Reis1 p48
Reis2 p79
Rpe p22
Plasmids
RbeOSU0608
RbeRML1021
Rty0337
RprME0315
rpr22 CDS268
Rak0473
Rau1373
Rma YP 005365114.1
Rel0451
Rja0439
Rra0466
Rmo851326
Rpe0548
Rph0443
RriBr1055
RriIo0505
RriSh0500
RmaB1434
Rrh0568
Rma 0451
RsID0451
RsI 426
Raf0491
Rpa0439
Rco 402
Rsi246 0441
Rsim C150025
Rfe1283
Rhe1567
Rca0381
Rca4100337
Chromosomes
Rfe pd36
Rfe p69
Rfel1 p69
Ram23 p11
Rmo p13
Reis4_p160115
Reis4_p160096
Rra2 p004
Rra1 p31
Rhe p36
Rpe p23
Reis2 p80-81
Reis1 p47
Plasmids
Rfe1282
Chromosomes
ZP 10761901.1|Pseudomonas pseudoalcaligenes CECT 5344
YP 001580469.1|Burkholderia multivorans ATCC 17616
ZP 06684430.1|Achromobacter piechaudii ATCC 43553
YP 005027723.1|Dechlorosoma suillum PS
EHY75890.1|Pseudomonas stutzeri ATCC 14405 CCUG 16156
YP 001250938.1|Legionella pneumophila str. Corby
0.2

Figure A5. Neighbor-joining (NJ) and maximum likelihood (ML) trees of *dna*A-like replication initiator protein. Bootstrap supports higher than or equal to 60% are shown on the branches. The short sequences of about 318 aa of *R. prowazekii* strains were included in the the third NJ tree of *dna*A-like domain and its flaking regions.

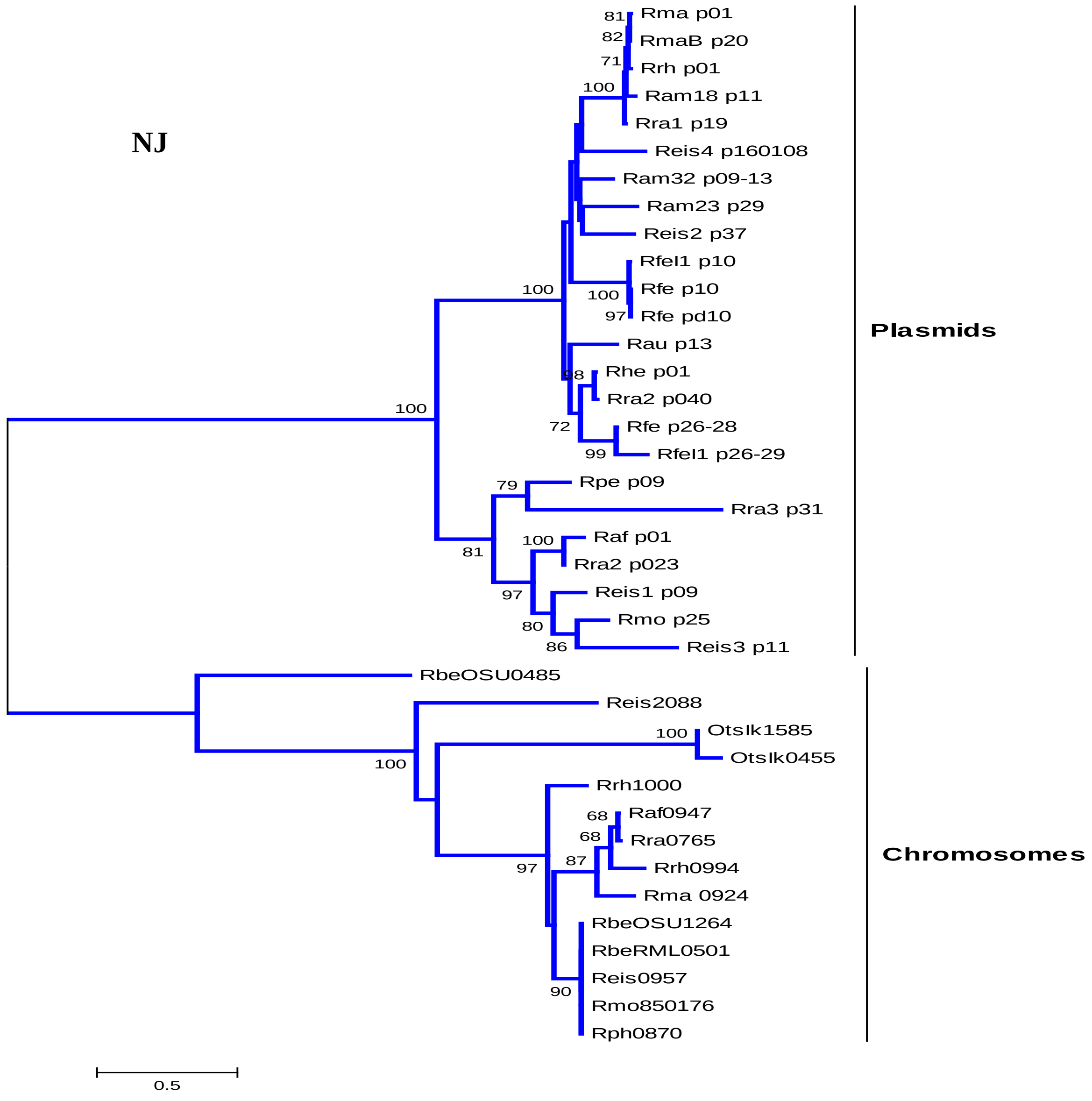

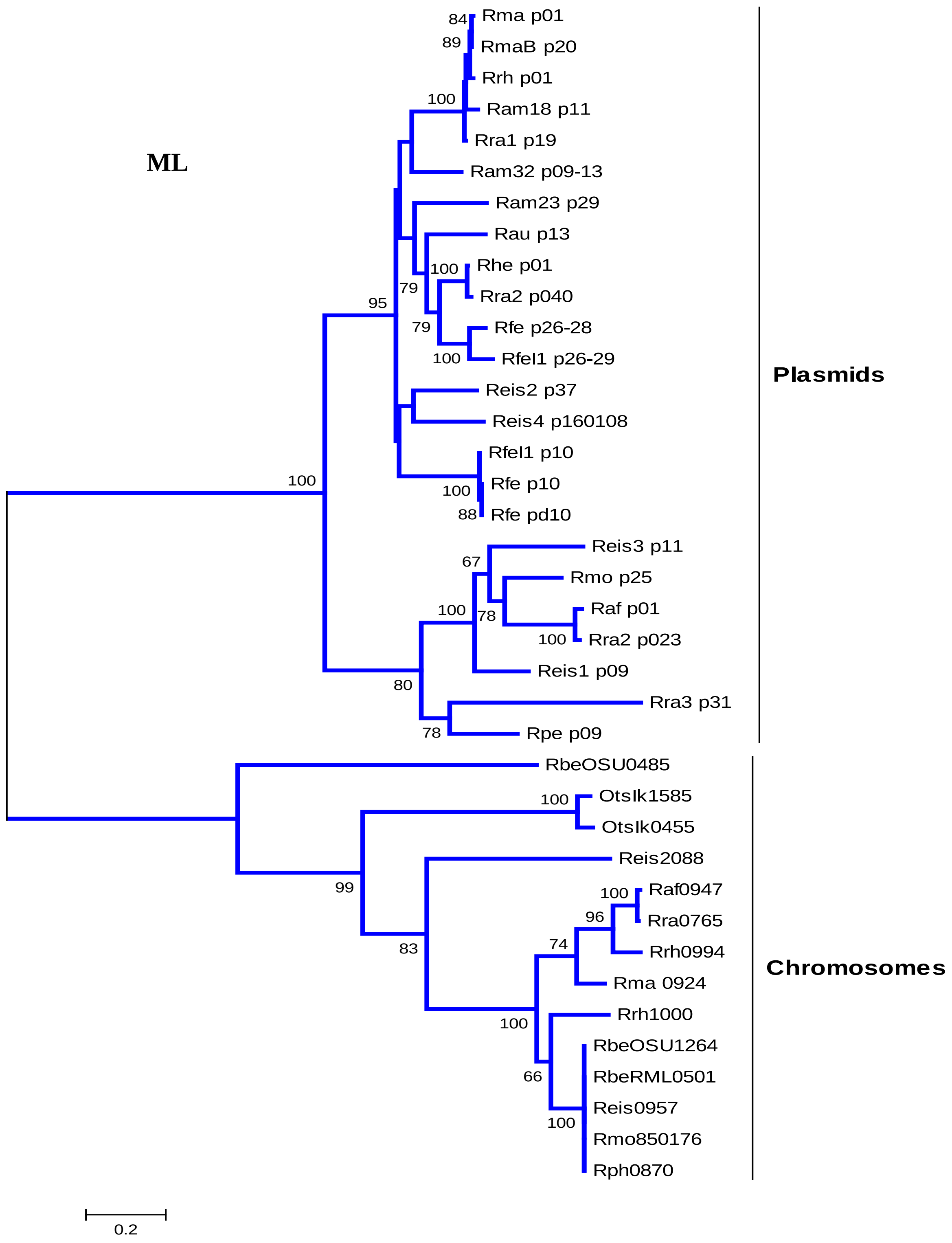
ML
Rma p01
RmaB p20
Rrh p01
Ram18 p11
Rra1 p19
Ram32 p09-13
Ram23 p29
Rau p13
Rhe p01
Rra2 p040
Rfe p26-28
Rfel1 p26-29
Reis2 p37
Reis4 p160108
Rfel1 p10
Rfe p10
Rfe pd10
Reis3 p11
Rmo p25
Raf p01
Rra2 p023
Reis1 p09
Rra3 p31
Rpe p09
RbeOSU0485
OtsIk1585
OtsIk0455
Reis2088
Raf0947
Rra0765
Rrh0994
Rma 0924
Rrh1000
RbeOSU1264
RbeRML0501
Reis0957
Rmo850176
Rph0870
Plasmids
Chromosomes
0.2

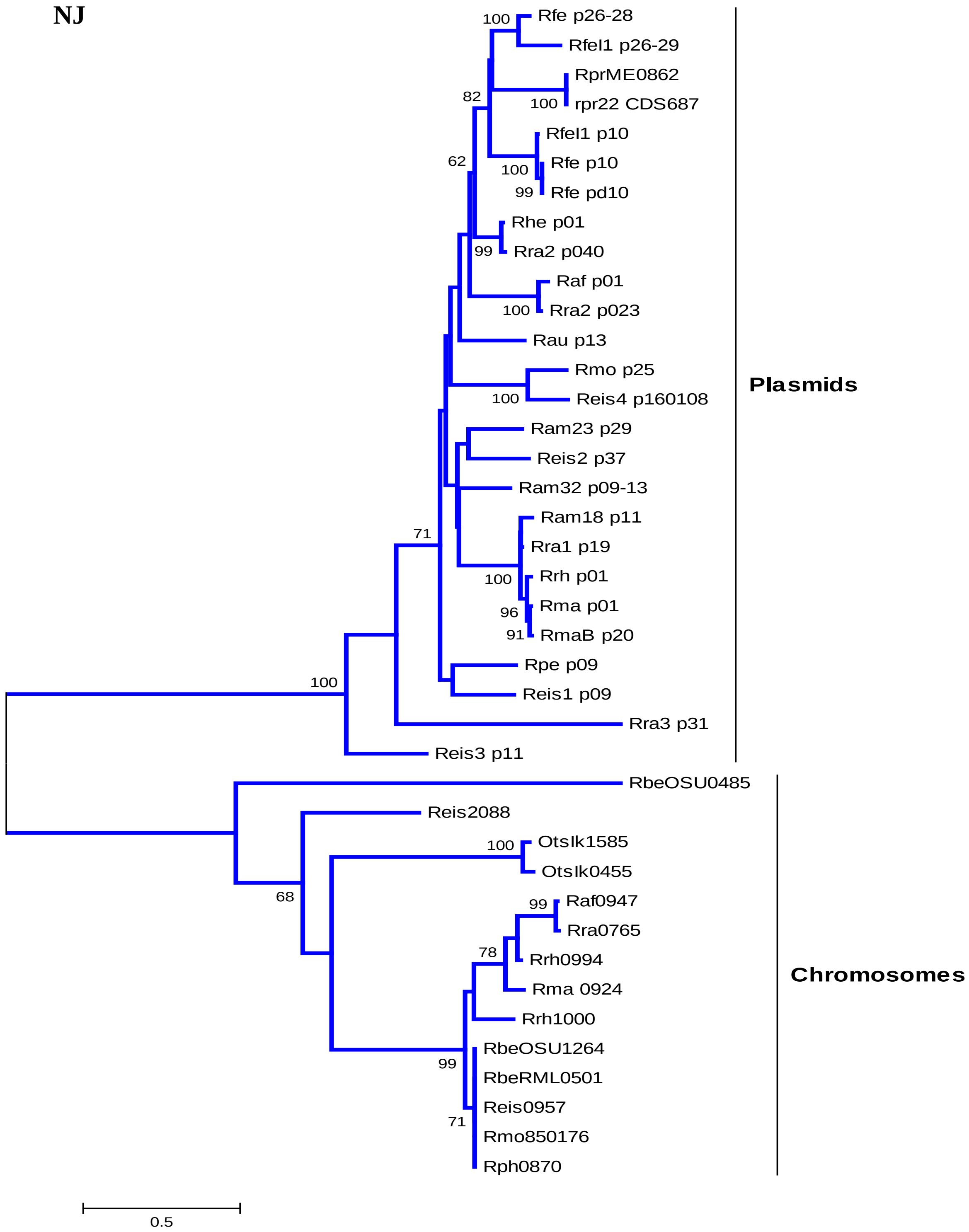
NJ
Rfe p26-28
Rfel1 p26-29
RprME0862
rpr22 CDS687
Rfel1 p10
Rfe p10
Rfe pd10
Rhe p01
Rra2 p040
Raf p01
Rra2 p023
Rau p13
Rmo p25
Reis4 p160108
Ram23 p29
Reis2 p37
Ram32 p09-13
Ram18 p11
Rra1 p19
Rrh p01
Rma p01
RmaB p20
Rpe p09
Reis1 p09
Rra3 p31
Reis3 p11
RbeOSU0485
Reis2088
OtsIk1585
OtsIk0455
Raf0947
Rra0765
Rrh0994
Rma 0924
Rrh1000
RbeOSU1264
RbeRML0501
Reis0957
Rmo850176
Rph0870
Plasmids
Chromosomes
100
82
100
62
100
99
99
100
100
100
71
100
96
91
100
100
99
78
68
99
71
0.5

Figure A6. Neighbor-joining (NJ) and maximum likelihood (ML) trees of helix-turn-helix DNA-binding domain. Bootstrap supports higher than or equal to 60% are shown on the branches.

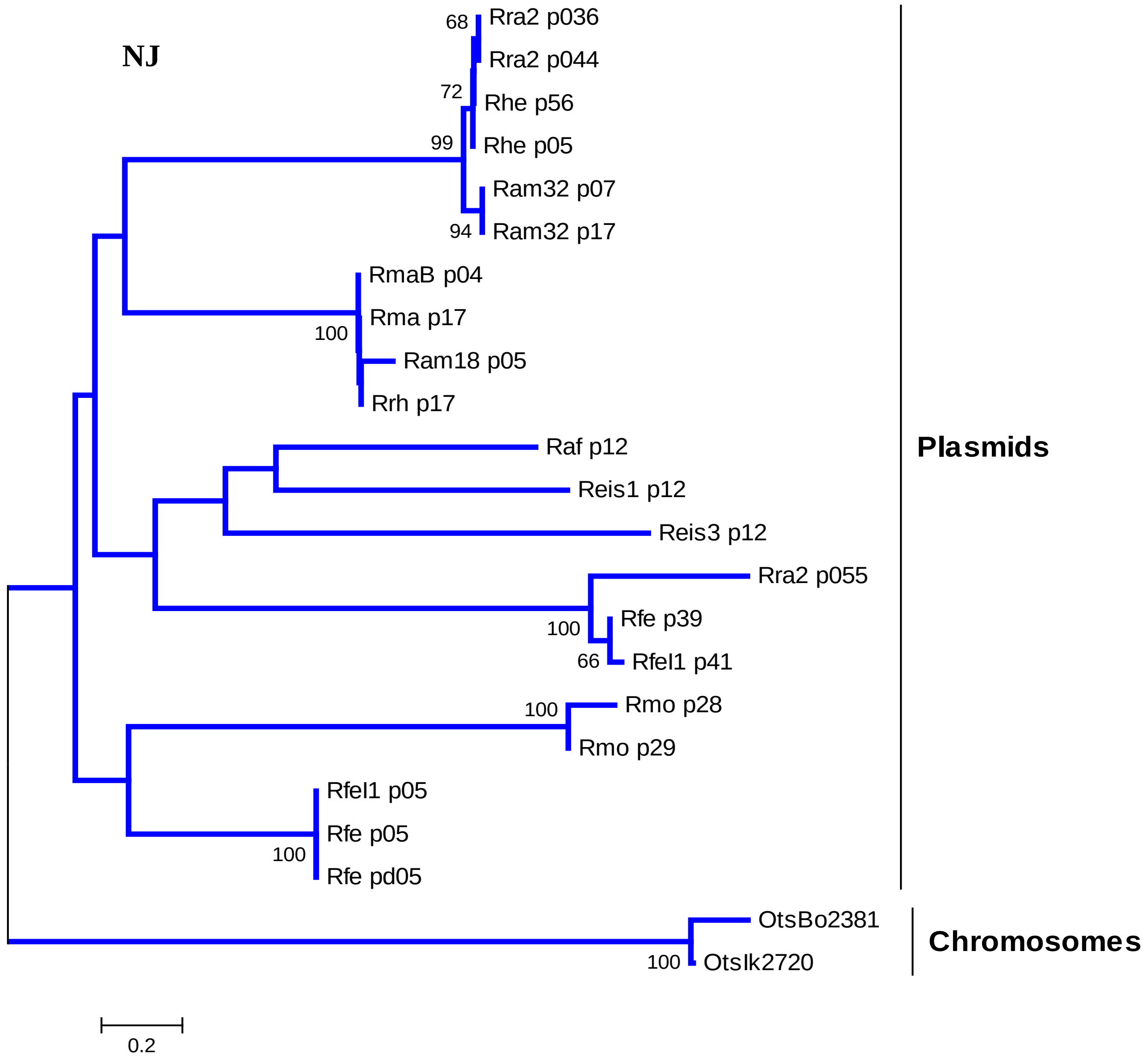

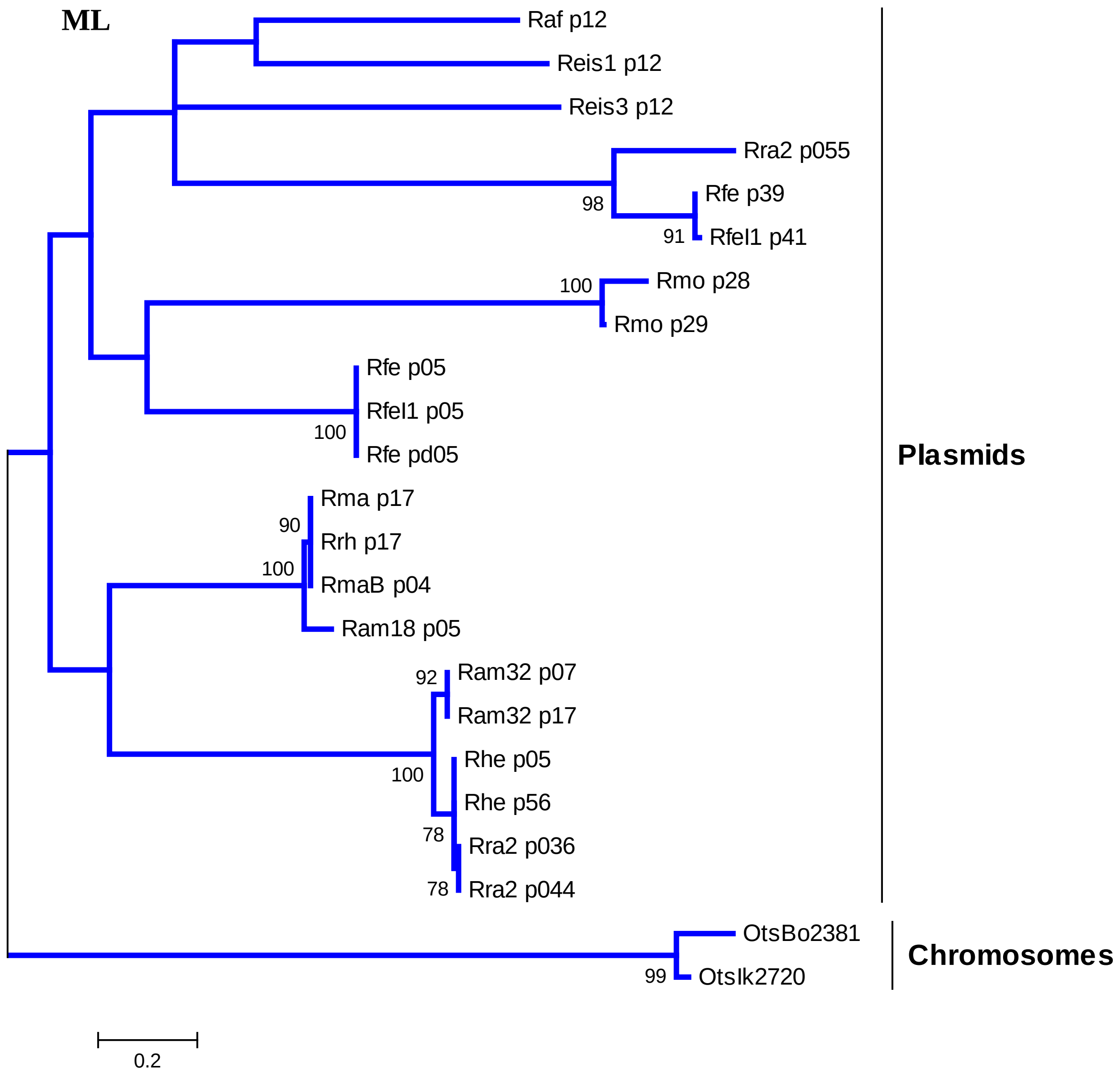

ML
Raf p12
Reis1 p12
Reis3 p12
Rra2 p055
98
Rfe p39
91
RfeI1 p41
100
Rmo p28
Rmo p29
Rfe p05
RfeI1 p05
100
Rfe pd05
Rma p17
90
Rrh p17
100
RmaB p04
Ram18 p05
92
Ram32 p07
Ram32 p17
Rhe p05
100
Rhe p56
78
Rra2 p036
78
Rra2 p044
OtsBo2381
99
OtsIk2720
Plasmids
Chromosomes
0.2

Figure A7. Neighbor-joining (NJ) and maximum likelihood (ML) trees of the autotransporter region of cell surface antigen Sca12. Bootstrap supports higher than or equal to 60% are shown on the branches.

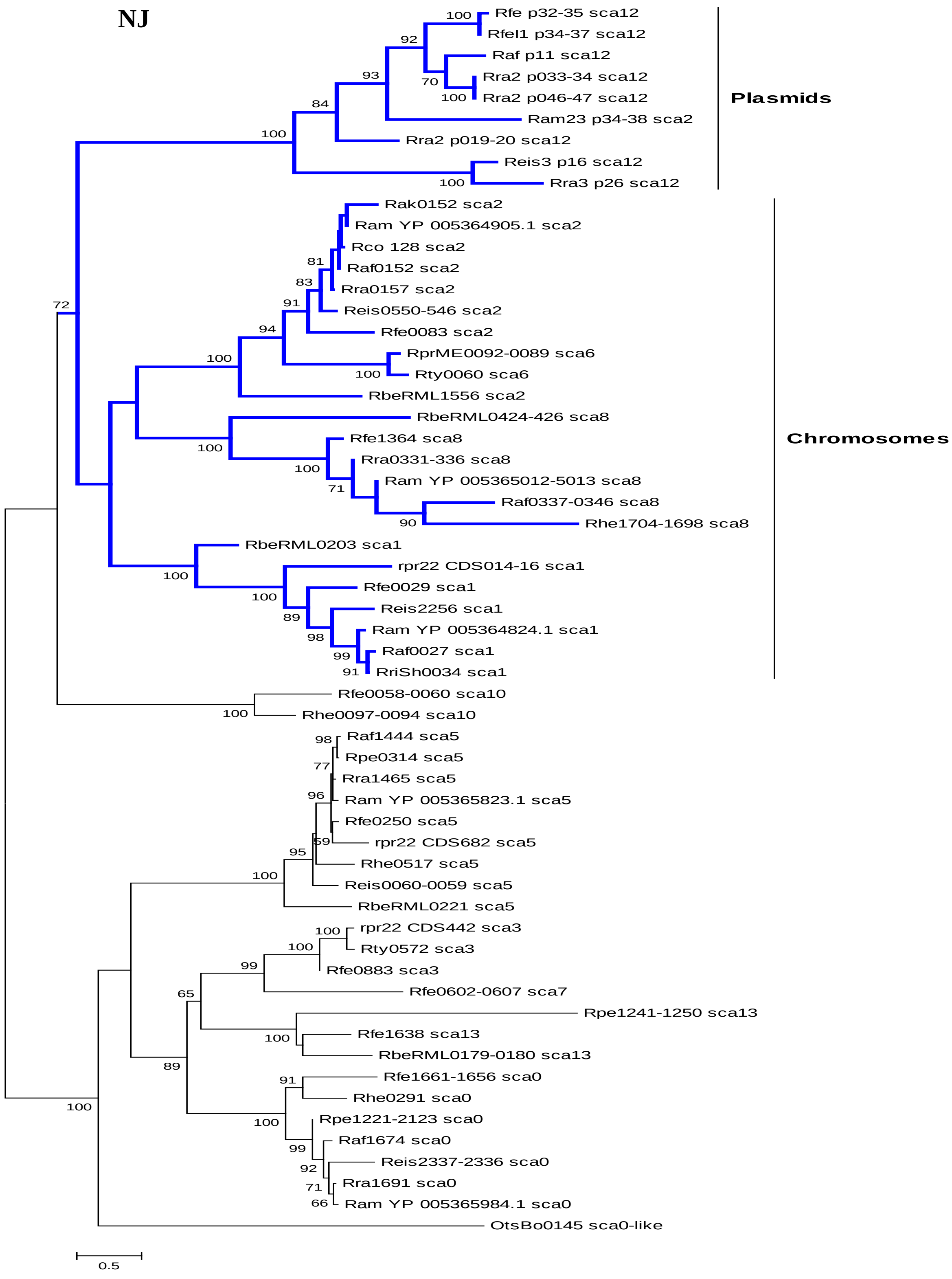

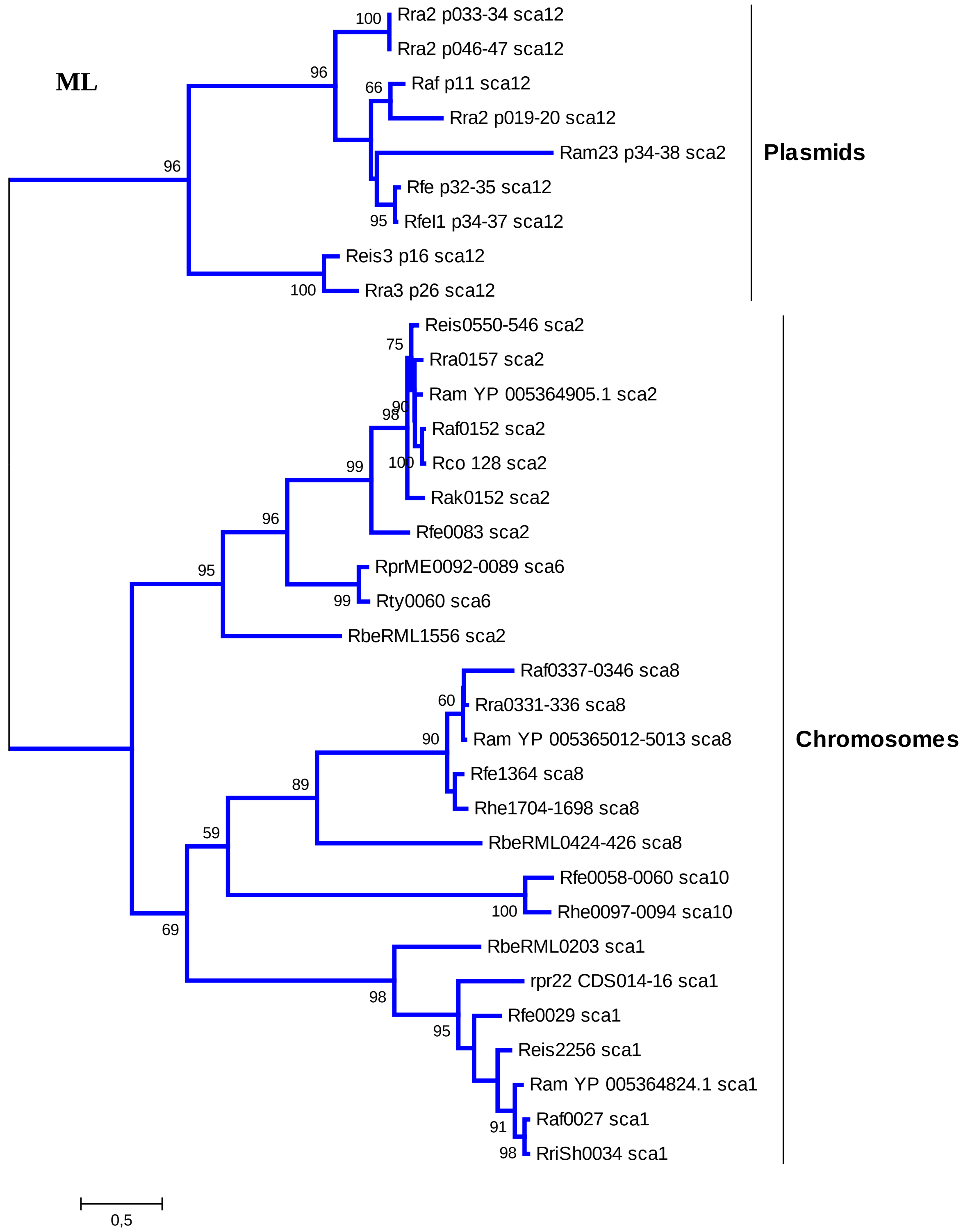

ML
Rra2 p033-34 sca12
Rra2 p046-47 sca12
Raf p11 sca12
Rra2 p019-20 sca12
Ram23 p34-38 sca2
Rfe p32-35 sca12
Rfel1 p34-37 sca12
Reis3 p16 sca12
Rra3 p26 sca12
Plasmids
Reis0550-546 sca2
Rra0157 sca2
Ram YP 005364905.1 sca2
Raf0152 sca2
Rco 128 sca2
Rak0152 sca2
Rfe0083 sca2
RprME0092-0089 sca6
Rty0060 sca6
RbeRML1556 sca2
Raf0337-0346 sca8
Rra0331-336 sca8
Ram YP 005365012-5013 sca8
Rfe1364 sca8
Rhe1704-1698 sca8
RbeRML0424-426 sca8
Rfe0058-0060 sca10
Rhe0097-0094 sca10
RbeRML0203 sca1
rpr22 CDS014-16 sca1
Rfe0029 sca1
Reis2256 sca1
Ram YP 005364824.1 sca1
Raf0027 sca1
RriSh0034 sca1
Chromosomes
0,5

Figure A8. Neighbor-joining (NJ) and maximum likelihood (ML) trees of conjugative transfer protein containing TraD domain. Bootstrap supports higher than or equal to 60% are shown on the branches.

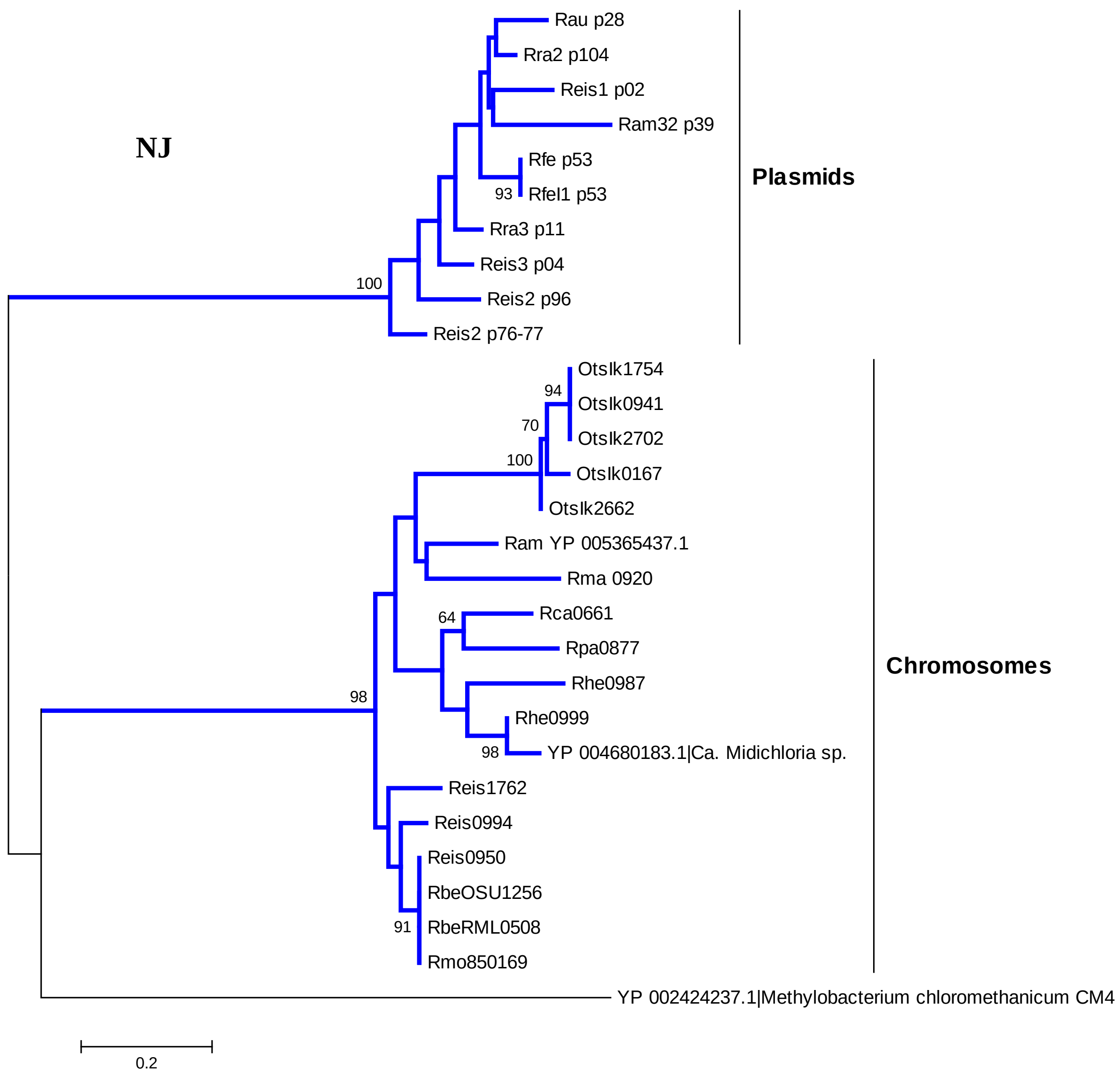

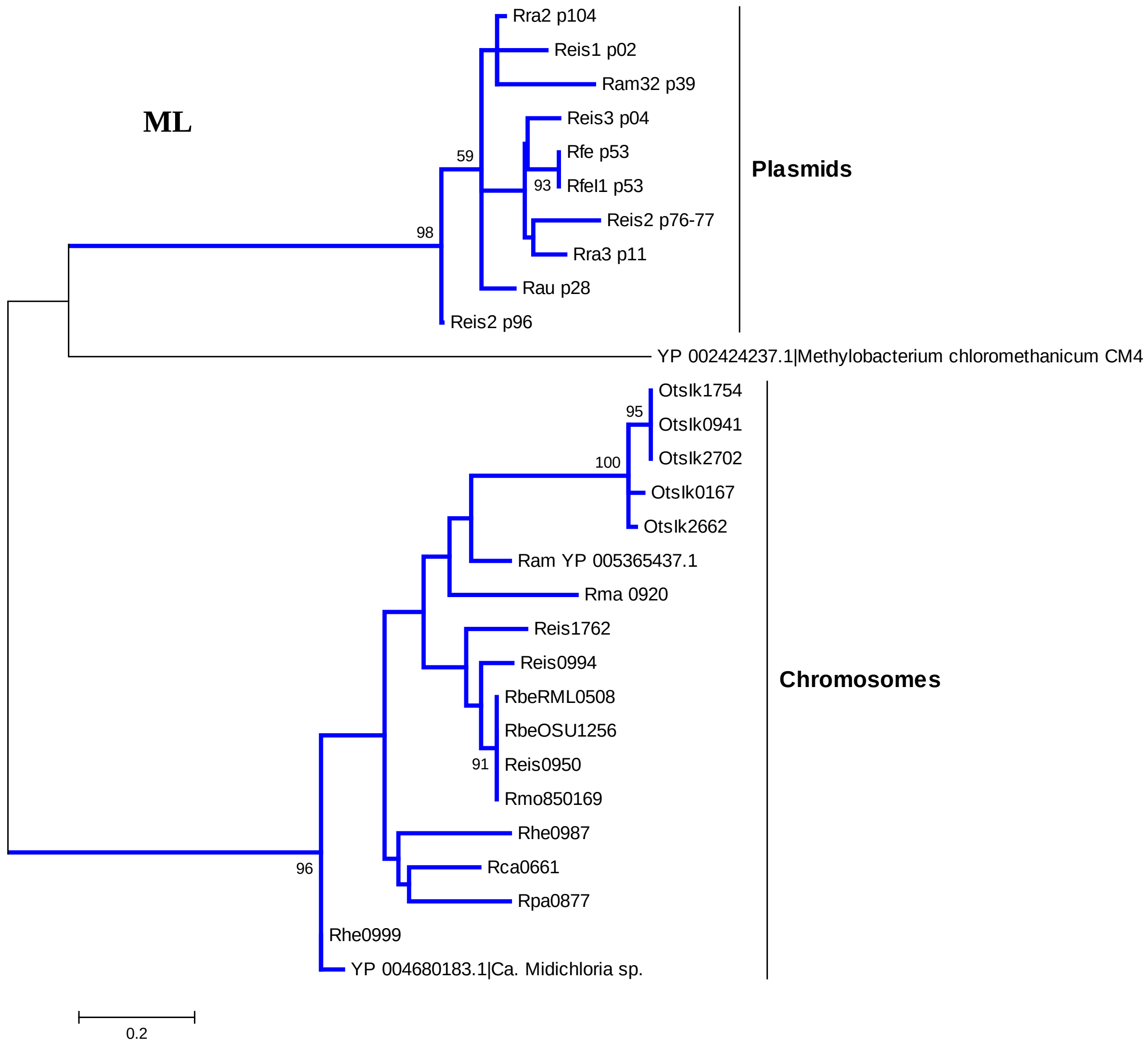
ML
Rra2 p104
Reis1 p02
Ram32 p39
Reis3 p04
Rfe p53
Rfel1 p53
Reis2 p76-77
Rra3 p11
Rau p28
Reis2 p96
59
93
98
Plasmids
YP 002424237.1|Methylobacterium chloromethanicum CM4
OtsIk1754
OtsIk0941
OtsIk2702
OtsIk0167
OtsIk2662
95
100
Ram YP 005365437.1
Rma 0920
Reis1762
Reis0994
RbeRML0508
RbeOSU1256
Reis0950
Rmo850169
91
Rhe0987
Rca0661
Rpa0877
Rhe0999
96
YP 004680183.1|Ca. Midichloria sp.
Chromosomes
0.2

Figure A9. Neighbor-joining (NJ) and maximum likelihood (ML) trees of leucine rich-repeat containing protein. Bootstrap supports higher than or equal to 60% are shown on the branches.

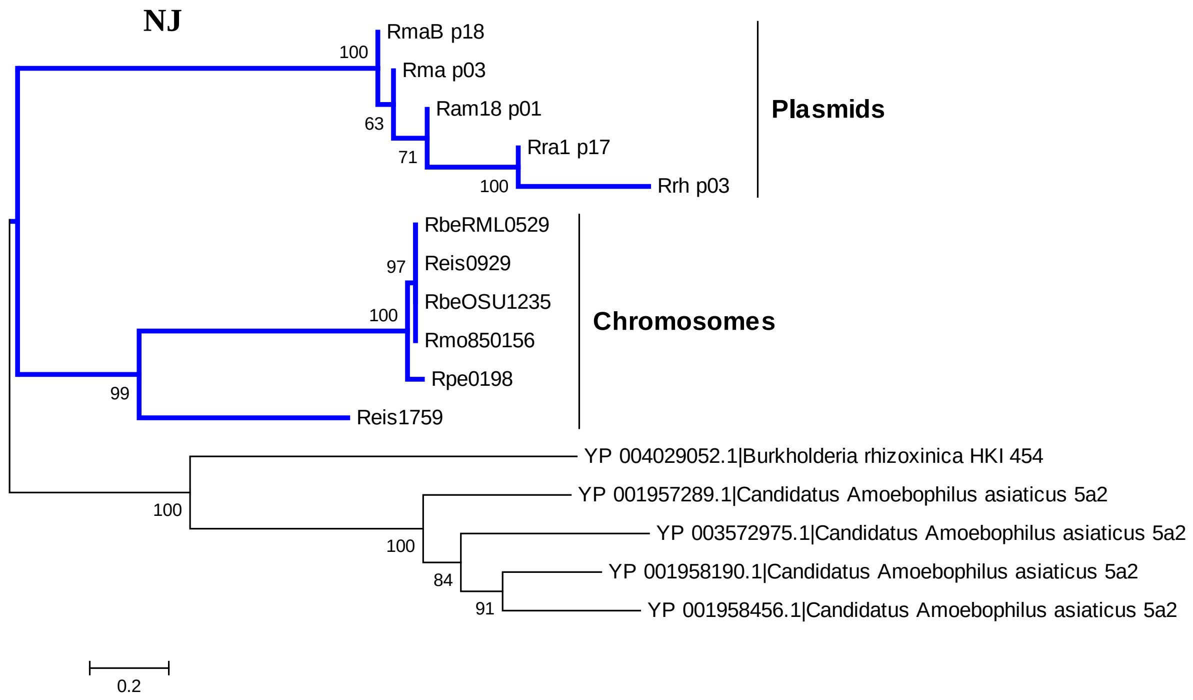

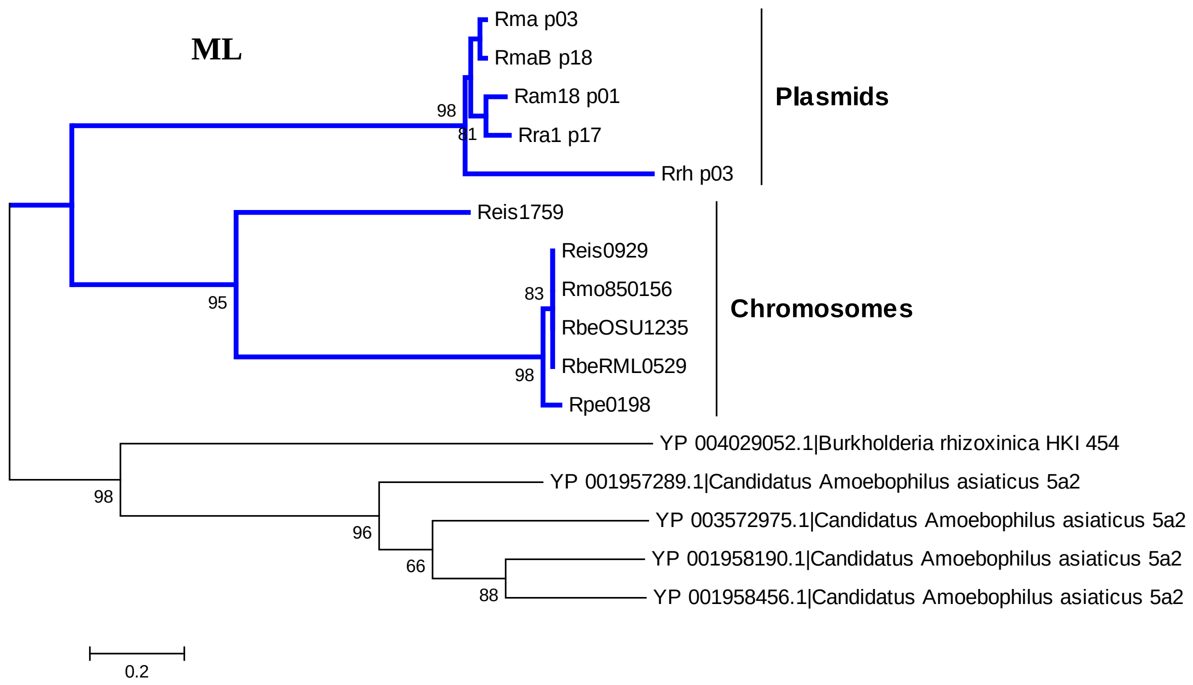
ML
Rma p03
RmaB p18
Ram18 p01
Rra1 p17
Rrh p03
Plasmids
98
81
Reis1759
Reis0929
Rmo850156
RbeOSU1235
RbeRML0529
Rpe0198
Chromosomes
95
83
98
YP 004029052.1|Burkholderia rhizoxinica HKI 454
YP 001957289.1|Candidatus Amoebophilus asiaticus 5a2
YP 003572975.1|Candidatus Amoebophilus asiaticus 5a2
YP 001958190.1|Candidatus Amoebophilus asiaticus 5a2
YP 001958456.1|Candidatus Amoebophilus asiaticus 5a2
98
96
66
88
0.2

Figure A10. Neighbor-joining (NJ) and maximum likelihood (ML) trees of transposase containing PDDEXK_2 domain. Bootstrap supports higher than or equal to 60% are shown on the branches.

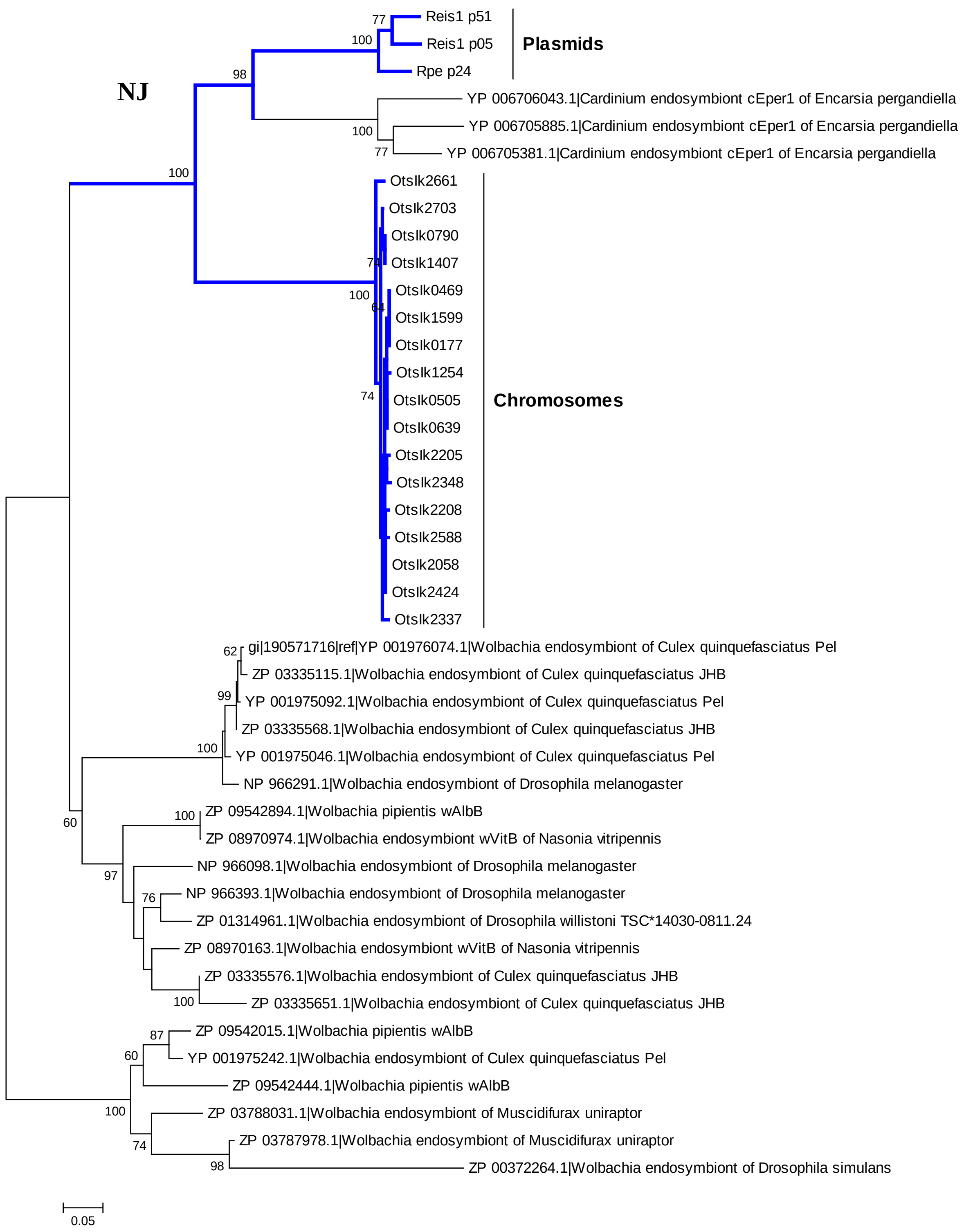

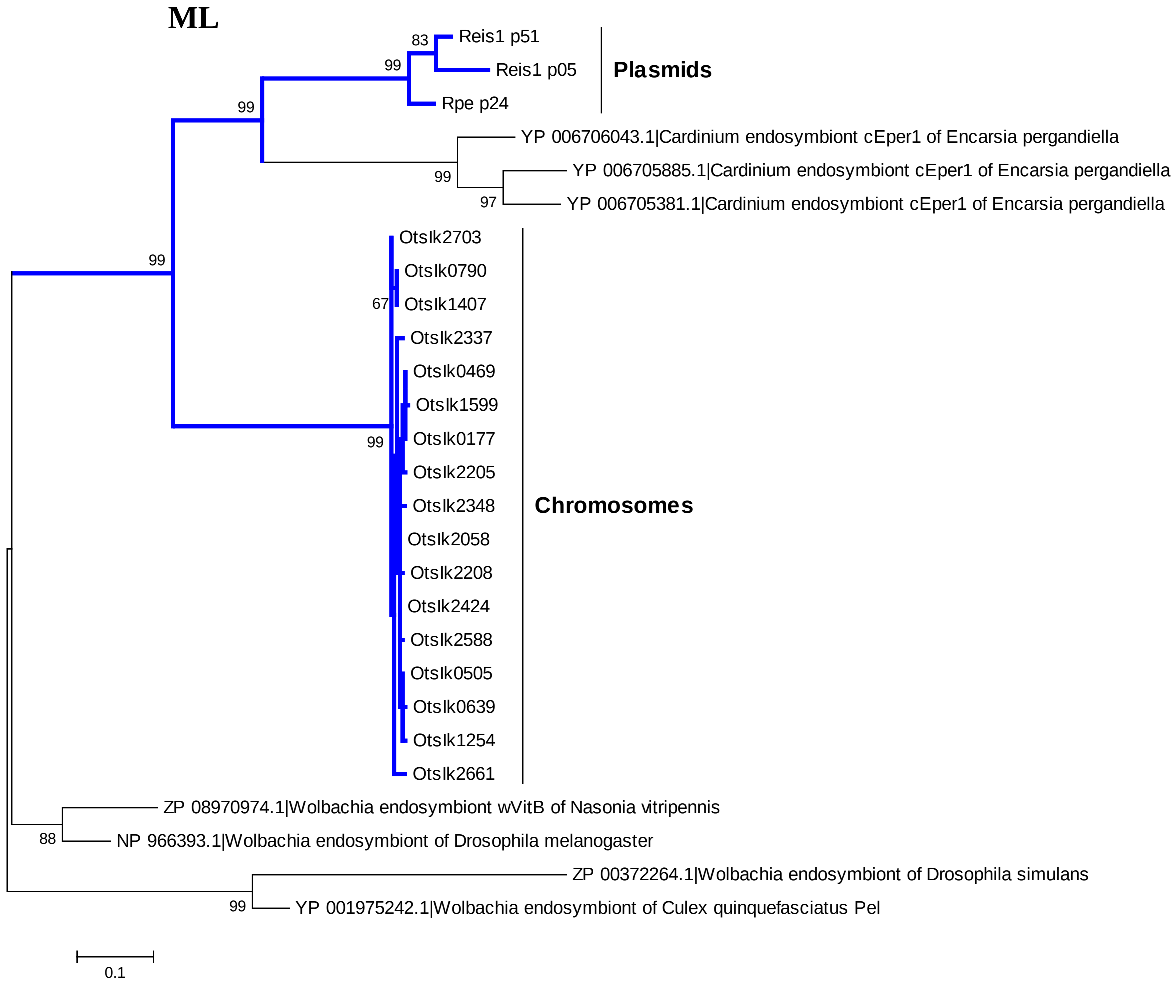
ML
Reis1 p51
Reis1 p05
Rpe p24
Plasmids
YP 006706043.1|Cardinium endosymbiont cEper1 of Encarsia pergandiella
YP 006705885.1|Cardinium endosymbiont cEper1 of Encarsia pergandiella
YP 006705381.1|Cardinium endosymbiont cEper1 of Encarsia pergandiella
OtsIk2703
OtsIk0790
OtsIk1407
OtsIk2337
OtsIk0469
OtsIk1599
OtsIk0177
OtsIk2205
OtsIk2348
OtsIk2058
OtsIk2208
OtsIk2424
OtsIk2588
OtsIk0505
OtsIk0639
OtsIk1254
OtsIk2661
Chromosomes
ZP 08970974.1|Wolbachia endosymbiont wVitB of Nasonia vitripennis
NP 966393.1|Wolbachia endosymbiont of Drosophila melanogaster
ZP 00372264.1|Wolbachia endosymbiont of Drosophila simulans
YP 001975242.1|Wolbachia endosymbiont of Culex quinquefasciatus Pel
83
99
99
99
99
97
99
67
99
88
99
0.1

Figure A11. Neighbor-joining (NJ) and maximum likelihood (ML) trees of transposase containing PDDEXK_2 domain. Bootstrap supports higher than or equal to 60% are shown on the branches.

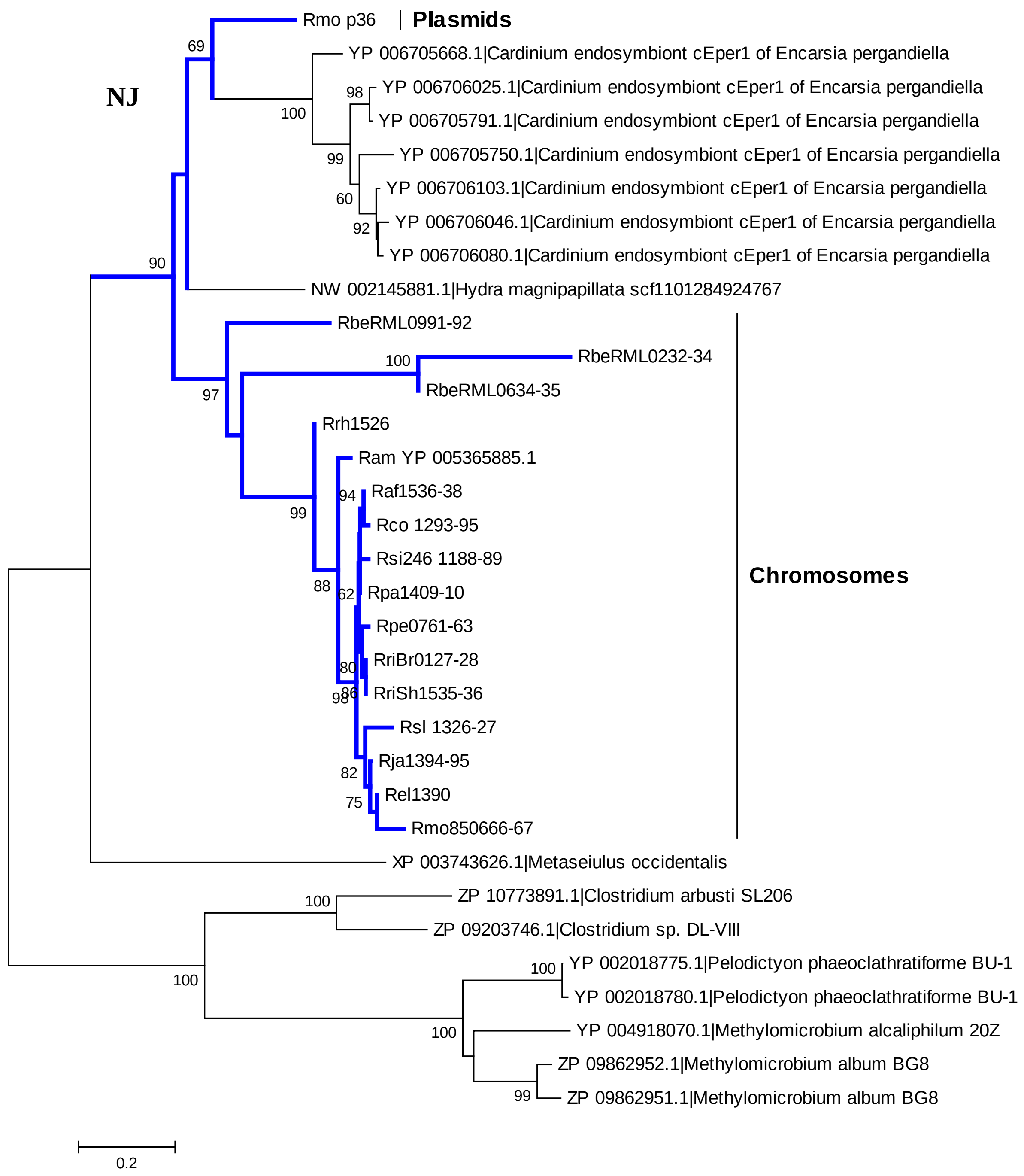

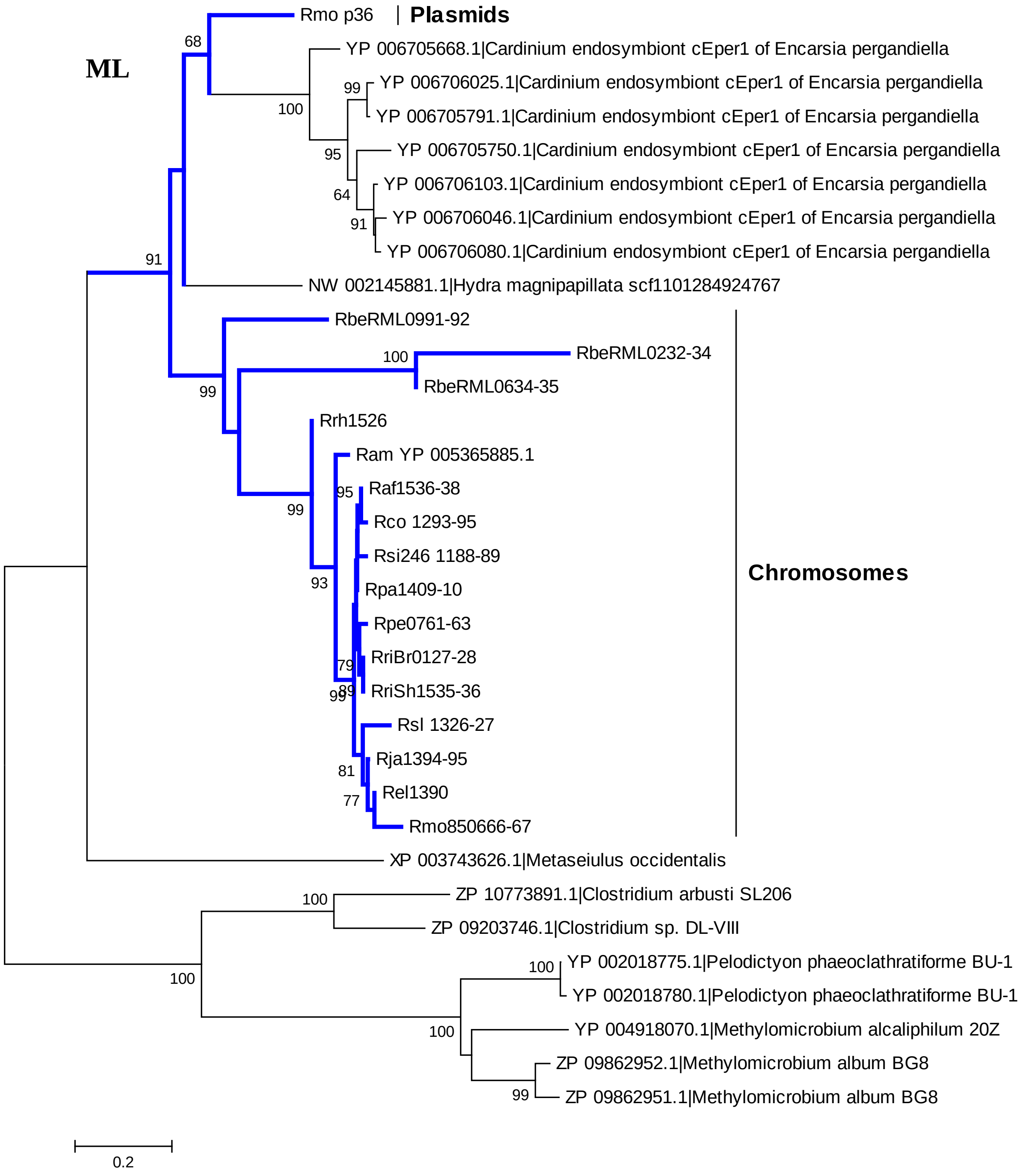

ML
Rmo p36
Plasmids
YP 006705668.1|Cardinium endosymbiont cEper1 of Encarsia pergandiella
YP 006706025.1|Cardinium endosymbiont cEper1 of Encarsia pergandiella
YP 006705791.1|Cardinium endosymbiont cEper1 of Encarsia pergandiella
YP 006705750.1|Cardinium endosymbiont cEper1 of Encarsia pergandiella
YP 006706103.1|Cardinium endosymbiont cEper1 of Encarsia pergandiella
YP 006706046.1|Cardinium endosymbiont cEper1 of Encarsia pergandiella
YP 006706080.1|Cardinium endosymbiont cEper1 of Encarsia pergandiella
NW 002145881.1|Hydra magnipapillata scf1101284924767
RbeRML0991-92
RbeRML0232-34
RbeRML0634-35
Rrh1526
Ram YP 005365885.1
Raf1536-38
Rco 1293-95
Rsi246 1188-89
Rpa1409-10
Rpe0761-63
RriBr0127-28
RriSh1535-36
Rsl 1326-27
Rja1394-95
Rel1390
Rmo850666-67
Chromosomes
XP 003743626.1|Metaseiulus occidentalis
ZP 10773891.1|Clostridium arbusti SL206
ZP 09203746.1|Clostridium sp. DL-VIII
YP 002018775.1|Pelodictyon phaeoclathratiforme BU-1
YP 002018780.1|Pelodictyon phaeoclathratiforme BU-1
YP 004918070.1|Methylomicrobium alcaliphilum 20Z
ZP 09862952.1|Methylomicrobium album BG8
ZP 09862951.1|Methylomicrobium album BG8
0.2

Figure A12. Neighbor-joining (NJ) and maximum likelihood (ML) trees of transposase/integrase containing HTH_38 and rve domains. Bootstrap supports higher than or equal to 60% are shown on the branches.

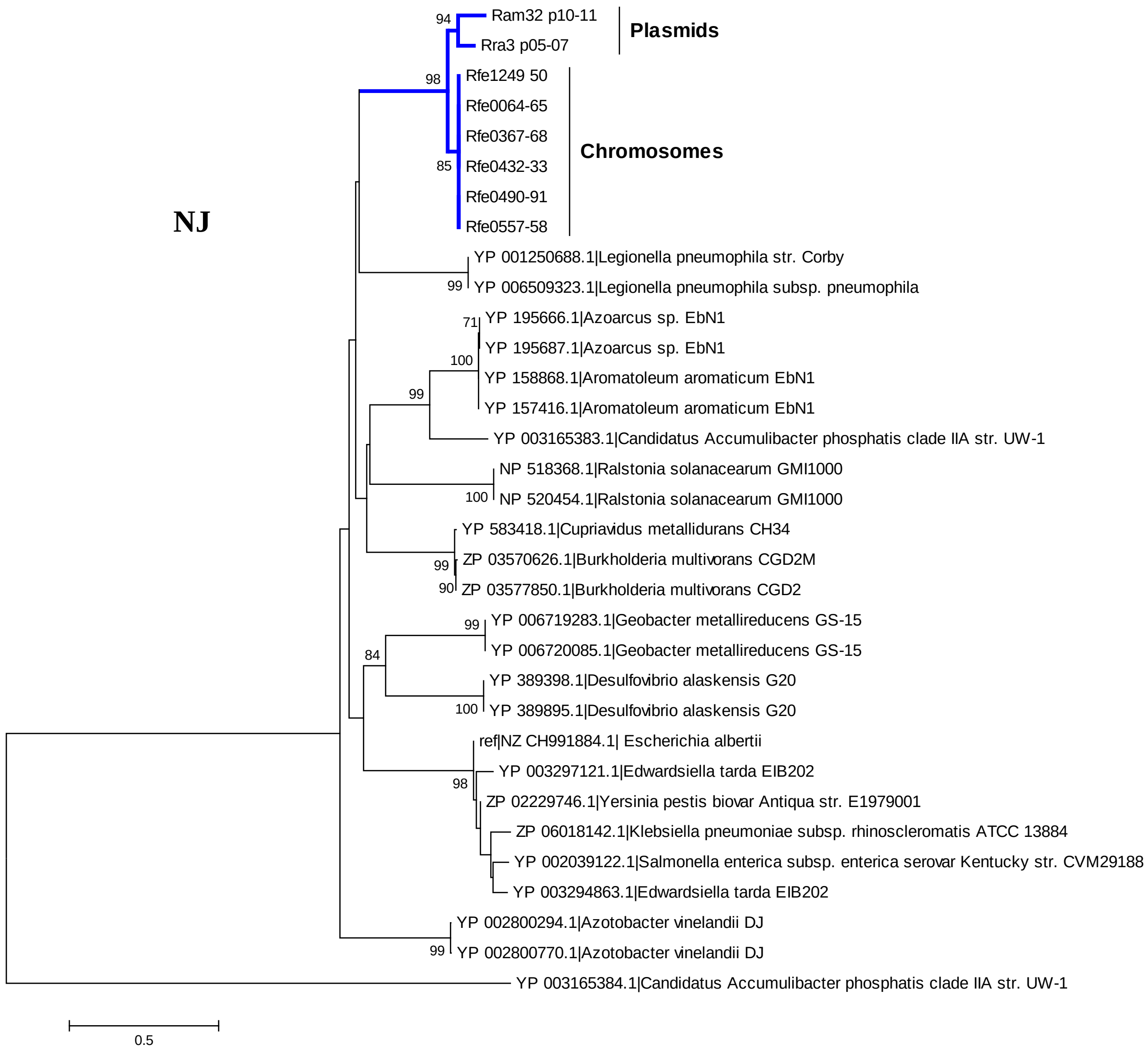

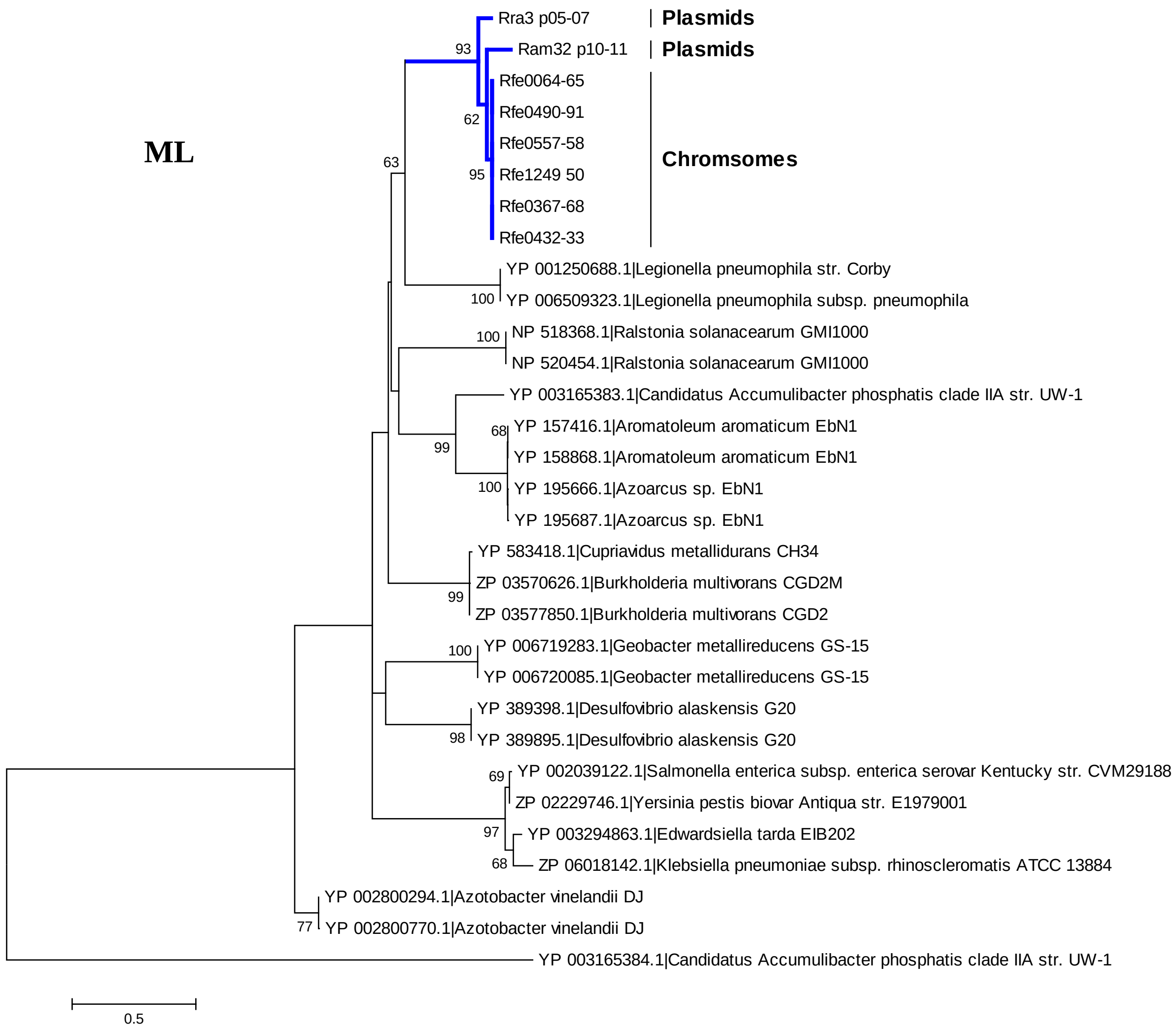
ML
Rra3 p05-07
Ram32 p10-11
Rfe0064-65
Rfe0490-91
Rfe0557-58
Rfe1249 50
Rfe0367-68
Rfe0432-33
Plasmids
Plasmids
Chromsomes
93
62
95
63
YP 001250688.1|Legionella pneumophila str. Corby
YP 006509323.1|Legionella pneumophila subsp. pneumophila
100
NP 518368.1|Ralstonia solanacearum GMI1000
NP 520454.1|Ralstonia solanacearum GMI1000
100
YP 003165383.1|Candidatus Accumulibacter phosphatis clade IIA str. UW-1
YP 157416.1|Aromatoleum aromaticum EbN1
YP 158868.1|Aromatoleum aromaticum EbN1
YP 195666.1|Azoarcus sp. EbN1
YP 195687.1|Azoarcus sp. EbN1
68
99
100
YP 583418.1|Cupriavidus metallidurans CH34
ZP 03570626.1|Burkholderia multivorans CGD2M
ZP 03577850.1|Burkholderia multivorans CGD2
99
YP 006719283.1|Geobacter metallireducens GS-15
YP 006720085.1|Geobacter metallireducens GS-15
100
YP 389398.1|Desulfovibrio alaskensis G20
YP 389895.1|Desulfovibrio alaskensis G20
98
YP 002039122.1|Salmonella enterica subsp. enterica serovar Kentucky str. CVM29188
ZP 02229746.1|Yersinia pestis biovar Antiqua str. E1979001
YP 003294863.1|Edwardsiella tarda EIB202
ZP 06018142.1|Klebsiella pneumoniae subsp. rhinoscleromatis ATCC 13884
69
97
68
YP 002800294.1|Azotobacter vinelandii DJ
YP 002800770.1|Azotobacter vinelandii DJ
77
YP 003165384.1|Candidatus Accumulibacter phosphatis clade IIA str. UW-1
0.5

Figure A13. Neighbor-joining (NJ) and maximum likelihood (ML) trees of DNA polymerase III, subunit epsilon. Bootstrap supports higher than or equal to 60% are shown on the branches.

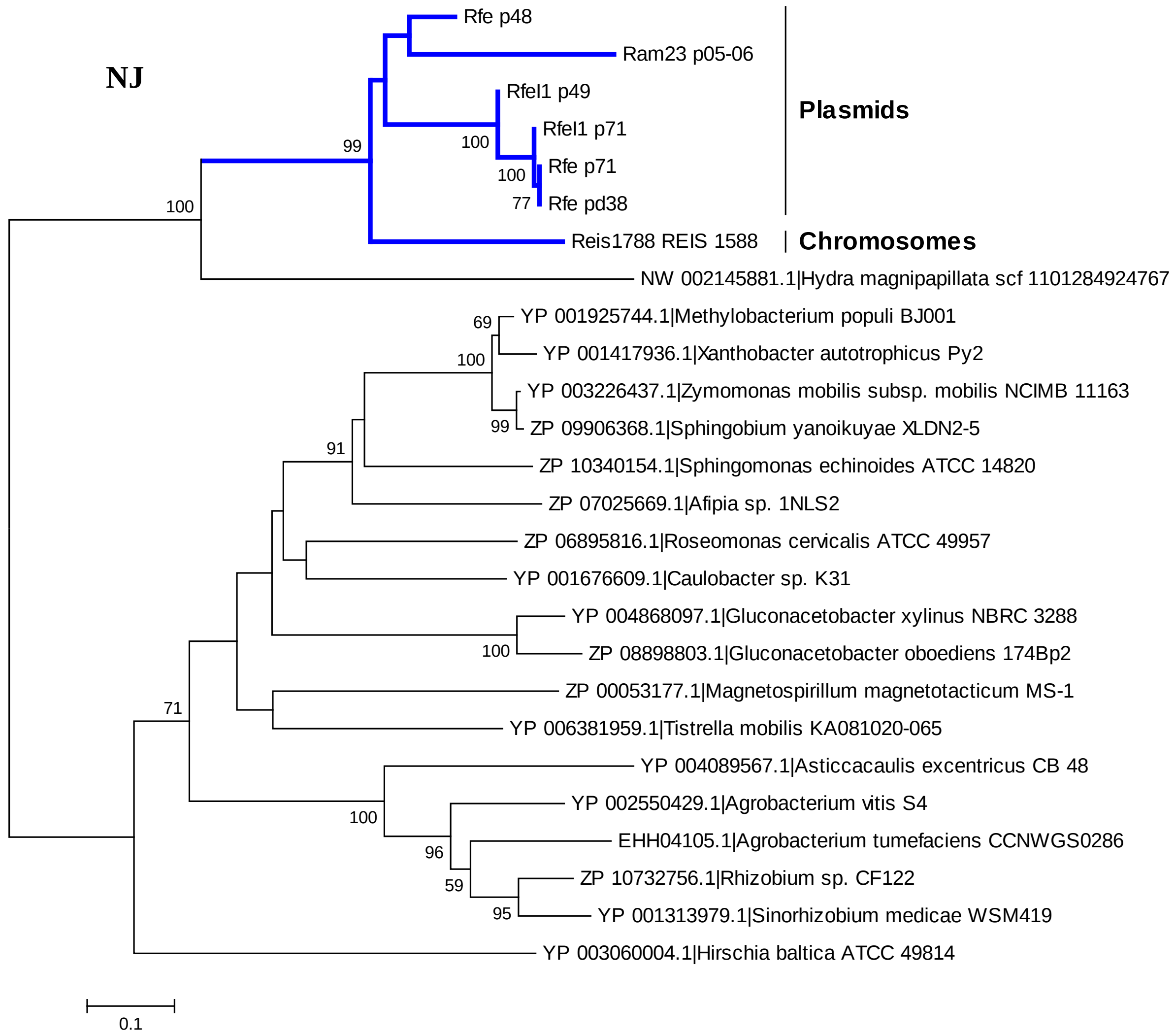

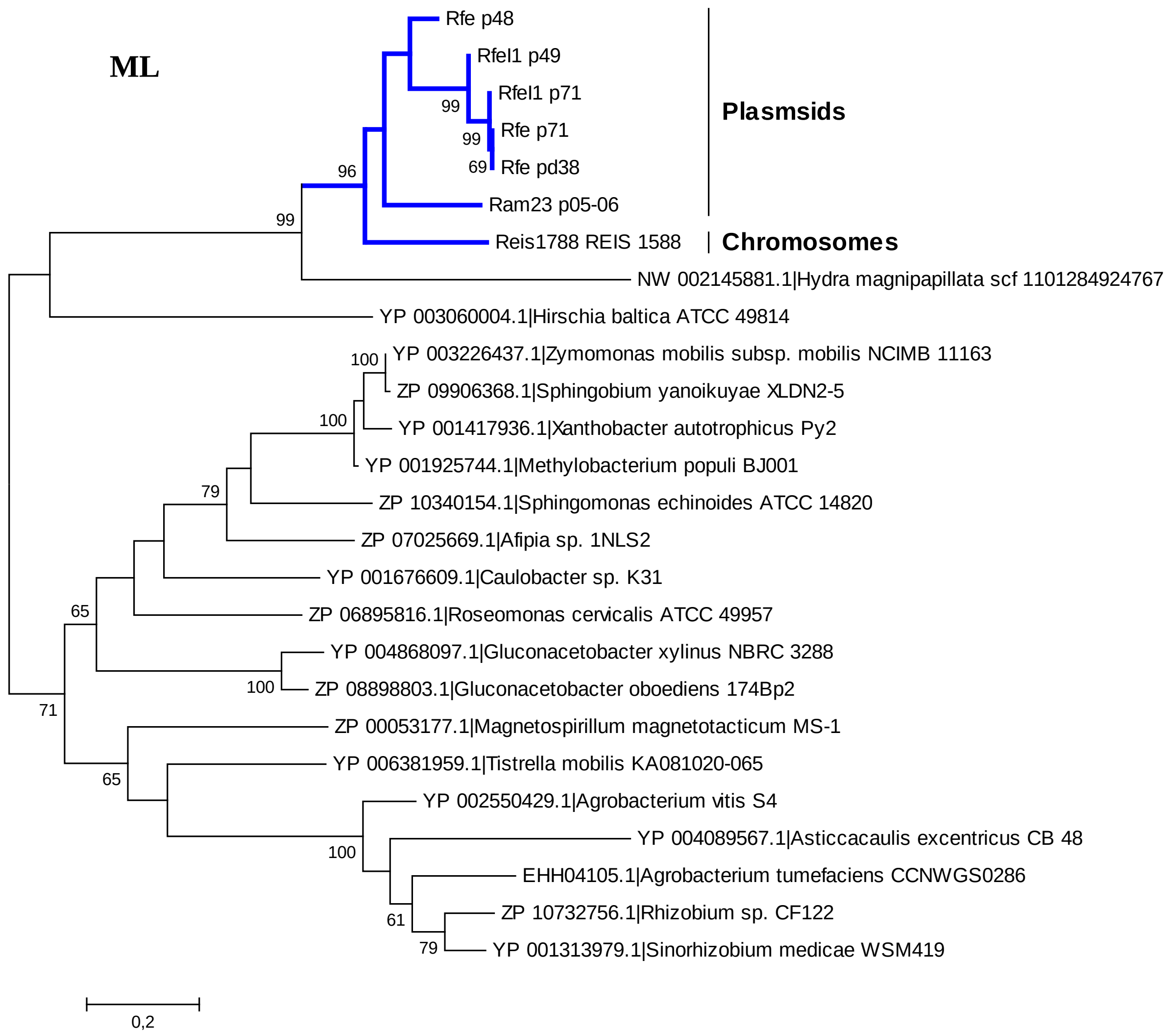
ML
Rfe p48
Rfel1 p49
Rfel1 p71
Rfe p71
Rfe pd38
Ram23 p05-06
Reis1788 REIS 1588
Plasmsids
Chromosomes
NW 002145881.1|Hydra magnipapillata scf 1101284924767
YP 003060004.1|Hirschia baltica ATCC 49814
YP 003226437.1|Zymomonas mobilis subsp. mobilis NCIMB 11163
ZP 09906368.1|Sphingobium yanoikuyae XLDN2-5
YP 001417936.1|Xanthobacter autotrophicus Py2
YP 001925744.1|Methylobacterium populi BJ001
ZP 10340154.1|Sphingomonas echinoides ATCC 14820
ZP 07025669.1|Afipia sp. 1NLS2
YP 001676609.1|Caulobacter sp. K31
ZP 06895816.1|Roseomonas cervicalis ATCC 49957
YP 004868097.1|Gluconacetobacter xylinus NBRC 3288
ZP 08898803.1|Gluconacetobacter oboediens 174Bp2
ZP 00053177.1|Magnetospirillum magnetotacticum MS-1
YP 006381959.1|Tistrella mobilis KA081020-065
YP 002550429.1|Agrobacterium vitis S4
YP 004089567.1|Asticcacaulis excentricus CB 48
EHH04105.1|Agrobacterium tumefaciens CCNWGS0286
ZP 10732756.1|Rhizobium sp. CF122
YP 001313979.1|Sinorhizobium medicae WSM419
99
99
69
96
99
100
100
79
65
100
71
65
100
61
79
0,2

Figure A14. Neighbor-joining (NJ) and maximum likelihood (ML) trees of major facilitator superfamily MFS-type transporter. Bootstrap supports higher than or equal to 60% are shown on the branches.

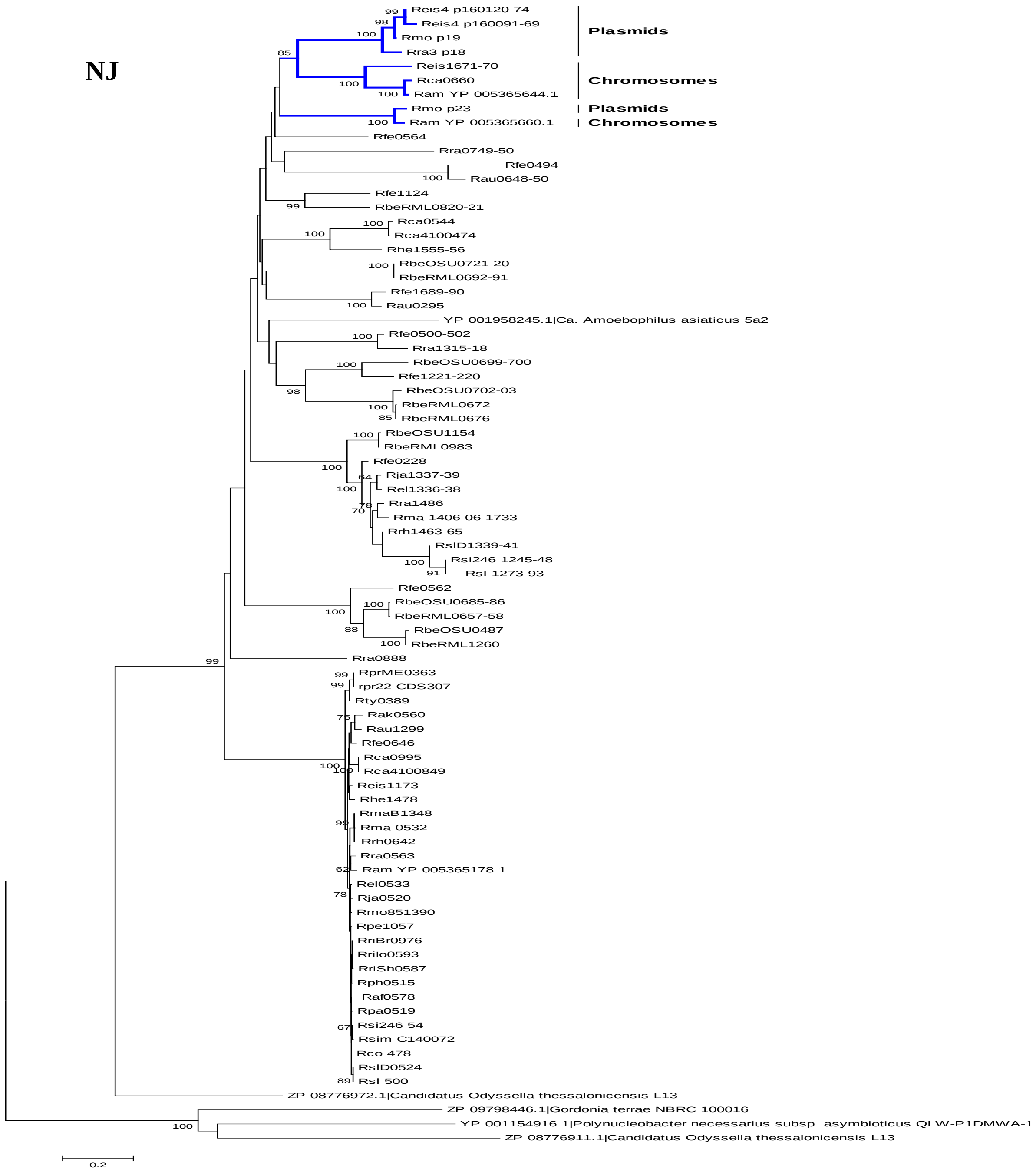

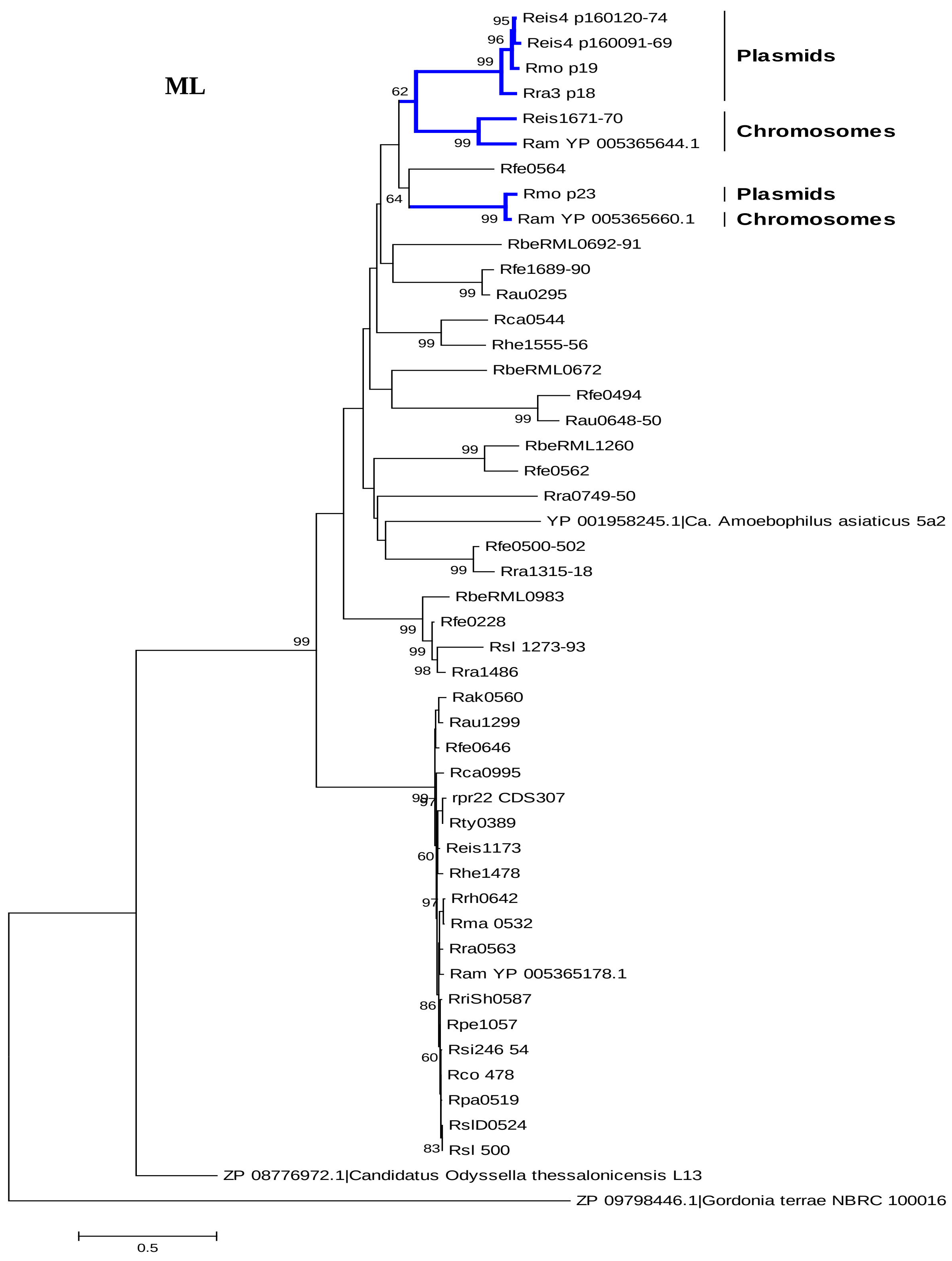
ML
Reis4 p160120-74
Reis4 p160091-69
Rmo p19
Rra3 p18
Plasmids
Reis1671-70
Ram YP 005365644.1
Chromosomes
Rfe0564
Rmo p23
Plasmids
Ram YP 005365660.1
Chromosomes
RbeRML0692-91
Rfe1689-90
Rau0295
Rca0544
Rhe1555-56
RbeRML0672
Rfe0494
Rau0648-50
RbeRML1260
Rfe0562
Rra0749-50
YP 001958245.1|Ca. Amoebophilus asiaticus 5a2
Rfe0500-502
Rra1315-18
RbeRML0983
Rfe0228
Rsl 1273-93
Rra1486
Rak0560
Rau1299
Rfe0646
Rca0995
rpr22 CDS307
Rty0389
Reis1173
Rhe1478
Rrh0642
Rma 0532
Rra0563
Ram YP 005365178.1
RriSh0587
Rpe1057
Rsi246 54
Rco 478
Rpa0519
RslD0524
Rsl 500
ZP 08776972.1|Candidatus Odyssella thessalonicensis L13
ZP 09798446.1|Gordonia terrae NBRC 100016
0.5

Figure A15. Neighbor-joining (NJ) and maximum likelihood (ML) trees of SMR-type multi-drug efflux transporter. Bootstrap supports higher than or equal to 60% are shown on the branches.

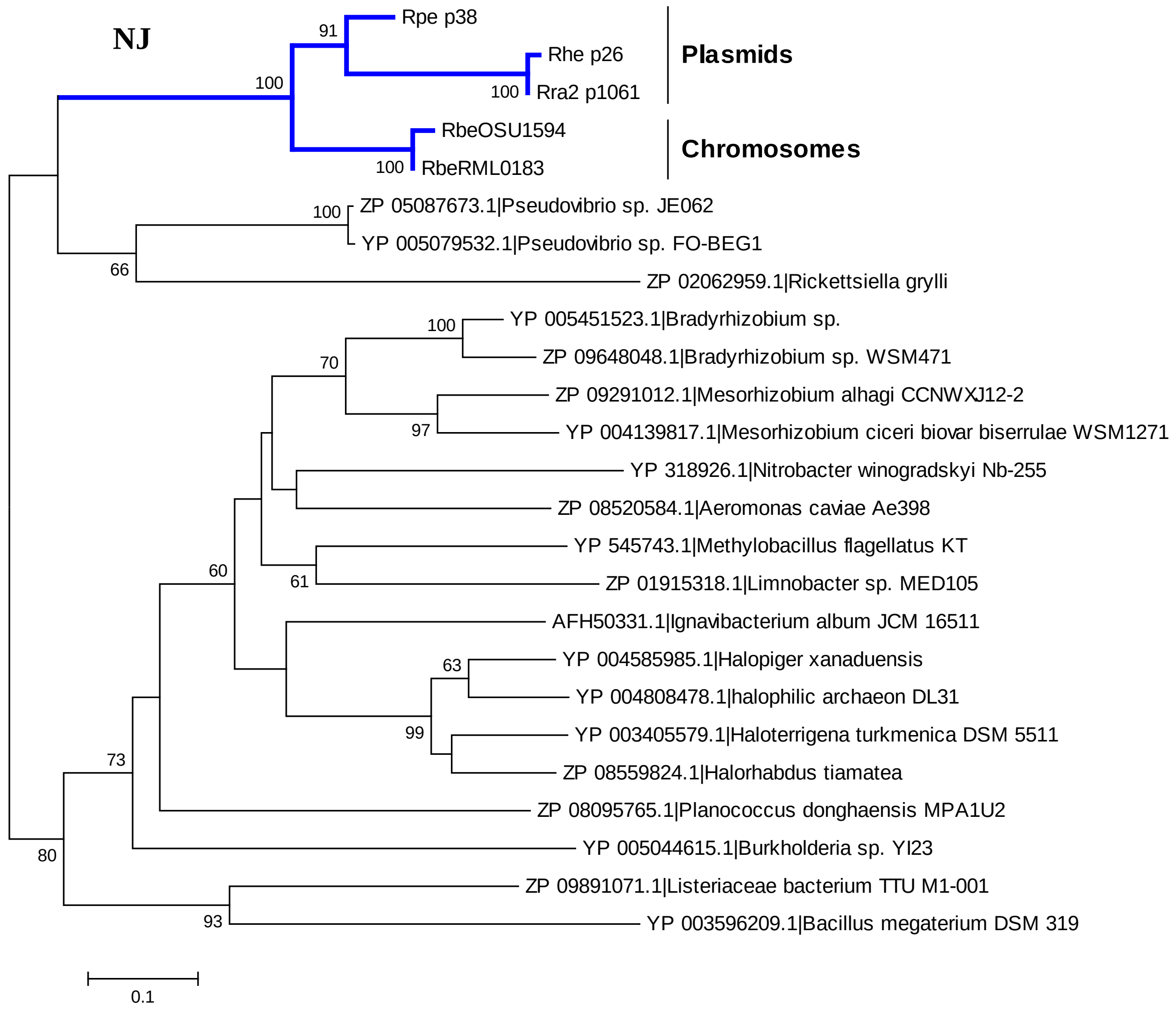

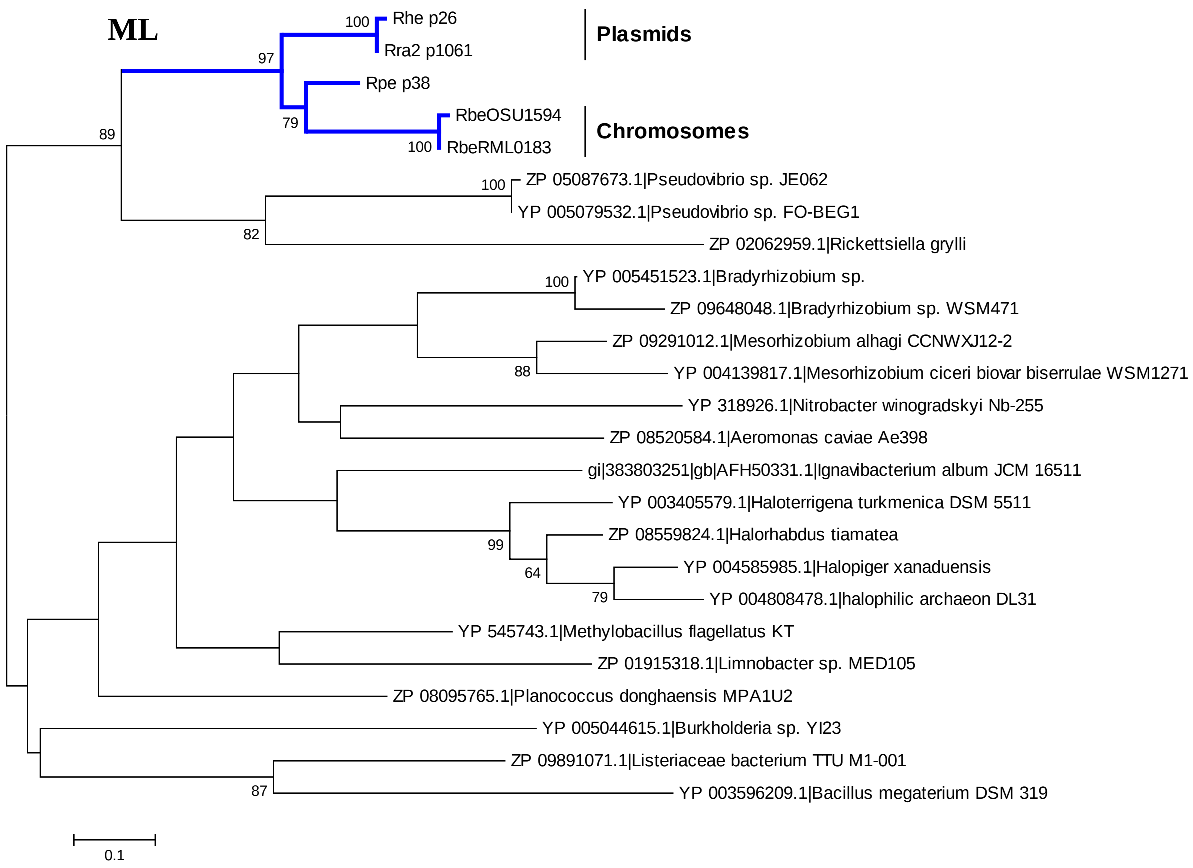

ML
Plasmids
Chromosomes
Rhe p26
Rra2 p1061
Rpe p38
RbeOSU1594
RbeRML0183
ZP 05087673.1|Pseudovibrio sp. JE062
YP 005079532.1|Pseudovibrio sp. FO-BEG1
ZP 02062959.1|Rickettsiella grylli
YP 005451523.1|Bradyrhizobium sp.
ZP 09648048.1|Bradyrhizobium sp. WSM471
ZP 09291012.1|Mesorhizobium alhagi CCNWXJ12-2
YP 004139817.1|Mesorhizobium ciceri biovar biserrulae WSM1271
YP 318926.1|Nitrobacter winogradskyi Nb-255
ZP 08520584.1|Aeromonas caviae Ae398
gi|383803251|gb|AFH50331.1|Ignavibacterium album JCM 16511
YP 003405579.1|Haloterrigena turkmenica DSM 5511
ZP 08559824.1|Halorhabdus tiamatea
YP 004585985.1|Halopiger xanaduensis
YP 004808478.1|halophilic archaeon DL31
YP 545743.1|Methylobacillus flagellatus KT
ZP 01915318.1|Limnobacter sp. MED105
ZP 08095765.1|Planococcus donghaensis MPA1U2
YP 005044615.1|Burkholderia sp. YI23
ZP 09891071.1|Listeriaceae bacterium TTU M1-001
YP 003596209.1|Bacillus megaterium DSM 319
0.1

Figure A16. Neighbor-joining (NJ) and maximum likelihood (ML) trees of type I restriction-modification system methyltransferase subunit. Bootstrap supports higher than or equal to 60% are shown on the branches.

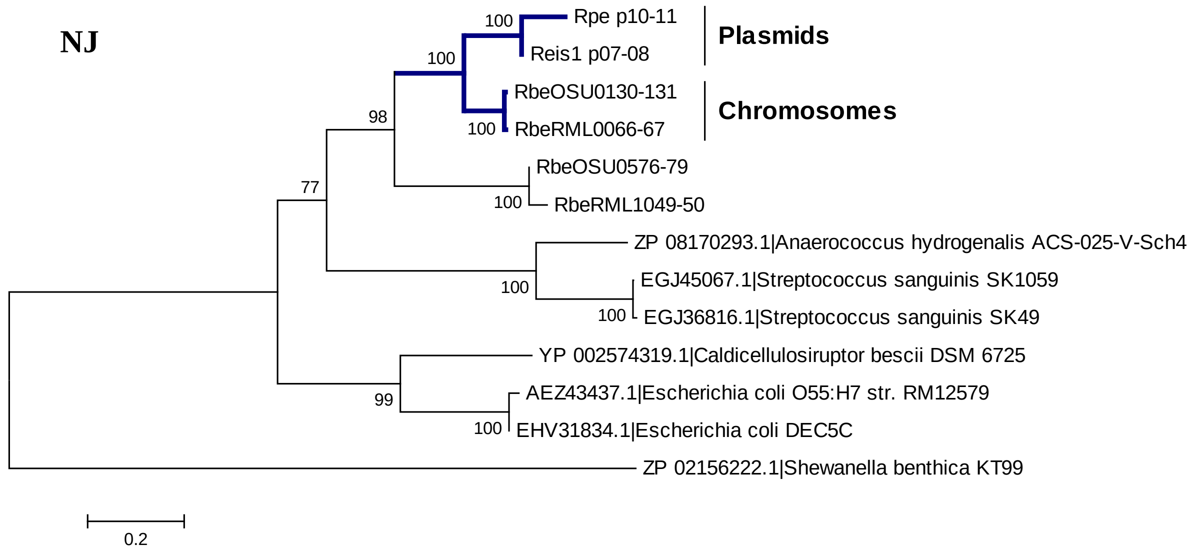

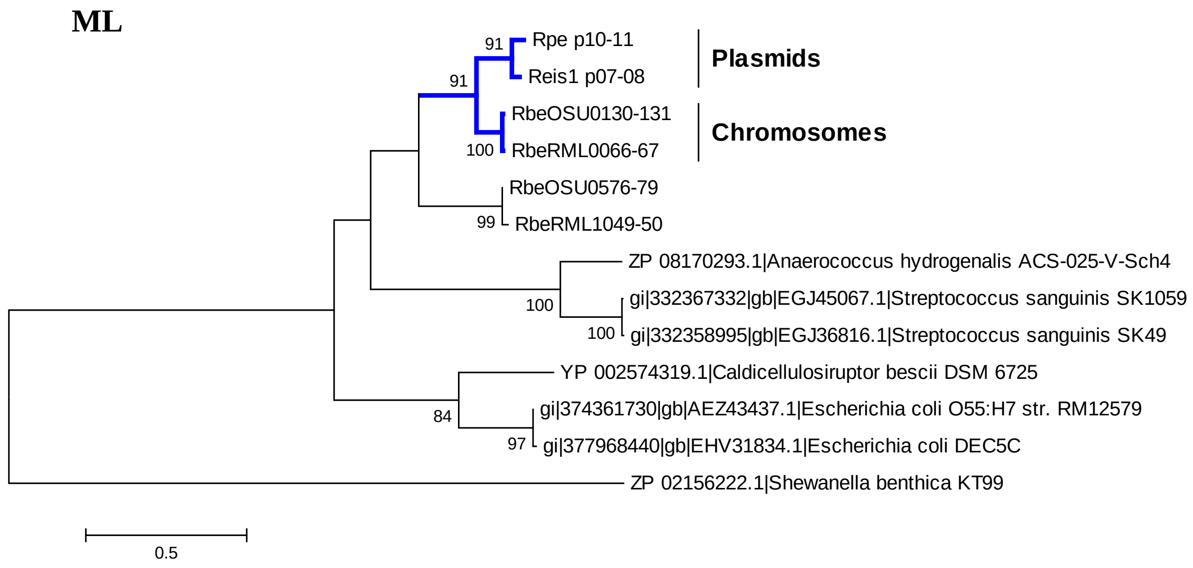
ML
Rpe p10-11
Reis1 p07-08
RbeOSU0130-131
RbeRML0066-67
RbeOSU0576-79
RbeRML1049-50
ZP 08170293.1|Anaerococcus hydrogenalis ACS-025-V-Sch4
gi|332367332|gb|EGJ45067.1|Streptococcus sanguinis SK1059
gi|332358995|gb|EGJ36816.1|Streptococcus sanguinis SK49
YP 002574319.1|Caldicellulosiruptor bescii DSM 6725
gi|374361730|gb|AEZ43437.1|Escherichia coli O55:H7 str. RM12579
gi|377968440|gb|EHV31834.1|Escherichia coli DEC5C
ZP 02156222.1|Shewanella benthica KT99
Plasmids
Chromosomes
91
91
100
99
100
100
84
97
0.5

Figure A17. Neighbor-joining (NJ) and maximum likelihood (ML) trees of peptidase family M50. Bootstrap supports higher than or equal to 60% are shown on the branches.

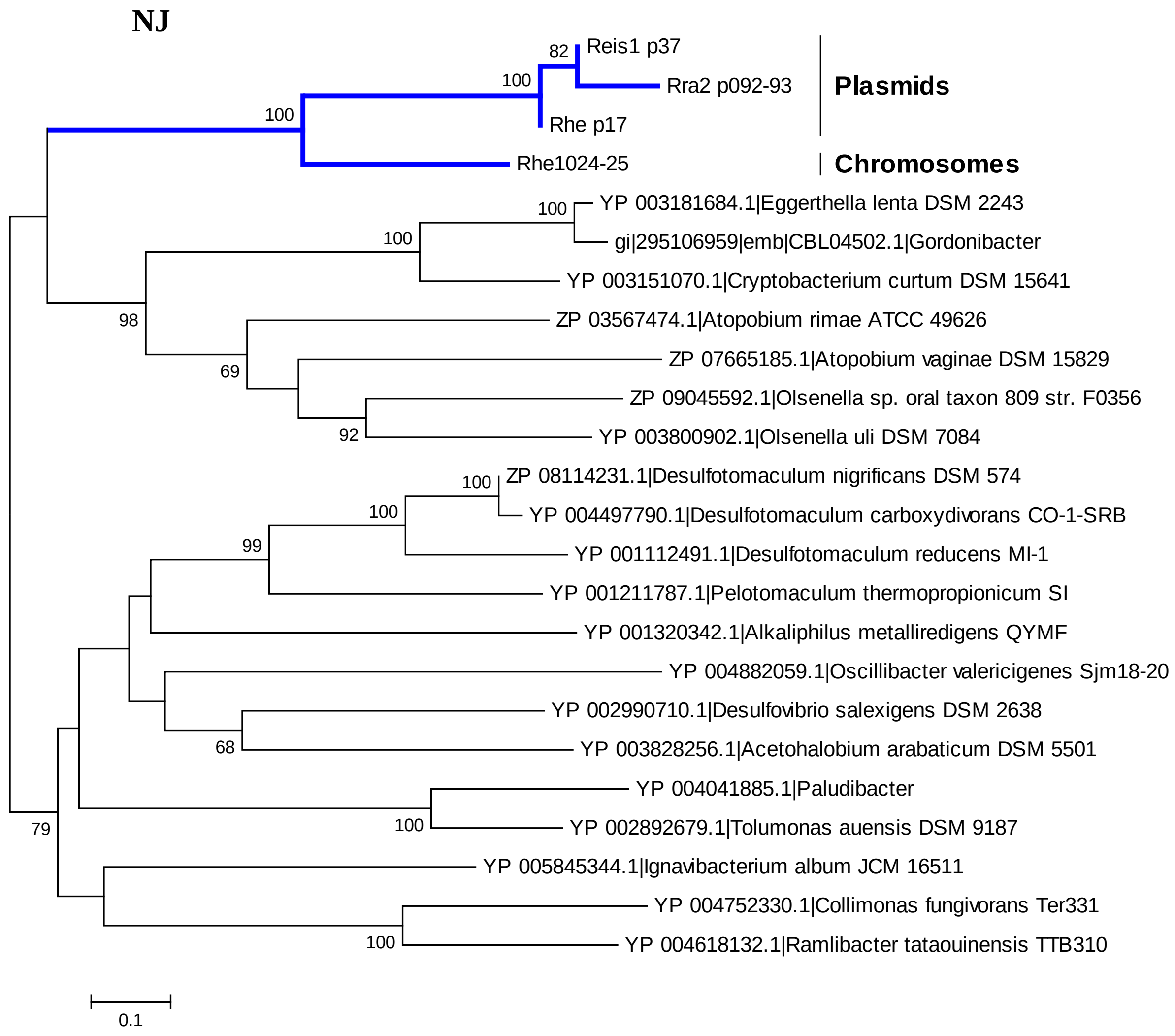

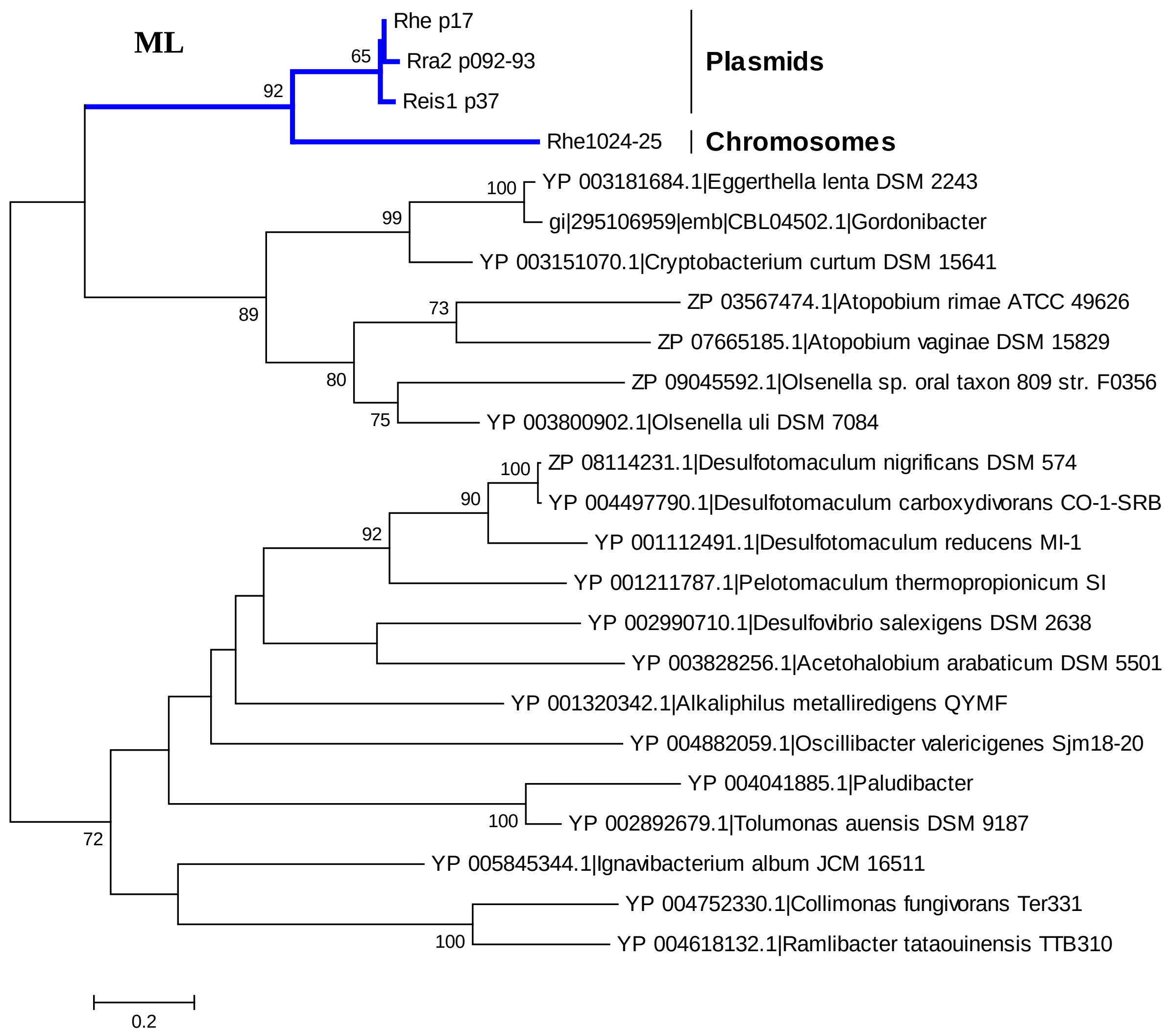
ML
Plasmids
Chromosomes
Rhe p17
Rra2 p092-93
Reis1 p37
Rhe1024-25
YP 003181684.1|Eggerthella lenta DSM 2243
gi|295106959|emb|CBL04502.1|Gordonibacter
YP 003151070.1|Cryptobacterium curtum DSM 15641
ZP 03567474.1|Atopobium rimae ATCC 49626
ZP 07665185.1|Atopobium vaginae DSM 15829
ZP 09045592.1|Olsenella sp. oral taxon 809 str. F0356
YP 003800902.1|Olsenella uli DSM 7084
ZP 08114231.1|Desulfotomaculum nigrificans DSM 574
YP 004497790.1|Desulfotomaculum carboxydivorans CO-1-SRB
YP 001112491.1|Desulfotomaculum reducens MI-1
YP 001211787.1|Pelotomaculum thermopropionicum SI
YP 002990710.1|Desulfovibrio salexigens DSM 2638
YP 003828256.1|Acetohalobium arabaticum DSM 5501
YP 001320342.1|Alkaliphilus metalliredigens QYMF
YP 004882059.1|Oscillibacter valericigenes Sjm18-20
YP 004041885.1|Paludibacter
YP 002892679.1|Tolumonas auensis DSM 9187
YP 005845344.1|Ignavibacterium album JCM 16511
YP 004752330.1|Collimonas fungivorans Ter331
YP 004618132.1|Ramlibacter tataouinensis TTB310
65
92
100
99
73
89
80
75
100
90
92
100
72
100
0.2

Figure A18. Neighbor-joining (NJ) and maximum likelihood (ML) trees of integral membrane protein. Bootstrap supports higher than or equal to 60% are shown on the branches.

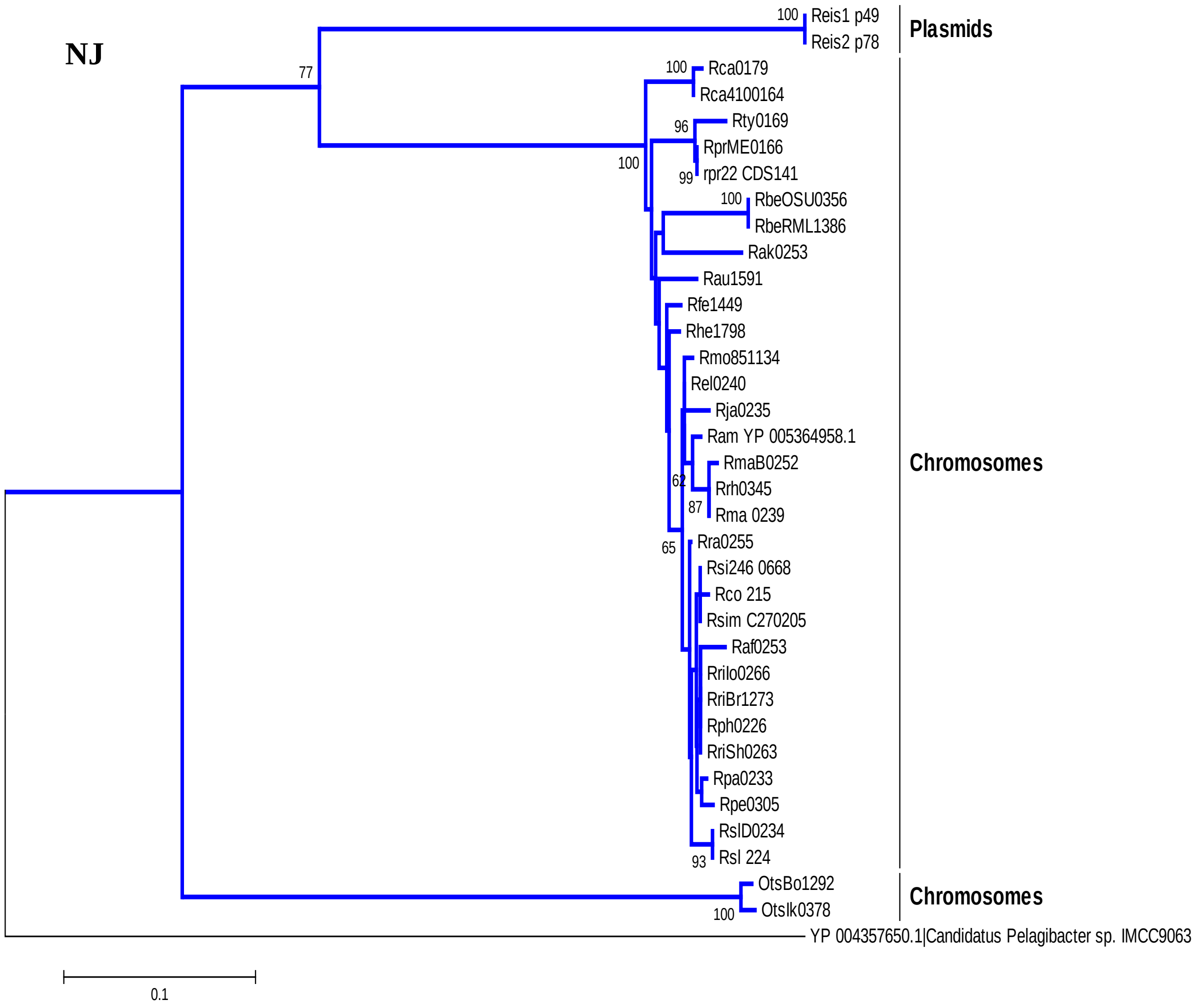

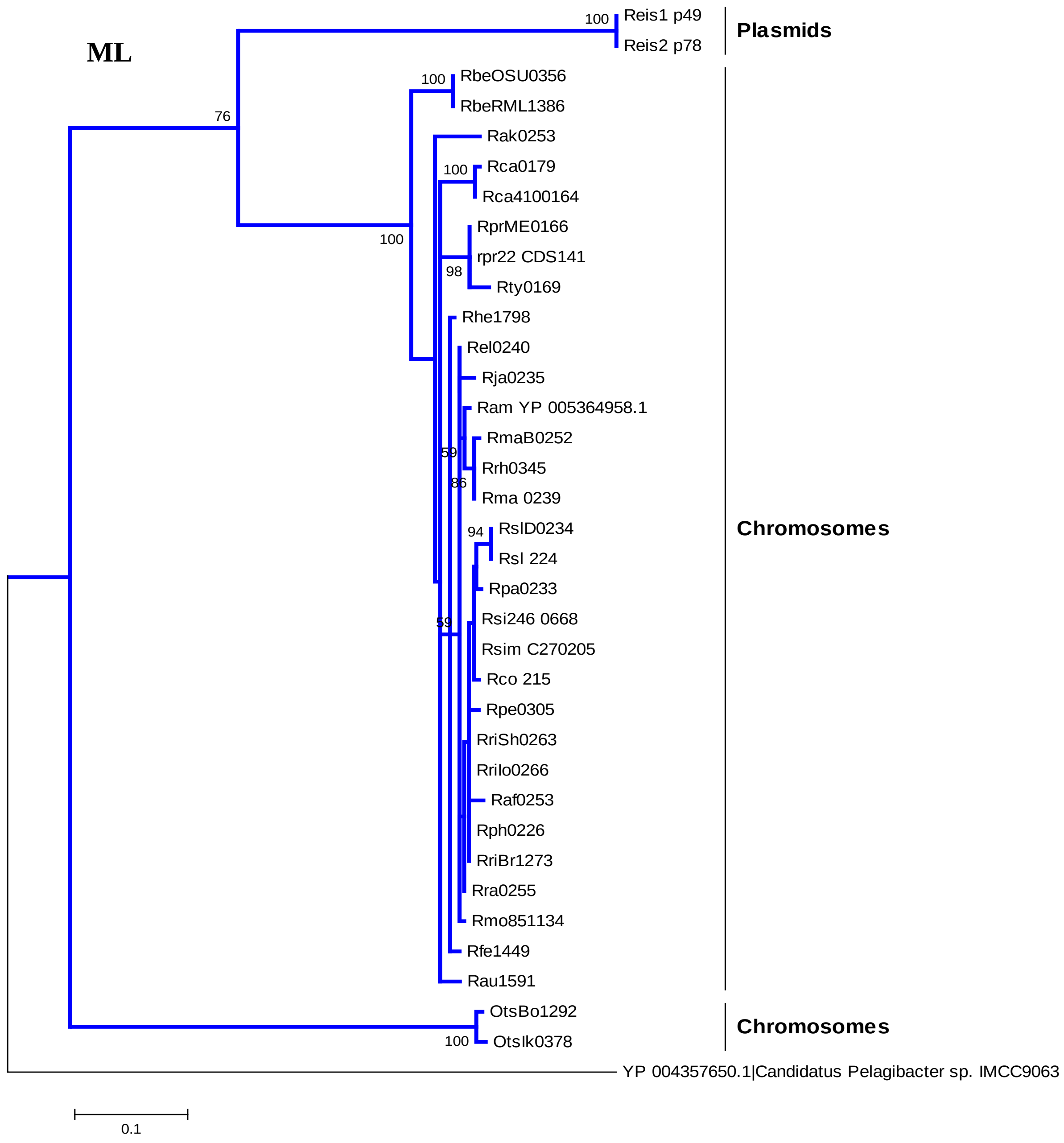
ML
100
Reis1 p49
Reis2 p78
Plasmids
76
100
RbeOSU0356
RbeRML1386
Rak0253
100
Rca0179
Rca4100164
100
RprME0166
rpr22 CDS141
98
Rty0169
Rhe1798
Rel0240
Rja0235
Ram YP 005364958.1
RmaB0252
59
Rrh0345
86
Rma 0239
94
RslD0234
Rsl 224
Rpa0233
59
Rsi246 0668
Rsim C270205
Rco 215
Rpe0305
RriSh0263
Rrilo0266
Raf0253
Rph0226
RriBr1273
Rra0255
Rmo851134
Rfe1449
Rau1591
Chromosomes
OtsBo1292
100
OtsIk0378
Chromosomes
YP 004357650.1|Candidatus Pelagibacter sp. IMCC9063
0.1

Figure A19. Neighbor-joining (NJ) and maximum likelihood (ML) trees of DNA adenine methylase domain protein. Bootstrap supports higher than or equal to 60% are shown on the branches.

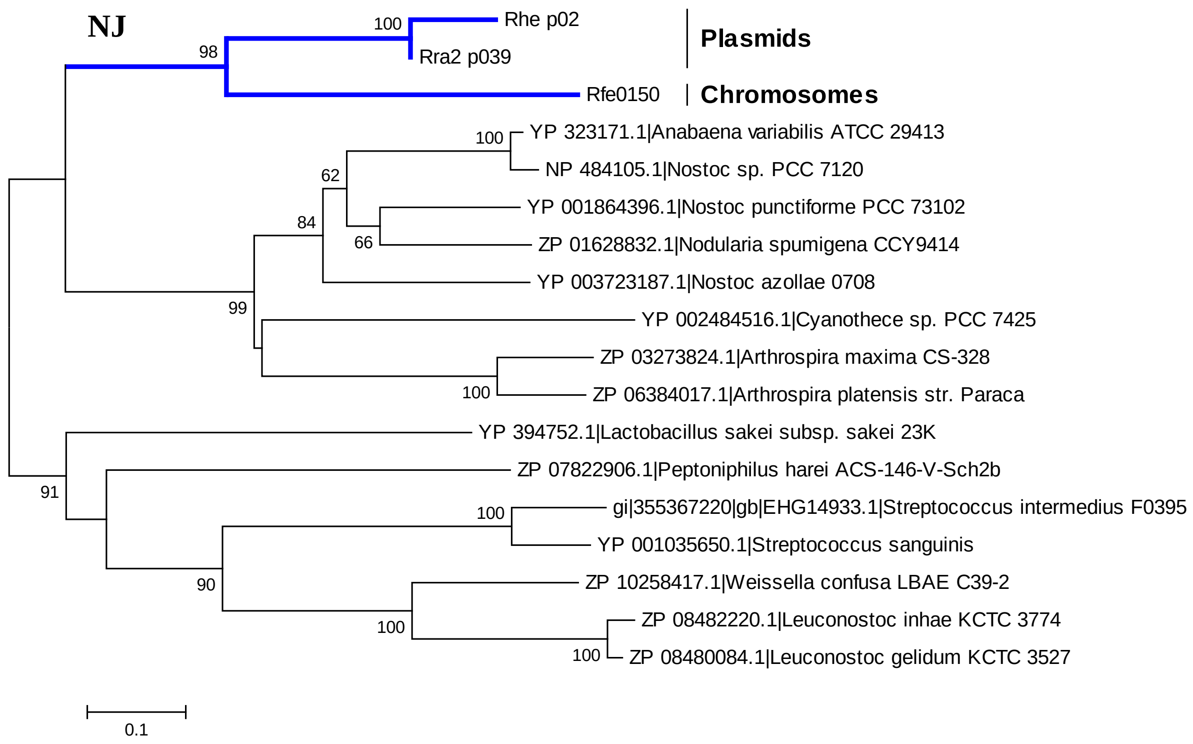

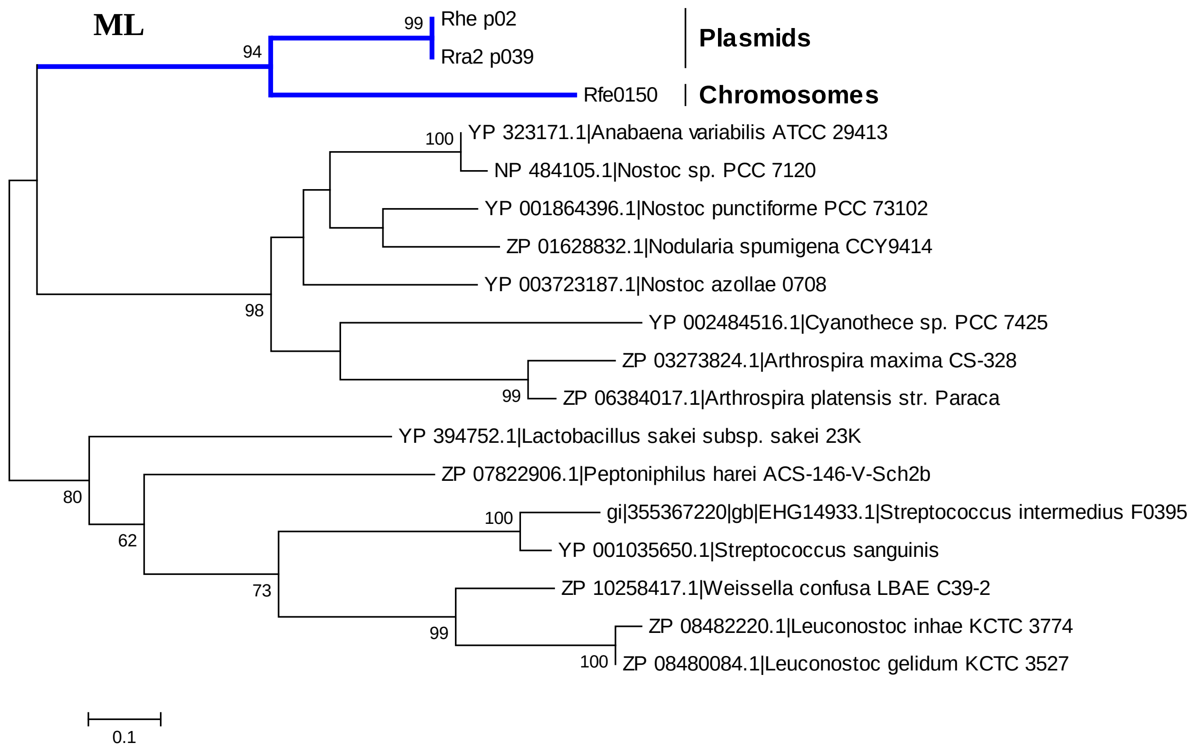
ML
99
Rhe p02
Rra2 p039
Plasmids
94
Rfe0150
Chromosomes
100
YP 323171.1|Anabaena variabilis ATCC 29413
NP 484105.1|Nostoc sp. PCC 7120
YP 001864396.1|Nostoc punctiforme PCC 73102
ZP 01628832.1|Nodularia spumigena CCY9414
YP 003723187.1|Nostoc azollae 0708
98
YP 002484516.1|Cyanothece sp. PCC 7425
ZP 03273824.1|Arthrospira maxima CS-328
99
ZP 06384017.1|Arthrospira platensis str. Paraca
YP 394752.1|Lactobacillus sakei subsp. sakei 23K
ZP 07822906.1|Peptoniphilus harei ACS-146-V-Sch2b
80
100
gi|355367220|gb|EHG14933.1|Streptococcus intermedius F0395
62
YP 001035650.1|Streptococcus sanguinis
73
ZP 10258417.1|Weissella confusa LBAE C39-2
99
ZP 08482220.1|Leuconostoc inhae KCTC 3774
100
ZP 08480084.1|Leuconostoc gelidum KCTC 3527
0.1

Figure A20. Neighbor-joining (NJ) and maximum likelihood (ML) trees of tryptophan-rich sensory protein, TspO_MBR superfamily protein. Bootstrap supports higher than or equal to 60% are shown on the branches.

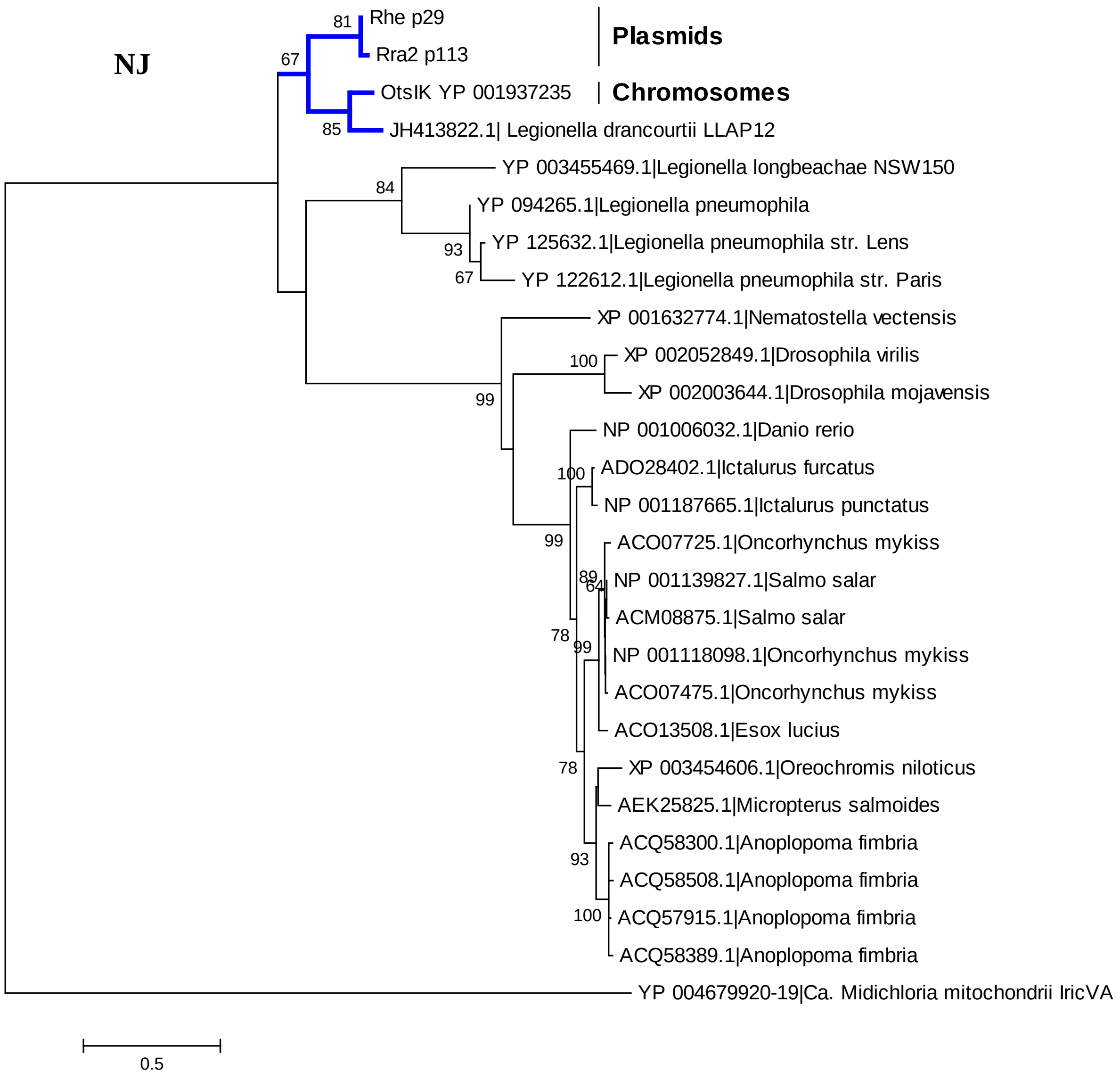

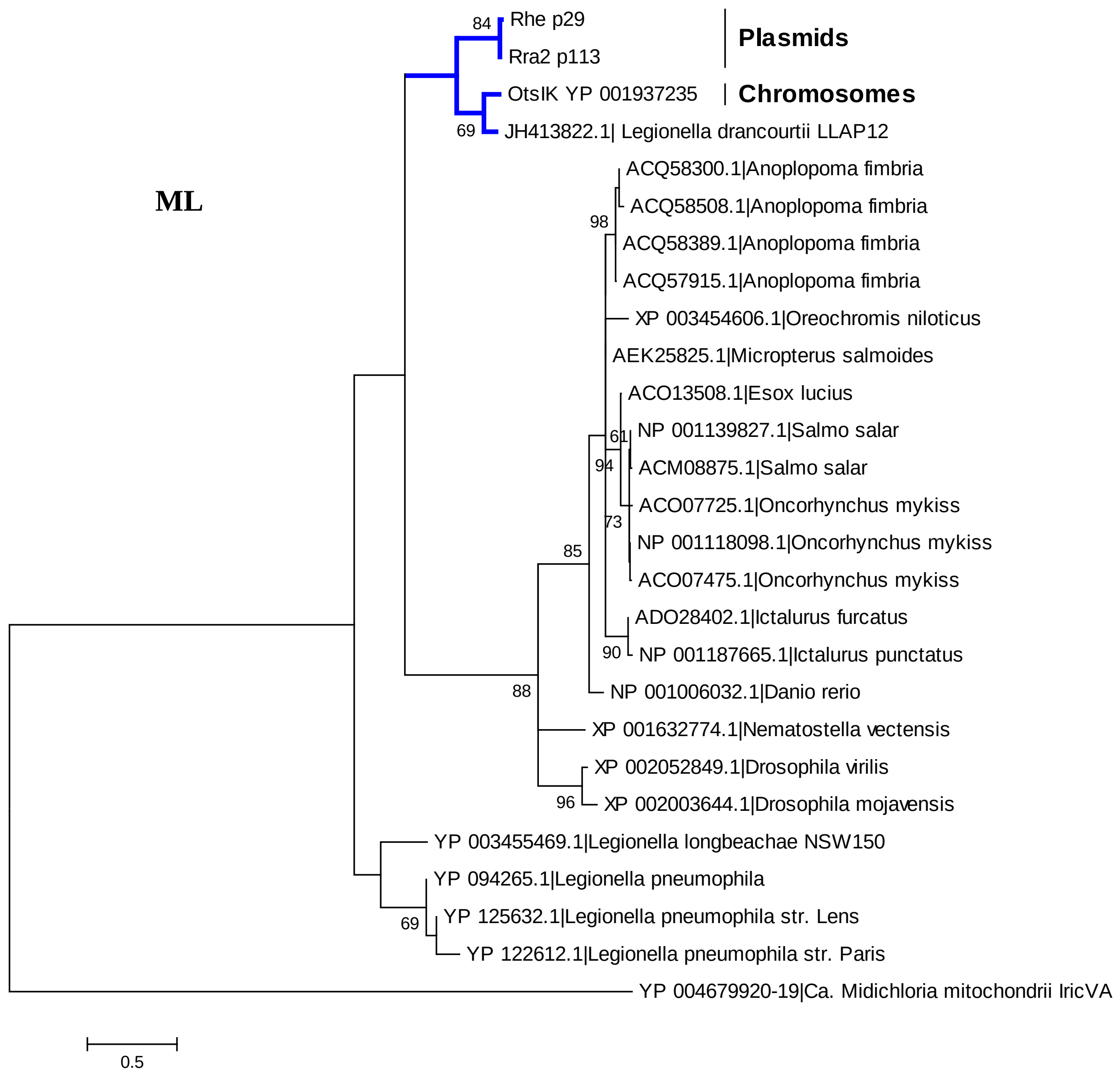
ML
Plasmids
Chromosomes
84
Rhe p29
Rra2 p113
OtsIK YP 001937235
69
JH413822.1| Legionella drancourtii LLAP12
ACQ58300.1|Anoplopoma fimbria
ACQ58508.1|Anoplopoma fimbria
98
ACQ58389.1|Anoplopoma fimbria
ACQ57915.1|Anoplopoma fimbria
XP 003454606.1|Oreochromis niloticus
AEK25825.1|Micropterus salmoides
ACO13508.1|Esox lucius
61
NP 001139827.1|Salmo salar
94
ACM08875.1|Salmo salar
ACO07725.1|Oncorhynchus mykiss
73
NP 001118098.1|Oncorhynchus mykiss
85
ACO07475.1|Oncorhynchus mykiss
ADO28402.1|Ictalurus furcatus
90
NP 001187665.1|Ictalurus punctatus
88
NP 001006032.1|Danio rerio
XP 001632774.1|Nematostella vectensis
XP 002052849.1|Drosophila virilis
96
XP 002003644.1|Drosophila mojavensis
YP 003455469.1|Legionella longbeachae NSW150
YP 094265.1|Legionella pneumophila
69
YP 125632.1|Legionella pneumophila str. Lens
YP 122612.1|Legionella pneumophila str. Paris
YP 004679920-19|Ca. Midichloria mitochondrii IricVA
0.5

Figure A21. Neighbor-joining (NJ) and maximum likelihood (ML) trees of conserved protein of unknown function. Bootstrap supports higher than or equal to 60% are shown on the branches.

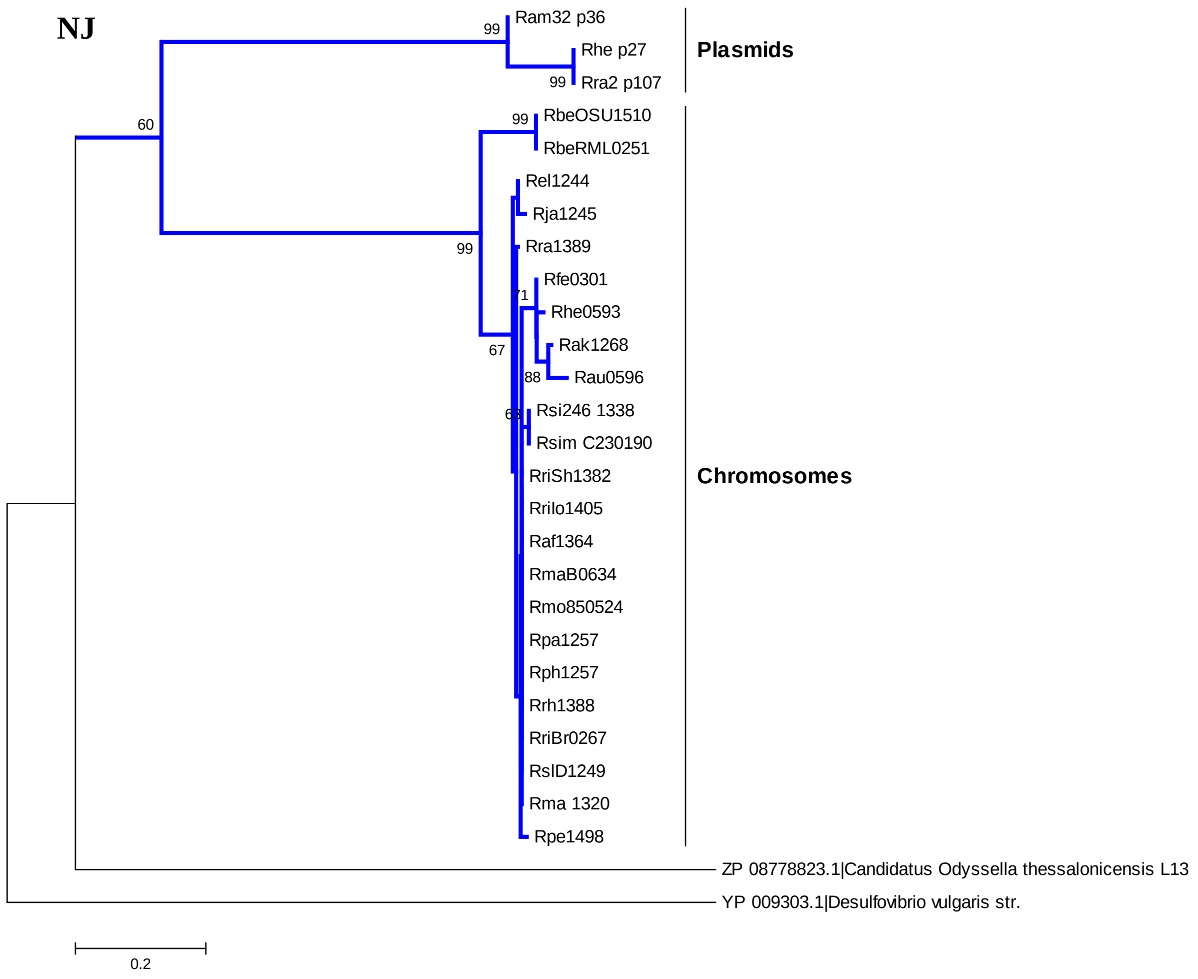

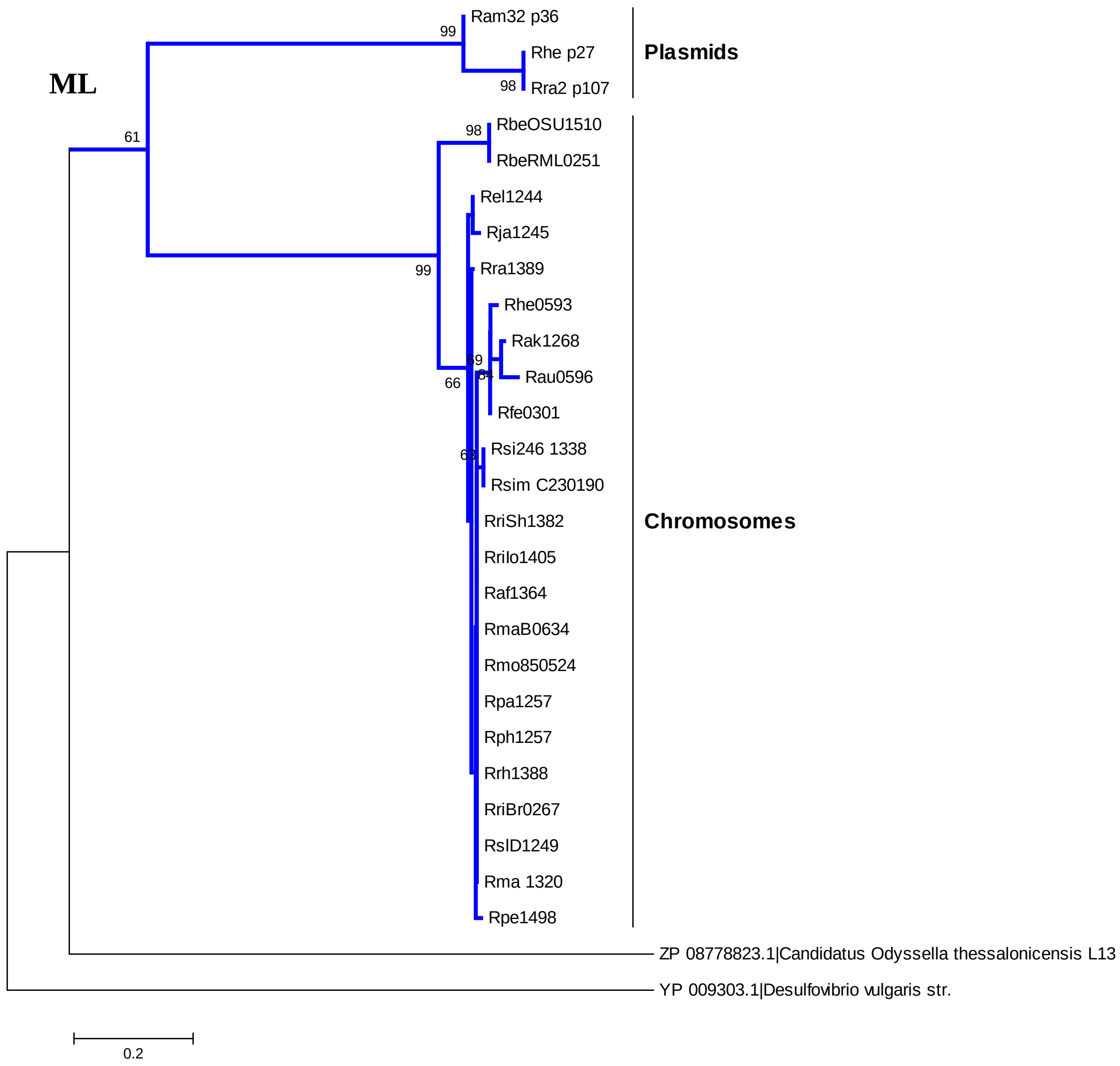
ML
Ram32 p36
Rhe p27
Rra2 p107
Plasmids
RbeOSU1510
RbeRML0251
Rel1244
Rja1245
Rra1389
Rhe0593
Rak1268
Rau0596
Rfe0301
Rsi246 1338
Rsim C230190
RriSh1382
Rrilo1405
Raf1364
RmaB0634
Rmo850524
Rpa1257
Rph1257
Rrh1388
RriBr0267
RslD1249
Rma 1320
Rpe1498
Chromosomes
ZP 08778823.1|Candidatus Odyssella thessalonicensis L13
YP 009303.1|Desulfovibrio vulgaris str.
99
98
61
98
99
66
69
84
0.2

Figure A22. Neighbor-joining (NJ) and maximum likelihood (ML) trees of transposase/IS protein containing AAA domain. Bootstrap supports higher than or equal to 60% are shown on the branches.

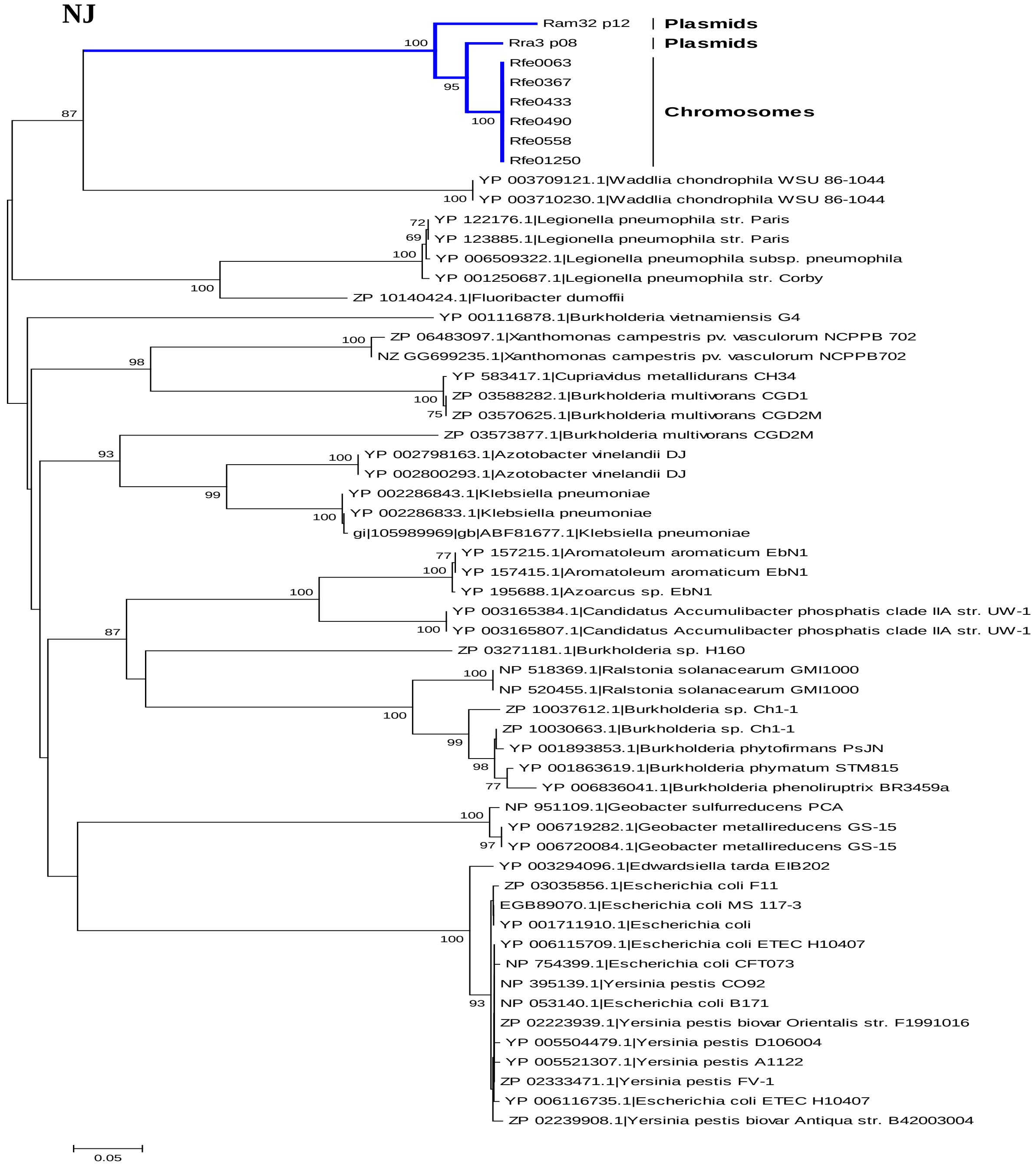

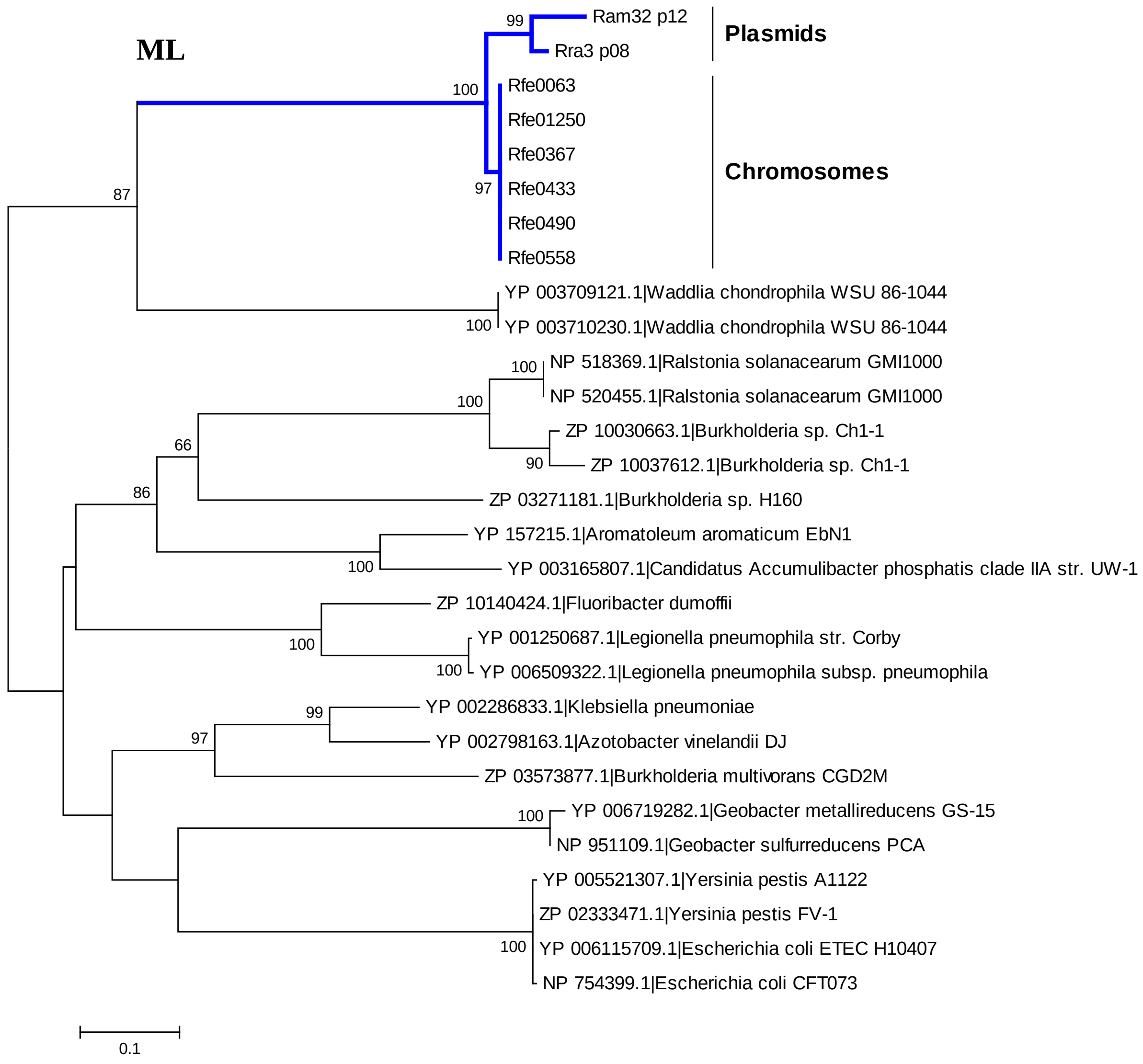

ML
Plasmids
Chromosomes
Ram32 p12
Rra3 p08
Rfe0063
Rfe01250
Rfe0367
Rfe0433
Rfe0490
Rfe0558
YP 003709121.1|Waddlia chondrophila WSU 86-1044
YP 003710230.1|Waddlia chondrophila WSU 86-1044
NP 518369.1|Ralstonia solanacearum GMI1000
NP 520455.1|Ralstonia solanacearum GMI1000
ZP 10030663.1|Burkholderia sp. Ch1-1
ZP 10037612.1|Burkholderia sp. Ch1-1
ZP 03271181.1|Burkholderia sp. H160
YP 157215.1|Aromatoleum aromaticum EbN1
YP 003165807.1|Candidatus Accumulibacter phosphatis clade IIA str. UW-1
ZP 10140424.1|Fluoribacter dumoffii
YP 001250687.1|Legionella pneumophila str. Corby
YP 006509322.1|Legionella pneumophila subsp. pneumophila
YP 002286833.1|Klebsiella pneumoniae
YP 002798163.1|Azotobacter vinelandii DJ
ZP 03573877.1|Burkholderia multivorans CGD2M
YP 006719282.1|Geobacter metallireducens GS-15
NP 951109.1|Geobacter sulfurreducens PCA
YP 005521307.1|Yersinia pestis A1122
ZP 02333471.1|Yersinia pestis FV-1
YP 006115709.1|Escherichia coli ETEC H10407
NP 754399.1|Escherichia coli CFT073
99
100
97
87
100
100
100
90
66
86
100
100
100
99
97
100
100
0.1

Figure A23. Neighbor-joining (NJ) and maximum likelihood (ML) trees of conserved protein Of unknown function. Bootstrap supports higher than or equal to 60% are shown on the branches.

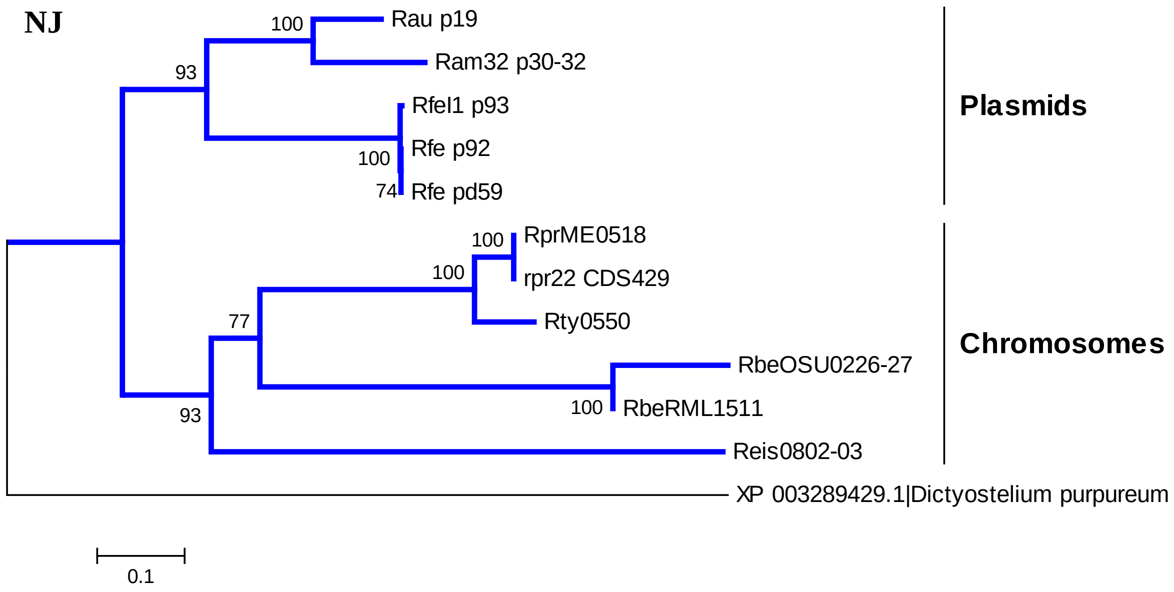

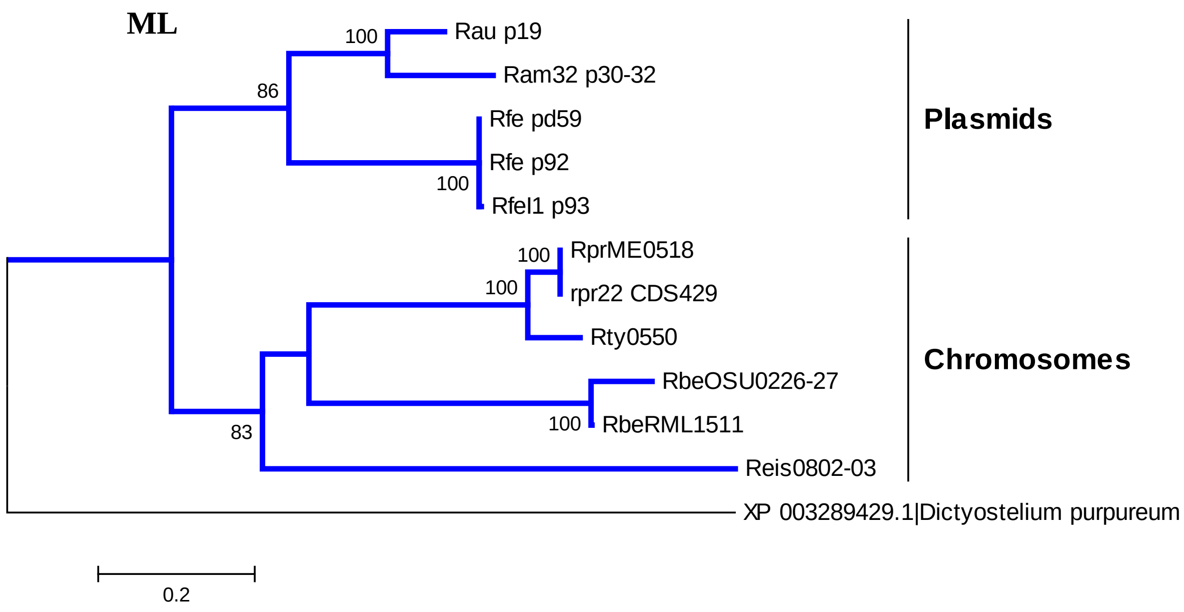
ML
100
Rau p19
86
Ram32 p30-32
Rfe pd59
Rfe p92
100
Rfel1 p93
Plasmids
100
RprME0518
100
rpr22 CDS429
Rty0550
RbeOSU0226-27
83
100
RbeRML1511
Reis0802-03
Chromosomes
XP 003289429.1|Dictyostelium purpureum
0.2

Figure A24. Neighbor-joining (NJ) and maximum likelihood (ML) trees of conserved protein Of unknown function. Bootstrap supports higher than or equal to 60% are shown on the branches.

NJ

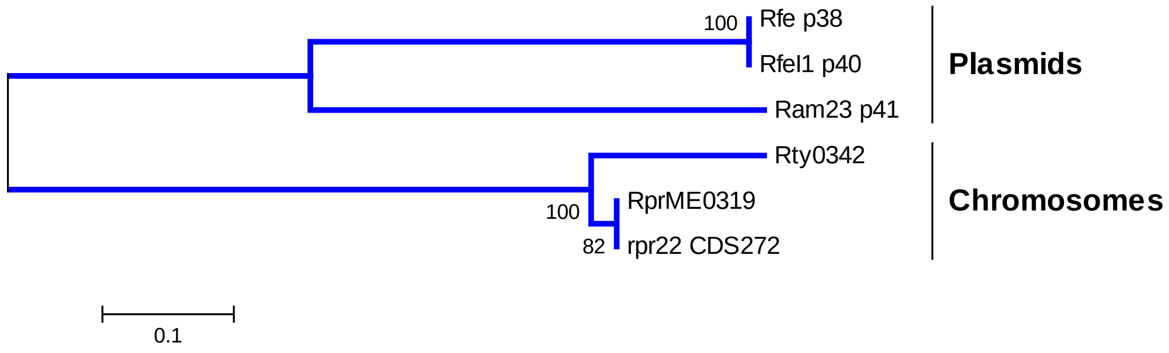

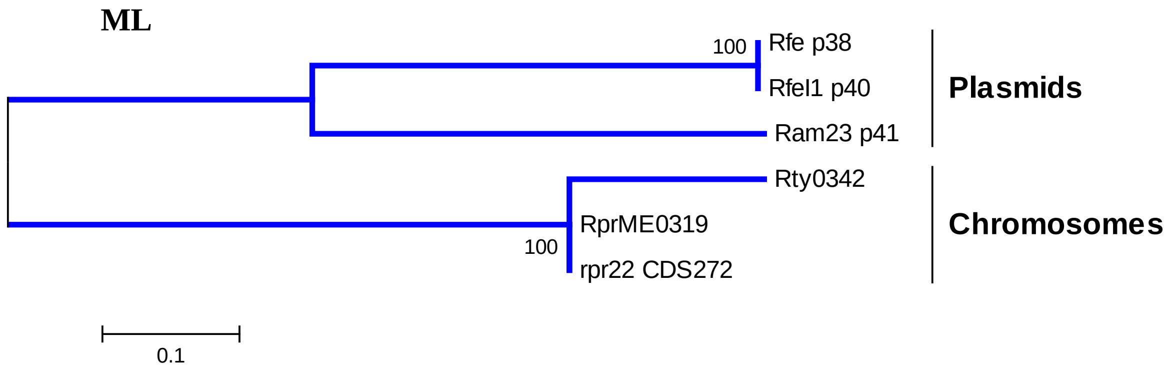
ML
100
Rfe p38
Rfel1 p40
Ram23 p41
Rty0342
RprME0319
100
rpr22 CDS272
Plasmids
Chromosomes
0.1

Figure A25. Neighbor-joining (NJ) and maximum likelihood (ML) trees of conserved protein Of unknown function. Bootstrap supports higher than or equal to 60% are shown on the branches.

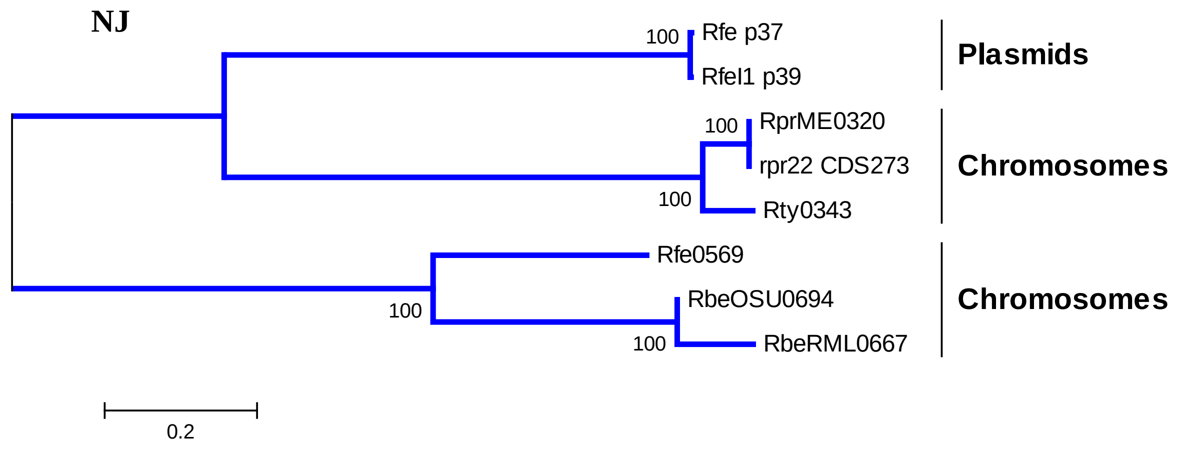

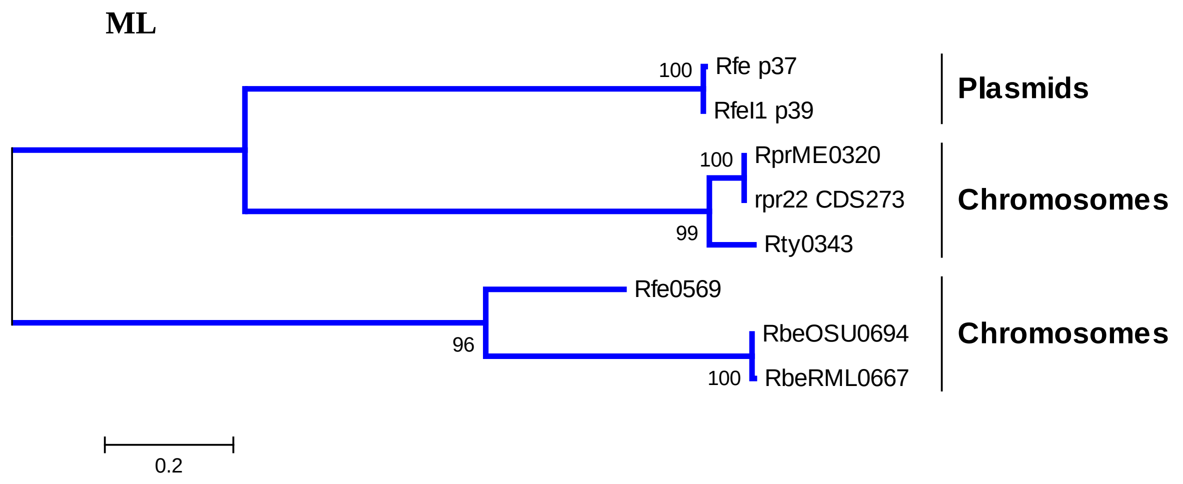
ML
100
Rfe p37
Rfel1 p39
Plasmids
100
RprME0320
rpr22 CDS273
99
Rty0343
Chromosomes
Rfe0569
96
RbeOSU0694
100
RbeRML0667
Chromosomes
0.2

Figure A26. Neighbor-joining (NJ) and maximum likelihood (ML) trees of conserved protein Of unknown function. Bootstrap supports higher than or equal to 60% are shown on the branches.

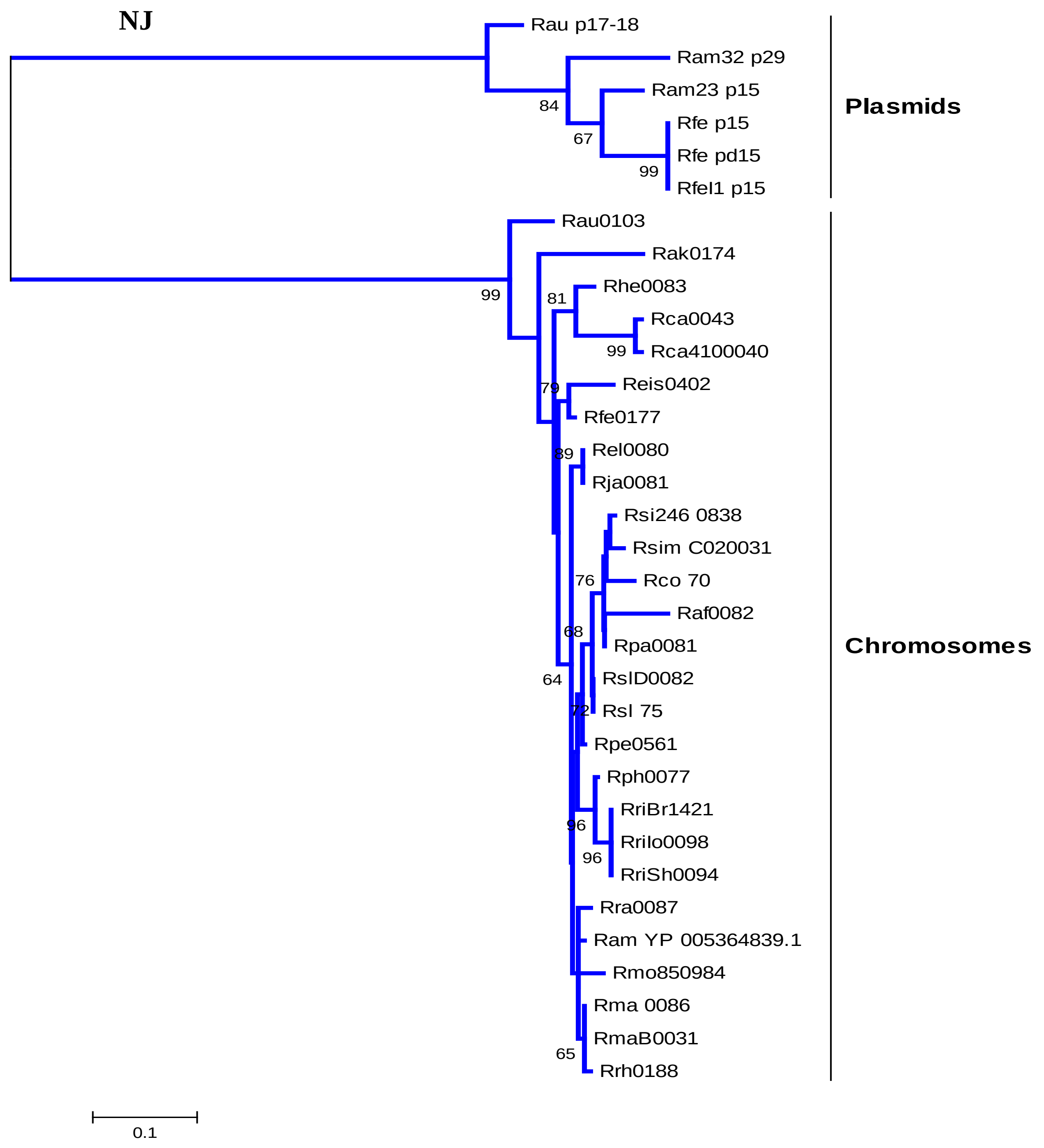

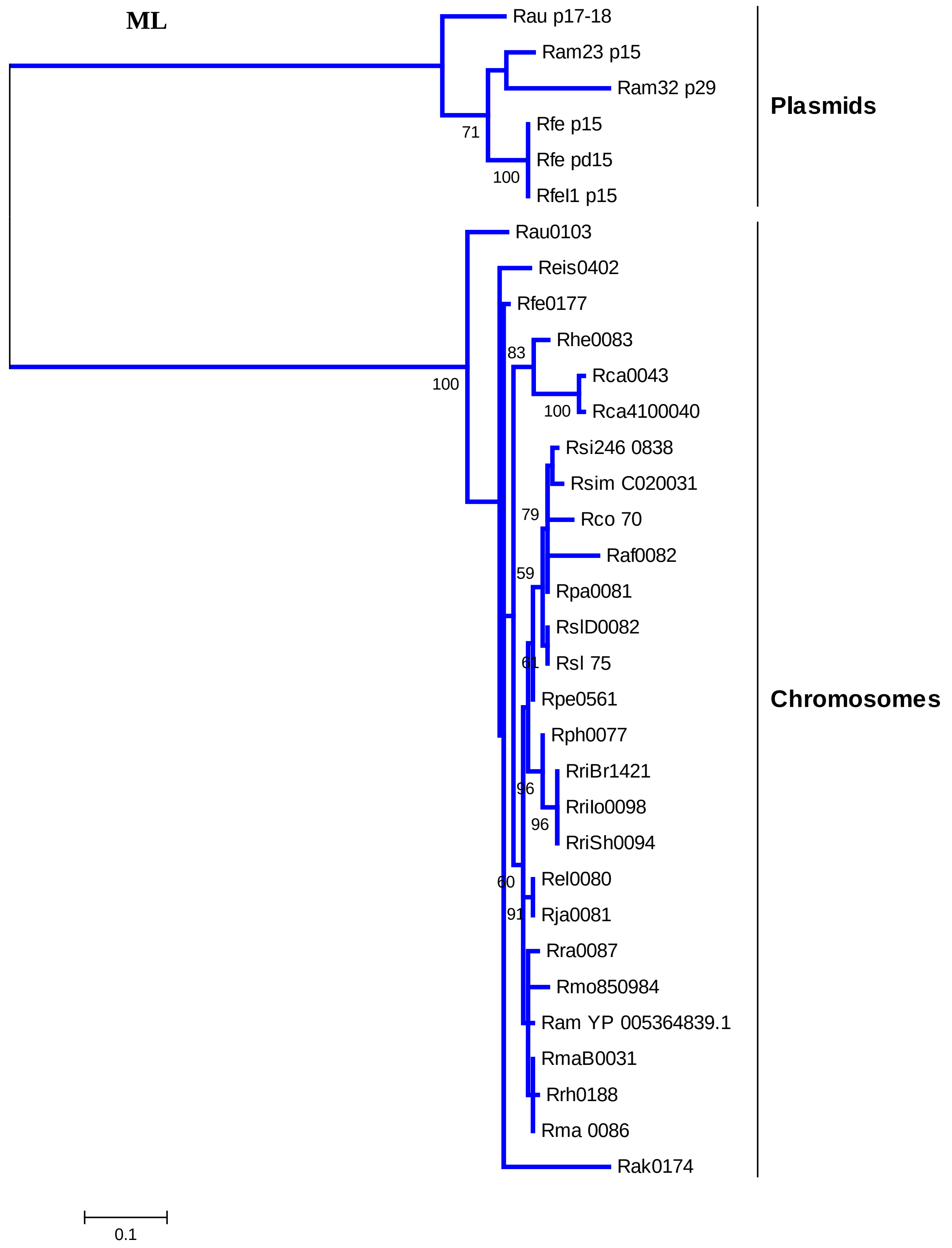
ML
Rau p17-18
Ram23 p15
Ram32 p29
71
Rfe p15
Rfe pd15
100
Rfel1 p15
Plasmids
Rau0103
Reis0402
Rfe0177
Rhe0083
83
Rca0043
100
Rca4100040
100
Rsi246 0838
Rsim C020031
79
Rco 70
Raf0082
59
Rpa0081
RslD0082
61
Rsl 75
Rpe0561
Rph0077
RriBr1421
96
Rrilo0098
96
RriSh0094
60
Rel0080
91
Rja0081
Rra0087
Rmo850984
Ram YP 005364839.1
RmaB0031
Rrh0188
Rma 0086
Rak0174
Chromosomes
0.1

Figure A27. Neighbor-joining (NJ) and maximum likelihood (ML) trees of conserved protein of unknown function. Bootstrap supports higher than or equal to 60% are shown on the branches.

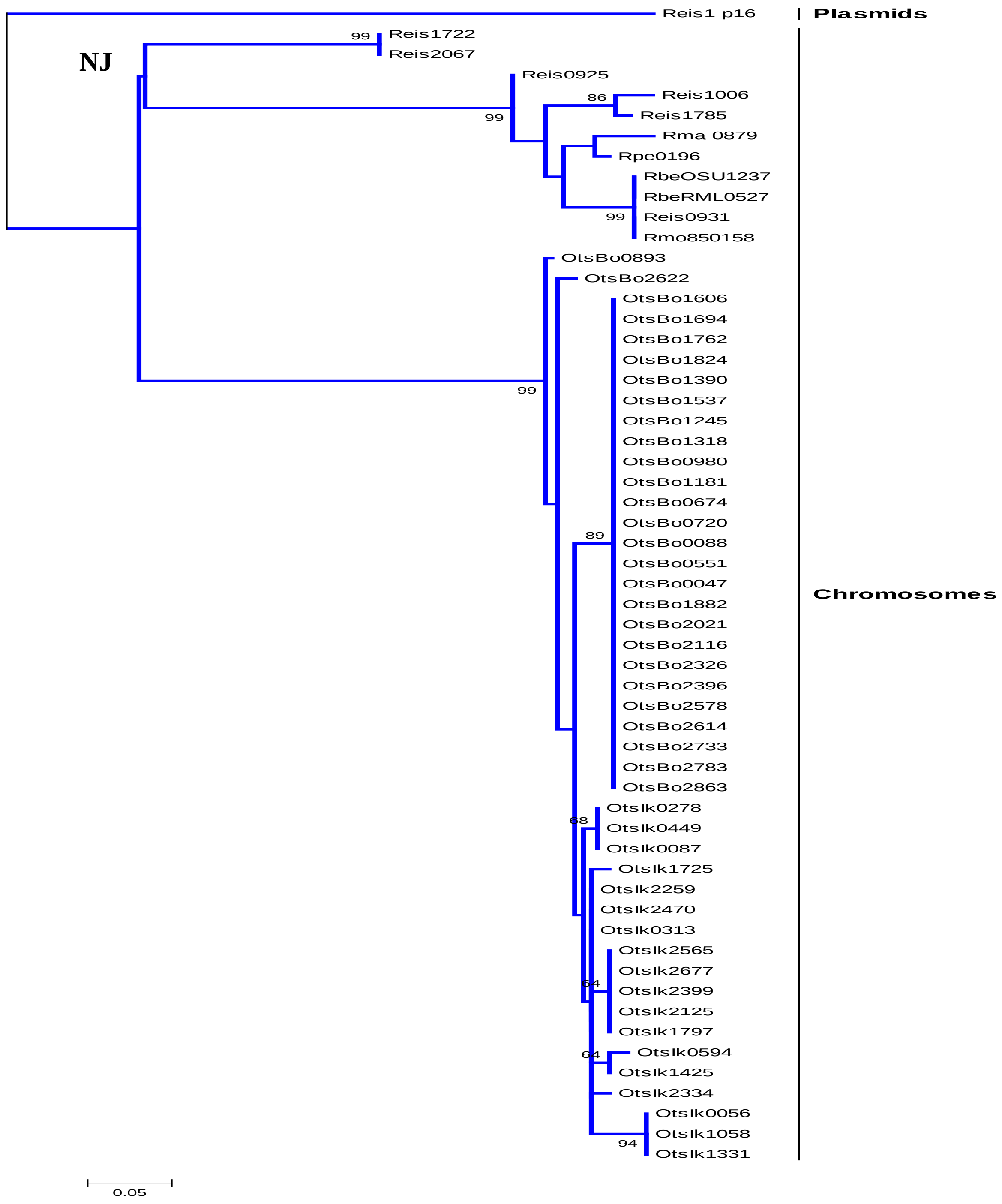

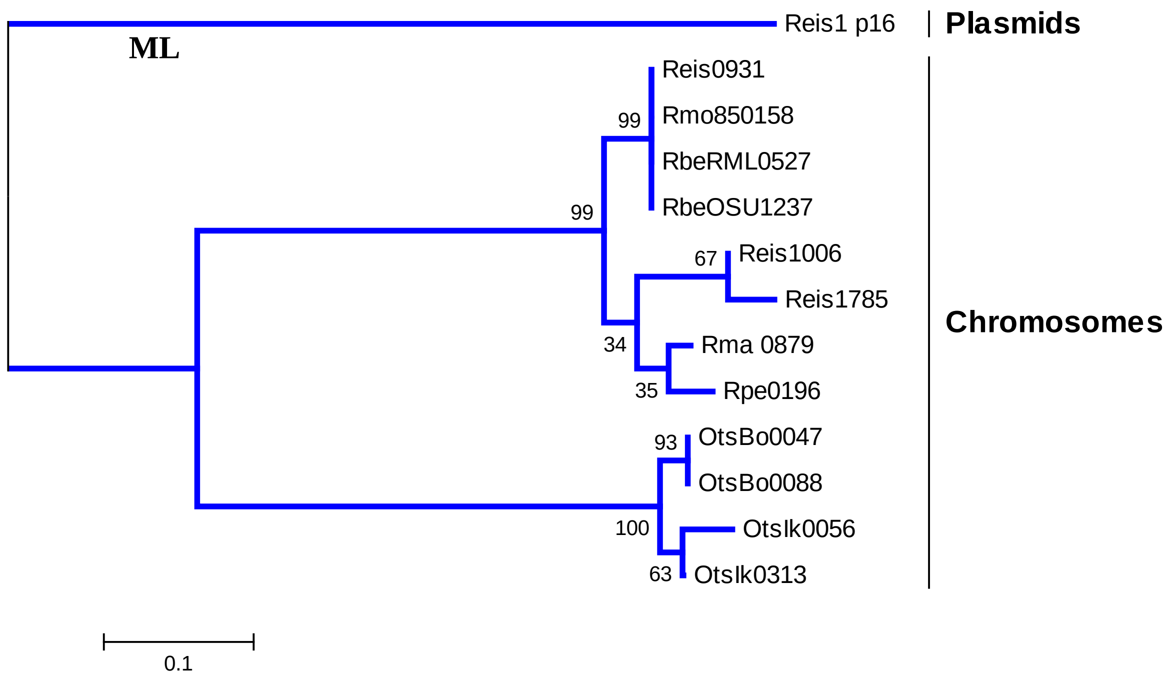
ML
Reis1 p16
Plasmids
Reis0931
Rmo850158
RbeRML0527
RbeOSU1237
Reis1006
Reis1785
Rma 0879
Rpe0196
OtsBo0047
OtsBo0088
OtsIk0056
OtsIk0313
Chromosomes
99
99
67
34
35
93
100
63
0.1

Figure A28. Neighbor-joining (NJ) and maximum likelihood (ML) trees of conserved protein of unknown function. Bootstrap supports higher than or equal to 60% are shown on the branches.

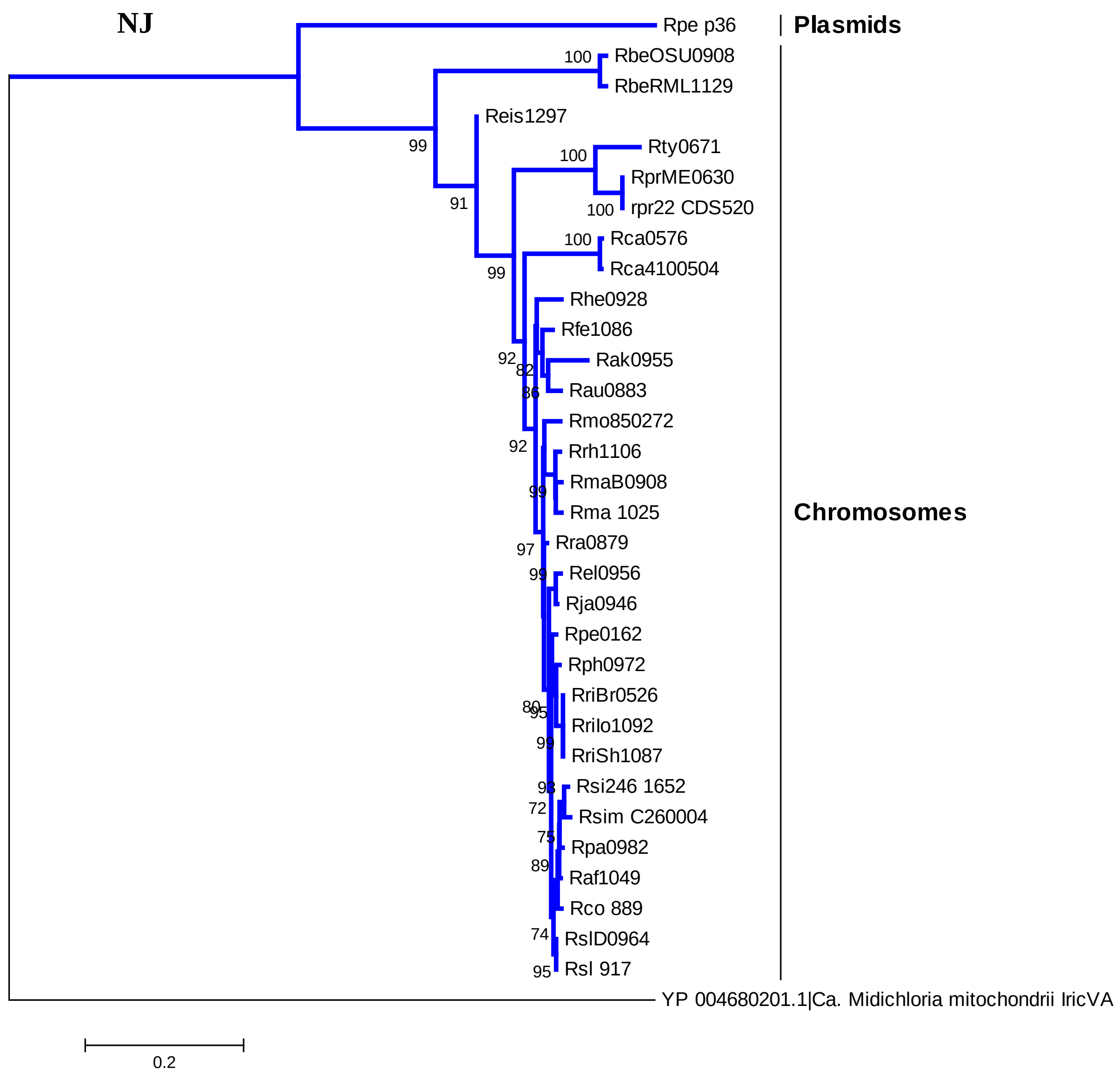

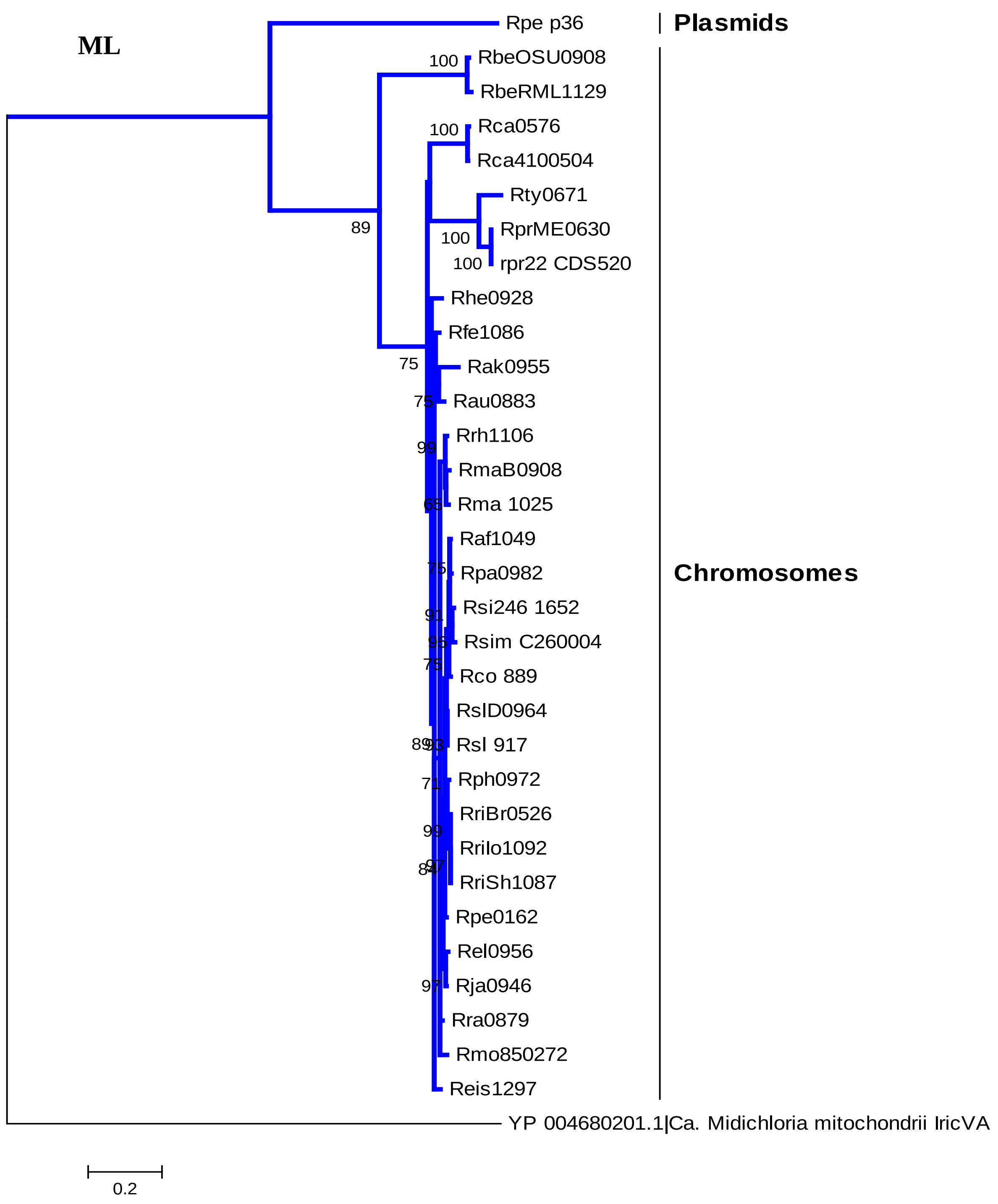

ML
Rpe p36
Plasmids
RbeOSU0908
RbeRML1129
Rca0576
Rca4100504
Rty0671
RprME0630
rpr22 CDS520
Rhe0928
Rfe1086
Rak0955
Rau0883
Rrh1106
RmaB0908
Rma 1025
Raf1049
Rpa0982
Rsi246 1652
Rsim C260004
Rco 889
RslD0964
Rsl 917
Rph0972
RriBr0526
RriIo1092
RriSh1087
Rpe0162
Rel0956
Rja0946
Rra0879
Rmo850272
Reis1297
Chromosomes
YP 004680201.1|Ca. Midichloria mitochondrii IricVA
0.2

Figure A29. Neighbor-joining (NJ) and maximum likelihood (ML) trees of conserved protein of unknown function. Bootstrap supports higher than or equal to 60% are shown on the branches.

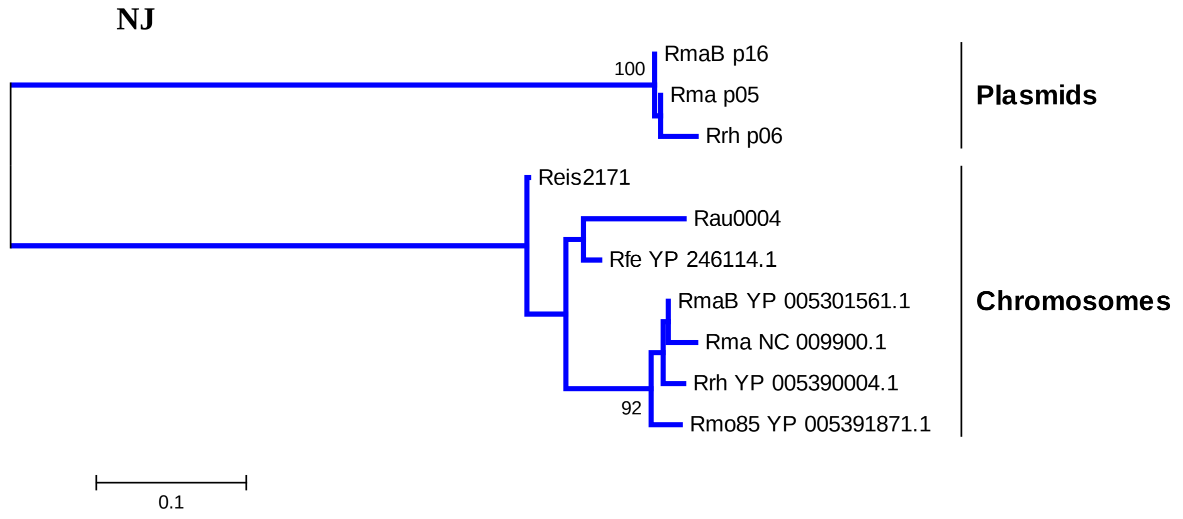

ML

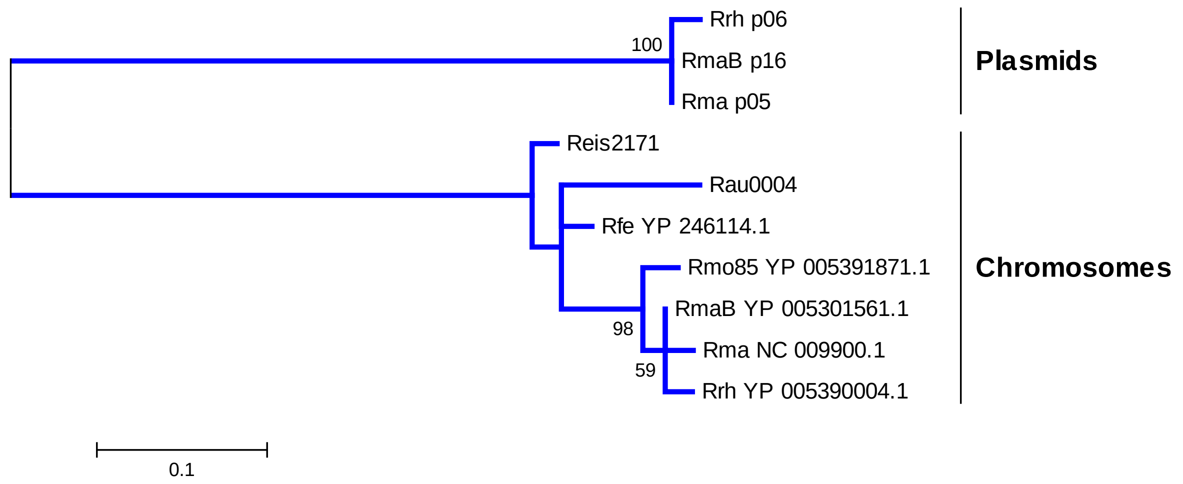

Rrh p06
100
RmaB p16
Rma p05
Plasmids
Reis2171
Rau0004
Rfe YP 246114.1
Rmo85 YP 005391871.1
RmaB YP 005301561.1
98
Rma NC 009900.1
59
Rrh YP 005390004.1
Chromosomes
0.1

Figure A30. Neighbor-joining (NJ) and maximum likelihood (ML) trees of conserved protein of unknown function. Bootstrap supports higher than or equal to 60% are shown on the branches.

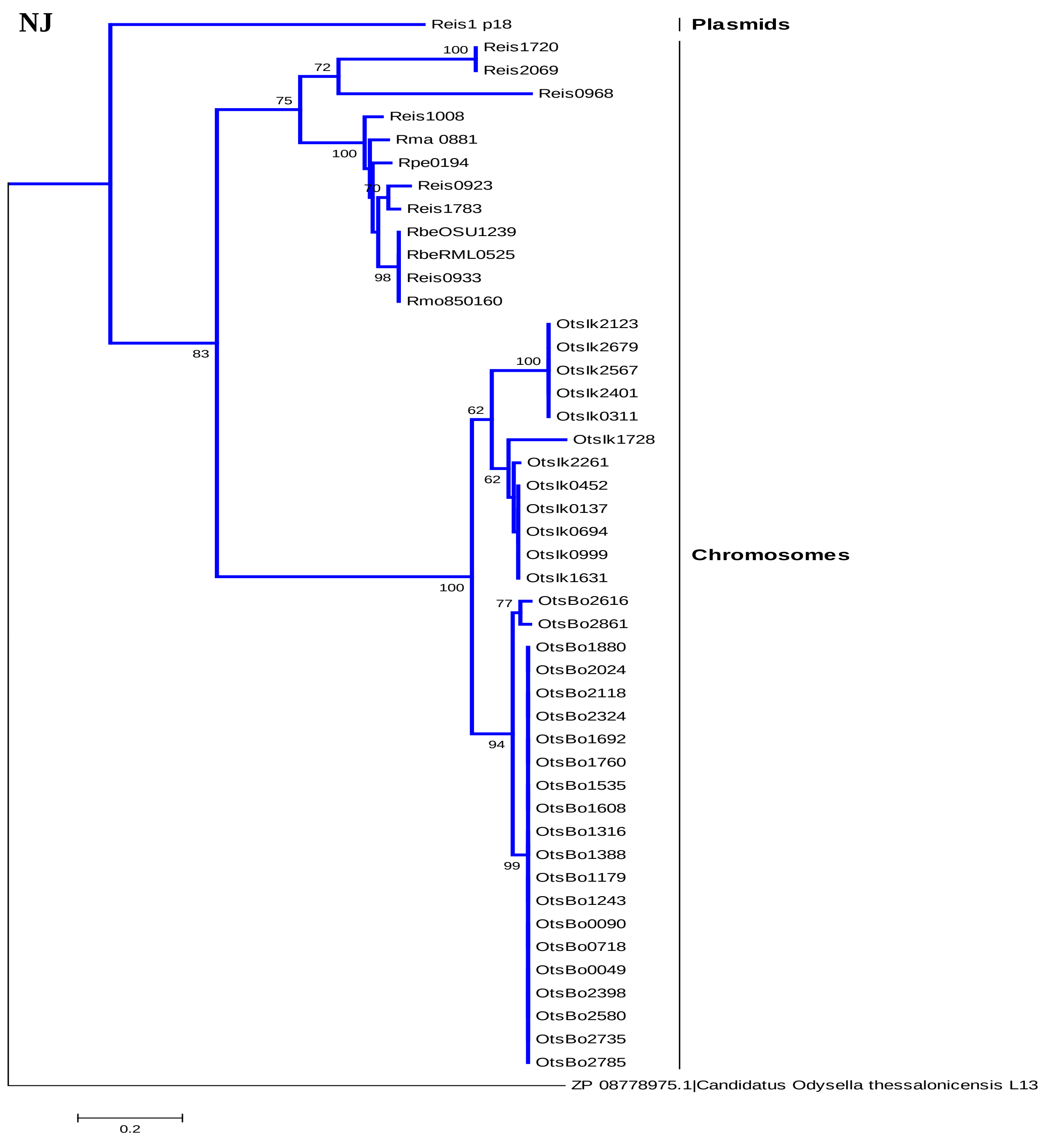

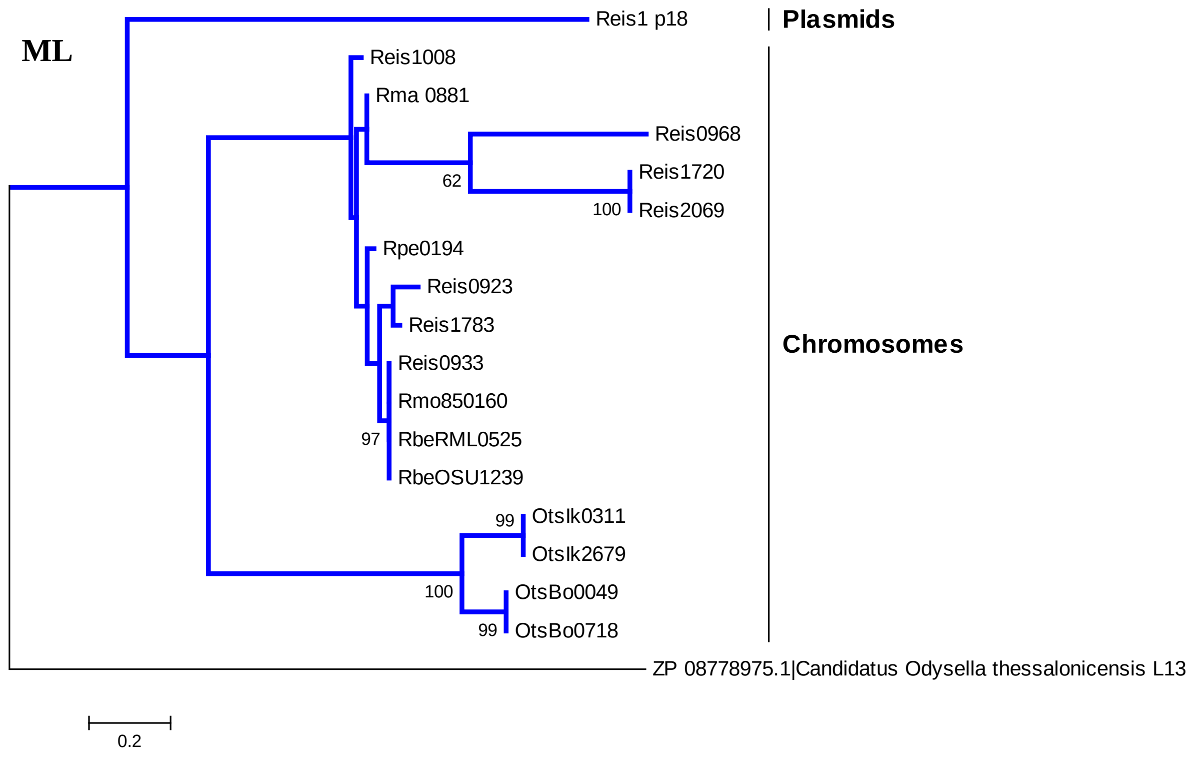
ML
Reis1 p18
Plasmids
Reis1008
Rma 0881
Reis0968
62
Reis1720
100
Reis2069
Rpe0194
Reis0923
Reis1783
Reis0933
Rmo850160
97
RbeRML0525
RbeOSU1239
Chromosomes
99
OtsIk0311
OtsIk2679
100
OtsBo0049
99
OtsBo0718
ZP 08778975.1|Candidatus Odysella thessalonicensis L13
0.2

Figure A31. Neighbor-joining (NJ) and maximum likelihood (ML) trees of conserved protein of unknown function. Bootstrap supports higher than or equal to 60% are shown on the branches.

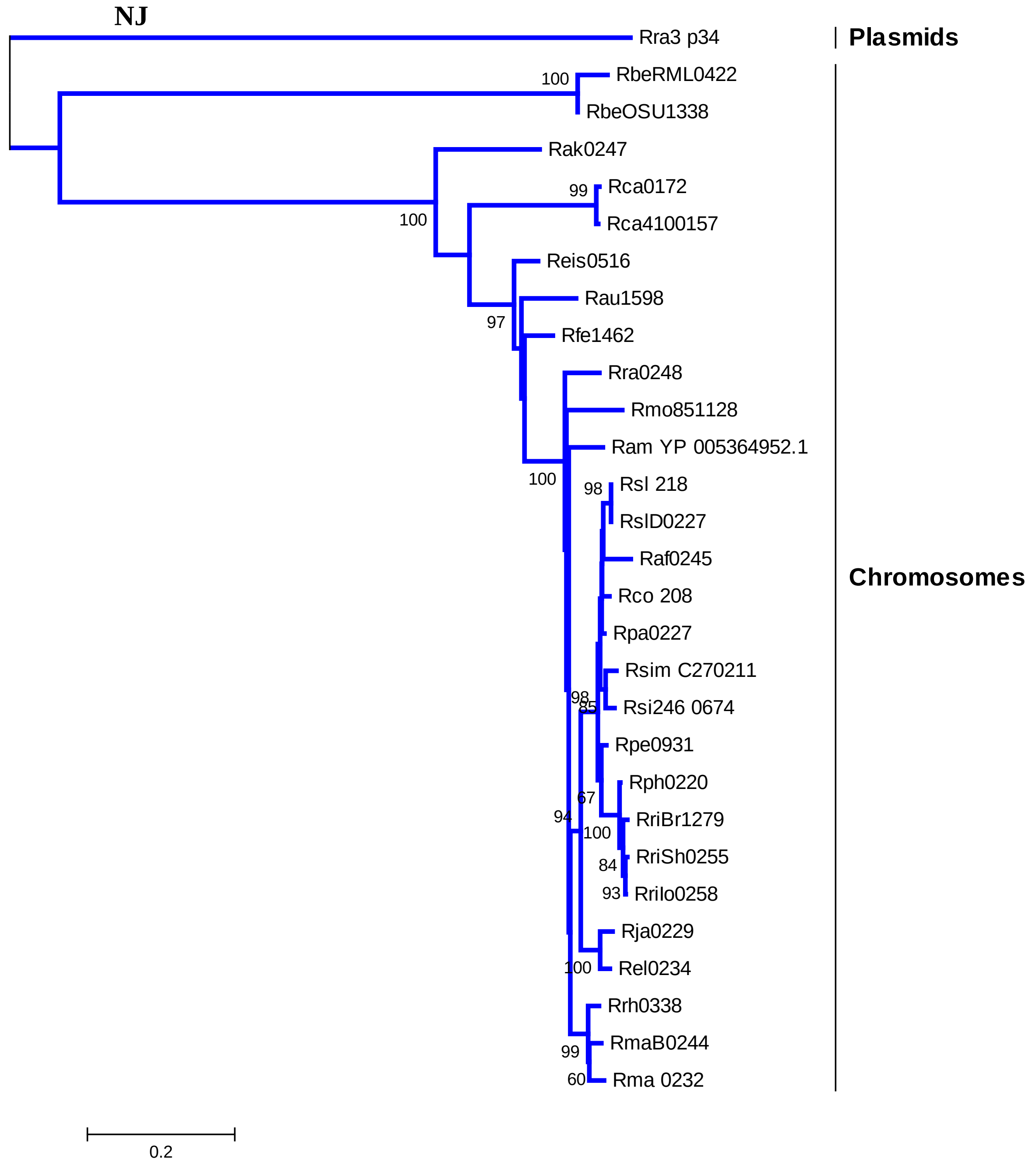

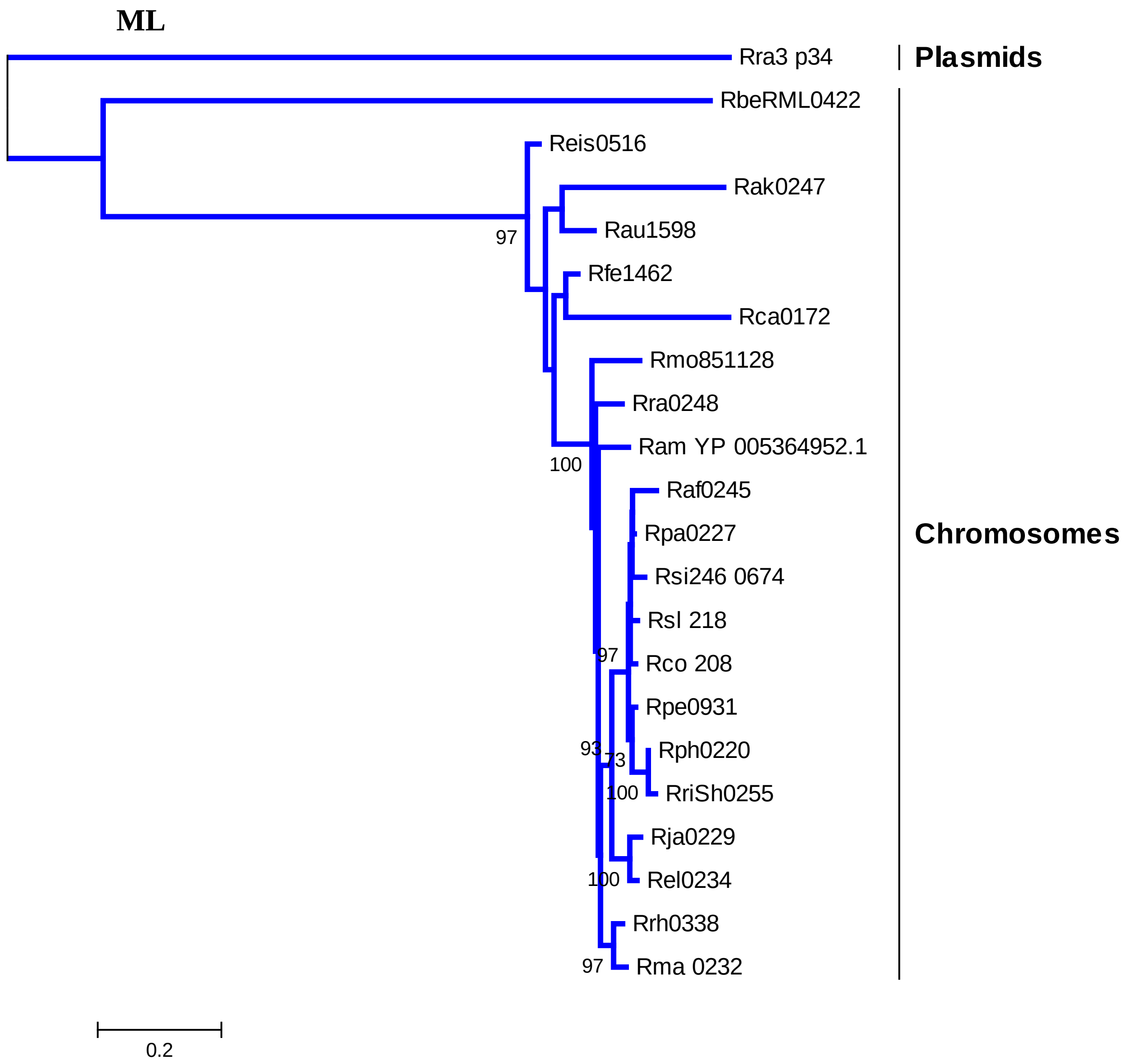
ML
Rra3 p34
RbeRML0422
Reis0516
Rak0247
Rau1598
Rfe1462
Rca0172
Rmo851128
Rra0248
Ram YP 005364952.1
Raf0245
Rpa0227
Rsi246 0674
Rsl 218
Rco 208
Rpe0931
Rph0220
RriSh0255
Rja0229
Rel0234
Rrh0338
Rma 0232
97
100
97
93
73
100
100
97
Plasmids
Chromosomes
0.2

Figure A32. Neighbor-joining (NJ) and maximum likelihood (ML) trees of conserved protein of unknown function. Bootstrap supports higher than or equal to 60% are shown on the branches.

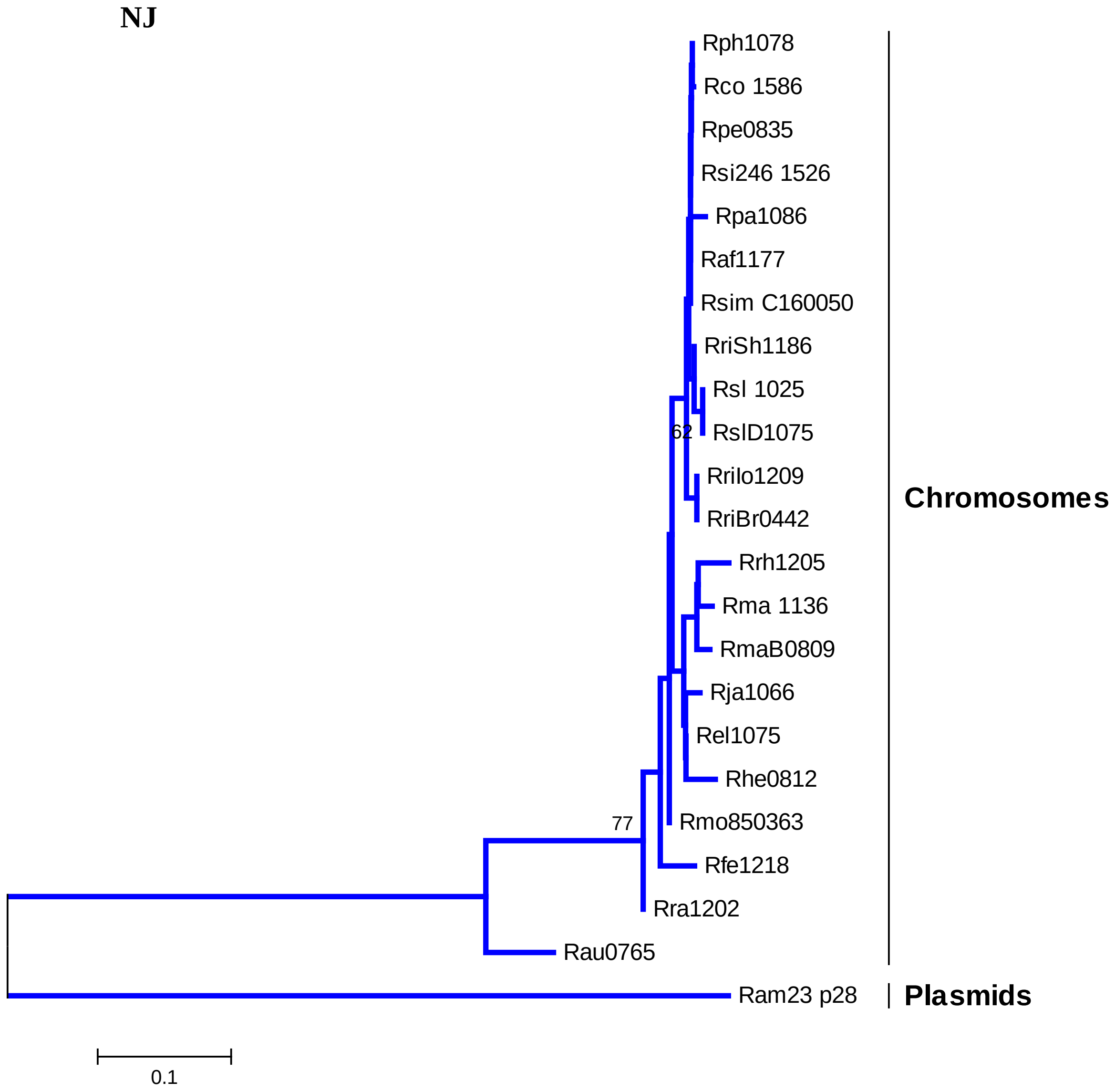

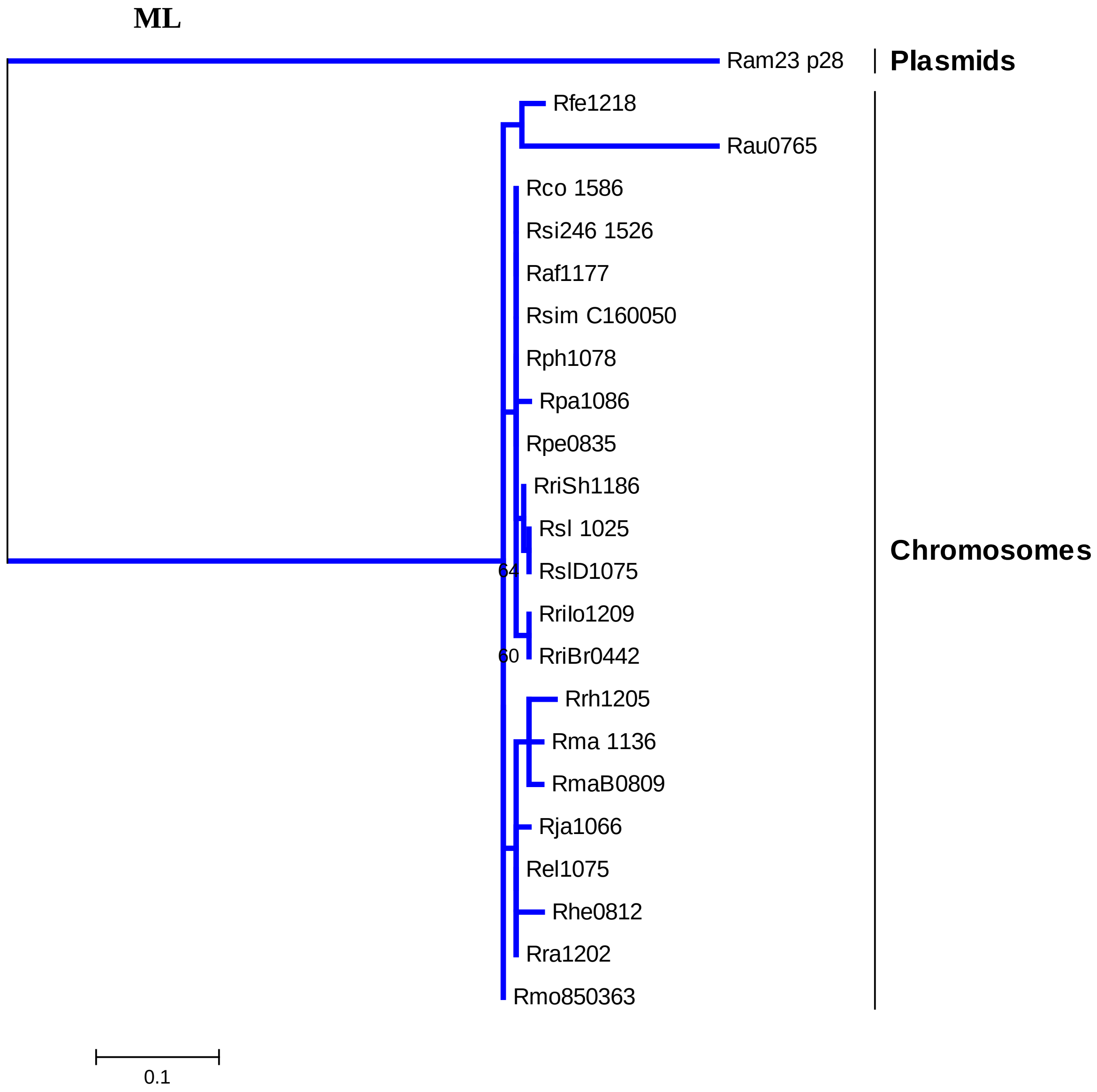
ML
Ram23 p28
Plasmids
Rfe1218
Rau0765
Rco 1586
Rsi246 1526
Raf1177
Rsim C160050
Rph1078
Rpa1086
Rpe0835
RriSh1186
Rsl 1025
RslD1075
64
Rrilo1209
RriBr0442
60
Rrh1205
Rma 1136
RmaB0809
Rja1066
Rel1075
Rhe0812
Rra1202
Rmo850363
Chromosomes
0.1

Figure A33. Neighbor-joining (NJ) and maximum likelihood (ML) trees of conserved protein of unknown function. Bootstrap supports higher than or equal to 60% are shown on the branches.

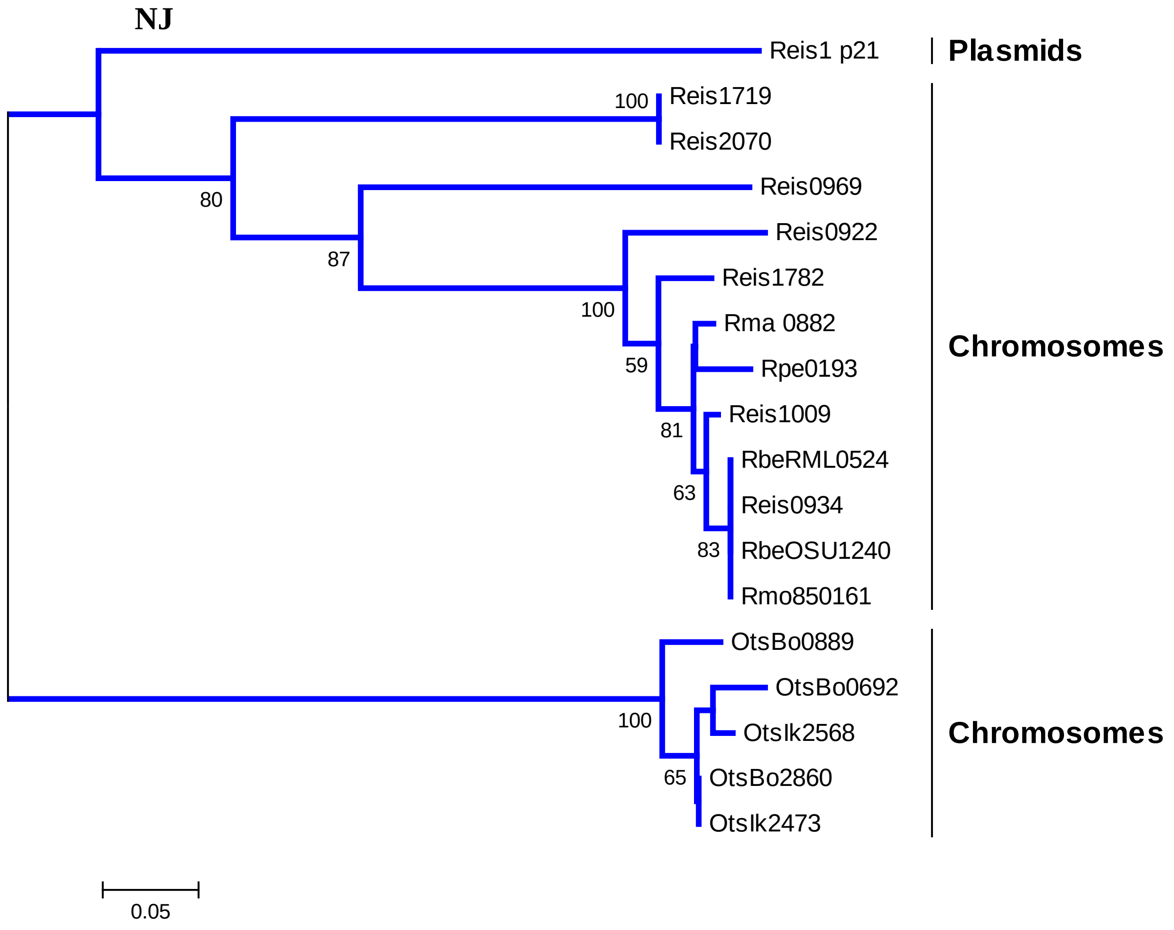

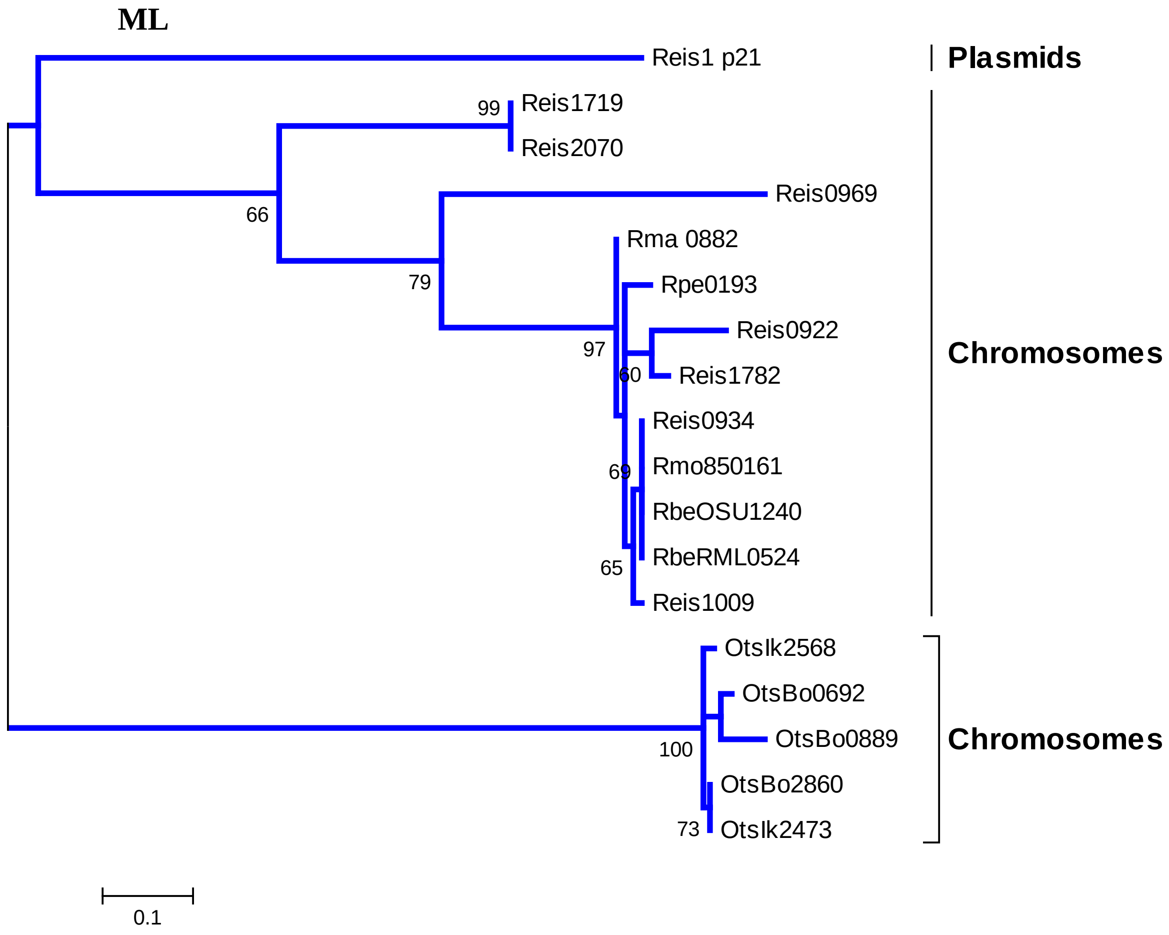
ML
Reis1 p21
Plasmids
99
Reis1719
Reis2070
66
Reis0969
79
Rma 0882
Rpe0193
Reis0922
97
60
Reis1782
Reis0934
69
Rmo850161
RbeOSU1240
RbeRML0524
65
Reis1009
Chromosomes
OtsIk2568
OtsBo0692
OtsBo0889
100
OtsBo2860
73
OtsIk2473
Chromosomes
0.1

Figure A34. Neighbor-joining (NJ) and maximum likelihood (ML) trees of guanosine polyphosphate pyrophosphohydrolase/synthetase SpoT22. Bootstrap supports higher than or equal to 60% are shown on the branches.

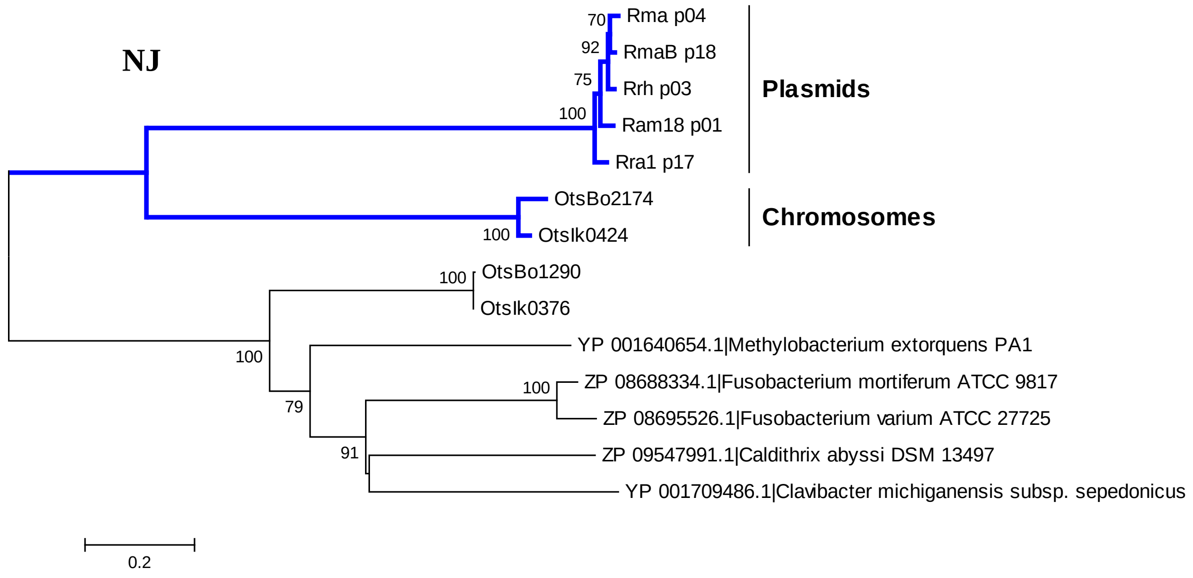

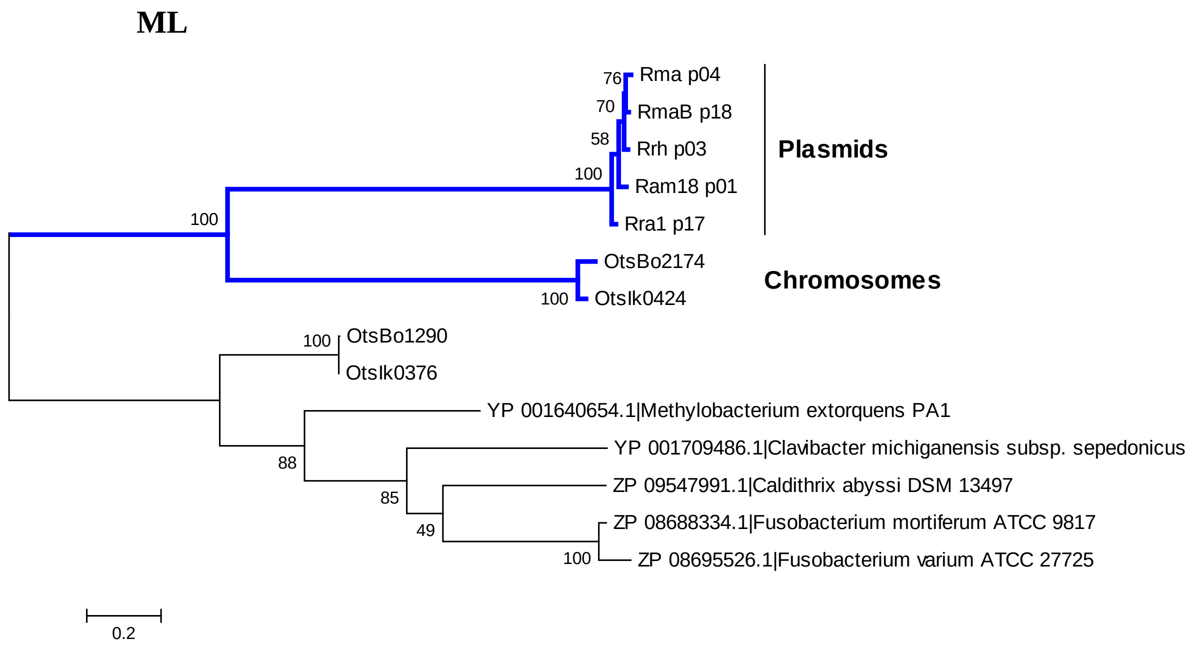

ML
Rma p04
RmaB p18
Rrh p03
Ram18 p01
Rra1 p17
Plasmids
OtsBo2174
OtsIk0424
Chromosomes
OtsBo1290
OtsIk0376
YP 001640654.1|Methylobacterium extorquens PA1
YP 001709486.1|Clavibacter michiganensis subsp. sepedonicus
ZP 09547991.1|Caldithrix abyssi DSM 13497
ZP 08688334.1|Fusobacterium mortiferum ATCC 9817
ZP 08695526.1|Fusobacterium varium ATCC 27725
76
70
58
100
100
100
100
88
85
49
100
0.2

Figure A35. Neighbor-joining (NJ) and maximum likelihood (ML) trees of cell surface antigen Sca4. Bootstrap supports higher than or equal to 60% are shown on the branches.

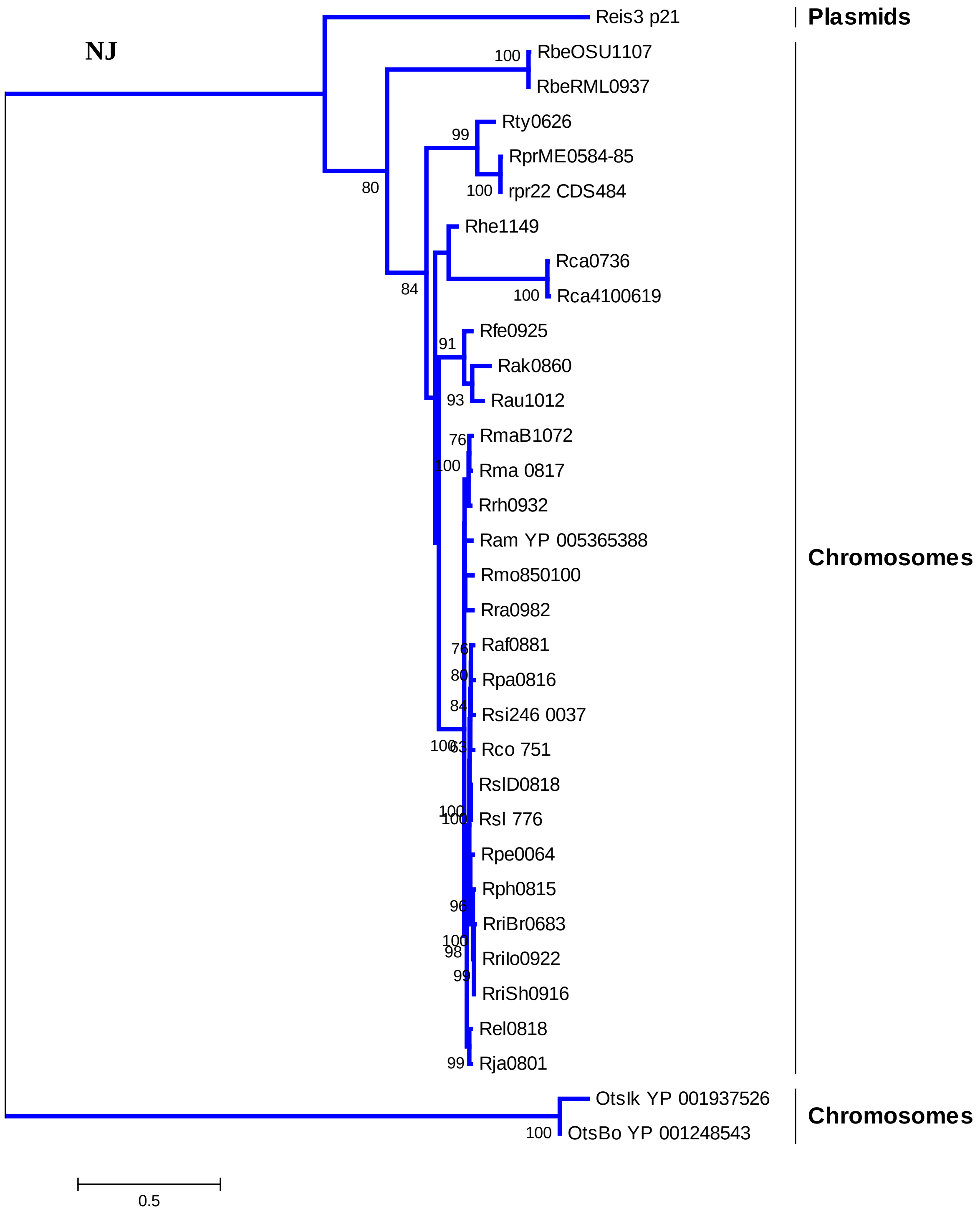

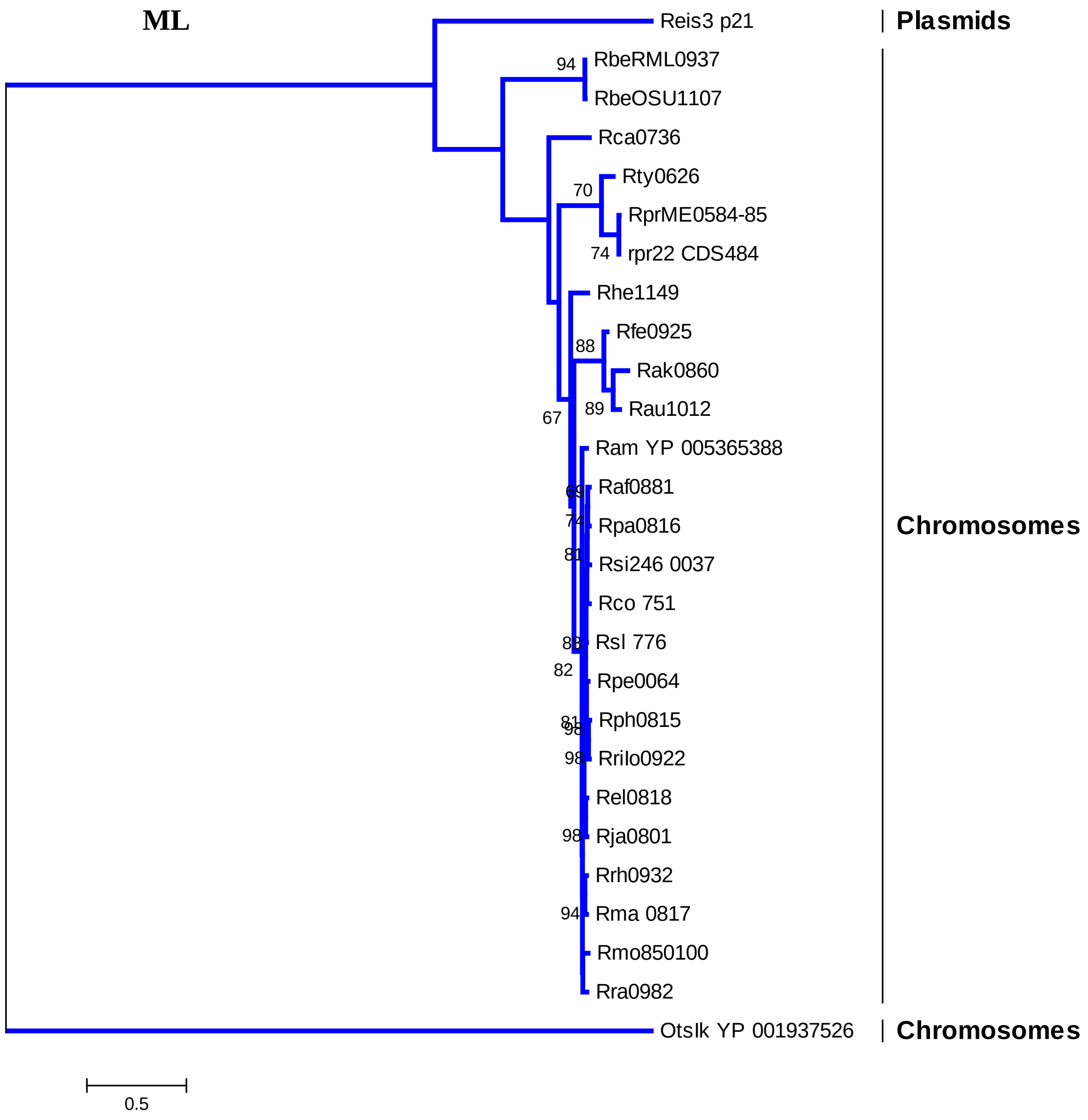
ML
Reis3 p21
Plasmids
RbeRML0937
RbeOSU1107
Rca0736
Rty0626
RprME0584-85
rpr22 CDS484
Rhe1149
Rfe0925
Rak0860
Rau1012
Ram YP 005365388
Raf0881
Rpa0816
Rsi246 0037
Rco 751
Rsl 776
Rpe0064
Rph0815
Rrilo0922
Rel0818
Rja0801
Rrh0932
Rma 0817
Rmo850100
Rra0982
Chromosomes
OtsIk YP 001937526
Chromosomes
0.5

Figure A36. Neighbor-joining (NJ) and maximum likelihood (ML) trees of conserved protein of unknown function.

NJ

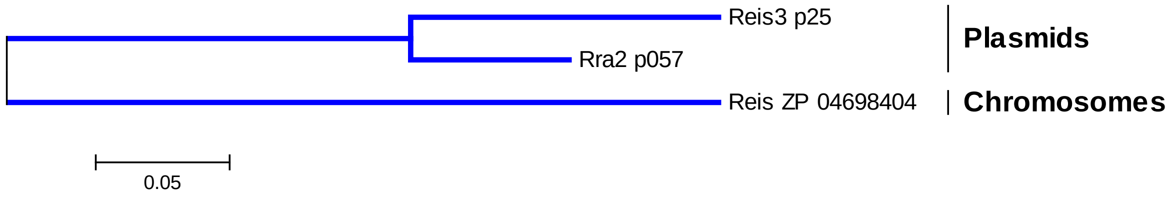

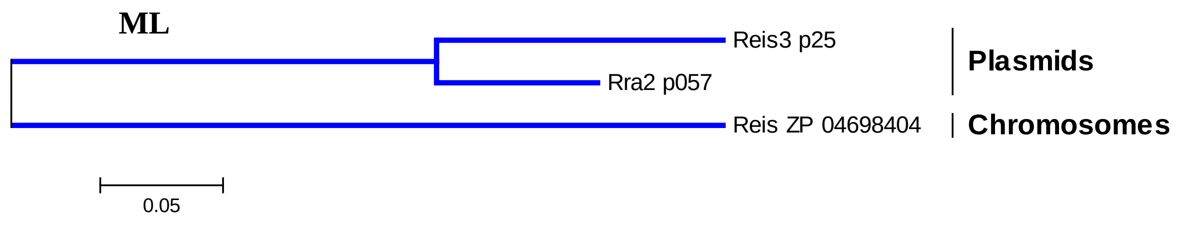
ML
Reis3 p25
Rra2 p057
Reis ZP 04698404
Plasmids
Chromosomes
0.05

Figure A37. Neighbor-joining (NJ) and maximum likelihood (ML) trees of conserved protein of transposase DDE-Tnp_1 domain. Bootstrap supports higher than or equal to 60% are shown on the branches.

.

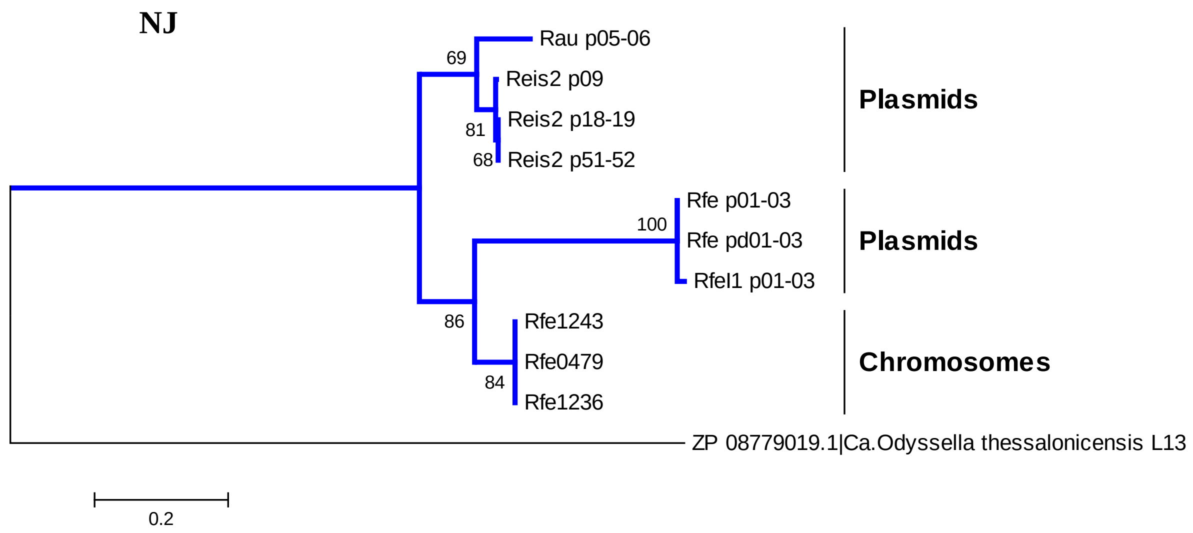

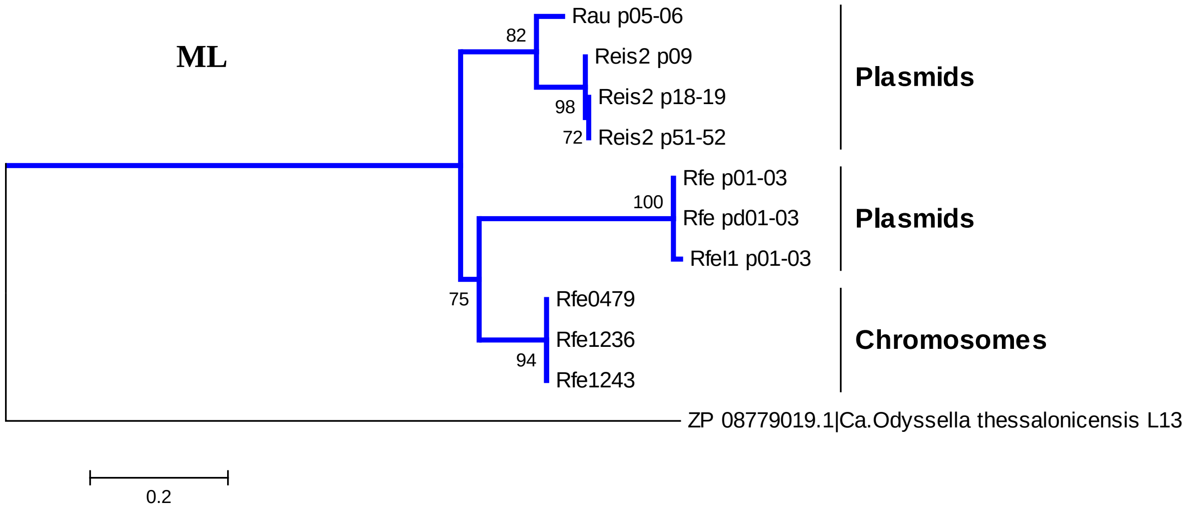
ML
Rau p05-06
Reis2 p09
Reis2 p18-19
Reis2 p51-52
Rfe p01-03
Rfe pd01-03
Rfel1 p01-03
Rfe0479
Rfe1236
Rfe1243
ZP 08779019.1|Ca.Odyssella thessalonicensis L13
82
98
72
100
75
94
Plasmids
Plasmids
Chromosomes
0.2

Figure A38. Neighbor-joining (NJ) and maximum likelihood (ML) trees of conjugative transfer protein containing TraW_N domain. Bootstrap supports higher than or equal to 60% are shown on the branches.

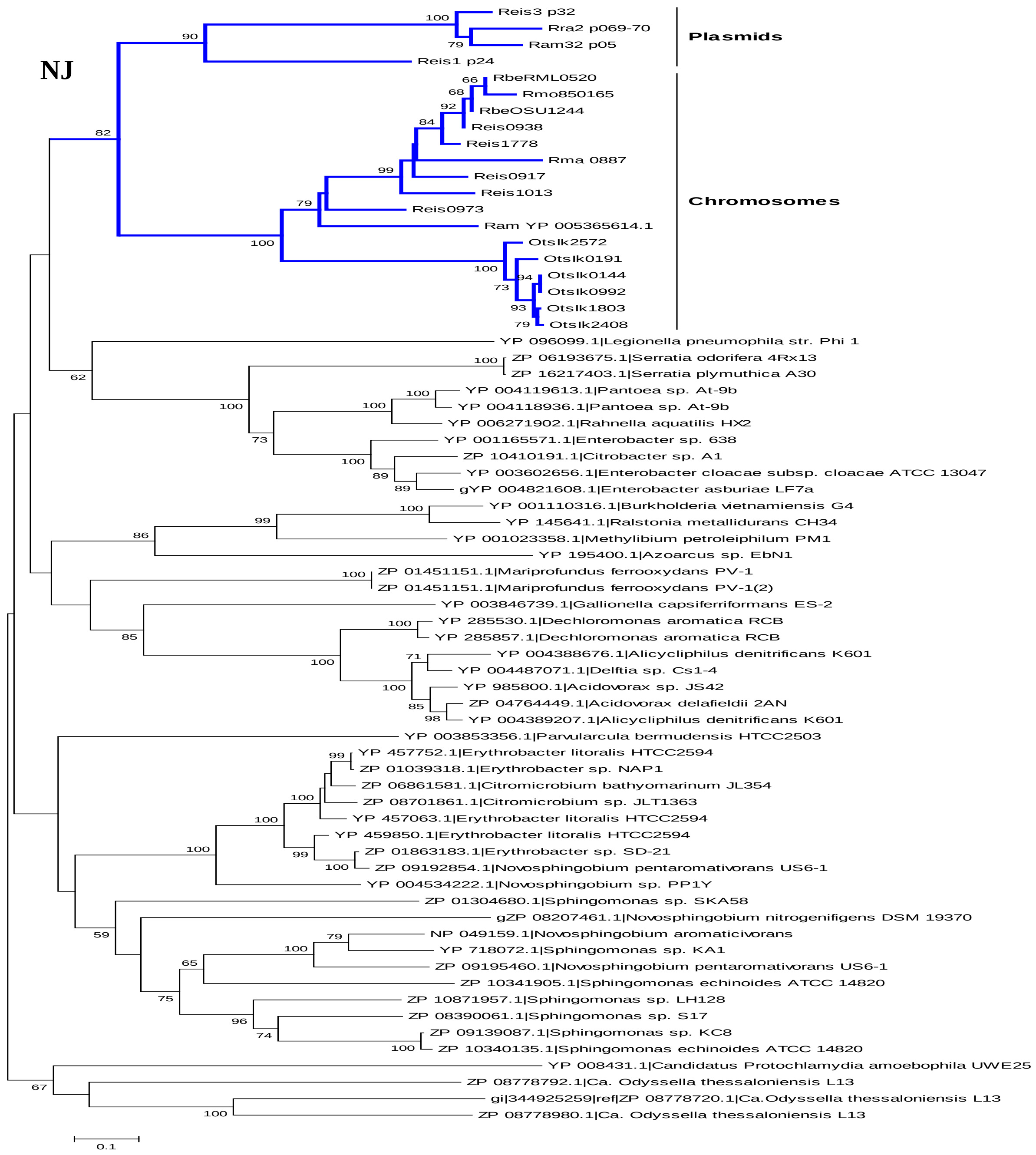

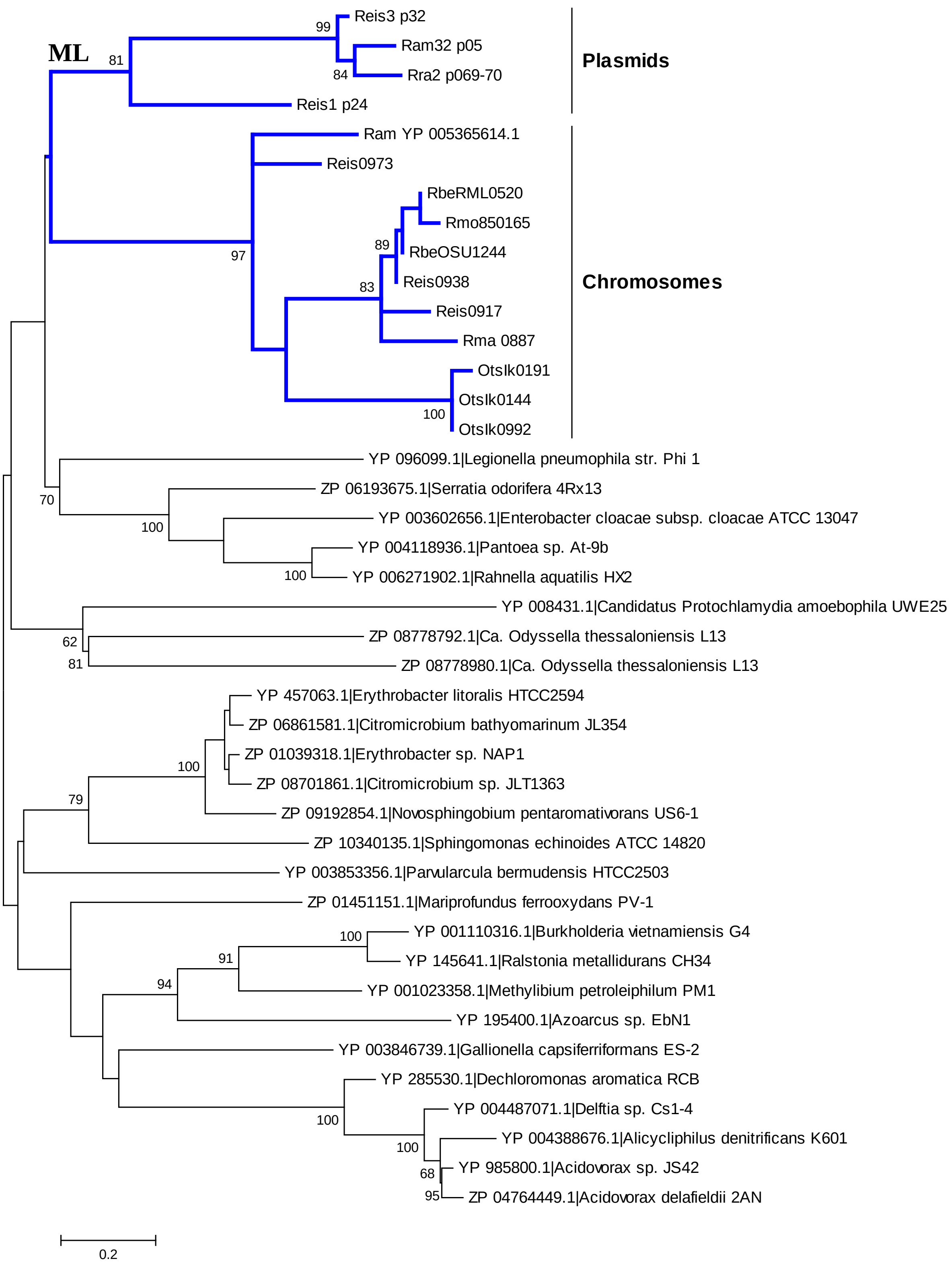

ML
Reis3 p32
Ram32 p05
Rra2 p069-70
Reis1 p24
Plasmids
Ram YP 005365614.1
Reis0973
RbeRML0520
Rmo850165
RbeOSU1244
Reis0938
Reis0917
Rma 0887
OtsIk0191
OtsIk0144
OtsIk0992
Chromosomes
YP 096099.1|Legionella pneumophila str. Phi 1
ZP 06193675.1|Serratia odorifera 4Rx13
YP 003602656.1|Enterobacter cloacae subsp. cloacae ATCC 13047
YP 004118936.1|Pantoea sp. At-9b
YP 006271902.1|Rahnella aquatilis HX2
YP 008431.1|Candidatus Protochlamydia amoebophila UWE25
ZP 08778792.1|Ca. Odyssella thessaloniensis L13
ZP 08778980.1|Ca. Odyssella thessaloniensis L13
YP 457063.1|Erythrobacter litoralis HTCC2594
ZP 06861581.1|Citromicrobium bathyomarinum JL354
ZP 01039318.1|Erythrobacter sp. NAP1
ZP 08701861.1|Citromicrobium sp. JLT1363
ZP 09192854.1|Novosphingobium pentaromativorans US6-1
ZP 10340135.1|Sphingomonas echinoides ATCC 14820
YP 003853356.1|Parvularcula bermudensis HTCC2503
ZP 01451151.1|Mariprofundus ferrooxydans PV-1
YP 001110316.1|Burkholderia vietnamiensis G4
YP 145641.1|Ralstonia metallidurans CH34
YP 001023358.1|Methylibium petroleiphilum PM1
YP 195400.1|Azoarcus sp. EbN1
YP 003846739.1|Gallionella capsiferriformans ES-2
YP 285530.1|Dechloromonas aromatica RCB
YP 004487071.1|Delftia sp. Cs1-4
YP 004388676.1|Alicycliphilus denitrificans K601
YP 985800.1|Acidovorax sp. JS42
ZP 04764449.1|Acidovorax delafieldii 2AN
99
84
81
97
89
83
100
70
100
100
62
81
100
79
100
91
94
100
100
68
95
0.2

Figure A39. Neighbor-joining (NJ) and maximum likelihood (ML) trees of conjugative transfer protein containing TraG domain. Bootstrap supports higher than or equal to 60% are shown on the branches.

NJ

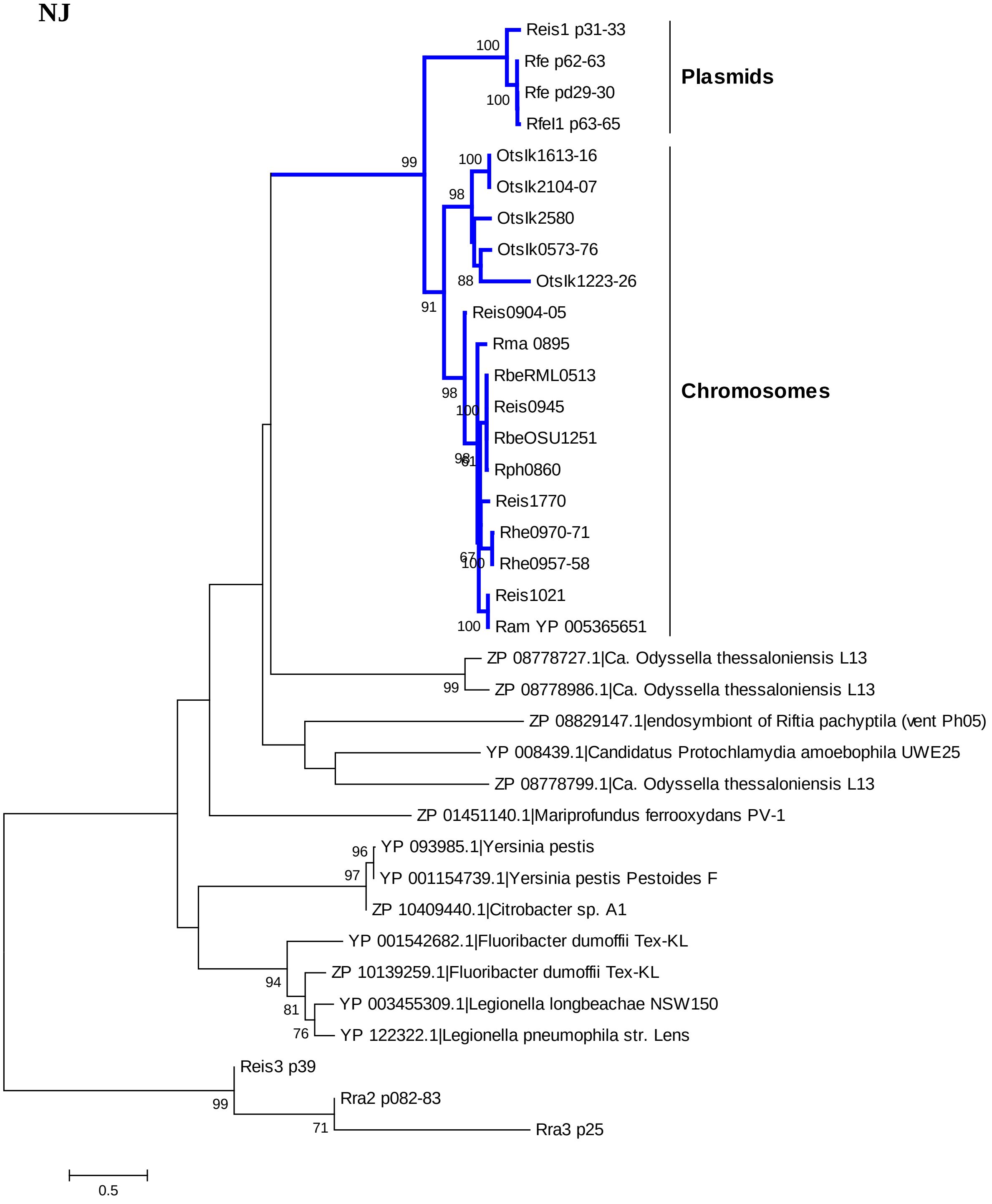

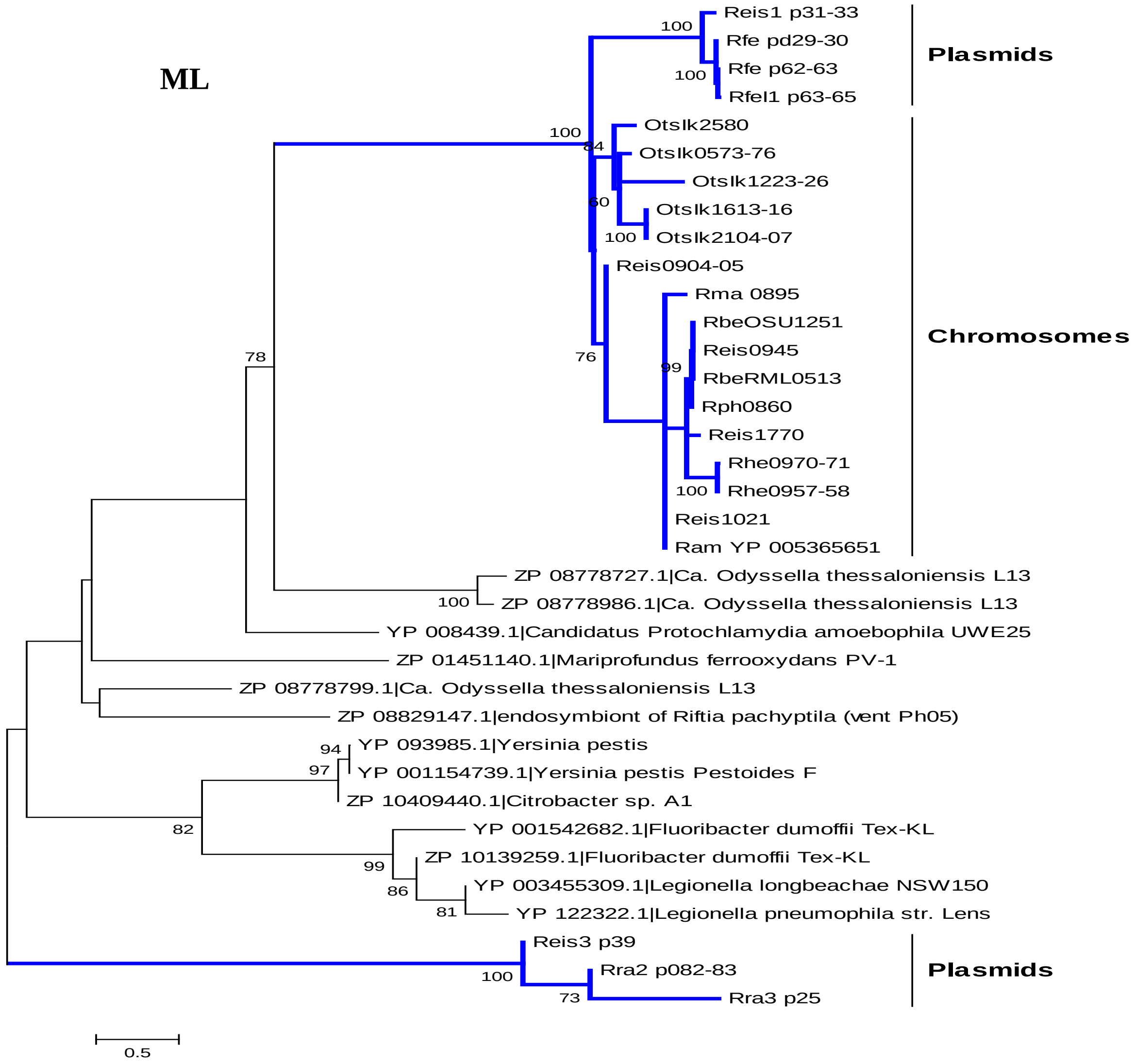
ML
Reis1 p31-33
Rfe pd29-30
Rfe p62-63
Rfel1 p63-65
Plasmids
OtsIk2580
OtsIk0573-76
OtsIk1223-26
OtsIk1613-16
OtsIk2104-07
Reis0904-05
Rma 0895
RbeOSU1251
Reis0945
RbeRML0513
Rph0860
Reis1770
Rhe0970-71
Rhe0957-58
Reis1021
Ram YP 005365651
Chromosomes
ZP 08778727.1|Ca. Odyssella thessaloniensis L13
ZP 08778986.1|Ca. Odyssella thessaloniensis L13
YP 008439.1|Candidatus Protochlamydia amoebophila UWE25
ZP 01451140.1|Mariprofundus ferrooxydans PV-1
ZP 08778799.1|Ca. Odyssella thessaloniensis L13
ZP 08829147.1|endosymbiont of Riftia pachyptila (vent Ph05)
YP 093985.1|Yersinia pestis
YP 001154739.1|Yersinia pestis Pestoides F
ZP 10409440.1|Citrobacter sp. A1
YP 001542682.1|Fluoribacter dumoffii Tex-KL
ZP 10139259.1|Fluoribacter dumoffii Tex-KL
YP 003455309.1|Legionella longbeachae NSW150
YP 122322.1|Legionella pneumophila str. Lens
Reis3 p39
Rra2 p082-83
Rra3 p25
Plasmids
100
100
100
84
60
100
76
99
100
78
100
94
97
82
99
86
81
100
73
0.5

Figure A40. Neighbor-joining (NJ) and maximum likelihood (ML) trees of conjugative transfer protein containing TraH domain. Bootstrap supports higher than or equal to 60% are shown on the branches.

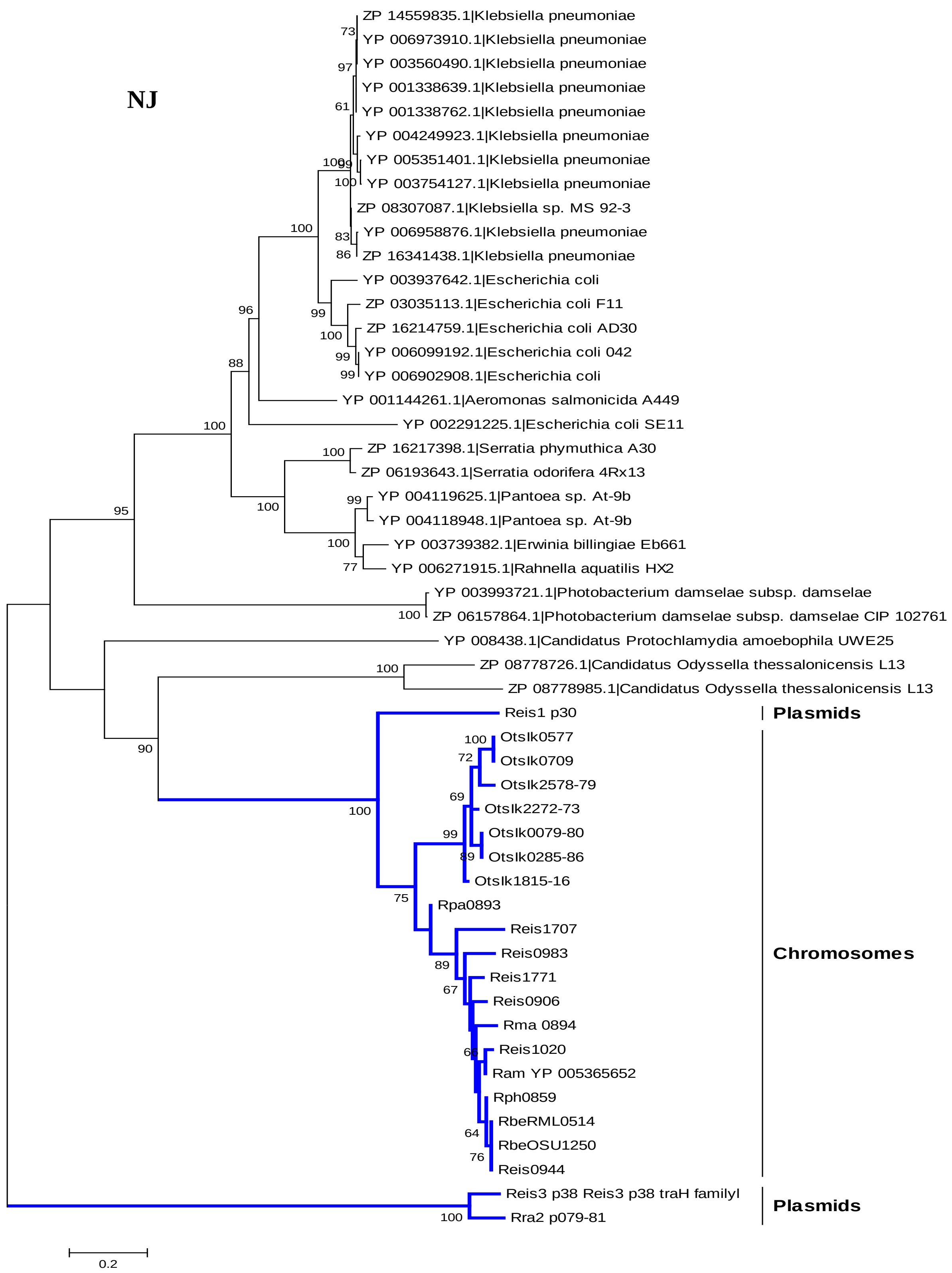

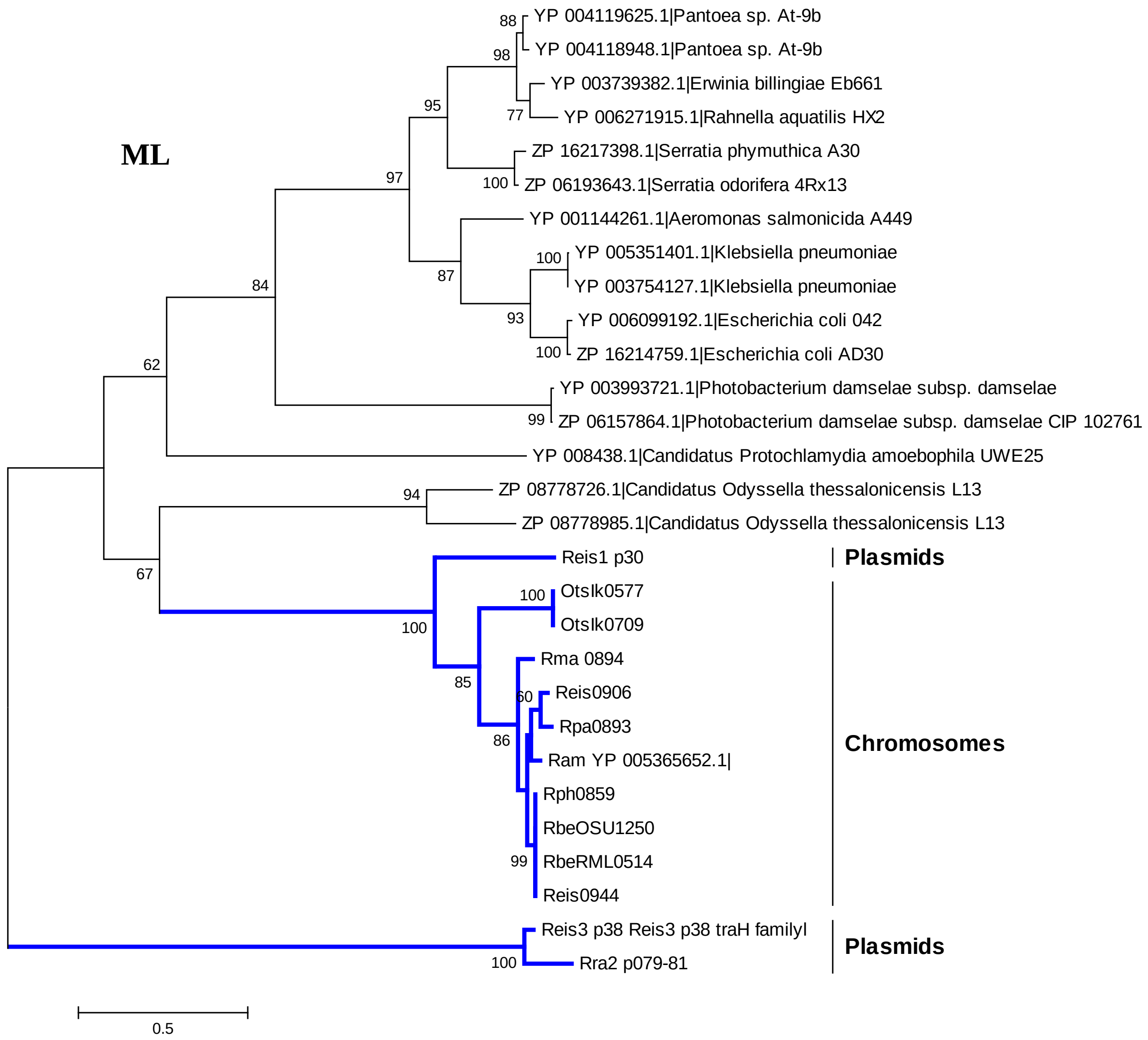
ML
YP 004119625.1|Pantoea sp. At-9b
YP 004118948.1|Pantoea sp. At-9b
YP 003739382.1|Erwinia billingiae Eb661
YP 006271915.1|Rahnella aquatilis HX2
ZP 16217398.1|Serratia phymuthica A30
ZP 06193643.1|Serratia odorifera 4Rx13
YP 001144261.1|Aeromonas salmonicida A449
YP 005351401.1|Klebsiella pneumoniae
YP 003754127.1|Klebsiella pneumoniae
YP 006099192.1|Escherichia coli 042
ZP 16214759.1|Escherichia coli AD30
YP 003993721.1|Photobacterium damselae subsp. damselae
ZP 06157864.1|Photobacterium damselae subsp. damselae CIP 102761
YP 008438.1|Candidatus Protochlamydia amoebophila UWE25
ZP 08778726.1|Candidatus Odyssella thessalonicensis L13
ZP 08778985.1|Candidatus Odyssella thessalonicensis L13
Reis1 p30
OtsIk0577
OtsIk0709
Rma 0894
Reis0906
Rpa0893
Ram YP 005365652.1|
Rph0859
RbeOSU1250
RbeRML0514
Reis0944
Reis3 p38 Reis3 p38 traH familyl
Rra2 p079-81
Plasmids
Chromosomes
Plasmids
88
98
95
77
97
100
84
87
100
93
100
62
99
94
67
100
100
85
60
86
99
100
0.5

Figure A41. Neighbor-joining (NJ) and maximum likelihood (ML) trees of conjugative transfer protein containing TraN domain. Bootstrap supports higher than or equal to 60% are shown on the branches.

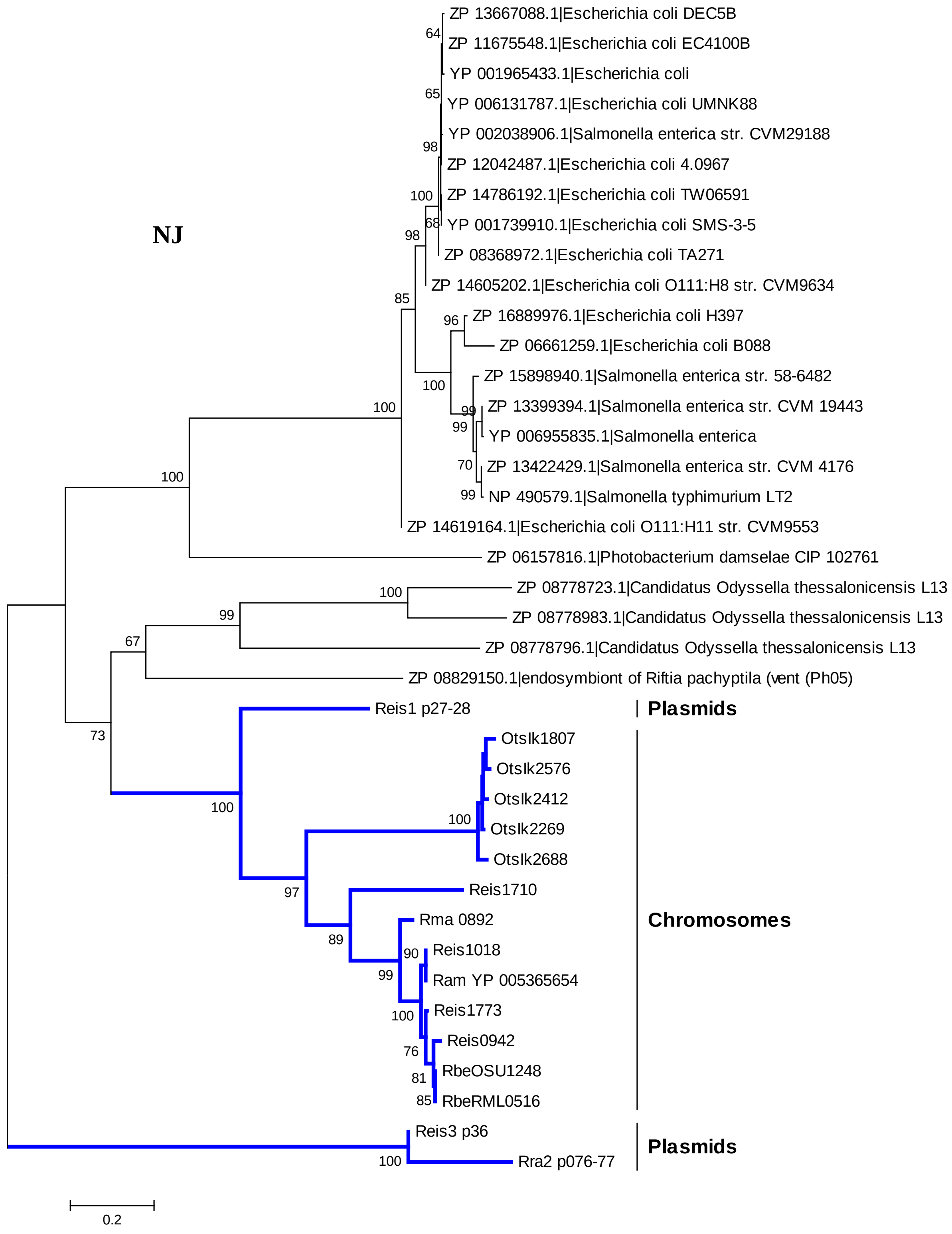

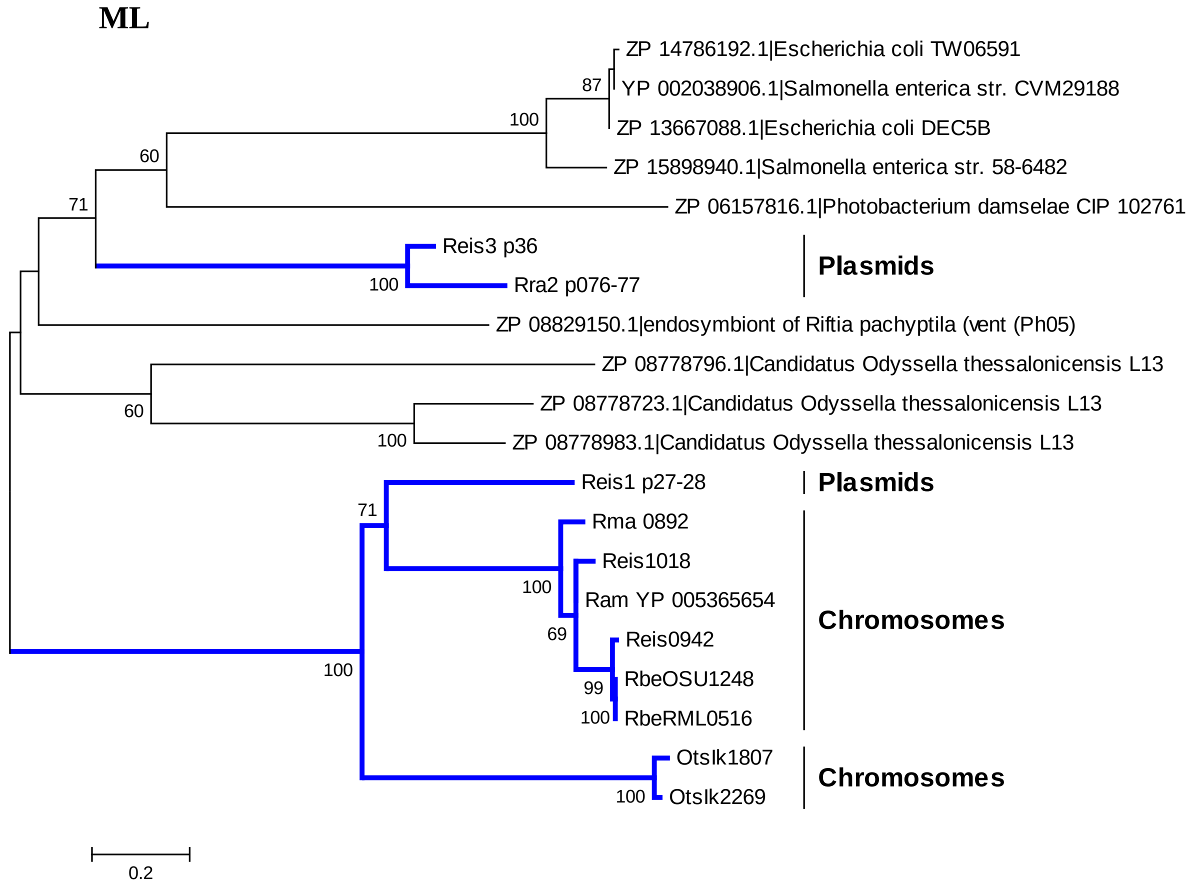

ML
ZP 14786192.1|Escherichia coli TW06591
YP 002038906.1|Salmonella enterica str. CVM29188
ZP 13667088.1|Escherichia coli DEC5B
ZP 15898940.1|Salmonella enterica str. 58-6482
ZP 06157816.1|Photobacterium damselae CIP 102761
Reis3 p36
Rra2 p076-77
Plasmids
ZP 08829150.1|endosymbiont of Riftia pachyptila (vent (Ph05)
ZP 08778796.1|Candidatus Odyssella thessalonicensis L13
ZP 08778723.1|Candidatus Odyssella thessalonicensis L13
ZP 08778983.1|Candidatus Odyssella thessalonicensis L13
Reis1 p27-28
Plasmids
Rma 0892
Reis1018
Ram YP 005365654
Reis0942
RbeOSU1248
RbeRML0516
Chromosomes
OtsIk1807
OtsIk2269
Chromosomes
87
100
60
71
100
60
100
71
100
69
99
100
100
100
0.2

Figure A42. Neighbor-joining (NJ) and maximum likelihood (ML) trees of conjugative transfer protein containing TrbC_Ftype domain. Bootstrap supports higher than or equal to 60% are shown on the branches.

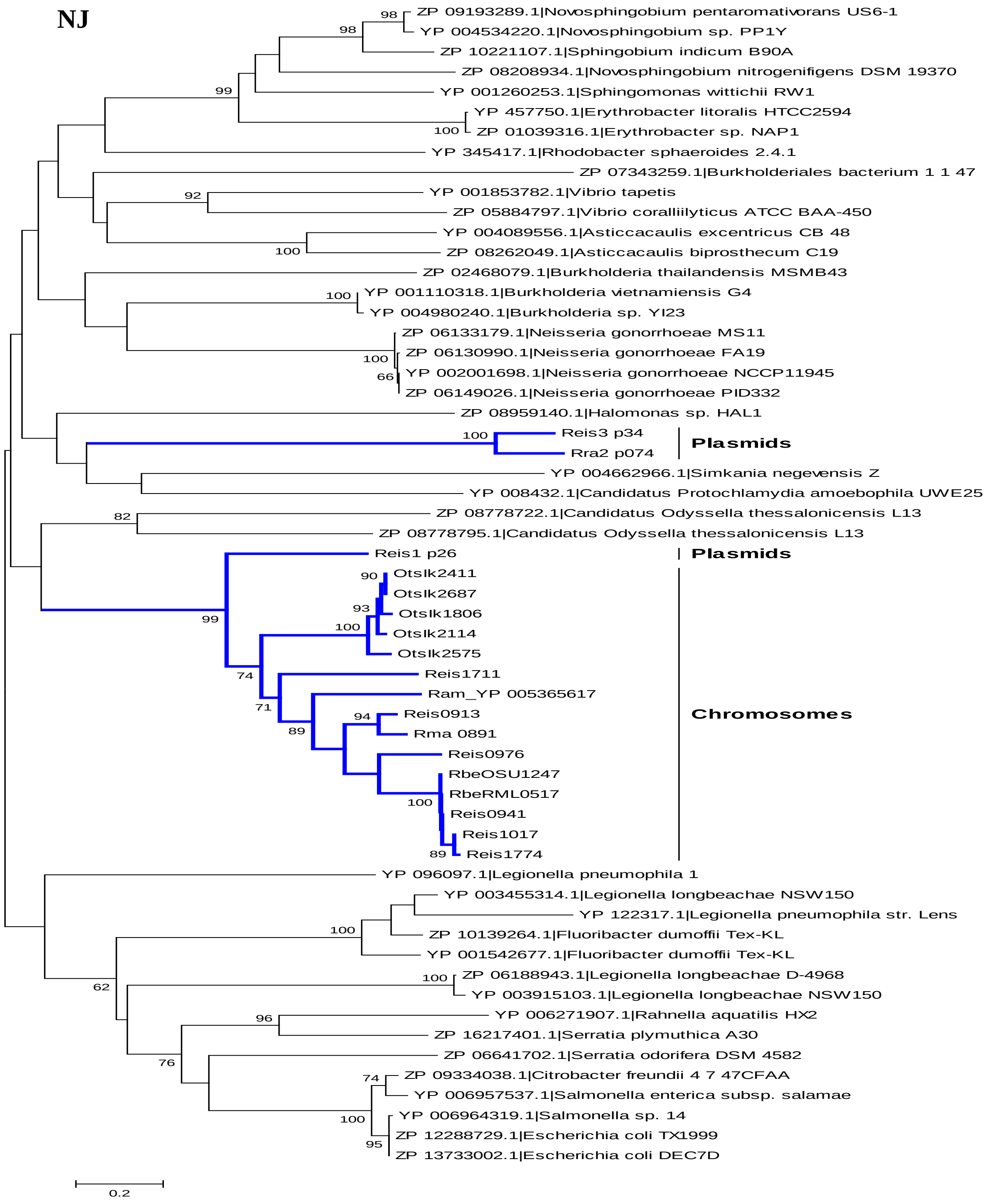

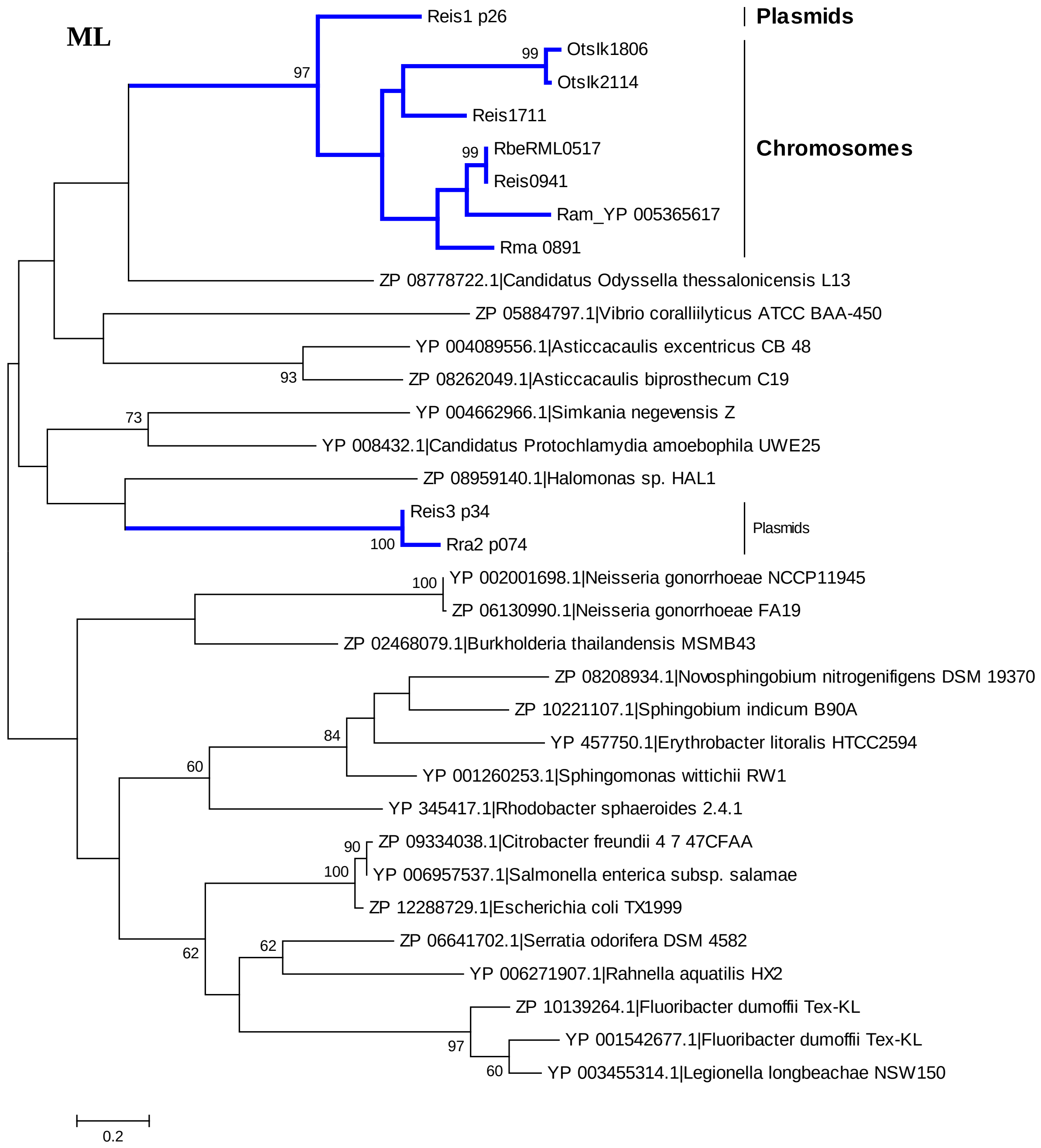
ML
Reis1 p26
Plasmids
OtsIk1806
OtsIk2114
Reis1711
RbeRML0517
Reis0941
Ram_YP 005365617
Rma 0891
Chromosomes
ZP 08778722.1|Candidatus Odyssella thessalonicensis L13
ZP 05884797.1|Vibrio coralliilyticus ATCC BAA-450
YP 004089556.1|Asticcacaulis excentricus CB 48
ZP 08262049.1|Asticcacaulis biprosthecum C19
YP 004662966.1|Simkania negevensis Z
YP 008432.1|Candidatus Protochlamydia amoebophila UWE25
ZP 08959140.1|Halomonas sp. HAL1
Reis3 p34
Rra2 p074
Plasmids
YP 002001698.1|Neisseria gonorrhoeae NCCP11945
ZP 06130990.1|Neisseria gonorrhoeae FA19
ZP 02468079.1|Burkholderia thailandensis MSMB43
ZP 08208934.1|Novosphingobium nitrogenifigens DSM 19370
ZP 10221107.1|Sphingobium indicum B90A
YP 457750.1|Erythrobacter litoralis HTCC2594
YP 001260253.1|Sphingomonas wittichii RW1
YP 345417.1|Rhodobacter sphaeroides 2.4.1
ZP 09334038.1|Citrobacter freundii 4 7 47CFAA
YP 006957537.1|Salmonella enterica subsp. salamae
ZP 12288729.1|Escherichia coli TX1999
ZP 06641702.1|Serratia odorifera DSM 4582
YP 006271907.1|Rahnella aquatilis HX2
ZP 10139264.1|Fluoribacter dumoffii Tex-KL
YP 001542677.1|Fluoribacter dumoffii Tex-KL
YP 003455314.1|Legionella longbeachae NSW150
97
99
99
93
73
100
100
84
60
90
100
62
62
97
60
0.2

Figure A43. Neighbor-joining (NJ) and maximum likelihood (ML) trees of conjugative transfer protein containing TraU domain. Bootstrap supports higher than or equal to 60% are shown on the branches.

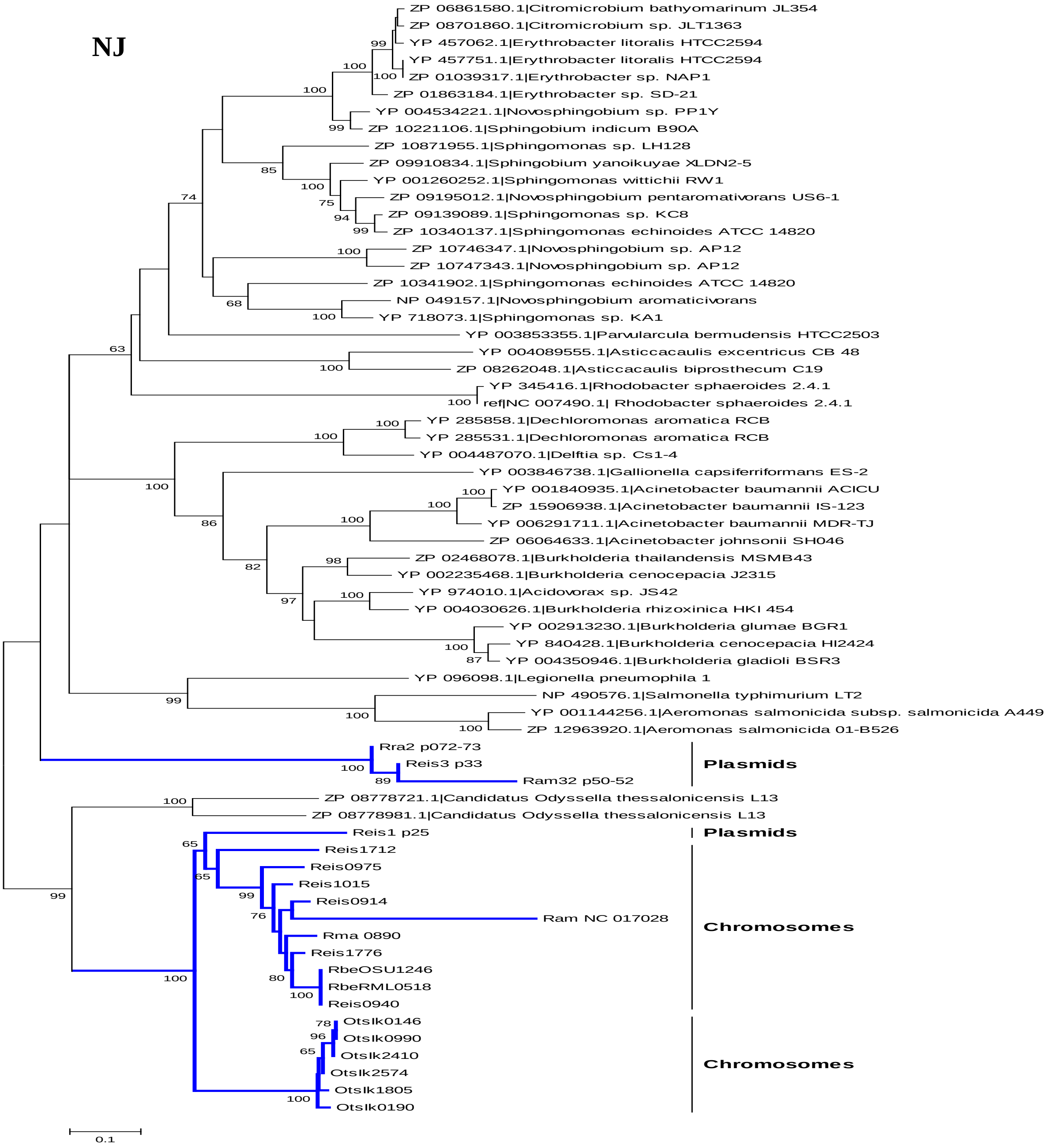

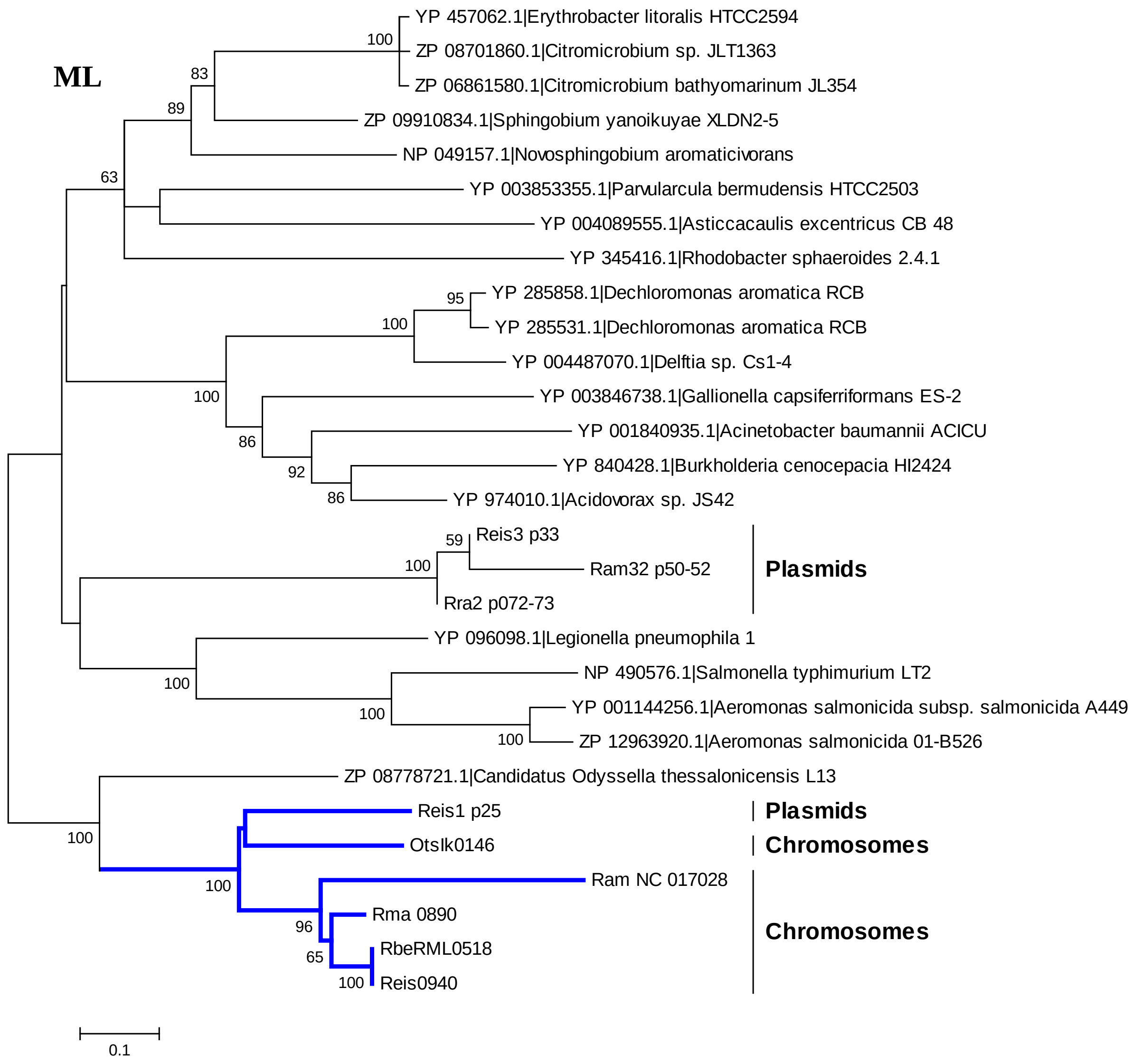

ML
YP 457062.1|Erythrobacter litoralis HTCC2594
ZP 08701860.1|Citromicrobium sp. JLT1363
ZP 06861580.1|Citromicrobium bathyomarinum JL354
ZP 09910834.1|Sphingobium yanoikuyae XLDN2-5
NP 049157.1|Novosphingobium aromaticivorans
YP 003853355.1|Parvularcula bermudensis HTCC2503
YP 004089555.1|Asticcacaulis excentricus CB 48
YP 345416.1|Rhodobacter sphaeroides 2.4.1
YP 285858.1|Dechloromonas aromatica RCB
YP 285531.1|Dechloromonas aromatica RCB
YP 004487070.1|Delftia sp. Cs1-4
YP 003846738.1|Gallionella capsiferriformans ES-2
YP 001840935.1|Acinetobacter baumannii ACICU
YP 840428.1|Burkholderia cenocepacia HI2424
YP 974010.1|Acidovorax sp. JS42
Reis3 p33
Ram32 p50-52
Rra2 p072-73
Plasmids
YP 096098.1|Legionella pneumophila 1
NP 490576.1|Salmonella typhimurium LT2
YP 001144256.1|Aeromonas salmonicida subsp. salmonicida A449
ZP 12963920.1|Aeromonas salmonicida 01-B526
ZP 08778721.1|Candidatus Odyssella thessalonicensis L13
Reis1 p25
Plasmids
OtsIk0146
Chromosomes
Ram NC 017028
Rma 0890
RbeRML0518
Reis0940
Chromosomes
100
83
89
63
95
100
100
86
92
86
59
100
100
100
100
100
100
96
65
100
0.1

Figure A44. Neighbor-joining (NJ) and maximum likelihood (ML) trees of conjugative transfer protein containing TraC_F_IV and AAA_10 domains. Bootstrap supports higher than or equal to 60% are shown on the branches.

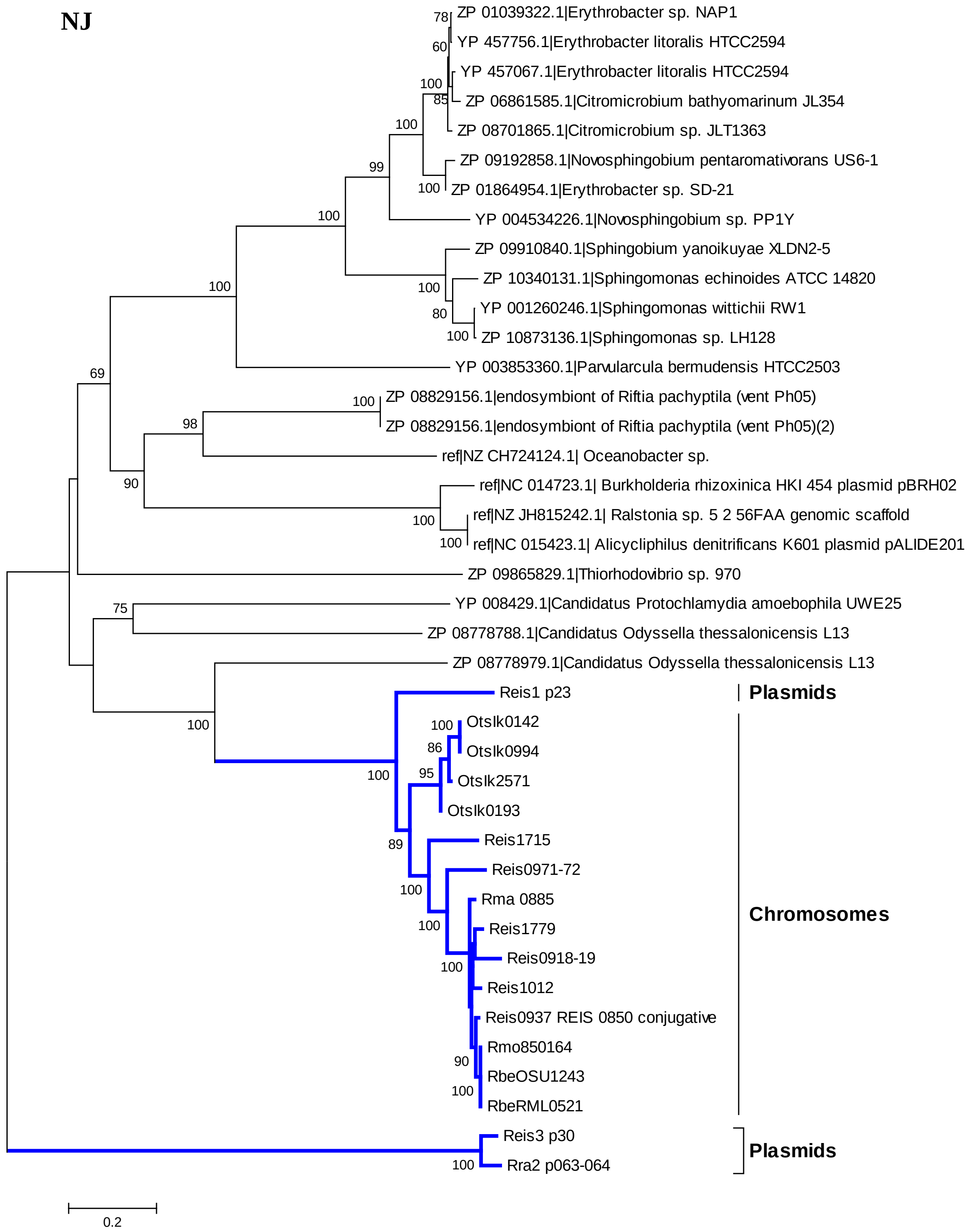

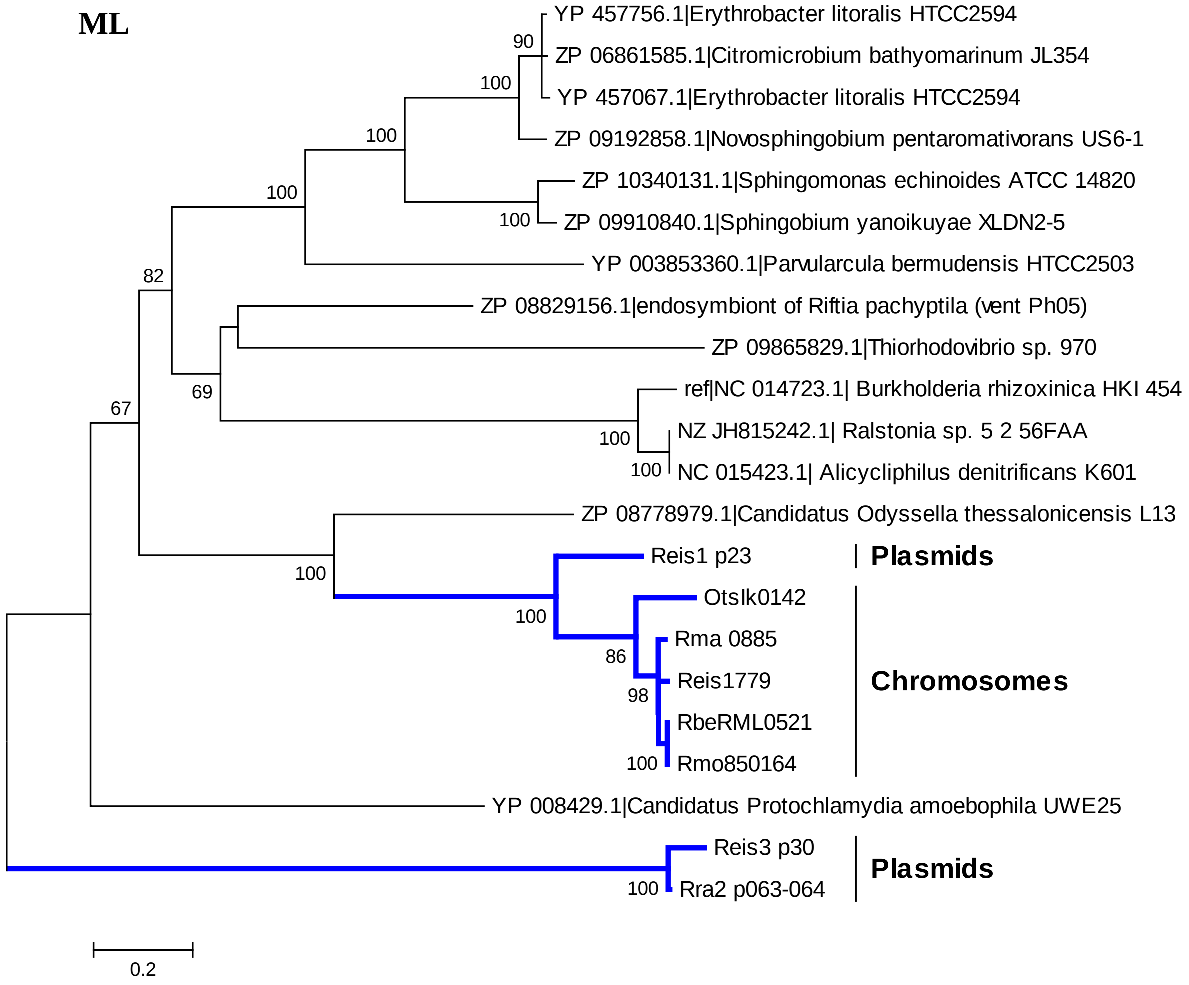
ML
YP 457756.1|Erythrobacter litoralis HTCC2594
ZP 06861585.1|Citromicrobium bathyomarinum JL354
YP 457067.1|Erythrobacter litoralis HTCC2594
ZP 09192858.1|Novosphingobium pentaromativorans US6-1
ZP 10340131.1|Sphingomonas echinoides ATCC 14820
ZP 09910840.1|Sphingobium yanoikuyae XLDN2-5
YP 003853360.1|Parvularcula bermudensis HTCC2503
ZP 08829156.1|endosymbiont of Riftia pachyptila (vent Ph05)
ZP 09865829.1|Thiorhodovibrio sp. 970
ref|NC 014723.1| Burkholderia rhizoxinica HKI 454
NZ JH815242.1| Ralstonia sp. 5 2 56FAA
NC 015423.1| Alicycliphilus denitrificans K601
ZP 08778979.1|Candidatus Odyssella thessalonicensis L13
Reis1 p23
Plasmids
OtsIk0142
Rma 0885
Reis1779
Chromosomes
RbeRML0521
Rmo850164
YP 008429.1|Candidatus Protochlamydia amoebophila UWE25
Reis3 p30
Rra2 p063-064
Plasmids
90
100
100
100
100
82
69
67
100
100
100
100
86
98
100
100
0.2

Figure A45. Neighbor-joining (NJ) and maximum likelihood (ML) trees of conjugative transfer protein containing TraE domain. Bootstrap supports higher than or equal to 60% are shown on the branches.

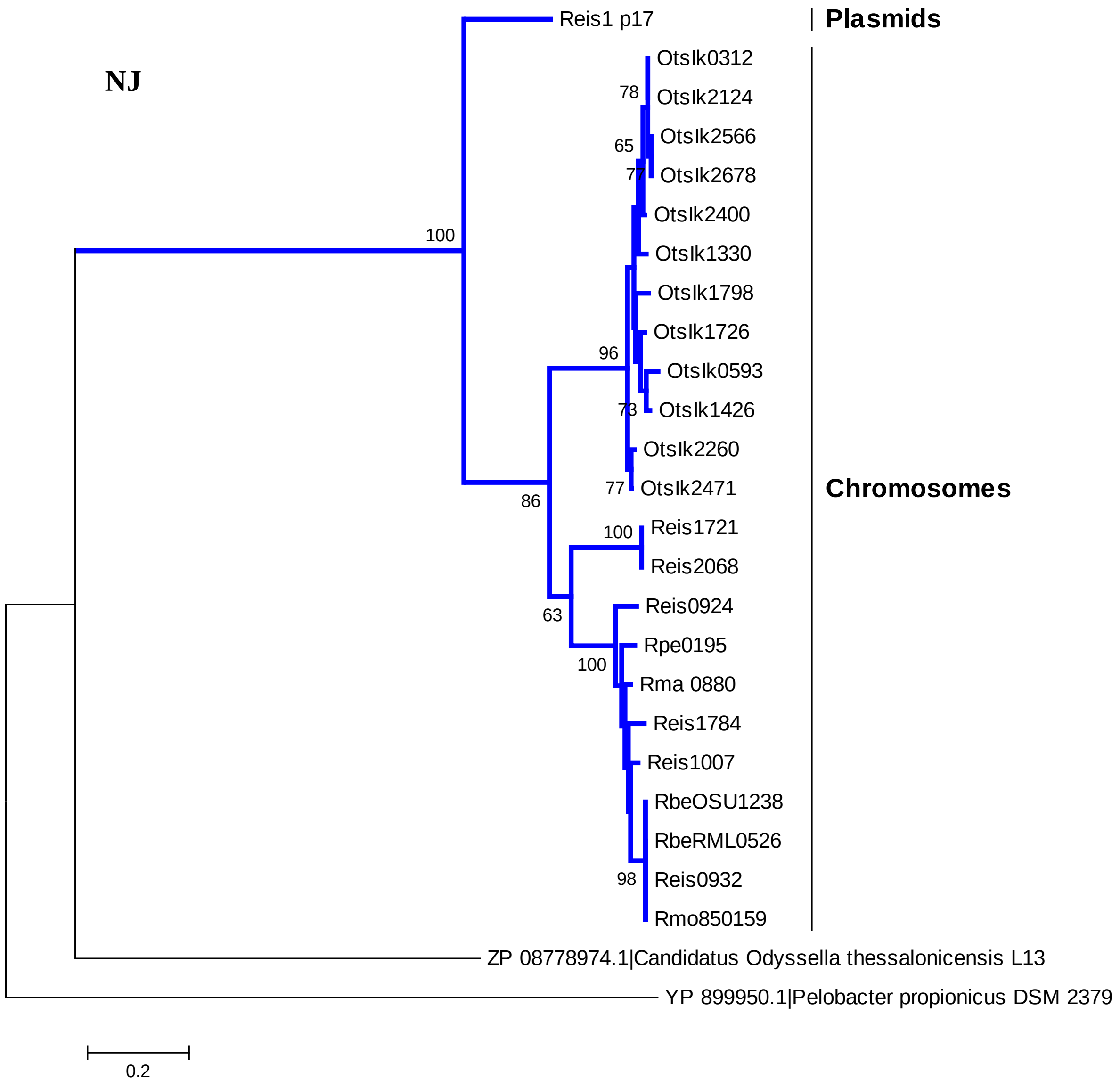

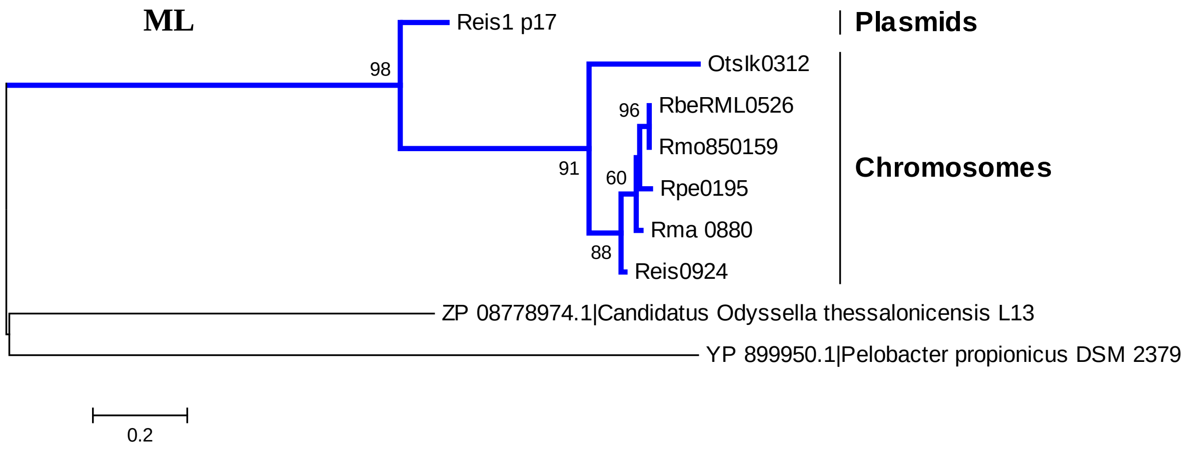
ML
Reis1 p17
Plasmids
98
OtsIk0312
96
RbeRML0526
Rmo850159
91
60
Rpe0195
Chromosomes
Rma 0880
88
Reis0924
ZP 08778974.1|Candidatus Odyssella thessalonicensis L13
YP 899950.1|Pelobacter propionicus DSM 2379
0.2

Figure A46. Neighbor-joining (NJ) and maximum likelihood (ML) trees of conjugative transfer protein containing TrbI domain. Bootstrap supports higher than or equal to 60% are shown on the branches.

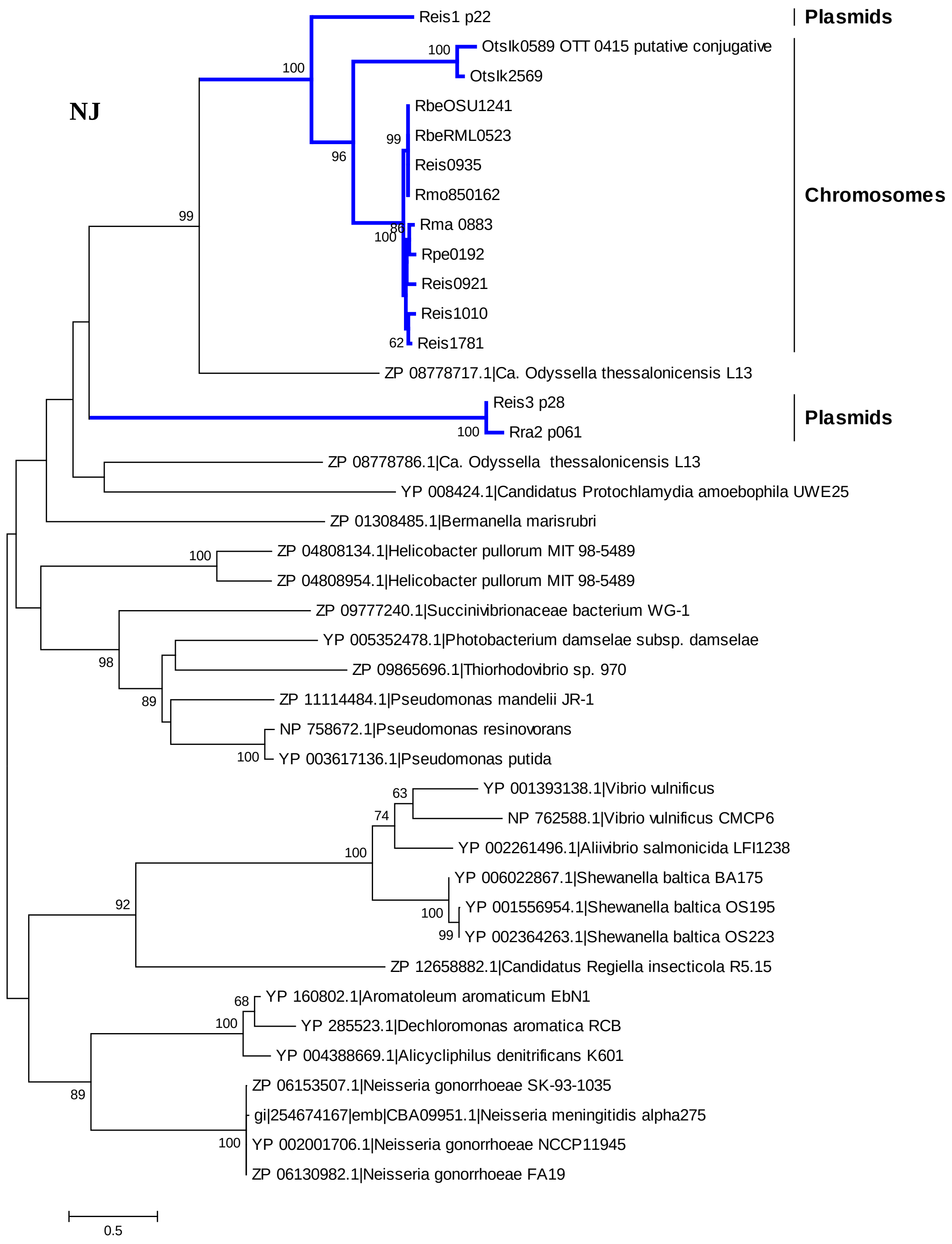

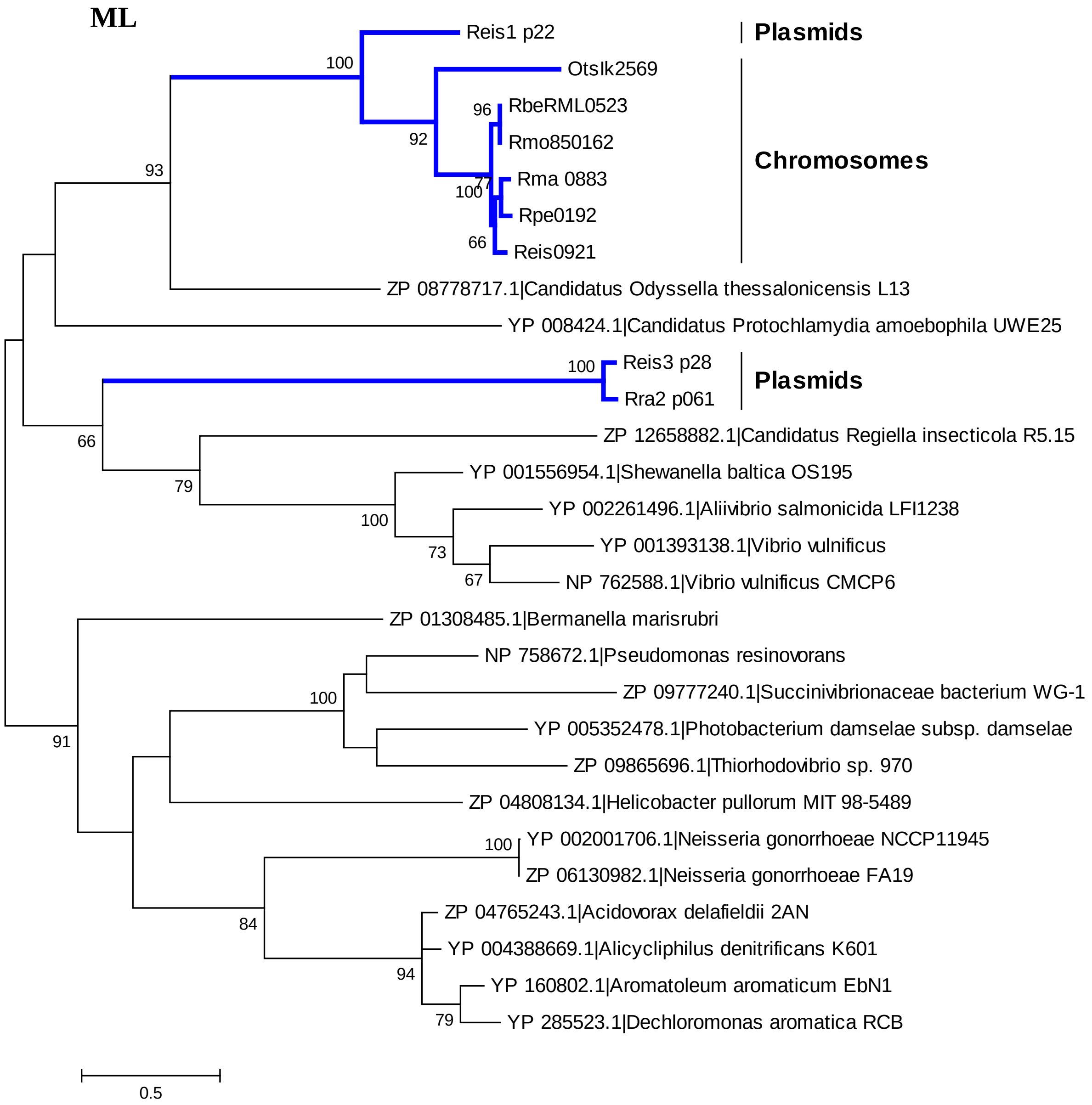

ML
Reis1 p22
Plasmids
OtsIk2569
RbeRML0523
Rmo850162
Chromosomes
Rma 0883
Rpe0192
Reis0921
ZP 08778717.1|Candidatus Odyssella thessalonicensis L13
YP 008424.1|Candidatus Protochlamydia amoebophila UWE25
Reis3 p28
Rra2 p061
Plasmids
ZP 12658882.1|Candidatus Regiella insecticola R5.15
YP 001556954.1|Shewanella baltica OS195
YP 002261496.1|Aliivibrio salmonicida LFI1238
YP 001393138.1|Vibrio vulnificus
NP 762588.1|Vibrio vulnificus CMCP6
ZP 01308485.1|Bermanella marisrubri
NP 758672.1|Pseudomonas resinovorans
ZP 09777240.1|Succinivibrionaceae bacterium WG-1
YP 005352478.1|Photobacterium damselae subsp. damselae
ZP 09865696.1|Thiorhodovibrio sp. 970
ZP 04808134.1|Helicobacter pullorum MIT 98-5489
YP 002001706.1|Neisseria gonorrhoeae NCCP11945
ZP 06130982.1|Neisseria gonorrhoeae FA19
ZP 04765243.1|Acidovorax delafieldii 2AN
YP 004388669.1|Alicycliphilus denitrificans K601
YP 160802.1|Aromatoleum aromaticum EbN1
YP 285523.1|Dechloromonas aromatica RCB
0.5

Figure A47. Neighbor-joining (NJ) and maximum likelihood (ML) trees of conjugative coupling protein containing TrwB and TraD_N domains. Bootstrap supports higher than or equal to 60% are shown on the branches.

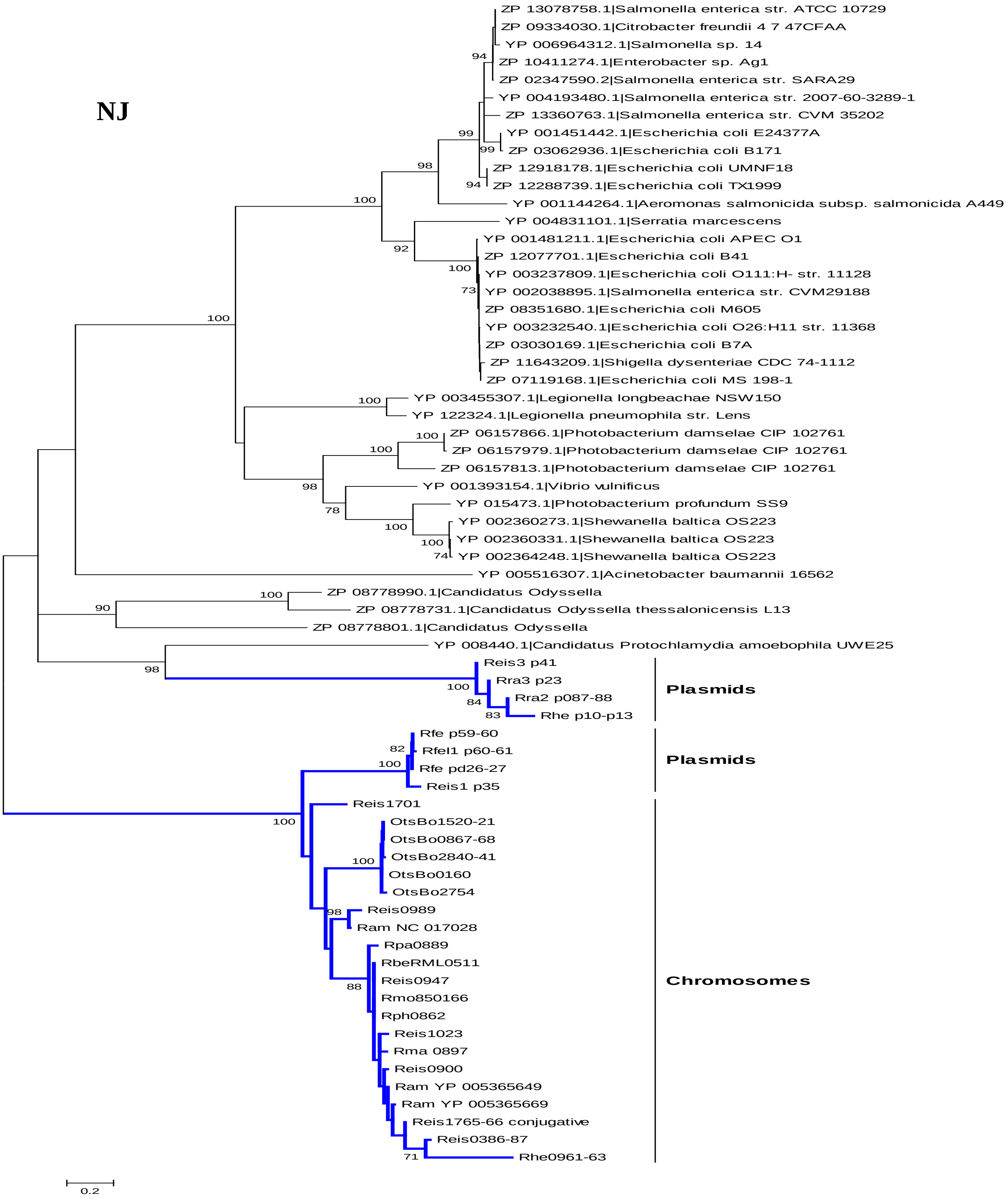

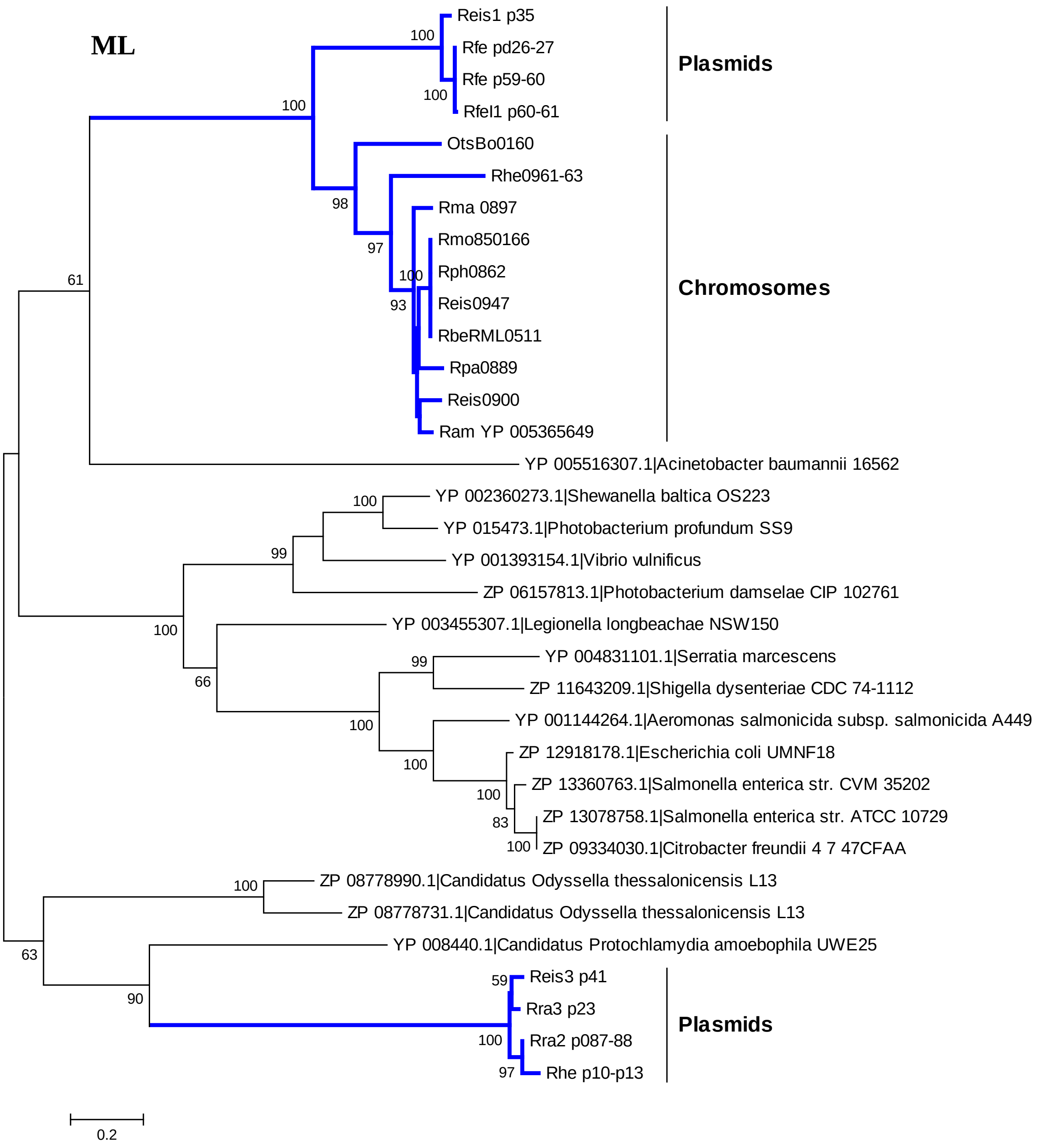
ML
Plasmids
Chromosomes
Plasmids
Reis1 p35
Rfe pd26-27
Rfe p59-60
Rfel1 p60-61
OtsBo0160
Rhe0961-63
Rma 0897
Rmo850166
Rph0862
Reis0947
RbeRML0511
Rpa0889
Reis0900
Ram YP 005365649
YP 005516307.1|Acinetobacter baumannii 16562
YP 002360273.1|Shewanella baltica OS223
YP 015473.1|Photobacterium profundum SS9
YP 001393154.1|Vibrio vulnificus
ZP 06157813.1|Photobacterium damselae CIP 102761
YP 003455307.1|Legionella longbeachae NSW150
YP 004831101.1|Serratia marcescens
ZP 11643209.1|Shigella dysenteriae CDC 74-1112
YP 001144264.1|Aeromonas salmonicida subsp. salmonicida A449
ZP 12918178.1|Escherichia coli UMNF18
ZP 13360763.1|Salmonella enterica str. CVM 35202
ZP 13078758.1|Salmonella enterica str. ATCC 10729
ZP 09334030.1|Citrobacter freundii 4 7 47CFAA
ZP 08778990.1|Candidatus Odyssella thessalonicensis L13
ZP 08778731.1|Candidatus Odyssella thessalonicensis L13
YP 008440.1|Candidatus Protochlamydia amoebophila UWE25
Reis3 p41
Rra3 p23
Rra2 p087-88
Rhe p10-p13
0.2

Figure A48. Neighbor-joining (NJ) and maximum likelihood (ML) trees of conjugative transfer protein containing TraF domain. Bootstrap supports higher than or equal to 60% are shown on the branches.

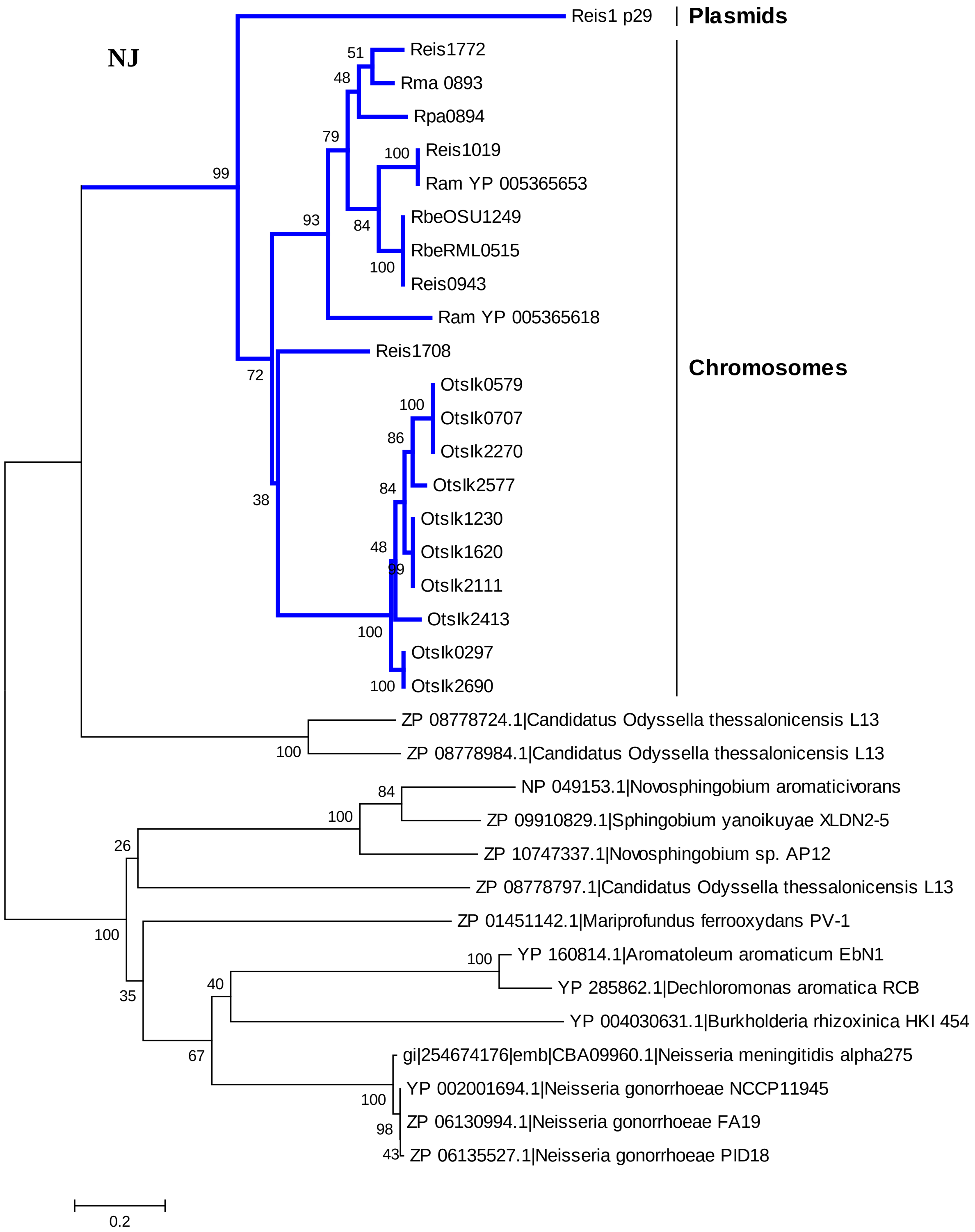

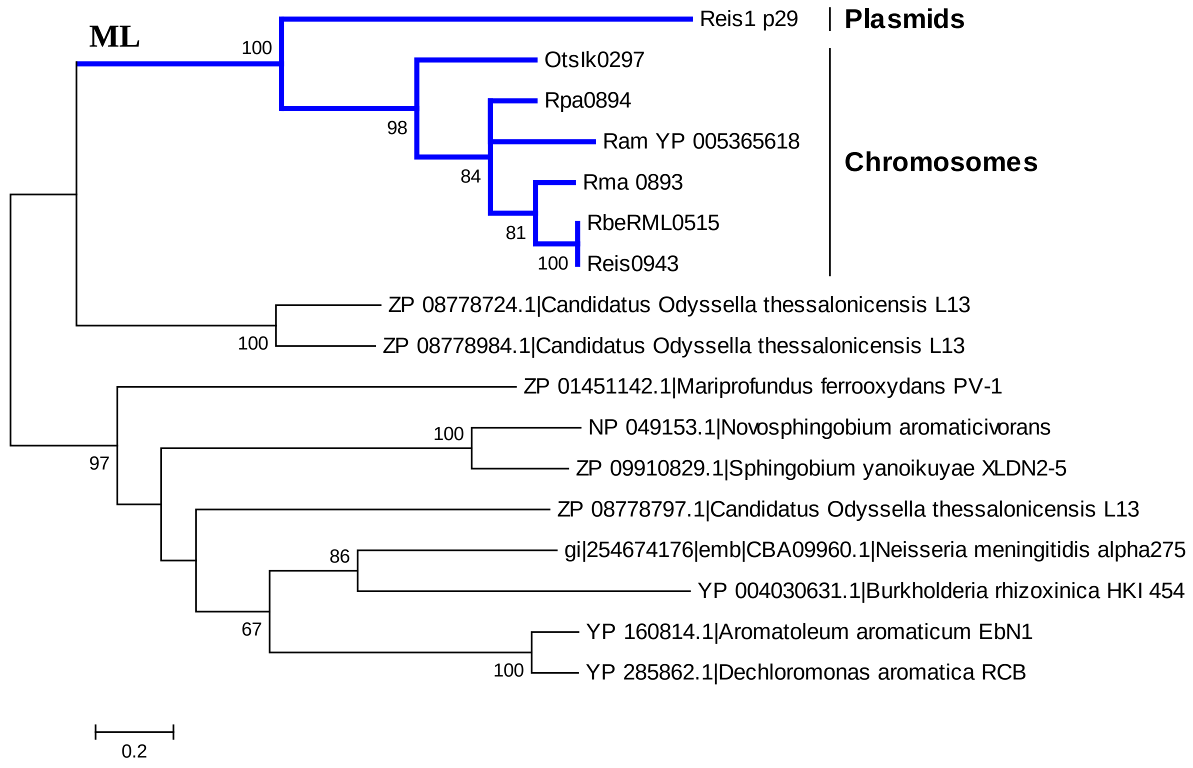

ML
Reis1 p29
Plasmids
OtsIk0297
Rpa0894
Ram YP 005365618
Chromosomes
Rma 0893
RbeRML0515
Reis0943
ZP 08778724.1|Candidatus Odyssella thessalonicensis L13
ZP 08778984.1|Candidatus Odyssella thessalonicensis L13
ZP 01451142.1|Mariprofundus ferrooxydans PV-1
NP 049153.1|Novosphingobium aromaticivorans
ZP 09910829.1|Sphingobium yanoikuyae XLDN2-5
ZP 08778797.1|Candidatus Odyssella thessalonicensis L13
gi|254674176|emb|CBA09960.1|Neisseria meningitidis alpha275
YP 004030631.1|Burkholderia rhizoxinica HKI 454
YP 160814.1|Aromatoleum aromaticum EbN1
YP 285862.1|Dechloromonas aromatica RCB
100
98
84
81
100
100
97
100
86
67
100
0.2

Figure A49. Neighbor-joining (NJ) and maximum likelihood (ML) trees of helicases RecD/TraA containing AAA_30 and UvrD_C_2 domains. Bootstrap supports higher than or equal to 60% are shown on the branches.

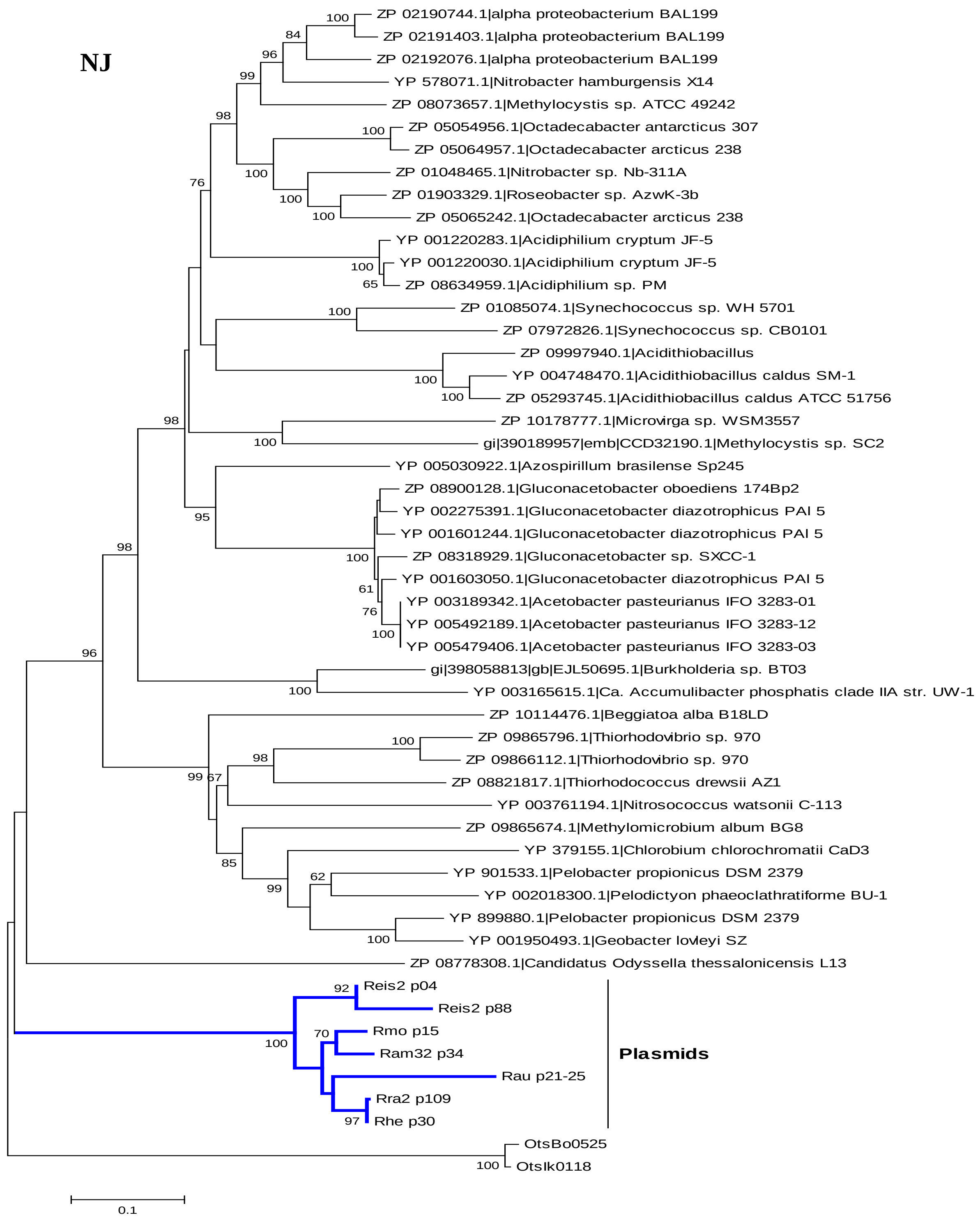

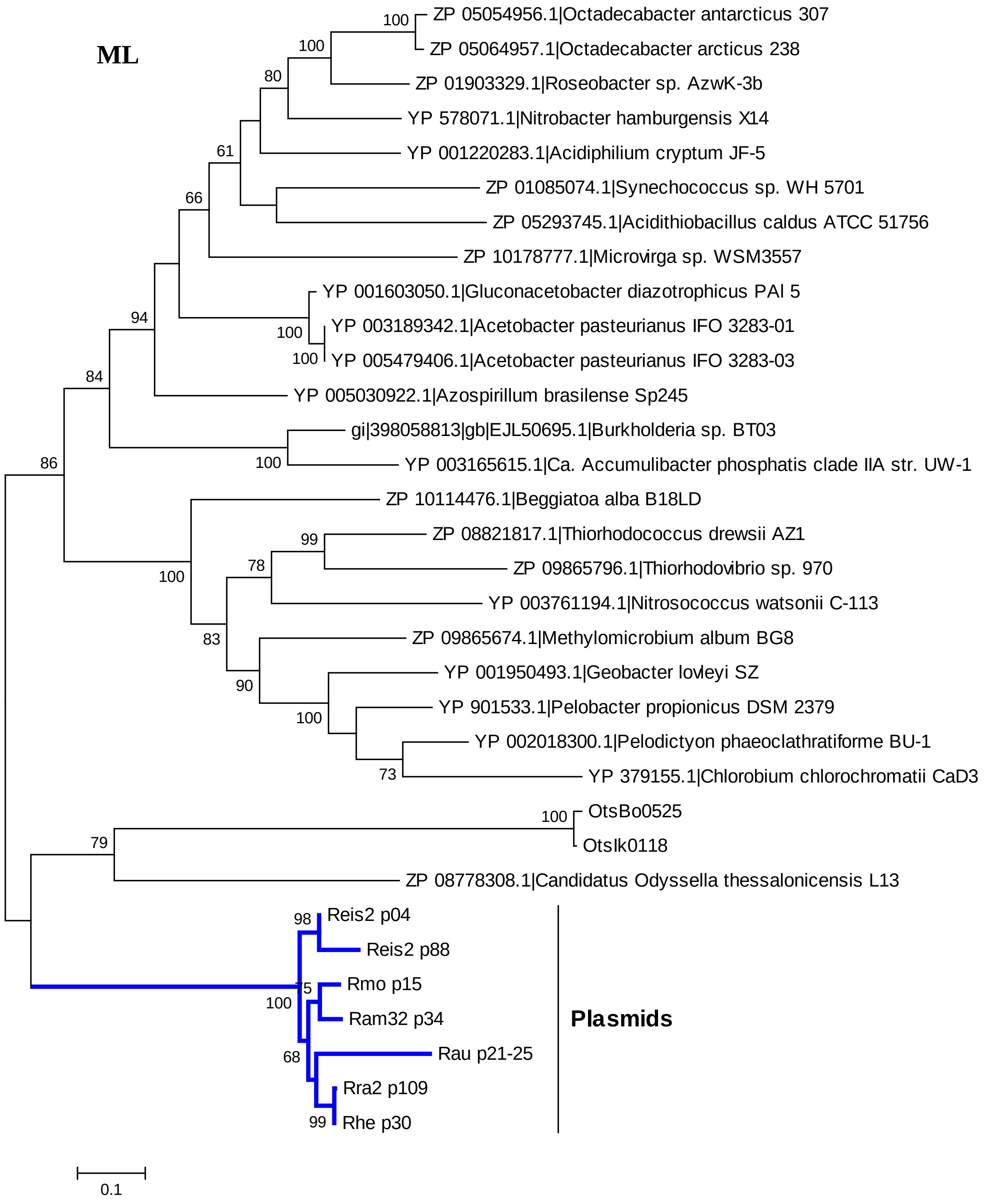

ML
ZP 05054956.1|Octadecabacter antarcticus 307
ZP 05064957.1|Octadecabacter arcticus 238
ZP 01903329.1|Roseobacter sp. AzwK-3b
YP 578071.1|Nitrobacter hamburgensis X14
YP 001220283.1|Acidiphilium cryptum JF-5
ZP 01085074.1|Synechococcus sp. WH 5701
ZP 05293745.1|Acidithiobacillus caldus ATCC 51756
ZP 10178777.1|Microvirga sp. WSM3557
YP 001603050.1|Gluconacetobacter diazotrophicus PAl 5
YP 003189342.1|Acetobacter pasteurianus IFO 3283-01
YP 005479406.1|Acetobacter pasteurianus IFO 3283-03
YP 005030922.1|Azospirillum brasilense Sp245
gi|398058813|gb|EJL50695.1|Burkholderia sp. BT03
YP 003165615.1|Ca. Accumulibacter phosphatis clade IIA str. UW-1
ZP 10114476.1|Beggiatoa alba B18LD
ZP 08821817.1|Thiorhodococcus drewsii AZ1
ZP 09865796.1|Thiorhodovibrio sp. 970
YP 003761194.1|Nitrosococcus watsonii C-113
ZP 09865674.1|Methylomicrobium album BG8
YP 001950493.1|Geobacter lovleyi SZ
YP 901533.1|Pelobacter propionicus DSM 2379
YP 002018300.1|Pelodictyon phaeoclathratiforme BU-1
YP 379155.1|Chlorobium chlorochromatii CaD3
OtsBo0525
OtsIk0118
ZP 08778308.1|Candidatus Odyssella thessalonicensis L13
Reis2 p04
Reis2 p88
Rmo p15
Ram32 p34
Rau p21-25
Rra2 p109
Rhe p30
Plasmids
100
100
80
61
66
94
84
86
100
100
100
99
78
100
83
90
100
73
100
79
98
75
100
68
99
0.1

Figure A50. Neighbor-joining (NJ) and maximum likelihood (ML) trees of conjugative transfer relaxase TraA_Ti containing MobA/MobL and AAA_30 domains. Bootstrap supports higher than or equal to 60% are shown on the branches.

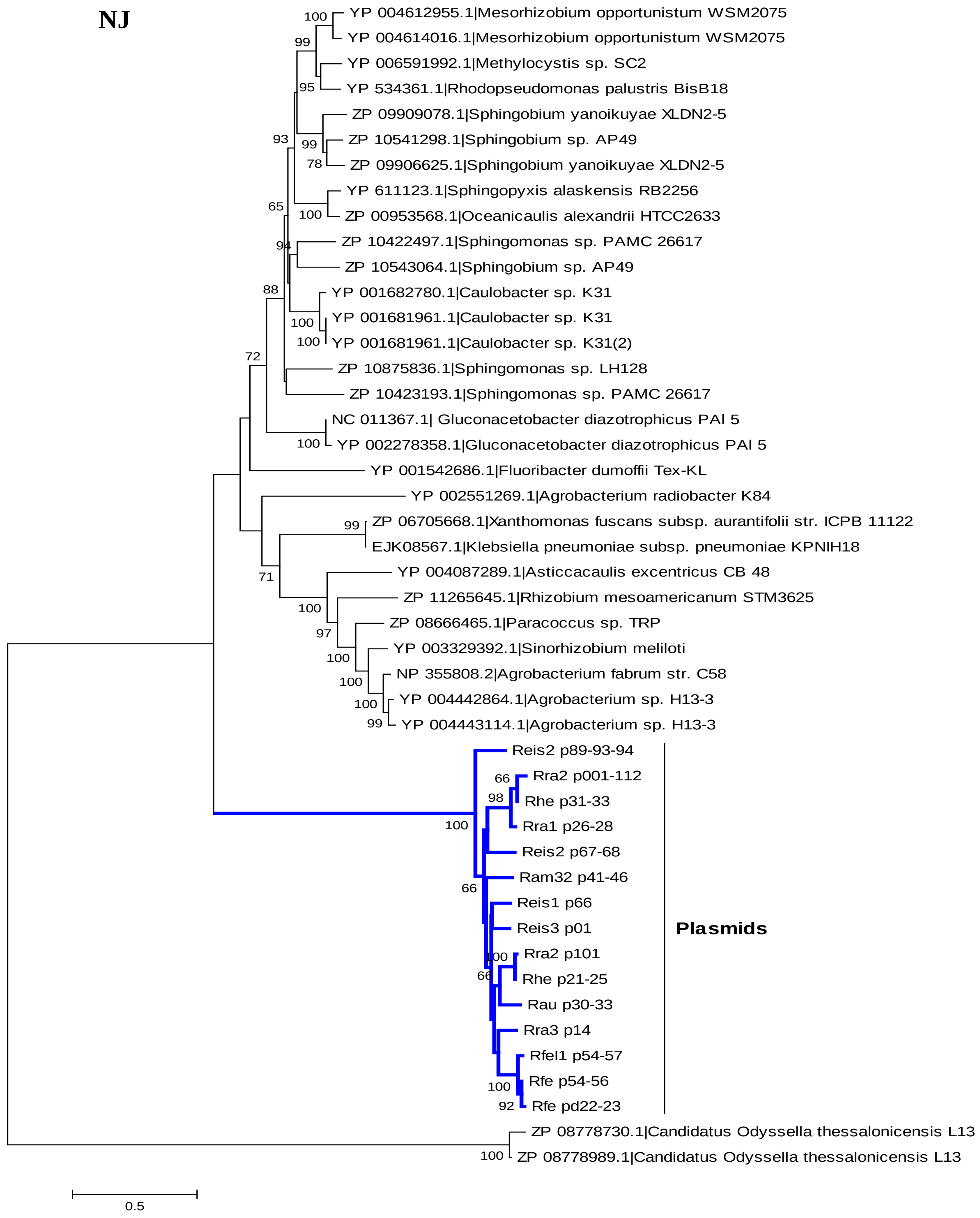

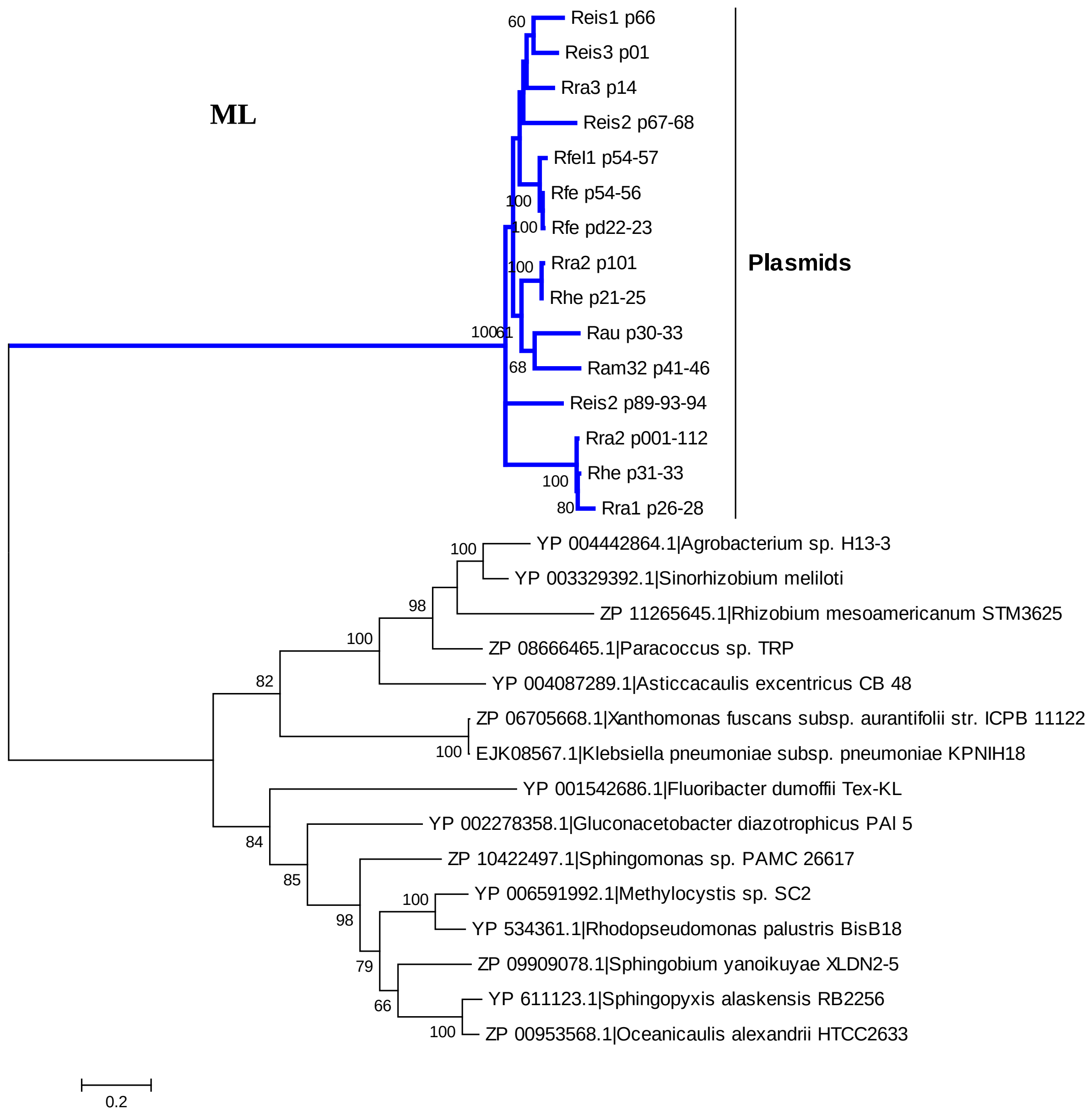
ML
Plasmids
Reis1 p66
Reis3 p01
Rra3 p14
Reis2 p67-68
Rfel1 p54-57
Rfe p54-56
Rfe pd22-23
Rra2 p101
Rhe p21-25
Rau p30-33
Ram32 p41-46
Reis2 p89-93-94
Rra2 p001-112
Rhe p31-33
Rra1 p26-28
YP 004442864.1|Agrobacterium sp. H13-3
YP 003329392.1|Sinorhizobium meliloti
ZP 11265645.1|Rhizobium mesoamericanum STM3625
ZP 08666465.1|Paracoccus sp. TRP
YP 004087289.1|Asticcacaulis excentricus CB 48
ZP 06705668.1|Xanthomonas fuscans subsp. aurantifolii str. ICPB 11122
EJK08567.1|Klebsiella pneumoniae subsp. pneumoniae KPNIH18
YP 001542686.1|Fluoribacter dumoffii Tex-KL
YP 002278358.1|Gluconacetobacter diazotrophicus PAl 5
ZP 10422497.1|Sphingomonas sp. PAMC 26617
YP 006591992.1|Methylocystis sp. SC2
YP 534361.1|Rhodopseudomonas palustris BisB18
ZP 09909078.1|Sphingobium yanoikuyae XLDN2-5
YP 611123.1|Sphingopyxis alaskensis RB2256
ZP 00953568.1|Oceanicaulis alexandrii HTCC2633
0.2

Figure A51. Neighbor-joining (NJ) and maximum likelihood (ML) trees of topoisomerase. Bootstrap supports higher than or equal to 60% are shown on the branches.

NJ

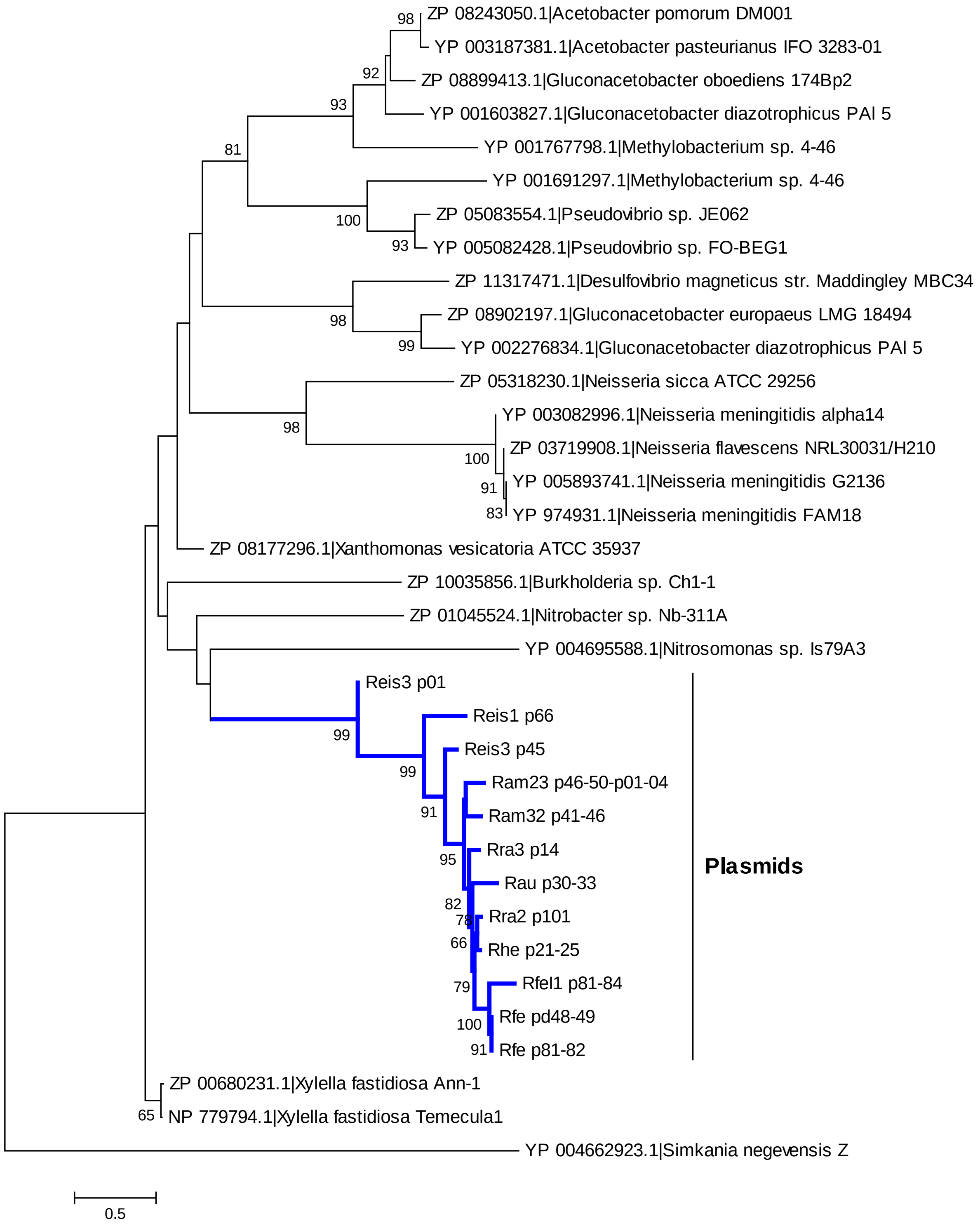

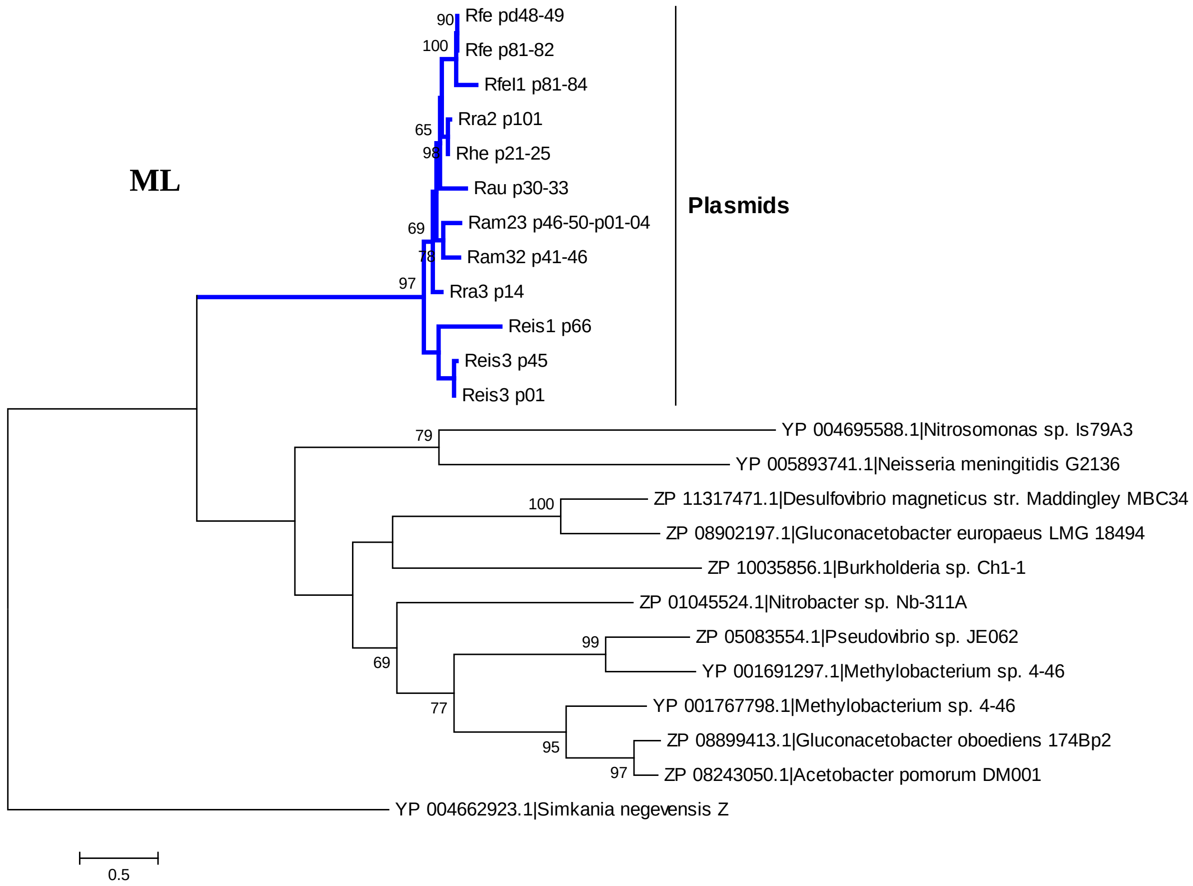
ML
Plasmids
Rfe pd48-49
Rfe p81-82
Rfel1 p81-84
Rra2 p101
Rhe p21-25
Rau p30-33
Ram23 p46-50-p01-04
Ram32 p41-46
Rra3 p14
Reis1 p66
Reis3 p45
Reis3 p01
YP 004695588.1|Nitrosomonas sp. Is79A3
YP 005893741.1|Neisseria meningitidis G2136
ZP 11317471.1|Desulfovibrio magneticus str. Maddingley MBC34
ZP 08902197.1|Gluconacetobacter europaeus LMG 18494
ZP 10035856.1|Burkholderia sp. Ch1-1
ZP 01045524.1|Nitrobacter sp. Nb-311A
ZP 05083554.1|Pseudovibrio sp. JE062
YP 001691297.1|Methylobacterium sp. 4-46
YP 001767798.1|Methylobacterium sp. 4-46
ZP 08899413.1|Gluconacetobacter oboediens 174Bp2
ZP 08243050.1|Acetobacter pomorum DM001
YP 004662923.1|Simkania negevensis Z
0.5

Figure A52. Neighbor-joining (NJ) and maximum likelihood (ML) trees of WGR domain-containing protein. Bootstrap supports higher than or equal to 60% are shown on the branches.

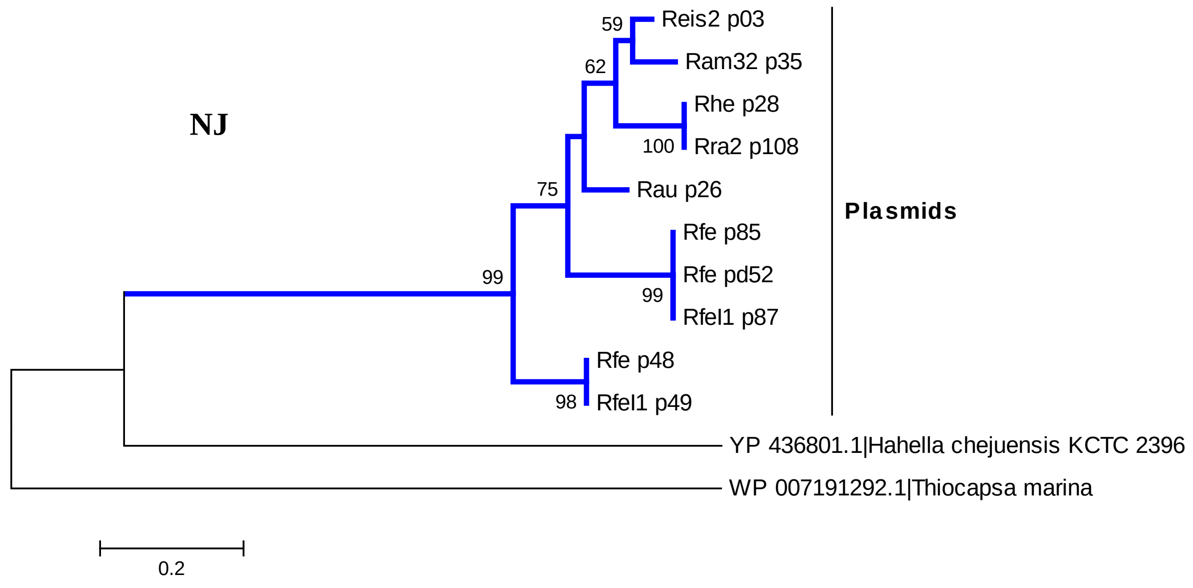

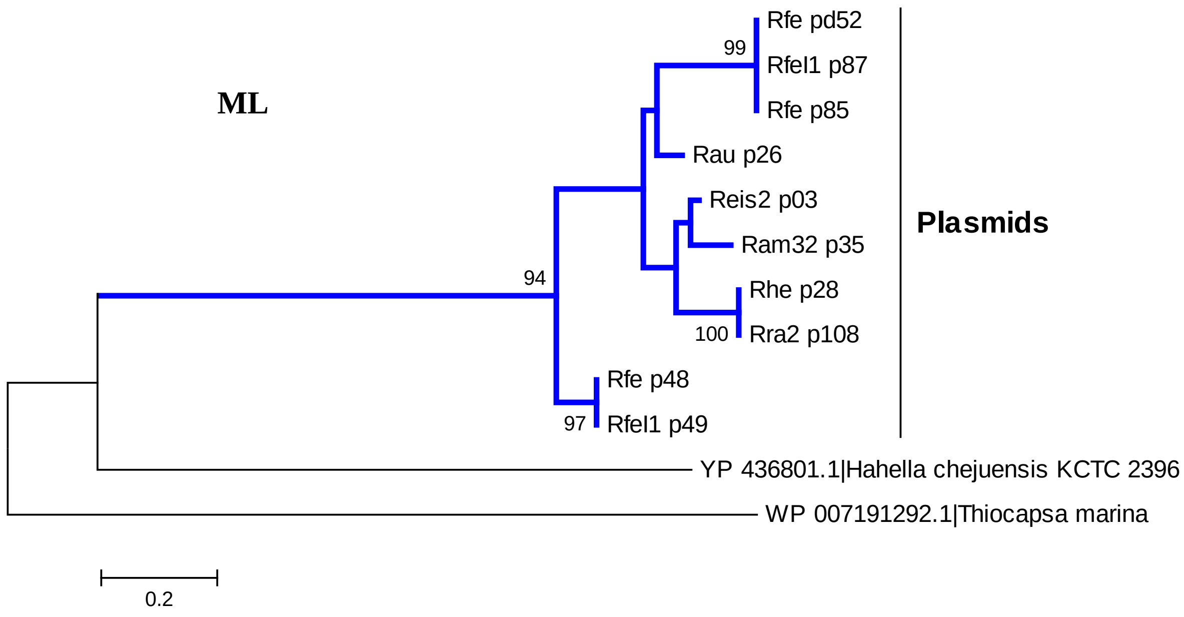
ML
Rfe pd52
99
Rfel1 p87
Rfe p85
Rau p26
Reis2 p03
Ram32 p35
Plasmids
94
Rhe p28
100
Rra2 p108
Rfe p48
97
Rfel1 p49
YP 436801.1|Hahella chejuensis KCTC 2396
WP 007191292.1|Thiocapsa marina
0.2

Figure A53. Neighbor-joining (NJ) and maximum likelihood (ML) trees of WGR domain-containing protein. Bootstrap supports higher than or equal to 60% are shown on the branches.

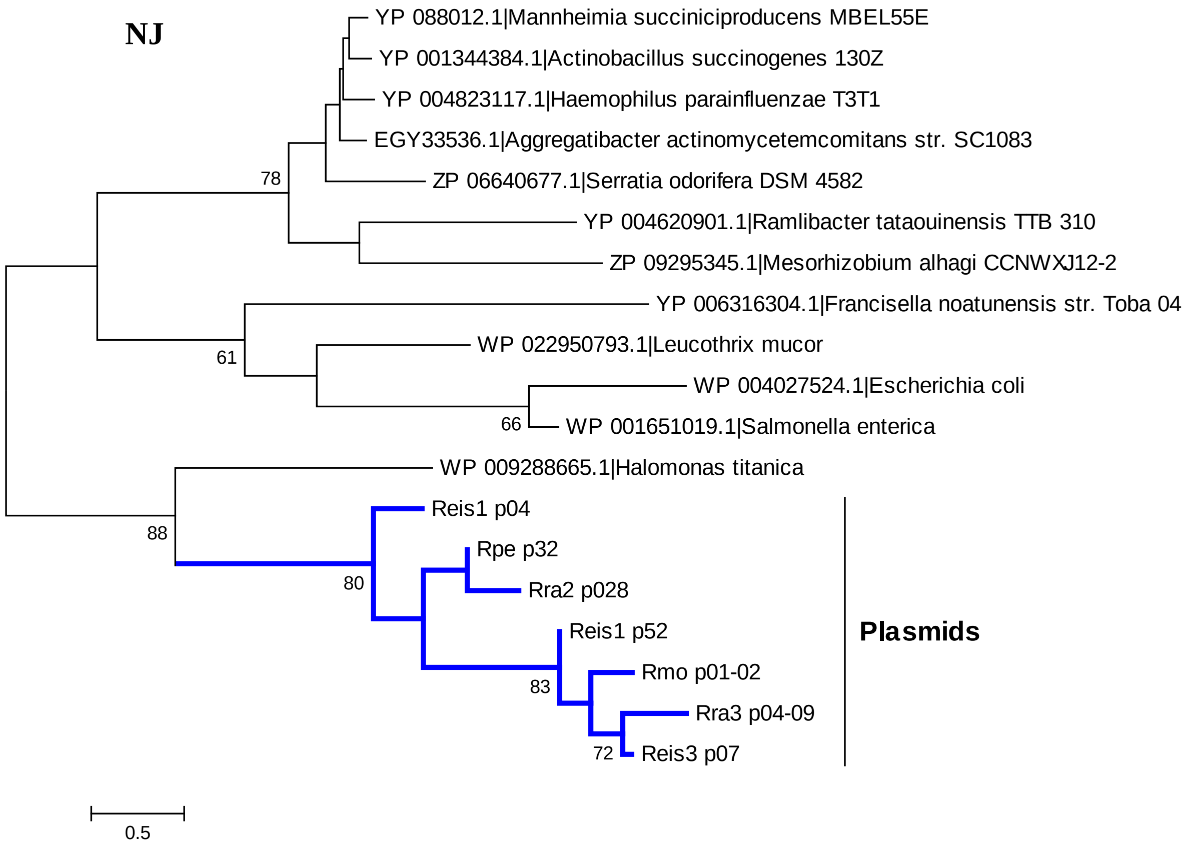

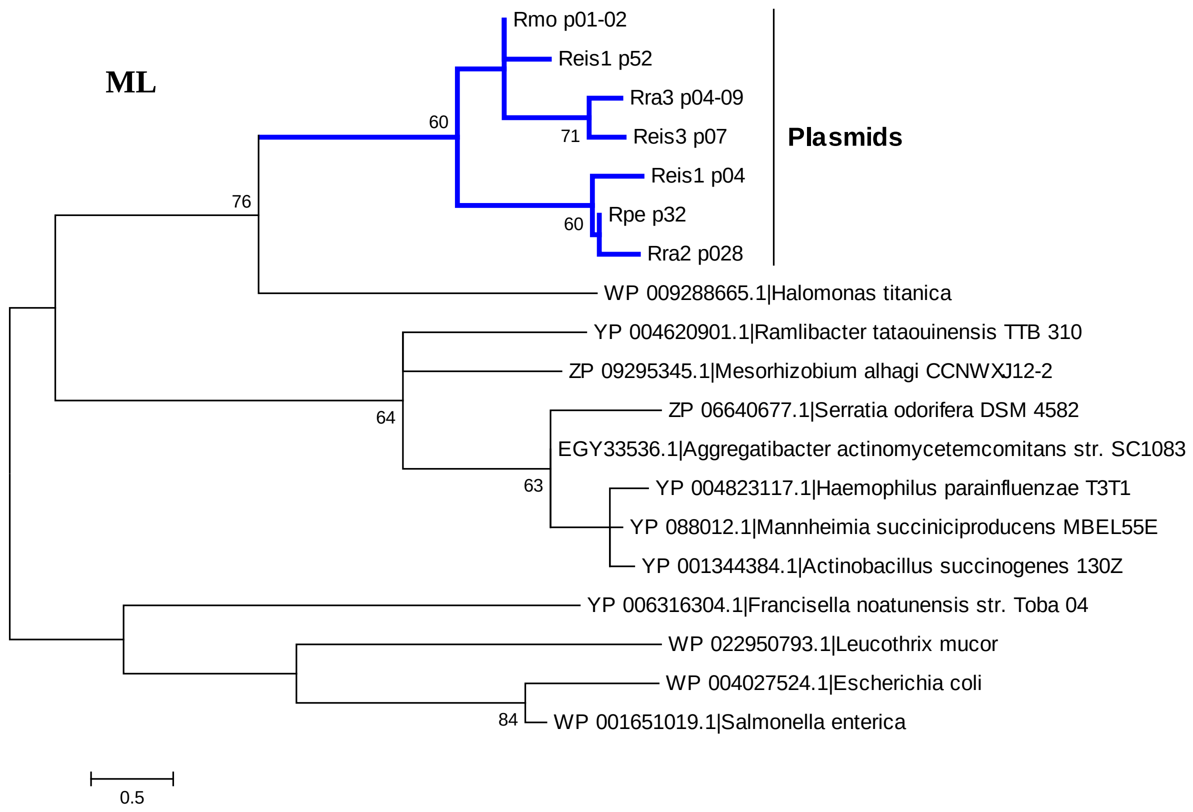
ML
Rmo p01-02
Reis1 p52
Rra3 p04-09
Reis3 p07
Reis1 p04
Rpe p32
Rra2 p028
Plasmids
60
71
76
60
WP 009288665.1|Halomonas titanica
YP 004620901.1|Ramlibacter tataouinensis TTB 310
ZP 09295345.1|Mesorhizobium alhagi CCNWXJ12-2
64
ZP 06640677.1|Serratia odorifera DSM 4582
EGY33536.1|Aggregatibacter actinomycetemcomitans str. SC1083
63
YP 004823117.1|Haemophilus parainfluenzae T3T1
YP 088012.1|Mannheimia succiniciproducens MBEL55E
YP 001344384.1|Actinobacillus succinogenes 130Z
YP 006316304.1|Francisella noatunensis str. Toba 04
WP 022950793.1|Leucothrix mucor
WP 004027524.1|Escherichia coli
84
WP 001651019.1|Salmonella enterica
0.5

Figure A54. Neighbor-joining (NJ) and maximum likelihood (ML) trees of transposase/integrase containing HTH_21 and rve domains. Bootstrap supports higher than or equal to 60% are shown on the branches.

NJ

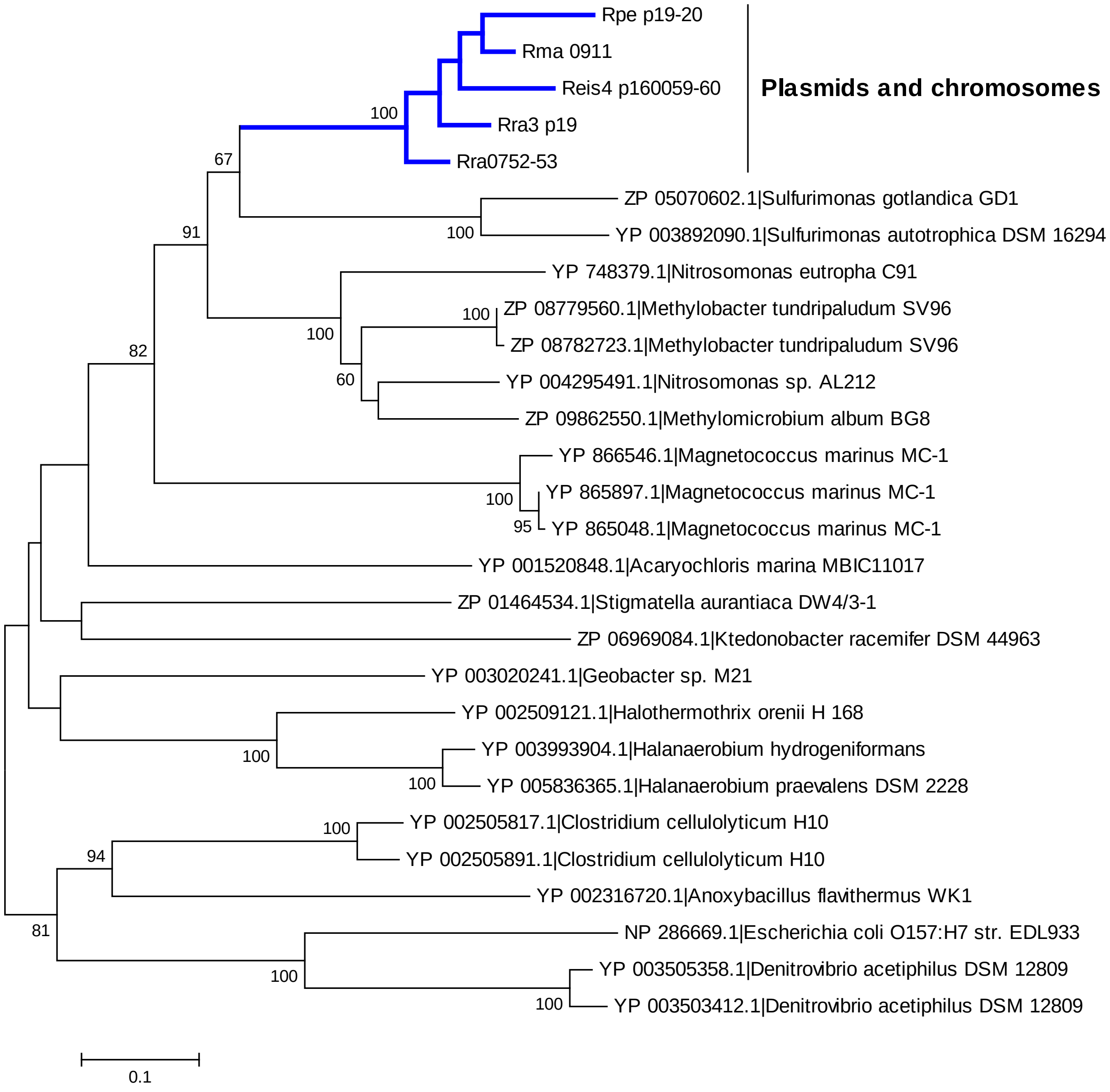

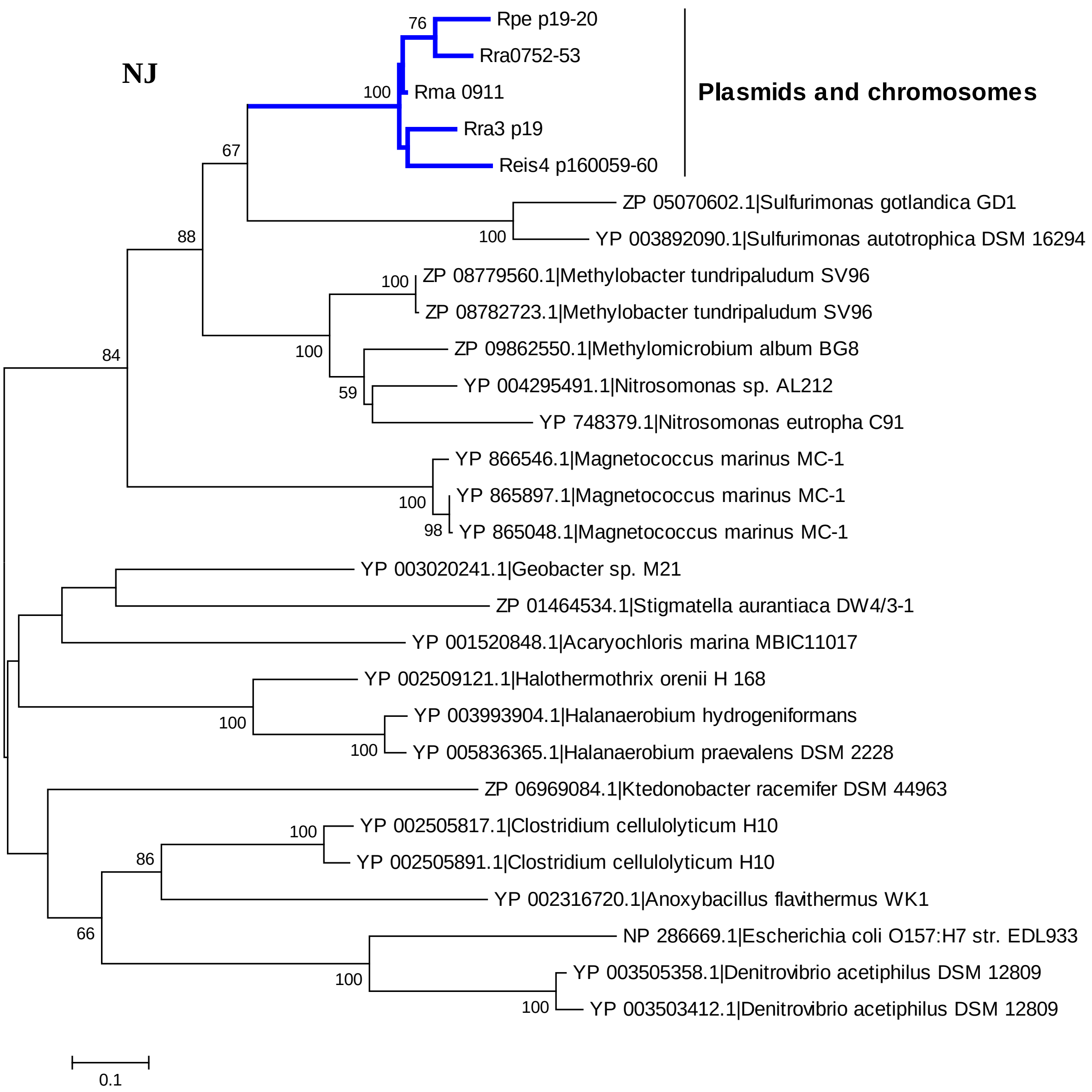
NJ
Plasmids and chromosomes
Rpe p19-20
Rra0752-53
Rma 0911
Rra3 p19
Reis4 p160059-60
ZP 05070602.1|Sulfurimonas gotlandica GD1
YP 003892090.1|Sulfurimonas autotrophica DSM 16294
ZP 08779560.1|Methylobacter tundripaludum SV96
ZP 08782723.1|Methylobacter tundripaludum SV96
ZP 09862550.1|Methylomicrobium album BG8
YP 004295491.1|Nitrosomonas sp. AL212
YP 748379.1|Nitrosomonas eutropha C91
YP 866546.1|Magnetococcus marinus MC-1
YP 865897.1|Magnetococcus marinus MC-1
YP 865048.1|Magnetococcus marinus MC-1
YP 003020241.1|Geobacter sp. M21
ZP 01464534.1|Stigmatella aurantiaca DW4/3-1
YP 001520848.1|Acaryochloris marina MBIC11017
YP 002509121.1|Halothermothrix orenii H 168
YP 003993904.1|Halanaerobium hydrogeniformans
YP 005836365.1|Halanaerobium praevalens DSM 2228
ZP 06969084.1|Ktedonobacter racemifer DSM 44963
YP 002505817.1|Clostridium cellulolyticum H10
YP 002505891.1|Clostridium cellulolyticum H10
YP 002316720.1|Anoxybacillus flavithermus WK1
NP 286669.1|Escherichia coli O157:H7 str. EDL933
YP 003505358.1|Denitrovibrio acetiphilus DSM 12809
YP 003503412.1|Denitrovibrio acetiphilus DSM 12809
76
100
67
88
84
100
100
100
59
100
98
100
100
100
86
66
100
100
0.1

Figure A55. Neighbor-joining (NJ) and maximum likelihood (ML) trees of transposase/integrase containing HTH_28 and rve domains. Bootstrap supports higher than or equal to 60% are shown on the branches.

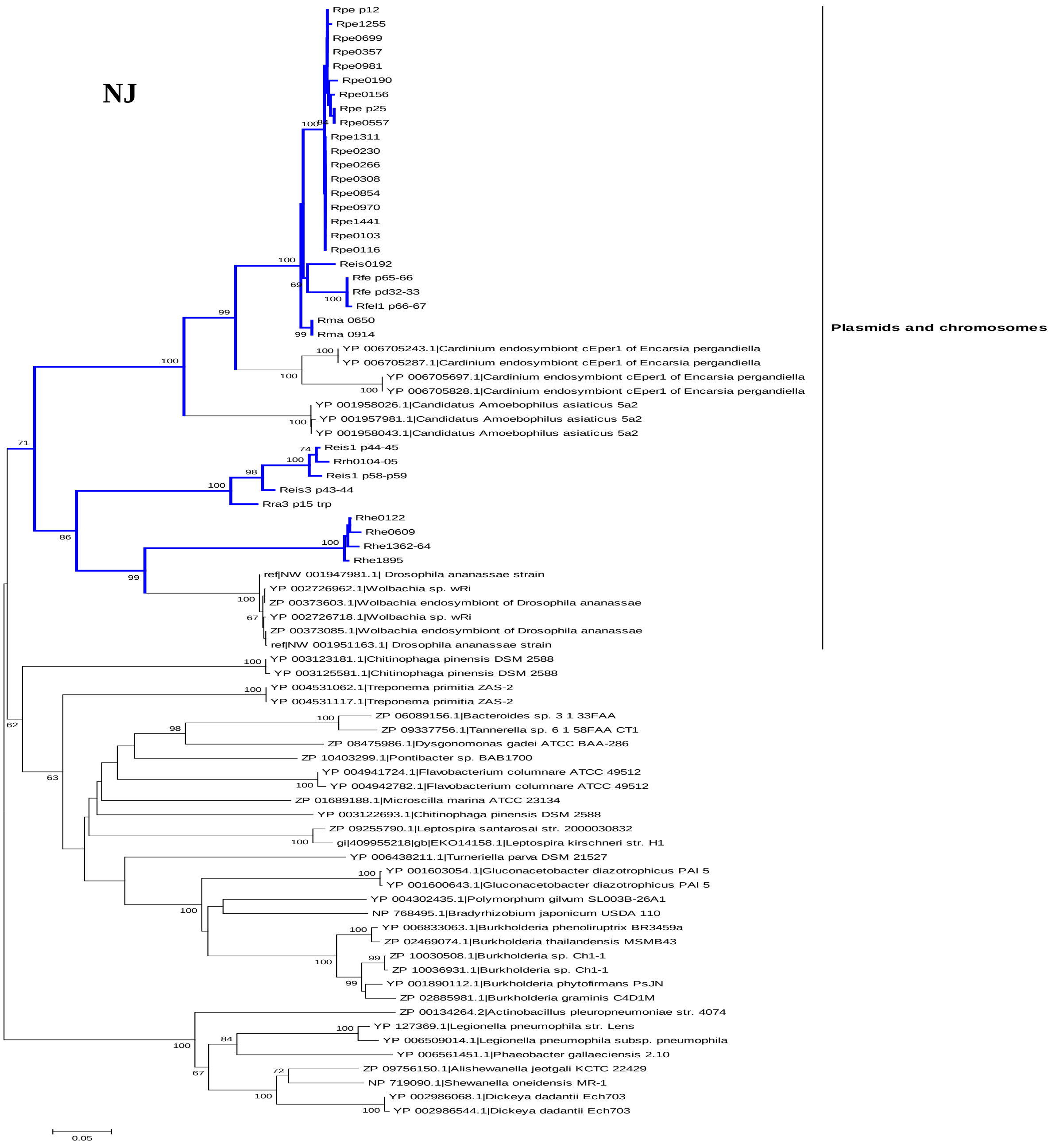

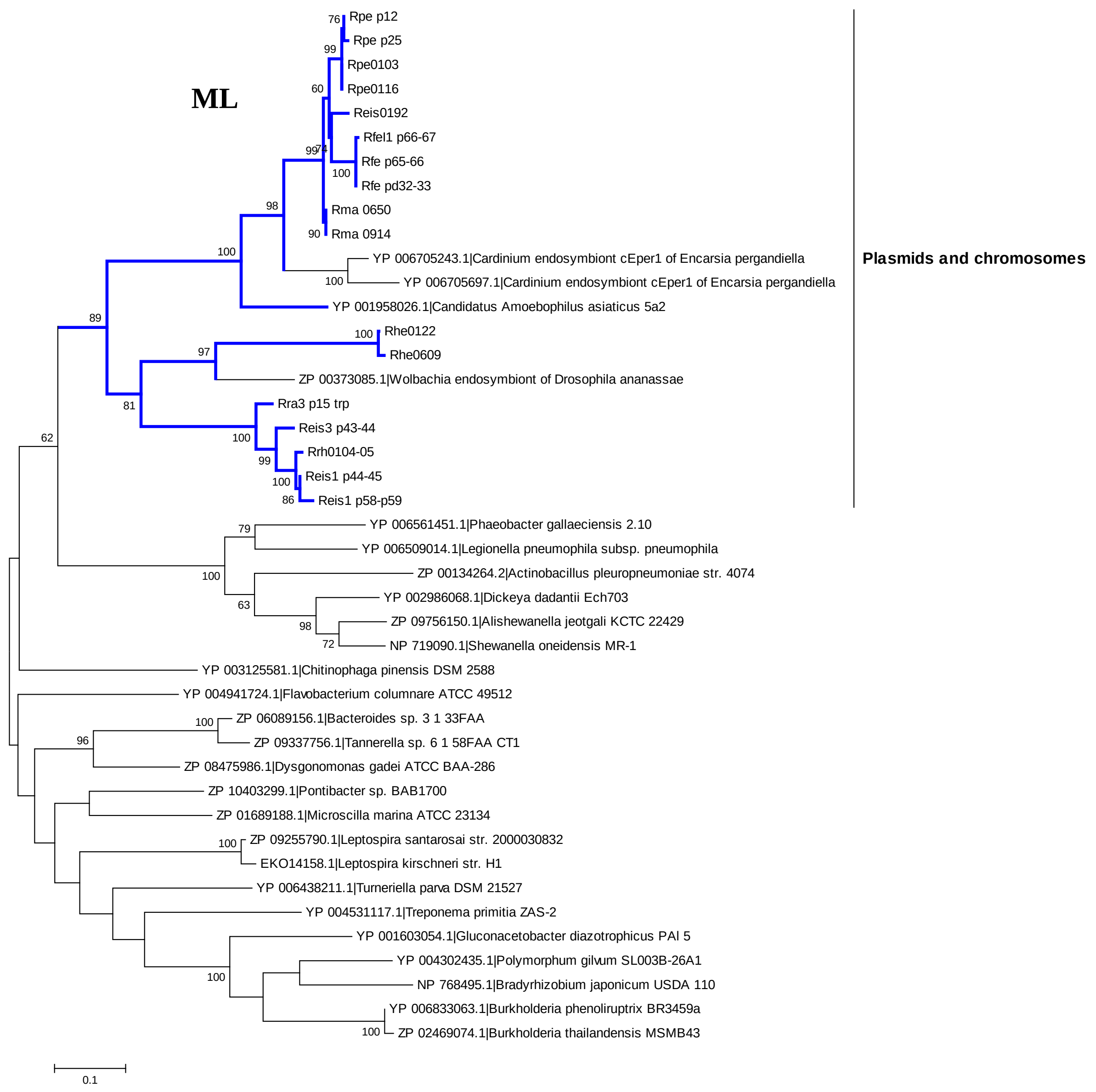

ML
Rpe p12
Rpe p25
Rpe0103
Rpe0116
Reis0192
Rfel1 p66-67
Rfe p65-66
Rfe pd32-33
Rma 0650
Rma 0914
YP 006705243.1|Cardinium endosymbiont cEper1 of Encarsia pergandiella
YP 006705697.1|Cardinium endosymbiont cEper1 of Encarsia pergandiella
YP 001958026.1|Candidatus Amoebophilus asiaticus 5a2
Rhe0122
Rhe0609
ZP 00373085.1|Wolbachia endosymbiont of Drosophila ananassae
Rra3 p15 trp
Reis3 p43-44
Rrh0104-05
Reis1 p44-45
Reis1 p58-p59
YP 006561451.1|Phaeobacter gallaeciensis 2.10
YP 006509014.1|Legionella pneumophila subsp. pneumophila
ZP 00134264.2|Actinobacillus pleuropneumoniae str. 4074
YP 002986068.1|Dickeya dadantii Ech703
ZP 09756150.1|Alishewanella jeotgali KCTC 22429
NP 719090.1|Shewanella oneidensis MR-1
YP 003125581.1|Chitinophaga pinensis DSM 2588
YP 004941724.1|Flavobacterium columnare ATCC 49512
ZP 06089156.1|Bacteroides sp. 3 1 33FAA
ZP 09337756.1|Tannerella sp. 6 1 58FAA CT1
ZP 08475986.1|Dysgonomonas gadei ATCC BAA-286
ZP 10403299.1|Pontibacter sp. BAB1700
ZP 01689188.1|Microscilla marina ATCC 23134
ZP 09255790.1|Leptospira santarosai str. 2000030832
EKO14158.1|Leptospira kirschneri str. H1
YP 006438211.1|Turneriella parva DSM 21527
YP 004531117.1|Treponema primitia ZAS-2
YP 001603054.1|Gluconacetobacter diazotrophicus PAI 5
YP 004302435.1|Polymorphum gilvum SL003B-26A1
NP 768495.1|Bradyrhizobium japonicum USDA 110
YP 006833063.1|Burkholderia phenoliruptrix BR3459a
ZP 02469074.1|Burkholderia thailandensis MSMB43
Plasmids and chromosomes
0.1

Figure A56. Neighbor-joining (NJ) and maximum likelihood (ML) trees of transposase_IS240 /integrase containing rve domain. Bootstrap supports higher than or equal to 60% are shown on the branches.

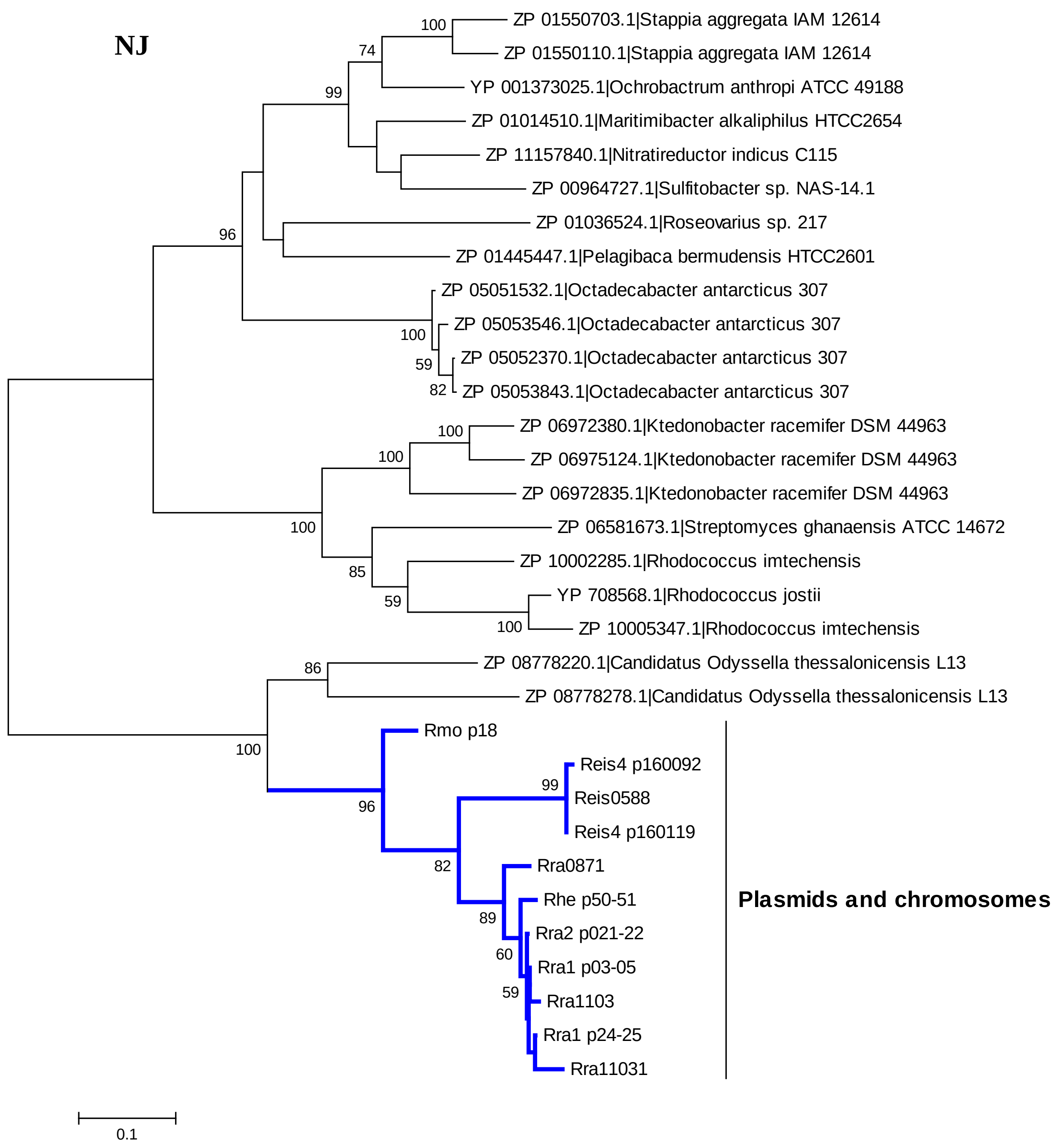

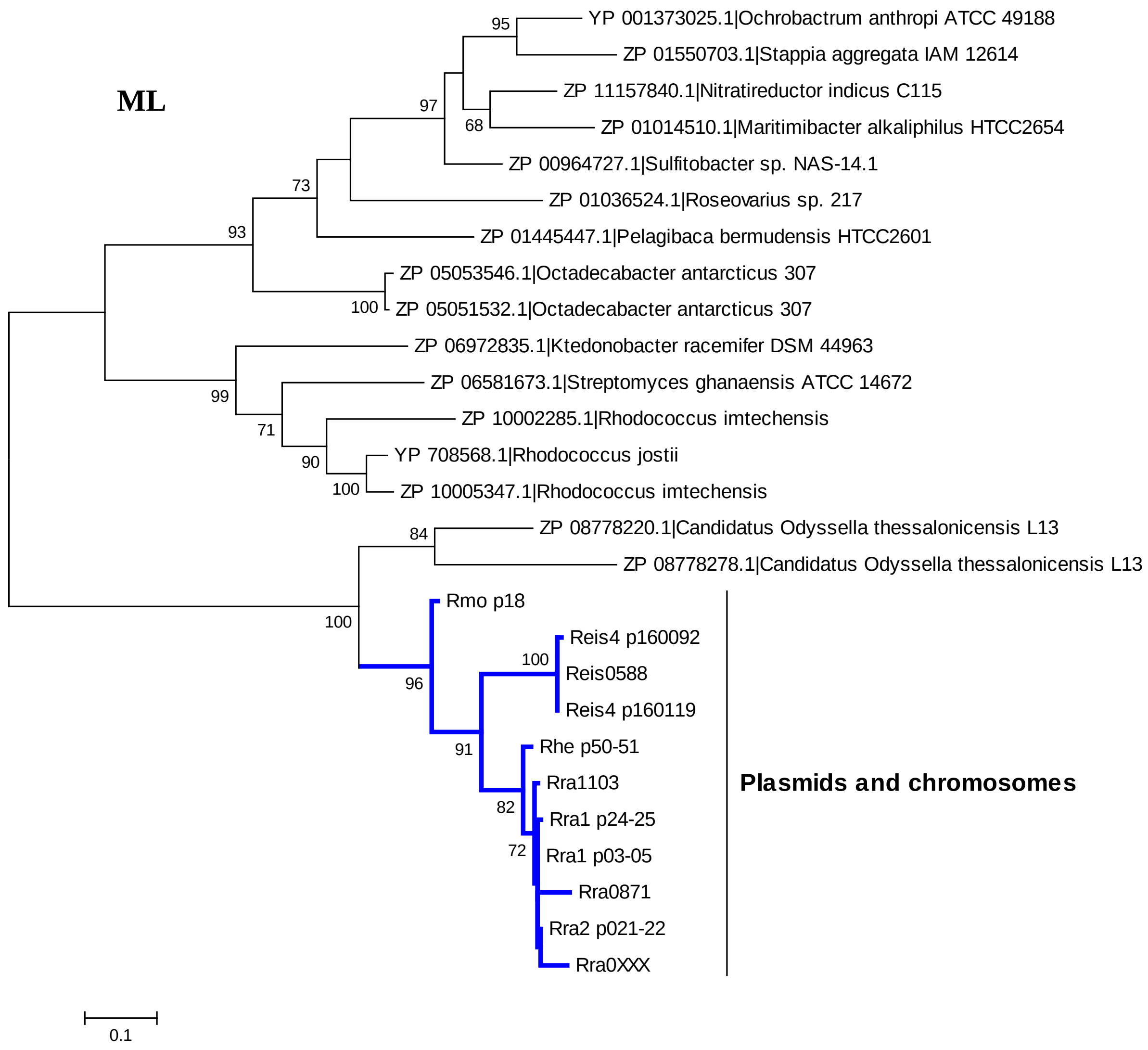
ML
YP 001373025.1|Ochrobactrum anthropi ATCC 49188
ZP 01550703.1|Stappia aggregata IAM 12614
ZP 11157840.1|Nitratireductor indicus C115
ZP 01014510.1|Maritimibacter alkaliphilus HTCC2654
ZP 00964727.1|Sulfitobacter sp. NAS-14.1
ZP 01036524.1|Roseovarius sp. 217
ZP 01445447.1|Pelagibaca bermudensis HTCC2601
ZP 05053546.1|Octadecabacter antarcticus 307
ZP 05051532.1|Octadecabacter antarcticus 307
ZP 06972835.1|Ktedonobacter racemifer DSM 44963
ZP 06581673.1|Streptomyces ghanaensis ATCC 14672
ZP 10002285.1|Rhodococcus imtechensis
YP 708568.1|Rhodococcus jostii
ZP 10005347.1|Rhodococcus imtechensis
ZP 08778220.1|Candidatus Odyssella thessalonicensis L13
ZP 08778278.1|Candidatus Odyssella thessalonicensis L13
Rmo p18
Reis4 p160092
Reis0588
Reis4 p160119
Rhe p50-51
Rra1103
Rra1 p24-25
Rra1 p03-05
Rra0871
Rra2 p021-22
Rra0XXX
Plasmids and chromosomes
95
97
68
73
93
100
99
71
90
100
84
100
96
100
91
82
72
0.1

Figure A57. Neighbor-joining (NJ) and maximum likelihood (ML) trees of transposase mutator family, containing MULE domain. Bootstrap supports higher than or equal to 60% are shown on the branches.

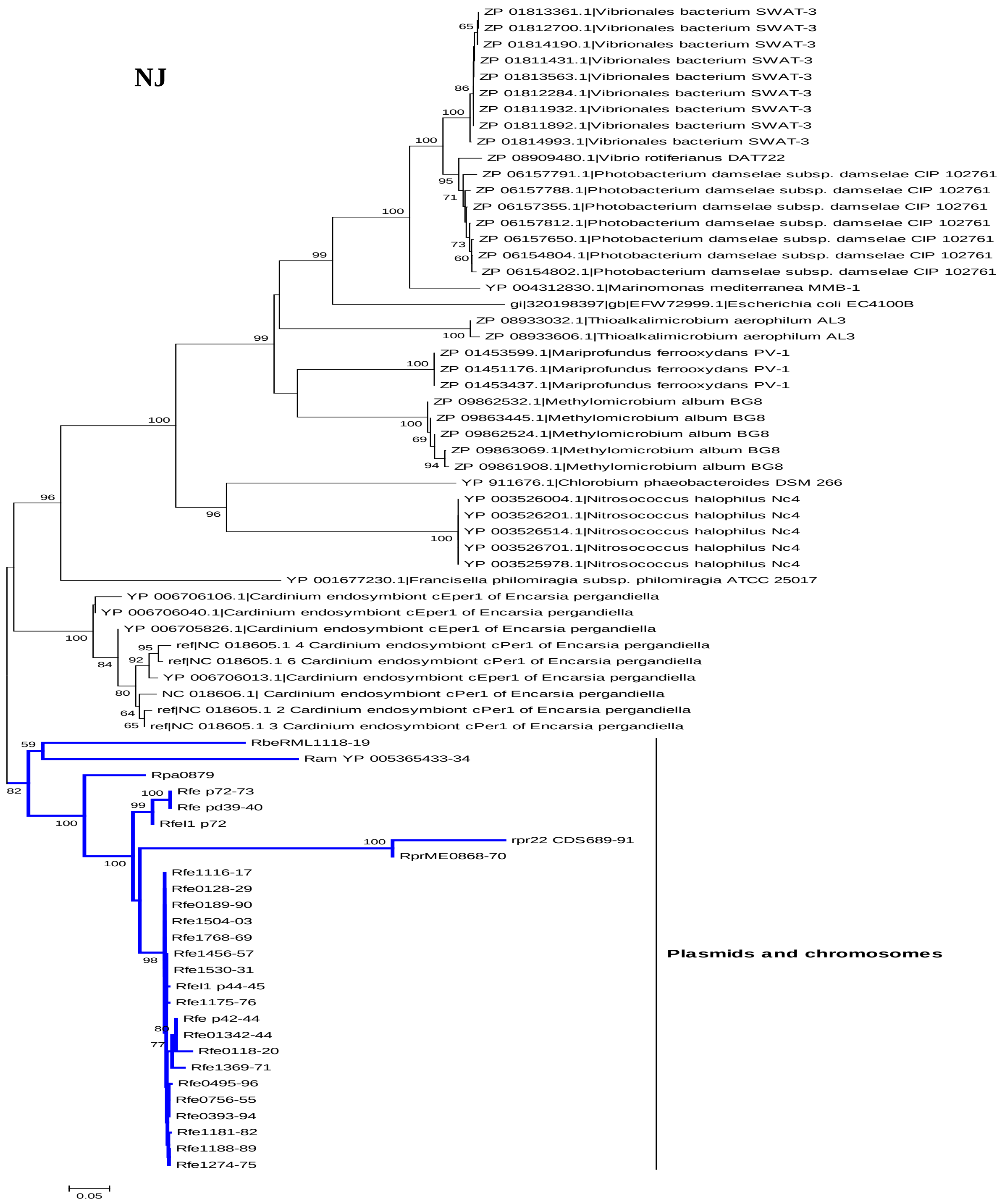

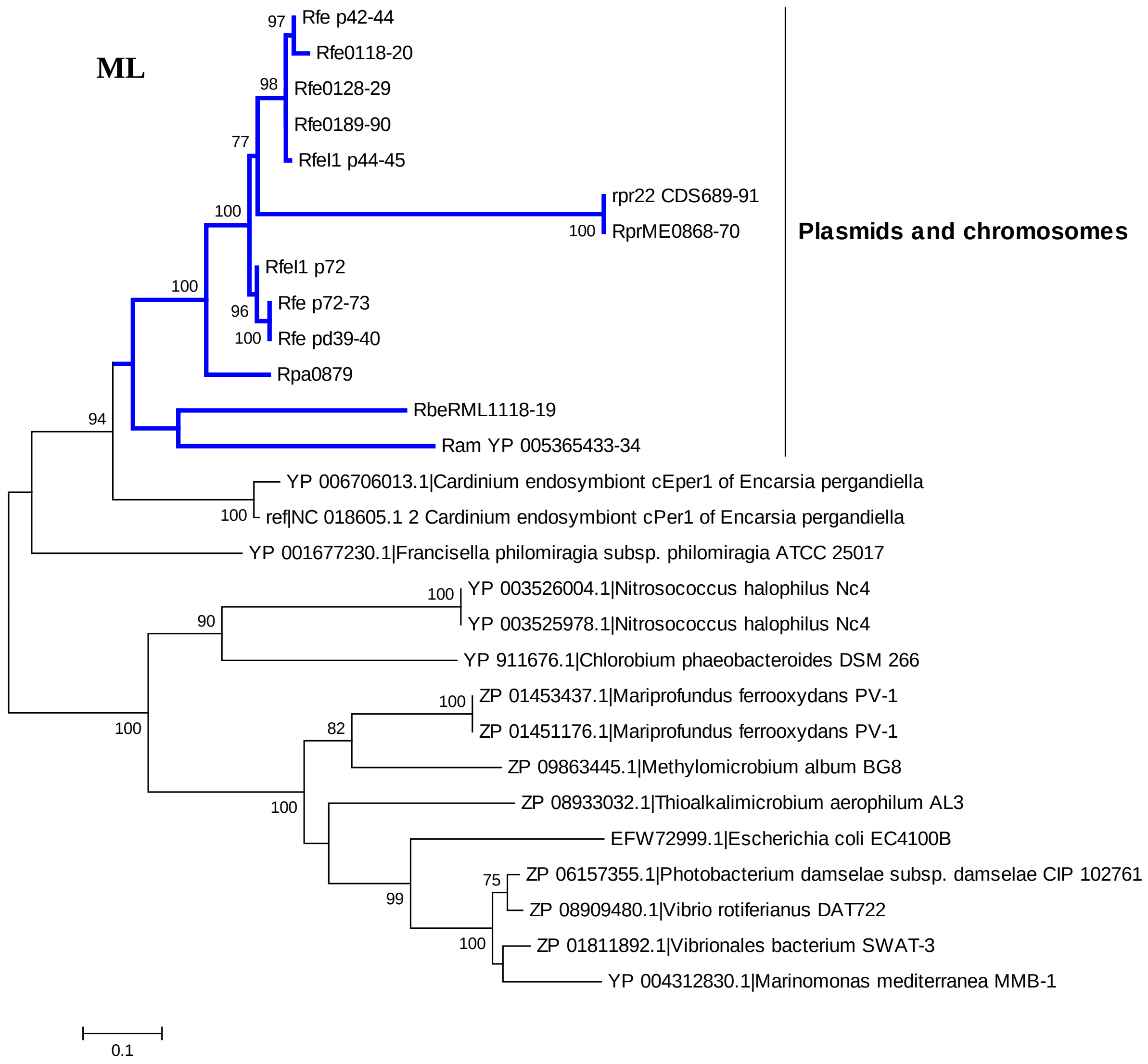

ML
Rfe p42-44
Rfe0118-20
Rfe0128-29
Rfe0189-90
Rfel1 p44-45
rpr22 CDS689-91
RprME0868-70
Rfel1 p72
Rfe p72-73
Rfe pd39-40
Rpa0879
RbeRML1118-19
Ram YP 005365433-34
Plasmids and chromosomes
YP 006706013.1|Cardinium endosymbiont cEper1 of Encarsia pergandiella
ref|NC 018605.1 2 Cardinium endosymbiont cPer1 of Encarsia pergandiella
YP 001677230.1|Francisella philomiragia subsp. philomiragia ATCC 25017
YP 003526004.1|Nitrosococcus halophilus Nc4
YP 003525978.1|Nitrosococcus halophilus Nc4
YP 911676.1|Chlorobium phaeobacteroides DSM 266
ZP 01453437.1|Mariprofundus ferrooxydans PV-1
ZP 01451176.1|Mariprofundus ferrooxydans PV-1
ZP 09863445.1|Methylomicrobium album BG8
ZP 08933032.1|Thioalkalimicrobium aerophilum AL3
EFW72999.1|Escherichia coli EC4100B
ZP 06157355.1|Photobacterium damselae subsp. damselae CIP 102761
ZP 08909480.1|Vibrio rotiferianus DAT722
ZP 01811892.1|Vibrionales bacterium SWAT-3
YP 004312830.1|Marinomonas mediterranea MMB-1
0.1

Figure A58. Neighbor-joining (NJ) and maximum likelihood (ML) trees of transposase mutator family, containing MULE domain. Bootstrap supports higher than or equal to 60% are shown on the branches.

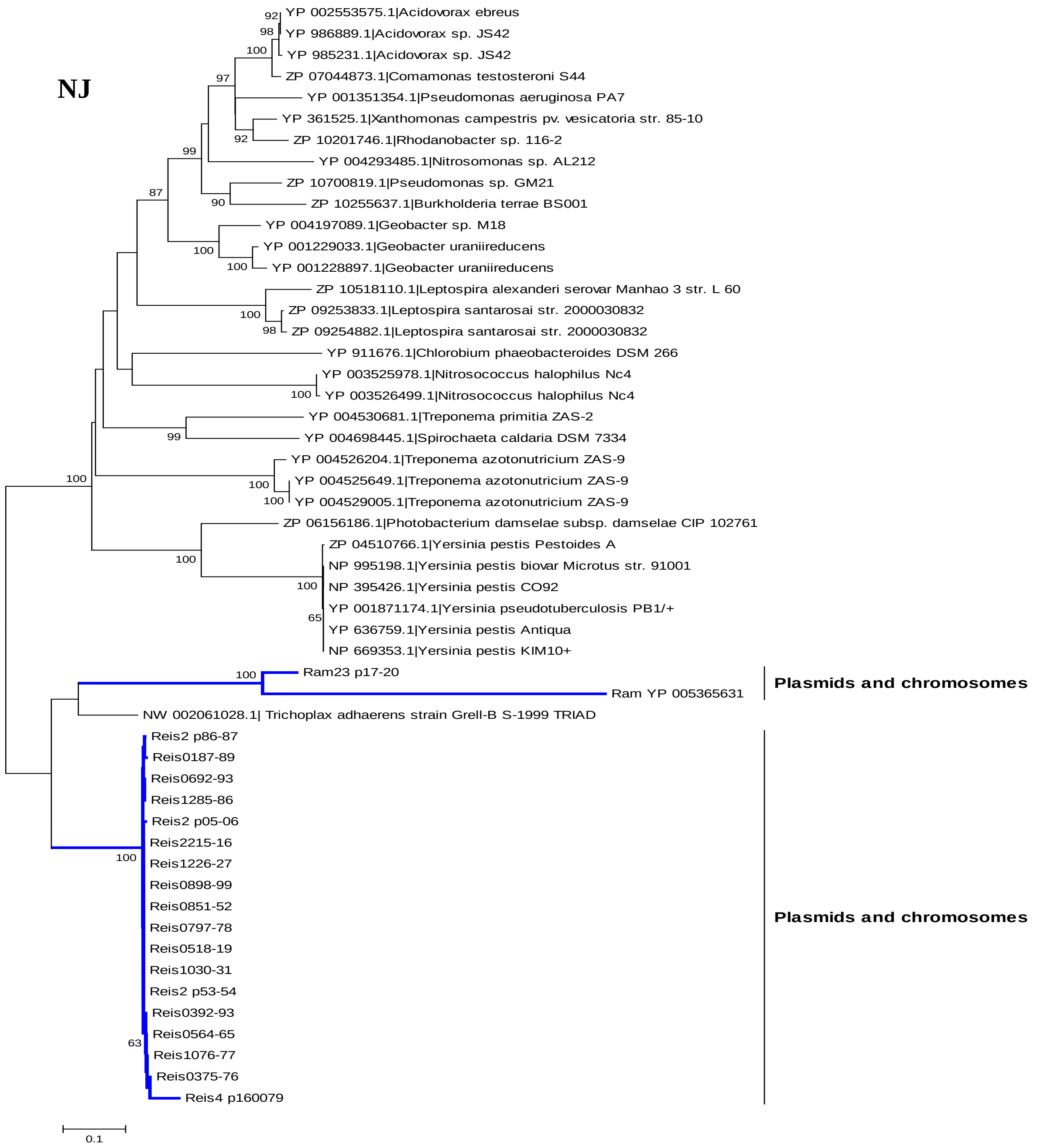

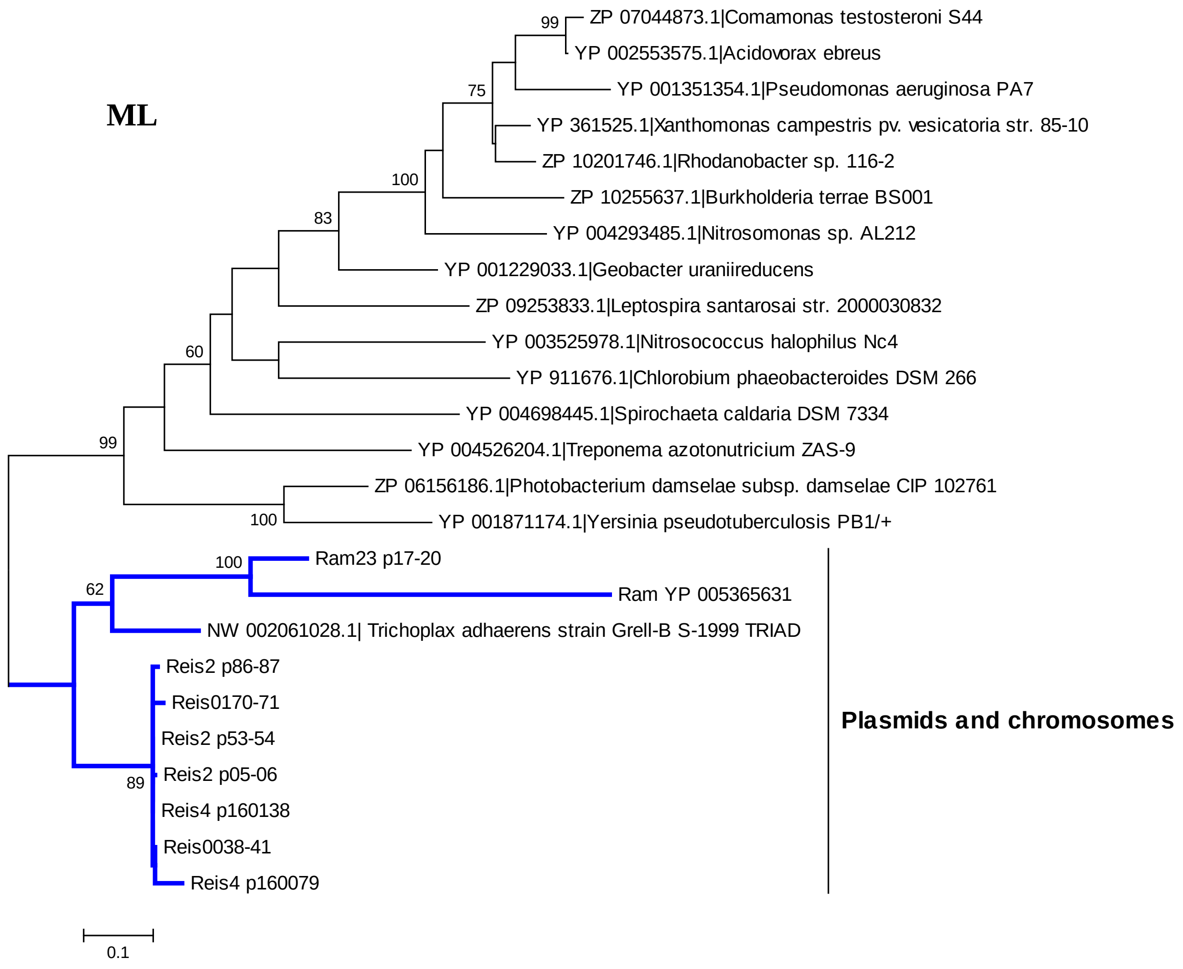
ML
ZP 07044873.1|Comamonas testosteroni S44
YP 002553575.1|Acidovorax ebreus
YP 001351354.1|Pseudomonas aeruginosa PA7
YP 361525.1|Xanthomonas campestris pv. vesicatoria str. 85-10
ZP 10201746.1|Rhodanobacter sp. 116-2
ZP 10255637.1|Burkholderia terrae BS001
YP 004293485.1|Nitrosomonas sp. AL212
YP 001229033.1|Geobacter uraniireducens
ZP 09253833.1|Leptospira santarosai str. 2000030832
YP 003525978.1|Nitrosococcus halophilus Nc4
YP 911676.1|Chlorobium phaeobacteroides DSM 266
YP 004698445.1|Spirochaeta caldaria DSM 7334
YP 004526204.1|Treponema azotonutricium ZAS-9
ZP 06156186.1|Photobacterium damselae subsp. damselae CIP 102761
YP 001871174.1|Yersinia pseudotuberculosis PB1/+
Ram23 p17-20
Ram YP 005365631
NW 002061028.1| Trichoplax adhaerens strain Grell-B S-1999 TRIAD
Reis2 p86-87
Reis0170-71
Reis2 p53-54
Reis2 p05-06
Reis4 p160138
Reis0038-41
Reis4 p160079
Plasmids and chromosomes
99
75
100
83
60
99
100
100
62
89
0.1

Figure A59. Neighbor-joining (NJ) and maximum likelihood (ML) trees of transposase containing HTH_28 and DDE_3 domain. Bootstrap supports higher than or equal to 60% are shown on the branches.

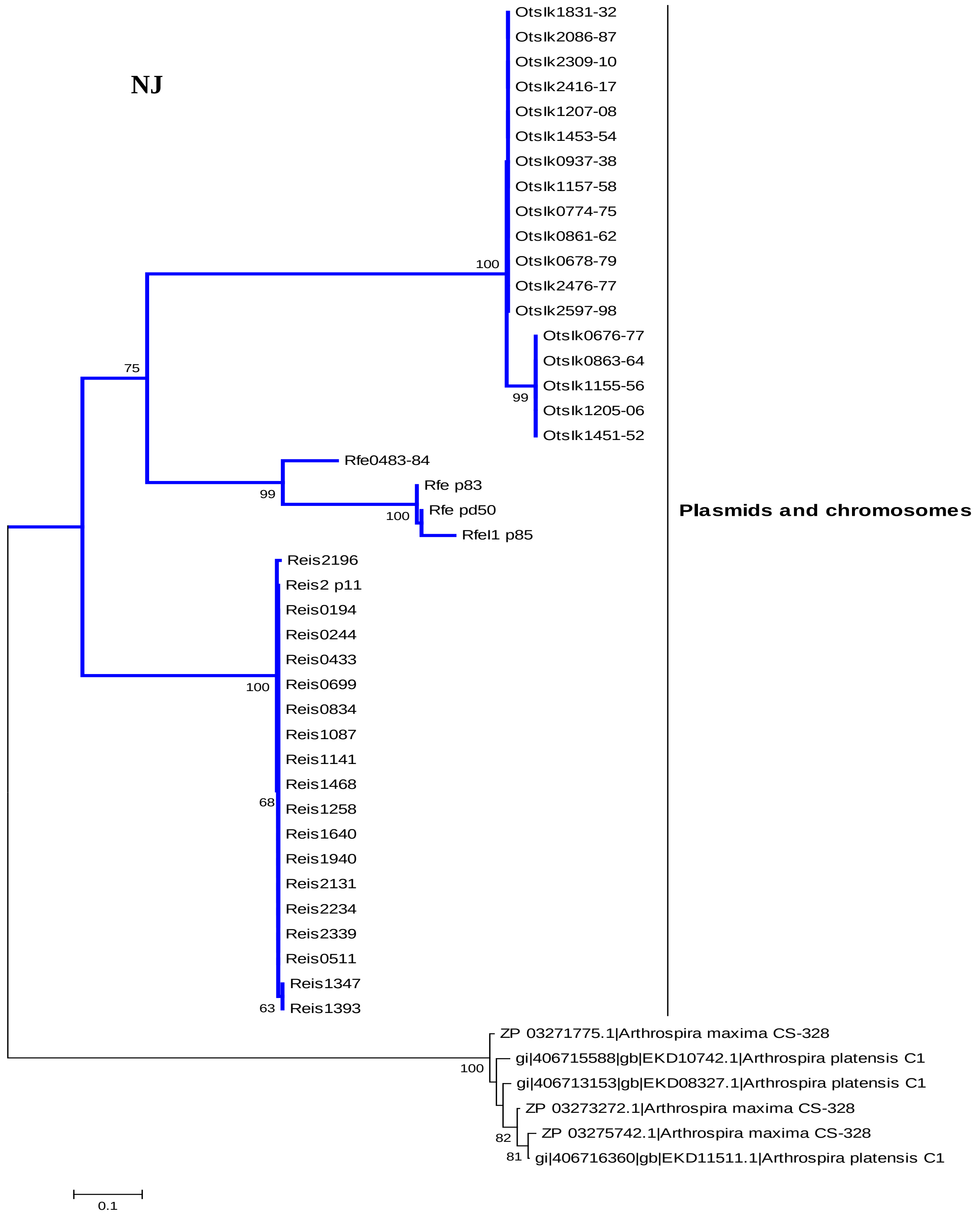

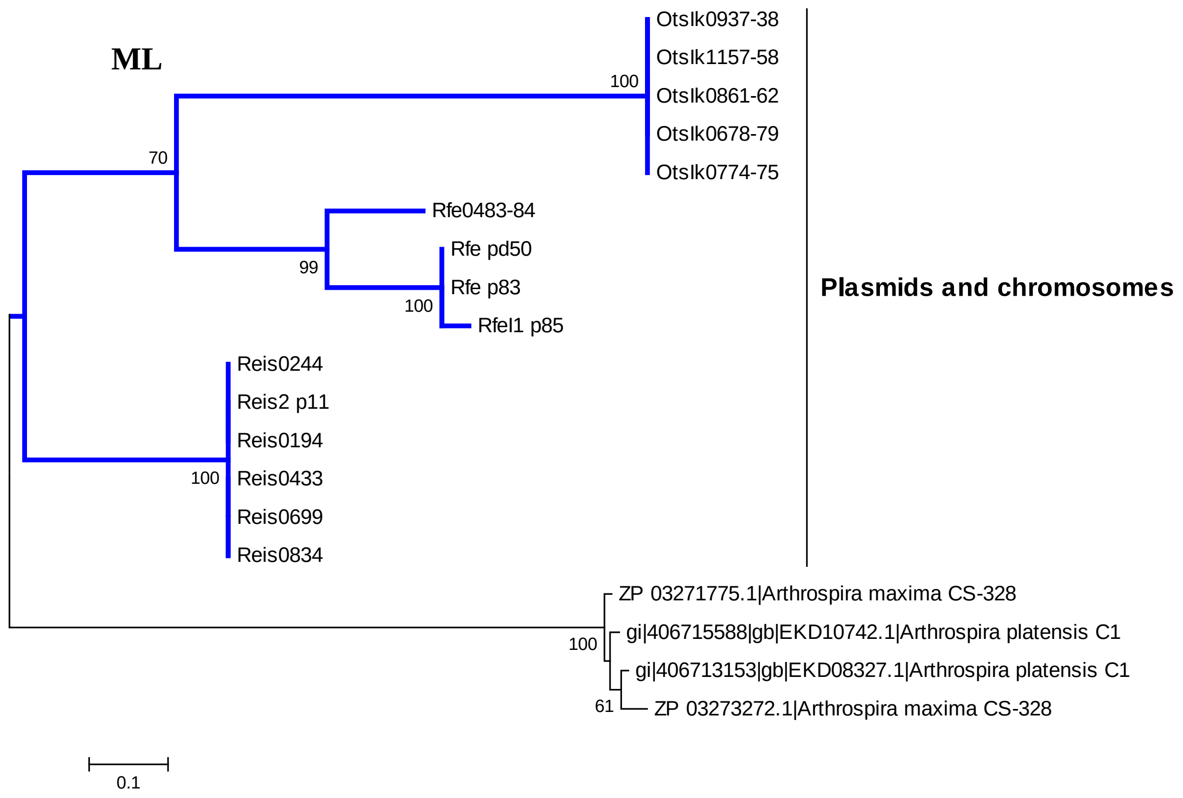
ML
OtsIk0937-38
OtsIk1157-58
OtsIk0861-62
OtsIk0678-79
OtsIk0774-75
100
70
Rfe0483-84
Rfe pd50
Rfe p83
Rfel1 p85
99
100
Reis0244
Reis2 p11
Reis0194
Reis0433
Reis0699
Reis0834
100
Plasmids and chromosomes
ZP 03271775.1|Arthrospira maxima CS-328
gi|406715588|gb|EKD10742.1|Arthrospira platensis C1
gi|406713153|gb|EKD08327.1|Arthrospira platensis C1
ZP 03273272.1|Arthrospira maxima CS-328
100
61
0.1

Figure A60. Neighbor-joining (NJ) and maximum likelihood (ML) trees of transposase IS200 familiy protein containing Y1 domain. Bootstrap supports higher than or equal to 60% are shown on the branches.

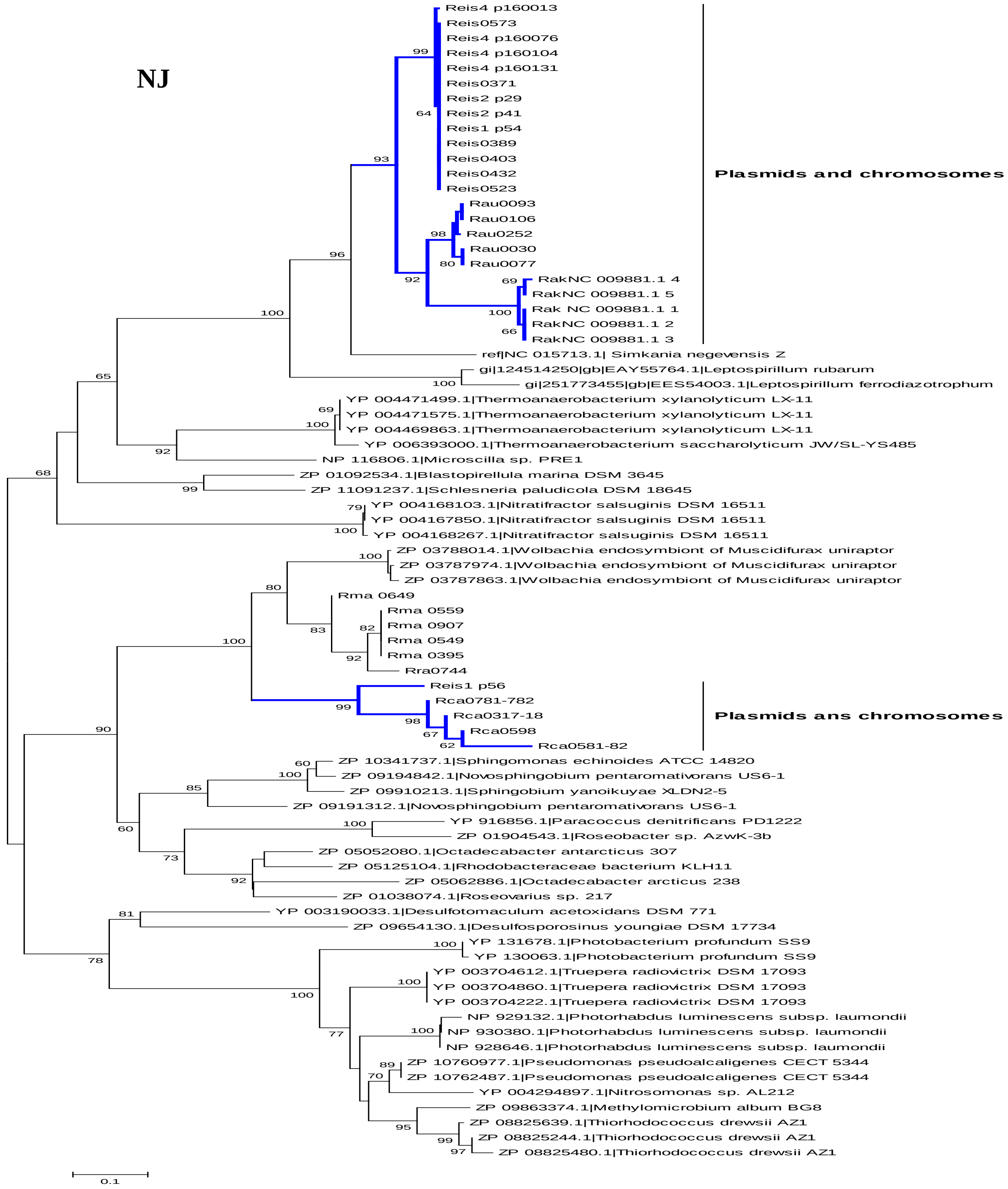

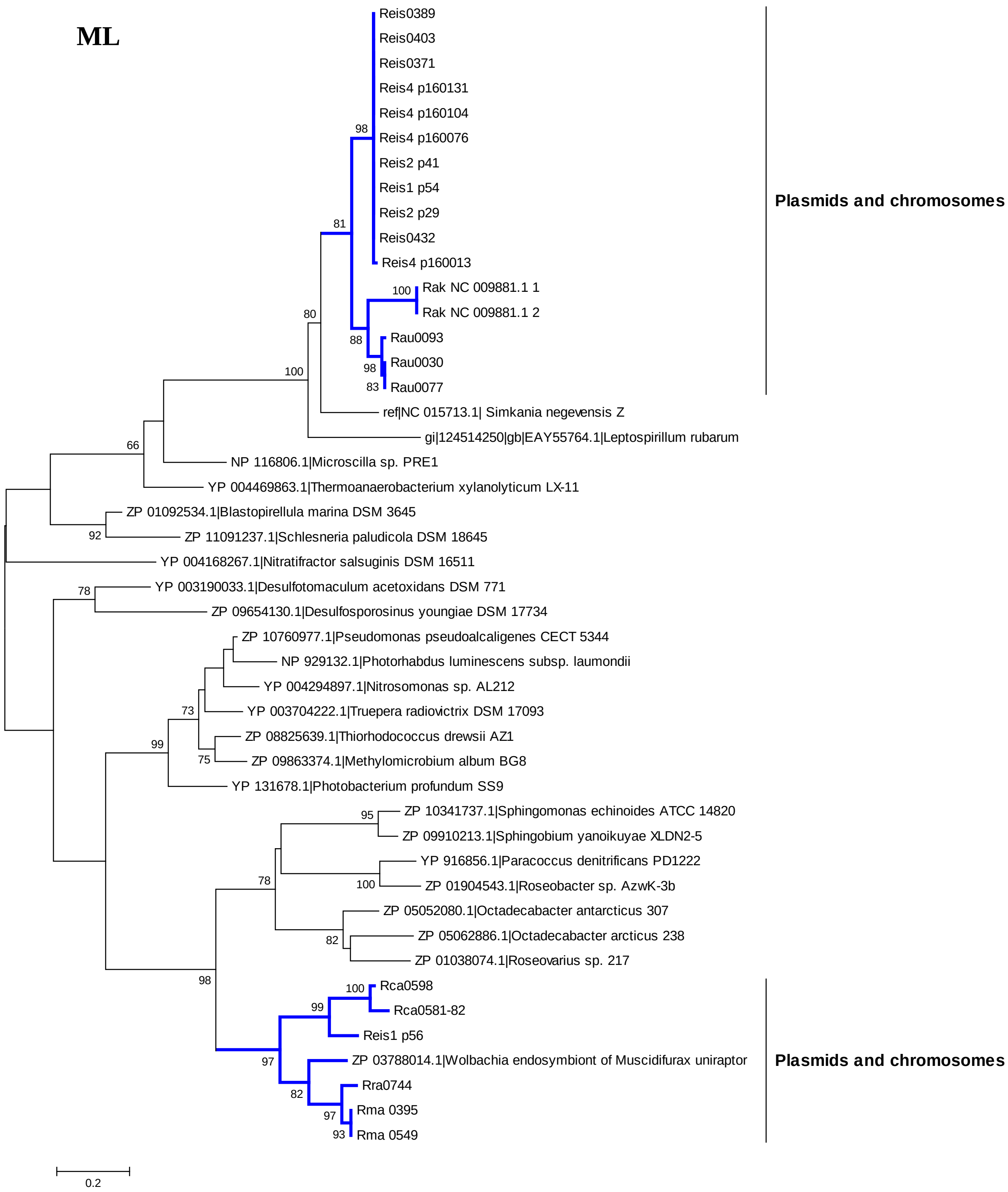
ML
Reis0389
Reis0403
Reis0371
Reis4 p160131
Reis4 p160104
Reis4 p160076
Reis2 p41
Reis1 p54
Reis2 p29
Reis0432
Reis4 p160013
Rak NC 009881.1 1
Rak NC 009881.1 2
Rau0093
Rau0030
Rau0077
ref|NC 015713.1| Simkania negevensis Z
gi|124514250|gb|EAY55764.1|Leptospirillum rubarum
NP 116806.1|Microscilla sp. PRE1
YP 004469863.1|Thermoanaerobacterium xylanolyticum LX-11
ZP 01092534.1|Blastopirellula marina DSM 3645
ZP 11091237.1|Schlesneria paludicola DSM 18645
YP 004168267.1|Nitratifractor salsuginis DSM 16511
YP 003190033.1|Desulfotomaculum acetoxidans DSM 771
ZP 09654130.1|Desulfosporosinus youngiae DSM 17734
ZP 10760977.1|Pseudomonas pseudoalcaligenes CECT 5344
NP 929132.1|Photorhabdus luminescens subsp. laumondii
YP 004294897.1|Nitrosomonas sp. AL212
YP 003704222.1|Truepera radiovictrix DSM 17093
ZP 08825639.1|Thiorhodococcus drewsii AZ1
ZP 09863374.1|Methylomicrobium album BG8
YP 131678.1|Photobacterium profundum SS9
ZP 10341737.1|Sphingomonas echinoides ATCC 14820
ZP 09910213.1|Sphingobium yanoikuyae XLDN2-5
YP 916856.1|Paracoccus denitrificans PD1222
ZP 01904543.1|Roseobacter sp. AzwK-3b
ZP 05052080.1|Octadecabacter antarcticus 307
ZP 05062886.1|Octadecabacter arcticus 238
ZP 01038074.1|Roseovarius sp. 217
Rca0598
Rca0581-82
Reis1 p56
ZP 03788014.1|Wolbachia endosymbiont of Muscidifurax uniraptor
Rra0744
Rma 0395
Rma 0549
Plasmids and chromosomes
Plasmids and chromosomes
98
81
100
80
88
98
83
100
66
92
78
73
99
75
95
100
78
82
98
100
99
97
82
97
93
0.2

Figure A61. Neighbor-joining (NJ) and maximum likelihood (ML) trees of transposase DDE_Tnp_1 domain. Bootstrap supports higher than or equal to 60% are shown on the branches.

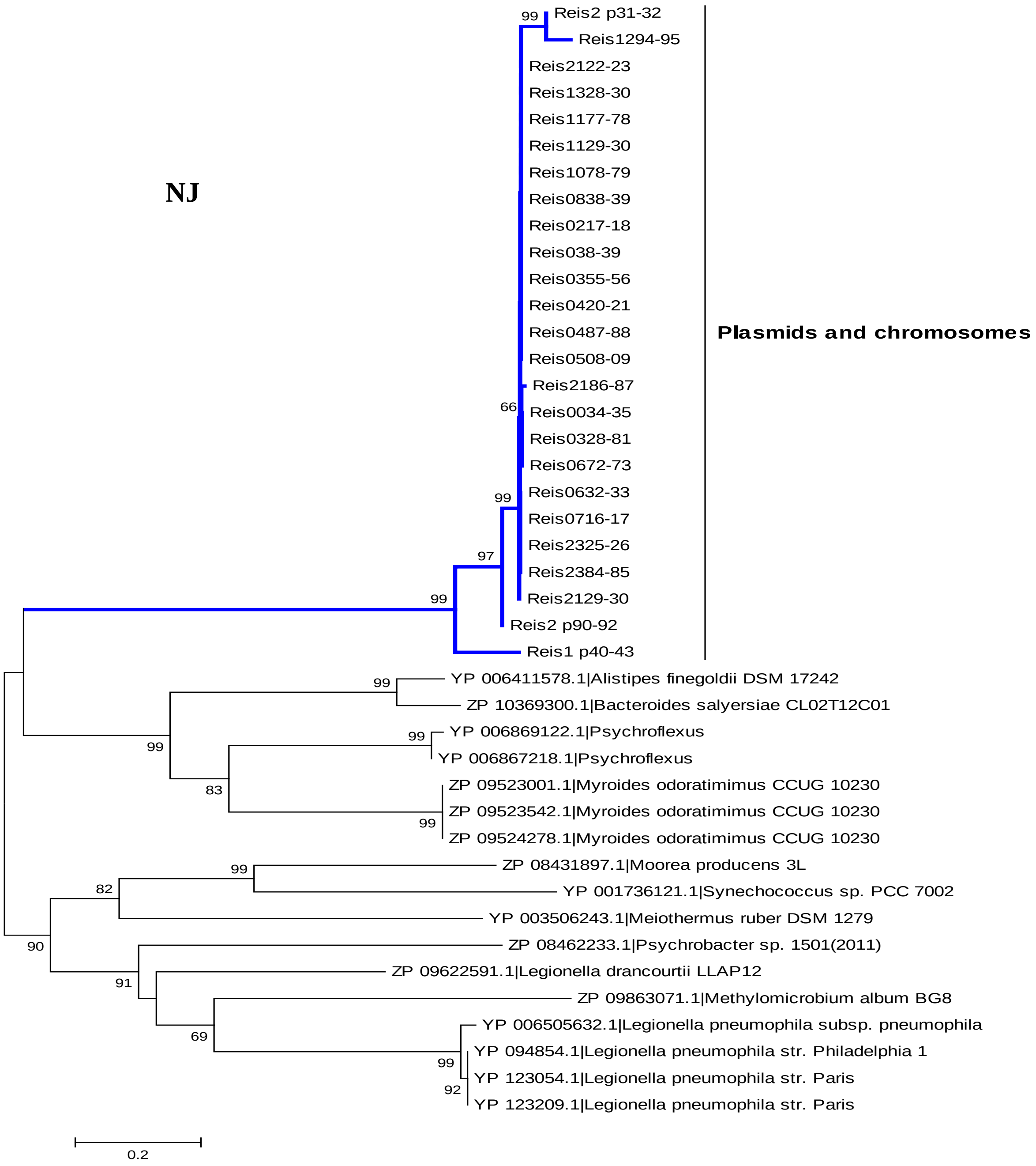

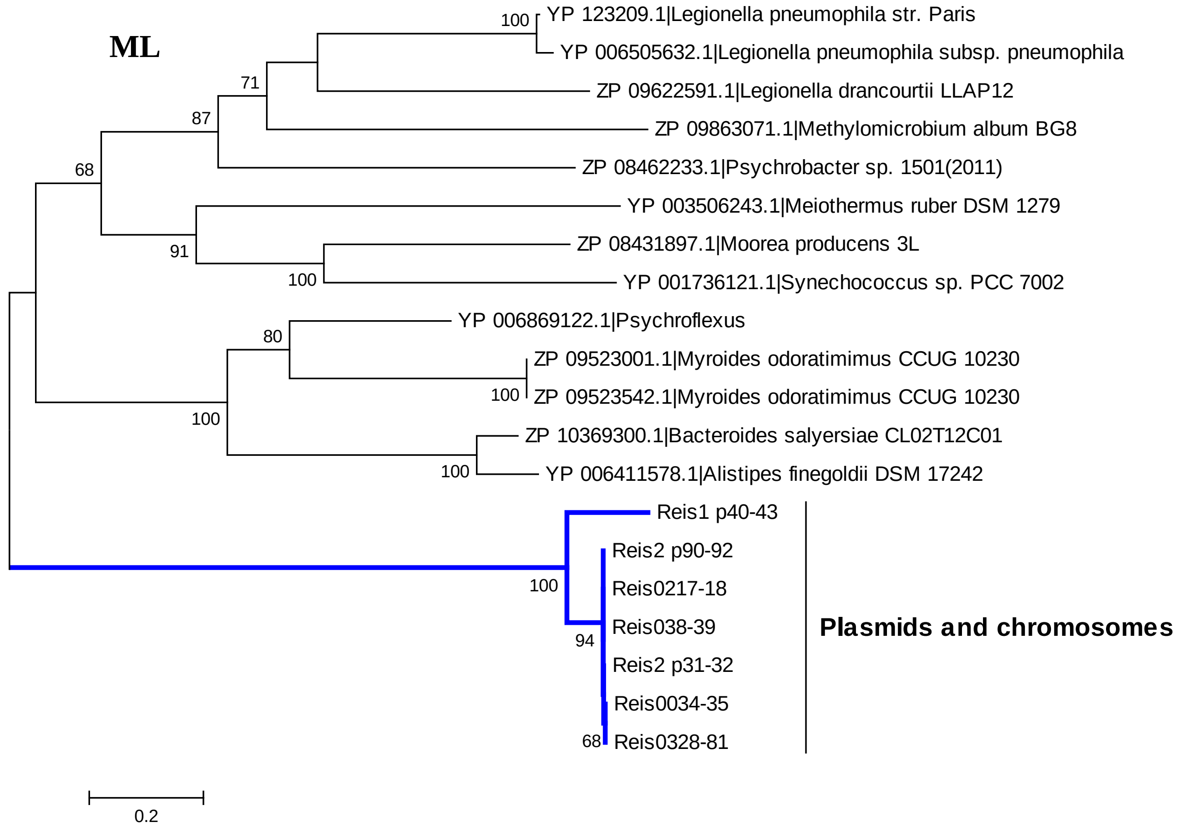

ML
YP 123209.1|Legionella pneumophila str. Paris
YP 006505632.1|Legionella pneumophila subsp. pneumophila
ZP 09622591.1|Legionella drancourtii LLAP12
ZP 09863071.1|Methylomicrobium album BG8
ZP 08462233.1|Psychrobacter sp. 1501(2011)
YP 003506243.1|Meiothermus ruber DSM 1279
ZP 08431897.1|Moorea producens 3L
YP 001736121.1|Synechococcus sp. PCC 7002
YP 006869122.1|Psychroflexus
ZP 09523001.1|Myroides odoratimimus CCUG 10230
ZP 09523542.1|Myroides odoratimimus CCUG 10230
ZP 10369300.1|Bacteroides salyersiae CL02T12C01
YP 006411578.1|Alistipes finegoldii DSM 17242
Reis1 p40-43
Reis2 p90-92
Reis0217-18
Reis038-39
Reis2 p31-32
Reis0034-35
Reis0328-81
Plasmids and chromosomes
100
71
87
68
91
100
80
100
100
100
100
94
68
0.2

Figure A62. Neighbor-joining (NJ) and maximum likelihood (ML) trees of RNA-directed DNA polymerases. Bootstrap supports higher than or equal to 60% are shown on the branches.

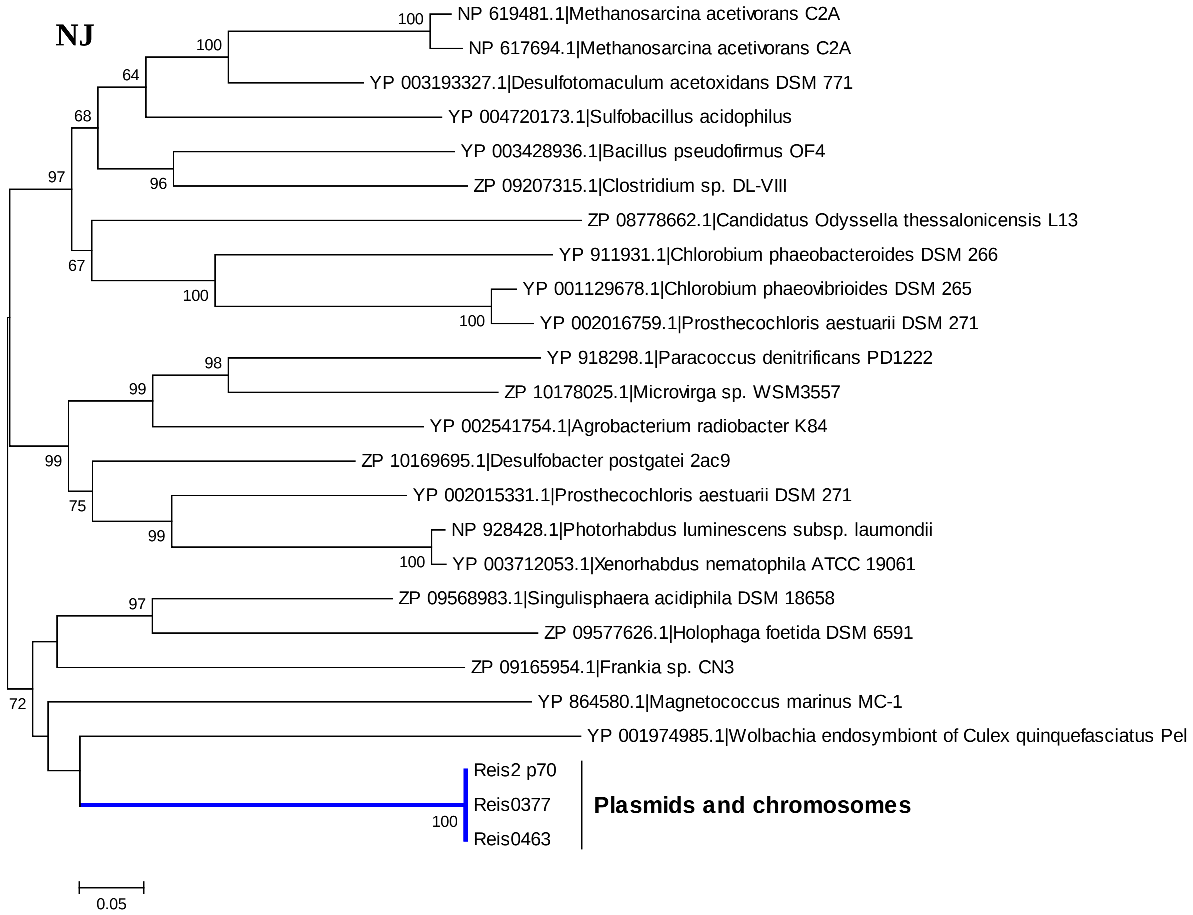

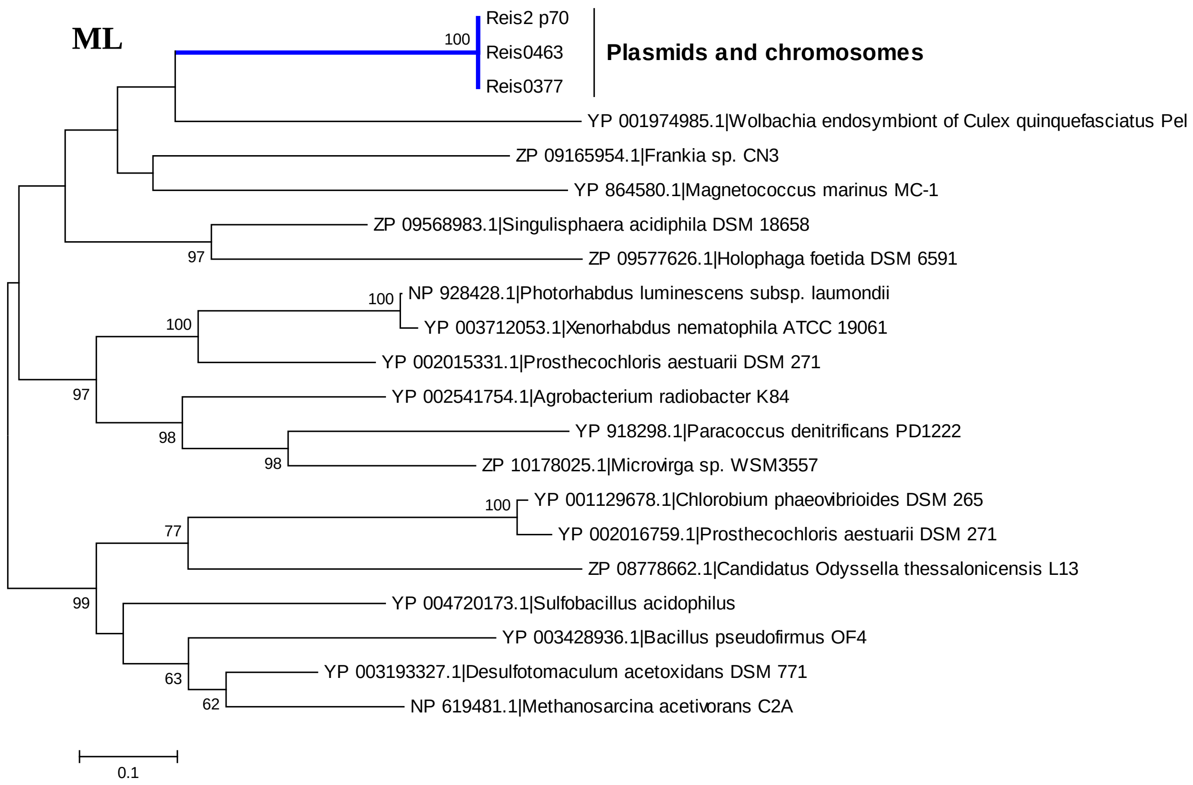
ML
Reis2 p70
Reis0463
Reis0377
Plasmids and chromosomes
100
YP 001974985.1|Wolbachia endosymbiont of Culex quinquefasciatus Pel
ZP 09165954.1|Frankia sp. CN3
YP 864580.1|Magnetococcus marinus MC-1
ZP 09568983.1|Singulisphaera acidiphila DSM 18658
97
ZP 09577626.1|Holophaga foetida DSM 6591
100
NP 928428.1|Photorhabdus luminescens subsp. laumondii
100
YP 003712053.1|Xenorhabdus nematophila ATCC 19061
YP 002015331.1|Prosthecochloris aestuarii DSM 271
97
YP 002541754.1|Agrobacterium radiobacter K84
98
YP 918298.1|Paracoccus denitrificans PD1222
98
ZP 10178025.1|Microvirga sp. WSM3557
100
YP 001129678.1|Chlorobium phaeovibrioides DSM 265
77
YP 002016759.1|Prosthecochloris aestuarii DSM 271
ZP 08778662.1|Candidatus Odyssella thessalonicensis L13
99
YP 004720173.1|Sulfobacillus acidophilus
YP 003428936.1|Bacillus pseudofirmus OF4
63
YP 003193327.1|Desulfotomaculum acetoxidans DSM 771
62
NP 619481.1|Methanosarcina acetivorans C2A
0.1

Figure A63. Neighbor-joining (NJ) and maximum likelihood (ML) trees of conserved protein of unknown function. Bootstrap supports higher than or equal to 60% are shown on the branches.

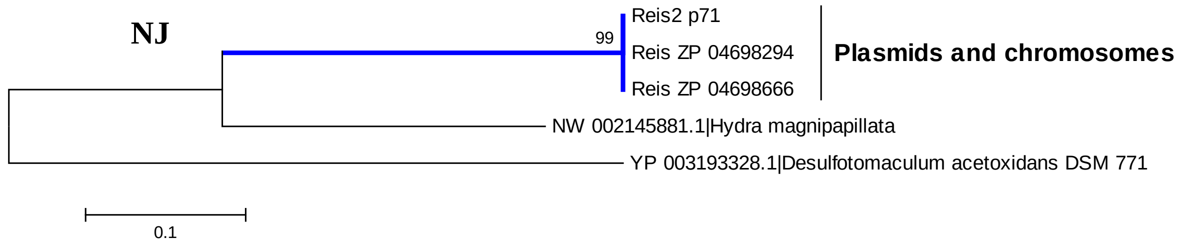

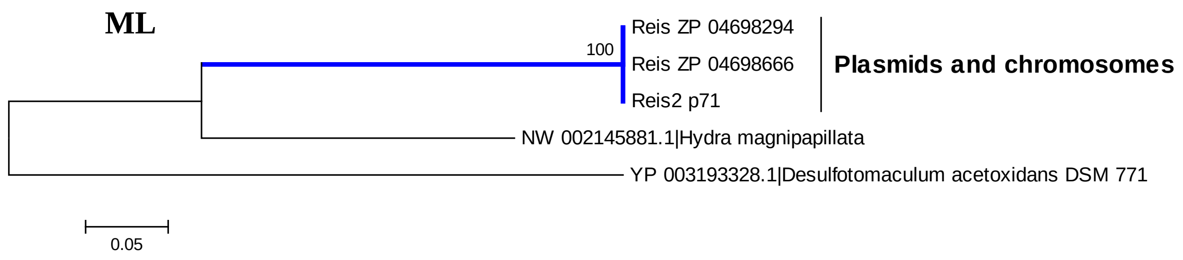
ML
100
Reis ZP 04698294
Reis ZP 04698666
Reis2 p71
Plasmids and chromosomes
NW 002145881.1|Hydra magnipapillata
YP 003193328.1|Desulfotomaculum acetoxidans DSM 771
0.05

Figure A64. Neighbor-joining (NJ) and maximum likelihood (ML) trees of conserved protein of unknown function. Bootstrap supports higher than or equal to 60% are shown on the branches.

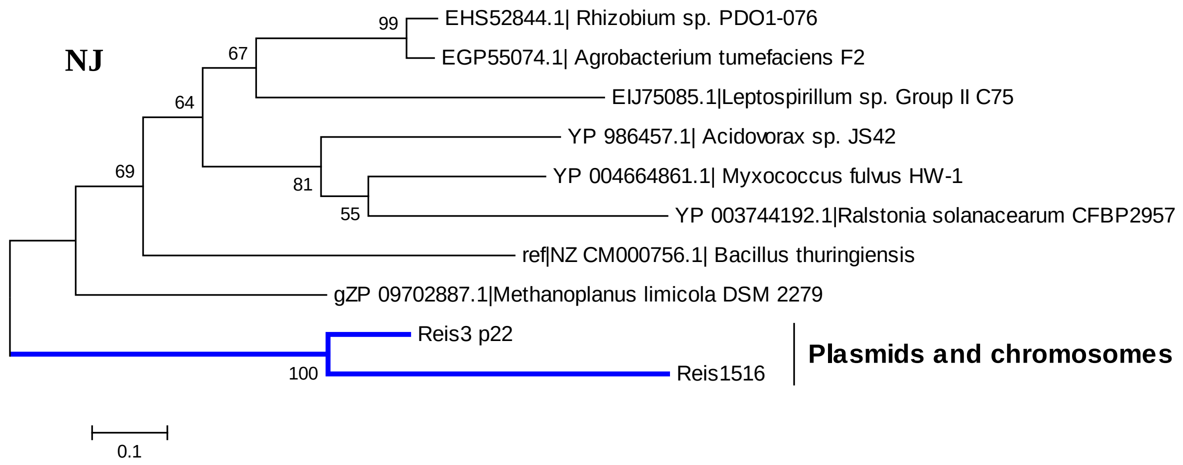

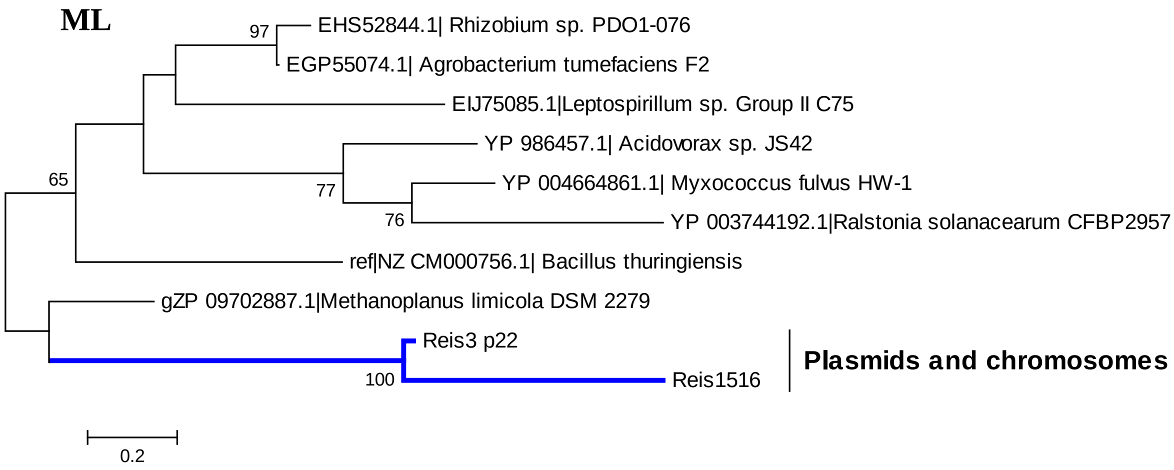
ML
97
EHS52844.1| Rhizobium sp. PDO1-076
EGP55074.1| Agrobacterium tumefaciens F2
EIJ75085.1|Leptospirillum sp. Group II C75
YP 986457.1| Acidovorax sp. JS42
65
77
YP 004664861.1| Myxococcus fulvus HW-1
76
YP 003744192.1|Ralstonia solanacearum CFBP2957
ref|NZ CM000756.1| Bacillus thuringiensis
gZP 09702887.1|Methanoplanus limicola DSM 2279
Reis3 p22
100
Reis1516
Plasmids and chromosomes
0.2

Figure A65. Neighbor-joining (NJ) and maximum likelihood (ML) trees of transposase containing HTH_1 domain. Bootstrap supports higher than or equal to 60% are shown on the branches.

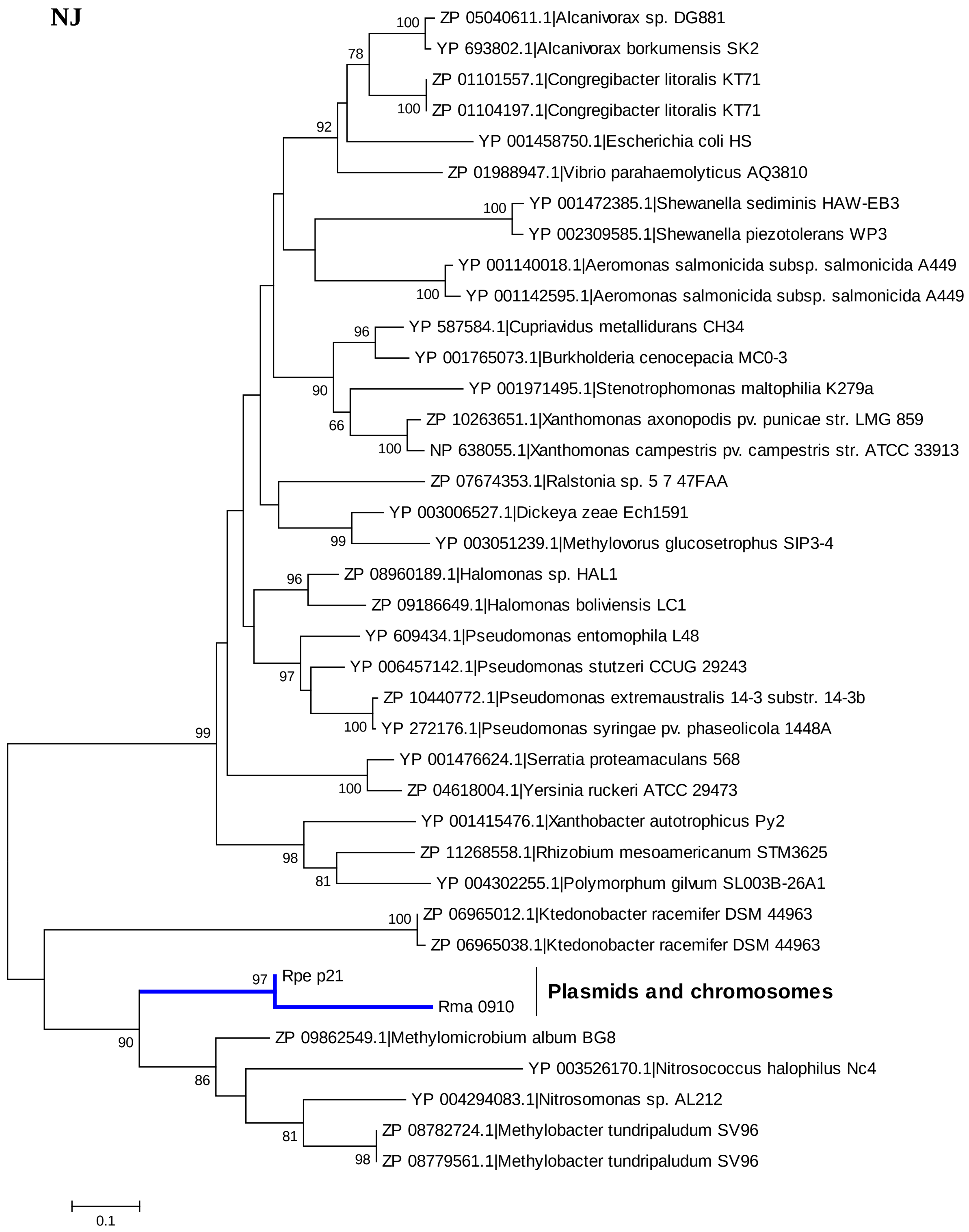

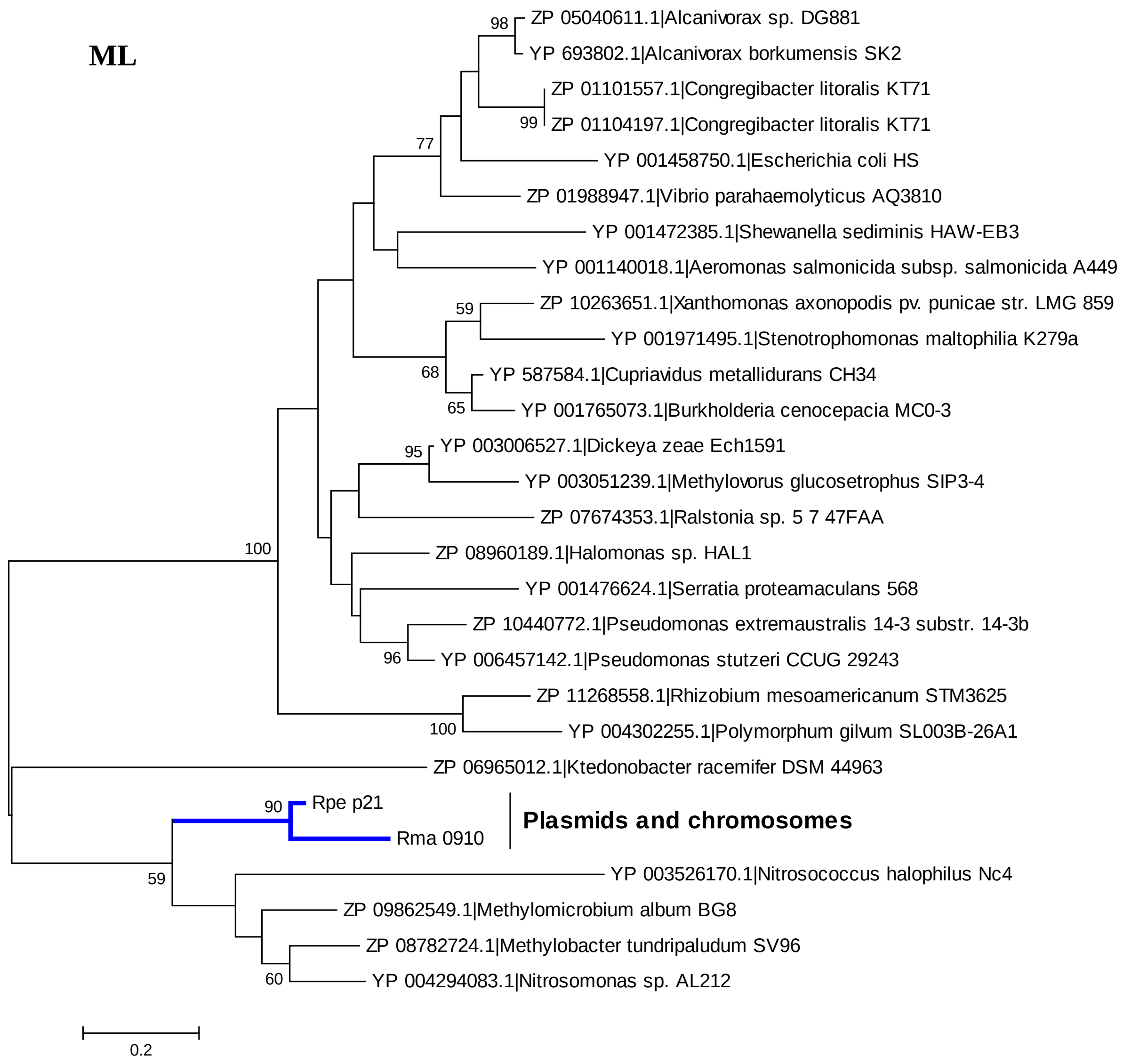
ML
ZP 05040611.1|Alcanivorax sp. DG881
YP 693802.1|Alcanivorax borkumensis SK2
ZP 01101557.1|Congregibacter litoralis KT71
ZP 01104197.1|Congregibacter litoralis KT71
YP 001458750.1|Escherichia coli HS
ZP 01988947.1|Vibrio parahaemolyticus AQ3810
YP 001472385.1|Shewanella sediminis HAW-EB3
YP 001140018.1|Aeromonas salmonicida subsp. salmonicida A449
ZP 10263651.1|Xanthomonas axonopodis pv. punicae str. LMG 859
YP 001971495.1|Stenotrophomonas maltophilia K279a
YP 587584.1|Cupriavidus metallidurans CH34
YP 001765073.1|Burkholderia cenocepacia MC0-3
YP 003006527.1|Dickeya zeae Ech1591
YP 003051239.1|Methylovorus glucosetrophus SIP3-4
ZP 07674353.1|Ralstonia sp. 5 7 47FAA
ZP 08960189.1|Halomonas sp. HAL1
YP 001476624.1|Serratia proteamaculans 568
ZP 10440772.1|Pseudomonas extremaustralis 14-3 substr. 14-3b
YP 006457142.1|Pseudomonas stutzeri CCUG 29243
ZP 11268558.1|Rhizobium mesoamericanum STM3625
YP 004302255.1|Polymorphum gilvum SL003B-26A1
ZP 06965012.1|Ktedonobacter racemifer DSM 44963
Rpe p21
Rma 0910
Plasmids and chromosomes
YP 003526170.1|Nitrosococcus halophilus Nc4
ZP 09862549.1|Methylomicrobium album BG8
ZP 08782724.1|Methylobacter tundripaludum SV96
YP 004294083.1|Nitrosomonas sp. AL212
98
99
77
59
68
65
95
100
96
100
90
59
60
0.2

Figure A66. Neighbor-joining (NJ) and maximum likelihood (ML) trees of transposase containing DDE_Tnp_1_3 domain. Bootstrap supports higher than or equal to 60% are shown on the branches.

NJ

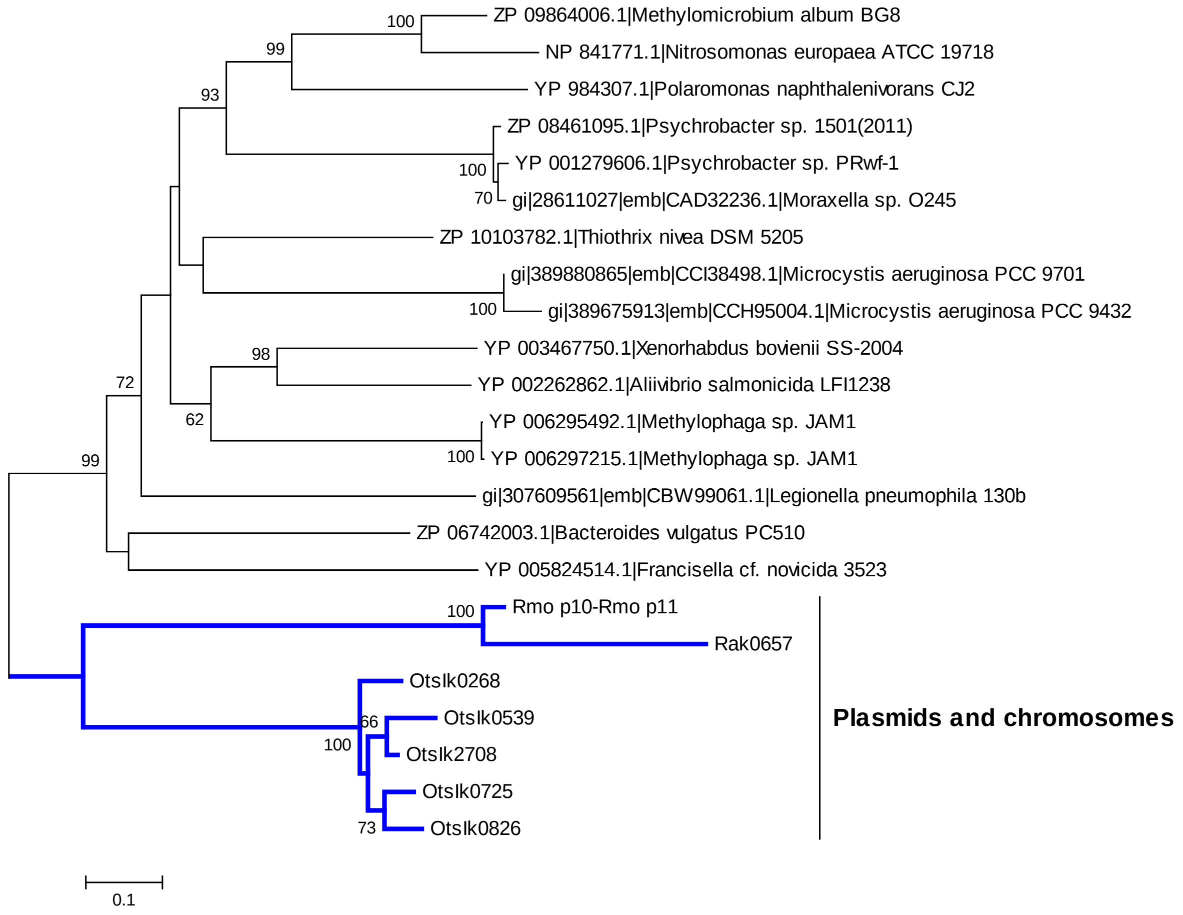

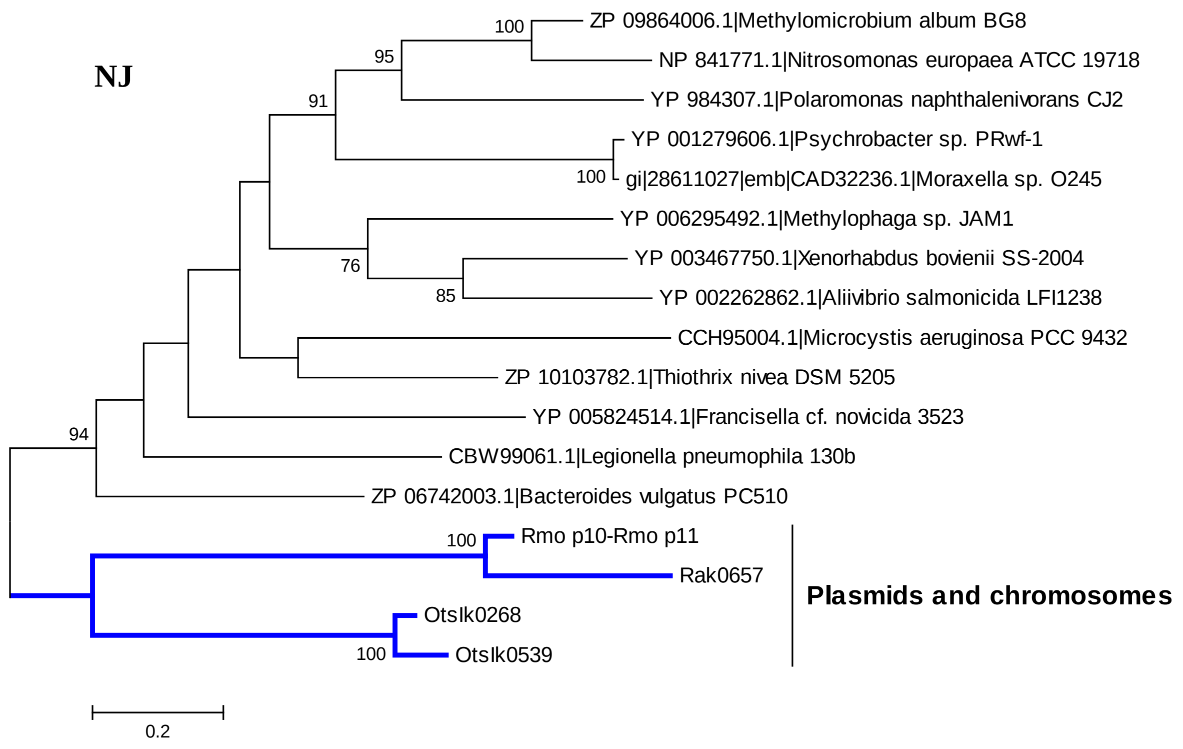
NJ
ZP 09864006.1|Methylomicrobium album BG8
NP 841771.1|Nitrosomonas europaea ATCC 19718
YP 984307.1|Polaromonas naphthalenivorans CJ2
YP 001279606.1|Psychrobacter sp. PRwf-1
gi|28611027|emb|CAD32236.1|Moraxella sp. O245
YP 006295492.1|Methylophaga sp. JAM1
YP 003467750.1|Xenorhabdus bovienii SS-2004
YP 002262862.1|Aliivibrio salmonicida LFI1238
CCH95004.1|Microcystis aeruginosa PCC 9432
ZP 10103782.1|Thiothrix nivea DSM 5205
YP 005824514.1|Francisella cf. novicida 3523
CBW99061.1|Legionella pneumophila 130b
ZP 06742003.1|Bacteroides vulgatus PC510
Rmo p10-Rmo p11
Rak0657
OtsIk0268
OtsIk0539
Plasmids and chromosomes
100
95
91
100
76
85
94
100
100
0.2

Figure A67. Neighbor-joining (NJ) and maximum likelihood (ML) trees of guanosine polyphosphate pyrophosphohydrolase/synthetase SpoT23. Bootstrap supports higher than or equal to 60% are shown on the branches.

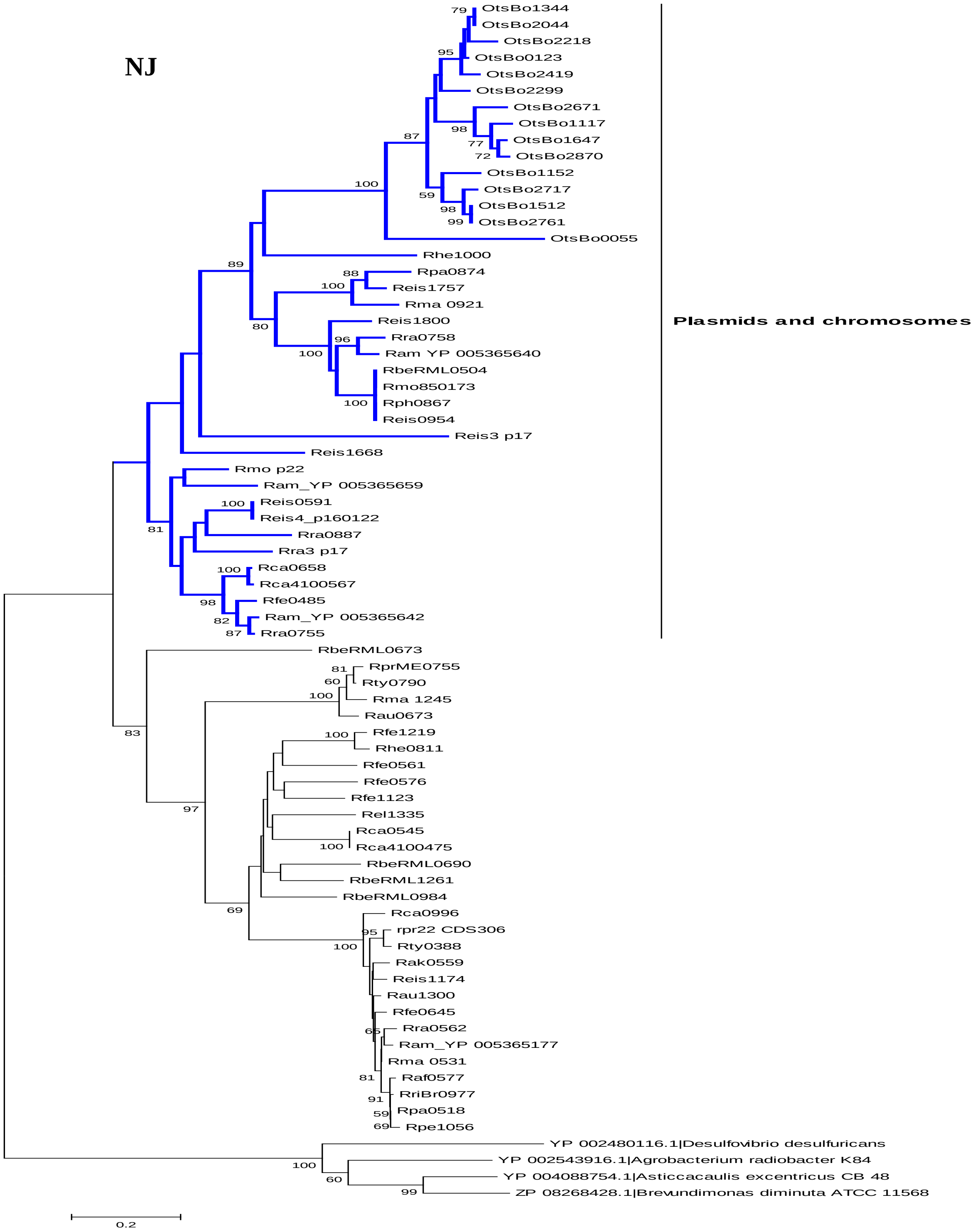

ML

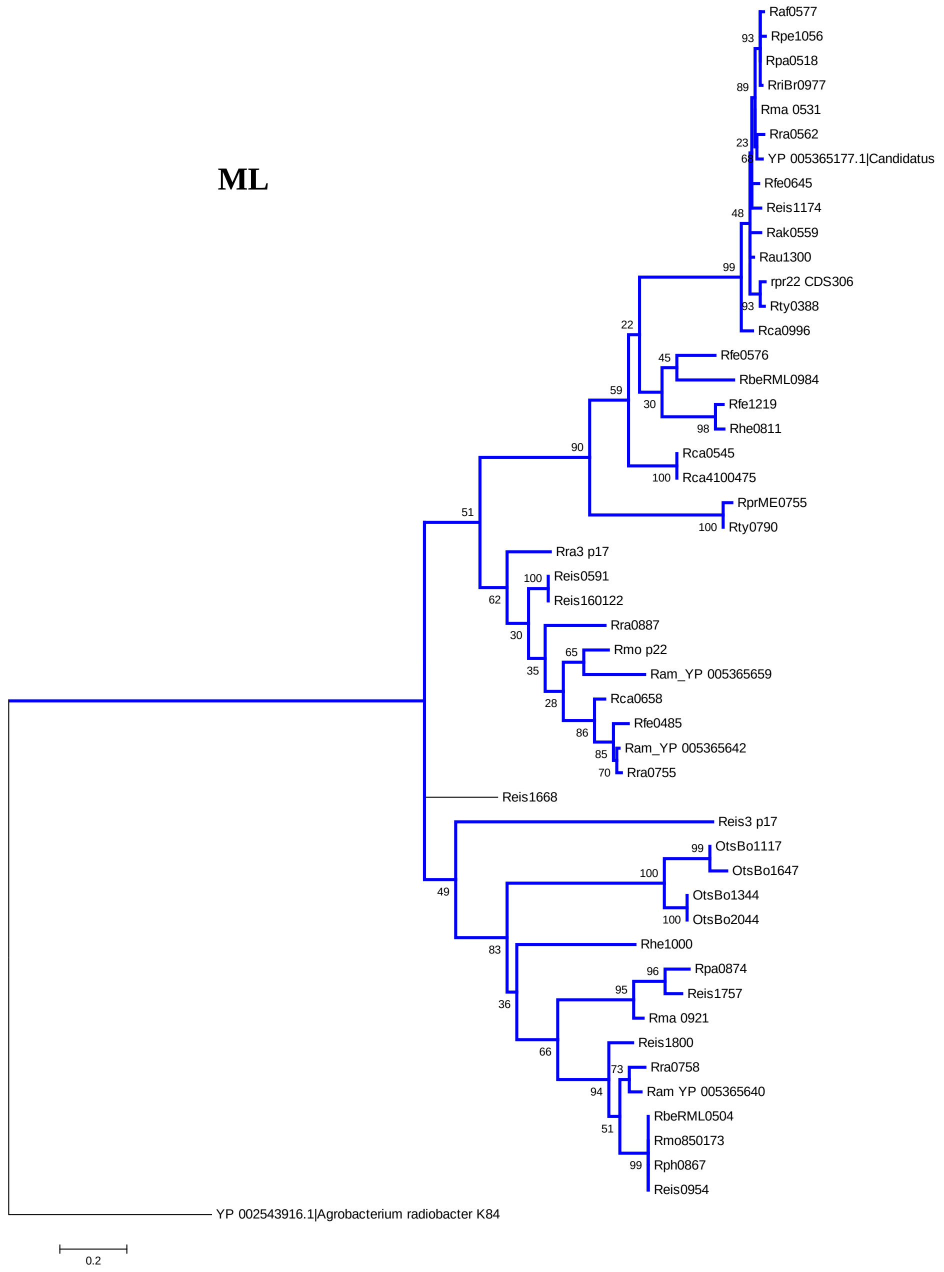

Plasmids and chromosomes

Figure A68. Neighbor-joining (NJ) and maximum likelihood (ML) trees of *hem*K gene. Bootstrap supports higher than or equal to 60% are shown on the branches.

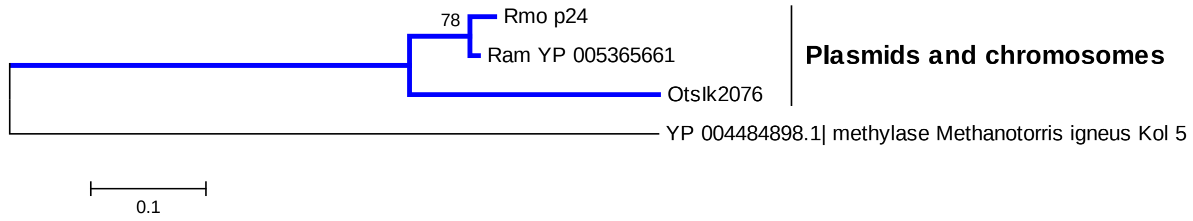

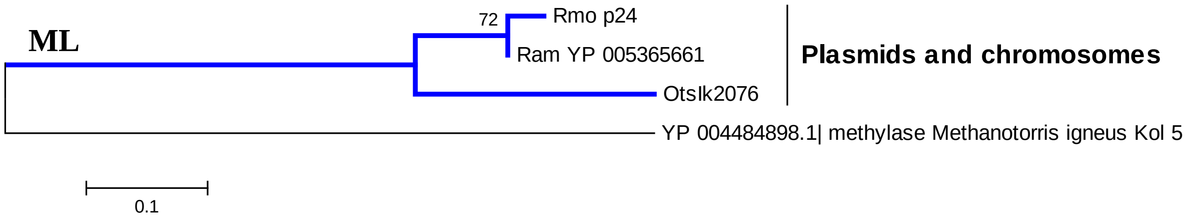
ML
72
Rmo p24
Ram YP 005365661
OtsIk2076
Plasmids and chromosomes
YP 004484898.1| methylase Methanotorris igneus Kol 5
0.1

Figure A69. Neighbor-joining (NJ) and maximum likelihood (ML) trees of deoxyribodipyrimidine photo-lyase. Bootstrap supports higher than or equal to 60% are shown on the branches.

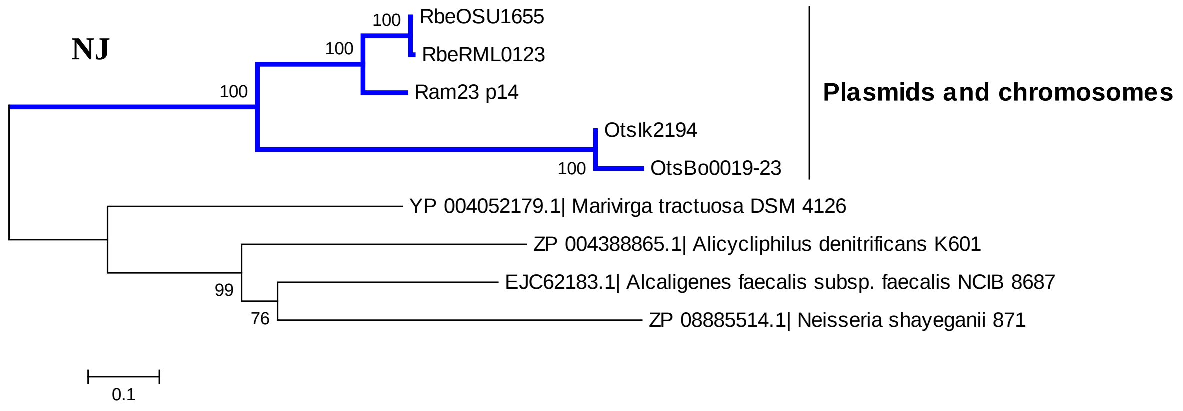

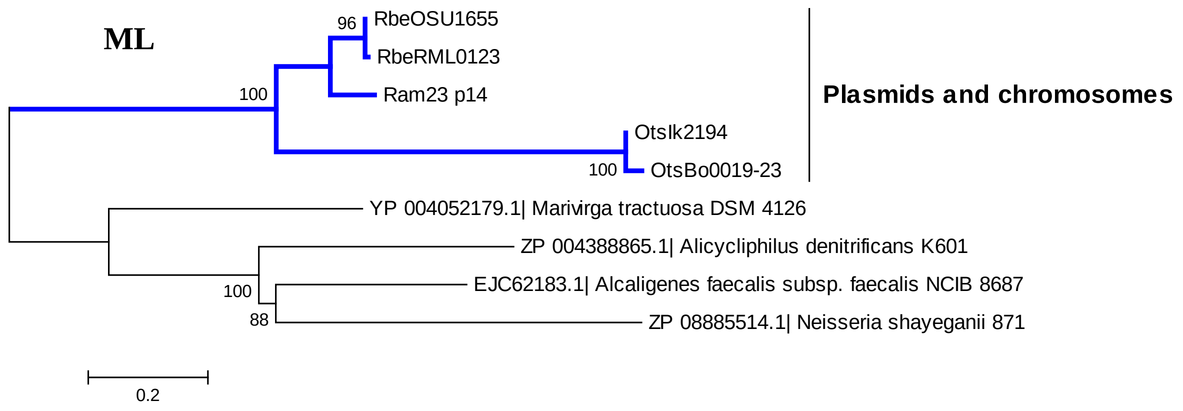

ML
96
RbeOSU1655
RbeRML0123
100
Ram23 p14
OtsIk2194
100
OtsBo0019-23
Plasmids and chromosomes
YP 004052179.1| Marivirga tractuosa DSM 4126
ZP 004388865.1| Alicycliphilus denitrificans K601
100
EJC62183.1| Alcaligenes faecalis subsp. faecalis NCIB 8687
88
ZP 08885514.1| Neisseria shayeganii 871
0.2

Figure A70. Neighbor-joining (NJ) and maximum likelihood (ML) trees of conserved protein of unknown function. Bootstrap supports higher than or equal to 60% are shown on the branches.

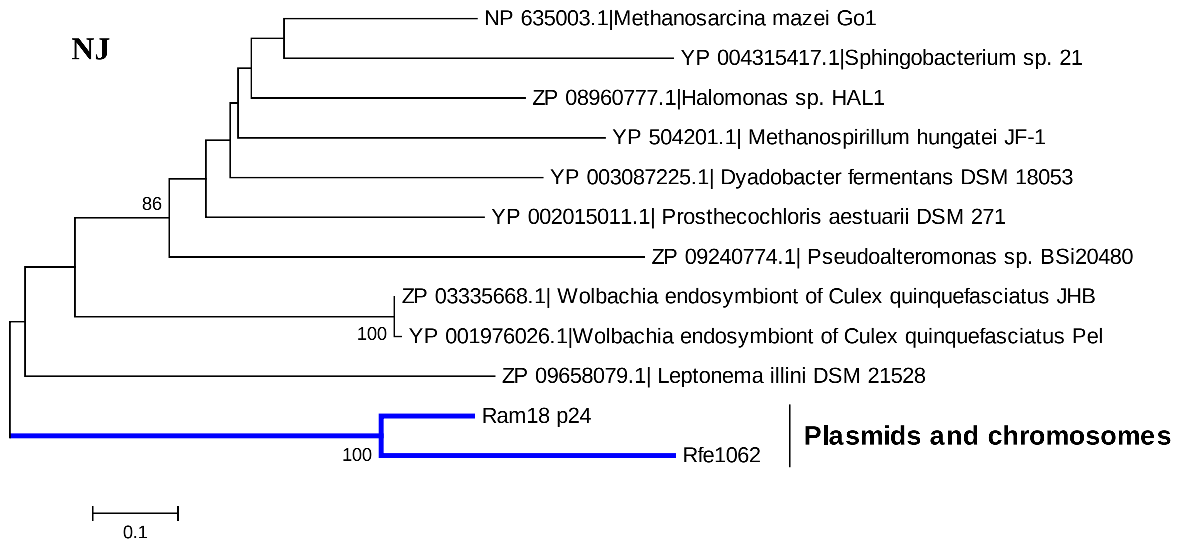

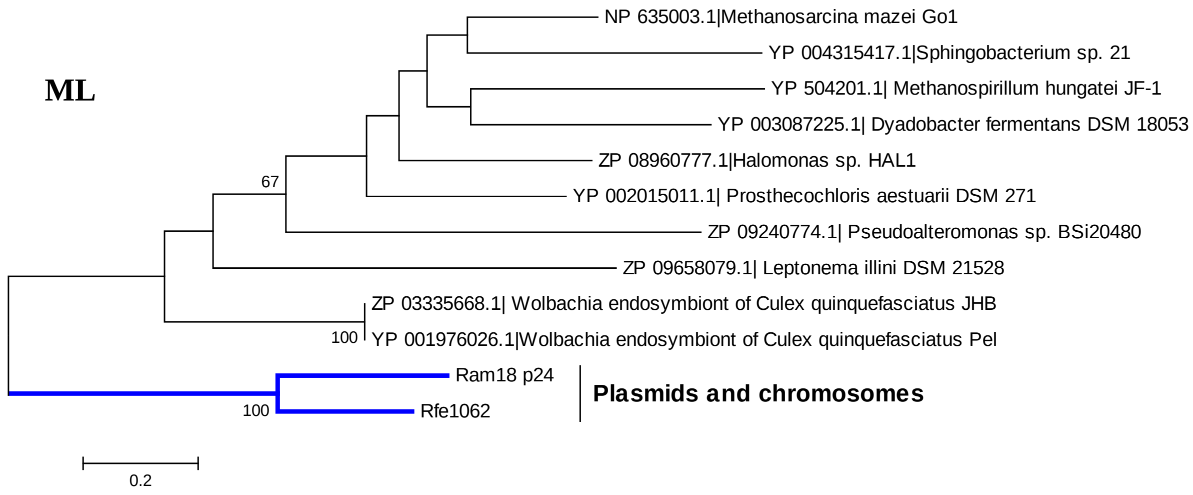

Figure A71. Neighbor-joining (NJ) and maximum likelihood (ML) trees of conserved protein of unknown function. Bootstrap supports higher than or equal to 60% are shown on the branches.

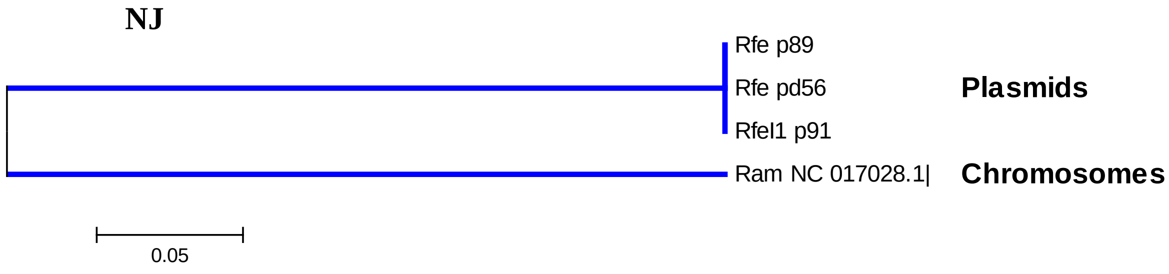

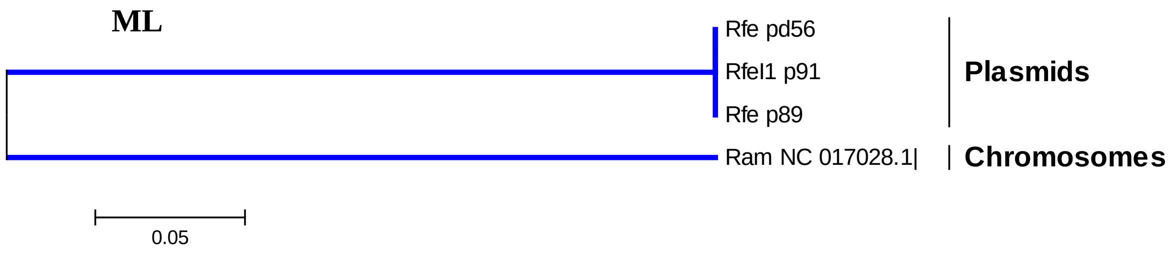

Figure A72. Neighbor-joining (NJ) and maximum likelihood (ML) trees of conserved protein of unknown function. Bootstrap supports higher than or equal to 60% are shown on the branches.

NJ

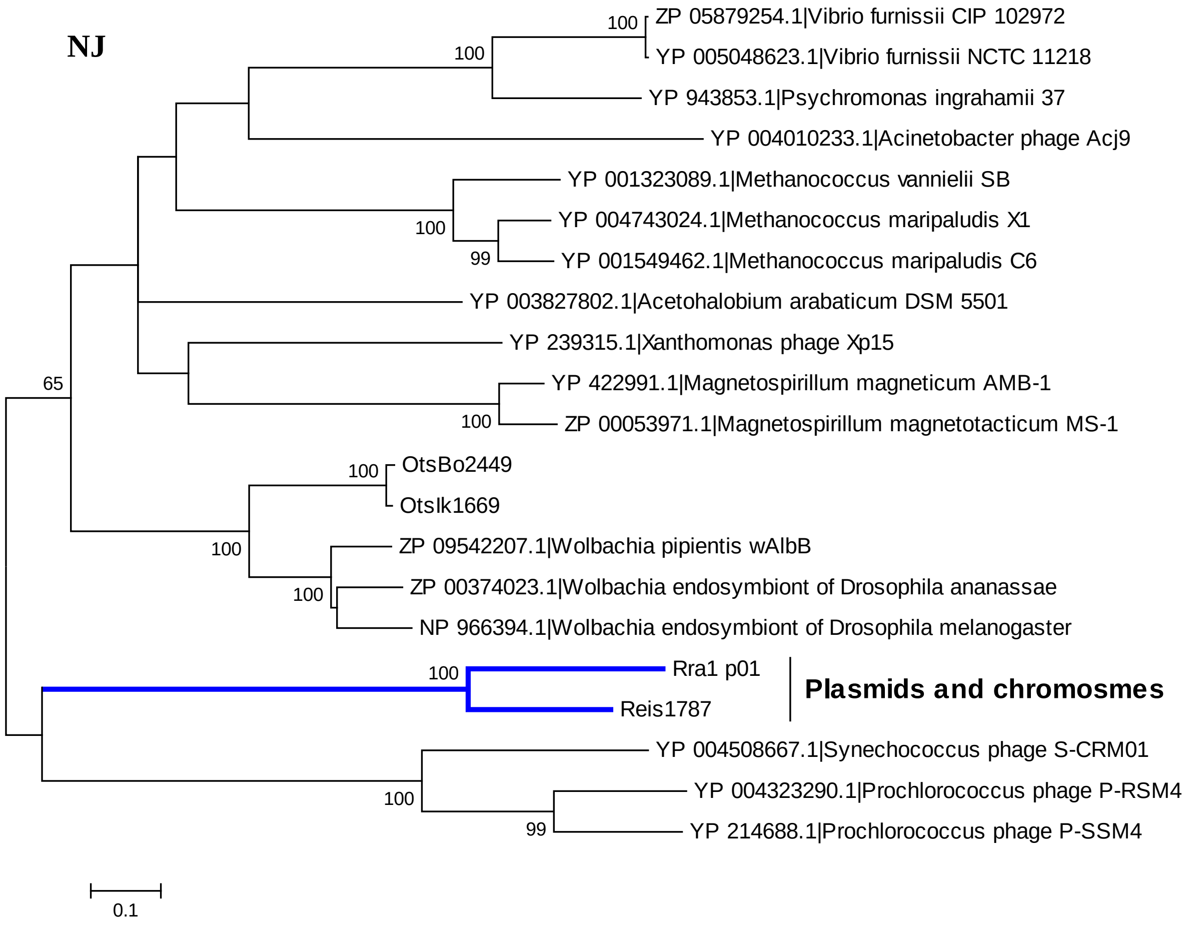

100
ZP 05879254.1|Vibrio furnissii CIP 102972
100
YP 005048623.1|Vibrio furnissii NCTC 11218
YP 943853.1|Psychromonas ingrahamii 37
YP 004010233.1|Acinetobacter phage Acj9
YP 001323089.1|Methanococcus vannielii SB
100
YP 004743024.1|Methanococcus maripaludis X1
99
YP 001549462.1|Methanococcus maripaludis C6
YP 003827802.1|Acetohalobium arabaticum DSM 5501
YP 239315.1|Xanthomonas phage Xp15
65
YP 422991.1|Magnetospirillum magneticum AMB-1
100
ZP 00053971.1|Magnetospirillum magnetotacticum MS-1
100
OtsBo2449
OtsIk1669
100
ZP 09542207.1|Wolbachia pipientis wAlbB
100
ZP 00374023.1|Wolbachia endosymbiont of Drosophila ananassae
NP 966394.1|Wolbachia endosymbiont of Drosophila melanogaster
100
Rra1 p01
Reis1787
Plasmids and chromosmes
YP 004508667.1|Synechococcus phage S-CRM01
100
YP 004323290.1|Prochlorococcus phage P-RSM4
99
YP 214688.1|Prochlorococcus phage P-SSM4
0.1

ML

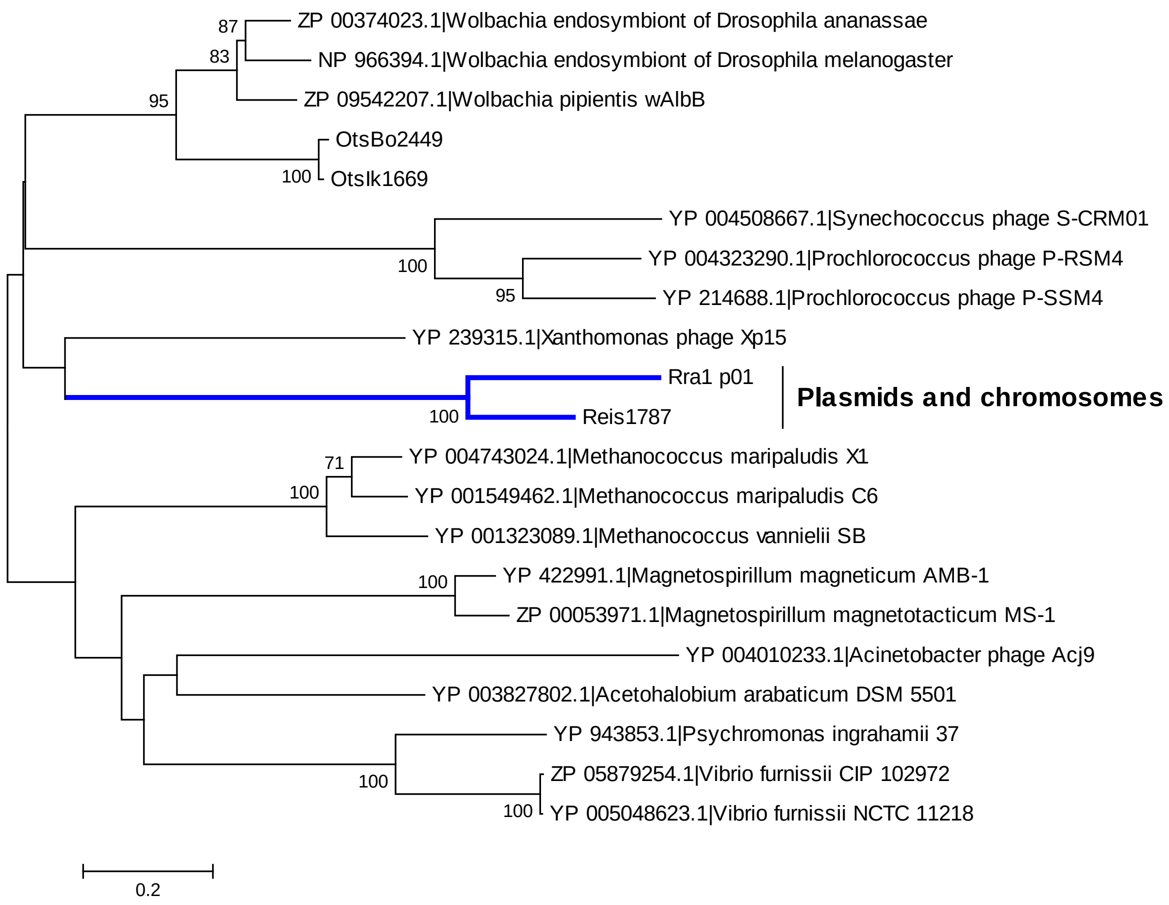

Figure A73. Neighbor-joining (NJ) and maximum likelihood (ML) trees of conserved protein of unknown function. Bootstrap supports higher than or equal to 60% are shown on the branches.

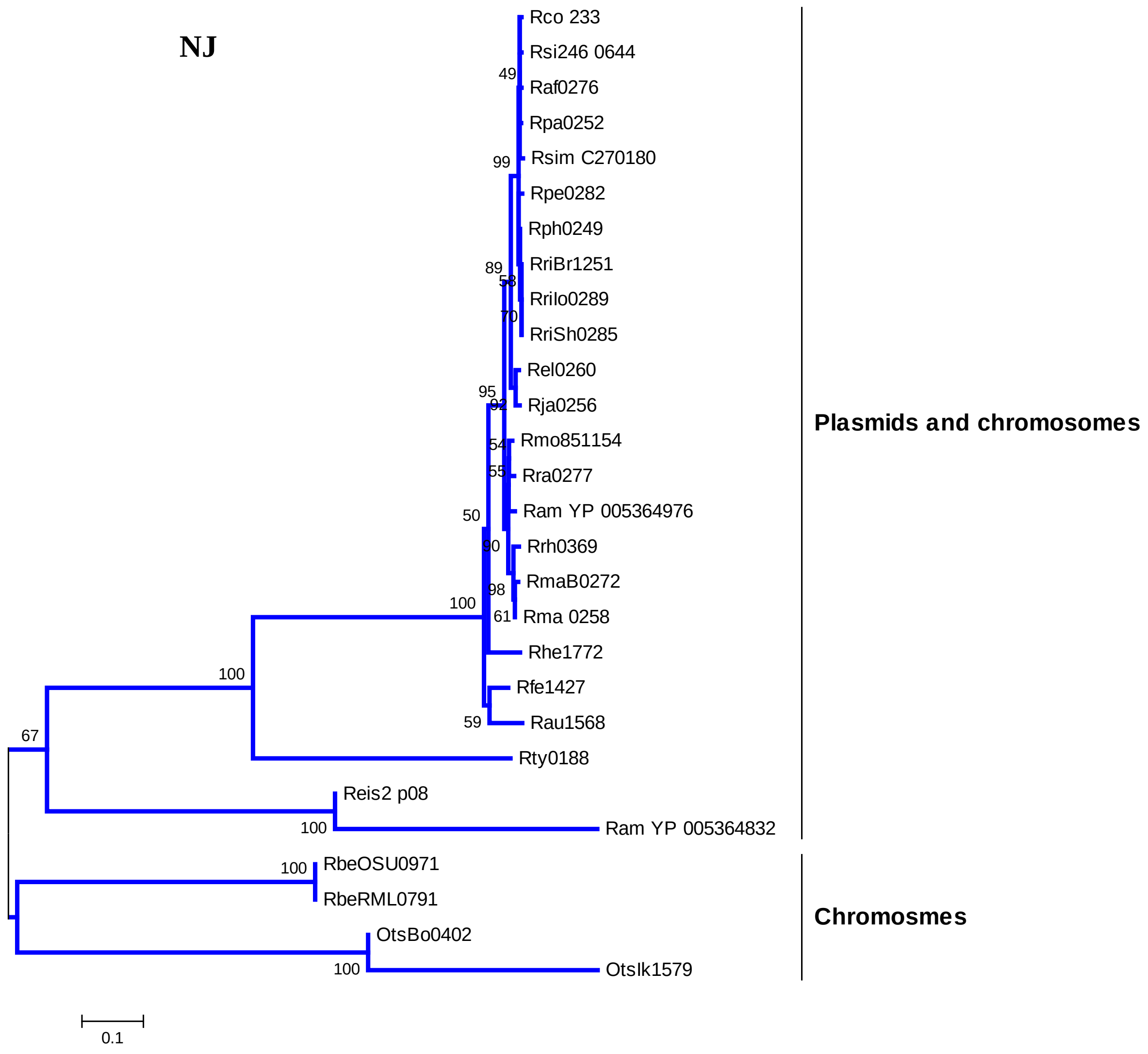
NJ
Rco 233
Rsi246 0644
Raf0276
Rpa0252
Rsim C270180
Rpe0282
Rph0249
RriBr1251
RriIo0289
RriSh0285
Rel0260
Rja0256
Rmo851154
Rra0277
Ram YP 005364976
Rrh0369
RmaB0272
Rma 0258
Rhe1772
Rfe1427
Rau1568
Rty0188
Reis2 p08
Ram YP 005364832
RbeOSU0971
RbeRML0791
OtsBo0402
OtsIk1579
Plasmids and chromosomes
Chromosmes
0.1

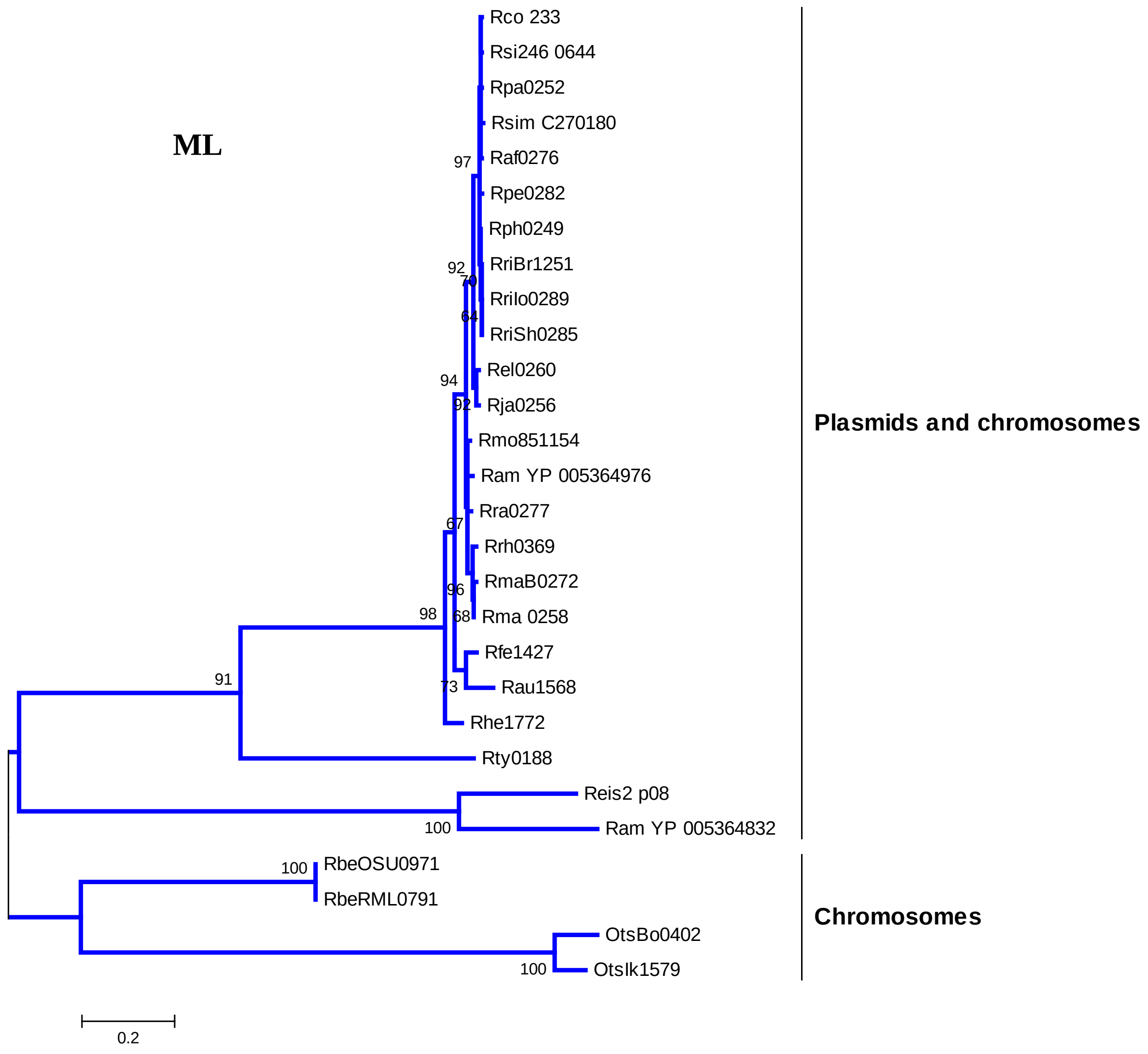
ML
Rco 233
Rsi246 0644
Rpa0252
Rsim C270180
Raf0276
Rpe0282
Rph0249
RriBr1251
RriIo0289
RriSh0285
Rel0260
Rja0256
Rmo851154
Ram YP 005364976
Rra0277
Rrh0369
RmaB0272
Rma 0258
Rfe1427
Rau1568
Rhe1772
Rty0188
Reis2 p08
Ram YP 005364832
RbeOSU0971
RbeRML0791
OtsBo0402
OtsIk1579
97
92
70
64
94
92
67
96
98
68
91
73
100
100
100
0.2
Plasmids and chromosomes
Chromosomes

Figure A74. Neighbor-joining (NJ) and maximum likelihood (ML) trees of transposase_20 IS116/IS110/IS902 family protein containing DEDD_IS110 domain. Bootstrap supports higher than or equal to 60% are shown on the branches.

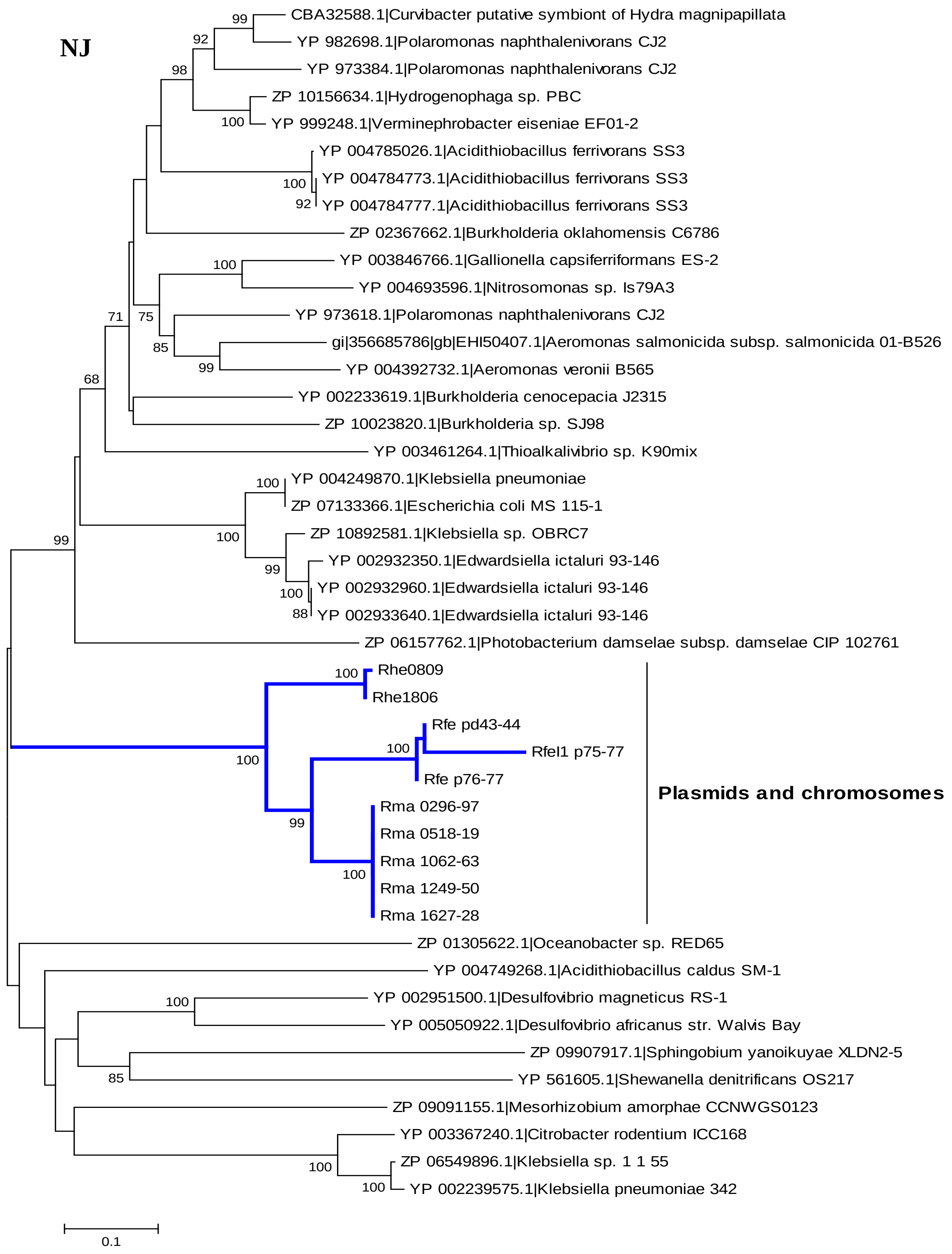

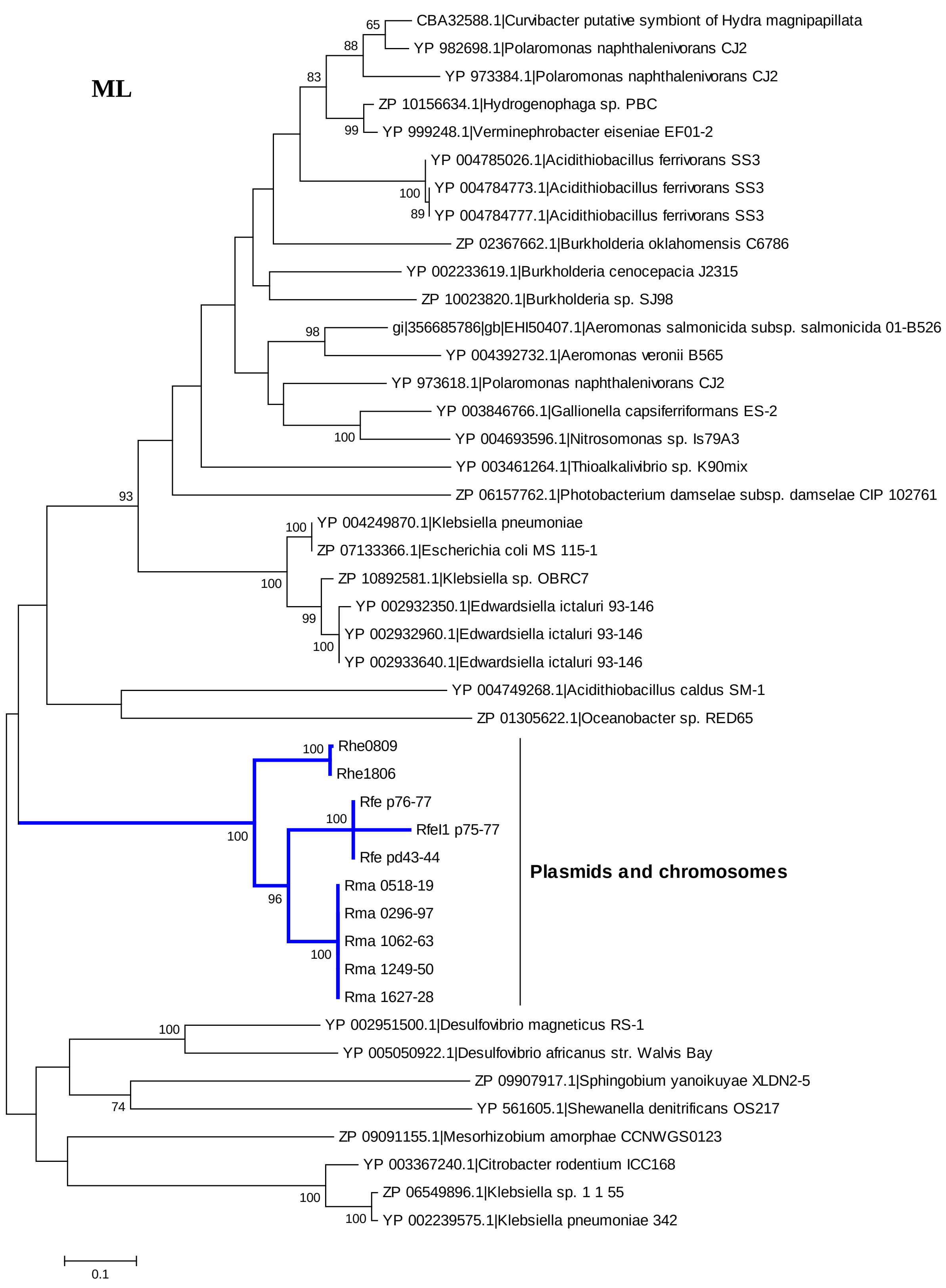
ML
CBA32588.1|Curvibacter putative symbiont of Hydra magnipapillata
YP 982698.1|Polaromonas naphthalenivorans CJ2
YP 973384.1|Polaromonas naphthalenivorans CJ2
ZP 10156634.1|Hydrogenophaga sp. PBC
YP 999248.1|Verminephrobacter eiseniae EF01-2
YP 004785026.1|Acidithiobacillus ferrivorans SS3
YP 004784773.1|Acidithiobacillus ferrivorans SS3
YP 004784777.1|Acidithiobacillus ferrivorans SS3
ZP 02367662.1|Burkholderia oklahomensis C6786
YP 002233619.1|Burkholderia cenocepacia J2315
ZP 10023820.1|Burkholderia sp. SJ98
gi|356685786|gb|EHI50407.1|Aeromonas salmonicida subsp. salmonicida 01-B526
YP 004392732.1|Aeromonas veronii B565
YP 973618.1|Polaromonas naphthalenivorans CJ2
YP 003846766.1|Gallionella capsiferriformans ES-2
YP 004693596.1|Nitrosomonas sp. Is79A3
YP 003461264.1|Thioalkalivibrio sp. K90mix
ZP 06157762.1|Photobacterium damselae subsp. damselae CIP 102761
YP 004249870.1|Klebsiella pneumoniae
ZP 07133366.1|Escherichia coli MS 115-1
ZP 10892581.1|Klebsiella sp. OBRC7
YP 002932350.1|Edwardsiella ictaluri 93-146
YP 002932960.1|Edwardsiella ictaluri 93-146
YP 002933640.1|Edwardsiella ictaluri 93-146
YP 004749268.1|Acidithiobacillus caldus SM-1
ZP 01305622.1|Oceanobacter sp. RED65
Rhe0809
Rhe1806
Rfe p76-77
Rfel1 p75-77
Rfe pd43-44
Rma 0518-19
Rma 0296-97
Rma 1062-63
Rma 1249-50
Rma 1627-28
Plasmids and chromosomes
YP 002951500.1|Desulfovibrio magneticus RS-1
YP 005050922.1|Desulfovibrio africanus str. Walvis Bay
ZP 09907917.1|Sphingobium yanoikuyae XLDN2-5
YP 561605.1|Shewanella denitrificans OS217
ZP 09091155.1|Mesorhizobium amorphae CCNWGS0123
YP 003367240.1|Citrobacter rodentium ICC168
ZP 06549896.1|Klebsiella sp. 1 1 55
YP 002239575.1|Klebsiella pneumoniae 342
65
88
83
99
100
89
98
100
93
100
100
99
100
100
100
100
96
100
100
74
100
100
0.1

Figure A75. Neighbor-joining (NJ) and maximum likelihood (ML) trees of NAD-dependent epimerase/dehydratase family protein. Bootstrap supports higher than or equal to 60% are shown on the branches.

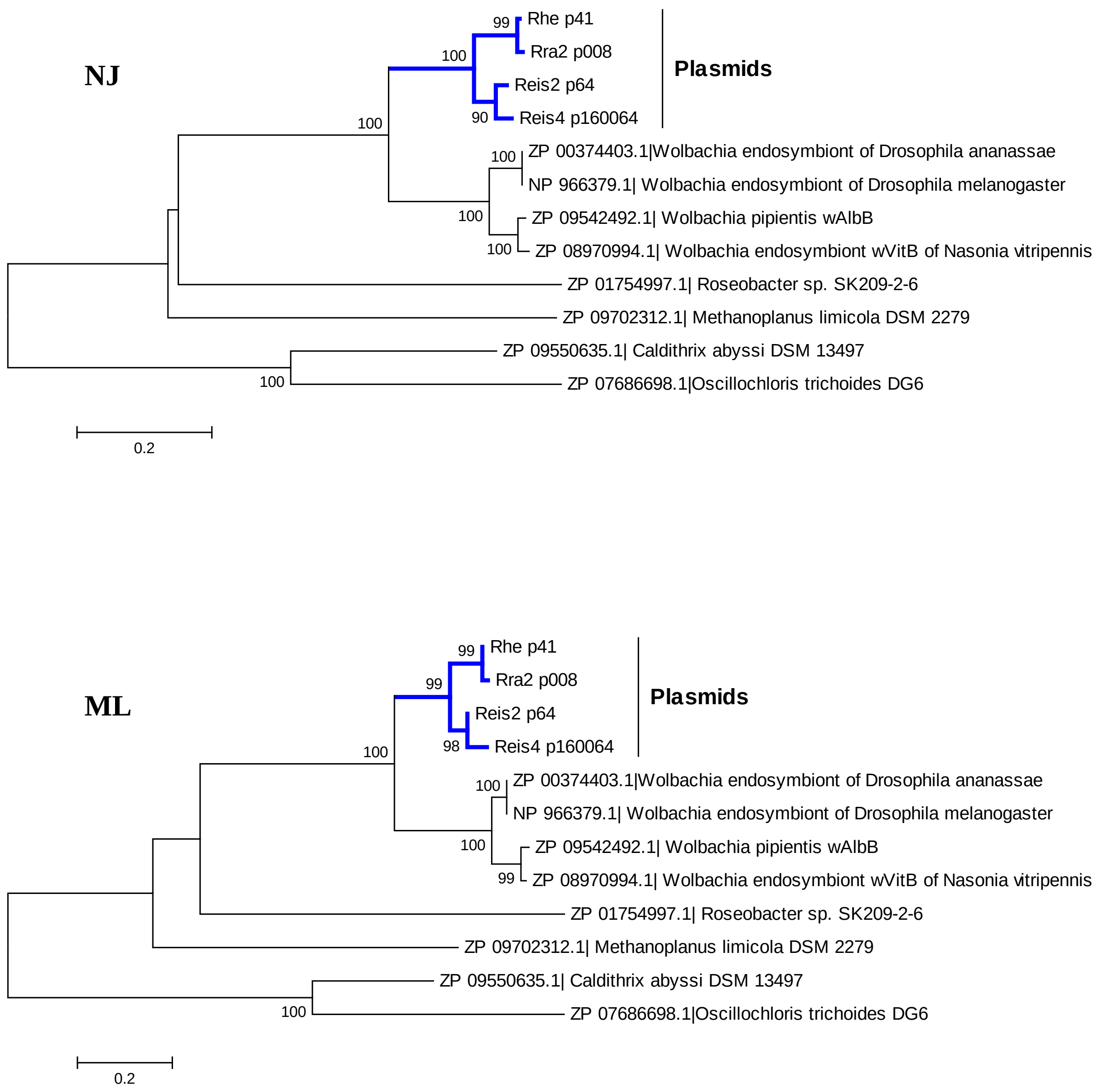

Figure A76. Neighbor-joining (NJ) and maximum likelihood (ML) trees of glycosyltransferase, group 1 family protein. Bootstrap supports higher than or equal to 60% are shown on the branches.

NJ

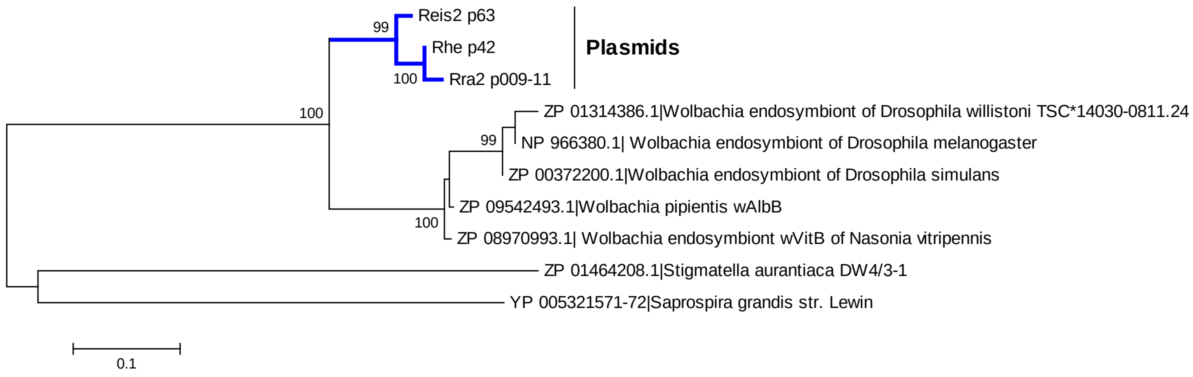

ML

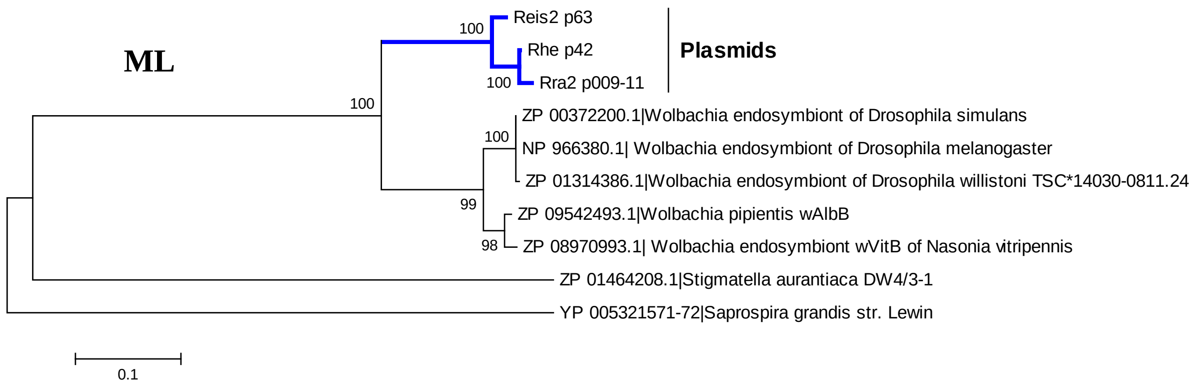

Figure A77. Neighbor-joining (NJ) and maximum likelihood (ML) trees of conserved protein of unknown function. Bootstrap supports higher than or equal to 60% are shown on the branches.

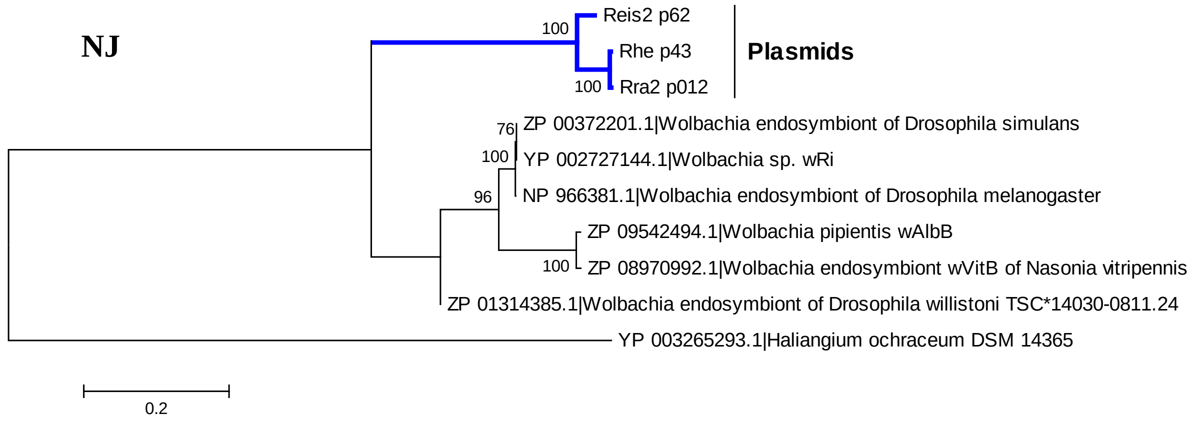

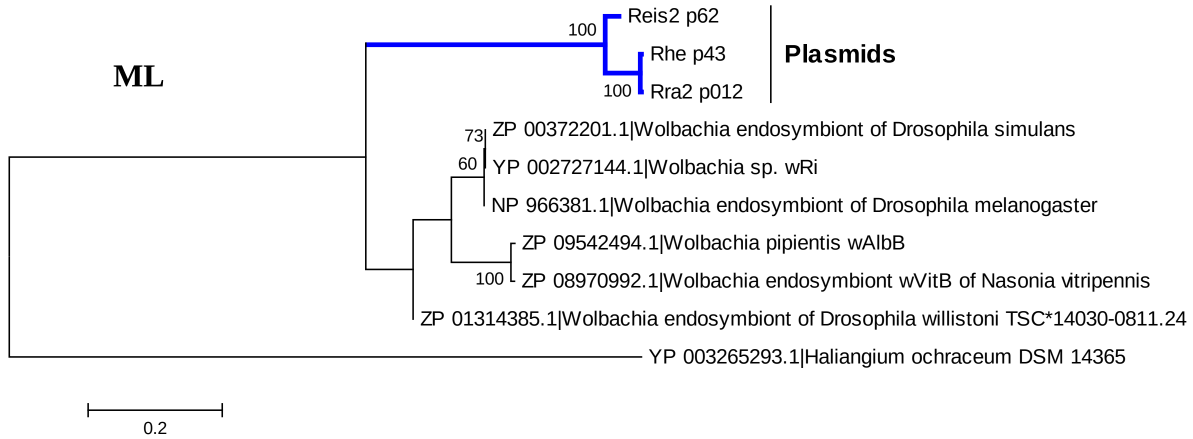

Figure A78. Neighbor-joining (NJ) and maximum likelihood (ML) trees of phytanoyl-CoA dioxygenase family protein. Bootstrap supports higher than or equal to 60% are shown on the branches.

NJ

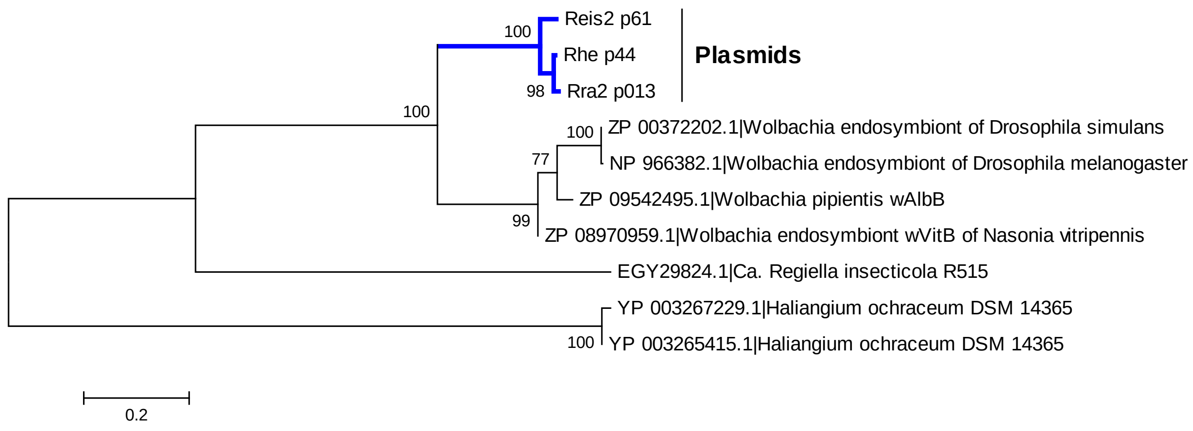

ML

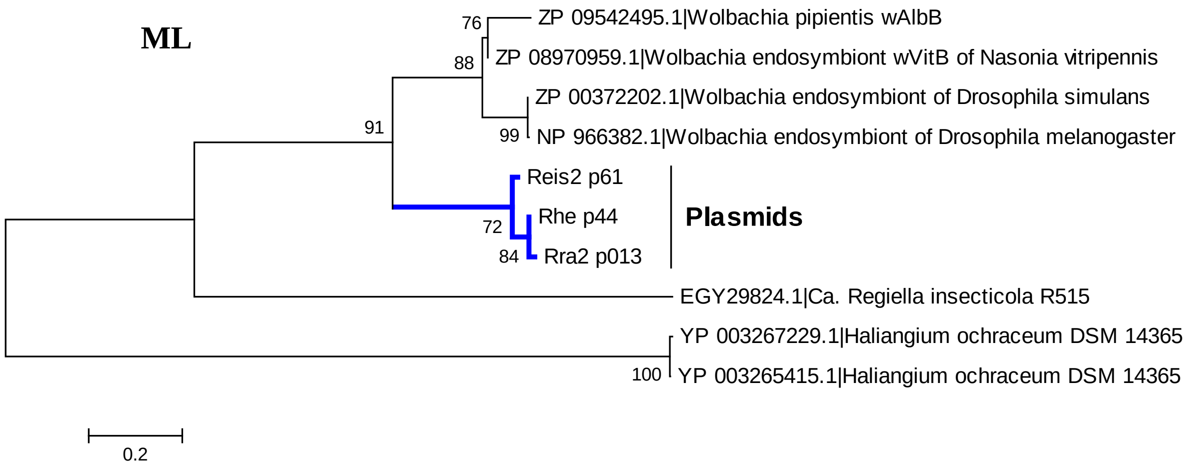

Figure A79. Neighbor-joining (NJ) and maximum likelihood (ML) trees of L-allo-threonine aldolase. Bootstrap supports higher than or equal to 60% are shown on the branches.

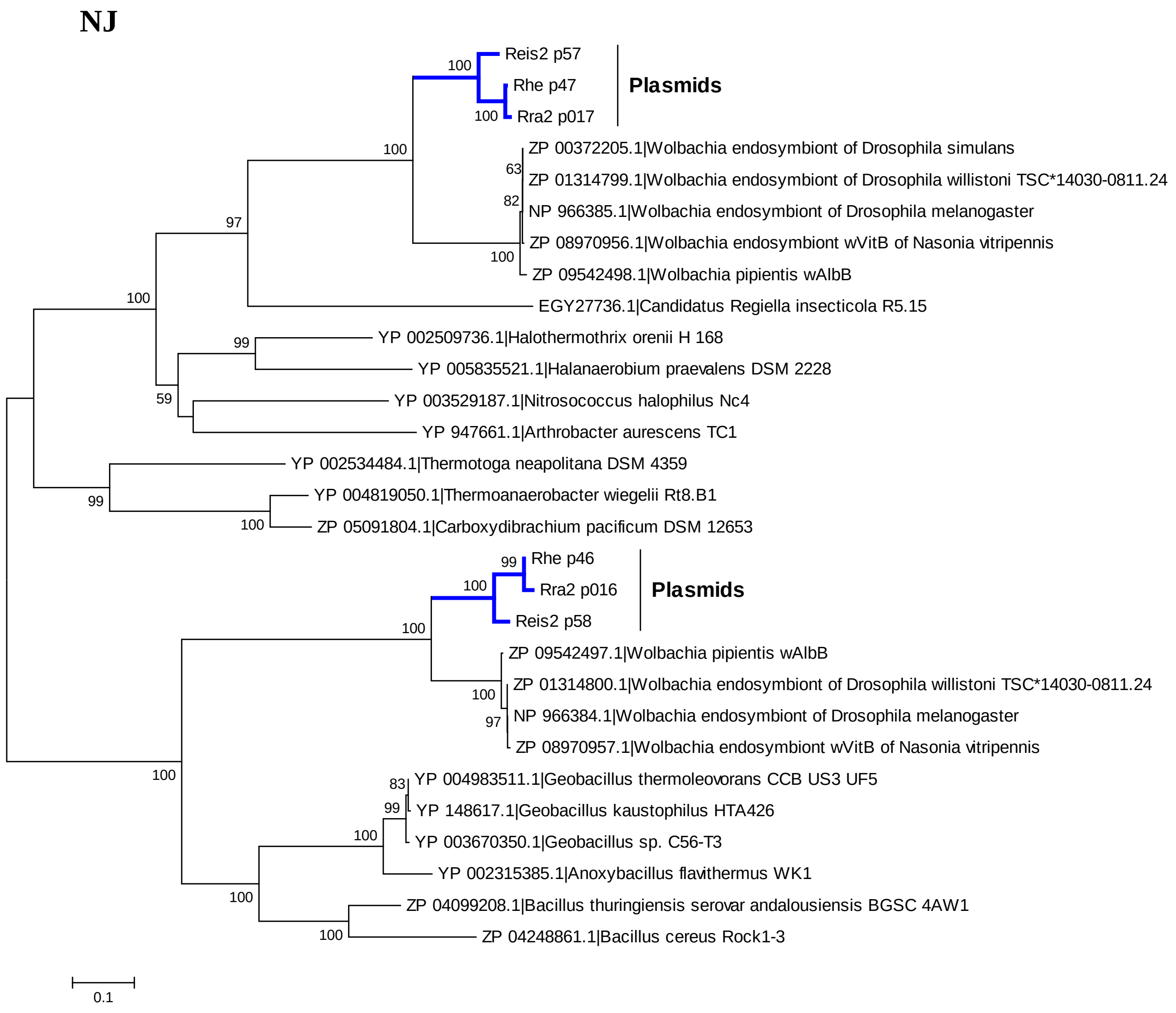

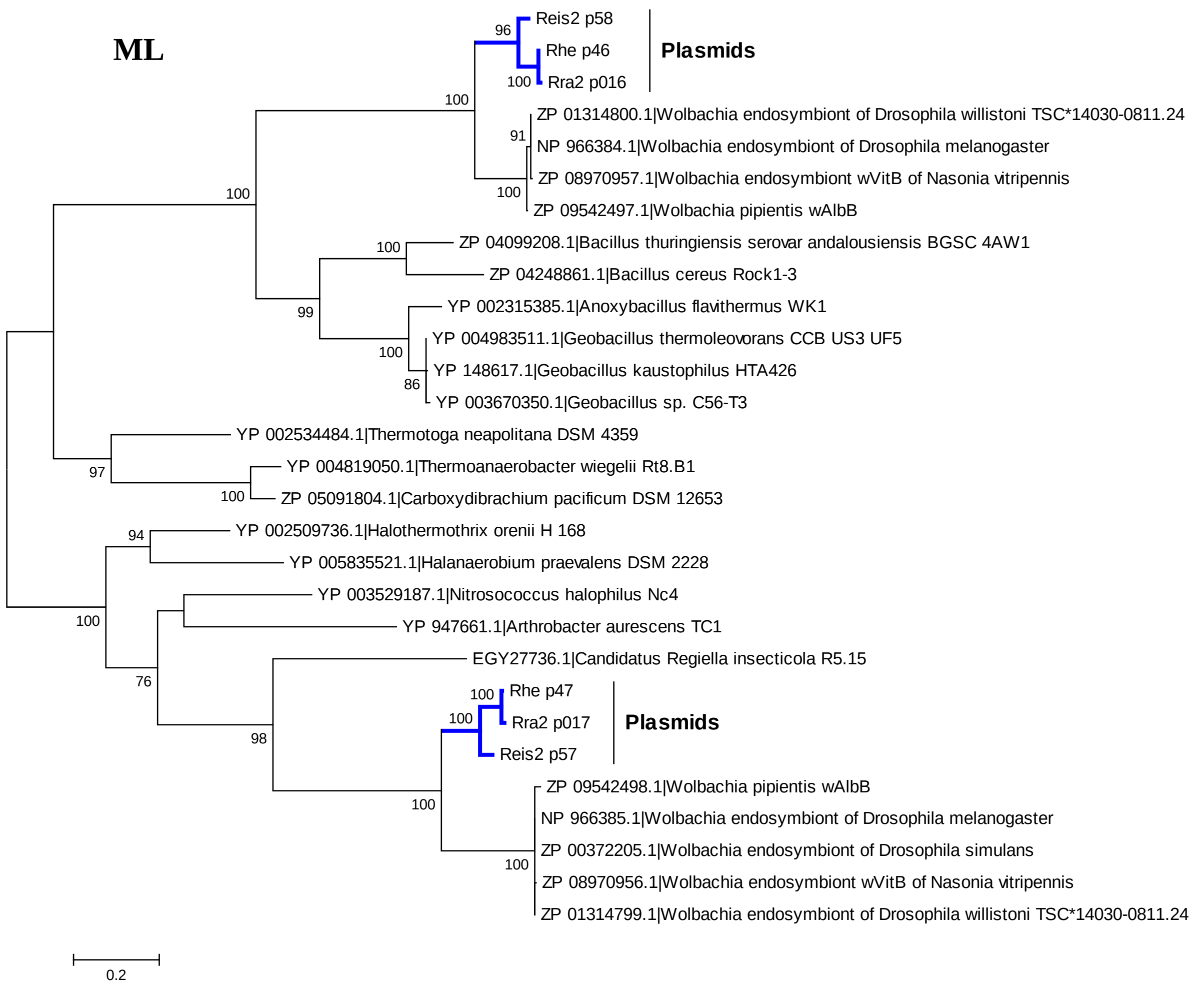
ML
Reis2 p58
Rhe p46
Rra2 p016
Plasmids
ZP 01314800.1|Wolbachia endosymbiont of Drosophila willistoni TSC*14030-0811.24
NP 966384.1|Wolbachia endosymbiont of Drosophila melanogaster
ZP 08970957.1|Wolbachia endosymbiont wVitB of Nasonia vitripennis
ZP 09542497.1|Wolbachia pipientis wAlbB
ZP 04099208.1|Bacillus thuringiensis serovar andalousiensis BGSC 4AW1
ZP 04248861.1|Bacillus cereus Rock1-3
YP 002315385.1|Anoxybacillus flavithermus WK1
YP 004983511.1|Geobacillus thermoleovorans CCB US3 UF5
YP 148617.1|Geobacillus kaustophilus HTA426
YP 003670350.1|Geobacillus sp. C56-T3
YP 002534484.1|Thermotoga neapolitana DSM 4359
YP 004819050.1|Thermoanaerobacter wiegelii Rt8.B1
ZP 05091804.1|Carboxydibrachium pacificum DSM 12653
YP 002509736.1|Halothermothrix orenii H 168
YP 005835521.1|Halanaerobium praevalens DSM 2228
YP 003529187.1|Nitrosococcus halophilus Nc4
YP 947661.1|Arthrobacter aurescens TC1
EGY27736.1|Candidatus Regiella insecticola R5.15
Rhe p47
Rra2 p017
Reis2 p57
Plasmids
ZP 09542498.1|Wolbachia pipientis wAlbB
NP 966385.1|Wolbachia endosymbiont of Drosophila melanogaster
ZP 00372205.1|Wolbachia endosymbiont of Drosophila simulans
ZP 08970956.1|Wolbachia endosymbiont wVitB of Nasonia vitripennis
ZP 01314799.1|Wolbachia endosymbiont of Drosophila willistoni TSC*14030-0811.24
96
100
100
91
100
100
100
99
100
100
86
97
100
94
100
76
98
100
100
100
100
0.2

Figure A80. Neighbor-joining (NJ) and maximum likelihood (ML) trees of ABC multidrug transporter, permease/ATP-binding protein. Bootstrap supports higher than or equal to 60% are shown on the branches.

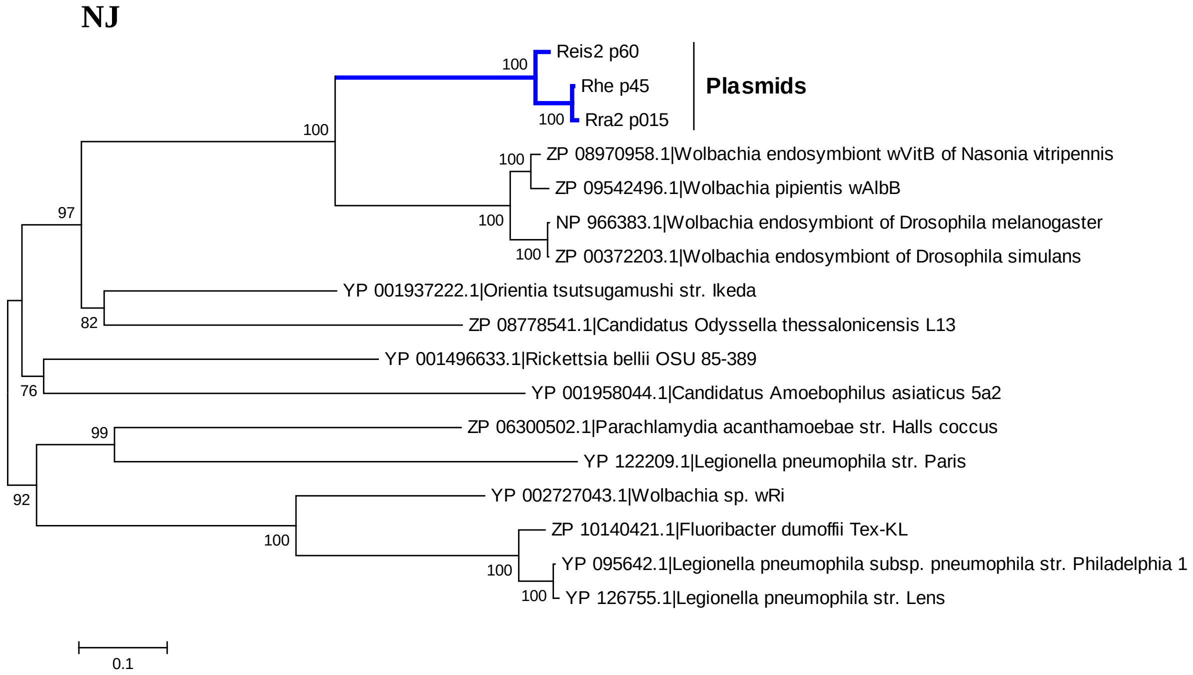

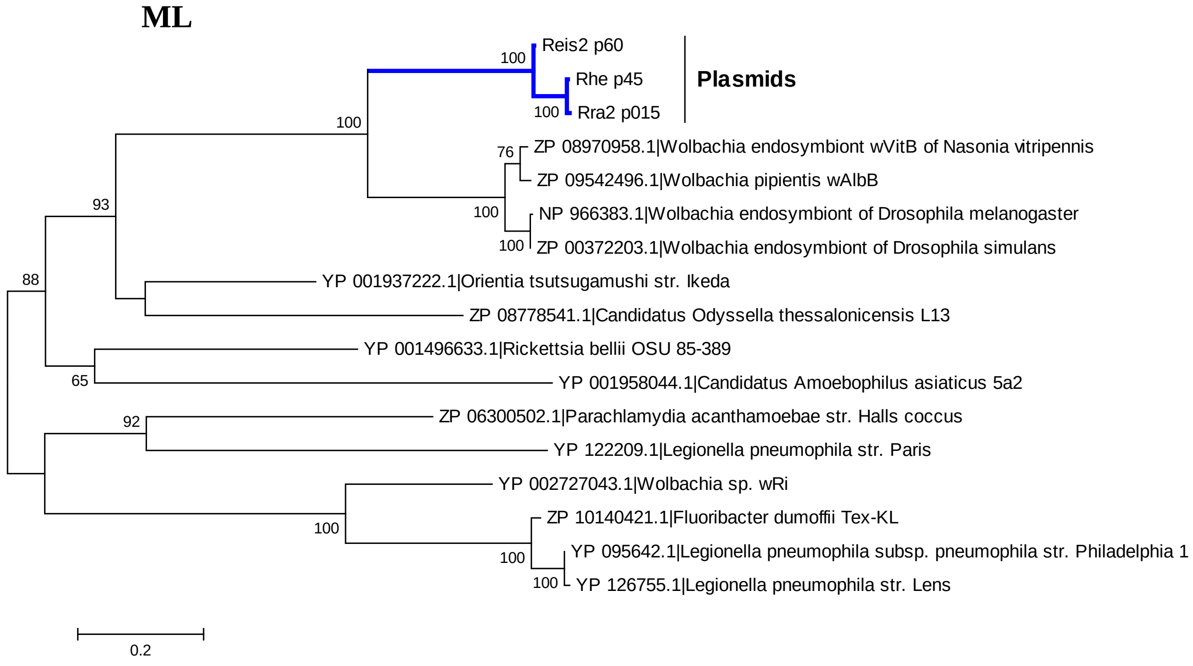

Figure A81. Neighbor-joining (NJ) and maximum likelihood (ML) trees of transaminase BioA gene. Bootstrap supports higher than or equal to 60% are shown on the branches.

NJ

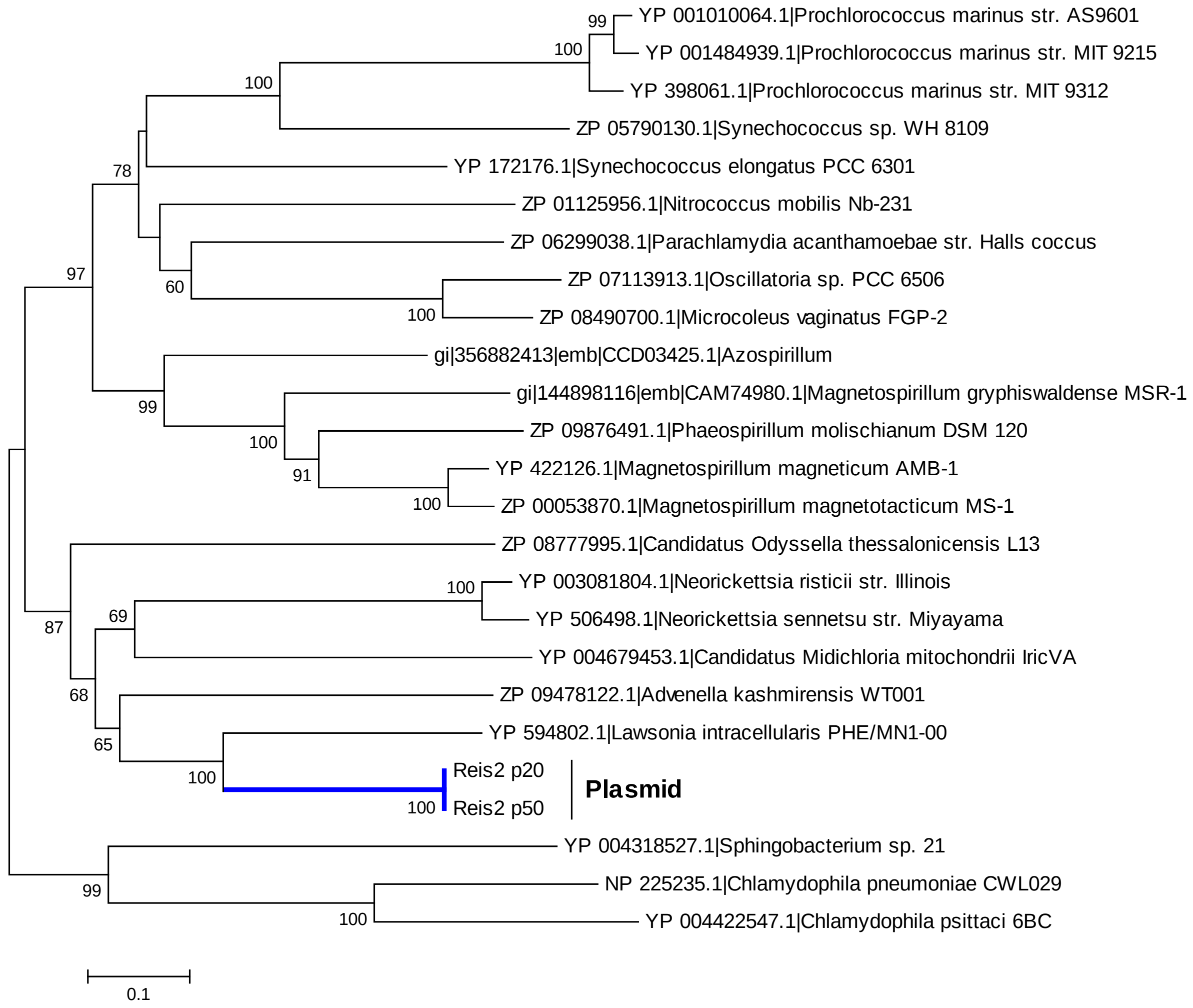

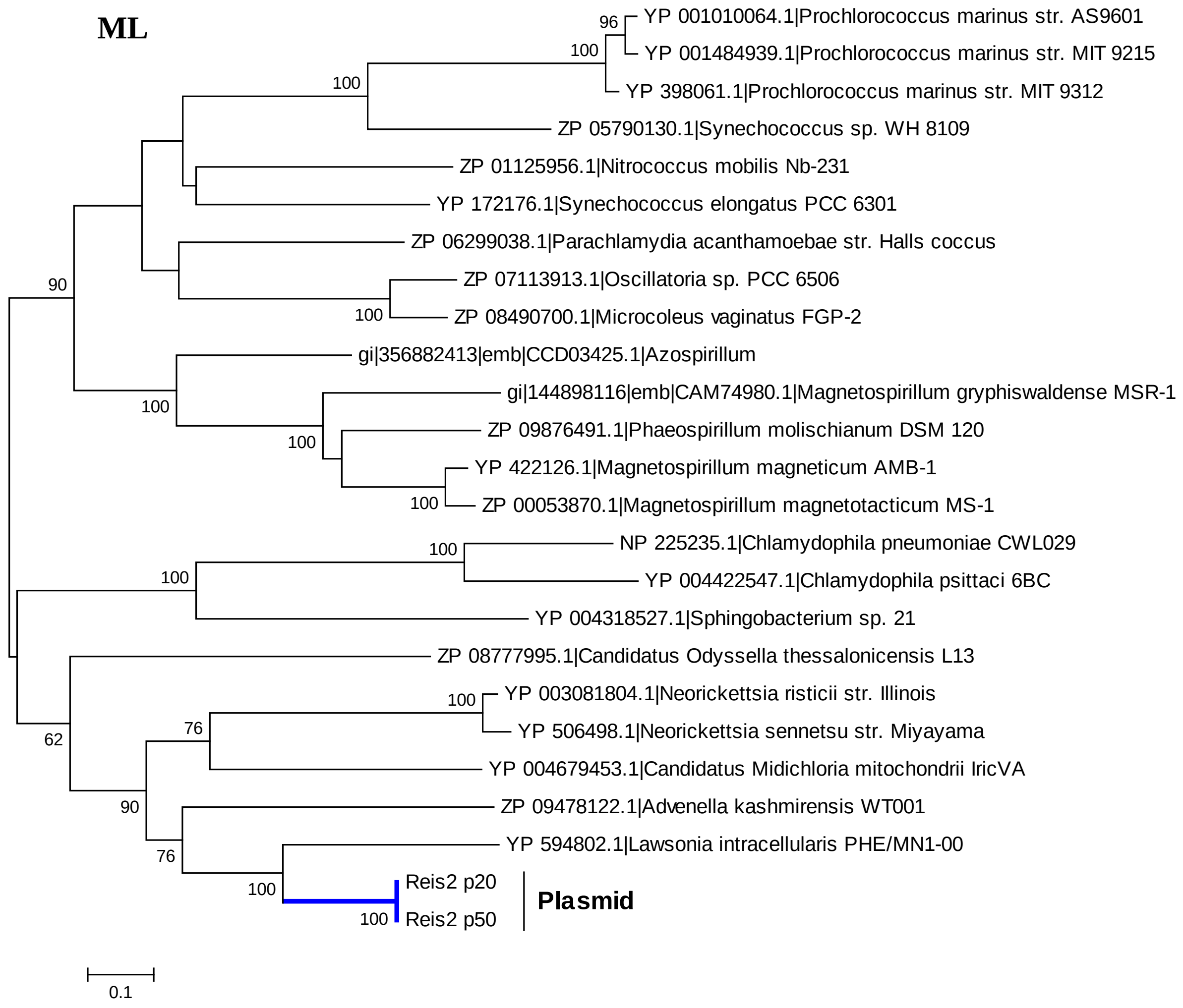
ML
YP 001010064.1|Prochlorococcus marinus str. AS9601
YP 001484939.1|Prochlorococcus marinus str. MIT 9215
YP 398061.1|Prochlorococcus marinus str. MIT 9312
ZP 05790130.1|Synechococcus sp. WH 8109
ZP 01125956.1|Nitrococcus mobilis Nb-231
YP 172176.1|Synechococcus elongatus PCC 6301
ZP 06299038.1|Parachlamydia acanthamoebae str. Halls coccus
ZP 07113913.1|Oscillatoria sp. PCC 6506
ZP 08490700.1|Microcoleus vaginatus FGP-2
gi|356882413|emb|CCD03425.1|Azospirillum
gi|144898116|emb|CAM74980.1|Magnetospirillum gryphiswaldense MSR-1
ZP 09876491.1|Phaeospirillum molischianum DSM 120
YP 422126.1|Magnetospirillum magneticum AMB-1
ZP 00053870.1|Magnetospirillum magnetotacticum MS-1
NP 225235.1|Chlamydophila pneumoniae CWL029
YP 004422547.1|Chlamydophila psittaci 6BC
YP 004318527.1|Sphingobacterium sp. 21
ZP 08777995.1|Candidatus Odyssella thessalonicensis L13
YP 003081804.1|Neorickettsia risticii str. Illinois
YP 506498.1|Neorickettsia sennetsu str. Miyayama
YP 004679453.1|Candidatus Midichloria mitochondrii IricVA
ZP 09478122.1|Advenella kashmirensis WT001
YP 594802.1|Lawsonia intracellularis PHE/MN1-00
Reis2 p20
Reis2 p50
Plasmid
0.1

Figure A82. Neighbor-joining (NJ) and maximum likelihood (ML) trees of dethiobiotin synthase BioD gene. Bootstrap supports higher than or equal to 60% are shown on the branches.

NJ

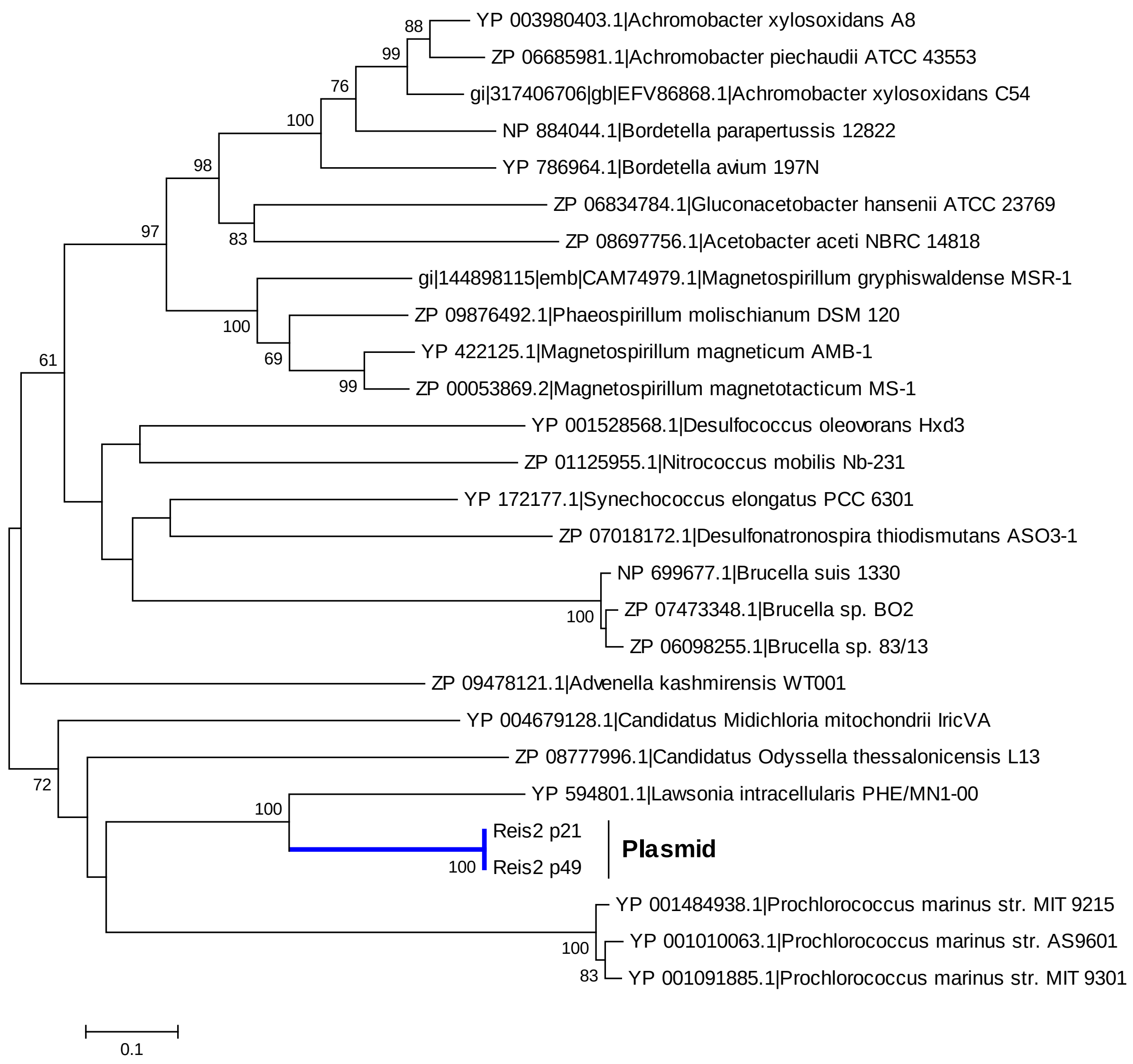

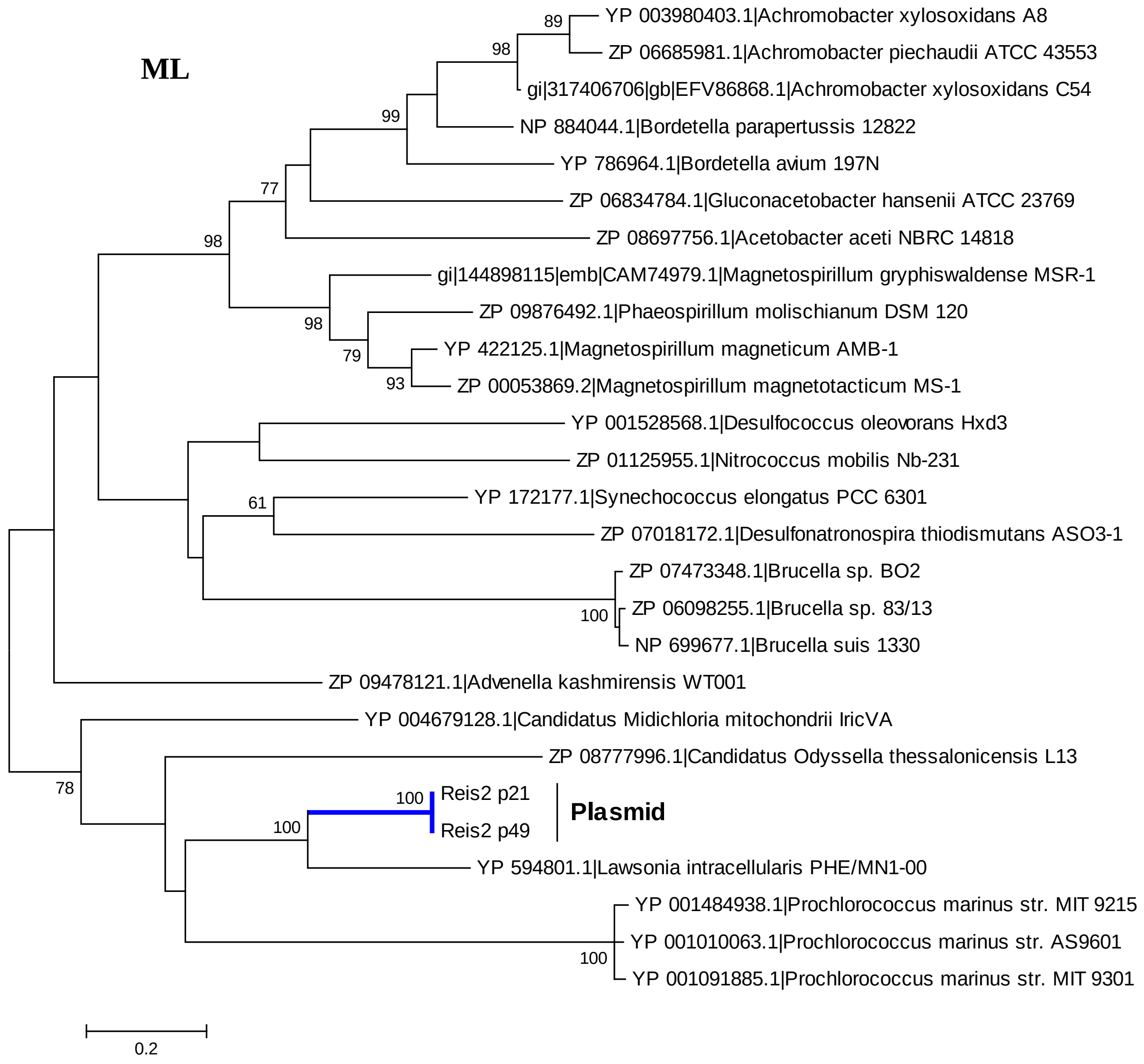
ML
YP 003980403.1|Achromobacter xylosoxidans A8
ZP 06685981.1|Achromobacter piechaudii ATCC 43553
gi|317406706|gb|EFV86868.1|Achromobacter xylosoxidans C54
NP 884044.1|Bordetella parapertussis 12822
YP 786964.1|Bordetella avium 197N
ZP 06834784.1|Gluconacetobacter hansenii ATCC 23769
ZP 08697756.1|Acetobacter aceti NBRC 14818
gi|144898115|emb|CAM74979.1|Magnetospirillum gryphiswaldense MSR-1
ZP 09876492.1|Phaeospirillum molischianum DSM 120
YP 422125.1|Magnetospirillum magneticum AMB-1
ZP 00053869.2|Magnetospirillum magnetotacticum MS-1
YP 001528568.1|Desulfococcus oleovorans Hxd3
ZP 01125955.1|Nitrococcus mobilis Nb-231
YP 172177.1|Synechococcus elongatus PCC 6301
ZP 07018172.1|Desulfonatronospira thiodismutans ASO3-1
ZP 07473348.1|Brucella sp. BO2
ZP 06098255.1|Brucella sp. 83/13
NP 699677.1|Brucella suis 1330
ZP 09478121.1|Advenella kashmirensis WT001
YP 004679128.1|Candidatus Midichloria mitochondrii IricVA
ZP 08777996.1|Candidatus Odyssella thessalonicensis L13
Reis2 p21
Reis2 p49
Plasmid
YP 594801.1|Lawsonia intracellularis PHE/MN1-00
YP 001484938.1|Prochlorococcus marinus str. MIT 9215
YP 001010063.1|Prochlorococcus marinus str. AS9601
YP 001091885.1|Prochlorococcus marinus str. MIT 9301
89
98
99
77
98
98
79
93
61
100
78
100
100
100
0.2

Figure A83. Neighbor-joining (NJ) and maximum likelihood (ML) trees of dethiobiotin synthase BioC gene. Bootstrap supports higher than or equal to 60% are shown on the branches.

NJ

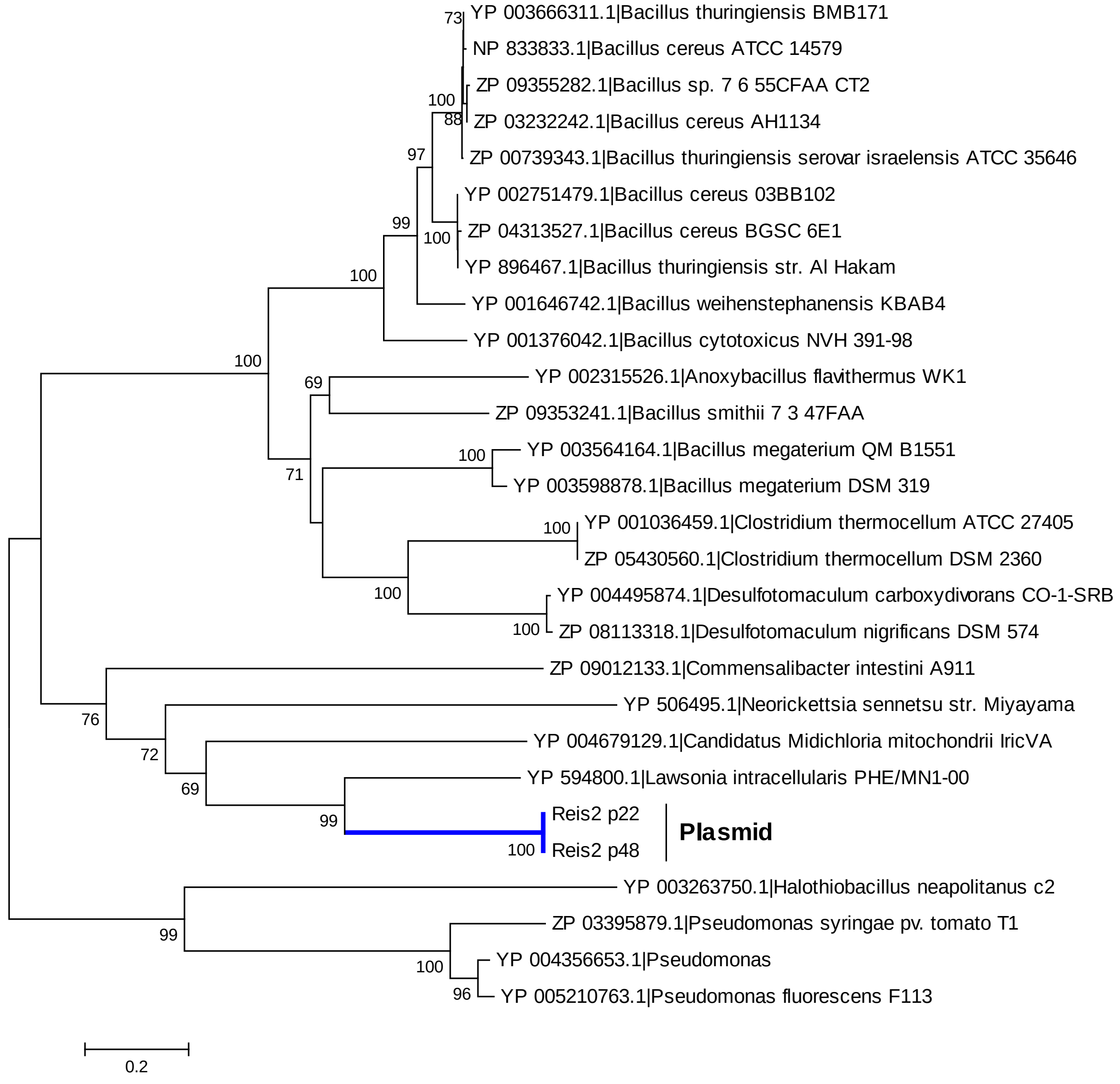

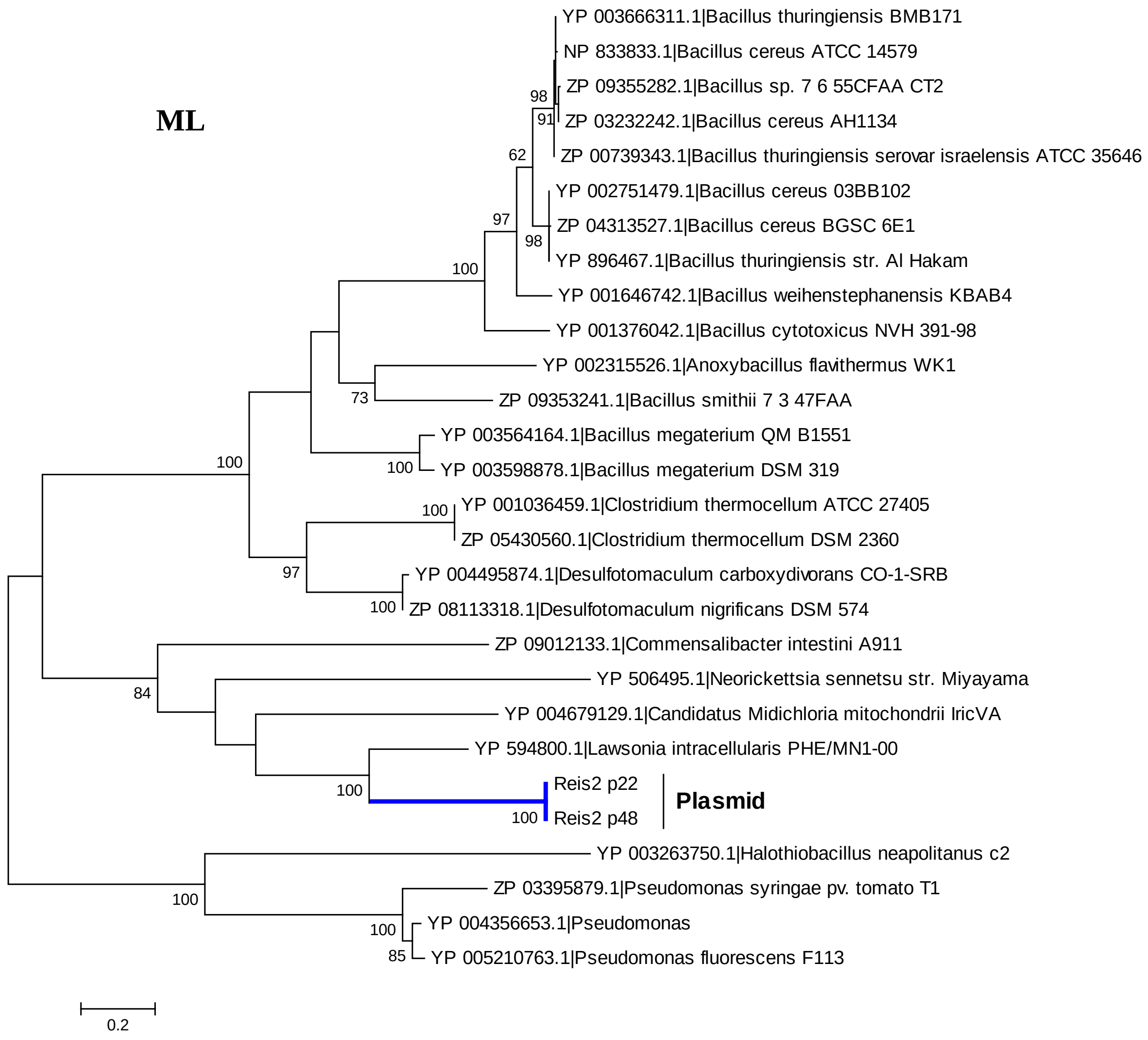
ML
YP 003666311.1|Bacillus thuringiensis BMB171
NP 833833.1|Bacillus cereus ATCC 14579
ZP 09355282.1|Bacillus sp. 7 6 55CFAA CT2
ZP 03232242.1|Bacillus cereus AH1134
ZP 00739343.1|Bacillus thuringiensis serovar israelensis ATCC 35646
YP 002751479.1|Bacillus cereus 03BB102
ZP 04313527.1|Bacillus cereus BGSC 6E1
YP 896467.1|Bacillus thuringiensis str. Al Hakam
YP 001646742.1|Bacillus weihenstephanensis KBAB4
YP 001376042.1|Bacillus cytotoxicus NVH 391-98
YP 002315526.1|Anoxybacillus flavithermus WK1
ZP 09353241.1|Bacillus smithii 7 3 47FAA
YP 003564164.1|Bacillus megaterium QM B1551
YP 003598878.1|Bacillus megaterium DSM 319
YP 001036459.1|Clostridium thermocellum ATCC 27405
ZP 05430560.1|Clostridium thermocellum DSM 2360
YP 004495874.1|Desulfotomaculum carboxydivorans CO-1-SRB
ZP 08113318.1|Desulfotomaculum nigrificans DSM 574
ZP 09012133.1|Commensalibacter intestini A911
YP 506495.1|Neorickettsia sennetsu str. Miyayama
YP 004679129.1|Candidatus Midichloria mitochondrii IricVA
YP 594800.1|Lawsonia intracellularis PHE/MN1-00
Reis2 p22
Reis2 p48
Plasmid
YP 003263750.1|Halothiobacillus neapolitanus c2
ZP 03395879.1|Pseudomonas syringae pv. tomato T1
YP 004356653.1|Pseudomonas
YP 005210763.1|Pseudomonas fluorescens F113
98
91
62
97
98
100
73
100
100
100
97
100
84
100
100
100
100
85
0.2

Figure A84. Neighbor-joining (NJ) and maximum likelihood (ML) trees of alpha/beta hydrolase family protein. Bootstrap supports higher than or equal to 60% are shown on the branches.

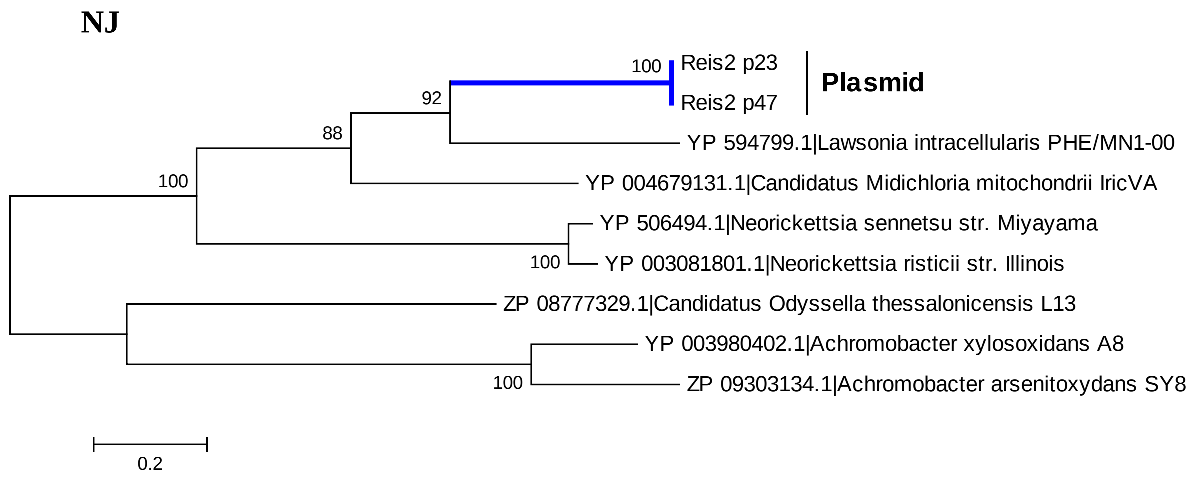

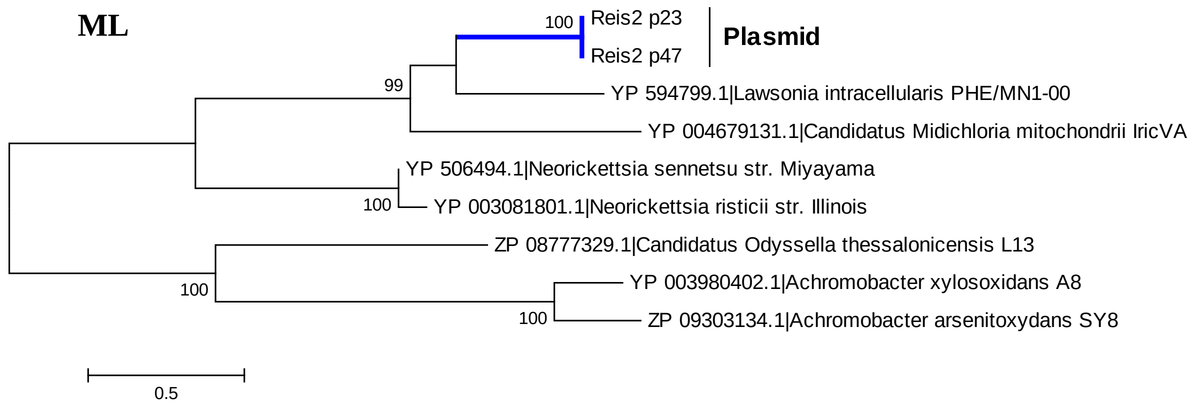

Figure A85. Neighbor-joining (NJ) and maximum likelihood (ML) trees of synthetase and related enzymes BioF gene. Bootstrap supports higher than or equal to 60% are shown on the branches.

NJ

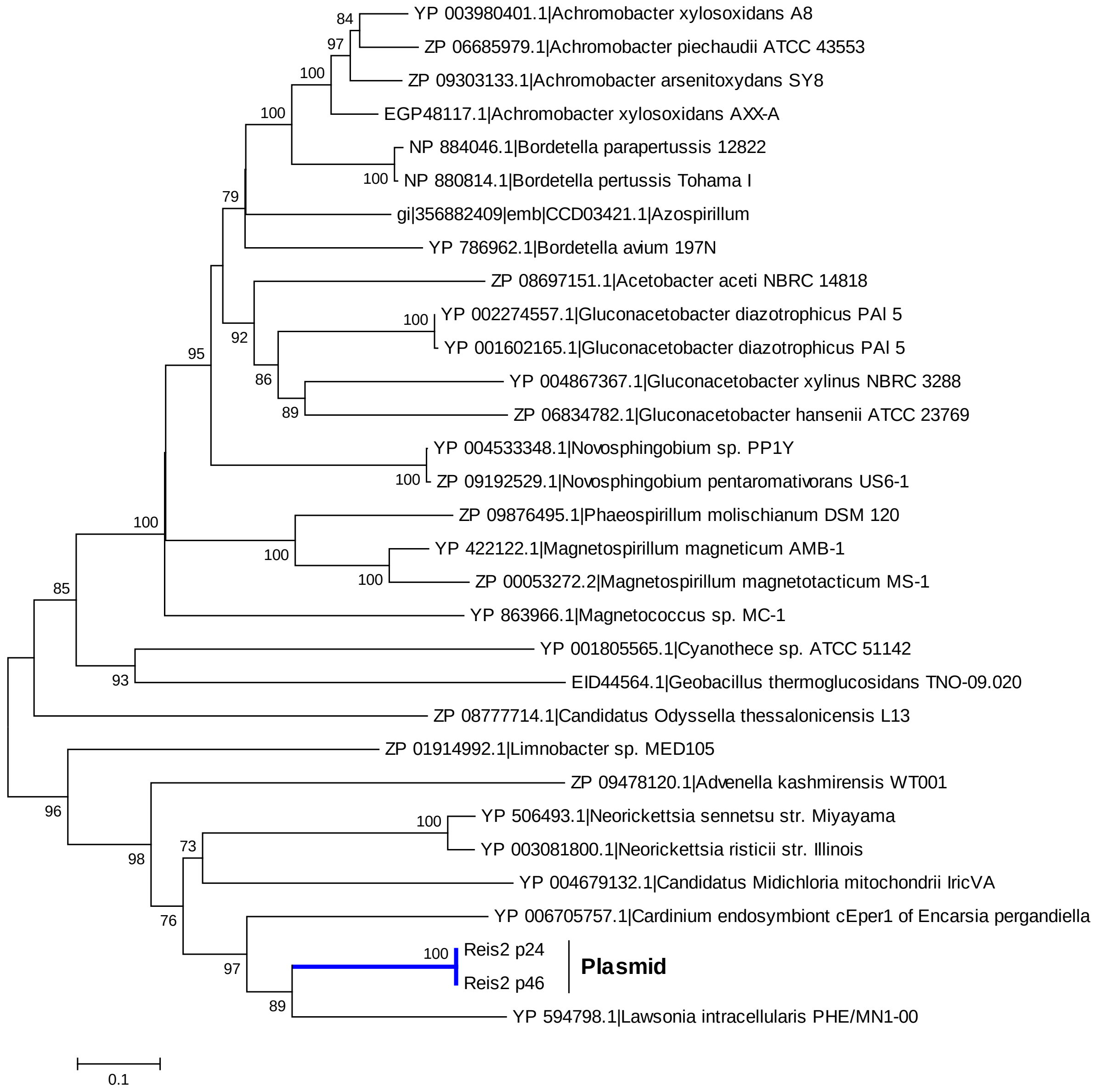

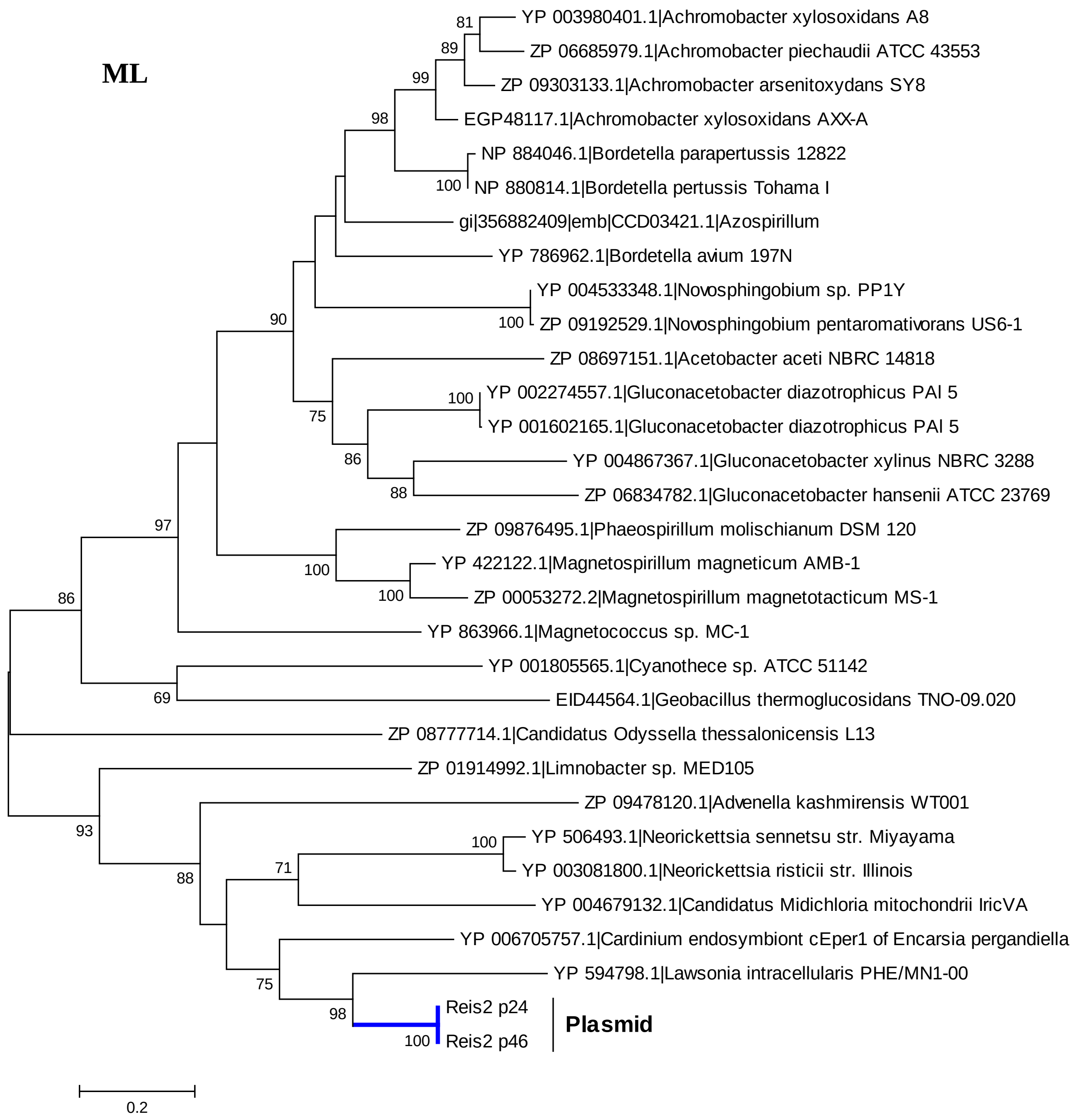
ML
YP 003980401.1|Achromobacter xylosoxidans A8
ZP 06685979.1|Achromobacter piechaudii ATCC 43553
ZP 09303133.1|Achromobacter arsenitoxydans SY8
EGP48117.1|Achromobacter xylosoxidans AXX-A
NP 884046.1|Bordetella parapertussis 12822
NP 880814.1|Bordetella pertussis Tohama I
gi|356882409|emb|CCD03421.1|Azospirillum
YP 786962.1|Bordetella avium 197N
YP 004533348.1|Novosphingobium sp. PP1Y
ZP 09192529.1|Novosphingobium pentaromativorans US6-1
ZP 08697151.1|Acetobacter aceti NBRC 14818
YP 002274557.1|Gluconacetobacter diazotrophicus PAl 5
YP 001602165.1|Gluconacetobacter diazotrophicus PAl 5
YP 004867367.1|Gluconacetobacter xylinus NBRC 3288
ZP 06834782.1|Gluconacetobacter hansenii ATCC 23769
ZP 09876495.1|Phaeospirillum molischianum DSM 120
YP 422122.1|Magnetospirillum magneticum AMB-1
ZP 00053272.2|Magnetospirillum magnetotacticum MS-1
YP 863966.1|Magnetococcus sp. MC-1
YP 001805565.1|Cyanothece sp. ATCC 51142
EID44564.1|Geobacillus thermoglucosidans TNO-09.020
ZP 08777714.1|Candidatus Odyssella thessalonicensis L13
ZP 01914992.1|Limnobacter sp. MED105
ZP 09478120.1|Advenella kashmirensis WT001
YP 506493.1|Neorickettsia sennetsu str. Miyayama
YP 003081800.1|Neorickettsia risticii str. Illinois
YP 004679132.1|Candidatus Midichloria mitochondrii IricVA
YP 006705757.1|Cardinium endosymbiont cEper1 of Encarsia pergandiella
YP 594798.1|Lawsonia intracellularis PHE/MN1-00
Reis2 p24
Reis2 p46
Plasmid
0.2

Figure A86. Neighbor-joining (NJ) and maximum likelihood (ML) trees of biotin synthase BioB gene. Bootstrap supports higher than or equal to 60% are shown on the branches.

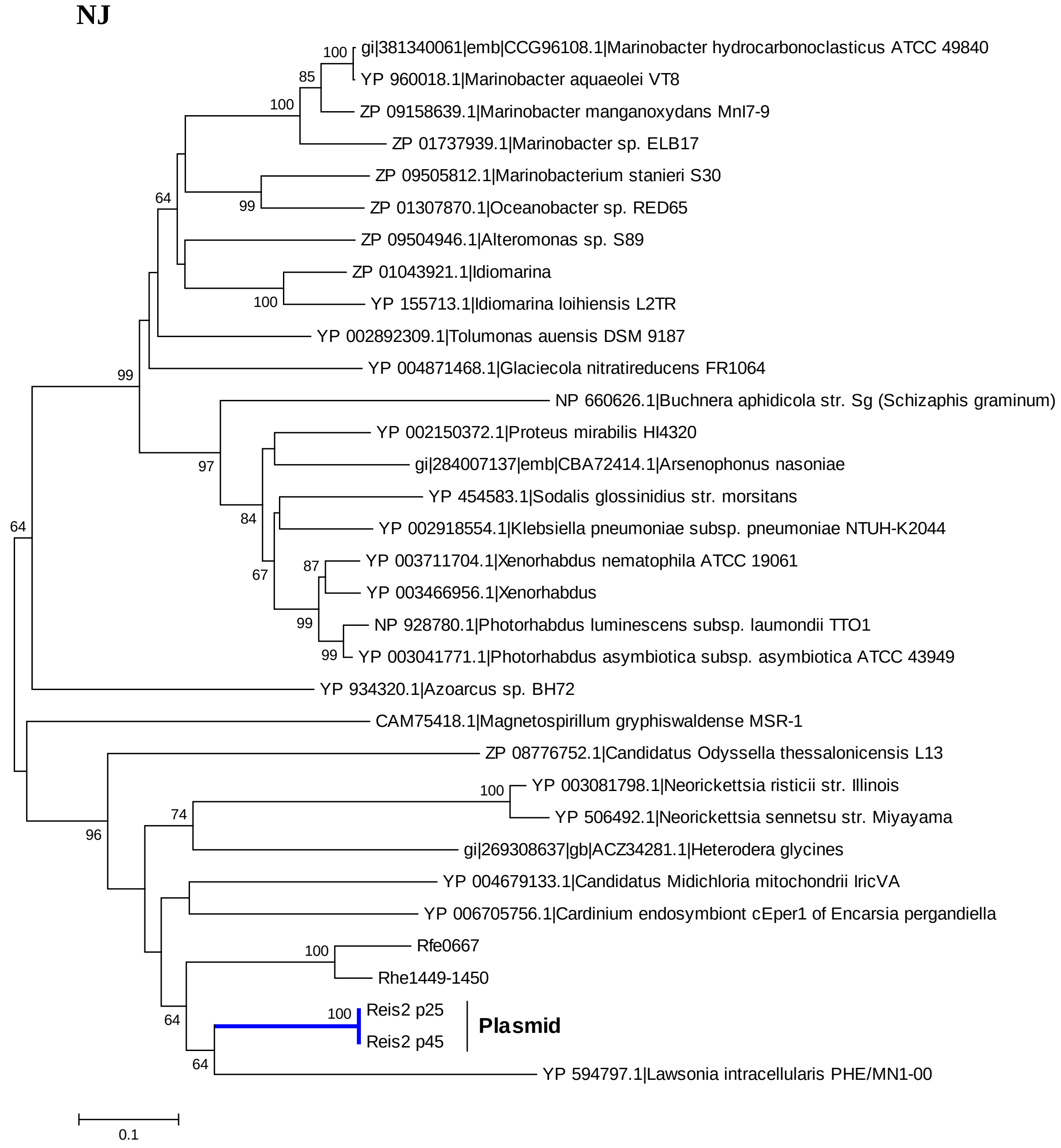

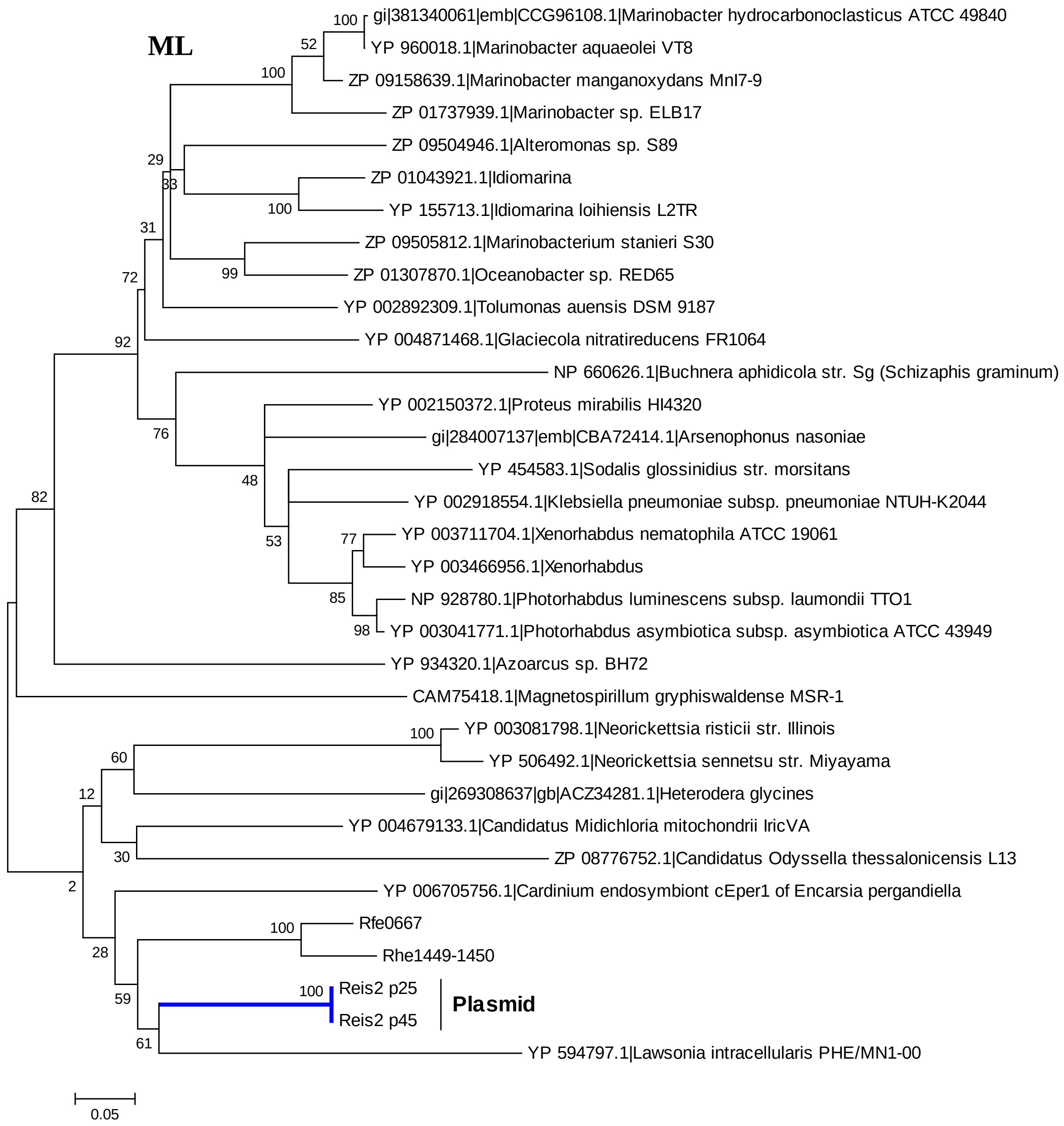
ML
gi|381340061|emb|CCG96108.1|Marinobacter hydrocarbonoclasticus ATCC 49840
YP 960018.1|Marinobacter aquaeolei VT8
ZP 09158639.1|Marinobacter manganoxydans MnI7-9
ZP 01737939.1|Marinobacter sp. ELB17
ZP 09504946.1|Alteromonas sp. S89
ZP 01043921.1|Idiomarina
YP 155713.1|Idiomarina loihiensis L2TR
ZP 09505812.1|Marinobacterium stanieri S30
ZP 01307870.1|Oceanobacter sp. RED65
YP 002892309.1|Tolumonas auensis DSM 9187
YP 004871468.1|Glaciecola nitratireducens FR1064
NP 660626.1|Buchnera aphidicola str. Sg (Schizaphis graminum)
YP 002150372.1|Proteus mirabilis HI4320
gi|284007137|emb|CBA72414.1|Arsenophonus nasoniae
YP 454583.1|Sodalis glossinidius str. morsitans
YP 002918554.1|Klebsiella pneumoniae subsp. pneumoniae NTUH-K2044
YP 003711704.1|Xenorhabdus nematophila ATCC 19061
YP 003466956.1|Xenorhabdus
NP 928780.1|Photorhabdus luminescens subsp. laumondii TTO1
YP 003041771.1|Photorhabdus asymbiotica subsp. asymbiotica ATCC 43949
YP 934320.1|Azoarcus sp. BH72
CAM75418.1|Magnetospirillum gryphiswaldense MSR-1
YP 003081798.1|Neorickettsia risticii str. Illinois
YP 506492.1|Neorickettsia sennetsu str. Miyayama
gi|269308637|gb|ACZ34281.1|Heterodera glycines
YP 004679133.1|Candidatus Midichloria mitochondrii IricVA
ZP 08776752.1|Candidatus Odyssella thessalonicensis L13
YP 006705756.1|Cardinium endosymbiont cEper1 of Encarsia pergandiella
Rfe0667
Rhe1449-1450
Reis2 p25
Reis2 p45
Plasmid
YP 594797.1|Lawsonia intracellularis PHE/MN1-00
100
52
100
29
33
100
31
99
72
92
76
48
82
53
77
85
98
100
60
12
30
2
100
28
100
59
61
0.05

Figure A87. Neighbor-joining (NJ) and maximum likelihood (ML) trees of acetyltransferase. Bootstrap supports higher than or equal to 60% are shown on the branches.

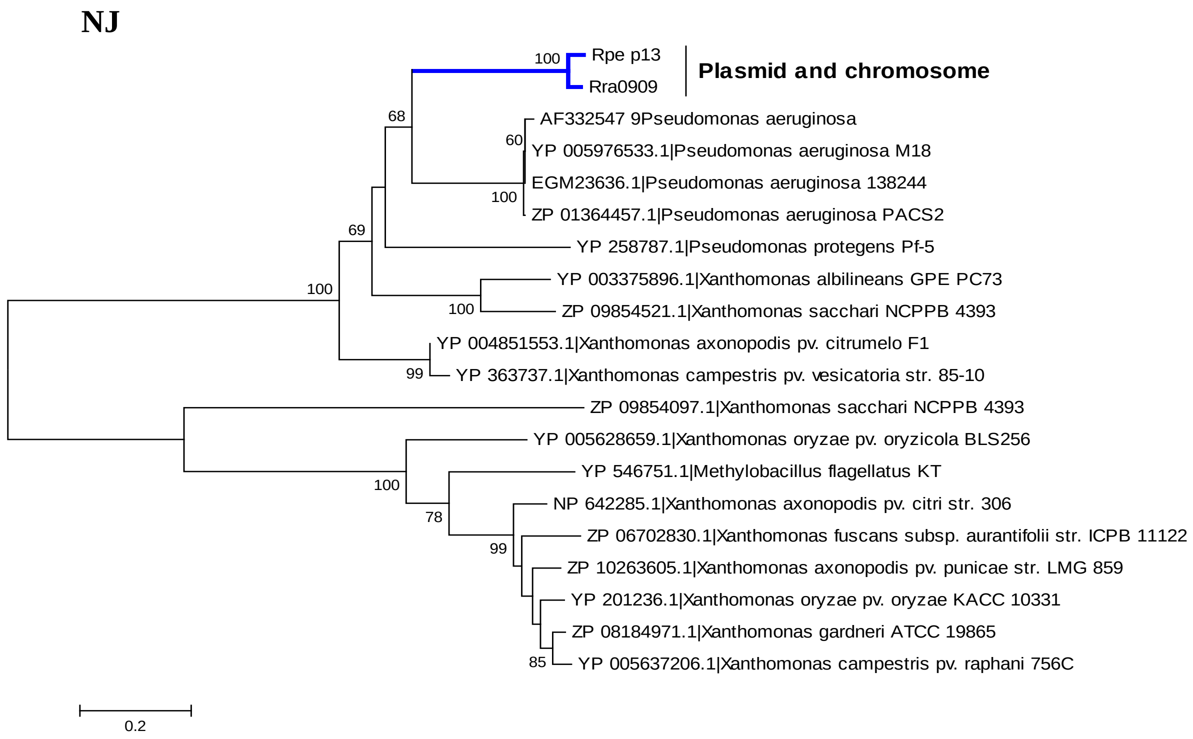

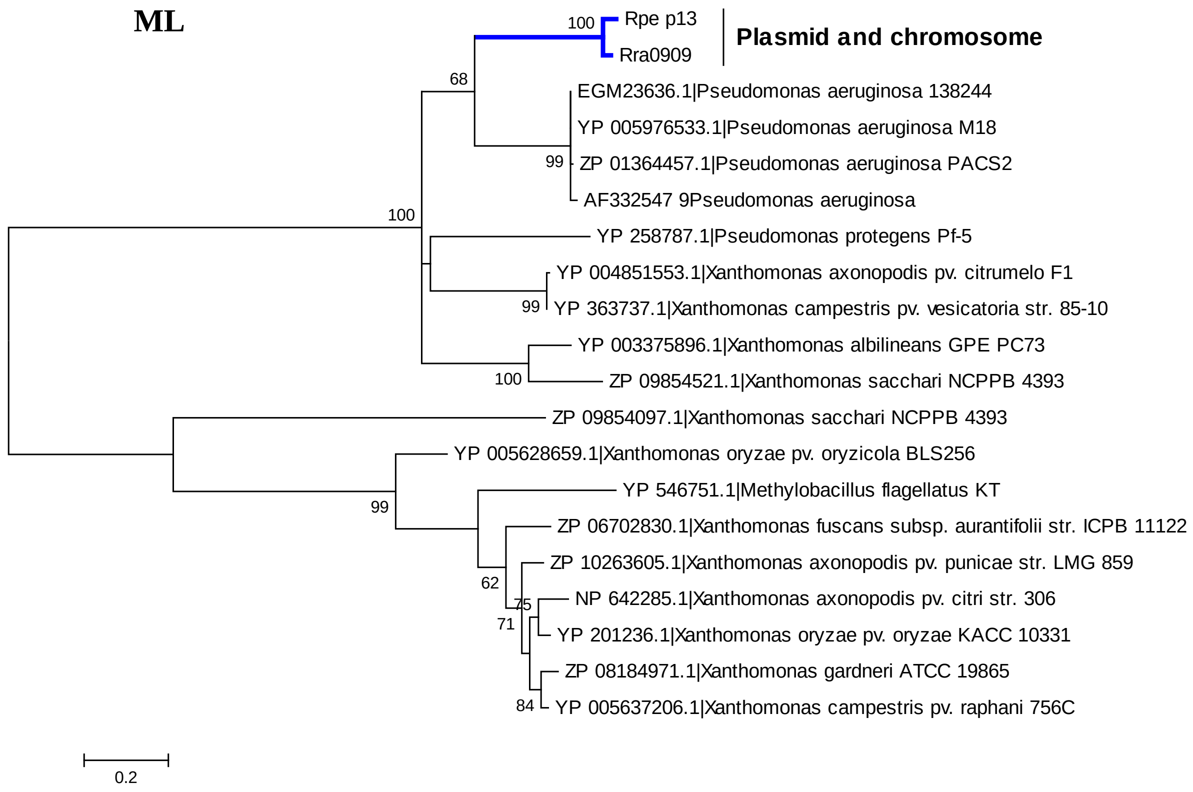

Figure A88. Neighbor-joining (NJ) and maximum likelihood (ML) trees of rieske non-heme iron oxygenase (RO) family protein. Bootstrap supports higher than or equal to 60% are shown on the branches.

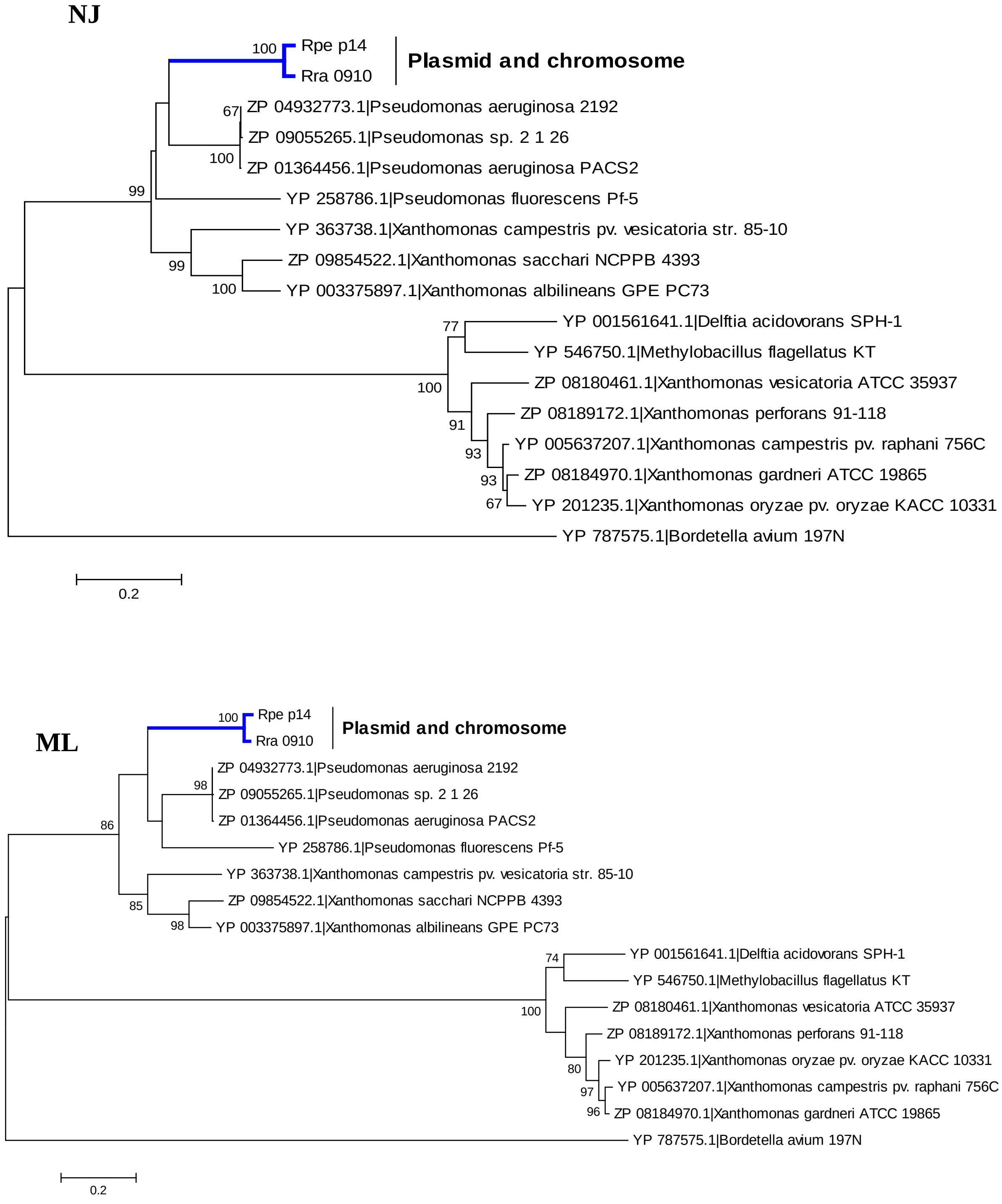

Figure A89. Neighbor-joining (NJ) and maximum likelihood (ML) trees of short chain dehydrogenase/reductase family protein. Bootstrap supports higher than or equal to 60% are shown on the branches.

NJ

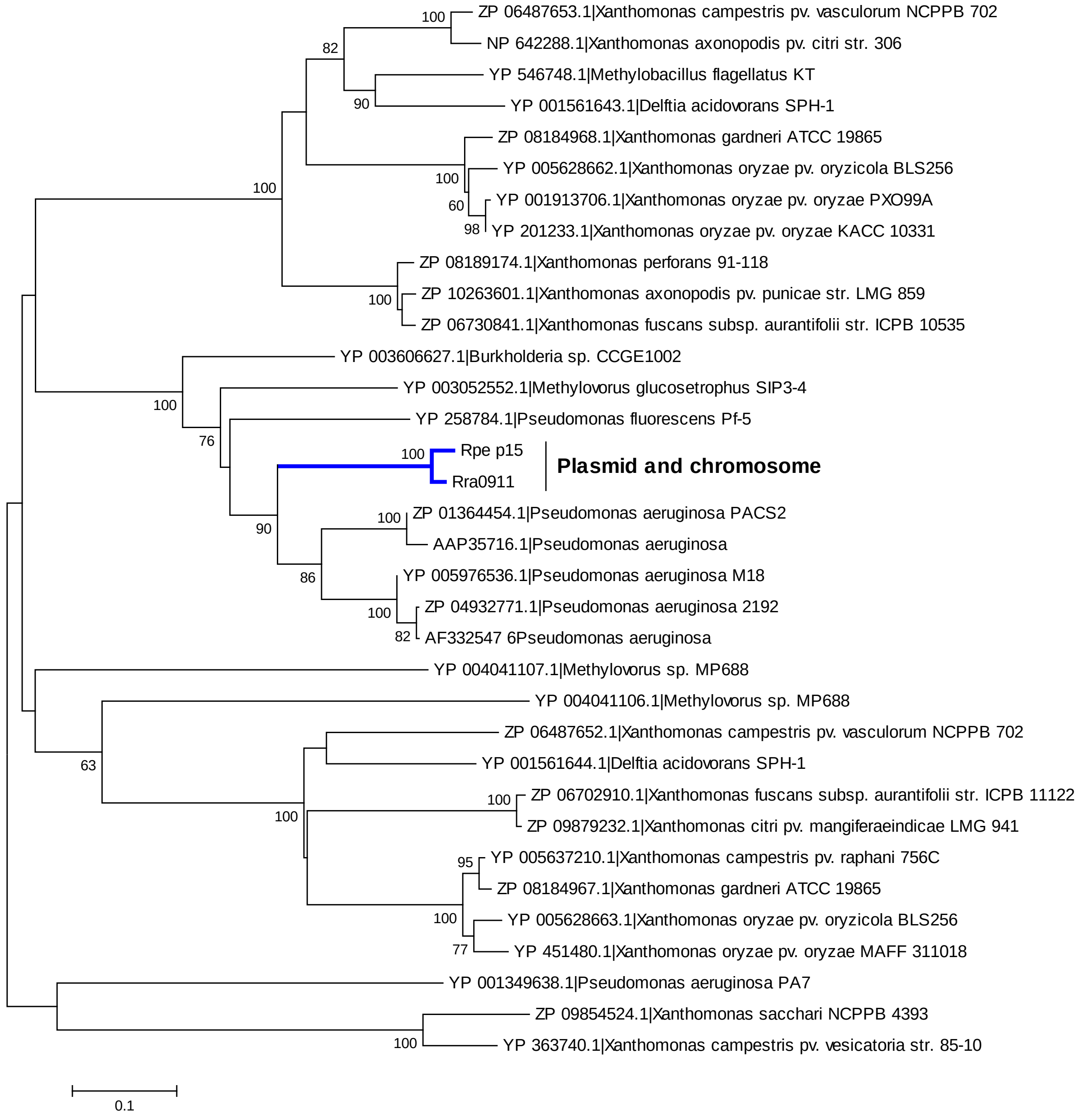

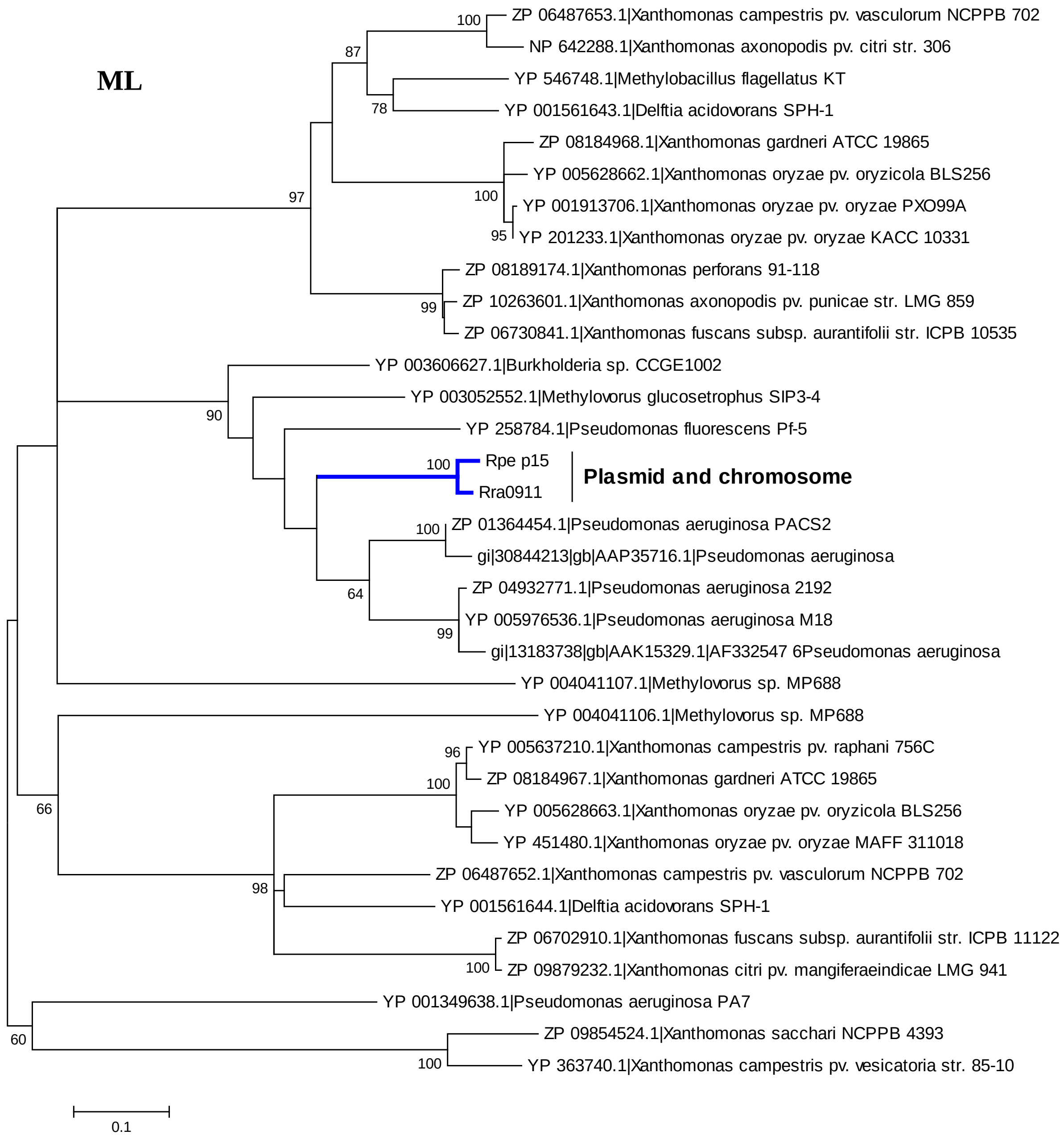
ML
ZP 06487653.1|Xanthomonas campestris pv. vasculorum NCPPB 702
NP 642288.1|Xanthomonas axonopodis pv. citri str. 306
YP 546748.1|Methylobacillus flagellatus KT
YP 001561643.1|Delftia acidovorans SPH-1
ZP 08184968.1|Xanthomonas gardneri ATCC 19865
YP 005628662.1|Xanthomonas oryzae pv. oryzicola BLS256
YP 001913706.1|Xanthomonas oryzae pv. oryzae PXO99A
YP 201233.1|Xanthomonas oryzae pv. oryzae KACC 10331
ZP 08189174.1|Xanthomonas perforans 91-118
ZP 10263601.1|Xanthomonas axonopodis pv. punicae str. LMG 859
ZP 06730841.1|Xanthomonas fuscans subsp. aurantifolii str. ICPB 10535
YP 003606627.1|Burkholderia sp. CCGE1002
YP 003052552.1|Methylovorus glucosetrophus SIP3-4
YP 258784.1|Pseudomonas fluorescens Pf-5
Rpe p15
Rra0911
Plasmid and chromosome
ZP 01364454.1|Pseudomonas aeruginosa PACS2
gi|30844213|gb|AAP35716.1|Pseudomonas aeruginosa
ZP 04932771.1|Pseudomonas aeruginosa 2192
YP 005976536.1|Pseudomonas aeruginosa M18
gi|13183738|gb|AAK15329.1|AF332547 6Pseudomonas aeruginosa
YP 004041107.1|Methylovorus sp. MP688
YP 004041106.1|Methylovorus sp. MP688
YP 005637210.1|Xanthomonas campestris pv. raphani 756C
ZP 08184967.1|Xanthomonas gardneri ATCC 19865
YP 005628663.1|Xanthomonas oryzae pv. oryzicola BLS256
YP 451480.1|Xanthomonas oryzae pv. oryzae MAFF 311018
ZP 06487652.1|Xanthomonas campestris pv. vasculorum NCPPB 702
YP 001561644.1|Delftia acidovorans SPH-1
ZP 06702910.1|Xanthomonas fuscans subsp. aurantifolii str. ICPB 11122
ZP 09879232.1|Xanthomonas citri pv. mangiferaeindicae LMG 941
YP 001349638.1|Pseudomonas aeruginosa PA7
ZP 09854524.1|Xanthomonas sacchari NCPPB 4393
YP 363740.1|Xanthomonas campestris pv. vesicatoria str. 85-10
100
87
78
97
100
95
99
90
100
100
64
99
96
100
66
98
100
60
100
0.1

Figure A90. Neighbor-joining (NJ) and maximum likelihood (ML) trees of 3-oxoacyl-(acyl-carrier-protein) synthase III. Bootstrap supports higher than or equal to 60% are shown on the branches.

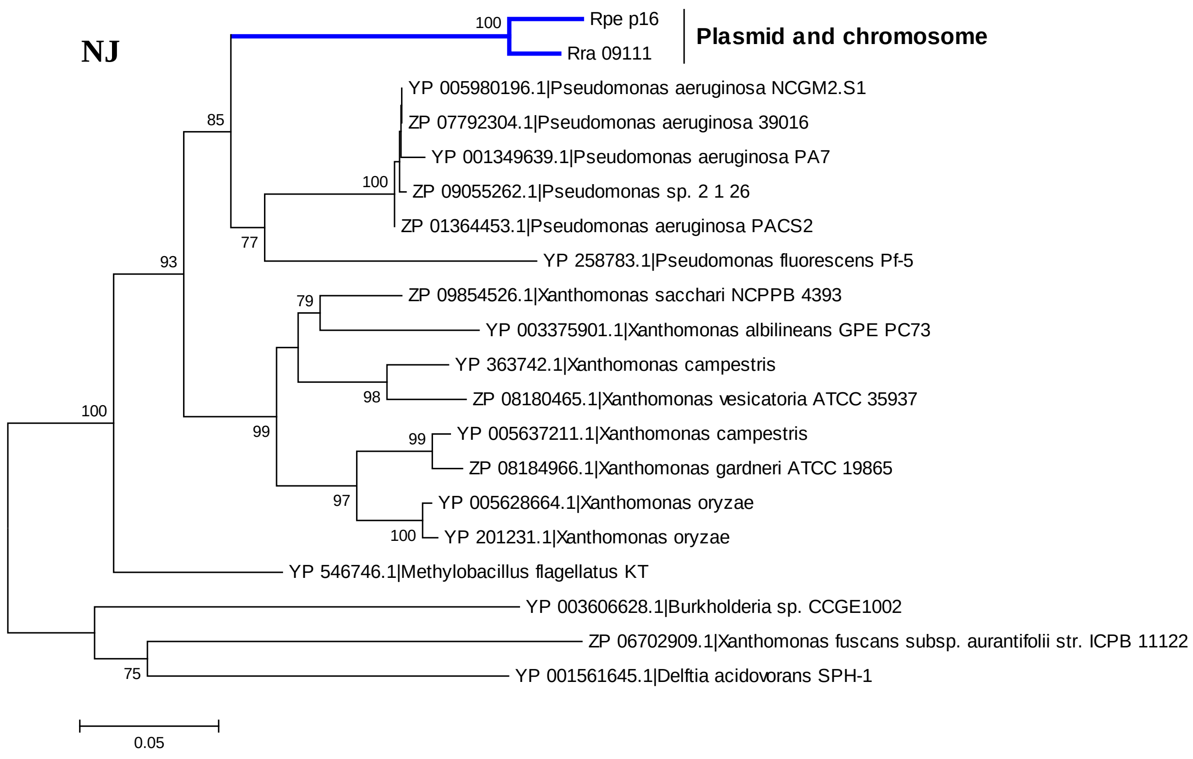

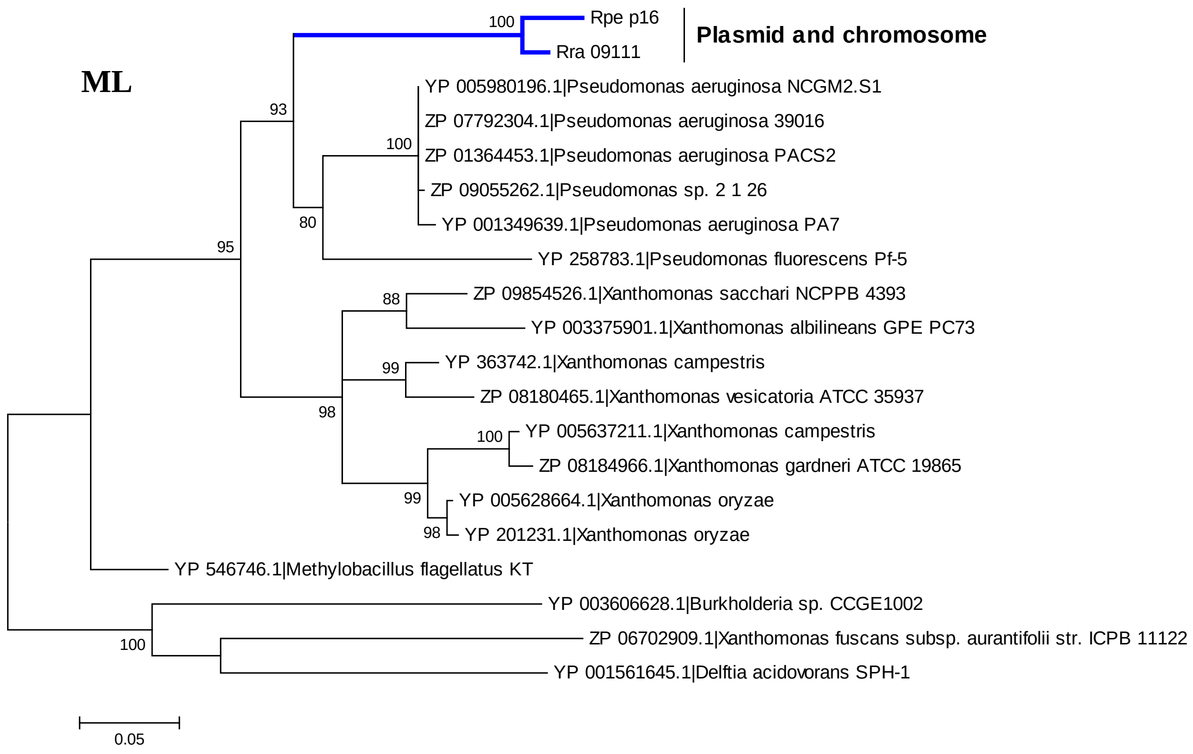

Figure A91. Neighbor-joining (NJ) and maximum likelihood (ML) trees of acyl carrier protein. Bootstrap supports higher than or equal to 60% are shown on the branches.

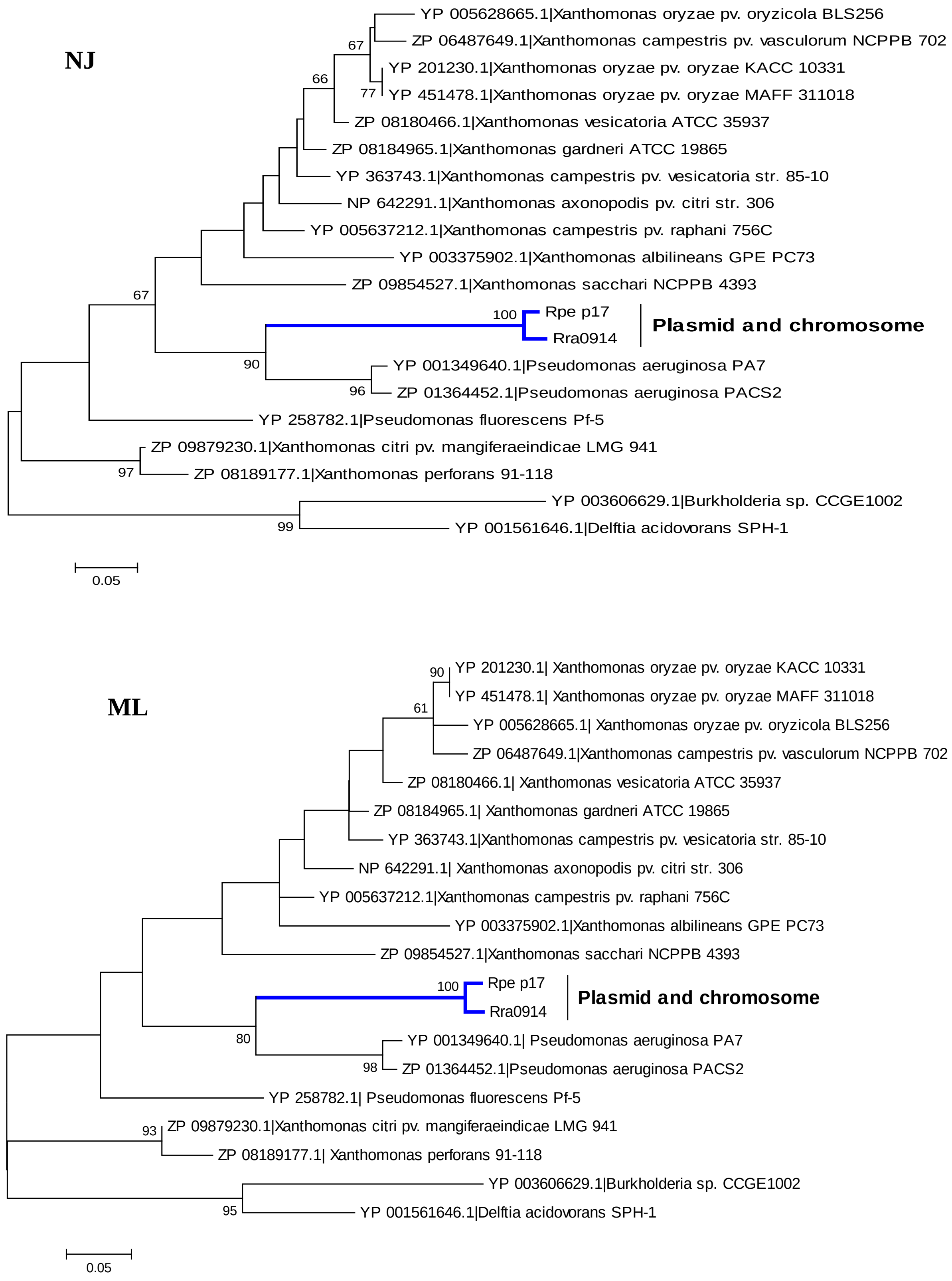

Figure A92. Neighbor-joining (NJ) and maximum likelihood (ML) trees of ABC multidrug transporter, permease/ATP-binding protein. Bootstrap supports higher than or equal to 60% are shown on the branches.

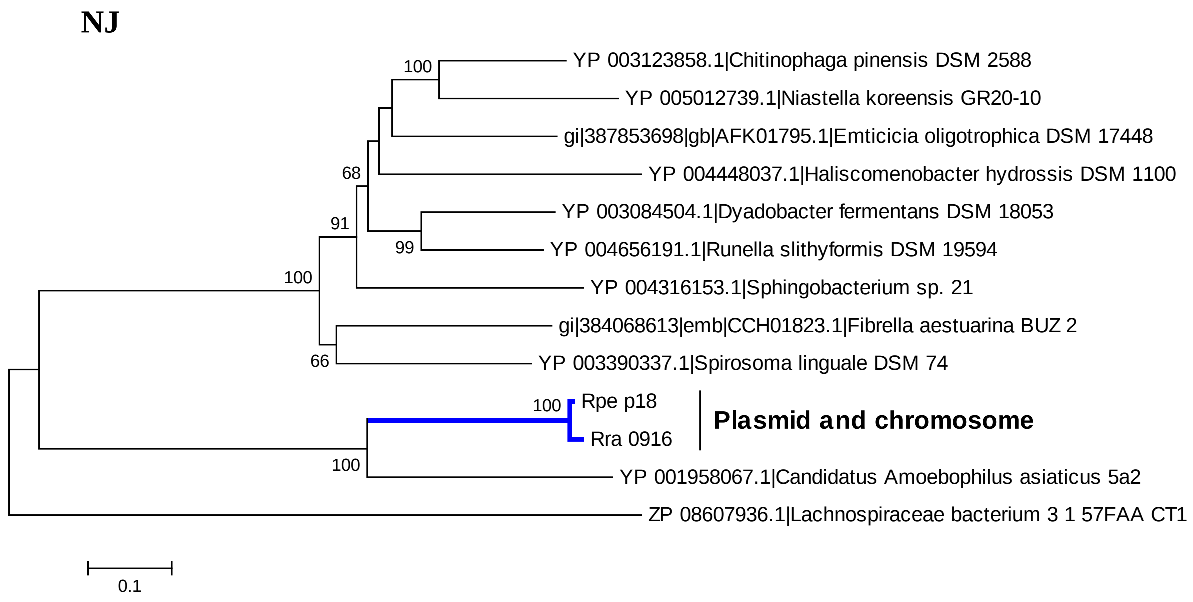

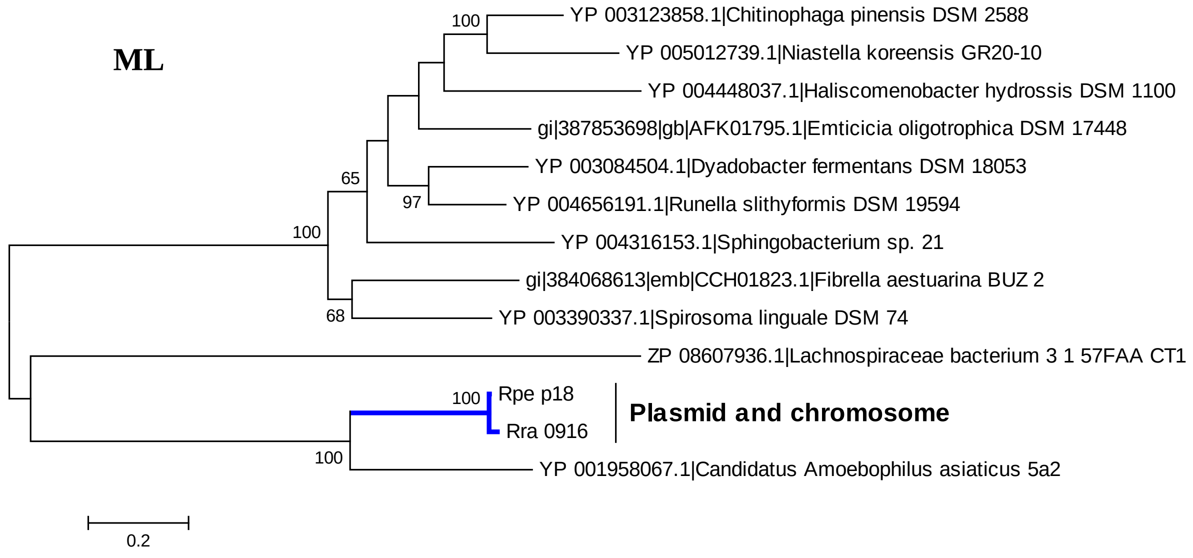

Figure A93. Neighbor-joining (NJ) and maximum likelihood (ML) trees of plasmid partitioning protein ParA. Bootstrap supports higher than or equal to 60% are shown on the branches.

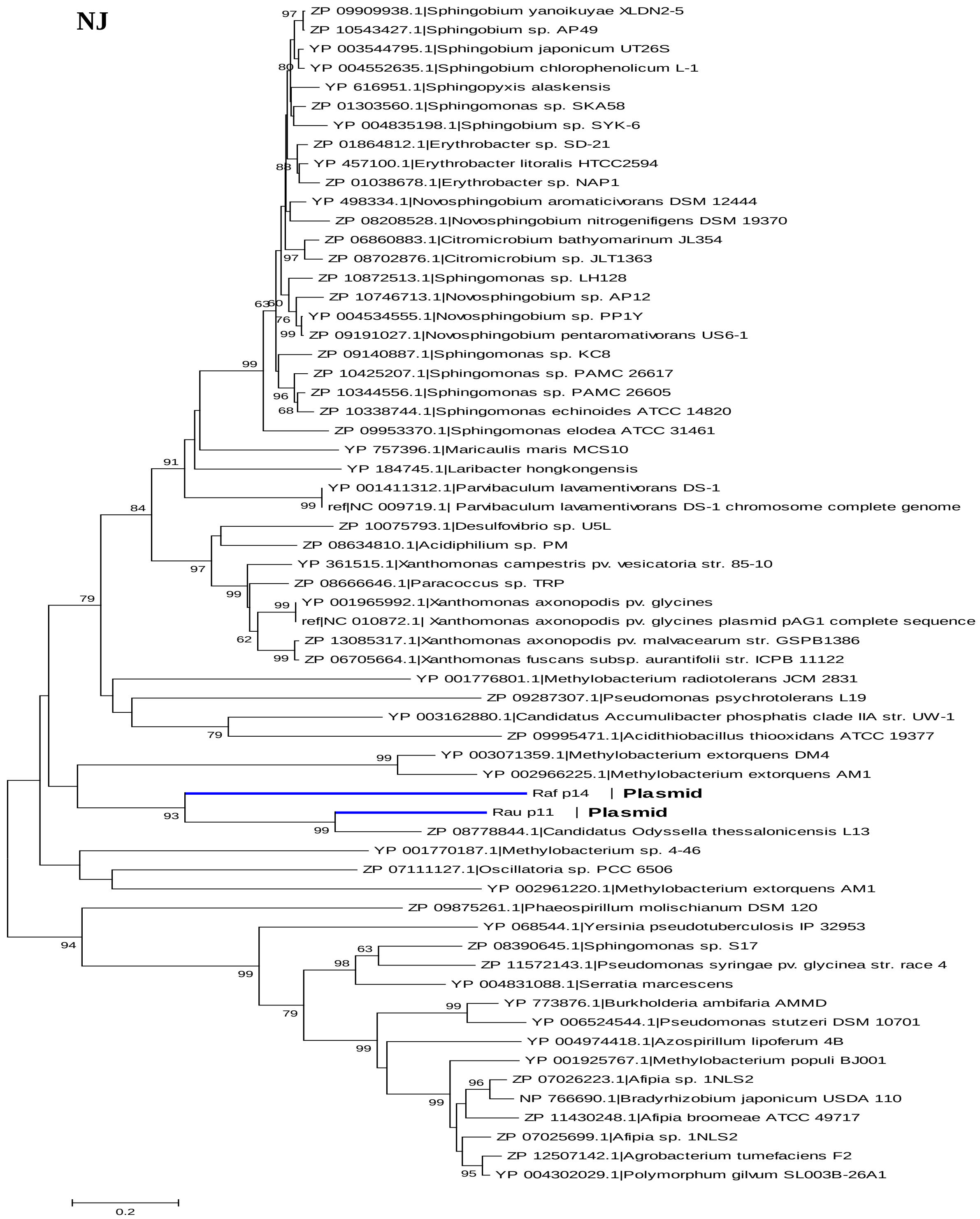

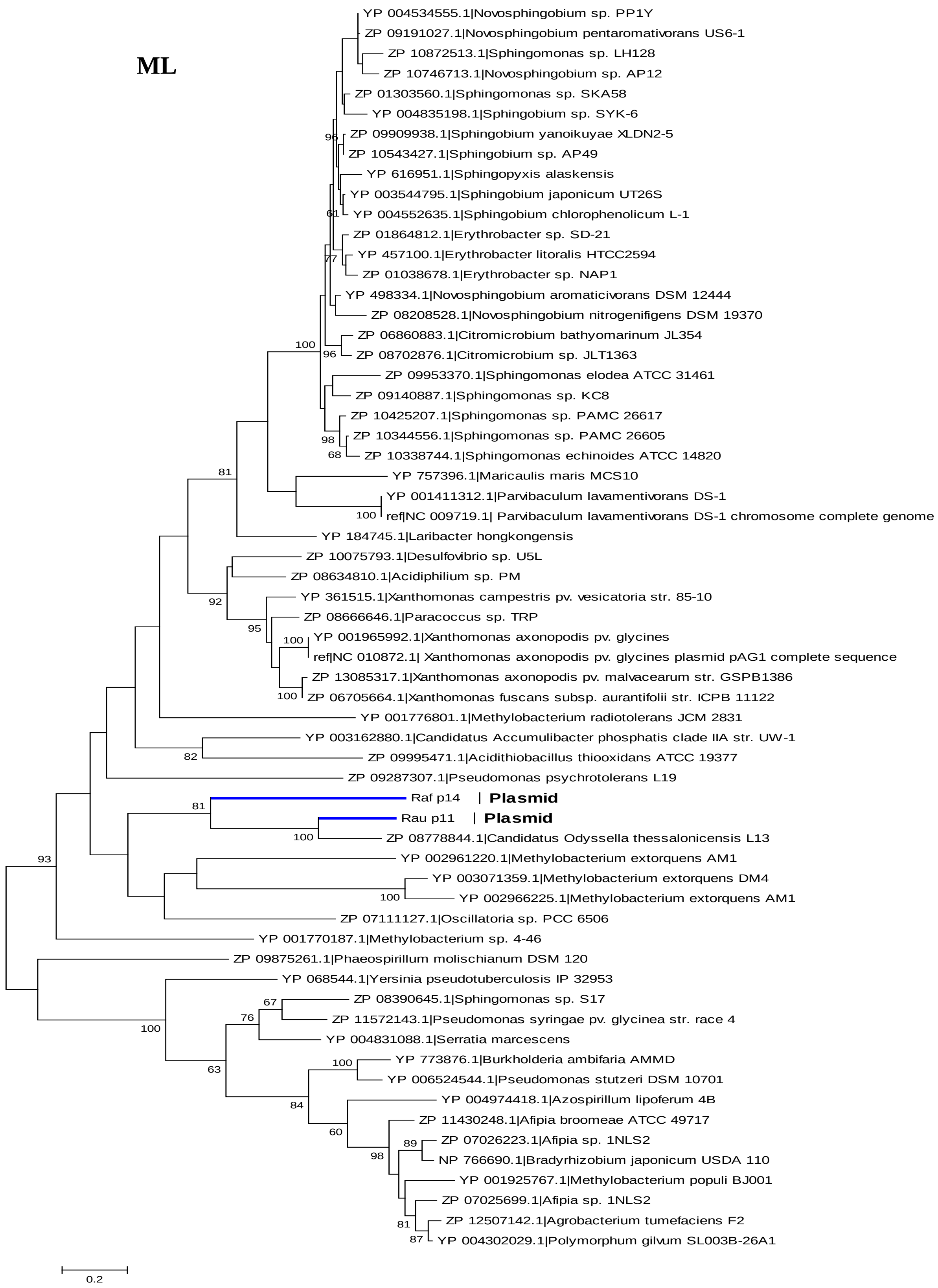
ML
YP 004534555.1|Novosphingobium sp. PP1Y
ZP 09191027.1|Novosphingobium pentaromativorans US6-1
ZP 10872513.1|Sphingomonas sp. LH128
ZP 10746713.1|Novosphingobium sp. AP12
ZP 01303560.1|Sphingomonas sp. SKA58
YP 004835198.1|Sphingobium sp. SYK-6
ZP 09909938.1|Sphingobium yanoikuyae XLDN2-5
ZP 10543427.1|Sphingobium sp. AP49
YP 616951.1|Sphingopyxis alaskensis
YP 003544795.1|Sphingobium japonicum UT26S
YP 004552635.1|Sphingobium chlorophenolicum L-1
ZP 01864812.1|Erythrobacter sp. SD-21
YP 457100.1|Erythrobacter litoralis HTCC2594
ZP 01038678.1|Erythrobacter sp. NAP1
YP 498334.1|Novosphingobium aromaticivorans DSM 12444
ZP 08208528.1|Novosphingobium nitrogenifigens DSM 19370
ZP 06860883.1|Citromicrobium bathyomarinum JL354
ZP 08702876.1|Citromicrobium sp. JLT1363
ZP 09953370.1|Sphingomonas elodea ATCC 31461
ZP 09140887.1|Sphingomonas sp. KC8
ZP 10425207.1|Sphingomonas sp. PAMC 26617
ZP 10344556.1|Sphingomonas sp. PAMC 26605
ZP 10338744.1|Sphingomonas echinoides ATCC 14820
YP 757396.1|Maricaulis maris MCS10
YP 001411312.1|Parvibaculum lavamentivorans DS-1
ref|NC 009719.1| Parvibaculum lavamentivorans DS-1 chromosome complete genome
YP 184745.1|Laribacter hongkongensis
ZP 10075793.1|Desulfovibrio sp. U5L
ZP 08634810.1|Acidiphilium sp. PM
YP 361515.1|Xanthomonas campestris pv. vesicatoria str. 85-10
ZP 08666646.1|Paracoccus sp. TRP
YP 001965992.1|Xanthomonas axonopodis pv. glycines
ref|NC 010872.1| Xanthomonas axonopodis pv. glycines plasmid pAG1 complete sequence
ZP 13085317.1|Xanthomonas axonopodis pv. malvacearum str. GSPB1386
ZP 06705664.1|Xanthomonas fuscans subsp. aurantifolii str. ICPB 11122
YP 001776801.1|Methylobacterium radiotolerans JCM 2831
YP 003162880.1|Candidatus Accumulibacter phosphatis clade IIA str. UW-1
ZP 09995471.1|Acidithiobacillus thiooxidans ATCC 19377
ZP 09287307.1|Pseudomonas psychrotolerans L19
Raf p14 | Plasmid
Rau p11 | Plasmid
ZP 08778844.1|Candidatus Odyssella thessalonicensis L13
YP 002961220.1|Methylobacterium extorquens AM1
YP 003071359.1|Methylobacterium extorquens DM4
YP 002966225.1|Methylobacterium extorquens AM1
ZP 07111127.1|Oscillatoria sp. PCC 6506
YP 001770187.1|Methylobacterium sp. 4-46
ZP 09875261.1|Phaeospirillum molischianum DSM 120
YP 068544.1|Yersinia pseudotuberculosis IP 32953
ZP 08390645.1|Sphingomonas sp. S17
ZP 11572143.1|Pseudomonas syringae pv. glycinea str. race 4
YP 004831088.1|Serratia marcescens
YP 773876.1|Burkholderia ambifaria AMMD
YP 006524544.1|Pseudomonas stutzeri DSM 10701
YP 004974418.1|Azospirillum lipoferum 4B
ZP 11430248.1|Afipia broomeae ATCC 49717
ZP 07026223.1|Afipia sp. 1NLS2
NP 766690.1|Bradyrhizobium japonicum USDA 110
YP 001925767.1|Methylobacterium populi BJ001
ZP 07025699.1|Afipia sp. 1NLS2
ZP 12507142.1|Agrobacterium tumefaciens F2
YP 004302029.1|Polymorphum gilvum SL003B-26A1
0.2

Figure A94. Neighbor-joining (NJ) and maximum likelihood (ML) trees of plasmid partitioning protein ParA. Bootstrap supports higher than or equal to 60% are shown on the branches.

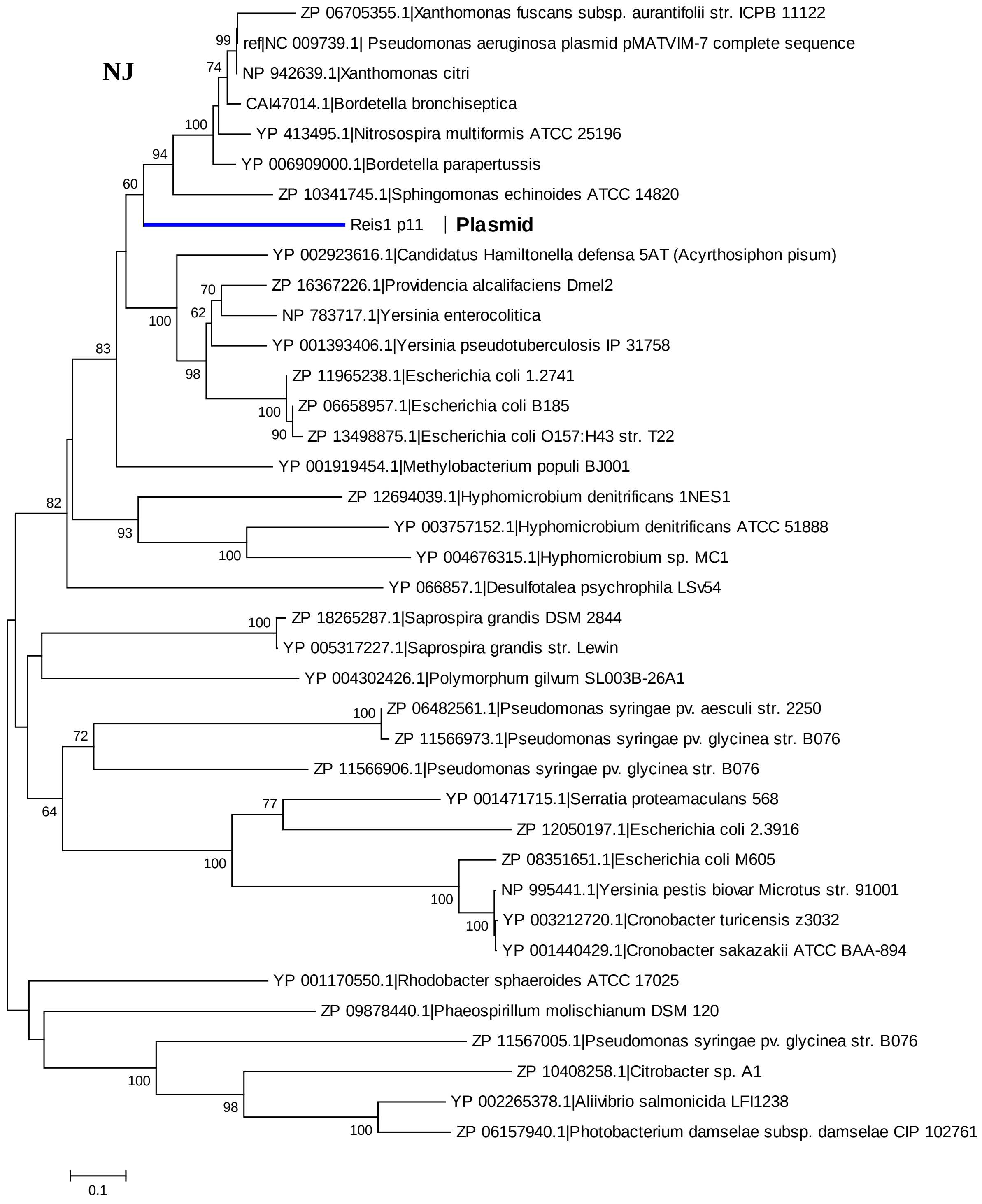

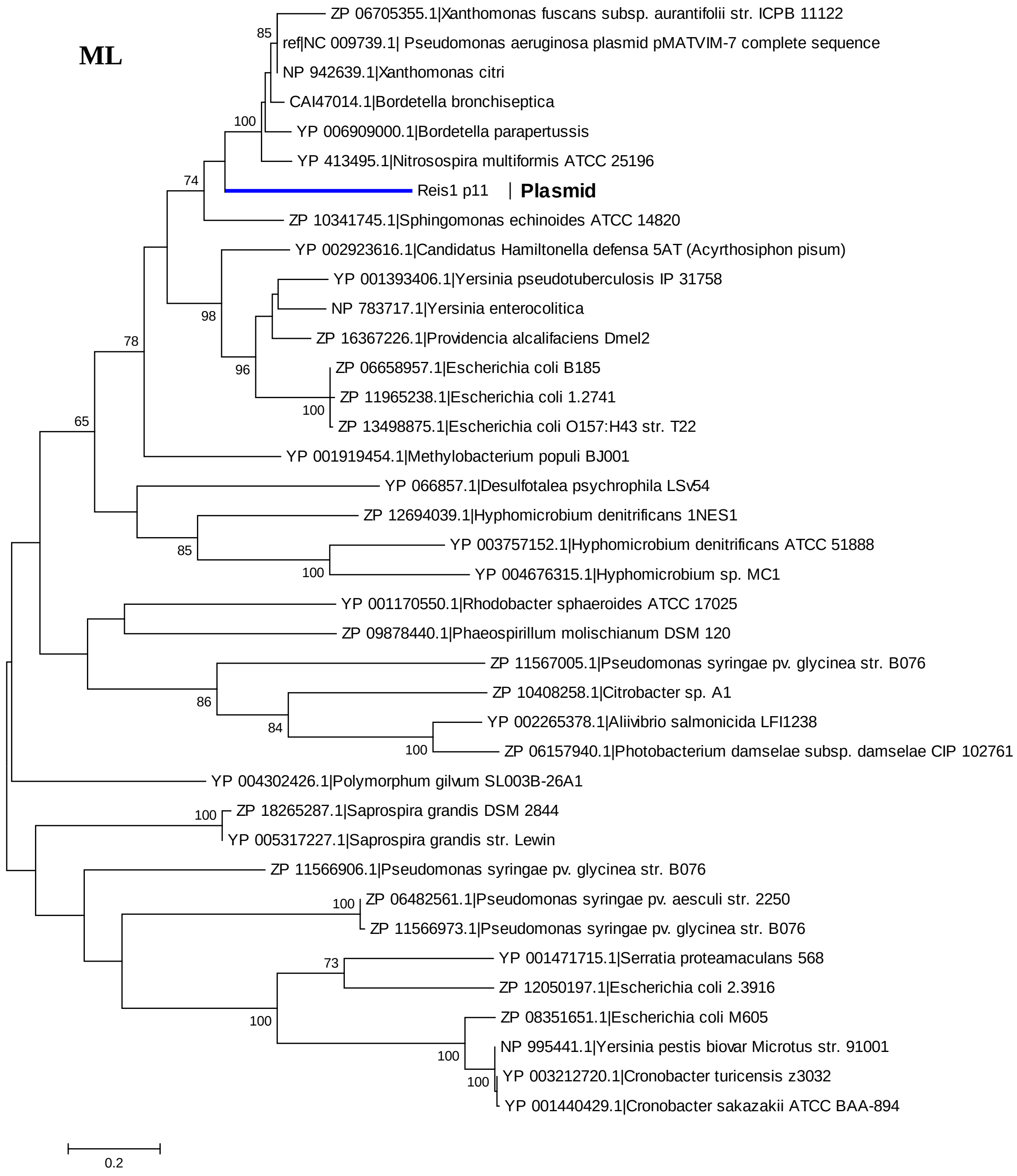
ML
ZP 06705355.1|Xanthomonas fuscans subsp. aurantifolii str. ICPB 11122
ref|NC 009739.1| Pseudomonas aeruginosa plasmid pMATVIM-7 complete sequence
NP 942639.1|Xanthomonas citri
CAI47014.1|Bordetella bronchiseptica
YP 006909000.1|Bordetella parapertussis
YP 413495.1|Nitrosospira multiformis ATCC 25196
Reis1 p11
Plasmid
ZP 10341745.1|Sphingomonas echinoides ATCC 14820
YP 002923616.1|Candidatus Hamiltonella defensa 5AT (Acyrthosiphon pisum)
YP 001393406.1|Yersinia pseudotuberculosis IP 31758
NP 783717.1|Yersinia enterocolitica
ZP 16367226.1|Providencia alcalifaciens Dmel2
ZP 06658957.1|Escherichia coli B185
ZP 11965238.1|Escherichia coli 1.2741
ZP 13498875.1|Escherichia coli O157:H43 str. T22
YP 001919454.1|Methylobacterium populi BJ001
YP 066857.1|Desulfotalea psychrophila LSv54
ZP 12694039.1|Hyphomicrobium denitrificans 1NES1
YP 003757152.1|Hyphomicrobium denitrificans ATCC 51888
YP 004676315.1|Hyphomicrobium sp. MC1
YP 001170550.1|Rhodobacter sphaeroides ATCC 17025
ZP 09878440.1|Phaeospirillum molischianum DSM 120
ZP 11567005.1|Pseudomonas syringae pv. glycinea str. B076
ZP 10408258.1|Citrobacter sp. A1
YP 002265378.1|Aliivibrio salmonicida LFI1238
ZP 06157940.1|Photobacterium damselae subsp. damselae CIP 102761
YP 004302426.1|Polymorphum gilvum SL003B-26A1
ZP 18265287.1|Saprospira grandis DSM 2844
YP 005317227.1|Saprospira grandis str. Lewin
ZP 11566906.1|Pseudomonas syringae pv. glycinea str. B076
ZP 06482561.1|Pseudomonas syringae pv. aesculi str. 2250
ZP 11566973.1|Pseudomonas syringae pv. glycinea str. B076
YP 001471715.1|Serratia proteamaculans 568
ZP 12050197.1|Escherichia coli 2.3916
ZP 08351651.1|Escherichia coli M605
NP 995441.1|Yersinia pestis biovar Microtus str. 91001
YP 003212720.1|Cronobacter turicensis z3032
YP 001440429.1|Cronobacter sakazakii ATCC BAA-894
85
100
74
98
96
100
78
65
85
100
86
84
100
100
100
73
100
100
100
0.2

Figure A95. Neighbor-joining (NJ) and maximum likelihood (ML) trees of plasmid partitioning protein ParA. Bootstrap supports higher than or equal to 60% are shown on the branches.

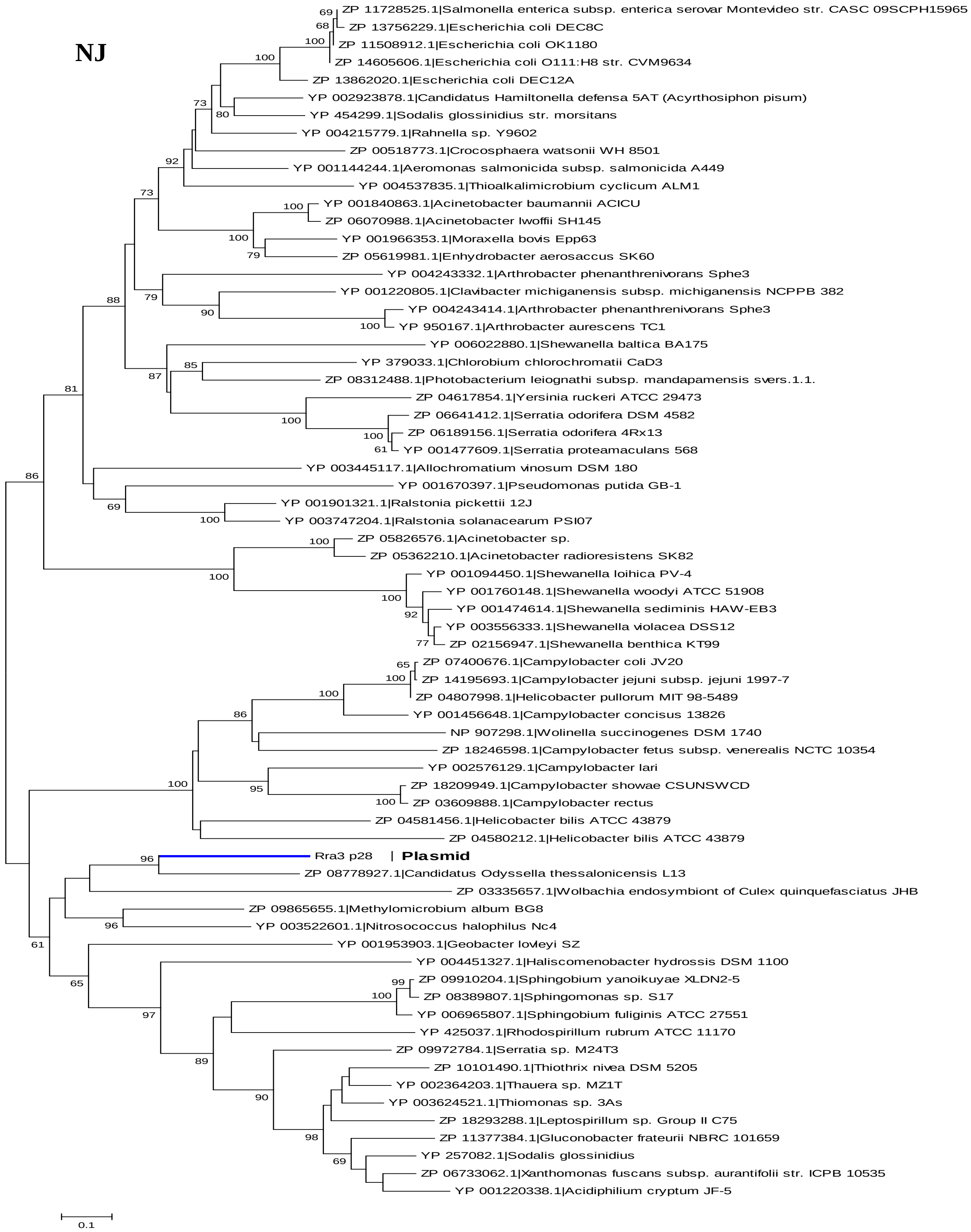

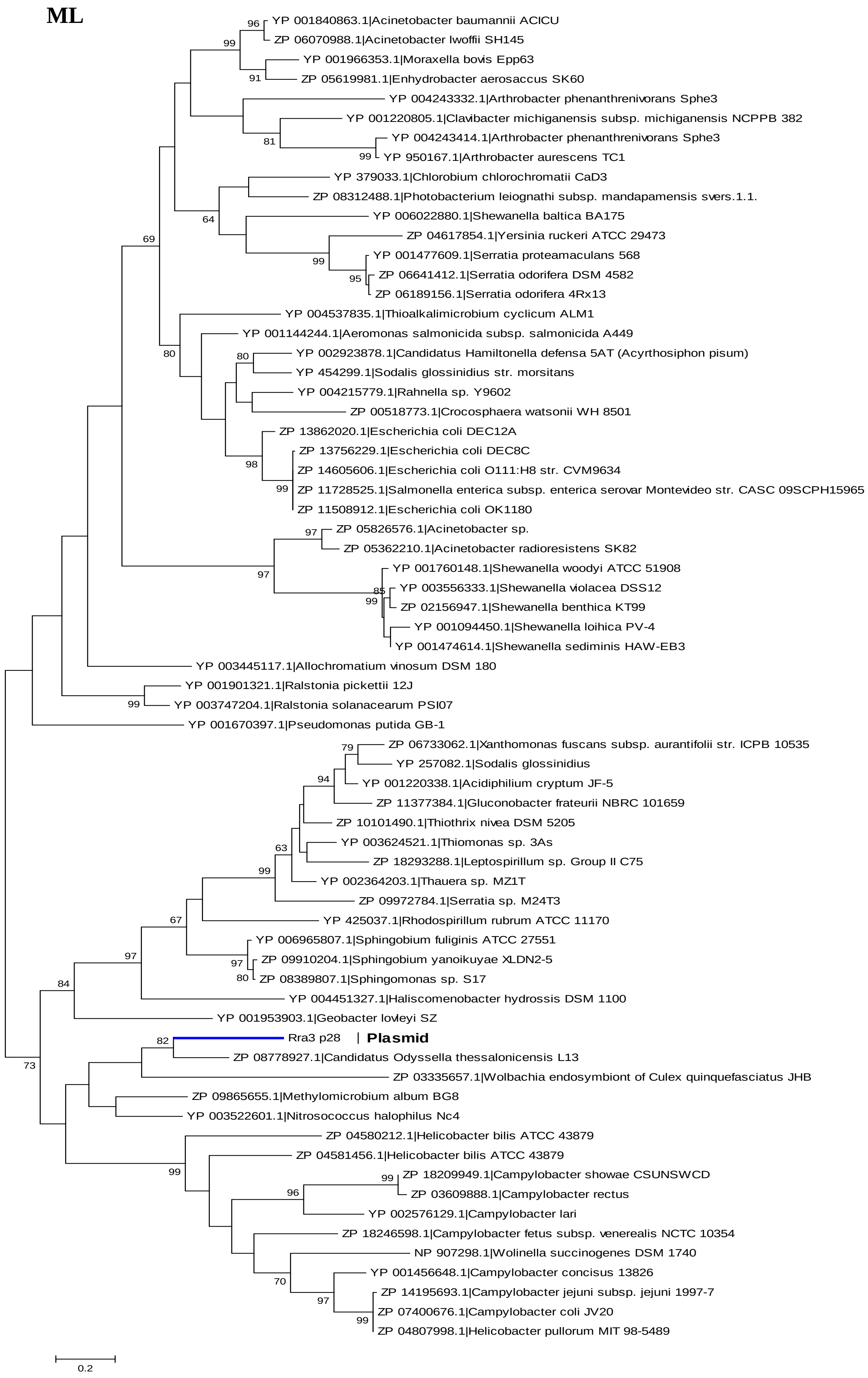
ML
YP 001840863.1|Acinetobacter baumannii ACICU
ZP 06070988.1|Acinetobacter lwoffii SH145
YP 001966353.1|Moraxella bovis Epp63
ZP 05619981.1|Enhydrobacter aerosaccus SK60
YP 004243332.1|Arthrobacter phenanthrenivorans Sphe3
YP 001220805.1|Clavibacter michiganensis subsp. michiganensis NCPPB 382
YP 004243414.1|Arthrobacter phenanthrenivorans Sphe3
YP 950167.1|Arthrobacter aurescens TC1
YP 379033.1|Chlorobium chlorochromatii CaD3
ZP 08312488.1|Photobacterium leiognathi subsp. mandapamensis svers.1.1.
YP 006022880.1|Shewanella baltica BA175
ZP 04617854.1|Yersinia ruckeri ATCC 29473
YP 001477609.1|Serratia proteamaculans 568
ZP 06641412.1|Serratia odorifera DSM 4582
ZP 06189156.1|Serratia odorifera 4Rx13
YP 004537835.1|Thioalkalimicrobium cyclicum ALM1
YP 001144244.1|Aeromonas salmonicida subsp. salmonicida A449
YP 002923878.1|Candidatus Hamiltonella defensa 5AT (Acyrthosiphon pisum)
YP 454299.1|Sodalis glossinidius str. morsitans
YP 004215779.1|Rahnella sp. Y9602
ZP 00518773.1|Crocosphaera watsonii WH 8501
ZP 13862020.1|Escherichia coli DEC12A
ZP 13756229.1|Escherichia coli DEC8C
ZP 14605606.1|Escherichia coli O111:H8 str. CVM9634
ZP 11728525.1|Salmonella enterica subsp. enterica serovar Montevideo str. CASC 09SCPH15965
ZP 11508912.1|Escherichia coli OK1180
ZP 05826576.1|Acinetobacter sp.
ZP 05362210.1|Acinetobacter radioresistens SK82
YP 001760148.1|Shewanella woodyi ATCC 51908
YP 003556333.1|Shewanella violacea DSS12
ZP 02156947.1|Shewanella benthica KT99
YP 001094450.1|Shewanella loihica PV-4
YP 001474614.1|Shewanella sediminis HAW-EB3
YP 003445117.1|Allochromatium vinosum DSM 180
YP 001901321.1|Ralstonia pickettii 12J
YP 003747204.1|Ralstonia solanacearum PSI07
YP 001670397.1|Pseudomonas putida GB-1
ZP 06733062.1|Xanthomonas fuscans subsp. aurantifolii str. ICPB 10535
YP 257082.1|Sodalis glossinidius
YP 001220338.1|Acidiphilium cryptum JF-5
ZP 11377384.1|Gluconobacter frateurii NBRC 101659
ZP 10101490.1|Thiothrix nivea DSM 5205
YP 003624521.1|Thiomonas sp. 3As
ZP 18293288.1|Leptospirillum sp. Group II C75
YP 002364203.1|Thauera sp. MZ1T
ZP 09972784.1|Serratia sp. M24T3
YP 425037.1|Rhodospirillum rubrum ATCC 11170
YP 006965807.1|Sphingobium fuliginis ATCC 27551
ZP 09910204.1|Sphingobium yanoikuyae XLDN2-5
ZP 08389807.1|Sphingomonas sp. S17
YP 004451327.1|Haliscomenobacter hydrossis DSM 1100
YP 001953903.1|Geobacter lovleyi SZ
Rra3 p28 | Plasmid
ZP 08778927.1|Candidatus Odyssella thessalonicensis L13
ZP 03335657.1|Wolbachia endosymbiont of Culex quinquefasciatus JHB
ZP 09865655.1|Methylomicrobium album BG8
YP 003522601.1|Nitrosococcus halophilus Nc4
ZP 04580212.1|Helicobacter bilis ATCC 43879
ZP 04581456.1|Helicobacter bilis ATCC 43879
ZP 18209949.1|Campylobacter showae CSUNSWCD
ZP 03609888.1|Campylobacter rectus
YP 002576129.1|Campylobacter lari
ZP 18246598.1|Campylobacter fetus subsp. venerealis NCTC 10354
NP 907298.1|Wolinella succinogenes DSM 1740
YP 001456648.1|Campylobacter concisus 13826
ZP 14195693.1|Campylobacter jejuni subsp. jejuni 1997-7
ZP 07400676.1|Campylobacter coli JV20
ZP 04807998.1|Helicobacter pullorum MIT 98-5489
0.2

Figure A96. Neighbor-joining (NJ) and maximum likelihood (ML) trees of plasmid partitioning protein ParA. Bootstrap supports higher than or equal to 60% are shown on the branches.

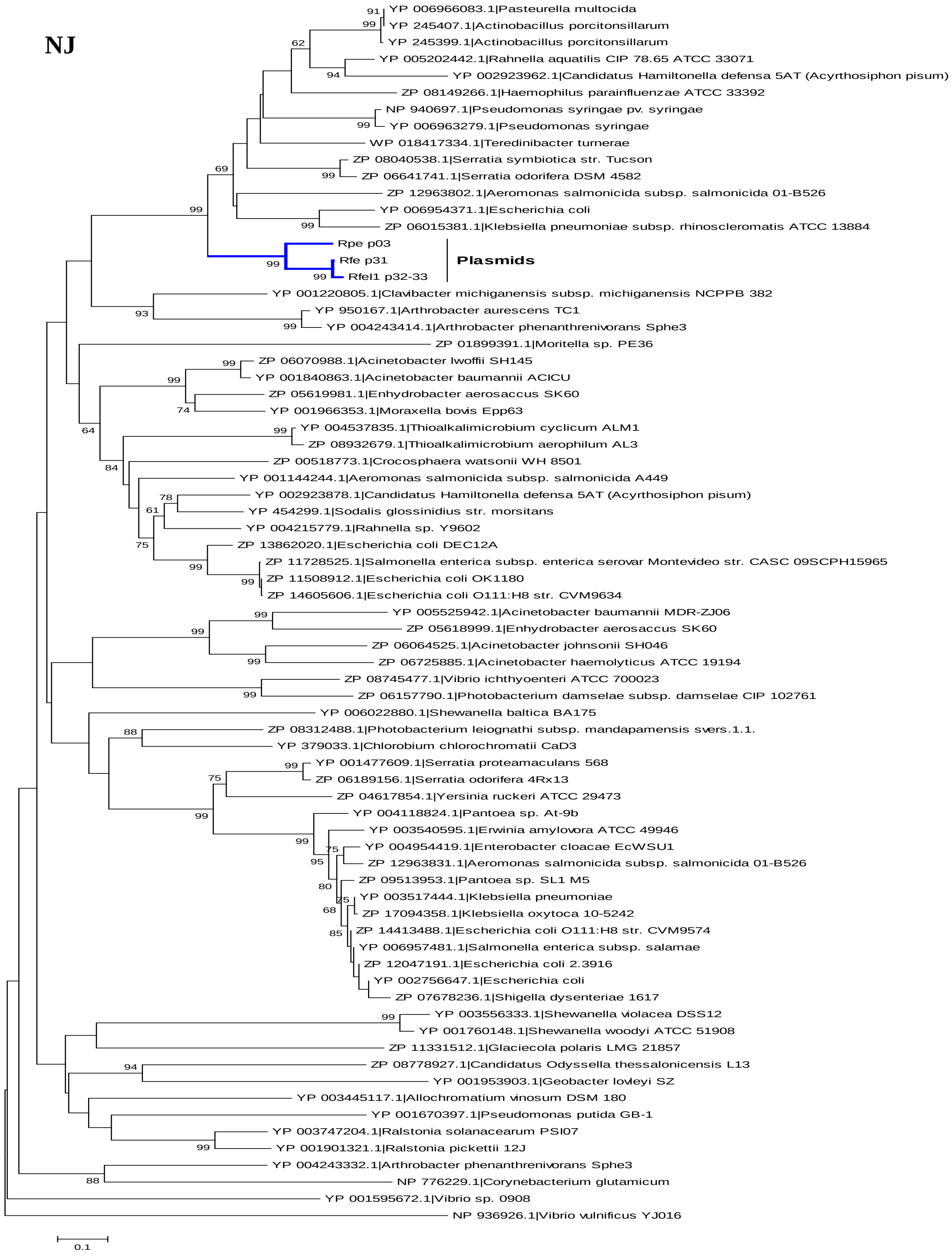

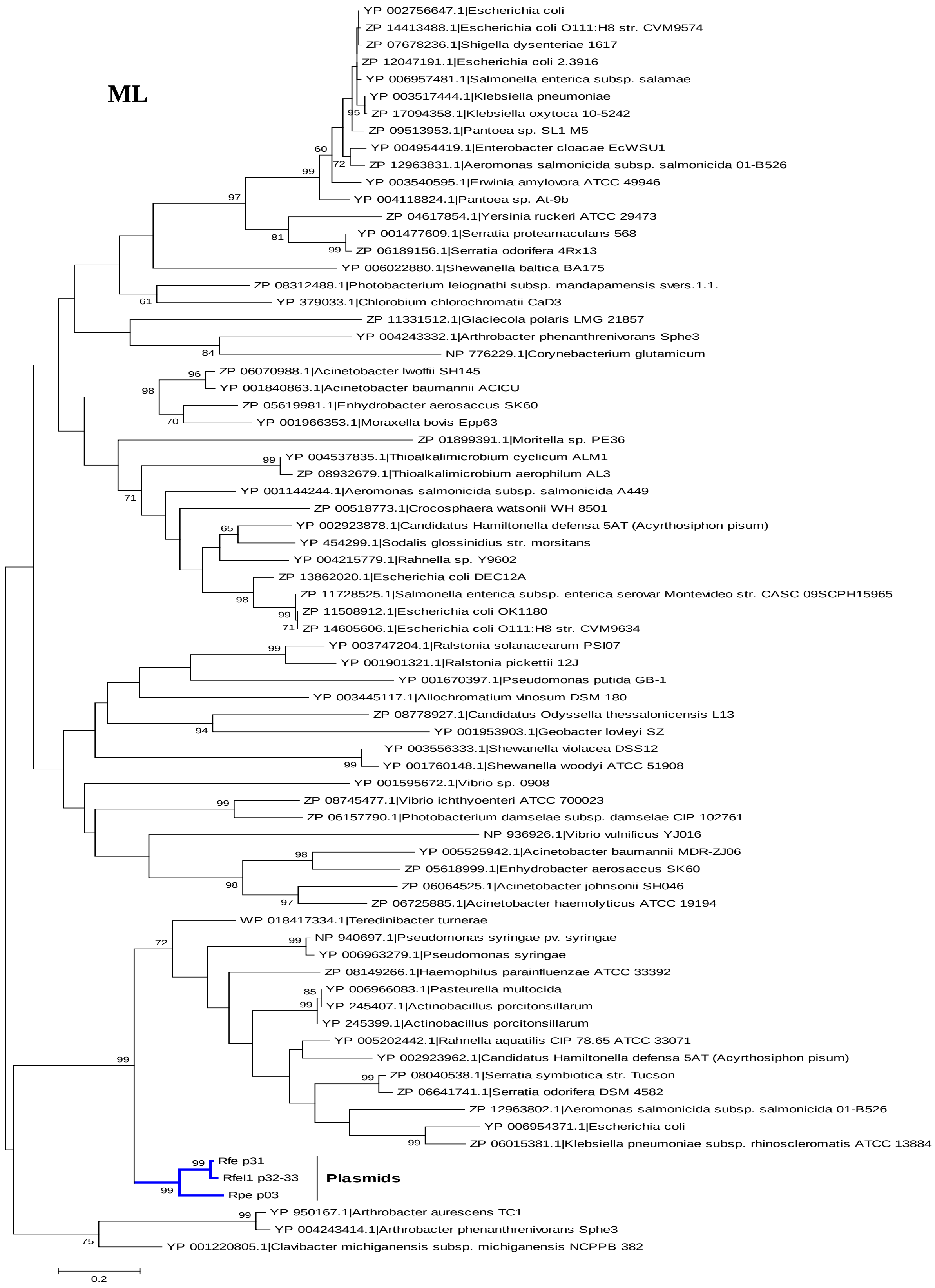
ML
YP 002756647.1|Escherichia coli
ZP 14413488.1|Escherichia coli O111:H8 str. CVM9574
ZP 07678236.1|Shigella dysenteriae 1617
ZP 12047191.1|Escherichia coli 2.3916
YP 006957481.1|Salmonella enterica subsp. salamae
YP 003517444.1|Klebsiella pneumoniae
ZP 17094358.1|Klebsiella oxytoca 10-5242
ZP 09513953.1|Pantoea sp. SL1 M5
YP 004954419.1|Enterobacter cloacae EcWSU1
ZP 12963831.1|Aeromonas salmonicida subsp. salmonicida 01-B526
YP 003540595.1|Erwinia amylovora ATCC 49946
YP 004118824.1|Pantoea sp. At-9b
ZP 04617854.1|Yersinia ruckeri ATCC 29473
YP 001477609.1|Serratia proteamaculans 568
ZP 06189156.1|Serratia odorifera 4Rx13
YP 006022880.1|Shewanella baltica BA175
ZP 08312488.1|Photobacterium leiognathi subsp. mandapamensis svers.1.1.
YP 379033.1|Chlorobium chlorochromatii CaD3
ZP 11331512.1|Glaciecola polaris LMG 21857
YP 004243332.1|Arthrobacter phenanthrenivorans Sphe3
NP 776229.1|Corynebacterium glutamicum
ZP 06070988.1|Acinetobacter lwoffii SH145
YP 001840863.1|Acinetobacter baumannii ACICU
ZP 05619981.1|Enhydrobacter aerosaccus SK60
YP 001966353.1|Moraxella bovis Epp63
ZP 01899391.1|Moritella sp. PE36
YP 004537835.1|Thioalkalimicrobium cyclicum ALM1
ZP 08932679.1|Thioalkalimicrobium aerophilum AL3
YP 001144244.1|Aeromonas salmonicida subsp. salmonicida A449
ZP 00518773.1|Crocosphaera watsonii WH 8501
YP 002923878.1|Candidatus Hamiltonella defensa 5AT (Acyrthosiphon pisum)
YP 454299.1|Sodalis glossinidius str. morsitans
YP 004215779.1|Rahnella sp. Y9602
ZP 13862020.1|Escherichia coli DEC12A
ZP 11728525.1|Salmonella enterica subsp. enterica serovar Montevideo str. CASC 09SCPH15965
ZP 11508912.1|Escherichia coli OK1180
ZP 14605606.1|Escherichia coli O111:H8 str. CVM9634
YP 003747204.1|Ralstonia solanacearum PSI07
YP 001901321.1|Ralstonia pickettii 12J
YP 001670397.1|Pseudomonas putida GB-1
YP 003445117.1|Allochromatium vinosum DSM 180
ZP 08778927.1|Candidatus Odyssella thessalonicensis L13
YP 001953903.1|Geobacter lovleyi SZ
YP 003556333.1|Shewanella violacea DSS12
YP 001760148.1|Shewanella woodyi ATCC 51908
YP 001595672.1|Vibrio sp. 0908
ZP 08745477.1|Vibrio ichthyoenteri ATCC 700023
ZP 06157790.1|Photobacterium damselae subsp. damselae CIP 102761
NP 936926.1|Vibrio vulnificus YJ016
YP 005525942.1|Acinetobacter baumannii MDR-ZJ06
ZP 05618999.1|Enhydrobacter aerosaccus SK60
ZP 06064525.1|Acinetobacter johnsonii SH046
ZP 06725885.1|Acinetobacter haemolyticus ATCC 19194
WP 018417334.1|Teredinibacter turnerae
NP 940697.1|Pseudomonas syringae pv. syringae
YP 006963279.1|Pseudomonas syringae
ZP 08149266.1|Haemophilus parainfluenzae ATCC 33392
YP 006966083.1|Pasteurella multocida
YP 245407.1|Actinobacillus porcitonsillarum
YP 245399.1|Actinobacillus porcitonsillarum
YP 005202442.1|Rahnella aquatilis CIP 78.65 ATCC 33071
YP 002923962.1|Candidatus Hamiltonella defensa 5AT (Acyrthosiphon pisum)
ZP 08040538.1|Serratia symbiotica str. Tucson
ZP 06641741.1|Serratia odorifera DSM 4582
ZP 12963802.1|Aeromonas salmonicida subsp. salmonicida 01-B526
YP 006954371.1|Escherichia coli
ZP 06015381.1|Klebsiella pneumoniae subsp. rhinoscleromatis ATCC 13884
Rfe p31
Rfel1 p32-33
Rpe p03
Plasmids
YP 950167.1|Arthrobacter aurescens TC1
YP 004243414.1|Arthrobacter phenanthrenivorans Sphe3
YP 001220805.1|Clavibacter michiganensis subsp. michiganensis NCPPB 382
0.2

Figure A97. Neighbor-joining (NJ) and maximum likelihood (ML) trees of plasmid partitioning protein ParA. Bootstrap supports higher than or equal to 60% are shown on the branches.

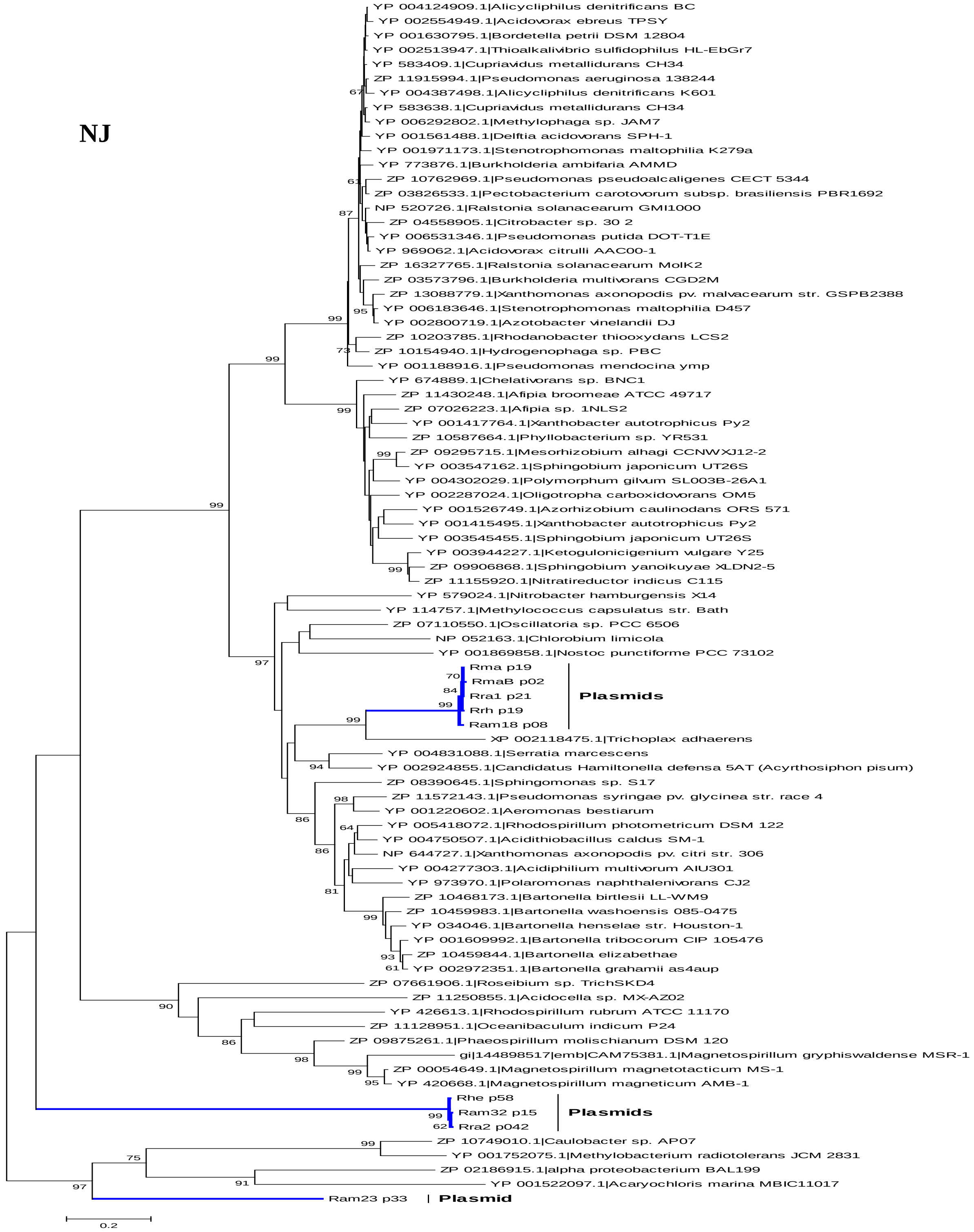

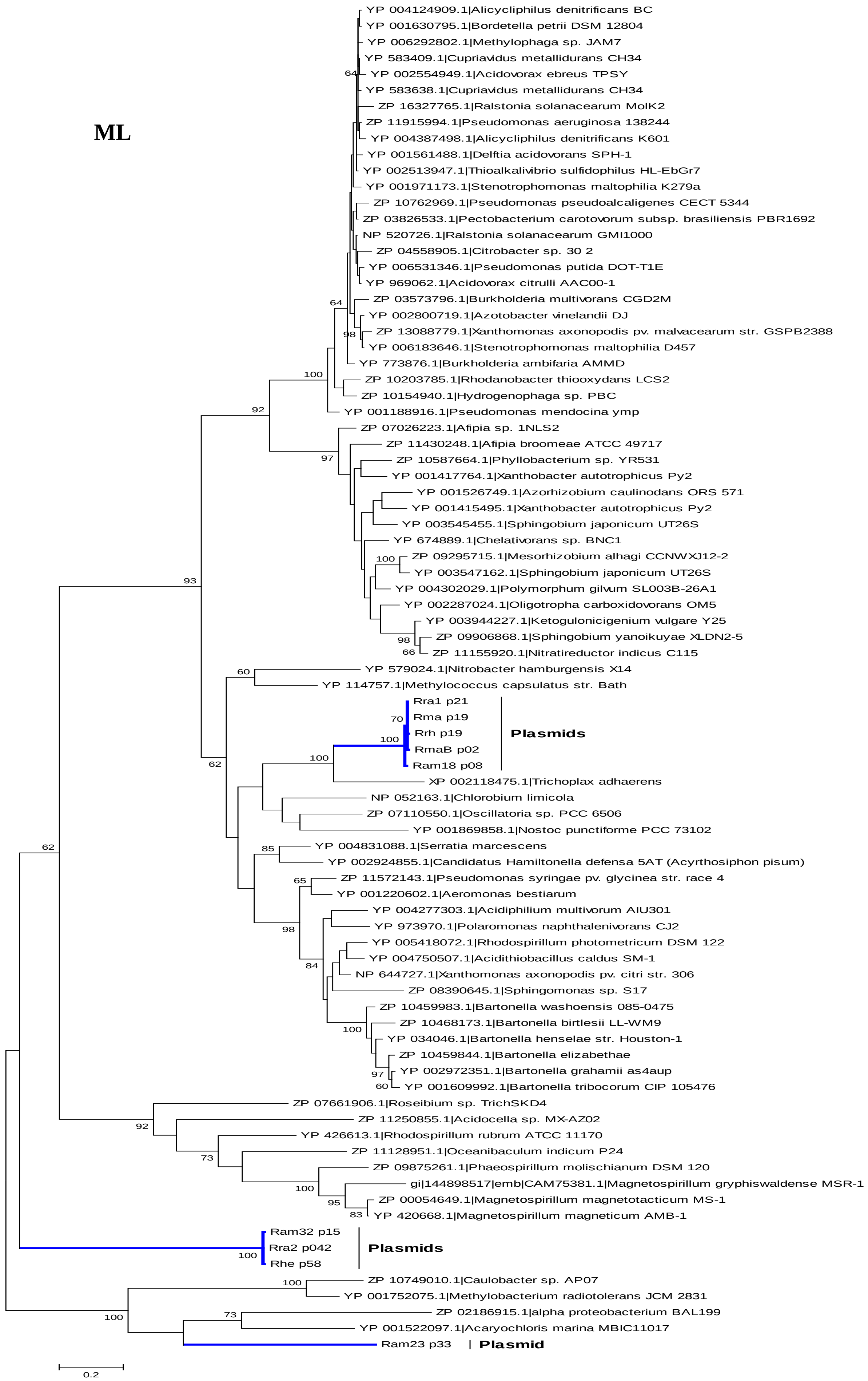
ML
YP 004124909.1|Alicycliphilus denitrificans BC
YP 001630795.1|Bordetella petrii DSM 12804
YP 006292802.1|Methylophaga sp. JAM7
YP 583409.1|Cupriavidus metallidurans CH34
YP 002554949.1|Acidovorax ebreus TPSY
YP 583638.1|Cupriavidus metallidurans CH34
ZP 16327765.1|Ralstonia solanacearum MolK2
ZP 11915994.1|Pseudomonas aeruginosa 138244
YP 004387498.1|Alicycliphilus denitrificans K601
YP 001561488.1|Delftia acidovorans SPH-1
YP 002513947.1|Thioalkalivibrio sulfidophilus HL-EbGr7
YP 001971173.1|Stenotrophomonas maltophilia K279a
ZP 10762969.1|Pseudomonas pseudoalcaligenes CECT 5344
ZP 03826533.1|Pectobacterium carotovorum subsp. brasiliensis PBR1692
NP 520726.1|Ralstonia solanacearum GMI1000
ZP 04558905.1|Citrobacter sp. 30 2
YP 006531346.1|Pseudomonas putida DOT-T1E
YP 969062.1|Acidovorax citrulli AAC00-1
ZP 03573796.1|Burkholderia multivorans CGD2M
YP 002800719.1|Azotobacter vinelandii DJ
ZP 13088779.1|Xanthomonas axonopodis pv. malvacearum str. GSPB2388
YP 006183646.1|Stenotrophomonas maltophilia D457
YP 773876.1|Burkholderia ambifaria AMMD
ZP 10203785.1|Rhodanobacter thiooxydans LCS2
ZP 10154940.1|Hydrogenophaga sp. PBC
YP 001188916.1|Pseudomonas mendocina ymp
ZP 07026223.1|Afipia sp. 1NLS2
ZP 11430248.1|Afipia broomeae ATCC 49717
ZP 10587664.1|Phyllobacterium sp. YR531
YP 001417764.1|Xanthobacter autotrophicus Py2
YP 001526749.1|Azorhizobium caulinodans ORS 571
YP 001415495.1|Xanthobacter autotrophicus Py2
YP 003545455.1|Sphingobium japonicum UT26S
YP 674889.1|Chelativorans sp. BNC1
ZP 09295715.1|Mesorhizobium alhagi CCNWXJ12-2
YP 003547162.1|Sphingobium japonicum UT26S
YP 004302029.1|Polymorphum gilvum SL003B-26A1
YP 002287024.1|Oligotropha carboxidovorans OM5
YP 003944227.1|Ketogulonicigenium vulgare Y25
ZP 09906868.1|Sphingobium yanoikuyae XLDN2-5
ZP 11155920.1|Nitratireductor indicus C115
YP 579024.1|Nitrobacter hamburgensis X14
YP 114757.1|Methylococcus capsulatus str. Bath
Rra1 p21
Rma p19
Rrh p19
RmaB p02
Ram18 p08
Plasmids
XP 002118475.1|Trichoplax adhaerens
NP 052163.1|Chlorobium limicola
ZP 07110550.1|Oscillatoria sp. PCC 6506
YP 001869858.1|Nostoc punctiforme PCC 73102
YP 004831088.1|Serratia marcescens
YP 002924855.1|Candidatus Hamiltonella defensa 5AT (Acyrthosiphon pisum)
ZP 11572143.1|Pseudomonas syringae pv. glycinea str. race 4
YP 001220602.1|Aeromonas bestiarum
YP 004277303.1|Acidiphilium multivorum AIU301
YP 973970.1|Polaromonas naphthalenivorans CJ2
YP 005418072.1|Rhodospirillum photometricum DSM 122
YP 004750507.1|Acidithiobacillus caldus SM-1
NP 644727.1|Xanthomonas axonopodis pv. citri str. 306
ZP 08390645.1|Sphingomonas sp. S17
ZP 10459983.1|Bartonella washoensis 085-0475
ZP 10468173.1|Bartonella birtlesii LL-WM9
YP 034046.1|Bartonella henselae str. Houston-1
ZP 10459844.1|Bartonella elizabethae
YP 002972351.1|Bartonella grahamii as4aup
YP 001609992.1|Bartonella tribocorum CIP 105476
ZP 07661906.1|Roseibium sp. TrichSKD4
ZP 11250855.1|Acidocella sp. MX-AZ02
YP 426613.1|Rhodospirillum rubrum ATCC 11170
ZP 11128951.1|Oceanibaculum indicum P24
ZP 09875261.1|Phaeospirillum molischianum DSM 120
gi|144898517|emb|CAM75381.1|Magnetospirillum gryphiswaldense MSR-1
ZP 00054649.1|Magnetospirillum magnetotacticum MS-1
YP 420668.1|Magnetospirillum magneticum AMB-1
Ram32 p15
Rra2 p042
Rhe p58
Plasmids
ZP 10749010.1|Caulobacter sp. AP07
YP 001752075.1|Methylobacterium radiotolerans JCM 2831
ZP 02186915.1|alpha proteobacterium BAL199
YP 001522097.1|Acaryochloris marina MBIC11017
Ram23 p33
Plasmid
64
64
98
100
92
97
93
100
98
66
60
70
100
100
62
62
85
65
98
84
100
97
60
92
73
100
95
83
100
100
100
73
0.2

Figure A98. Neighbor-joining (NJ) and maximum likelihood (ML) trees of plasmid partitioning protein ParA. Bootstrap supports higher than or equal to 60% are shown on the branches.

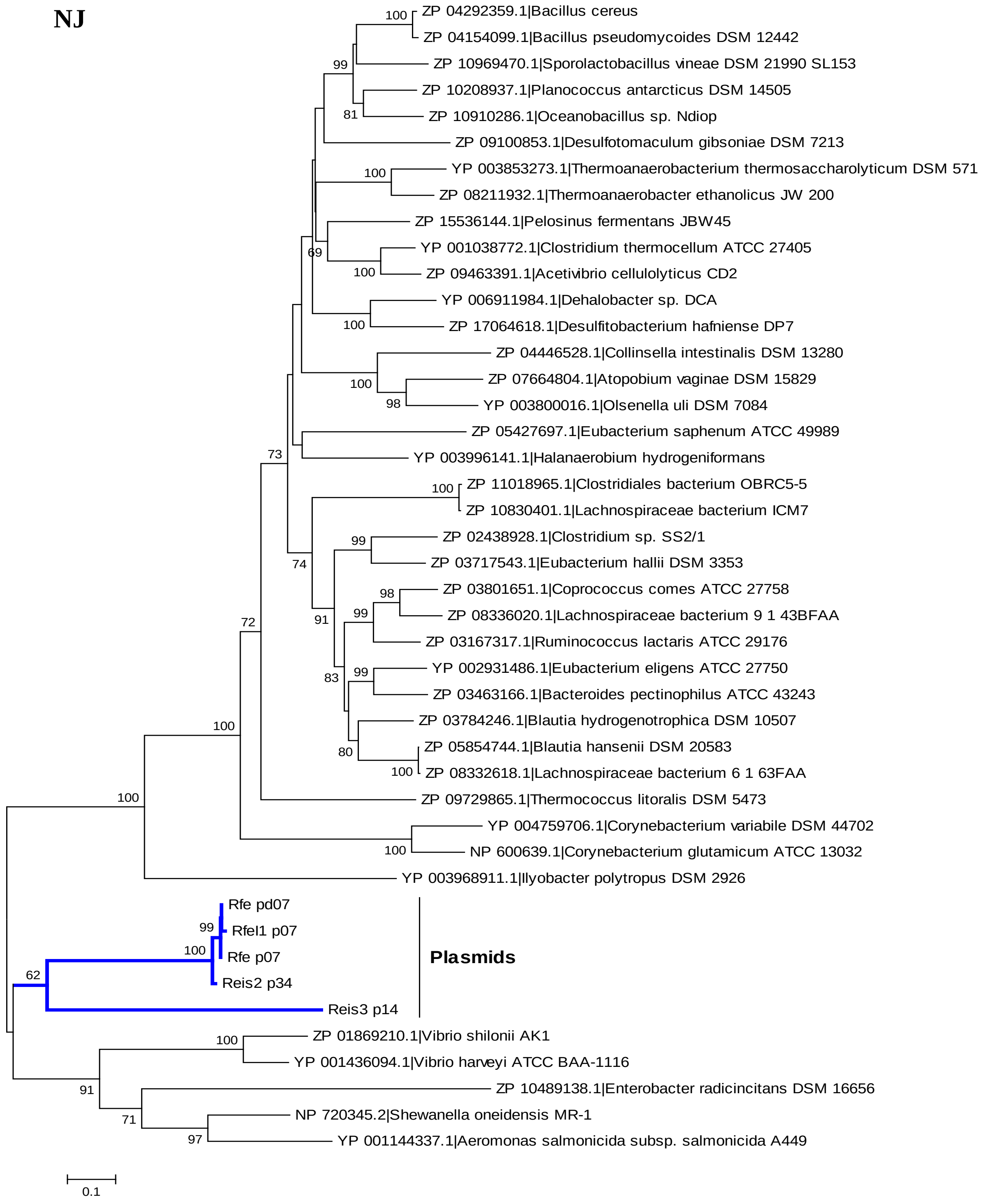

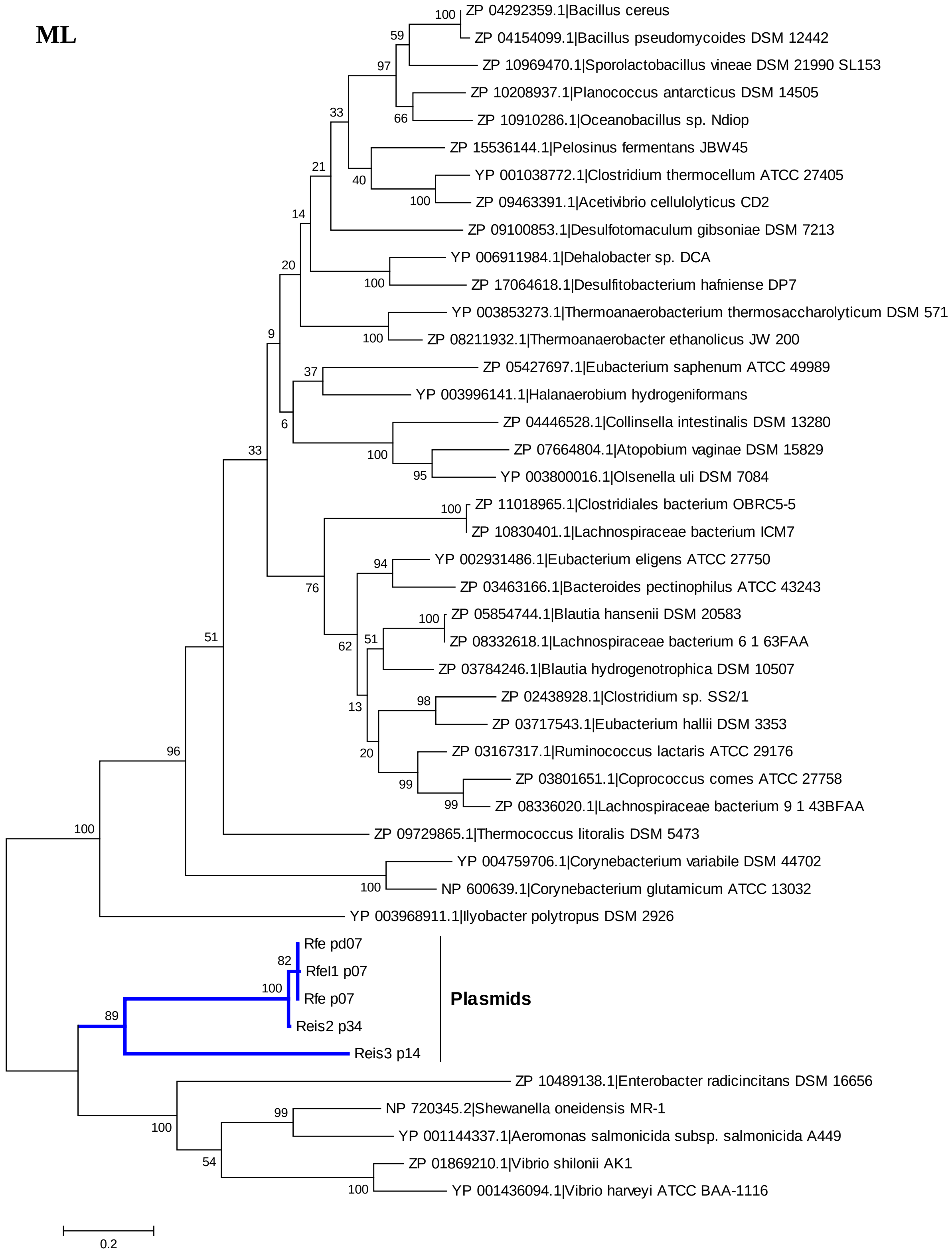
ML
ZP 04292359.1|Bacillus cereus
ZP 04154099.1|Bacillus pseudomycoides DSM 12442
ZP 10969470.1|Sporolactobacillus vineae DSM 21990 SL153
ZP 10208937.1|Planococcus antarcticus DSM 14505
ZP 10910286.1|Oceanobacillus sp. Ndiop
ZP 15536144.1|Pelosinus fermentans JBW45
YP 001038772.1|Clostridium thermocellum ATCC 27405
ZP 09463391.1|Acetivibrio cellulolyticus CD2
ZP 09100853.1|Desulfotomaculum gibsoniae DSM 7213
YP 006911984.1|Dehalobacter sp. DCA
ZP 17064618.1|Desulfitobacterium hafniense DP7
YP 003853273.1|Thermoanaerobacterium thermosaccharolyticum DSM 571
ZP 08211932.1|Thermoanaerobacter ethanolicus JW 200
ZP 05427697.1|Eubacterium saphenum ATCC 49989
YP 003996141.1|Halanaerobium hydrogeniformans
ZP 04446528.1|Collinsella intestinalis DSM 13280
ZP 07664804.1|Atopobium vaginae DSM 15829
YP 003800016.1|Olsenella uli DSM 7084
ZP 11018965.1|Clostridiales bacterium OBRC5-5
ZP 10830401.1|Lachnospiraceae bacterium ICM7
YP 002931486.1|Eubacterium eligens ATCC 27750
ZP 03463166.1|Bacteroides pectinophilus ATCC 43243
ZP 05854744.1|Blautia hansenii DSM 20583
ZP 08332618.1|Lachnospiraceae bacterium 6 1 63FAA
ZP 03784246.1|Blautia hydrogenotrophica DSM 10507
ZP 02438928.1|Clostridium sp. SS2/1
ZP 03717543.1|Eubacterium hallii DSM 3353
ZP 03167317.1|Ruminococcus lactaris ATCC 29176
ZP 03801651.1|Coprococcus comes ATCC 27758
ZP 08336020.1|Lachnospiraceae bacterium 9 1 43BFAA
ZP 09729865.1|Thermococcus litoralis DSM 5473
YP 004759706.1|Corynebacterium variabile DSM 44702
NP 600639.1|Corynebacterium glutamicum ATCC 13032
YP 003968911.1|Ilyobacter polytropus DSM 2926
Rfe pd07
Rfel1 p07
Rfe p07
Reis2 p34
Reis3 p14
Plasmids
ZP 10489138.1|Enterobacter radicincitans DSM 16656
NP 720345.2|Shewanella oneidensis MR-1
YP 001144337.1|Aeromonas salmonicida subsp. salmonicida A449
ZP 01869210.1|Vibrio shilonii AK1
YP 001436094.1|Vibrio harveyi ATCC BAA-1116
0.2

Figure A99. Neighbor-joining (NJ) and maximum likelihood (ML) trees of ParB-like nuclease domain-containing protein. Bootstrap supports higher than or equal to 60% are shown on the branches.

NJ

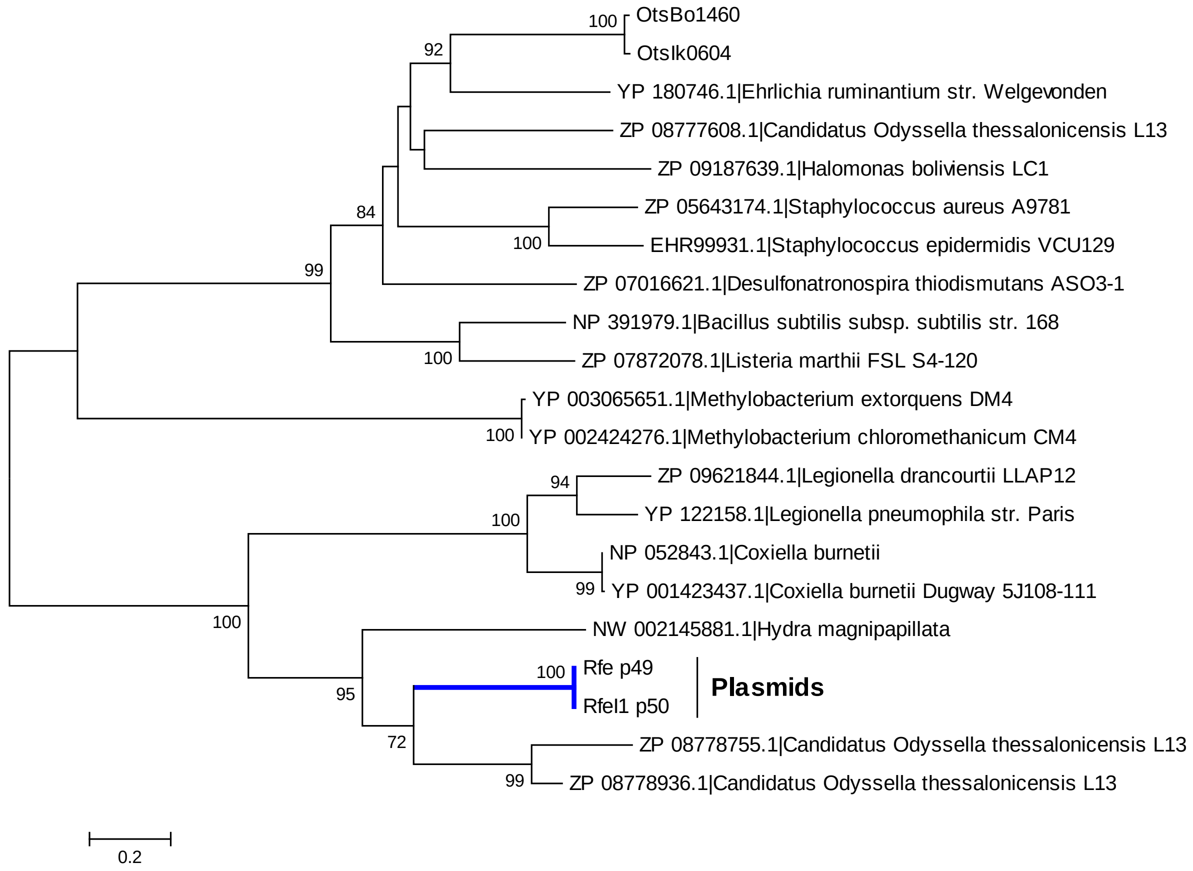

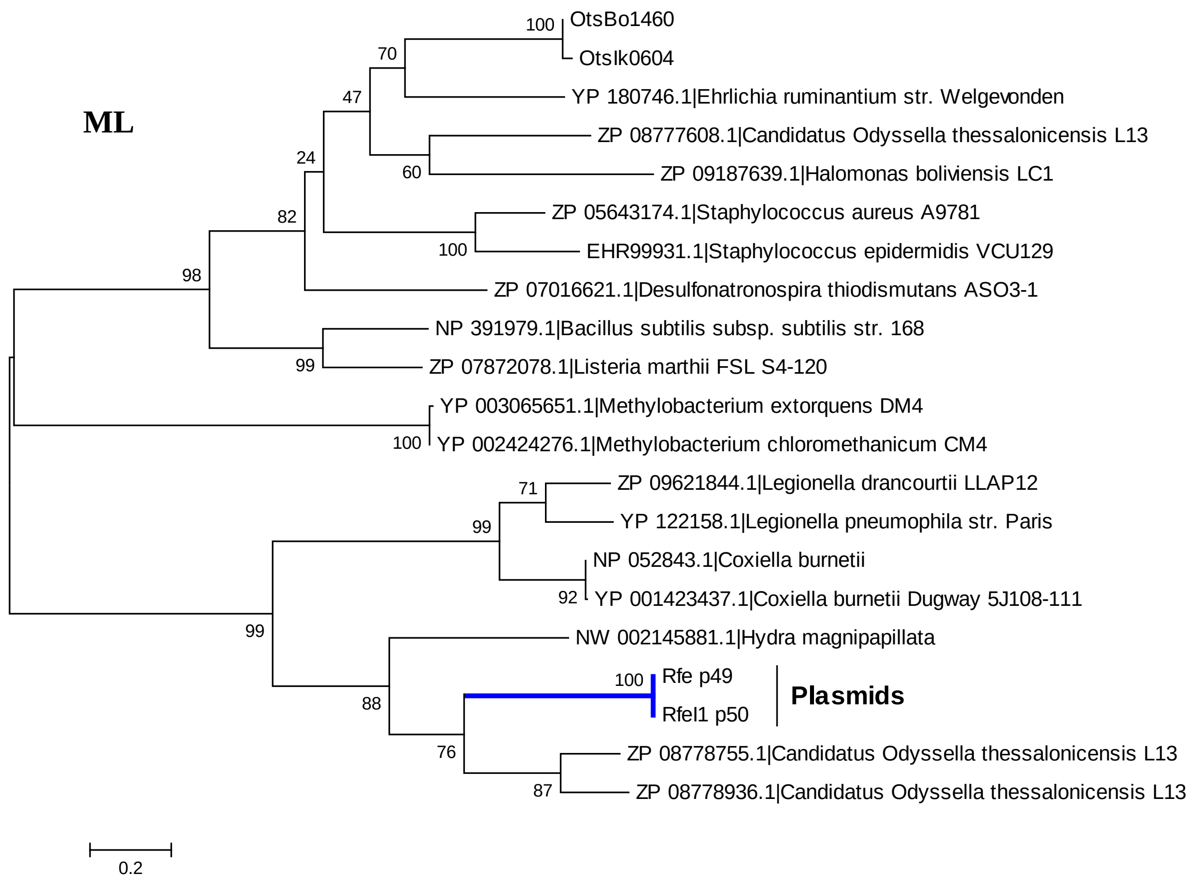

ML
OtsBo1460
OtsIk0604
YP 180746.1|Ehrlichia ruminantium str. Welgevonden
ZP 08777608.1|Candidatus Odyssella thessalonicensis L13
ZP 09187639.1|Halomonas boliviensis LC1
ZP 05643174.1|Staphylococcus aureus A9781
EHR99931.1|Staphylococcus epidermidis VCU129
ZP 07016621.1|Desulfonatronospira thiodismutans ASO3-1
NP 391979.1|Bacillus subtilis subsp. subtilis str. 168
ZP 07872078.1|Listeria marthii FSL S4-120
YP 003065651.1|Methylobacterium extorquens DM4
YP 002424276.1|Methylobacterium chloromethanicum CM4
ZP 09621844.1|Legionella drancourtii LLAP12
YP 122158.1|Legionella pneumophila str. Paris
NP 052843.1|Coxiella burnetii
YP 001423437.1|Coxiella burnetii Dugway 5J108-111
NW 002145881.1|Hydra magnipapillata
Rfe p49
Rfel1 p50
Plasmids
ZP 08778755.1|Candidatus Odyssella thessalonicensis L13
ZP 08778936.1|Candidatus Odyssella thessalonicensis L13
100
70
47
24
60
82
100
98
99
100
71
99
92
99
100
88
76
87
0.2

Figure A100. Neighbor-joining (NJ) and maximum likelihood (ML) trees of resolvase protein containing HTH_7 domain. Bootstrap supports higher than or equal to 60% are shown on the branches.

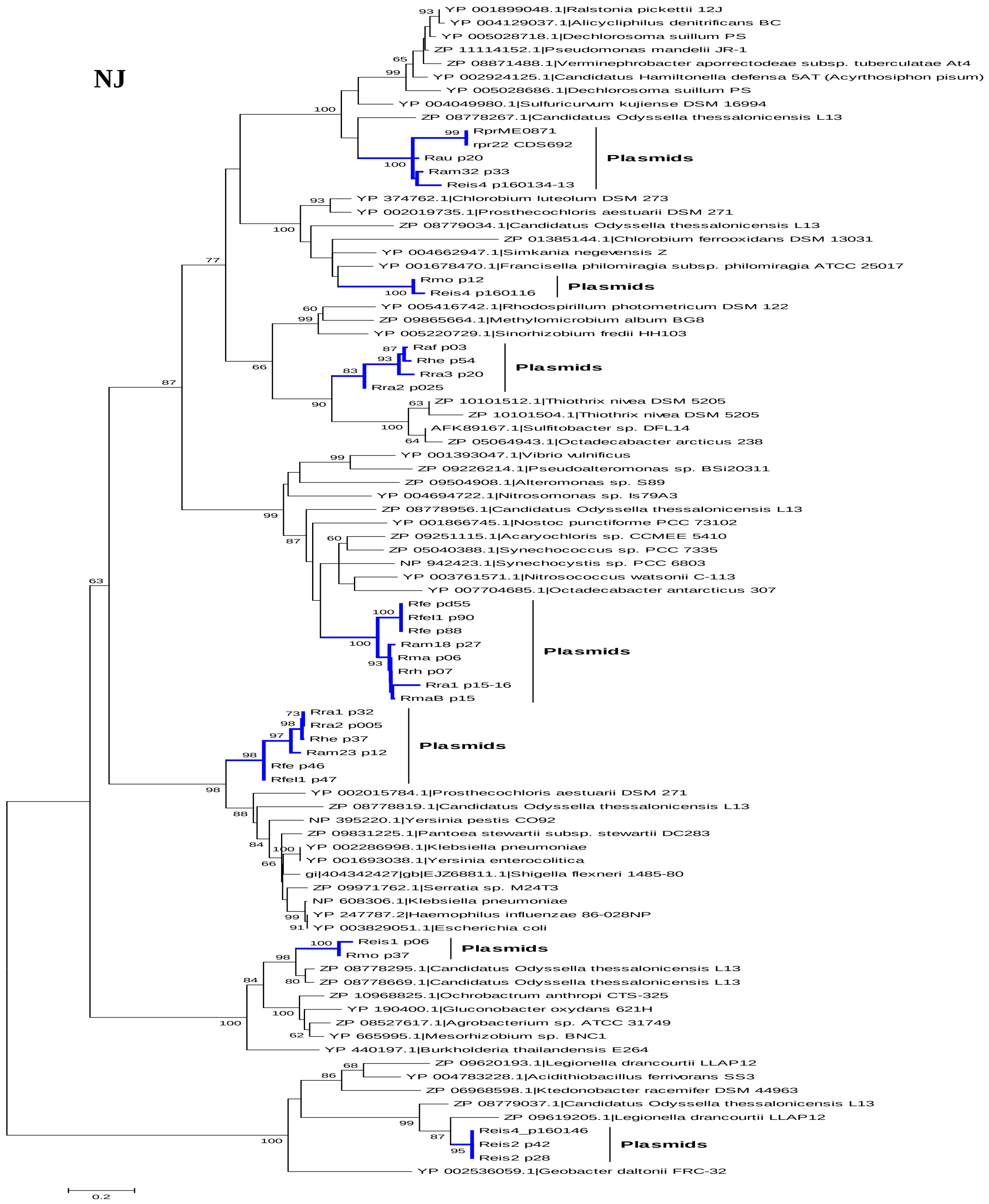

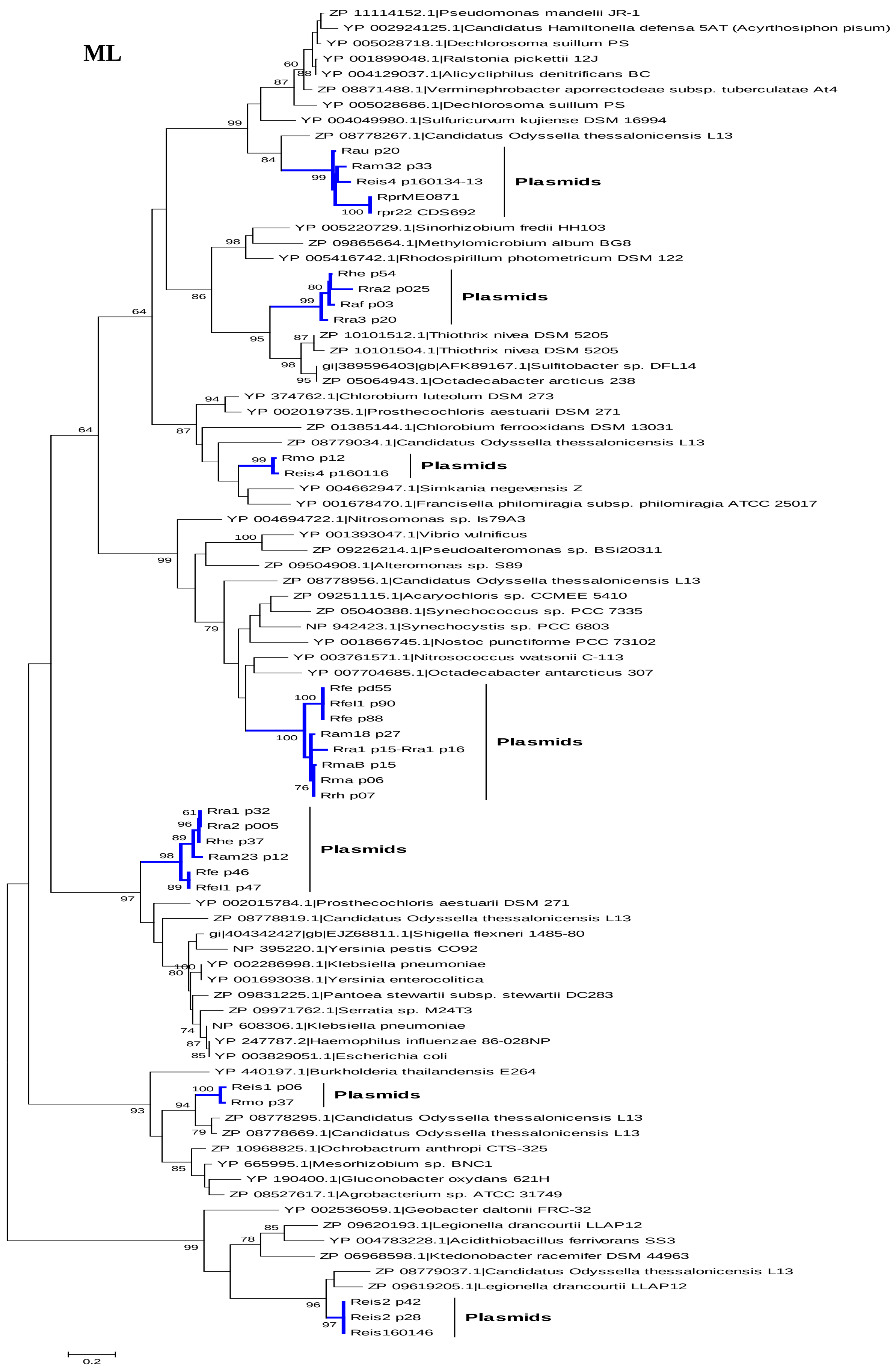
ML
ZP 11114152.1|Pseudomonas mandelii JR-1
YP 002924125.1|Candidatus Hamiltonella defensa 5AT (Acyrthosiphon pisum)
YP 005028718.1|Dechlorosoma suillum PS
YP 001899048.1|Ralstonia pickettii 12J
YP 004129037.1|Alicycliphilus denitrificans BC
ZP 08871488.1|Verminephrobacter aporrectodeae subsp. tuberculatae At4
YP 005028686.1|Dechlorosoma suillum PS
YP 004049980.1|Sulfuricurvum kujiense DSM 16994
ZP 08778267.1|Candidatus Odyssella thessalonicensis L13
Rau p20
Ram32 p33
Reis4 p160134-13
RprME0871
rpr22 CDS692
Plasmids
YP 005220729.1|Sinorhizobium fredii HH103
ZP 09865664.1|Methylomicrobium album BG8
YP 005416742.1|Rhodospirillum photometricum DSM 122
Rhe p54
Rra2 p025
Raf p03
Rra3 p20
Plasmids
ZP 10101512.1|Thiothrix nivea DSM 5205
ZP 10101504.1|Thiothrix nivea DSM 5205
gi|389596403|gb|AFK89167.1|Sulfitobacter sp. DFL14
ZP 05064943.1|Octadecabacter arcticus 238
YP 374762.1|Chlorobium luteolum DSM 273
YP 002019735.1|Prosthecochloris aestuarii DSM 271
ZP 01385144.1|Chlorobium ferrooxidans DSM 13031
ZP 08779034.1|Candidatus Odyssella thessalonicensis L13
Rmo p12
Reis4 p160116
Plasmids
YP 004662947.1|Simkania negevensis Z
YP 001678470.1|Francisella philomiragia subsp. philomiragia ATCC 25017
YP 004694722.1|Nitrosomonas sp. Is79A3
YP 001393047.1|Vibrio vulnificus
ZP 09226214.1|Pseudoalteromonas sp. BSi20311
ZP 09504908.1|Alteromonas sp. S89
ZP 08778956.1|Candidatus Odyssella thessalonicensis L13
ZP 09251115.1|Acaryochloris sp. CCMEE 5410
ZP 05040388.1|Synechococcus sp. PCC 7335
NP 942423.1|Synechocystis sp. PCC 6803
YP 001866745.1|Nostoc punctiforme PCC 73102
YP 003761571.1|Nitrosococcus watsonii C-113
YP 007704685.1|Octadecabacter antarcticus 307
Rfe pd55
Rfel1 p90
Rfe p88
Ram18 p27
Rra1 p15-Rra1 p16
RmaB p15
Rma p06
Rrh p07
Plasmids
Rra1 p32
Rra2 p005
Rhe p37
Ram23 p12
Rfe p46
Rfel1 p47
Plasmids
YP 002015784.1|Prosthecochloris aestuarii DSM 271
ZP 08778819.1|Candidatus Odyssella thessalonicensis L13
gi|404342427|gb|EJZ68811.1|Shigella flexneri 1485-80
NP 395220.1|Yersinia pestis CO92
YP 002286998.1|Klebsiella pneumoniae
YP 001693038.1|Yersinia enterocolitica
ZP 09831225.1|Pantoea stewartii subsp. stewartii DC283
ZP 09971762.1|Serratia sp. M24T3
NP 608306.1|Klebsiella pneumoniae
YP 247787.2|Haemophilus influenzae 86-028NP
YP 003829051.1|Escherichia coli
YP 440197.1|Burkholderia thailandensis E264
Reis1 p06
Rmo p37
Plasmids
ZP 08778295.1|Candidatus Odyssella thessalonicensis L13
ZP 08778669.1|Candidatus Odyssella thessalonicensis L13
ZP 10968825.1|Ochrobactrum anthropi CTS-325
YP 665995.1|Mesorhizobium sp. BNC1
YP 190400.1|Gluconobacter oxydans 621H
ZP 08527617.1|Agrobacterium sp. ATCC 31749
YP 002536059.1|Geobacter daltonii FRC-32
ZP 09620193.1|Legionella drancourtii LLAP12
YP 004783228.1|Acidithiobacillus ferrivorans SS3
ZP 06968598.1|Ktedonobacter racemifer DSM 44963
ZP 08779037.1|Candidatus Odyssella thessalonicensis L13
ZP 09619205.1|Legionella drancourtii LLAP12
Reis2 p42
Reis2 p28
Reis160146
Plasmids
0.2

Figure A101. Neighbor-joining (NJ) and maximum likelihood (ML) trees of transposase_IS240 / integrase containing rve domain. Bootstrap supports higher than or equal to 60% are shown on the branches.

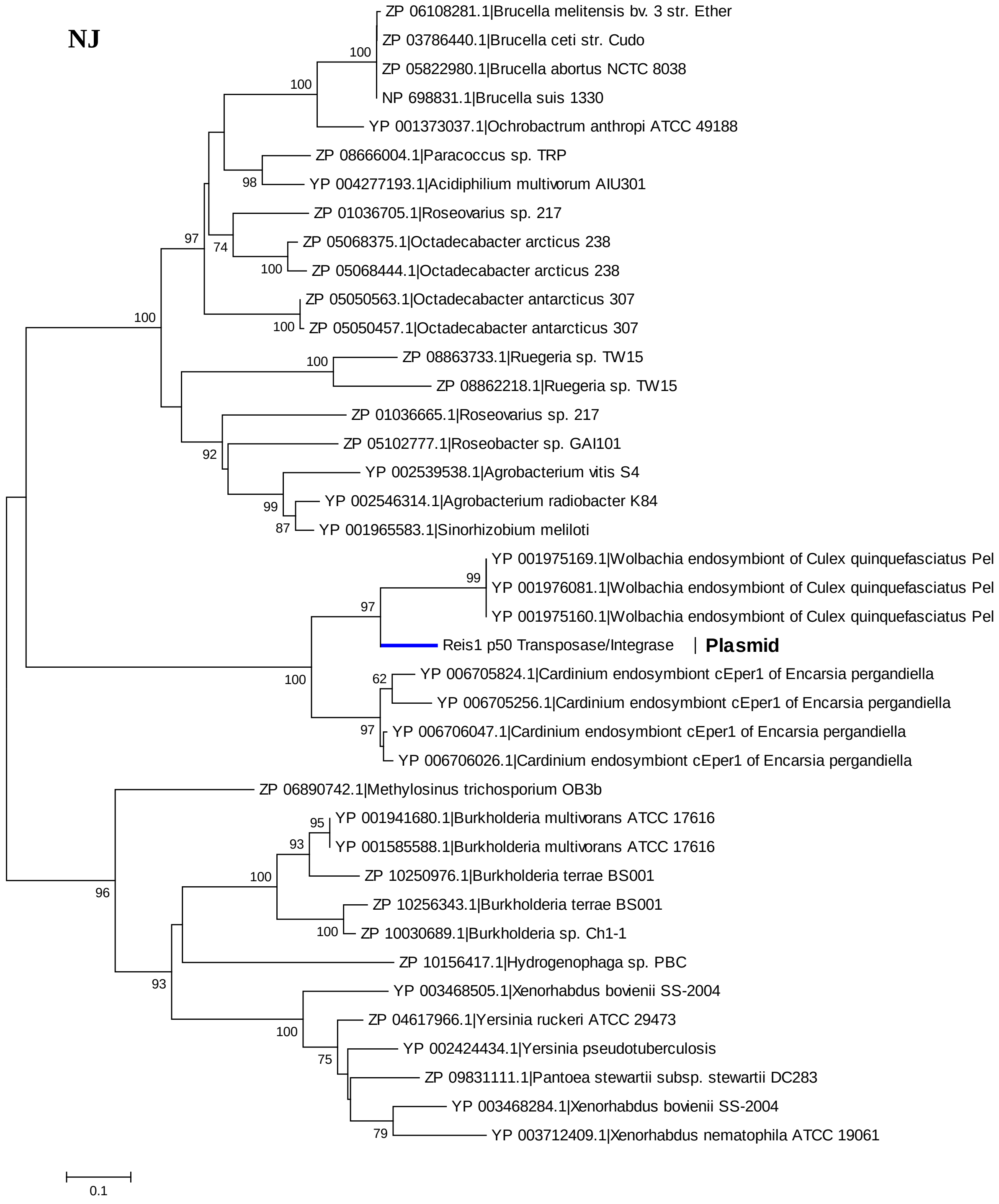

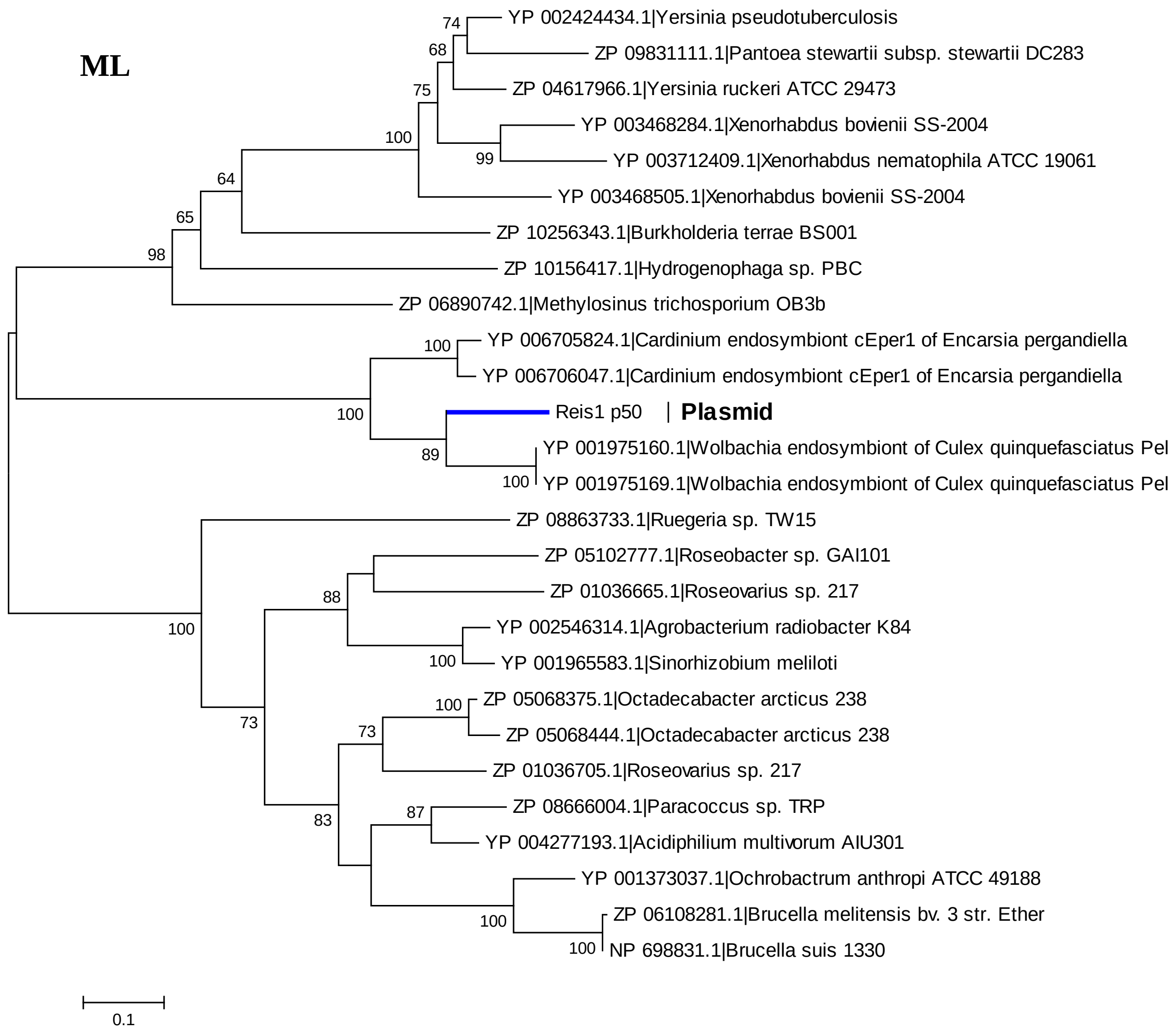
ML
YP 002424434.1|Yersinia pseudotuberculosis
ZP 09831111.1|Pantoea stewartii subsp. stewartii DC283
ZP 04617966.1|Yersinia ruckeri ATCC 29473
YP 003468284.1|Xenorhabdus bovienii SS-2004
YP 003712409.1|Xenorhabdus nematophila ATCC 19061
YP 003468505.1|Xenorhabdus bovienii SS-2004
ZP 10256343.1|Burkholderia terrae BS001
ZP 10156417.1|Hydrogenophaga sp. PBC
ZP 06890742.1|Methylosinus trichosporium OB3b
YP 006705824.1|Cardinium endosymbiont cEper1 of Encarsia pergandiella
YP 006706047.1|Cardinium endosymbiont cEper1 of Encarsia pergandiella
Reis1 p50 | Plasmid
YP 001975160.1|Wolbachia endosymbiont of Culex quinquefasciatus Pel
YP 001975169.1|Wolbachia endosymbiont of Culex quinquefasciatus Pel
ZP 08863733.1|Ruegeria sp. TW15
ZP 05102777.1|Roseobacter sp. GAI101
ZP 01036665.1|Roseovarius sp. 217
YP 002546314.1|Agrobacterium radiobacter K84
YP 001965583.1|Sinorhizobium meliloti
ZP 05068375.1|Octadecabacter arcticus 238
ZP 05068444.1|Octadecabacter arcticus 238
ZP 01036705.1|Roseovarius sp. 217
ZP 08666004.1|Paracoccus sp. TRP
YP 004277193.1|Acidiphilium multivorum AIU301
YP 001373037.1|Ochrobactrum anthropi ATCC 49188
ZP 06108281.1|Brucella melitensis bv. 3 str. Ether
NP 698831.1|Brucella suis 1330
74
68
75
100
99
64
65
98
100
100
89
100
100
88
100
100
73
73
83
87
100
100
0.1

Figure A102. Neighbor-joining (NJ) and maximum likelihood (ML) trees of transposase mutator family, containing MULE domain. Bootstrap supports higher than or equal to 60% are shown on the branches.

NJ

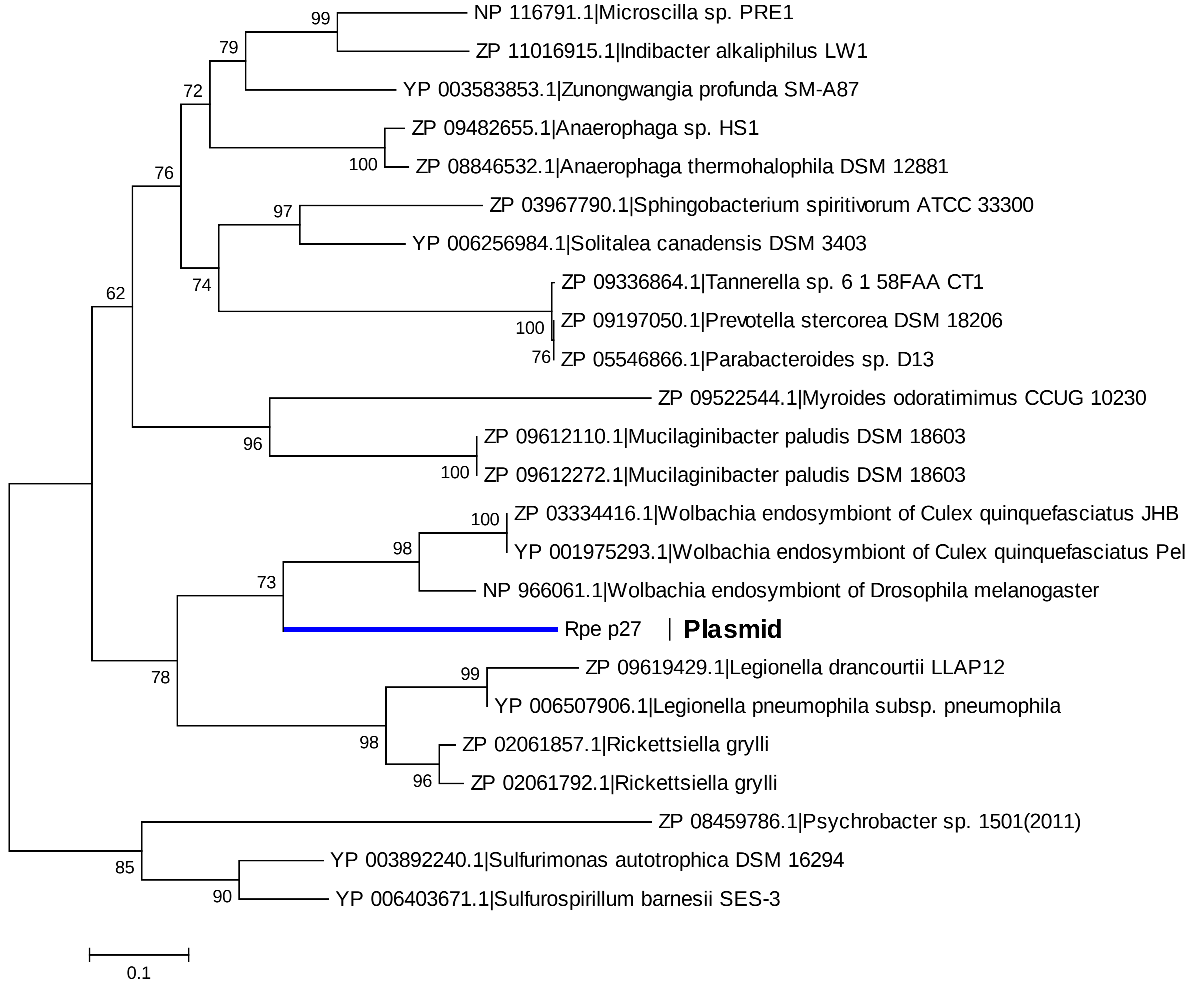

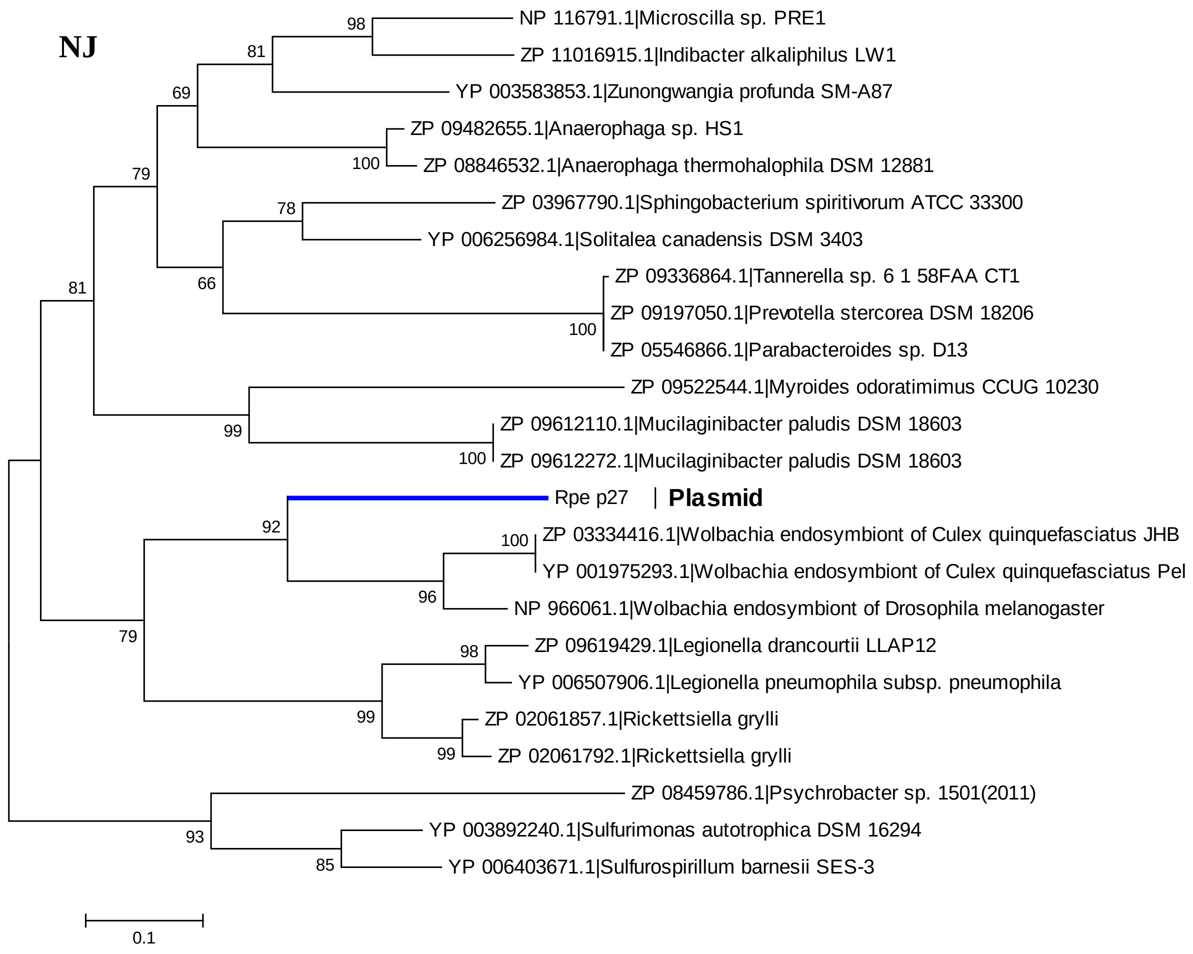
NJ
NP 116791.1|Microscilla sp. PRE1
ZP 11016915.1|Indibacter alkaliphilus LW1
YP 003583853.1|Zunongwangia profunda SM-A87
ZP 09482655.1|Anaerophaga sp. HS1
ZP 08846532.1|Anaerophaga thermohalophila DSM 12881
ZP 03967790.1|Sphingobacterium spiritivorum ATCC 33300
YP 006256984.1|Solitalea canadensis DSM 3403
ZP 09336864.1|Tannerella sp. 6 1 58FAA CT1
ZP 09197050.1|Prevotella stercorea DSM 18206
ZP 05546866.1|Parabacteroides sp. D13
ZP 09522544.1|Myroides odoratimimus CCUG 10230
ZP 09612110.1|Mucilaginibacter paludis DSM 18603
ZP 09612272.1|Mucilaginibacter paludis DSM 18603
Rpe p27
Plasmid
ZP 03334416.1|Wolbachia endosymbiont of Culex quinquefasciatus JHB
YP 001975293.1|Wolbachia endosymbiont of Culex quinquefasciatus Pel
NP 966061.1|Wolbachia endosymbiont of Drosophila melanogaster
ZP 09619429.1|Legionella drancourtii LLAP12
YP 006507906.1|Legionella pneumophila subsp. pneumophila
ZP 02061857.1|Rickettsiella grylli
ZP 02061792.1|Rickettsiella grylli
ZP 08459786.1|Psychrobacter sp. 1501(2011)
YP 003892240.1|Sulfurimonas autotrophica DSM 16294
YP 006403671.1|Sulfurospirillum barnesii SES-3
0.1

Figure A103. Neighbor-joining (NJ) and maximum likelihood (ML) trees of transposase containing DUF4158 and DDE_Tn3 domains. Bootstrap supports higher than or equal to 60% are shown on the branches.

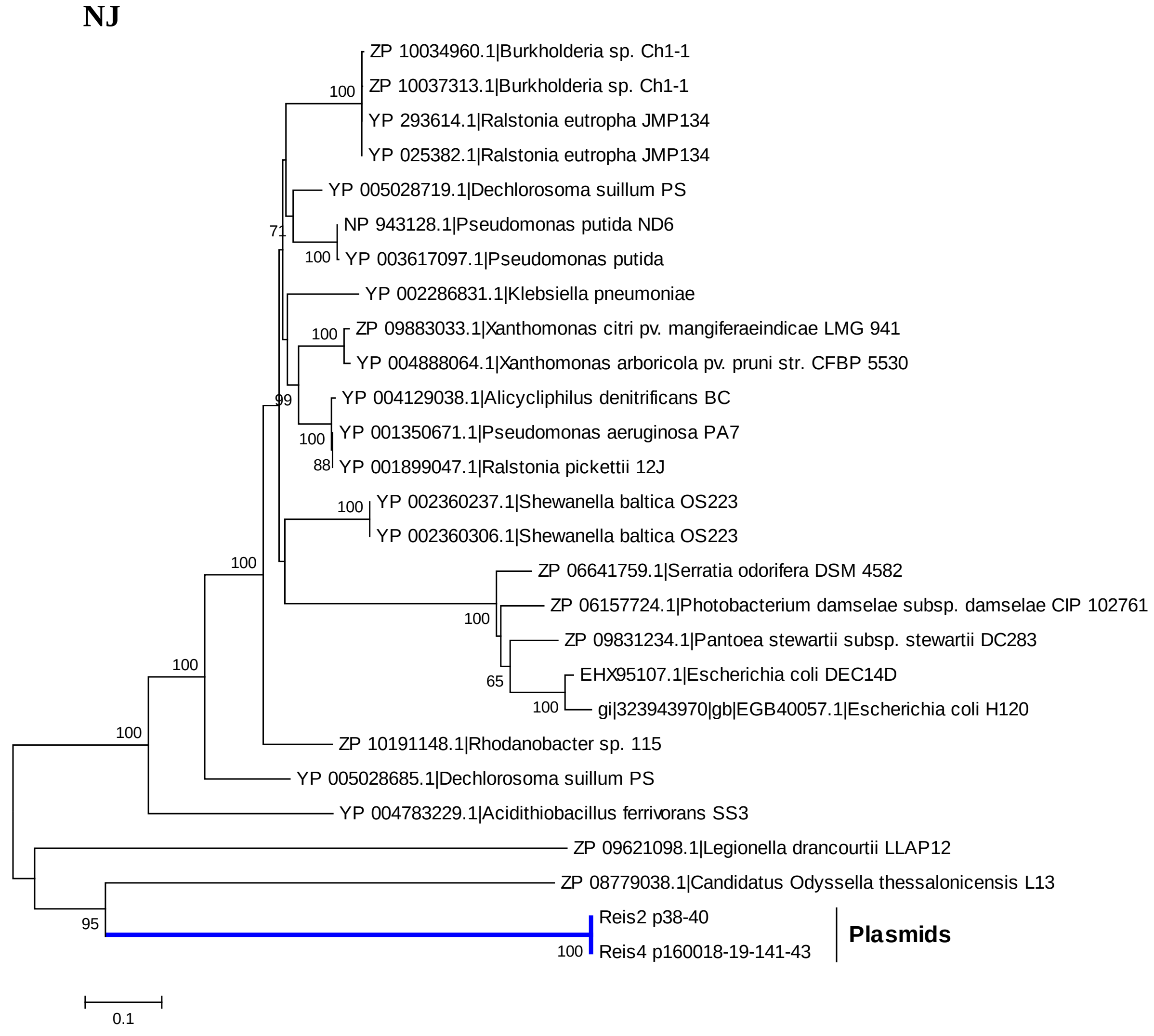

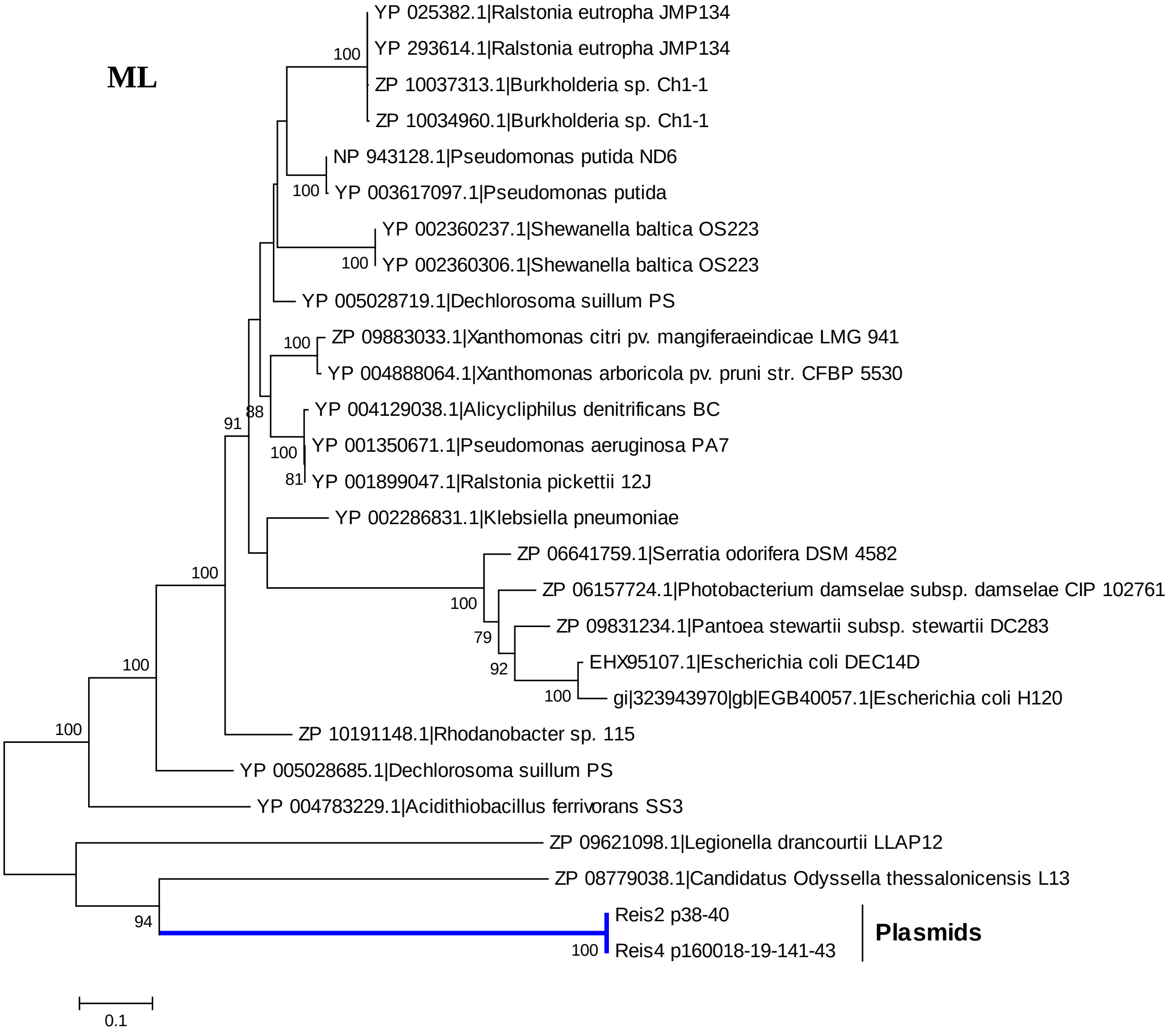
ML
YP 025382.1|Ralstonia eutropha JMP134
YP 293614.1|Ralstonia eutropha JMP134
ZP 10037313.1|Burkholderia sp. Ch1-1
ZP 10034960.1|Burkholderia sp. Ch1-1
NP 943128.1|Pseudomonas putida ND6
YP 003617097.1|Pseudomonas putida
YP 002360237.1|Shewanella baltica OS223
YP 002360306.1|Shewanella baltica OS223
YP 005028719.1|Dechlorosoma suillum PS
ZP 09883033.1|Xanthomonas citri pv. mangiferaeindicae LMG 941
YP 004888064.1|Xanthomonas arboricola pv. pruni str. CFBP 5530
YP 004129038.1|Alicycliphilus denitrificans BC
YP 001350671.1|Pseudomonas aeruginosa PA7
YP 001899047.1|Ralstonia pickettii 12J
YP 002286831.1|Klebsiella pneumoniae
ZP 06641759.1|Serratia odorifera DSM 4582
ZP 06157724.1|Photobacterium damselae subsp. damselae CIP 102761
ZP 09831234.1|Pantoea stewartii subsp. stewartii DC283
EHX95107.1|Escherichia coli DEC14D
gi|323943970|gb|EGB40057.1|Escherichia coli H120
ZP 10191148.1|Rhodanobacter sp. 115
YP 005028685.1|Dechlorosoma suillum PS
YP 004783229.1|Acidithiobacillus ferrivorans SS3
ZP 09621098.1|Legionella drancourtii LLAP12
ZP 08779038.1|Candidatus Odyssella thessalonicensis L13
Reis2 p38-40
Reis4 p160018-19-141-43
Plasmids
100
100
100
88
91
100
81
100
79
92
100
100
100
100
94
100
0.1

Figure A104. Neighbor-joining (NJ) and maximum likelihood (ML) trees of transposase containing DDE_IS66_C domain. Bootstrap supports higher than or equal to 60% are shown on the branches.

NJ

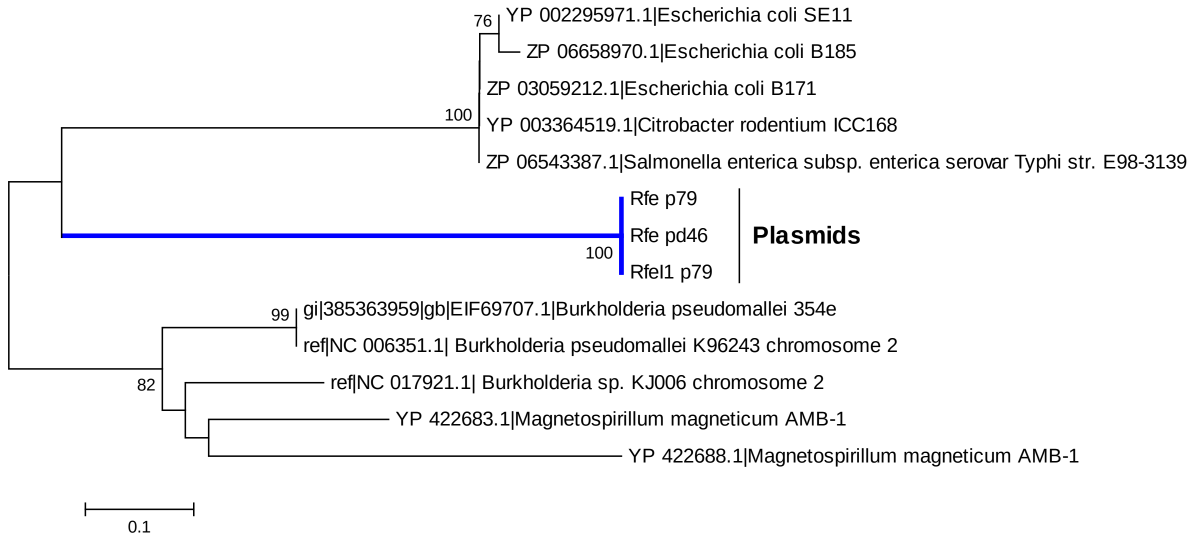

ML

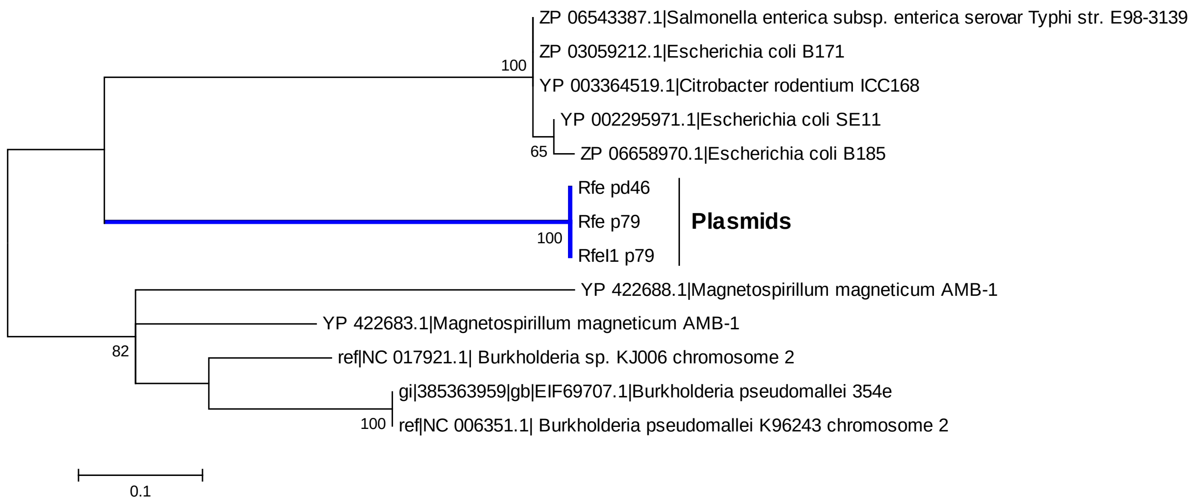

Figure A105. Neighbor-joining (NJ) and maximum likelihood (ML) trees of transposase/integrase containing HTH_28/32 and rve domains. Bootstrap supports higher than or equal to 60% are shown on the branches.

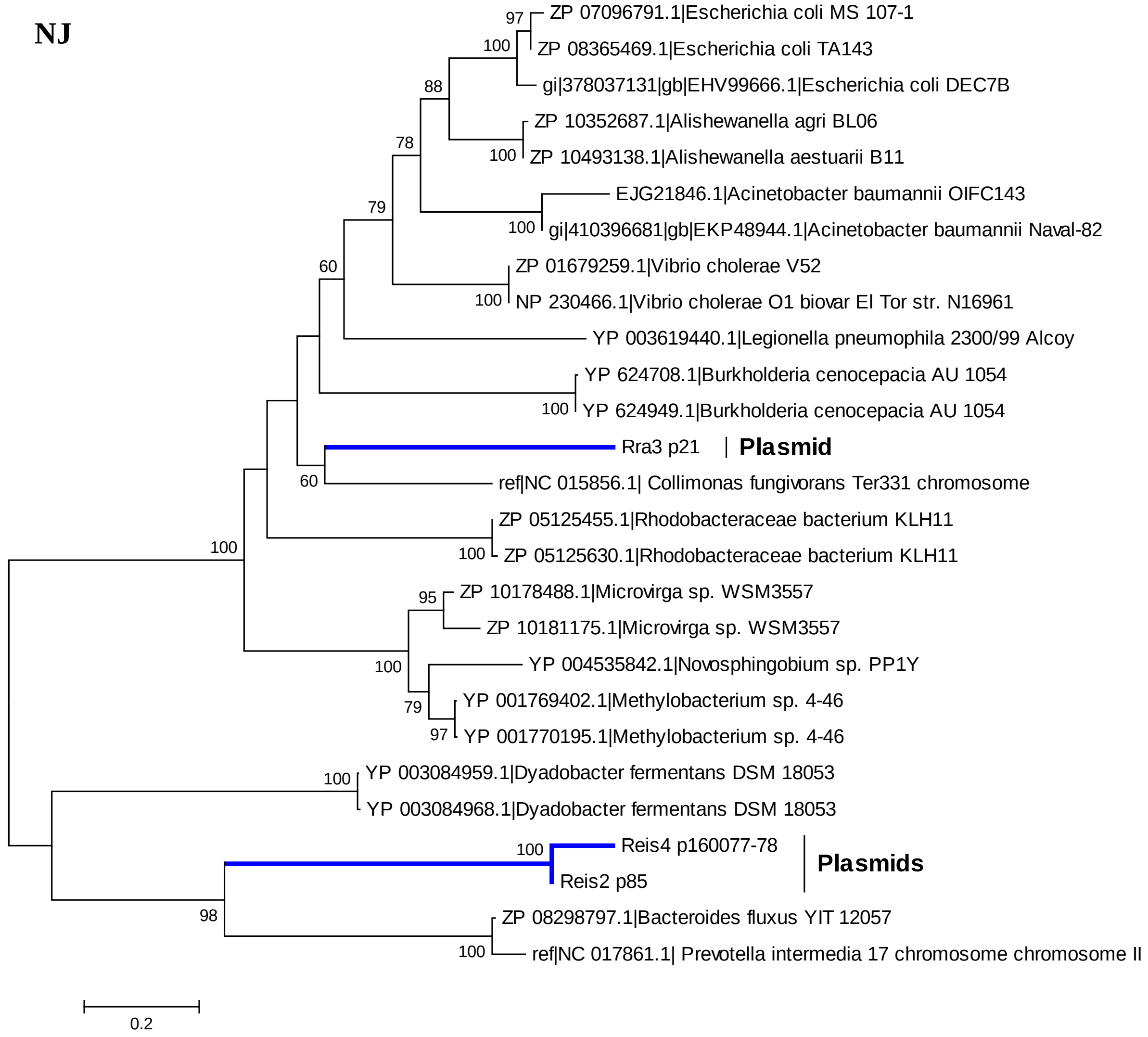

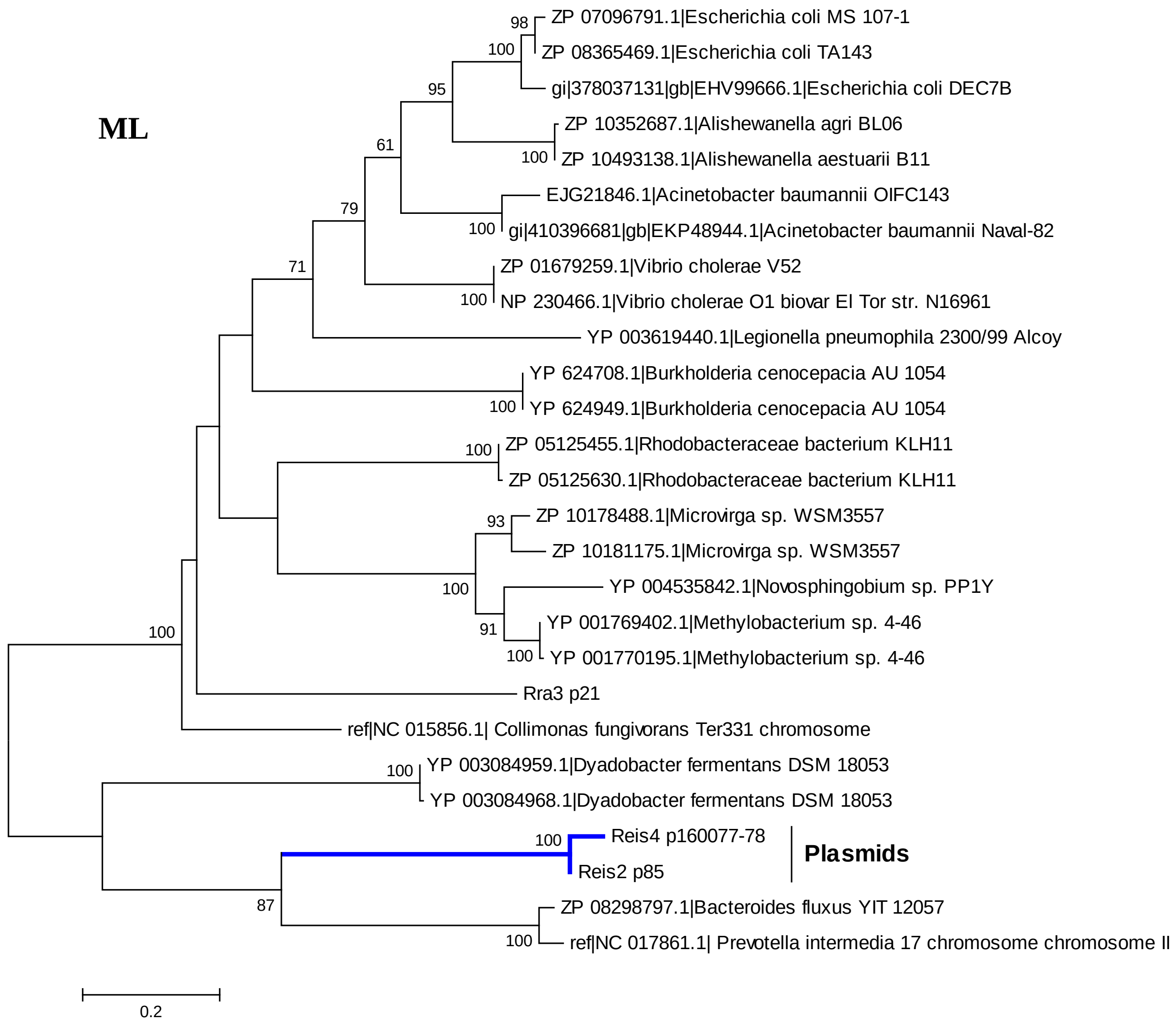
ML
ZP 07096791.1|Escherichia coli MS 107-1
ZP 08365469.1|Escherichia coli TA143
gi|378037131|gb|EHV99666.1|Escherichia coli DEC7B
ZP 10352687.1|Alishewanella agri BL06
ZP 10493138.1|Alishewanella aestuarii B11
EJG21846.1|Acinetobacter baumannii OIFC143
gi|410396681|gb|EKP48944.1|Acinetobacter baumannii Naval-82
ZP 01679259.1|Vibrio cholerae V52
NP 230466.1|Vibrio cholerae O1 biovar El Tor str. N16961
YP 003619440.1|Legionella pneumophila 2300/99 Alcoy
YP 624708.1|Burkholderia cenocepacia AU 1054
YP 624949.1|Burkholderia cenocepacia AU 1054
ZP 05125455.1|Rhodobacteraceae bacterium KLH11
ZP 05125630.1|Rhodobacteraceae bacterium KLH11
ZP 10178488.1|Microvirga sp. WSM3557
ZP 10181175.1|Microvirga sp. WSM3557
YP 004535842.1|Novosphingobium sp. PP1Y
YP 001769402.1|Methylobacterium sp. 4-46
YP 001770195.1|Methylobacterium sp. 4-46
Rra3 p21
ref|NC 015856.1| Collimonas fungivorans Ter331 chromosome
YP 003084959.1|Dyadobacter fermentans DSM 18053
YP 003084968.1|Dyadobacter fermentans DSM 18053
Reis4 p160077-78
Reis2 p85
Plasmids
ZP 08298797.1|Bacteroides fluxus YIT 12057
ref|NC 017861.1| Prevotella intermedia 17 chromosome chromosome II
98
100
95
61
79
71
100
100
100
100
100
93
100
91
100
100
100
100
87
100
0.2

Figure A106. Neighbor-joining (NJ) and maximum likelihood (ML) trees of transposase. Bootstrap supports higher than or equal to 60% are shown on the branches.

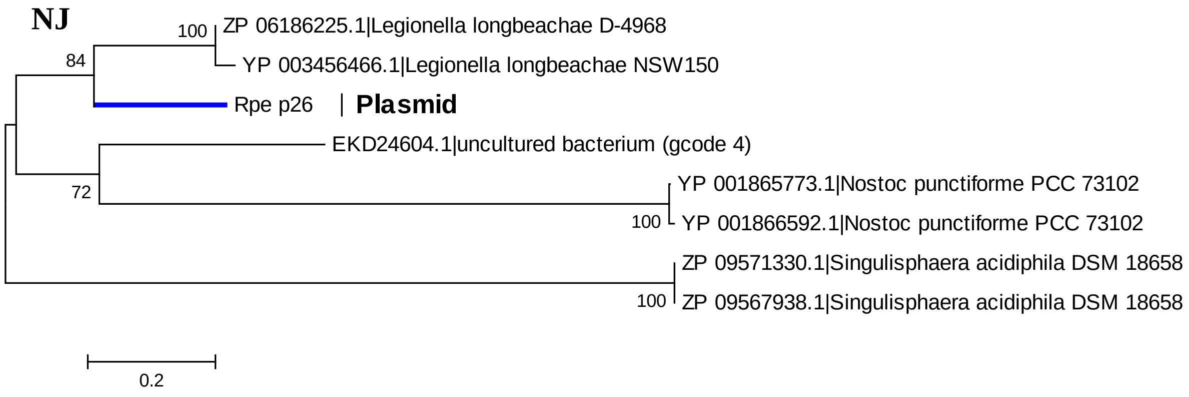

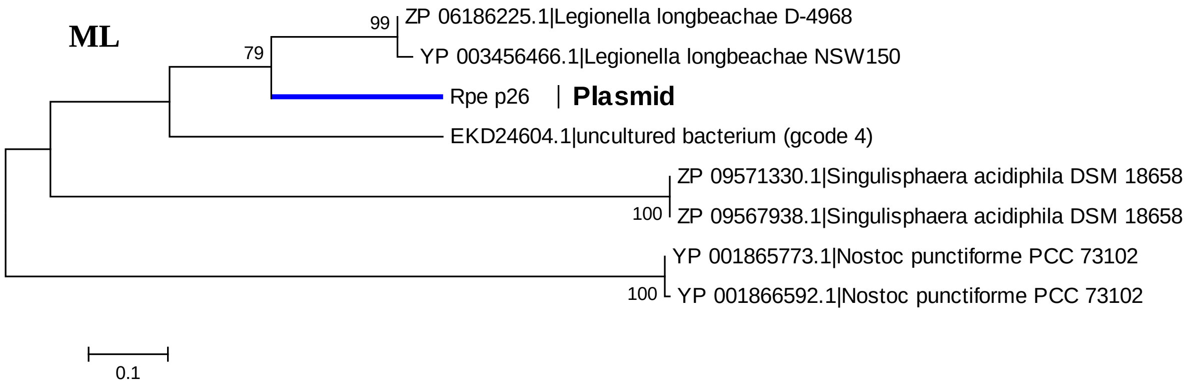

Figure A107. Neighbor-joining (NJ) and maximum likelihood (ML) trees of RNA-directed DNA polymarases. Bootstrap supports higher than or equal to 60% are shown on the branches.

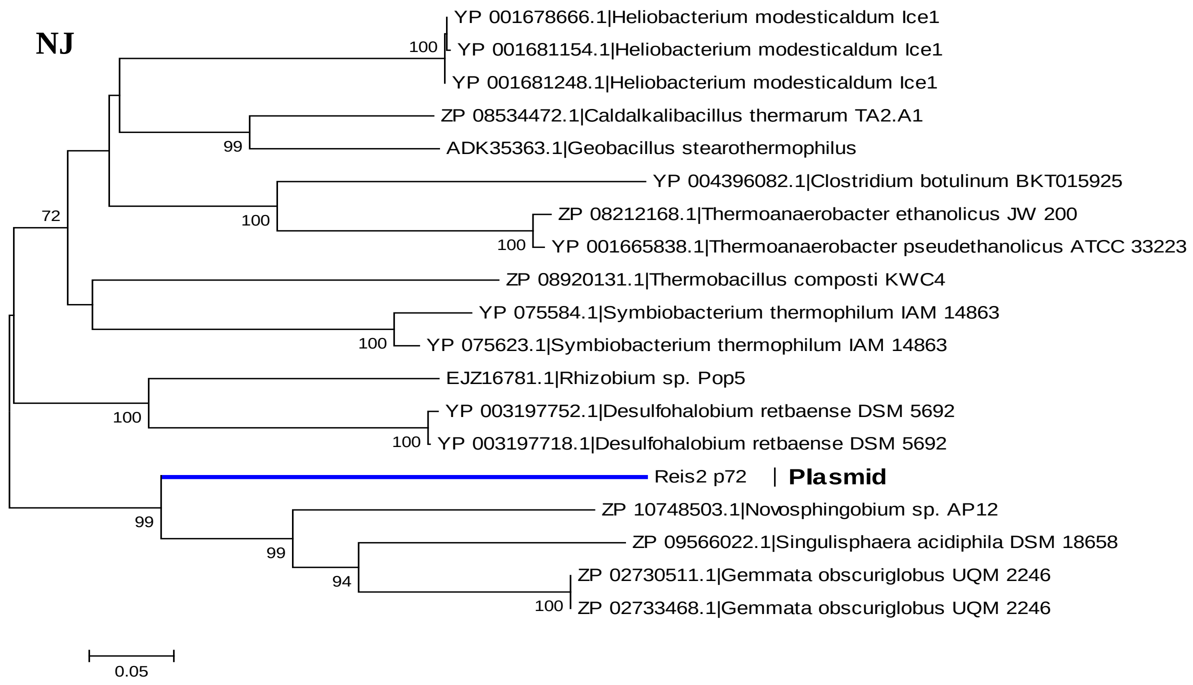

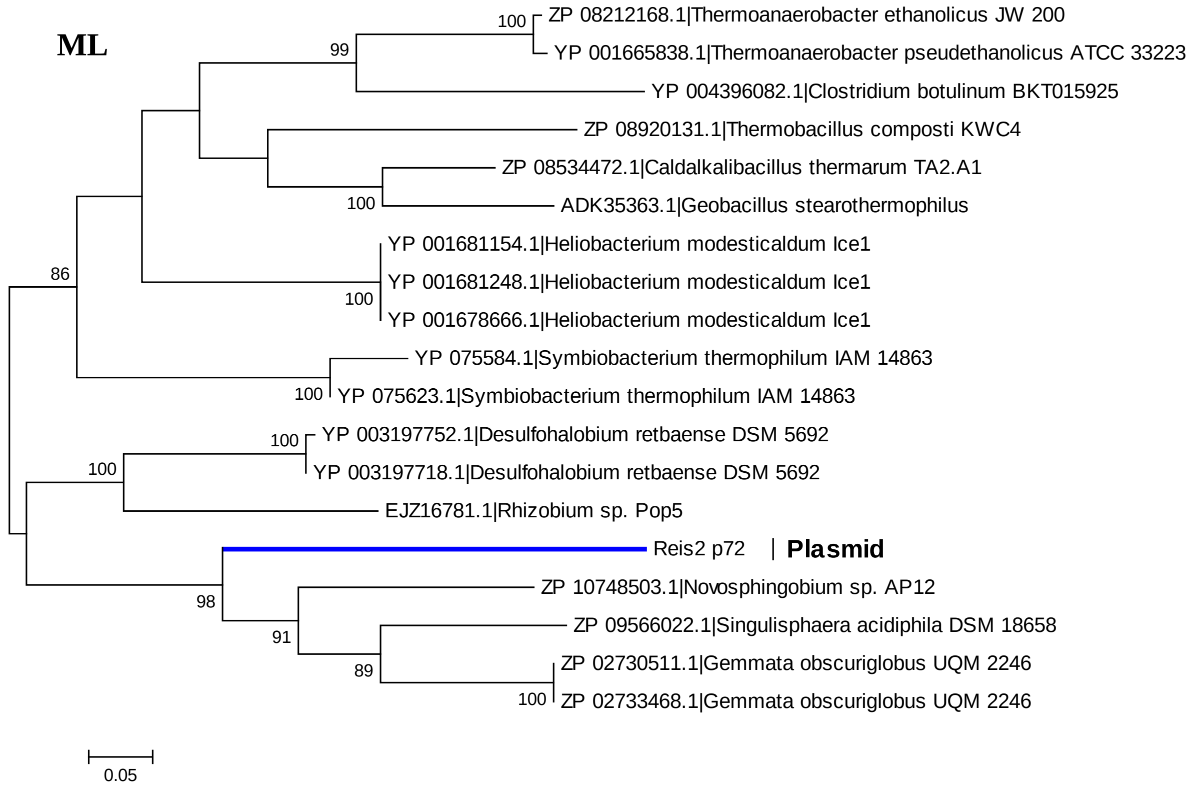

Figure A108. Neighbor-joining (NJ) and maximum likelihood (ML) trees of mobile mystery protein B. Bootstrap supports higher than or equal to 60% are shown on the branches.

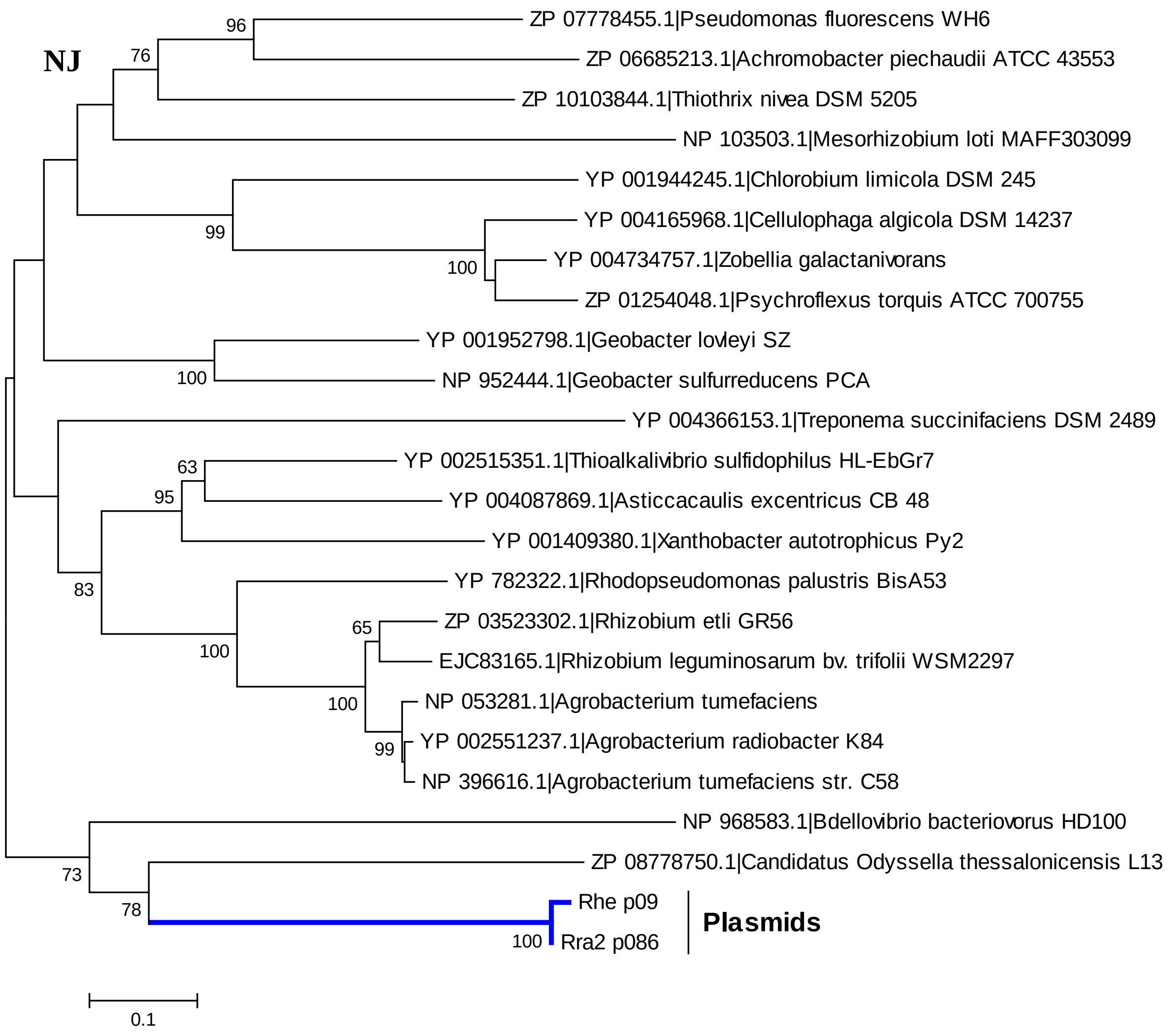

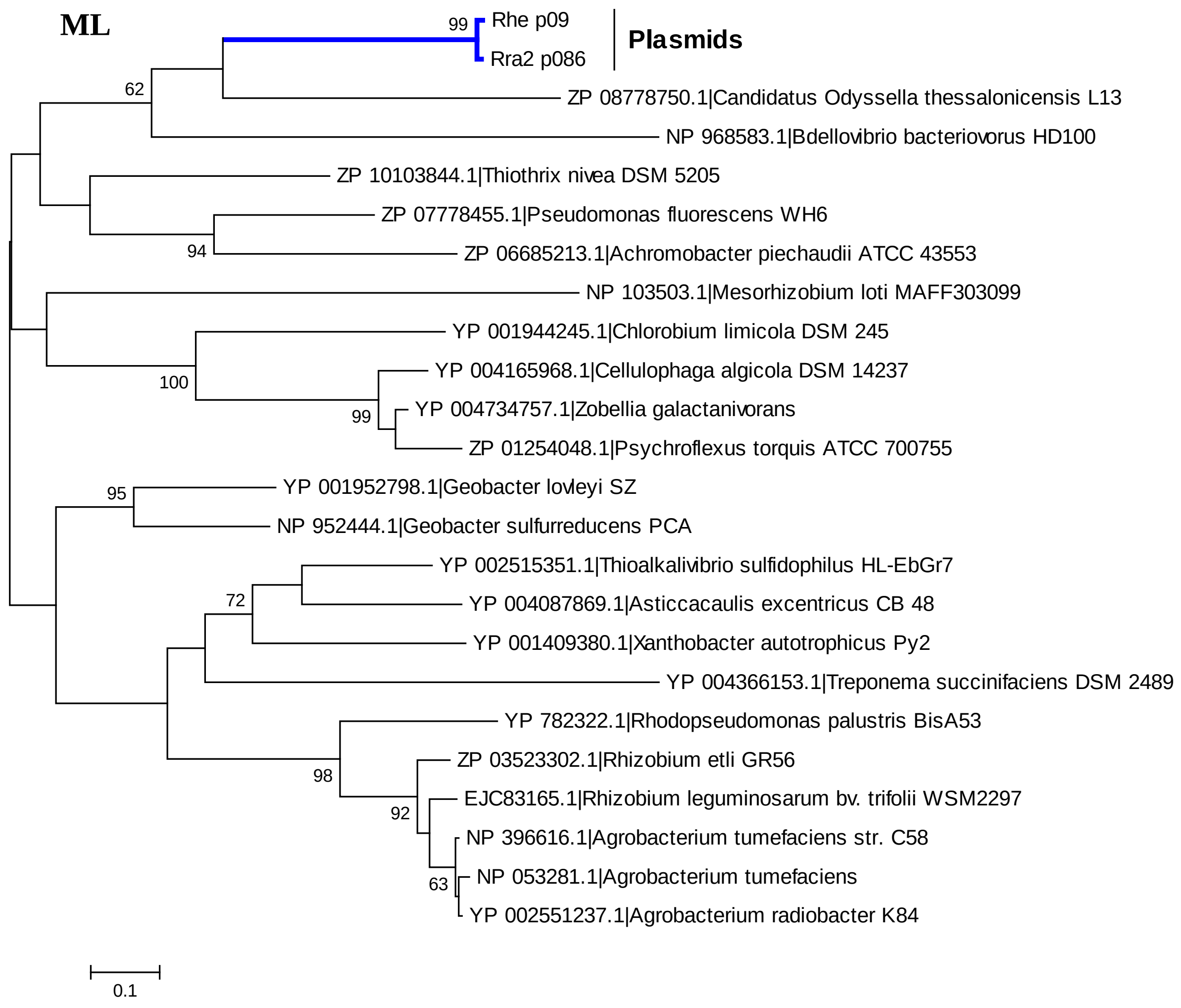
ML
Plasmids
Rhe p09
Rra2 p086
ZP 08778750.1|Candidatus Odyssella thessalonicensis L13
NP 968583.1|Bdellovibrio bacteriovorus HD100
ZP 10103844.1|Thiothrix nivea DSM 5205
ZP 07778455.1|Pseudomonas fluorescens WH6
ZP 06685213.1|Achromobacter piechaudii ATCC 43553
NP 103503.1|Mesorhizobium loti MAFF303099
YP 001944245.1|Chlorobium limicola DSM 245
YP 004165968.1|Cellulophaga algicola DSM 14237
YP 004734757.1|Zobellia galactanivorans
ZP 01254048.1|Psychroflexus torquis ATCC 700755
YP 001952798.1|Geobacter lovleyi SZ
NP 952444.1|Geobacter sulfurreducens PCA
YP 002515351.1|Thioalkalivibrio sulfidophilus HL-EbGr7
YP 004087869.1|Asticcacaulis excentricus CB 48
YP 001409380.1|Xanthobacter autotrophicus Py2
YP 004366153.1|Treponema succinifaciens DSM 2489
YP 782322.1|Rhodopseudomonas palustris BisA53
ZP 03523302.1|Rhizobium etli GR56
EJC83165.1|Rhizobium leguminosarum bv. trifolii WSM2297
NP 396616.1|Agrobacterium tumefaciens str. C58
NP 053281.1|Agrobacterium tumefaciens
YP 002551237.1|Agrobacterium radiobacter K84
99
62
94
100
99
95
72
98
92
63
0.1

Figure A109. Neighbor-joining (NJ) and maximum likelihood (ML) trees of conserved protein of unknown function. Bootstrap supports higher than or equal to 60% are shown on the branches.

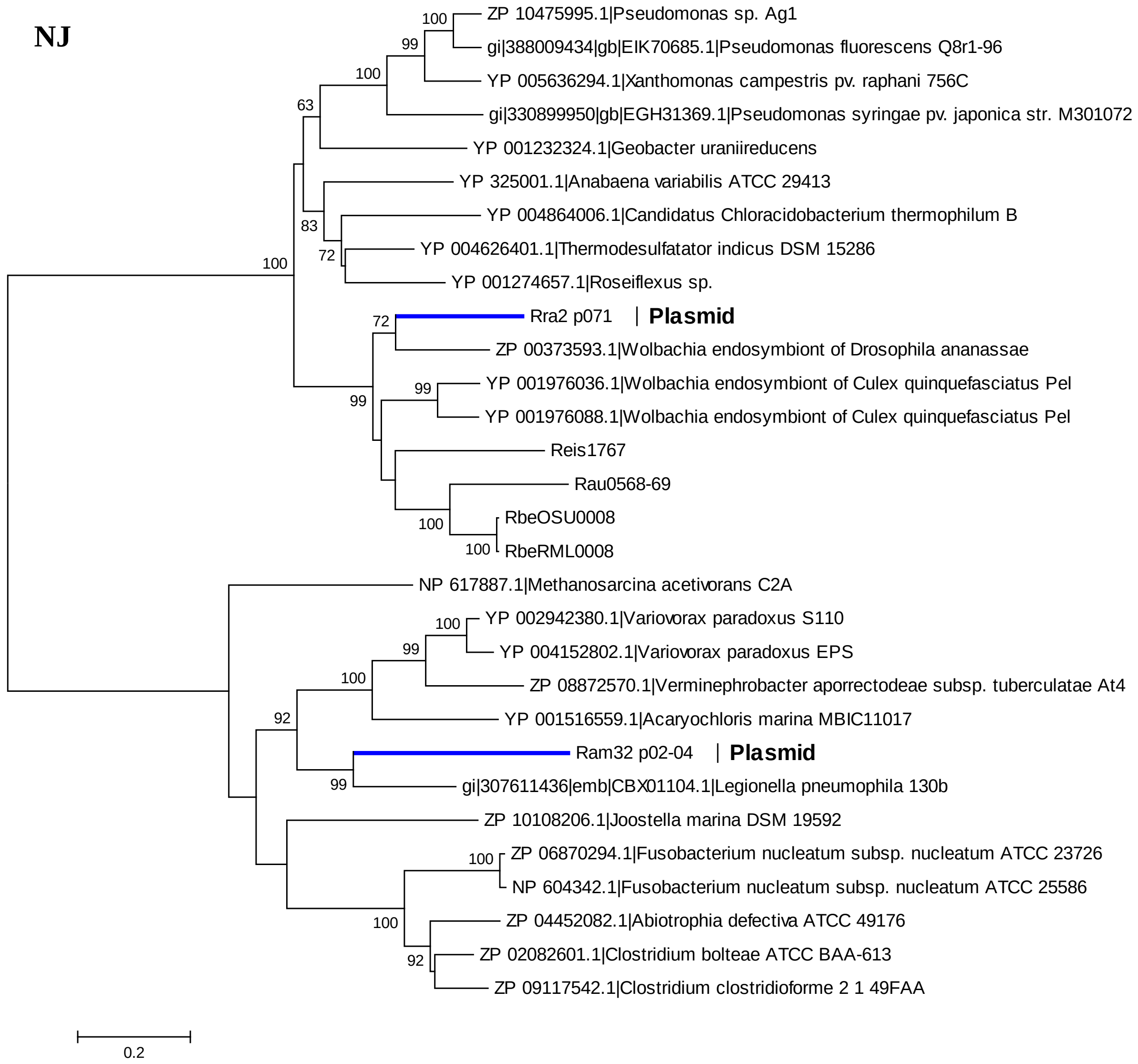

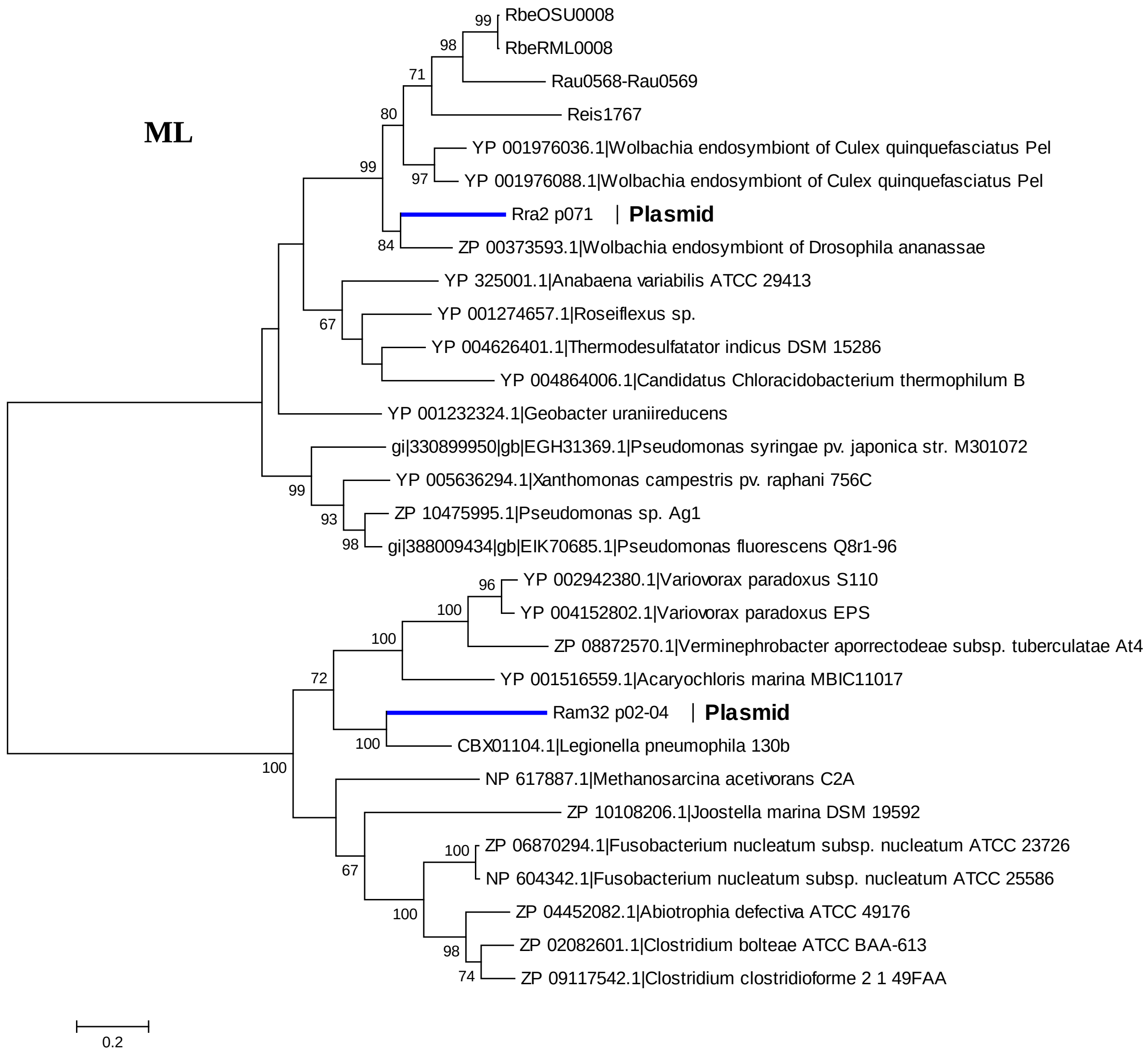
ML
RbeOSU0008
RbeRML0008
Rau0568-Rau0569
Reis1767
YP 001976036.1|Wolbachia endosymbiont of Culex quinquefasciatus Pel
YP 001976088.1|Wolbachia endosymbiont of Culex quinquefasciatus Pel
Rra2 p071
Plasmid
ZP 00373593.1|Wolbachia endosymbiont of Drosophila ananassae
YP 325001.1|Anabaena variabilis ATCC 29413
YP 001274657.1|Roseiflexus sp.
YP 004626401.1|Thermodesulfatator indicus DSM 15286
YP 004864006.1|Candidatus Chloracidobacterium thermophilum B
YP 001232324.1|Geobacter uraniireducens
gi|330899950|gb|EGH31369.1|Pseudomonas syringae pv. japonica str. M301072
YP 005636294.1|Xanthomonas campestris pv. raphani 756C
ZP 10475995.1|Pseudomonas sp. Ag1
gi|388009434|gb|EIK70685.1|Pseudomonas fluorescens Q8r1-96
YP 002942380.1|Variovorax paradoxus S110
YP 004152802.1|Variovorax paradoxus EPS
ZP 08872570.1|Verminephrobacter aporrectodeae subsp. tuberculatae At4
YP 001516559.1|Acaryochloris marina MBIC11017
Ram32 p02-04
Plasmid
CBX01104.1|Legionella pneumophila 130b
NP 617887.1|Methanosarcina acetivorans C2A
ZP 10108206.1|Joostella marina DSM 19592
ZP 06870294.1|Fusobacterium nucleatum subsp. nucleatum ATCC 23726
NP 604342.1|Fusobacterium nucleatum subsp. nucleatum ATCC 25586
ZP 04452082.1|Abiotrophia defectiva ATCC 49176
ZP 02082601.1|Clostridium bolteae ATCC BAA-613
ZP 09117542.1|Clostridium clostridioforme 2 1 49FAA
99
98
71
80
99
97
84
67
99
93
98
96
100
100
72
100
100
100
67
100
98
74
0.2

Figure A110. Neighbor-joining (NJ) and maximum likelihood (ML) trees of conserved protein of unknown function. Bootstrap supports higher than or equal to 60% are shown on the branches.

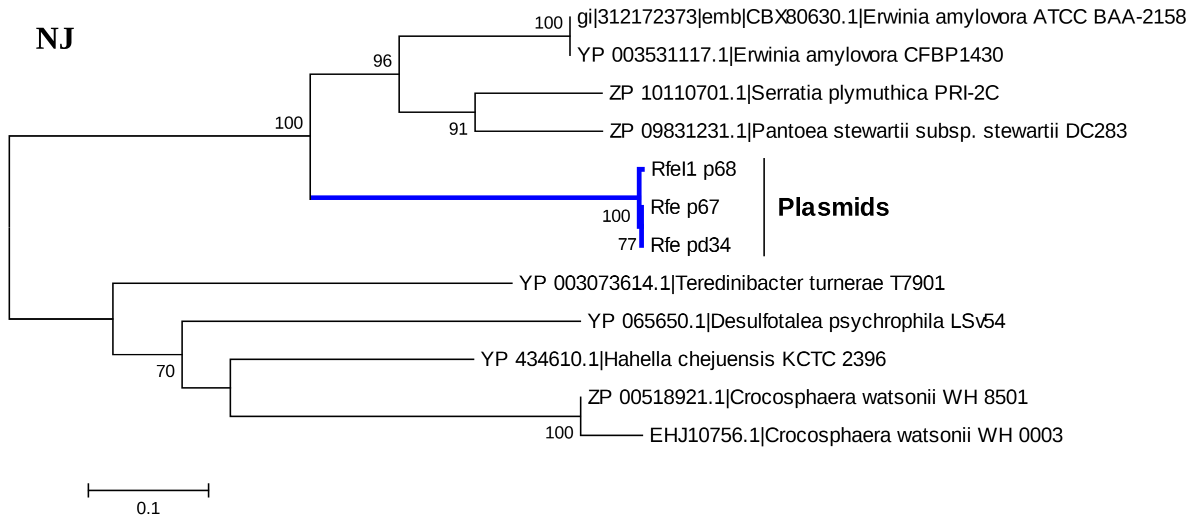

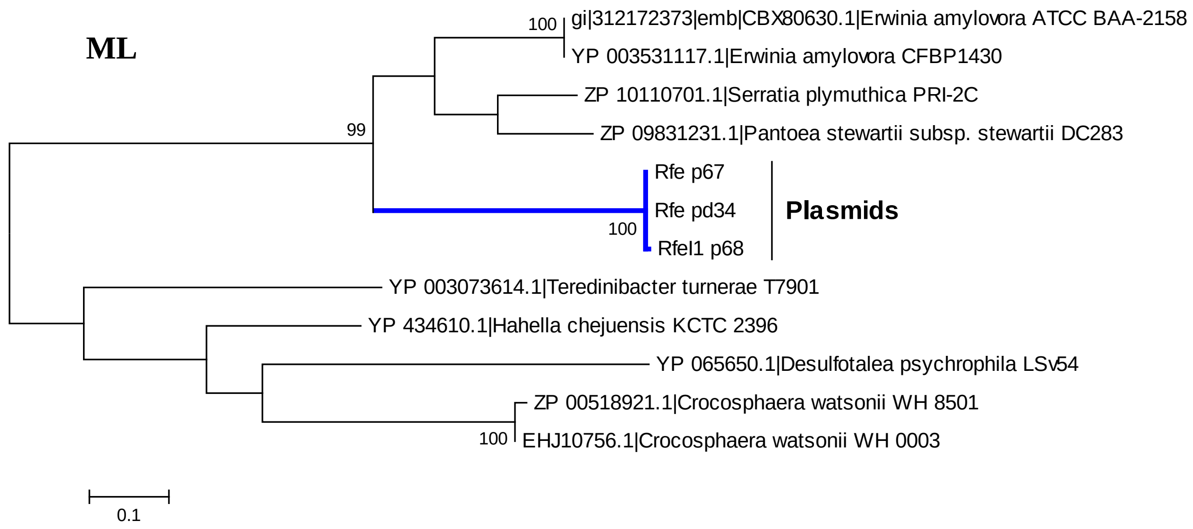

Figure A111. Neighbor-joining (NJ) and maximum likelihood (ML) trees of conserved protein of unknown function. Bootstrap supports higher than or equal to 60% are shown on the branches.

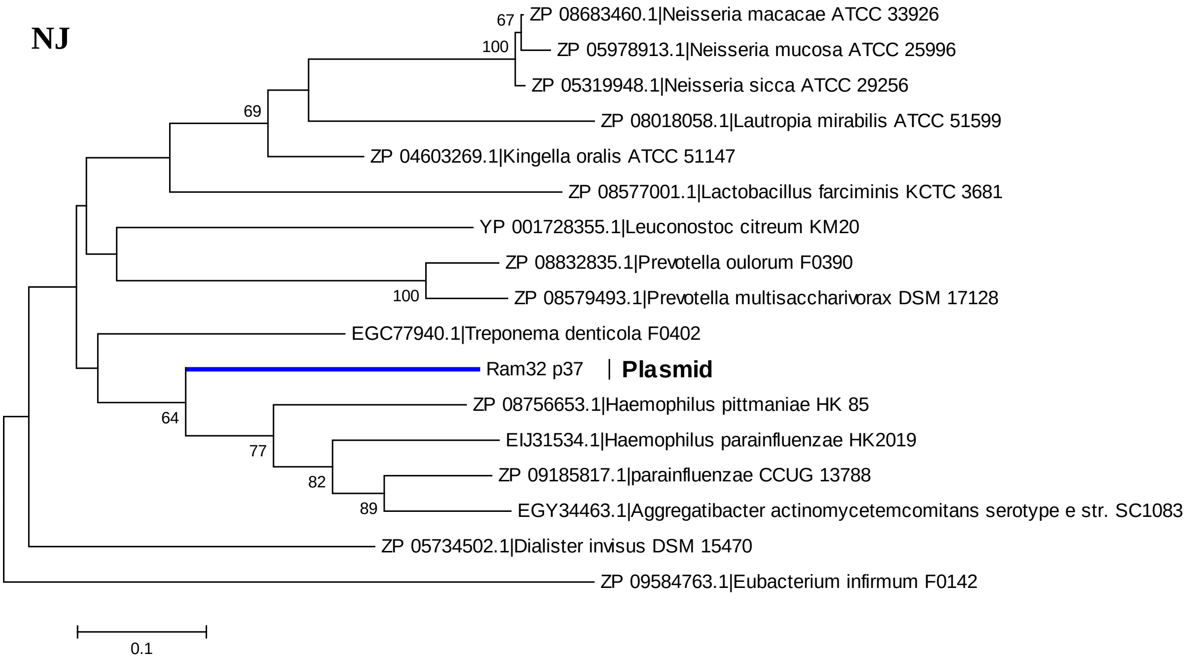

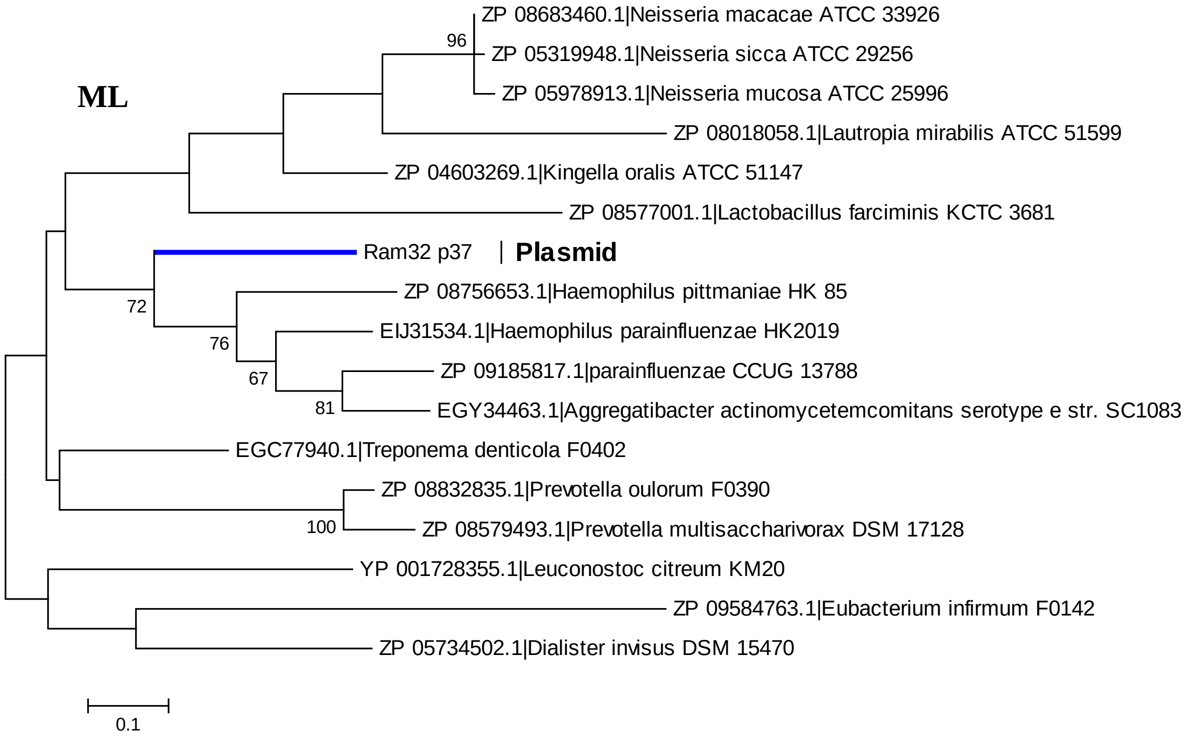

Figure A112. Neighbor-joining (NJ) and maximum likelihood (ML) trees of transposase/integrase containing HTH_21 and rve domains. Bootstrap supports higher than or equal to 60% are shown on the branches.

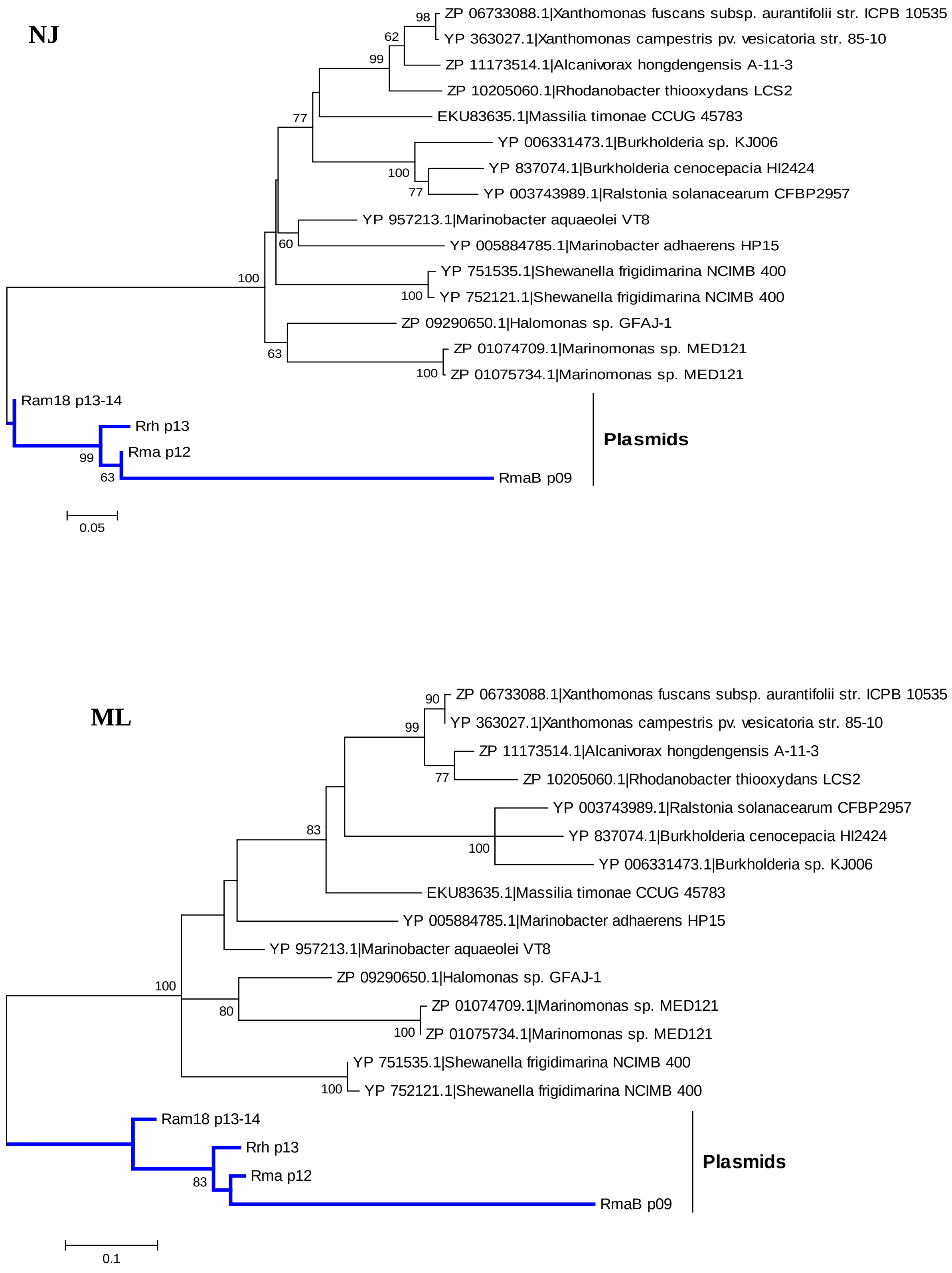

Figure A113. Neighbor-joining (NJ) and maximum likelihood (ML) trees of cytosine-specific methyltransferase. Bootstrap supports higher than or equal to 60% are shown on the branches.

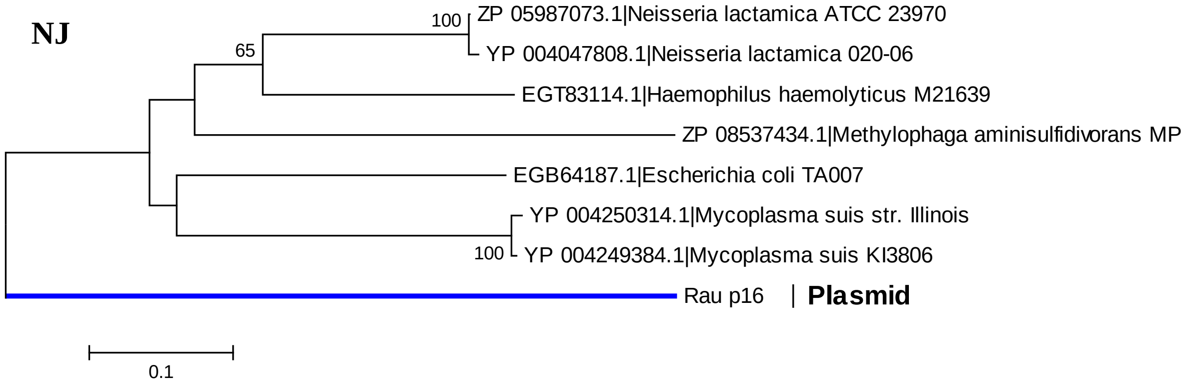

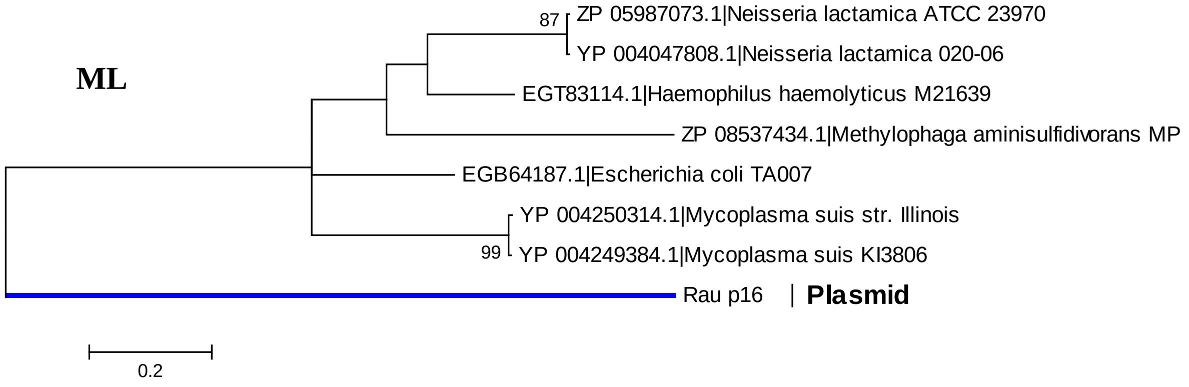

Figure A114. Neighbor-joining (NJ) and maximum likelihood (ML) trees of conserved protein of unknown function. Bootstrap supports higher than or equal to 60% are shown on the branches.

NJ

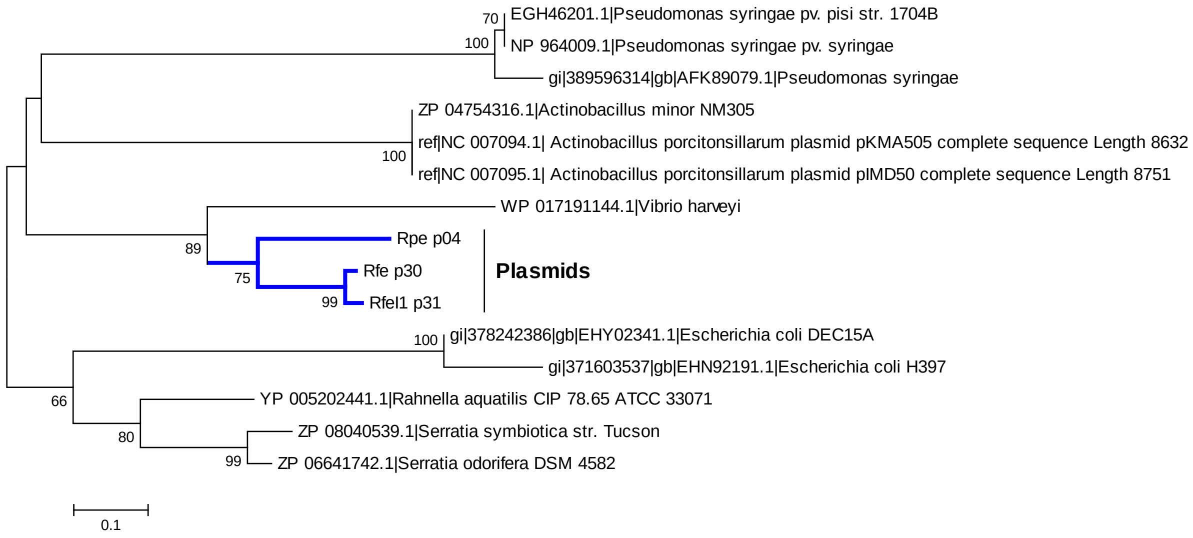

ML

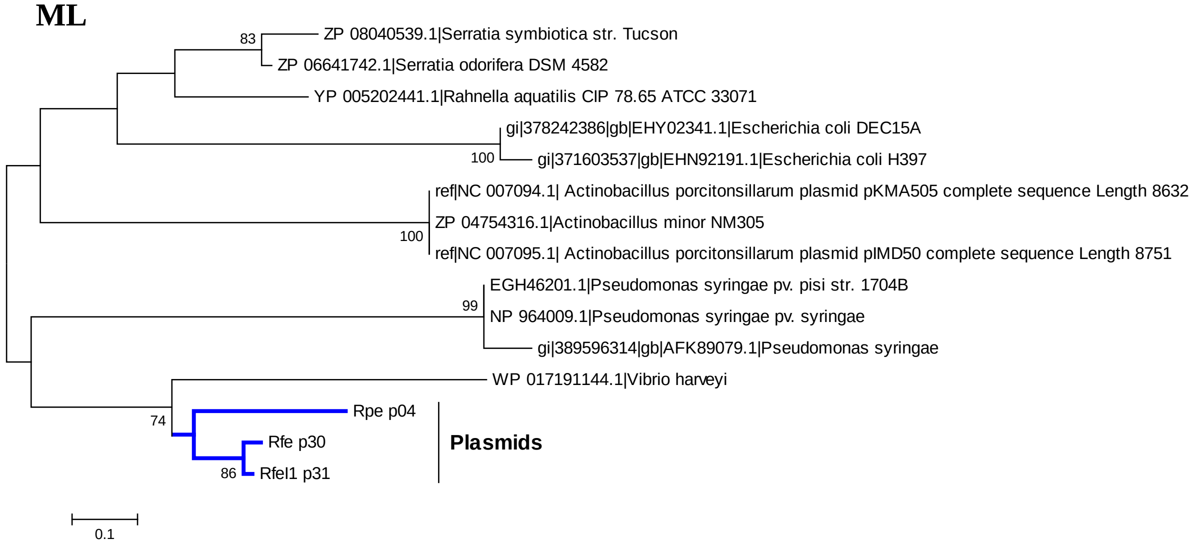

Figure A115. Neighbor-joining (NJ) and maximum likelihood (ML) trees of conserved protein of unknown function. Bootstrap supports higher than or equal to 60% are shown on the branches.

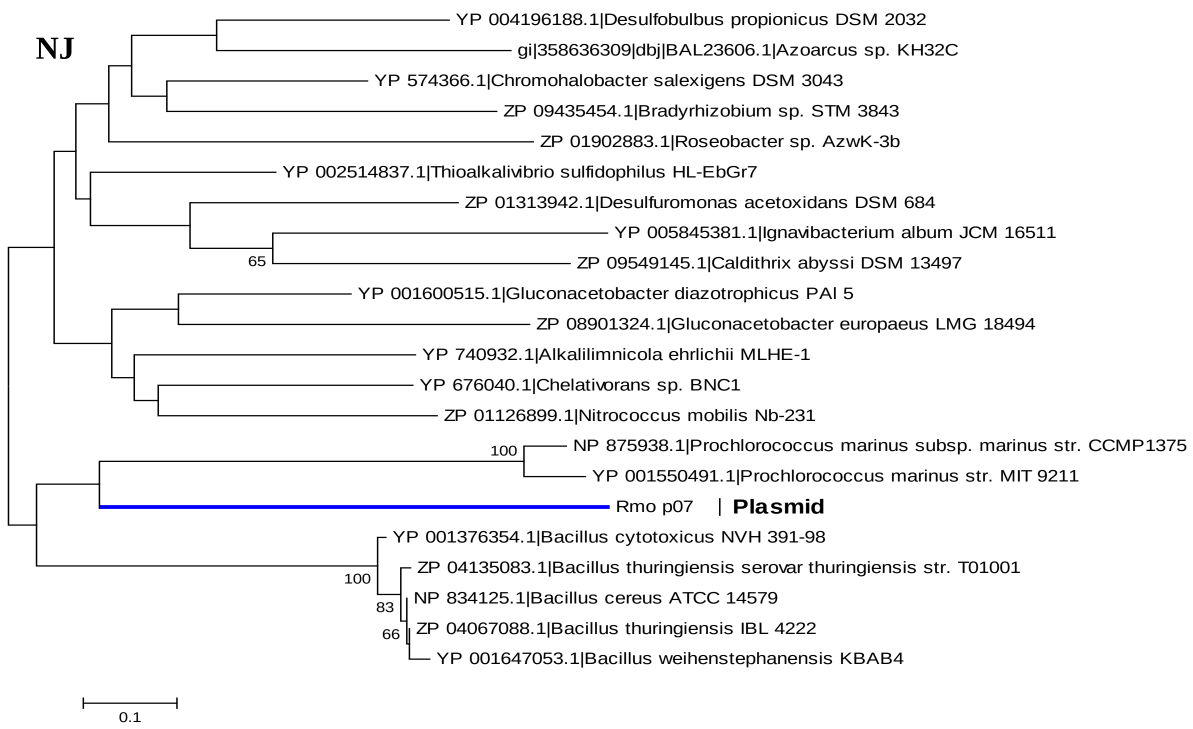

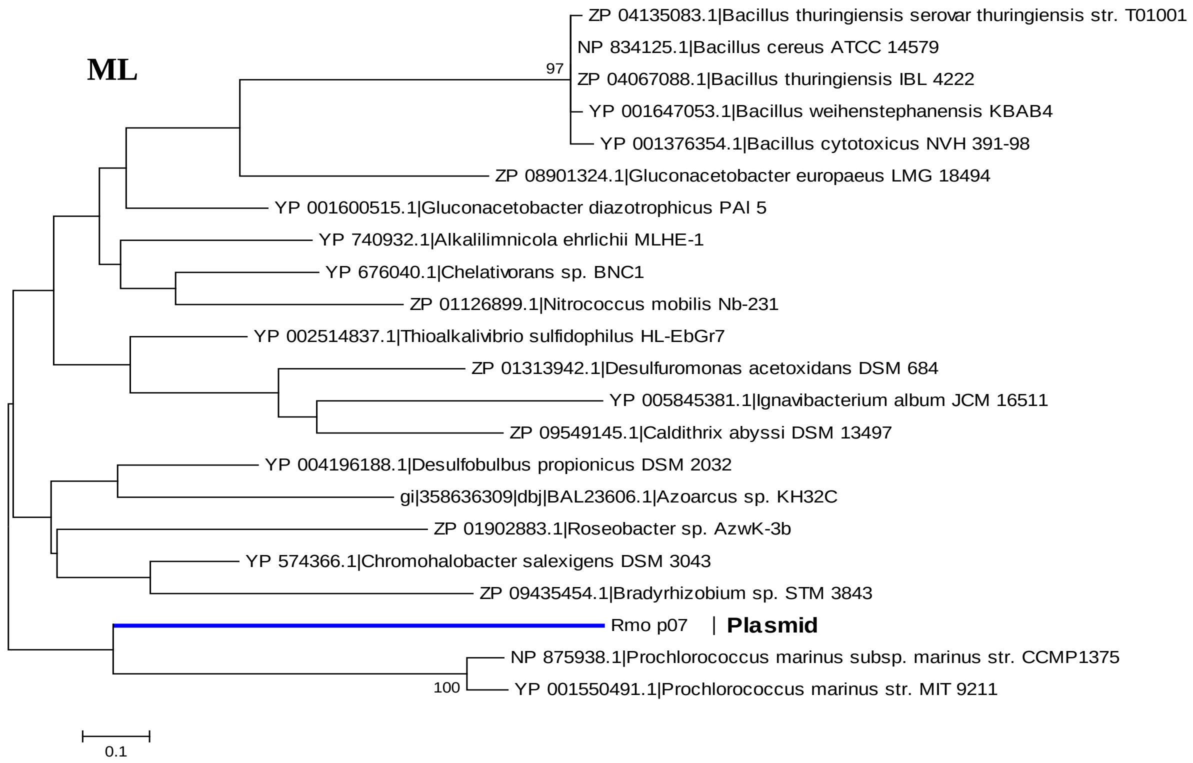

Figure A116. Neighbor-joining (NJ) and maximum likelihood (ML) trees of putative lytic transglycosylase (LT) domain protein. Bootstrap supports higher than or equal to 60% are shown on the branches.

NJ

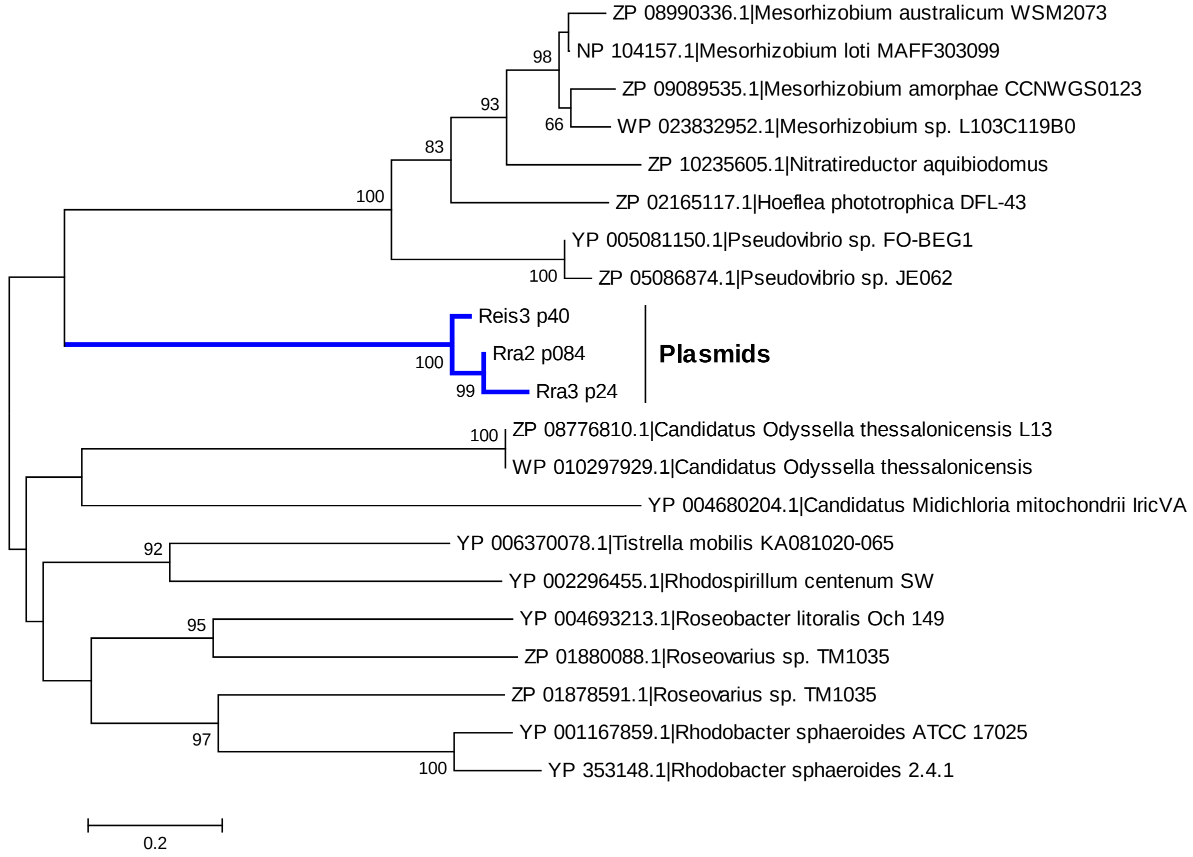

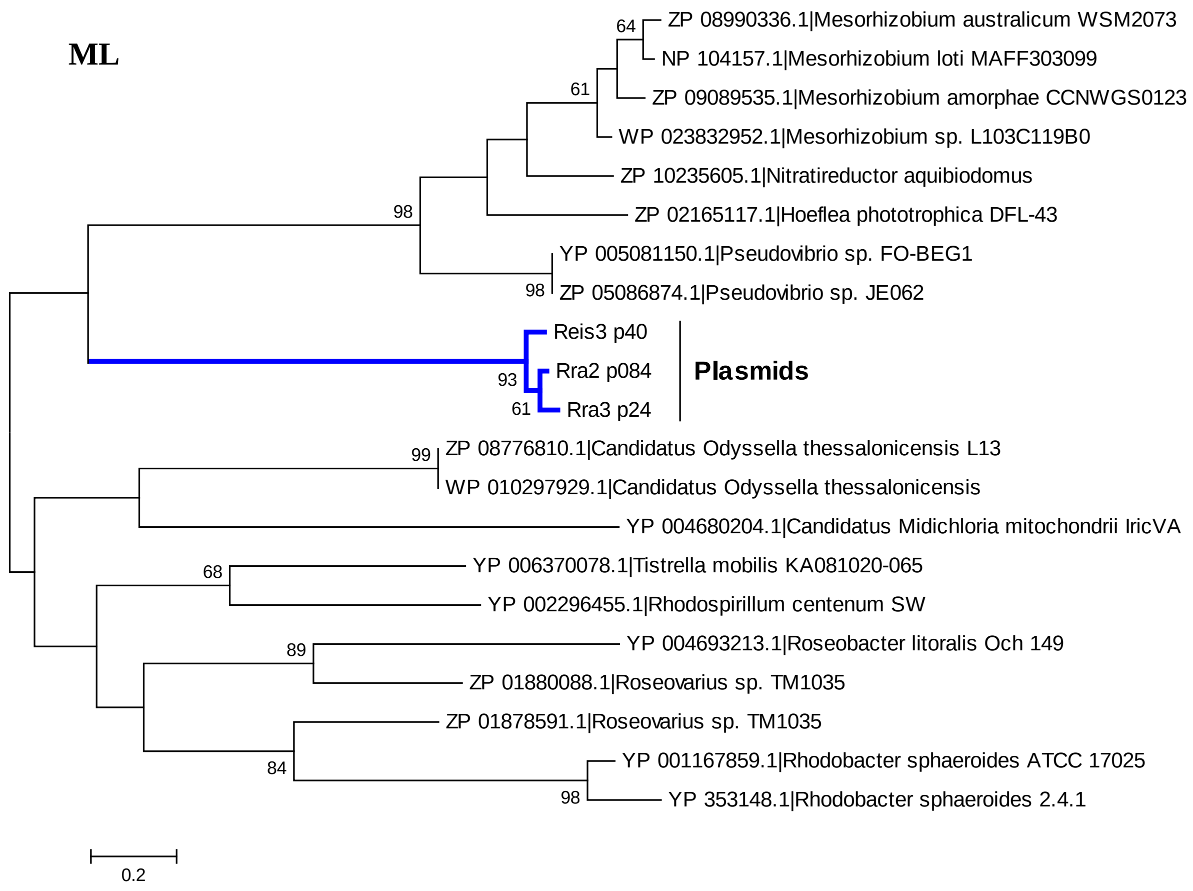
ML
ZP 08990336.1|Mesorhizobium australicum WSM2073
NP 104157.1|Mesorhizobium loti MAFF303099
ZP 09089535.1|Mesorhizobium amorphae CCNWGS0123
WP 023832952.1|Mesorhizobium sp. L103C119B0
ZP 10235605.1|Nitratireductor aquibiodomus
ZP 02165117.1|Hoeflea phototrophica DFL-43
YP 005081150.1|Pseudovibrio sp. FO-BEG1
ZP 05086874.1|Pseudovibrio sp. JE062
Reis3 p40
Rra2 p084
Rra3 p24
Plasmids
ZP 08776810.1|Candidatus Odyssella thessalonicensis L13
WP 010297929.1|Candidatus Odyssella thessalonicensis
YP 004680204.1|Candidatus Midichloria mitochondrii IricVA
YP 006370078.1|Tistrella mobilis KA081020-065
YP 002296455.1|Rhodospirillum centenum SW
YP 004693213.1|Roseobacter litoralis Och 149
ZP 01880088.1|Roseovarius sp. TM1035
ZP 01878591.1|Roseovarius sp. TM1035
YP 001167859.1|Rhodobacter sphaeroides ATCC 17025
YP 353148.1|Rhodobacter sphaeroides 2.4.1
64
61
98
98
93
61
99
68
89
84
98
0.2

Figure A117. Neighbor-joining (NJ) and maximum likelihood (ML) trees of putative DNA-binding protein. Bootstrap supports higher than or equal to 60% are shown on the branches.

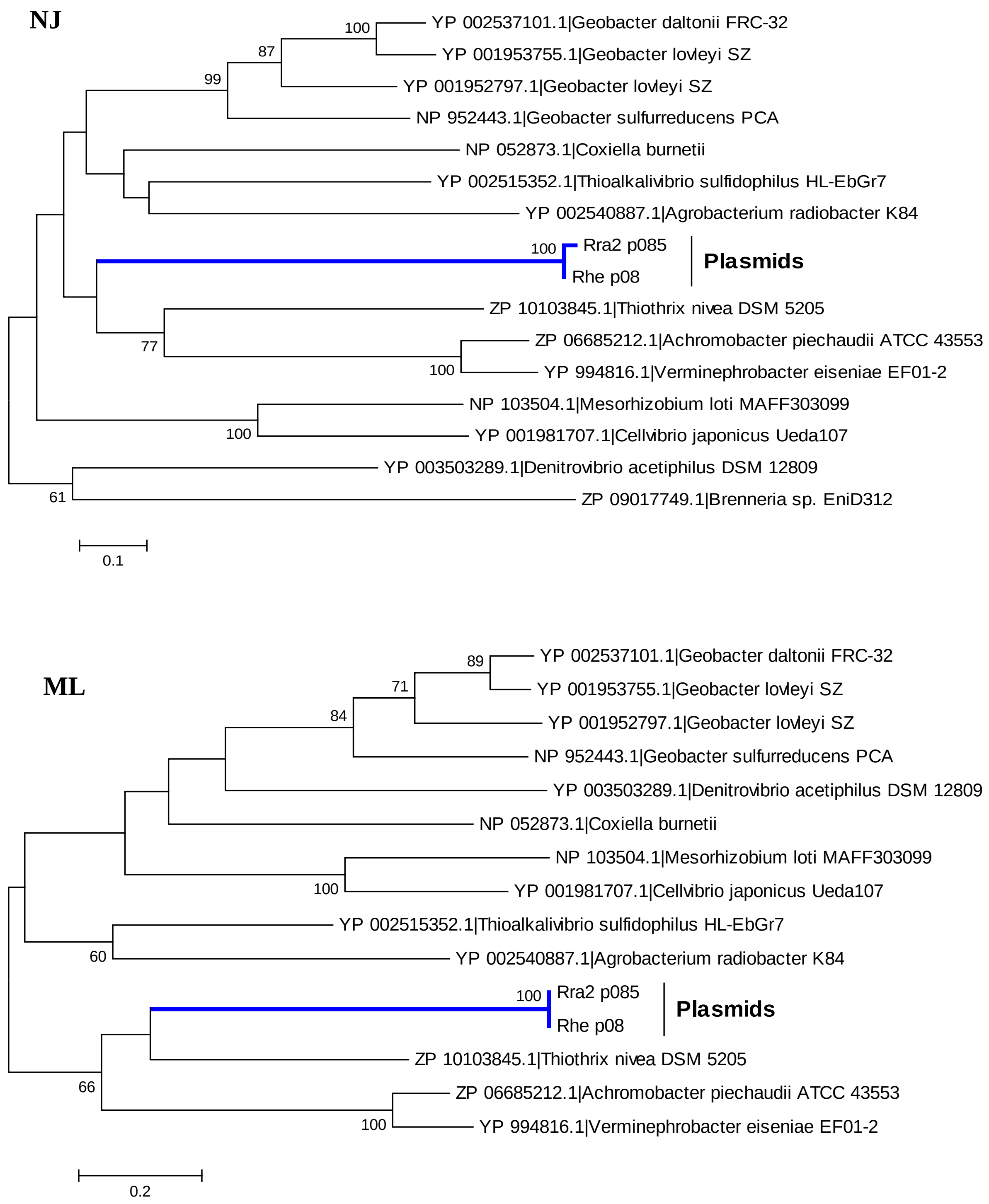

Figure A118. Neighbor-joining (NJ) and maximum likelihood (ML) trees of ParA-like family protein. Bootstrap supports higher than or equal to 60% are shown on the branches.

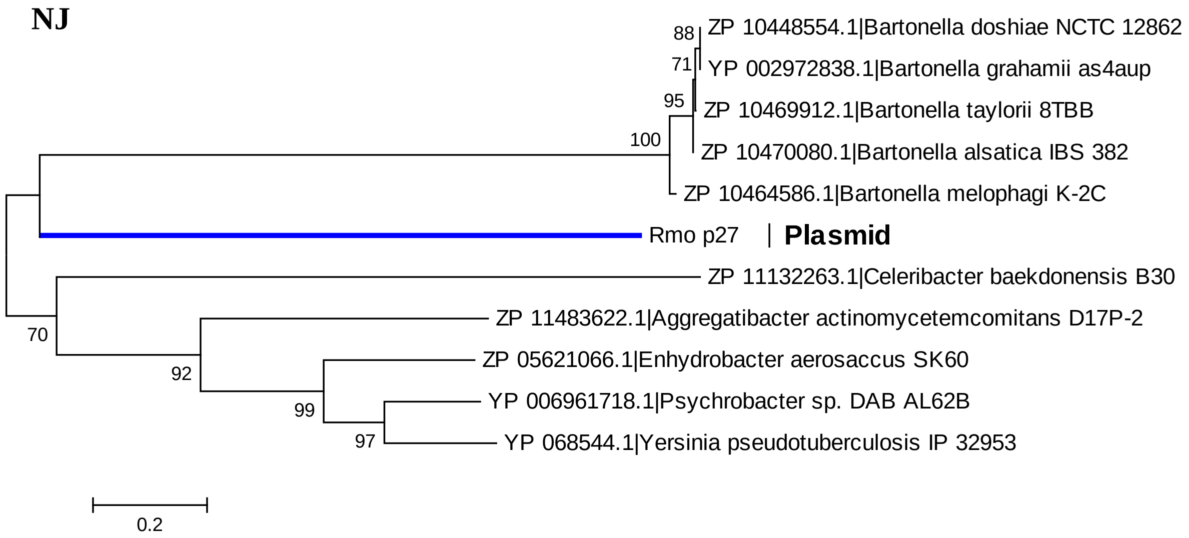

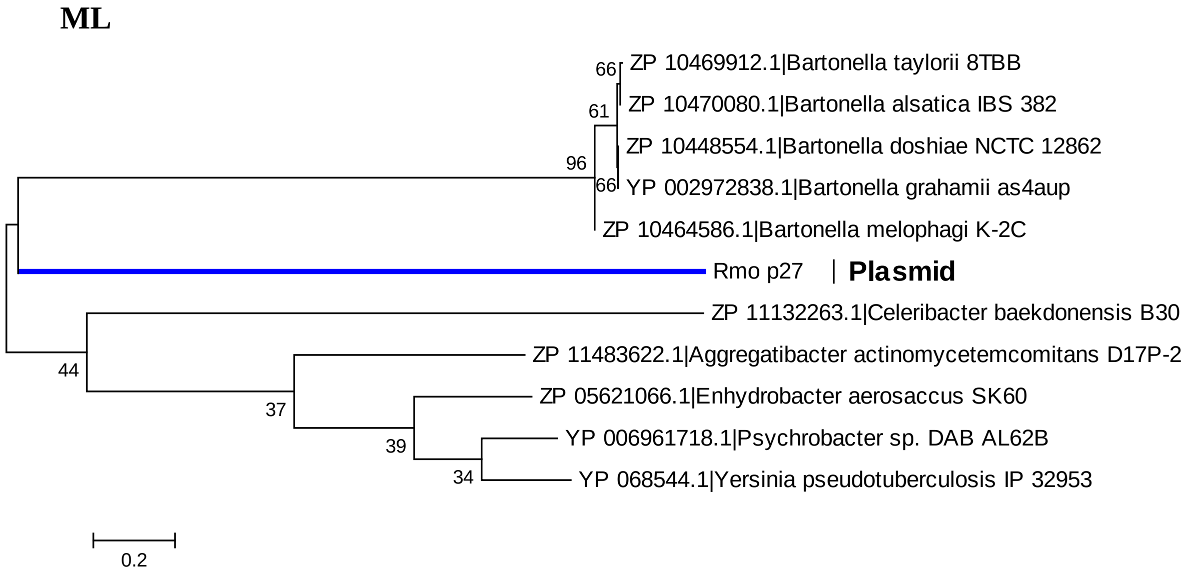

Figure A119. Neighbor-joining (NJ) and maximum likelihood (ML) trees of type I restriction-modification system methyltransferase subunit. Bootstrap supports higher than or equal to 60% are shown on the branches.

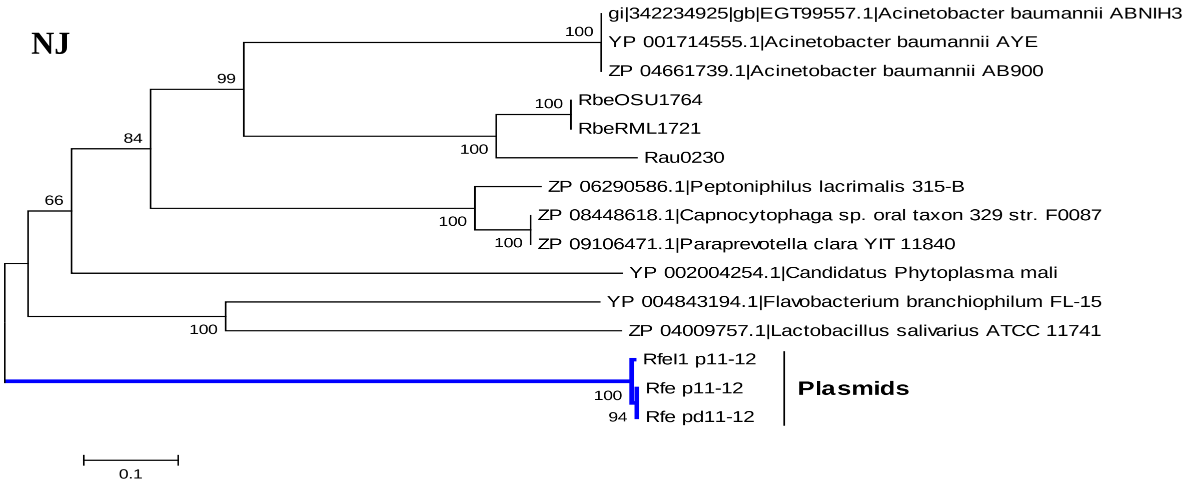

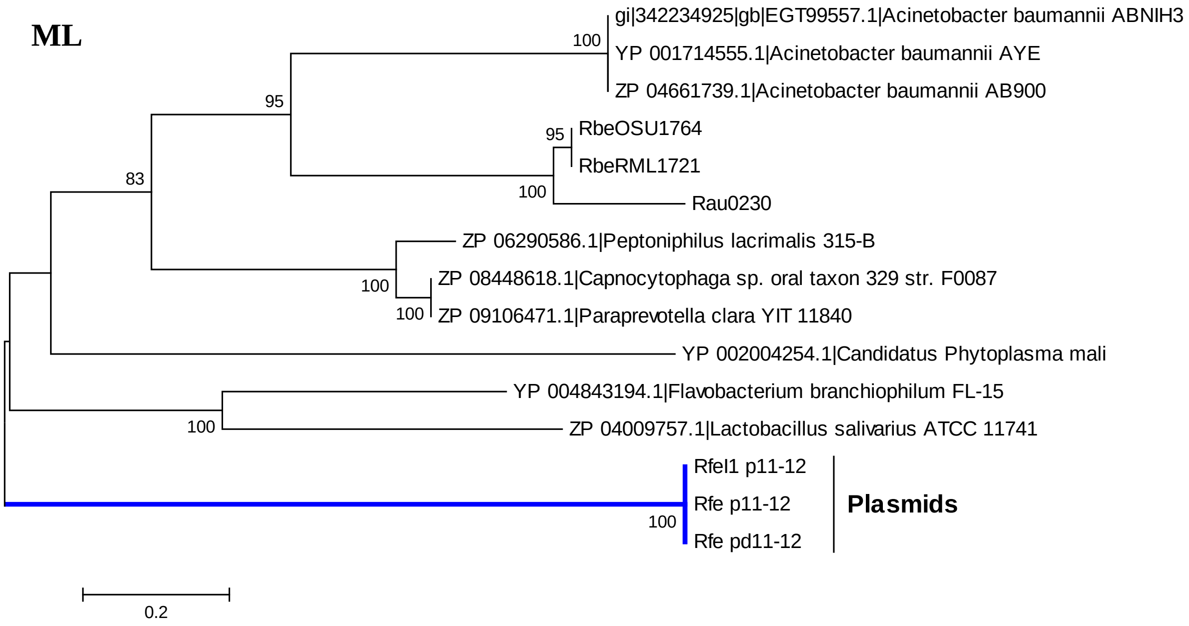

Figure A120. Neighbor-joining (NJ) and maximum likelihood (ML) trees of conserved protein of unknown function. Bootstrap supports higher than or equal to 60% are shown on the branches.

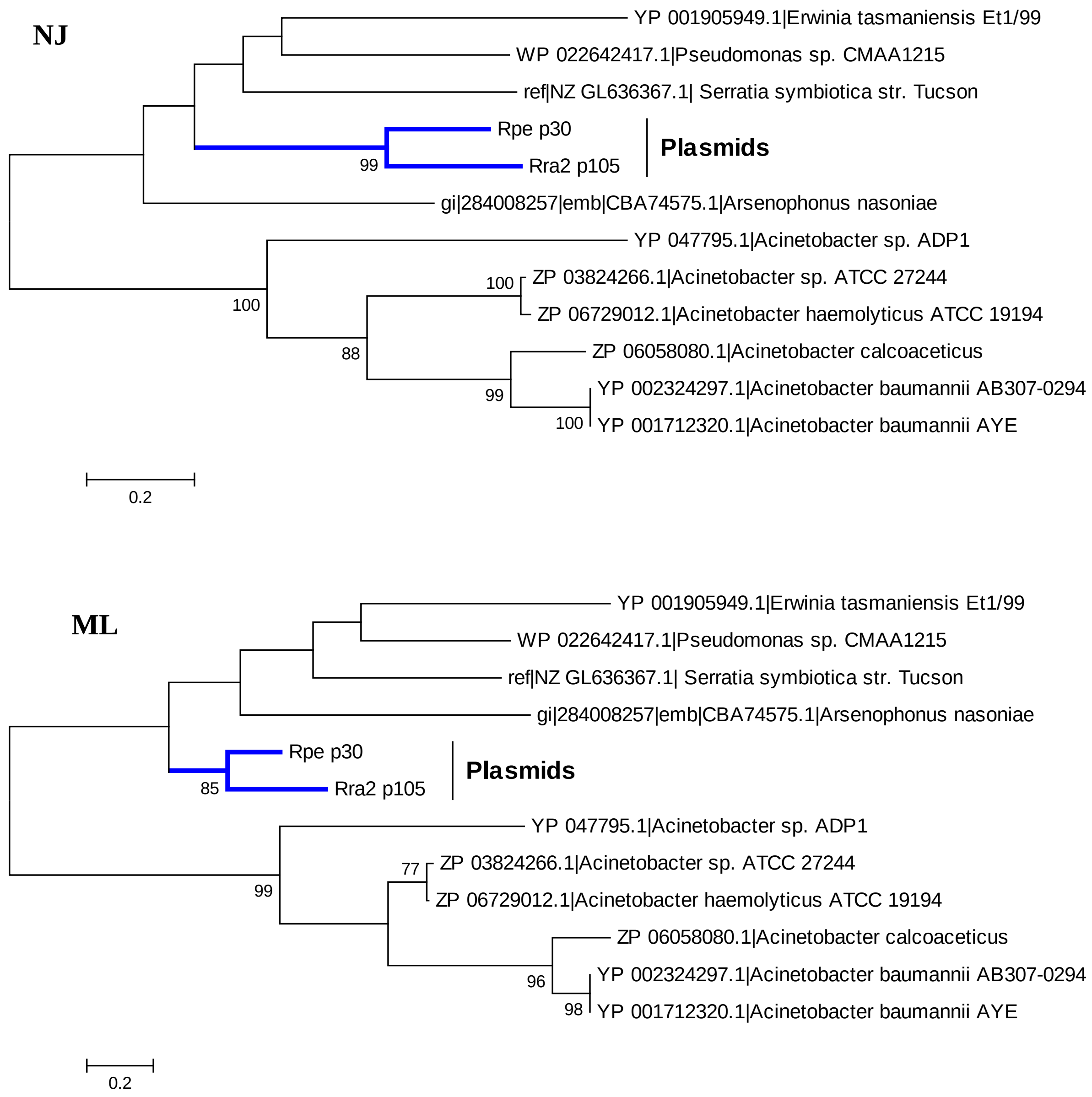

Figure A121. Neighbor-joining (NJ) and maximum likelihood (ML) trees of conserved protein of unknown function. Bootstrap supports higher than or equal to 60% are shown on the branches.

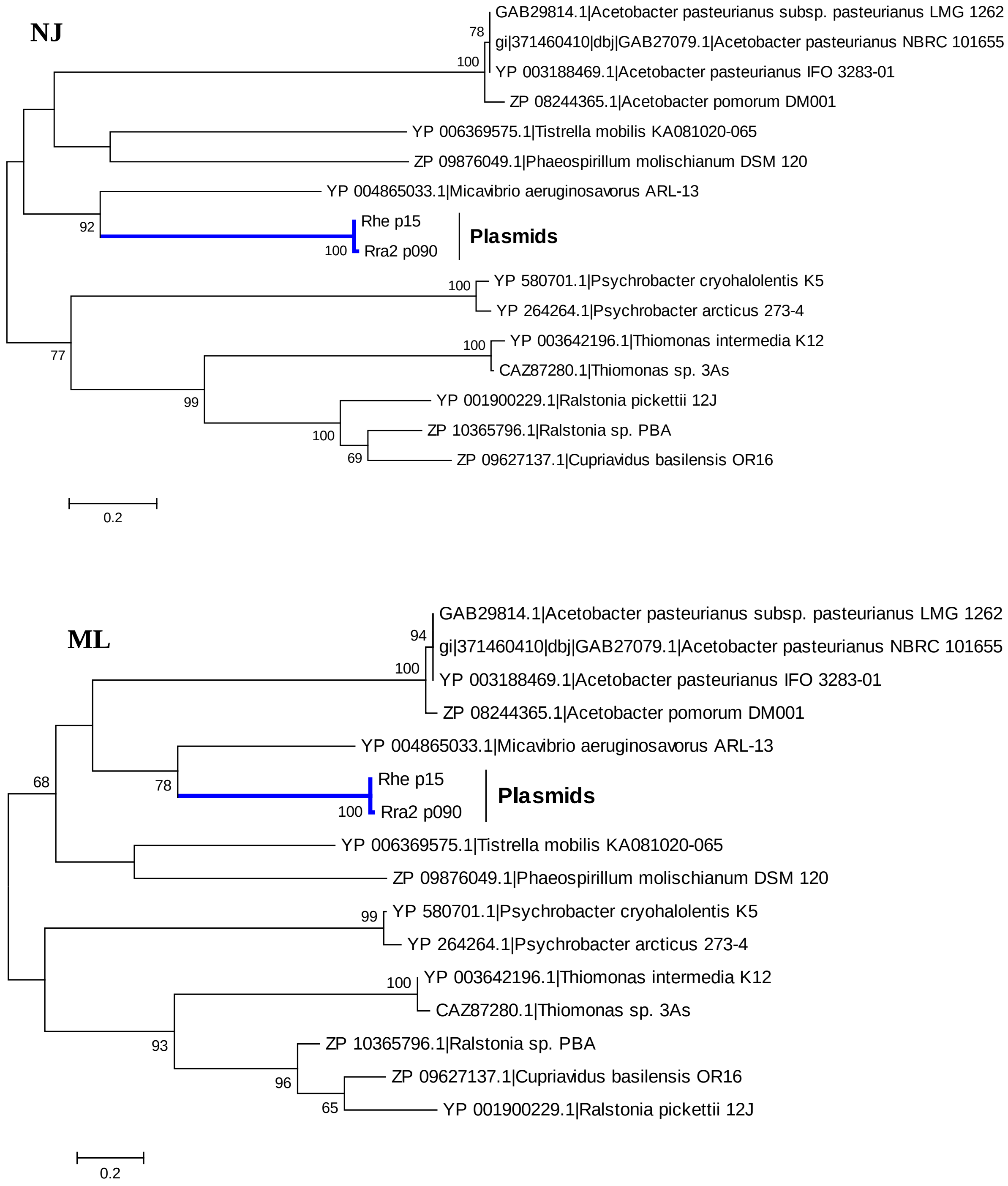

Figure A122. Neighbor-joining (NJ) and maximum likelihood (ML) trees of conserved protein of unknown function. Bootstrap supports higher than or equal to 60% are shown on the branches.

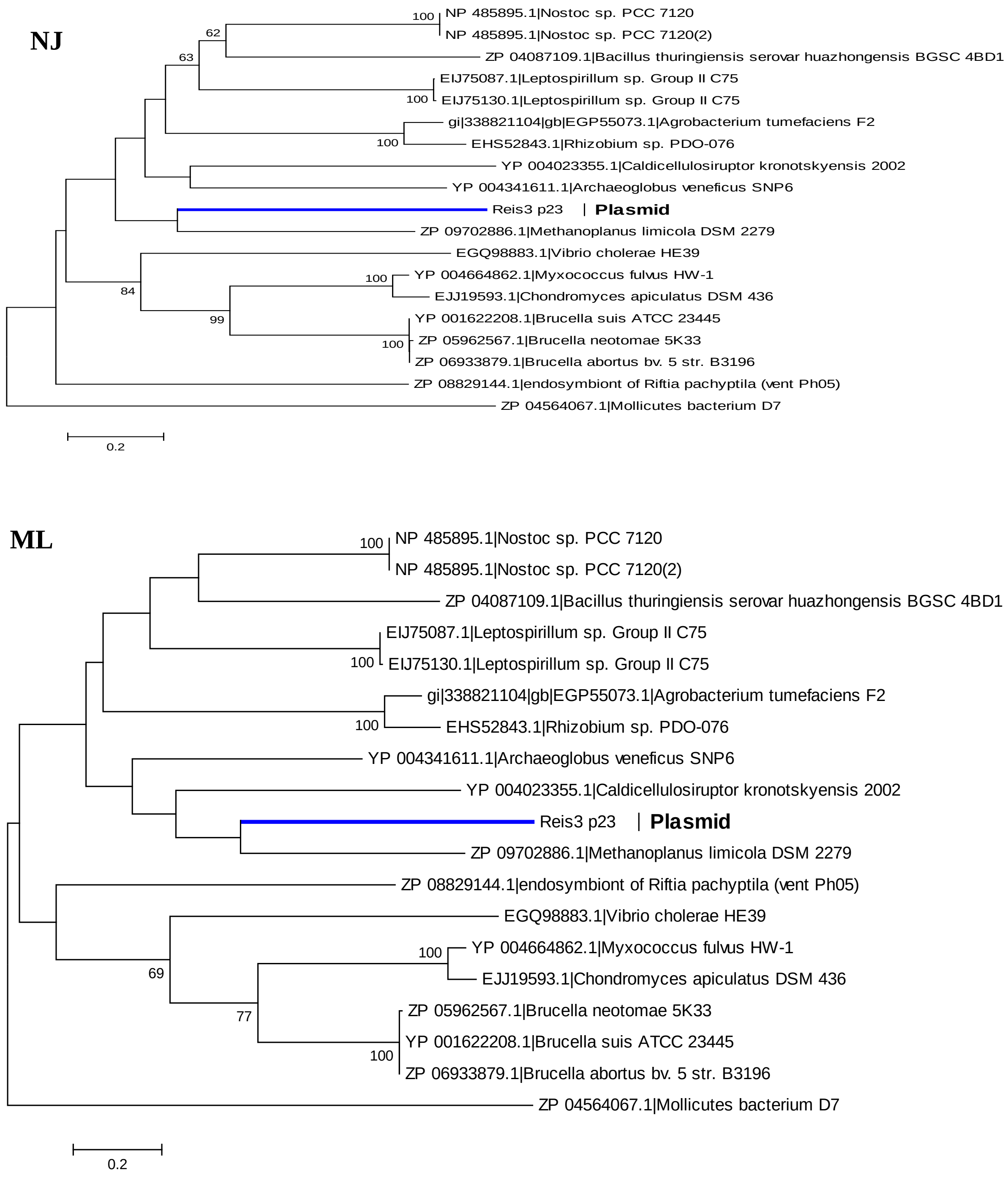

Figure A123. Neighbor-joining (NJ) and maximum likelihood (ML) trees of conserved protein of unknown function. Bootstrap supports higher than or equal to 60% are shown on the branches.

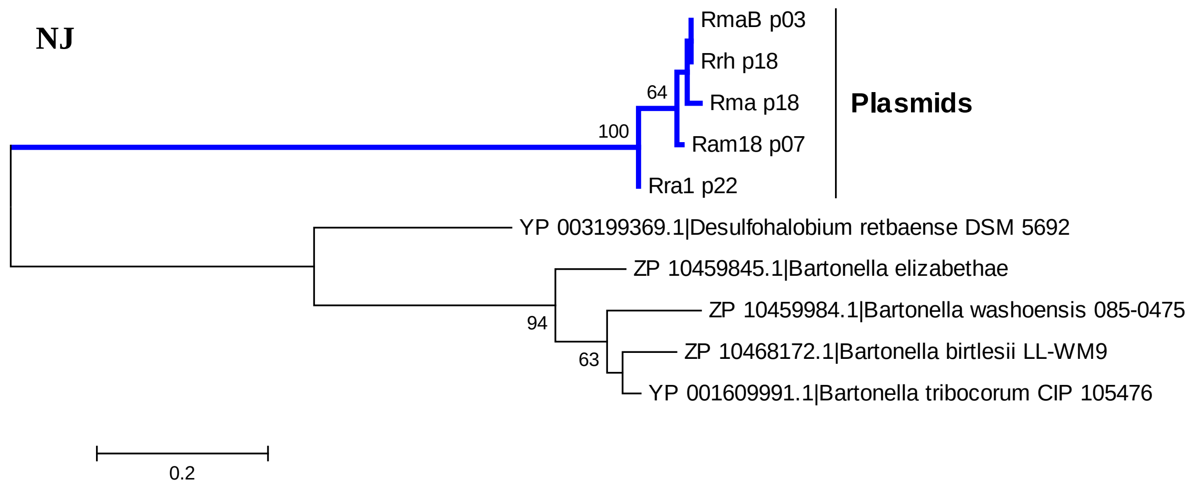

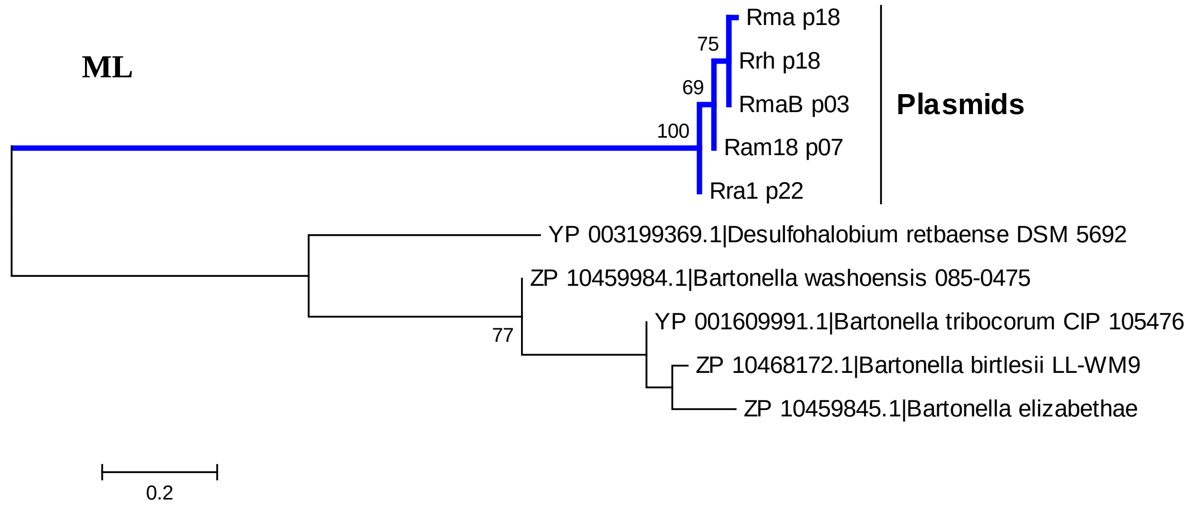

Figure A124. Neighbor-joining (NJ) and maximum likelihood (ML) trees of conserved protein of unknown function. Bootstrap supports higher than or equal to 60% are shown on the branches.

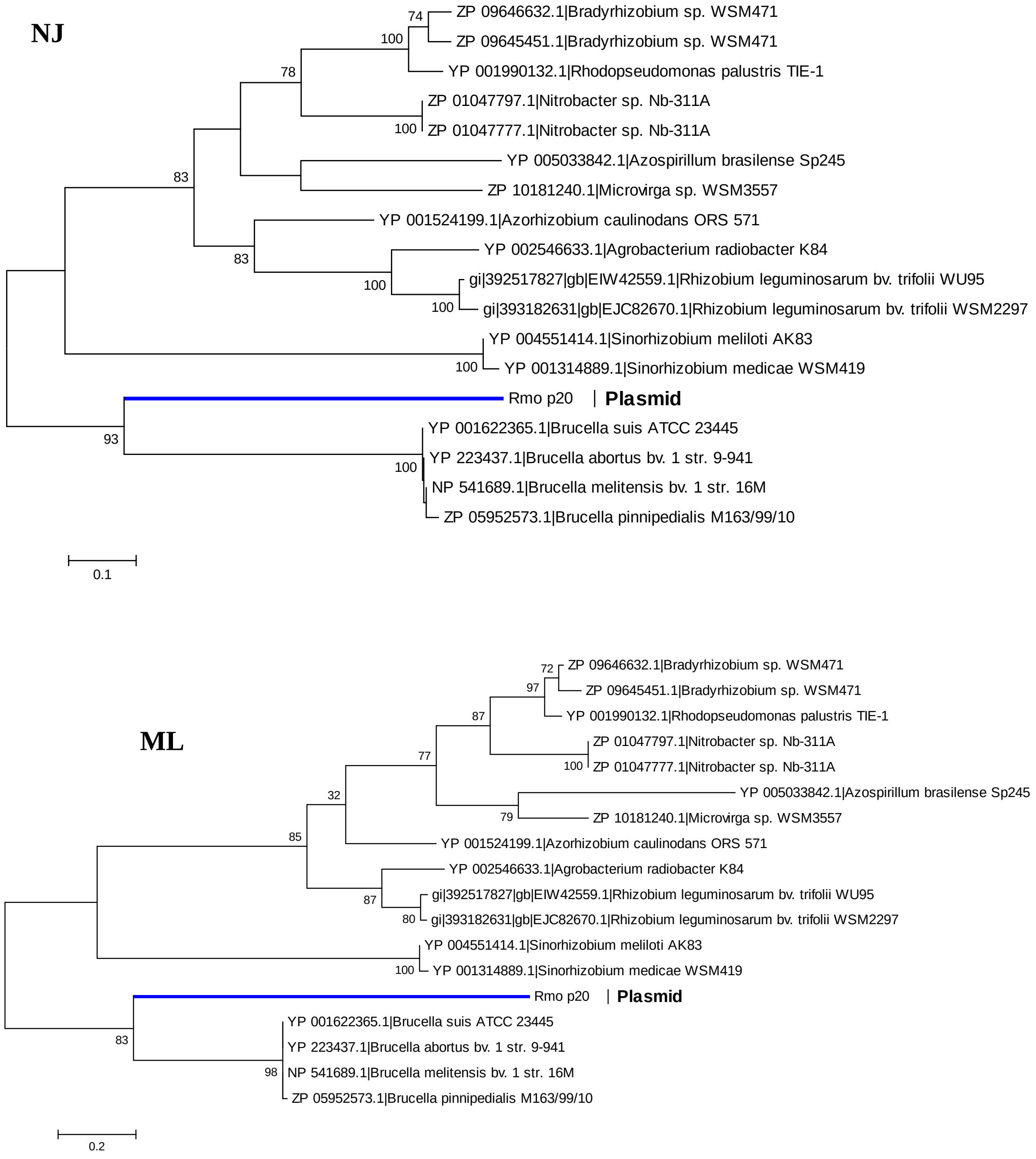

Figure A125. Neighbor-joining (NJ) and maximum likelihood (ML) trees of conserved protein of unknown function. Bootstrap supports higher than or equal to 60% are shown on the branches.

NJ

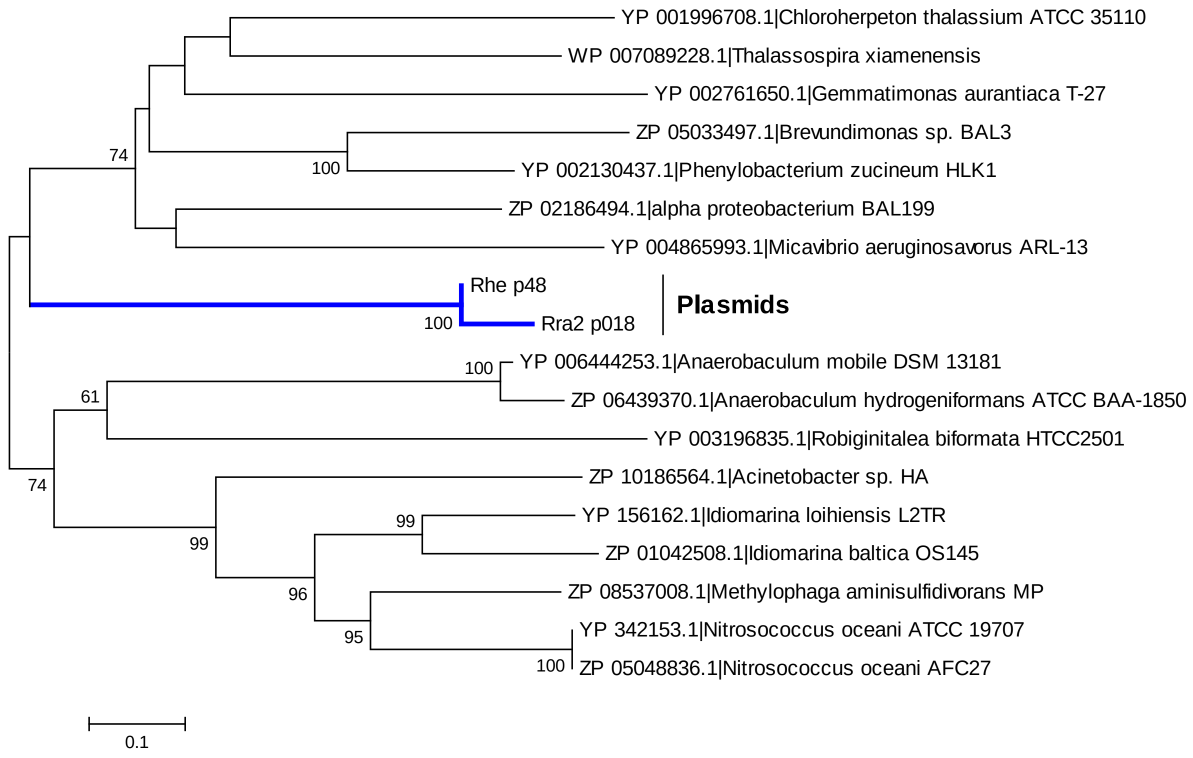

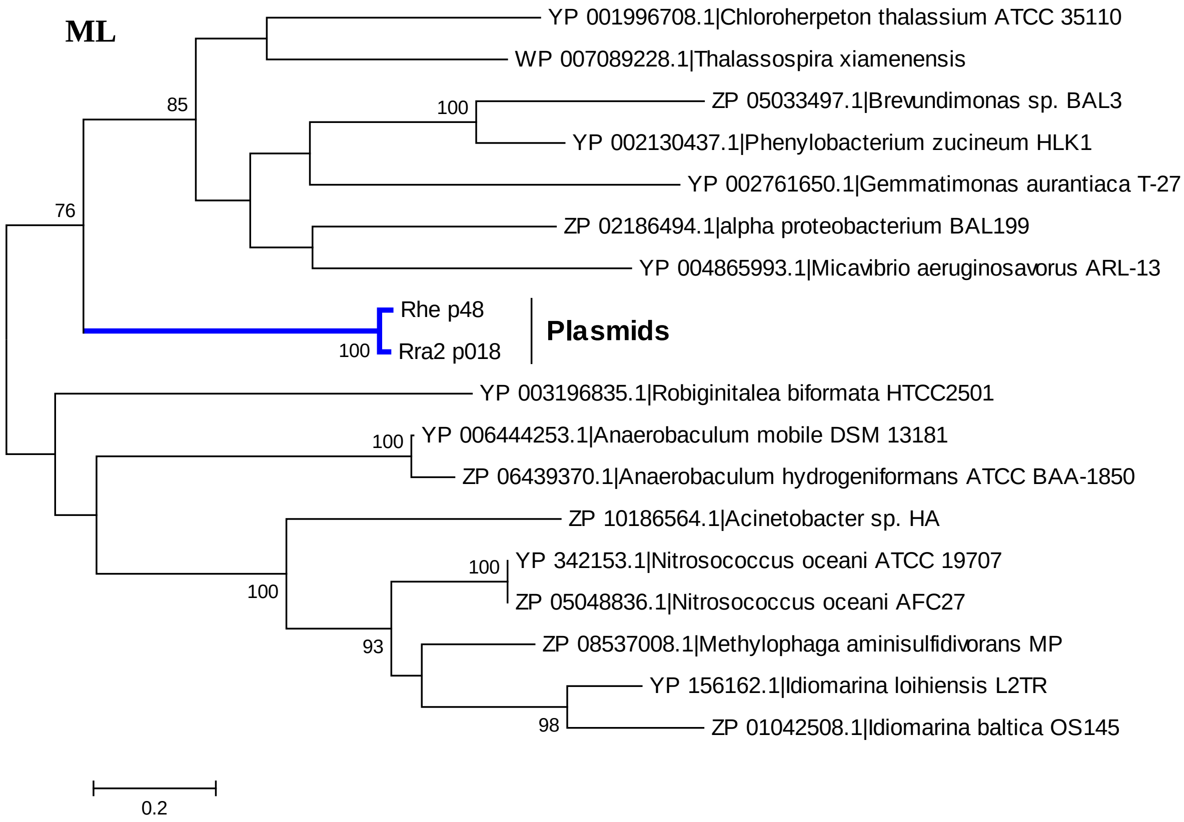
ML
YP 001996708.1|Chloroherpeton thalassium ATCC 35110
WP 007089228.1|Thalassospira xiamenensis
ZP 05033497.1|Brevundimonas sp. BAL3
YP 002130437.1|Phenylobacterium zucineum HLK1
YP 002761650.1|Gemmatimonas aurantiaca T-27
ZP 02186494.1|alpha proteobacterium BAL199
YP 004865993.1|Micavibrio aeruginosavorus ARL-13
Rhe p48
Rra2 p018
Plasmids
YP 003196835.1|Robiginitalea biformata HTCC2501
YP 006444253.1|Anaerobaculum mobile DSM 13181
ZP 06439370.1|Anaerobaculum hydrogeniformans ATCC BAA-1850
ZP 10186564.1|Acinetobacter sp. HA
YP 342153.1|Nitrosococcus oceani ATCC 19707
ZP 05048836.1|Nitrosococcus oceani AFC27
ZP 08537008.1|Methylophaga aminisulfidivorans MP
YP 156162.1|Idiomarina loihiensis L2TR
ZP 01042508.1|Idiomarina baltica OS145
85
100
76
100
100
100
100
93
98
0.2

Figure A126. Neighbor-joining (NJ) and maximum likelihood (ML) trees of DNA-directed DNA polymerase. Bootstrap supports higher than or equal to 60% are shown on the branches.

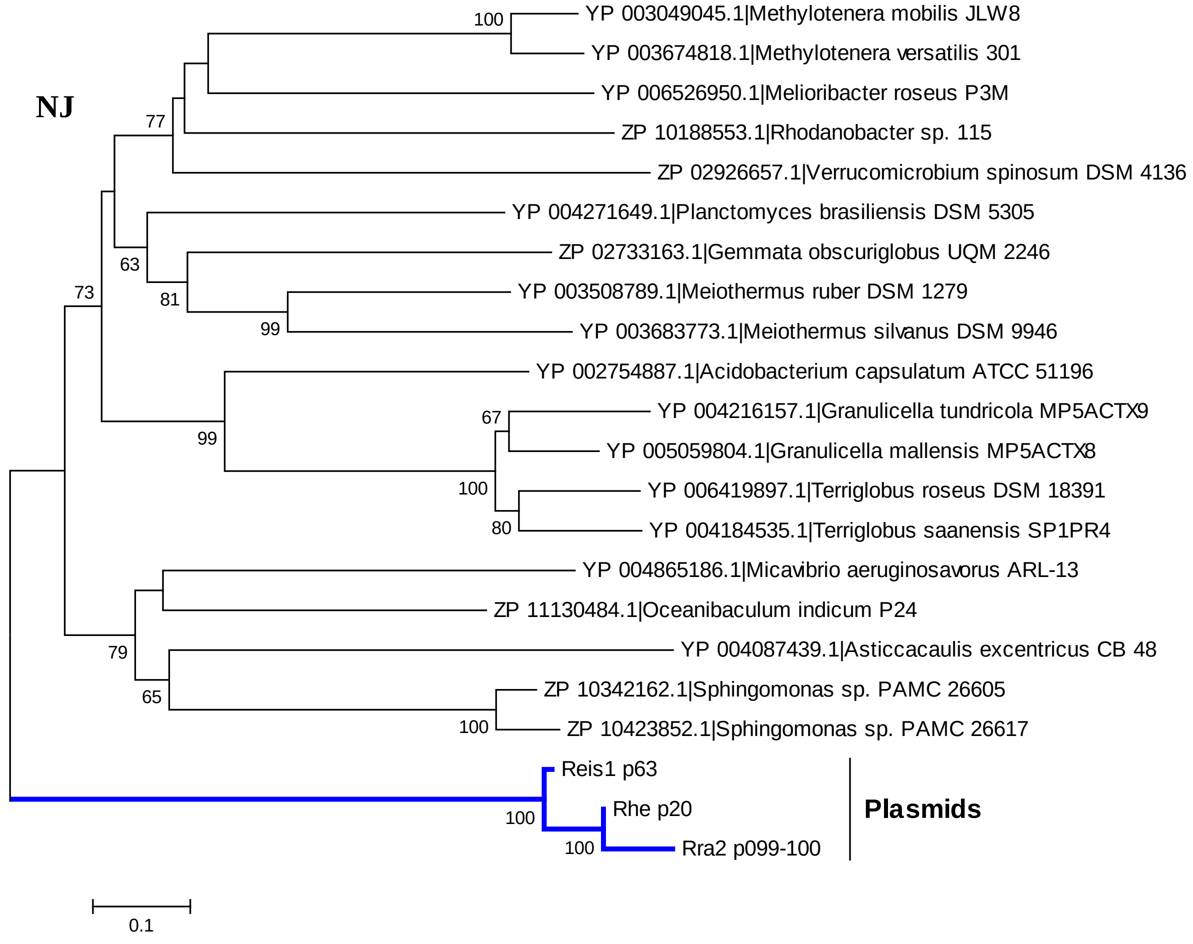

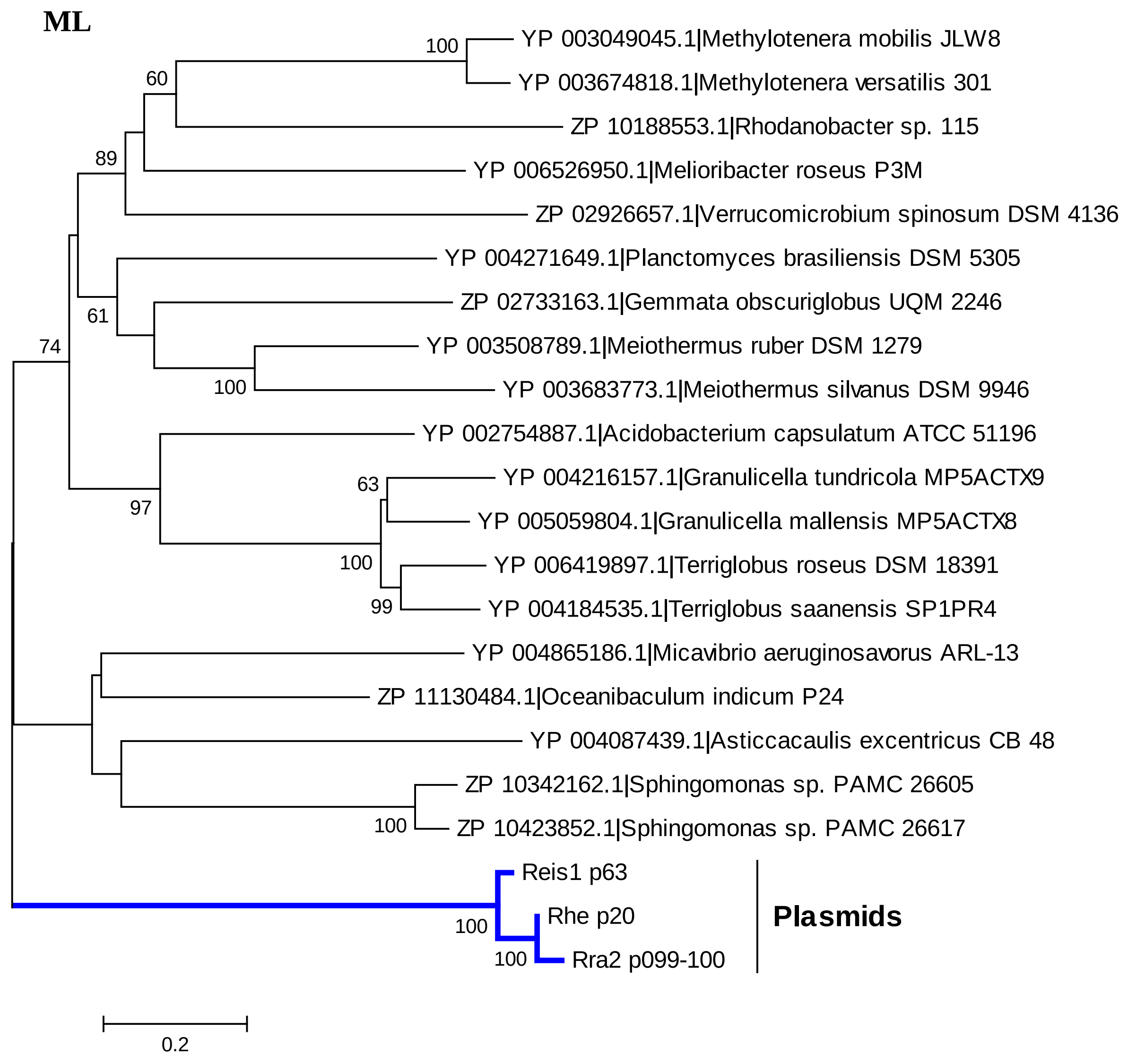
ML
YP 003049045.1|Methylotenera mobilis JLW8
YP 003674818.1|Methylotenera versatilis 301
ZP 10188553.1|Rhodanobacter sp. 115
YP 006526950.1|Melioribacter roseus P3M
ZP 02926657.1|Verrucomicrobium spinosum DSM 4136
YP 004271649.1|Planctomyces brasiliensis DSM 5305
ZP 02733163.1|Gemmata obscuriglobus UQM 2246
YP 003508789.1|Meiothermus ruber DSM 1279
YP 003683773.1|Meiothermus silvanus DSM 9946
YP 002754887.1|Acidobacterium capsulatum ATCC 51196
YP 004216157.1|Granulicella tundricola MP5ACTX9
YP 005059804.1|Granulicella mallensis MP5ACTX8
YP 006419897.1|Terriglobus roseus DSM 18391
YP 004184535.1|Terriglobus saanensis SP1PR4
YP 004865186.1|Micavibrio aeruginosavorus ARL-13
ZP 11130484.1|Oceanibaculum indicum P24
YP 004087439.1|Asticcacaulis excentricus CB 48
ZP 10342162.1|Sphingomonas sp. PAMC 26605
ZP 10423852.1|Sphingomonas sp. PAMC 26617
Reis1 p63
Rhe p20
Rra2 p099-100
Plasmids
100
60
89
61
74
100
97
63
100
99
100
100
100
0.2

Figure A127. Neighbor-joining (NJ) and maximum likelihood (ML) trees of hyaluronidase. Bootstrap supports higher than or equal to 60% are shown on the branches.

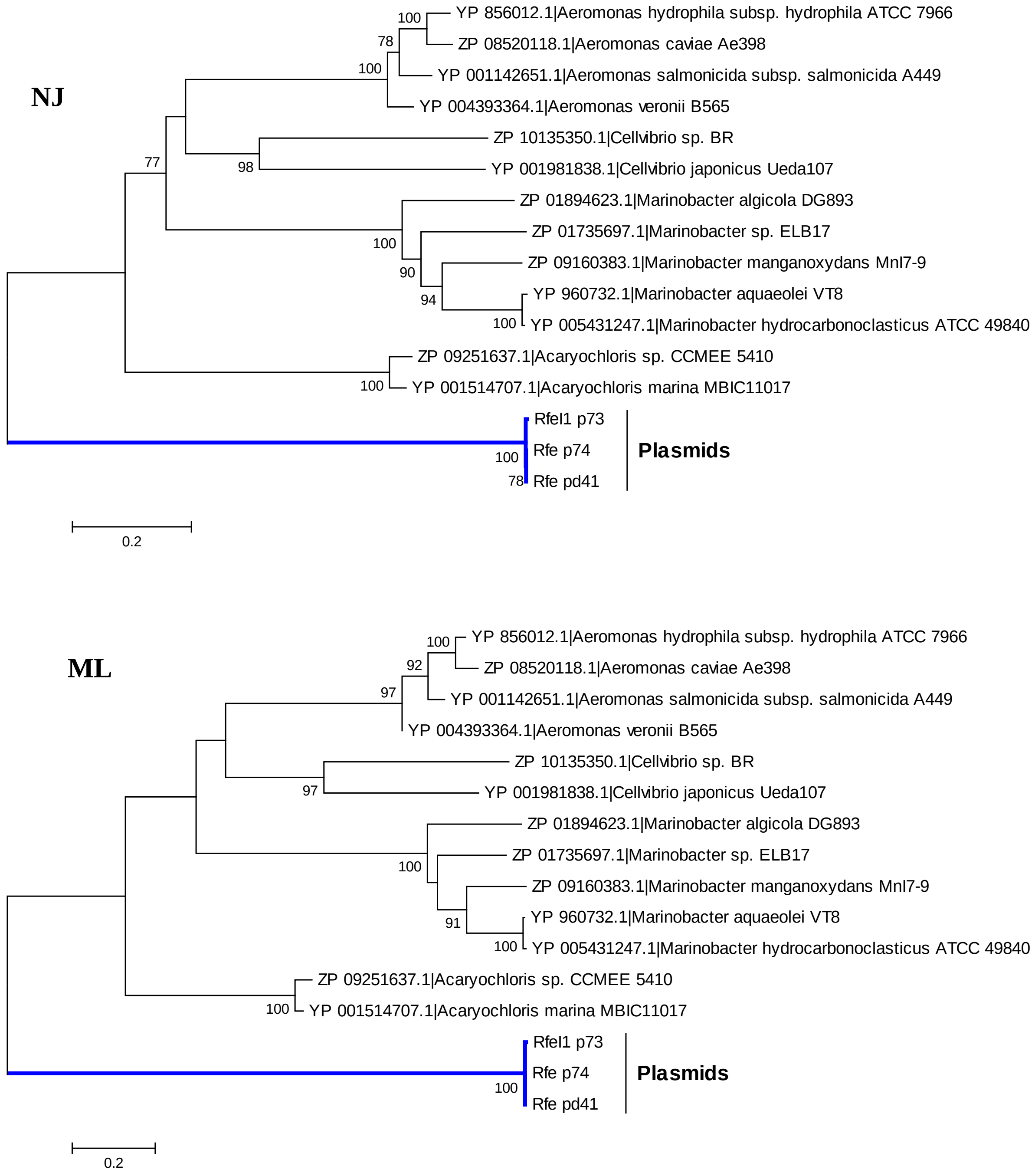

Figure A128. Neighbor-joining (NJ) and maximum likelihood (ML) trees of conserved protein of unknown function. Bootstrap supports higher than or equal to 60% are shown on the branches.

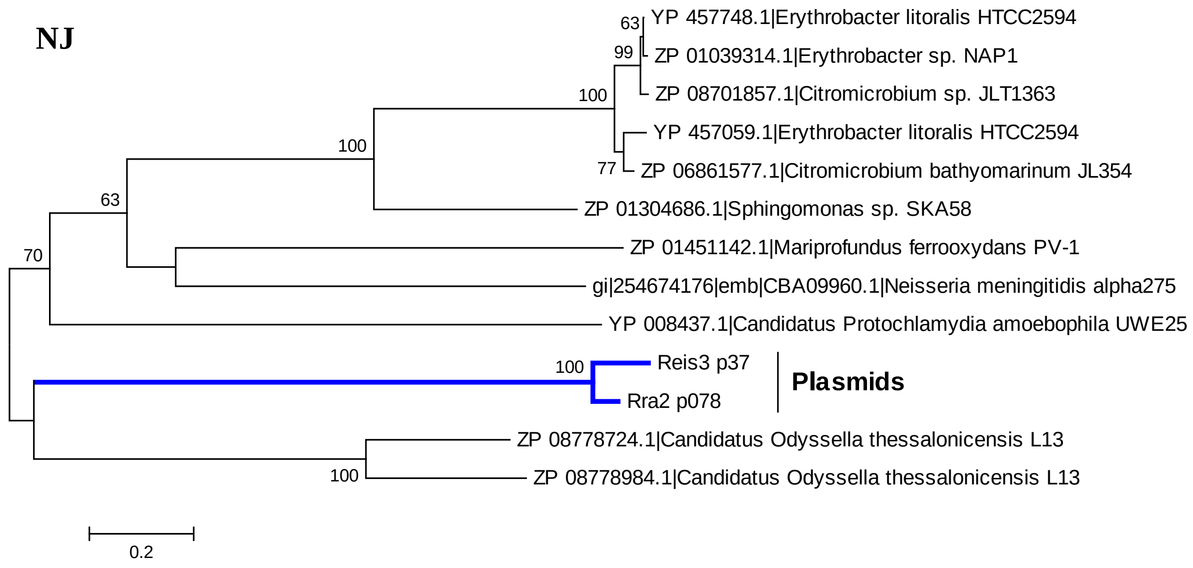

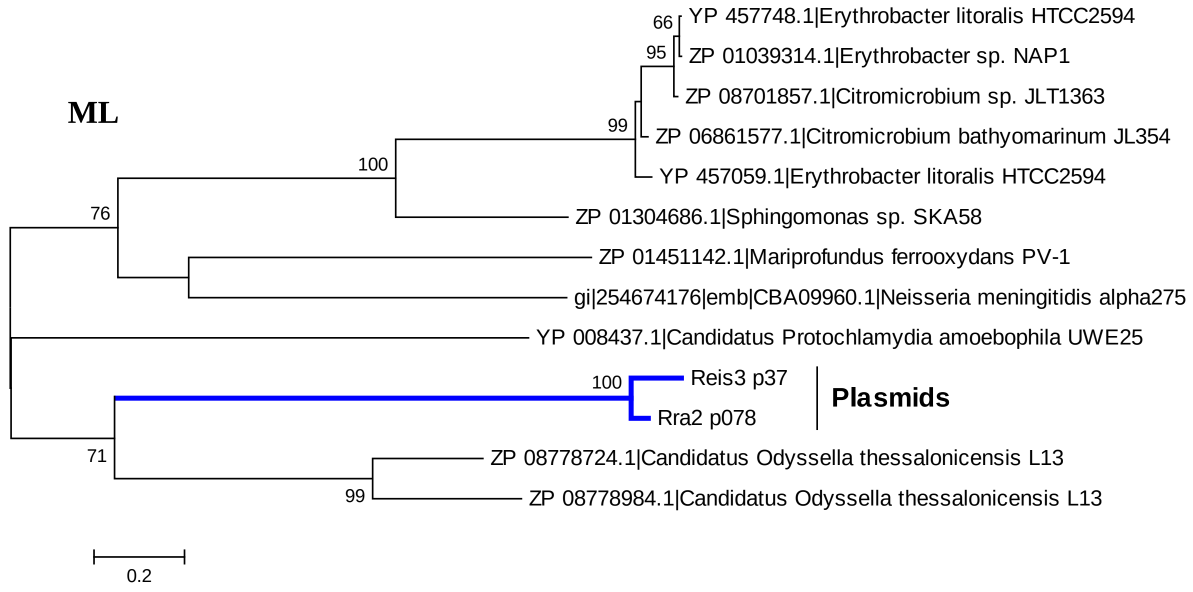

Figure A129. Neighbor-joining (NJ) and maximum likelihood (ML) trees of lon ATP-dependent protease. Bootstrap supports higher than or equal to 60% are shown on the branches.

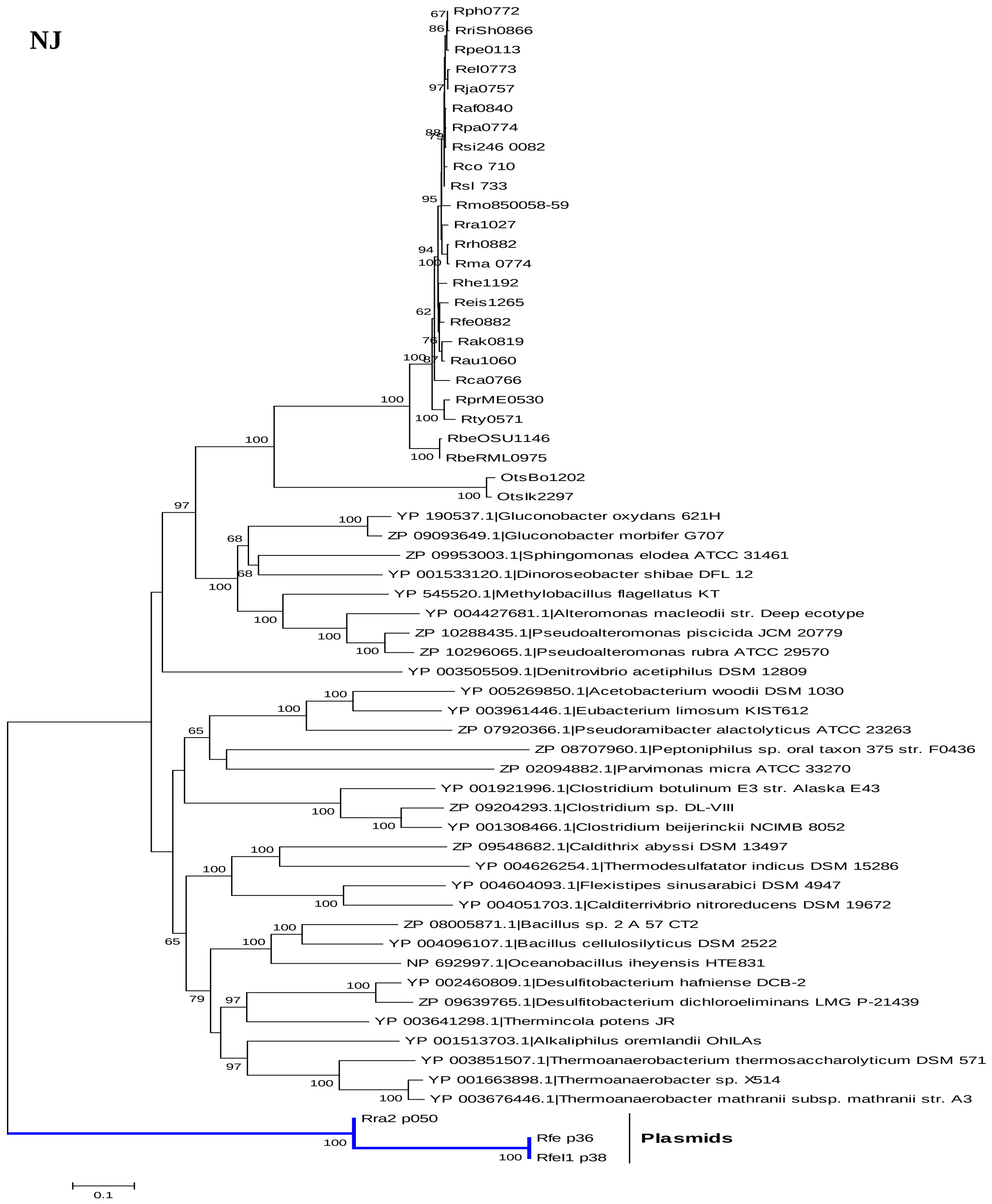

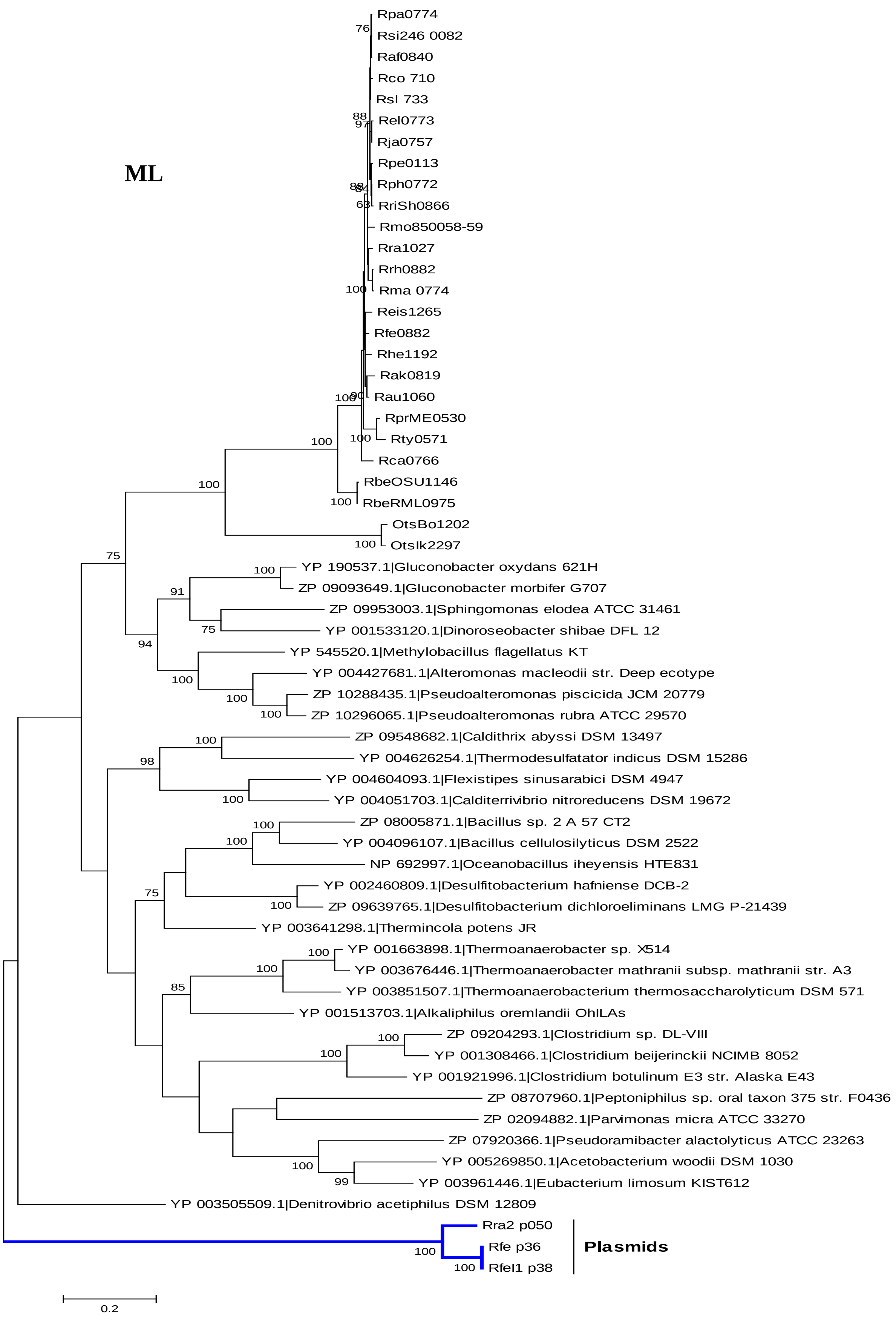
ML
Rpa0774
Rsi246 0082
Raf0840
Rco 710
Rsl 733
Rel0773
Rja0757
Rpe0113
Rph0772
RriSh0866
Rmo850058-59
Rra1027
Rrh0882
Rma 0774
Reis1265
Rfe0882
Rhe1192
Rak0819
Rau1060
RprME0530
Rty0571
Rca0766
RbeOSU1146
RbeRML0975
OtsBo1202
OtsIk2297
YP 190537.1|Gluconobacter oxydans 621H
ZP 09093649.1|Gluconobacter morbifer G707
ZP 09953003.1|Sphingomonas elodea ATCC 31461
YP 001533120.1|Dinoroseobacter shibae DFL 12
YP 545520.1|Methylobacillus flagellatus KT
YP 004427681.1|Alteromonas macleodii str. Deep ecotype
ZP 10288435.1|Pseudoalteromonas piscicida JCM 20779
ZP 10296065.1|Pseudoalteromonas rubra ATCC 29570
ZP 09548682.1|Caldithrix abyssi DSM 13497
YP 004626254.1|Thermodesulfatator indicus DSM 15286
YP 004604093.1|Flexistipes sinusarabici DSM 4947
YP 004051703.1|Calditerrivibrio nitroreducens DSM 19672
ZP 08005871.1|Bacillus sp. 2 A 57 CT2
YP 004096107.1|Bacillus cellulosilyticus DSM 2522
NP 692997.1|Oceanobacillus iheyensis HTE831
YP 002460809.1|Desulfitobacterium hafniense DCB-2
ZP 09639765.1|Desulfitobacterium dichloroeliminans LMG P-21439
YP 003641298.1|Thermincola potens JR
YP 001663898.1|Thermoanaerobacter sp. X514
YP 003676446.1|Thermoanaerobacter mathranii subsp. mathranii str. A3
YP 003851507.1|Thermoanaerobacterium thermosaccharolyticum DSM 571
YP 001513703.1|Alkaliphilus oremlandii OhILAs
ZP 09204293.1|Clostridium sp. DL-VIII
YP 001308466.1|Clostridium beijerinckii NCIMB 8052
YP 001921996.1|Clostridium botulinum E3 str. Alaska E43
ZP 08707960.1|Peptoniphilus sp. oral taxon 375 str. F0436
ZP 02094882.1|Parvimonas micra ATCC 33270
ZP 07920366.1|Pseudoramibacter alactolyticus ATCC 23263
YP 005269850.1|Acetobacterium woodii DSM 1030
YP 003961446.1|Eubacterium limosum KIST612
YP 003505509.1|Denitrovibrio acetiphilus DSM 12809
Rra2 p050
Rfe p36
RfeI1 p38
Plasmids
0.2

Figure A130. Neighbor-joining (NJ) and maximum likelihood (ML) trees of conserved protein of unknown function. Bootstrap supports higher than or equal to 60% are shown on the branches.

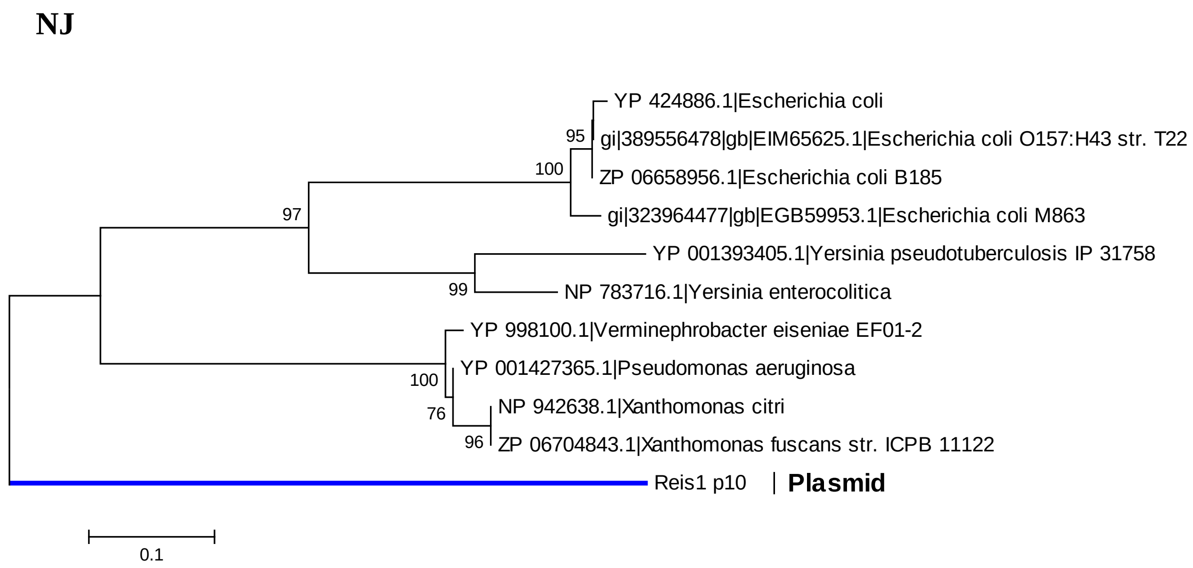

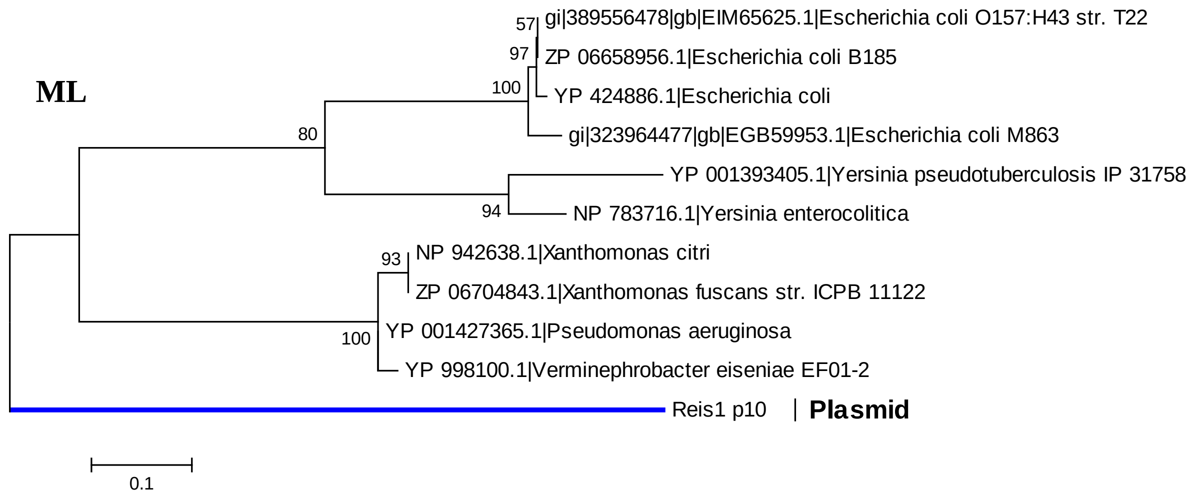

Figure A131. Neighbor-joining (NJ) and maximum likelihood (ML) trees of conserved protein of unknown function. Bootstrap supports higher than or equal to 60% are shown on the branches.

NJ

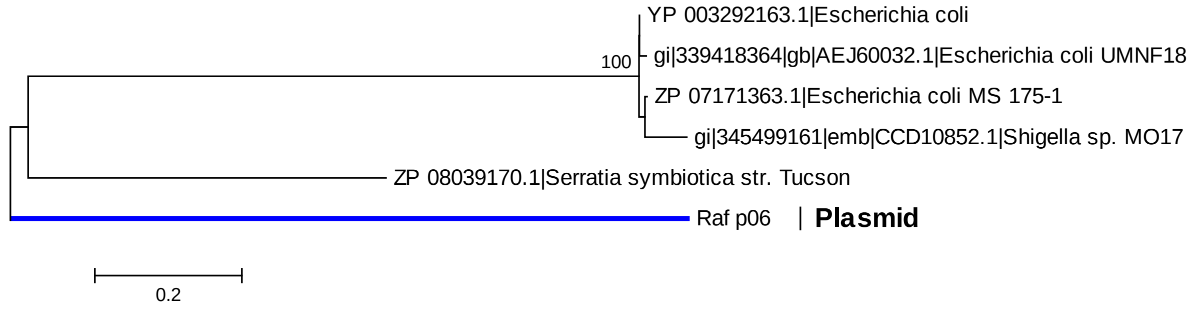

ML

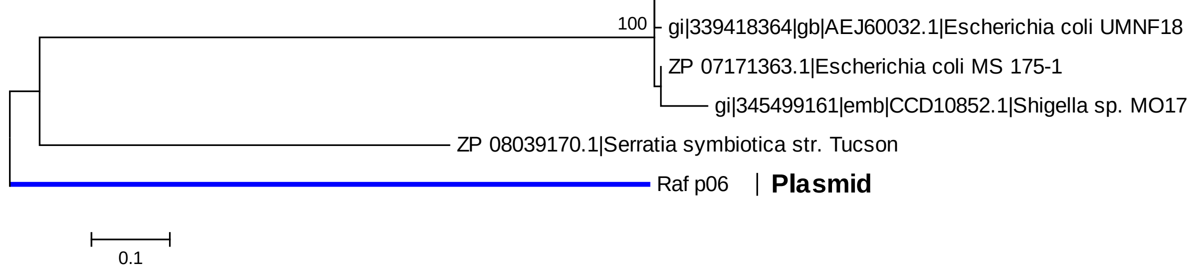

Figure A132. Neighbor-joining (NJ) and maximum likelihood (ML) trees of conserved protein of unknown function. Bootstrap supports higher than or equal to 60% are shown on the branches.

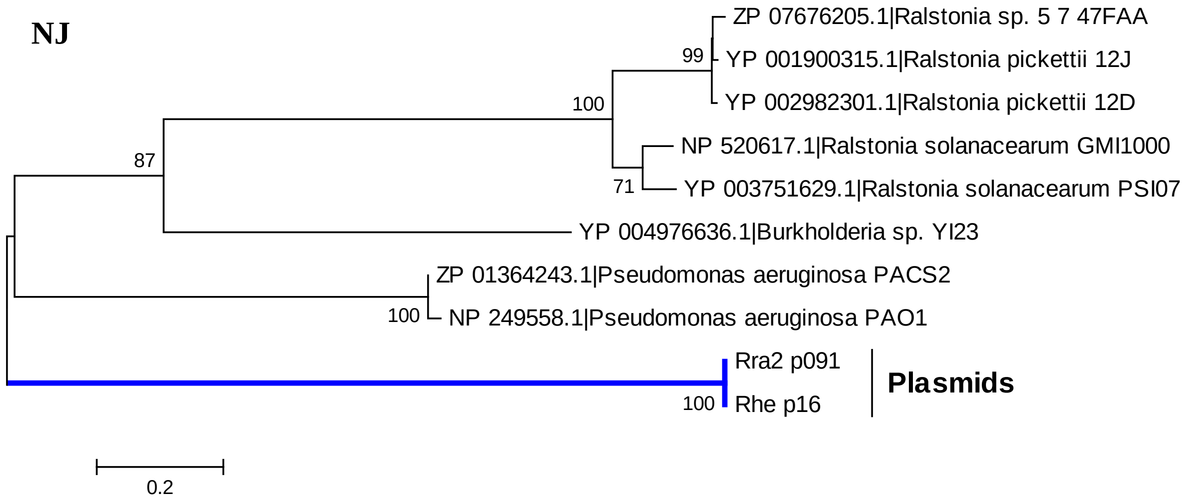

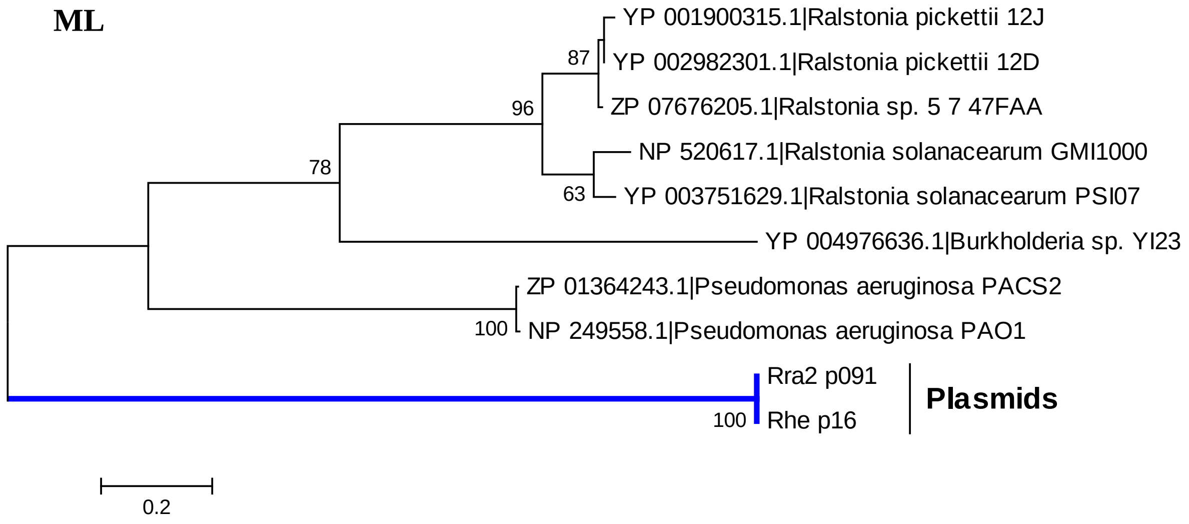

Figure A133. Neighbor-joining (NJ) and maximum likelihood (ML) trees of transposase. Bootstrap supports higher than or equal to 60% are shown on the branches.

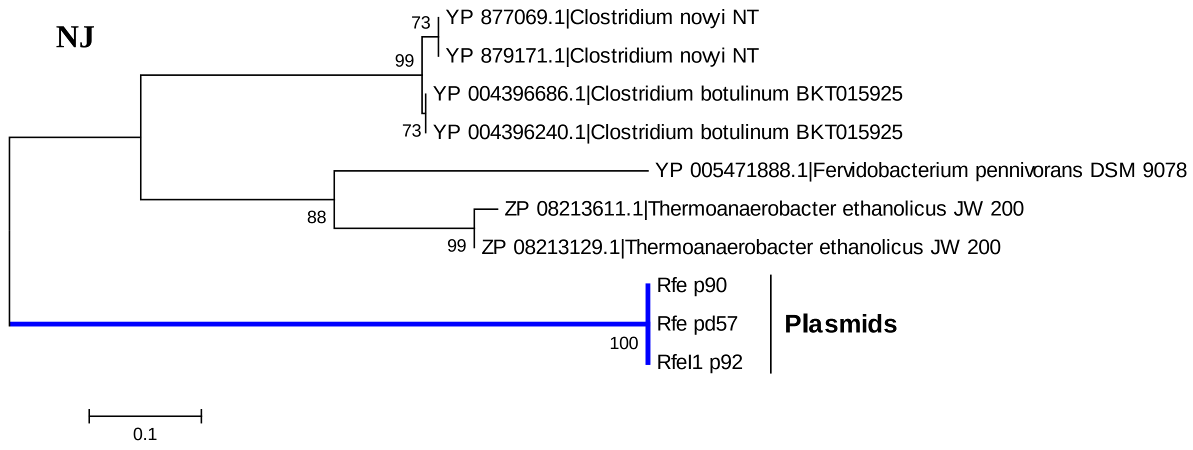

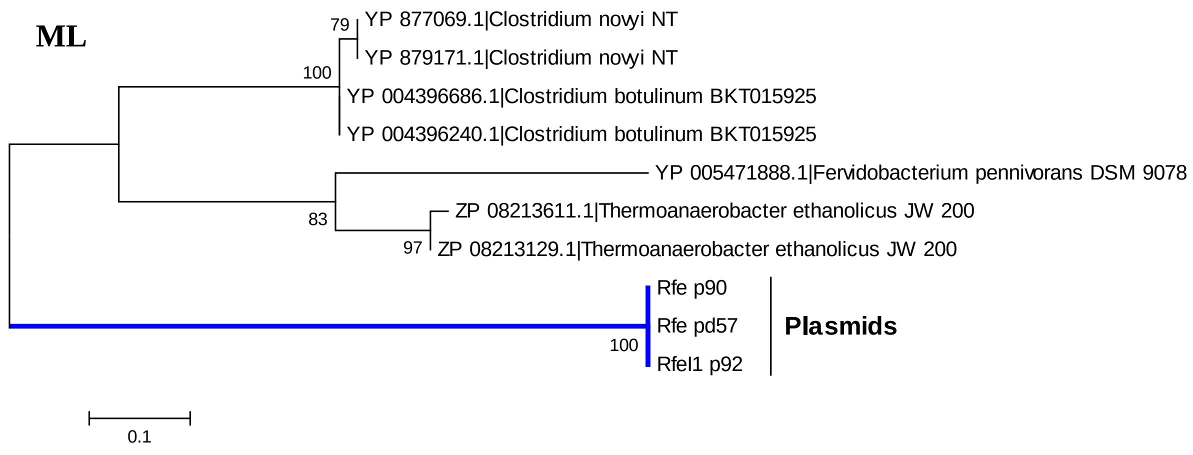

Figure A134. Neighbor-joining (NJ) and maximum likelihood (ML) trees of transposase containing DDE_Tnp_1_3 domain. Bootstrap supports higher than or equal to 60% are shown on the branches.

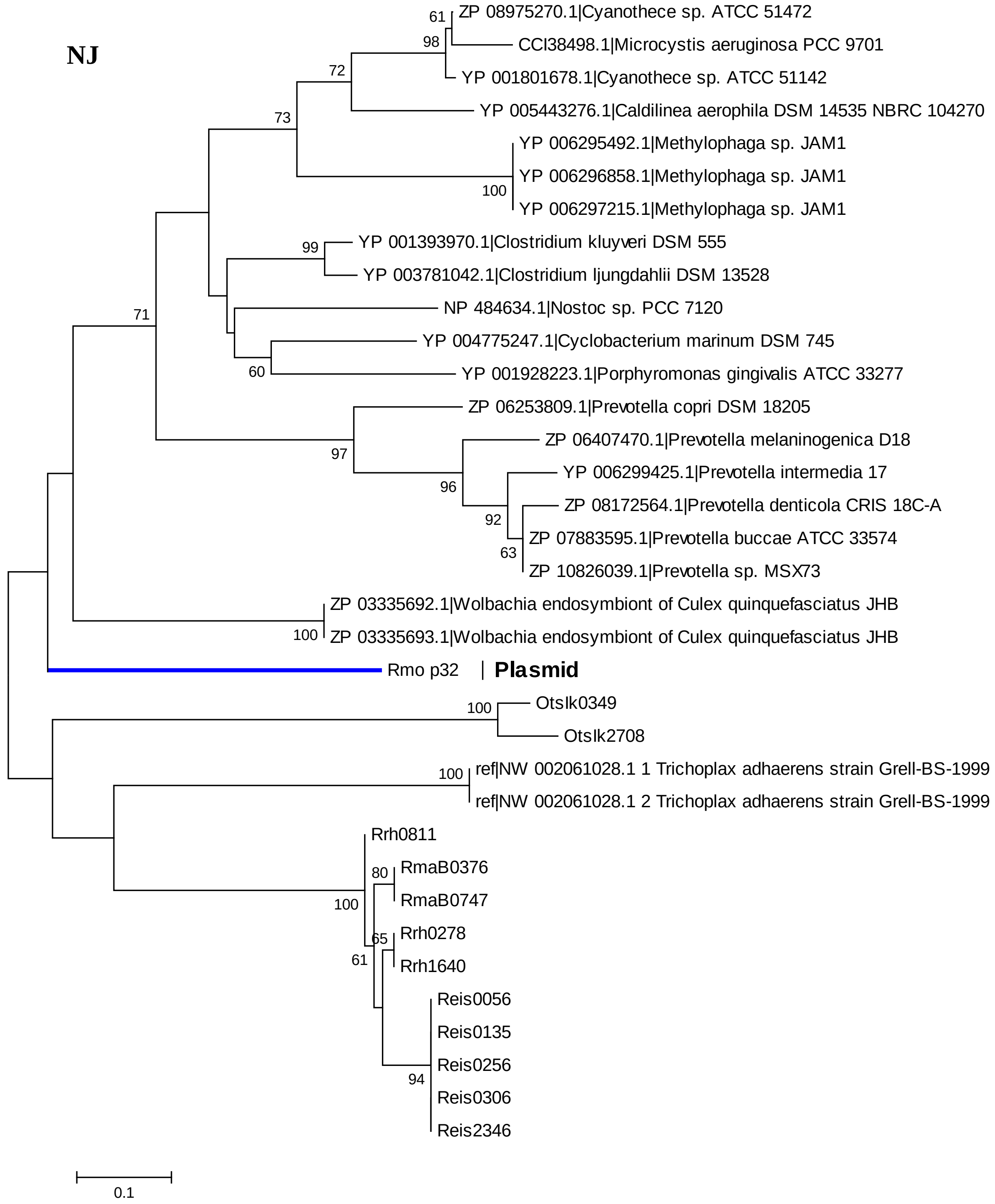

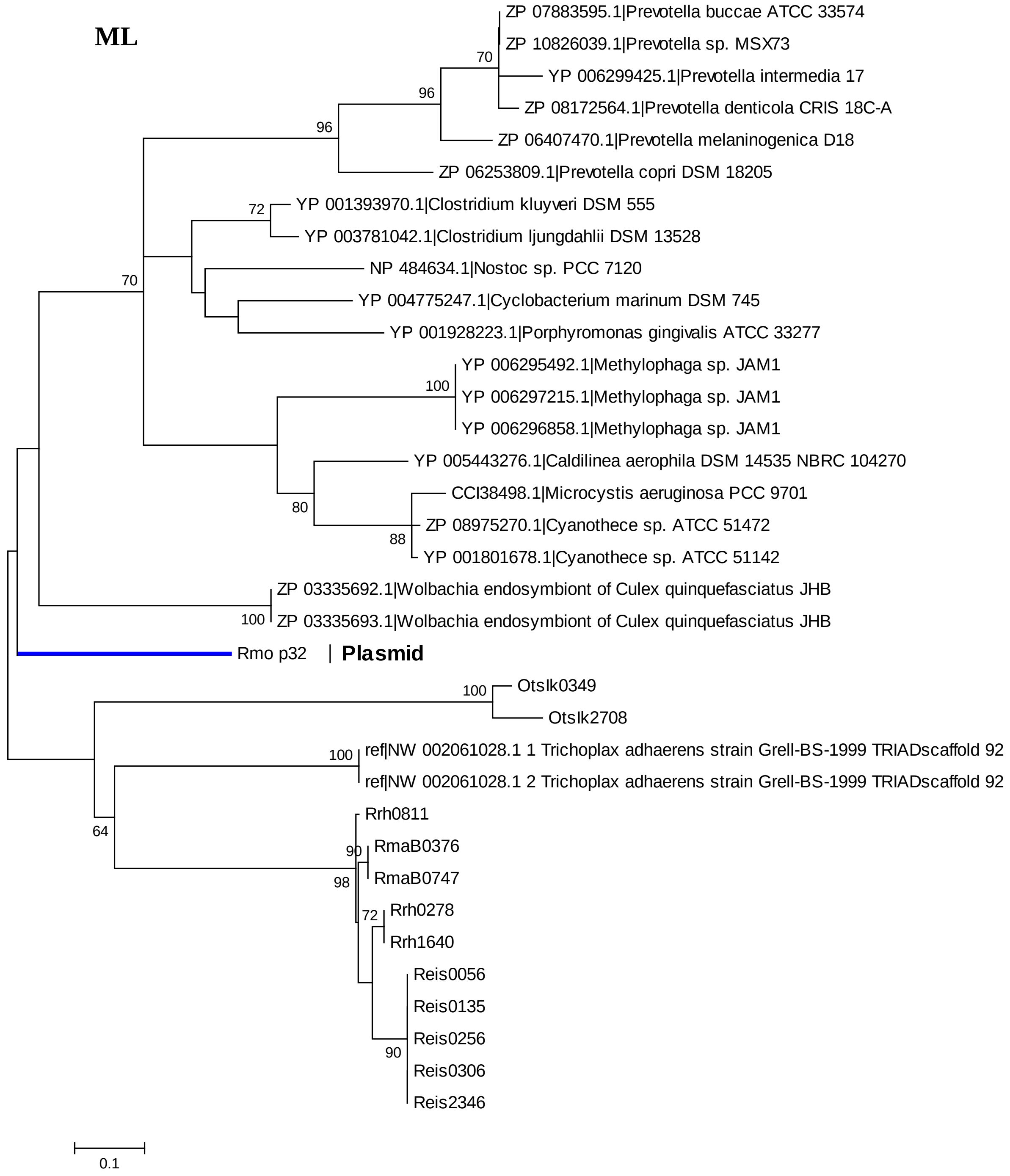

ML
ZP 07883595.1|Prevotella buccae ATCC 33574
ZP 10826039.1|Prevotella sp. MSX73
YP 006299425.1|Prevotella intermedia 17
ZP 08172564.1|Prevotella denticola CRIS 18C-A
ZP 06407470.1|Prevotella melaninogenica D18
ZP 06253809.1|Prevotella copri DSM 18205
YP 001393970.1|Clostridium kluyveri DSM 555
YP 003781042.1|Clostridium ljungdahlii DSM 13528
NP 484634.1|Nostoc sp. PCC 7120
YP 004775247.1|Cyclobacterium marinum DSM 745
YP 001928223.1|Porphyromonas gingivalis ATCC 33277
YP 006295492.1|Methylophaga sp. JAM1
YP 006297215.1|Methylophaga sp. JAM1
YP 006296858.1|Methylophaga sp. JAM1
YP 005443276.1|Caldilinea aerophila DSM 14535 NBRC 104270
CCI38498.1|Microcystis aeruginosa PCC 9701
ZP 08975270.1|Cyanothece sp. ATCC 51472
YP 001801678.1|Cyanothece sp. ATCC 51142
ZP 03335692.1|Wolbachia endosymbiont of Culex quinquefasciatus JHB
ZP 03335693.1|Wolbachia endosymbiont of Culex quinquefasciatus JHB
Rmo p32
Plasmid
OtsIk0349
OtsIk2708
ref|NW 002061028.1 1 Trichoplax adhaerens strain Grell-BS-1999 TRIADscaffold 92
ref|NW 002061028.1 2 Trichoplax adhaerens strain Grell-BS-1999 TRIADscaffold 92
Rrh0811
RmaB0376
RmaB0747
Rrh0278
Rrh1640
Reis0056
Reis0135
Reis0256
Reis0306
Reis2346
70
96
96
72
70
100
80
88
100
100
100
90
98
72
90
64
0.1

Figure A135. Neighbor-joining (NJ) and maximum likelihood (ML) trees of conjugative transfer protein containing TraK domain. Bootstrap supports higher than or equal to 60% are shown on the branches.

NJ

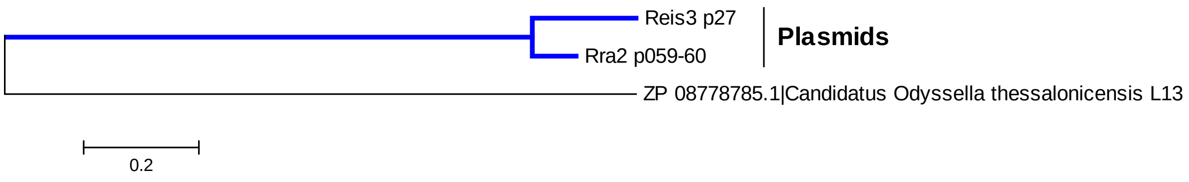

ML

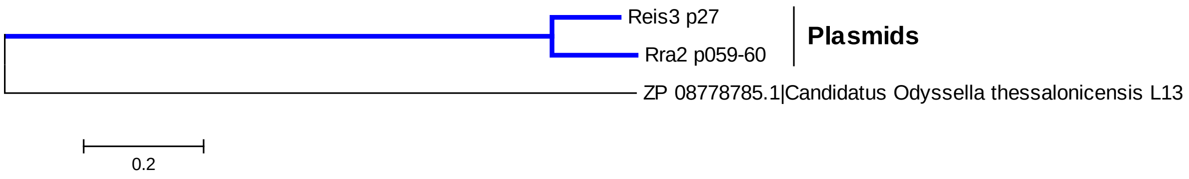

Figure A136. Neighbor-joining (NJ) and maximum likelihood (ML) trees of conjugative transfer protein containing TraK domain. Bootstrap supports higher than or equal to 60% are shown on the branches.

NJ

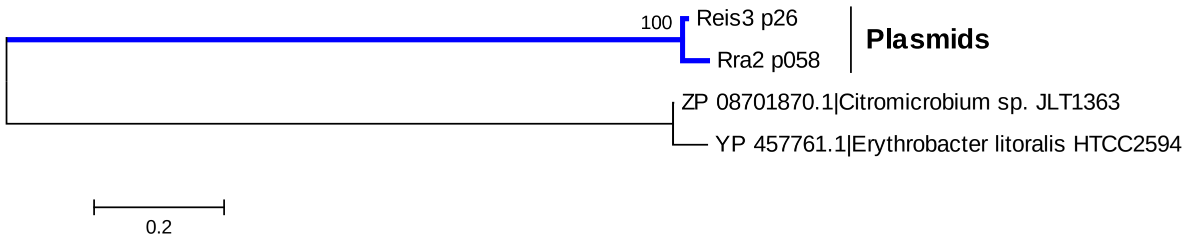

ML

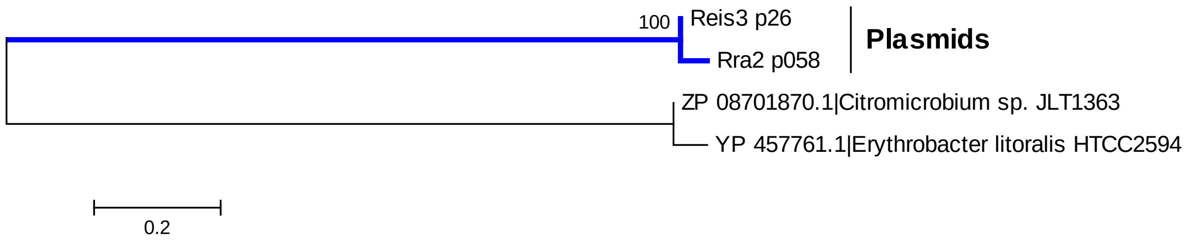

Supplement: S3 File — (PDF) [file pone.0147492.s003.pdf]
